# Bayesian Modeling of the Yeast SH3 Domain Interactome Predicts Spatiotemporal Dynamics of Endocytosis Proteins

**DOI:** 10.1371/journal.pbio.1000218

**Published:** 2009-10-20

**Authors:** Raffi Tonikian, Xiaofeng Xin, Christopher P. Toret, David Gfeller, Christiane Landgraf, Simona Panni, Serena Paoluzi, Luisa Castagnoli, Bridget Currell, Somasekar Seshagiri, Haiyuan Yu, Barbara Winsor, Marc Vidal, Mark B. Gerstein, Gary D. Bader, Rudolf Volkmer, Gianni Cesareni, David G. Drubin, Philip M. Kim, Sachdev S. Sidhu, Charles Boone

**Affiliations:** 1 Terrence Donnelly Center for Cellular and Biomolecular Research, Banting and Best Department of Medical Research, University of Toronto, Toronto, Ontario, Canada; 2 Department of Molecular Genetics, University of Toronto, Toronto, Ontario, Canada; 3 Department of Molecular and Cell Biology, University of California, Berkeley, Berkeley, California, United States of America; 4 Institute of Medical Immunology, Charité-Universitätsmedizin Berlin, Berlin, Germany; 5 Department of Biology, University of Rome Tor Vergata, Rome, Italy; 6 Department of Cell Biology, University of Calabria, Rende, Italy; 7 Department of Molecular Biology, Genentech, South San Francisco, California, United States of America; 8 Center for Cancer Systems Biology (CCSB), Department of Cancer Biology, Dana-Farber Cancer Institute and Department of Genetics, Harvard Medical School, Boston, Massachusetts, United States of America; 9 CNRS et Université de Strasbourg UMR7156, Génétique moléculaire, Génomique et Microbiologie, Strasbourg, France; 10 Department of Molecular Biophysics and Biochemistry, Yale University, New Haven, Connecticut, United States of America; 11 Program in Computational Biology and Bioinformatics, Yale University, New Haven, Connecticut, United States of America; 12 Department of Computer Science, Yale University, New Haven, Connecticut, United States of America; 13 Research Institute “Fondazione Santa Lucia”, Rome, Italy; 14 Department of Protein Engineering, Genentech, South San Francisco, California, United States of America; University of Massachusetts Medical School, United States of America

## Abstract

SH3 domains are peptide recognition modules that mediate the assembly of diverse biological complexes. We scanned billions of phage-displayed peptides to map the binding specificities of the SH3 domain family in the budding yeast, *Saccharomyces cerevisiae*. Although most of the SH3 domains fall into the canonical classes I and II, each domain utilizes distinct features of its cognate ligands to achieve binding selectivity. Furthermore, we uncovered several SH3 domains with specificity profiles that clearly deviate from the two canonical classes. In conjunction with phage display, we used yeast two-hybrid and peptide array screening to independently identify SH3 domain binding partners. The results from the three complementary techniques were integrated using a Bayesian algorithm to generate a high-confidence yeast SH3 domain interaction map. The interaction map was enriched for proteins involved in endocytosis, revealing a set of SH3-mediated interactions that underlie formation of protein complexes essential to this biological pathway. We used the SH3 domain interaction network to predict the dynamic localization of several previously uncharacterized endocytic proteins, and our analysis suggests a novel role for the SH3 domains of Lsb3p and Lsb4p as hubs that recruit and assemble several endocytic complexes.

## Abstract 2

A genome-scale specificity and interaction map for yeast SH3 domain-containing proteins reveal how family members show selective binding to target proteins and predicts the dynamic localization of new candidate endocytosis proteins.

## Author Summary

Significant diversity exists in protein structure and function, yet certain structural domains are used repeatedly across species to execute similar functions. The SH3 domain is one such common structural domain. It is found in signaling proteins and mediates protein–protein interactions by binding to short peptide sequences generally composed of proline. To investigate both the generality and selectivity of peptide binding by SH3 domains, we examined peptide specificity for almost all SH3 domains encoded within the proteome of the budding yeast, *Saccharomyces cerevisiae*, using a range of experimental methods. We found that although most of the intrinsic binding specificity for SH3 domains can be summarized by the two previously described canonical binding modes, each individual SH3 domain that we studied utilizes unique features of its cognate ligand to achieve binding selectivity. Moreover, some domains exhibit binding specificities that are distinct from the two canonical classes. We integrated peptide-SH3 domain binding data from three complementary screening techniques using a Bayesian statistical model to generate a protein–protein interaction network for the budding yeast SH3 domain family. This network was highly enriched in endocytosis proteins and their interactions. By examining these interactions in detail, we show that our SH3 domain network can be used to predict the temporal localization of several previously uncharacterized proteins to dynamic complexes that orchestrate the process of endocytosis.

## Introduction

Families of peptide recognition modules (PRMs), such as PDZ (PSD-95/Discs-large/ZO-1), SH2 (Src homology 2), and SH3 (Src homology 3) domains bind peptide motifs within proteins to mediate protein–protein interactions required for the assembly of stable or transient biological complexes [1]. Thus, PRMs function to dynamically orchestrate biological pathways [1]. PRM family members can be identified directly from whole-genome sequences; therefore, it is possible to explore the recognition specificity of entire families using a variety of different experimental approaches [2],[3]. Here, we explore the potential for mapping SH3 domain protein interaction networks by a Bayesian integration of results from three complementary experimental screening approaches: phage display, peptide array, and yeast two-hybrid analysis.

In general, PRMs engage in protein–protein interactions by recognizing a core motif common to a domain family as well as additional ligand features that are more specific for each family member as is the case for PDZ domains [3]. Initial studies determined that SH3 domains bind to proline-rich sequences containing a core PXXP motif (where X is any amino acid) flanked by a positively charged residue [4],[5]. Class I domains bind to ligands conforming to the consensus +XXPXXP (where + is either arginine or lysine), and do so in an orientation opposite to that of class II domains, which recognize PXXPX+ motifs [6],[7]. More recently, a number of alternative SH3 domain binding motifs have been identified, highlighting a wider breadth of SH3 specificities [8]–[11].

A general genome-wide analysis of PRMs would involve defining all the domains from their primary sequence, profiling their ligand-binding specificities in detail, predicting natural ligands for each domain, and mapping large-scale protein–protein interaction networks for each domain family. Here, we present the first high-resolution analysis of the yeast SH3 domain family. First, we used large-scale phage-displayed peptide libraries and extensive sequencing to generate high-resolution binding profiles, which we show accurately represent binding specificity across multiple SH3 domain ligand positions. Second, we used the resulting specificity profiles to identify putative interactions in the yeast proteome, which were subsequently confirmed using oriented synthetic peptide arrays. Third, we conducted large-scale yeast two-hybrid screens to generate a physical interaction network mediated by the set of yeast SH3 domains. Finally, the datasets were integrated using Bayesian networks to generate a global SH3 domain interaction map in yeast.

Applying the integrated probabilistic network revealed an intricate array of SH3-mediated interactions amongst proteins that make up the endocytic machinery. Investigation and comparison of the dynamics of protein localization within this network showed that the modular network predictions of the spatiotemporal dynamics of several novel endocytotic components were correct. In particular, our analysis predicts that the SH3 domains from Lsb3p and Lsb4p interact with multiple endocytic proteins and therefore might act as hubs to cluster these proteins at sites of endocytosis.

## Results

### Yeast SH3 Domain Specificity Map

We used peptide phage display to conduct a large-scale analysis of yeast SH3 domain specificity. We cloned DNA fragments encoding all 27 unique yeast SH3 domains using boundaries taken as the union of the domain lengths identified by three domain detection tools, BLAST [12], PFAM [13], and SMART [14], with an additional ten amino acids included on either side of the overlapping domain region to facilitate cloning (Table S1). We expressed the domains in bacteria as proteins fused to the C-terminus of glutathione *S*-transferase (GST) and purified 24 out of 27 fusion proteins in a stable, soluble form. For two of the three recalcitrant domains, the C-terminal domain of Bem1p (Bem1-2) and the Bud14p domain, we extended the sequence boundaries by examining the conservation of the domain regions across diverse fungal species. Based on this analysis, the domain boundaries for these two SH3 domains were extended, enabling the isolation of stable GST fusion proteins (Table S2 and Text S1). The third recalcitrant domain, the N-terminal domain of Sla1p (Sla1-1), could only be purified in tandem with Sla1-2, and we denoted the dual domain protein as Sla1-1/2.

The GST-SH3 domain fusion proteins were used as targets in binding selections with a combination of random and biased peptide–phage libraries. We were successful in obtaining ligands for all SH3 domains except Bud14 and Cdc25, and we isolated a total of 1,871 unique peptides. These results extend results from our previous study [2] and represent nearly an 8-fold increase in binding data. The set of aligned ligands for each domain was used to compile a position weight matrix (PWM), which captures the frequency of amino acid preferences at each ligand position. Some ligand sets contained two distinct groups of ligands, and for these, two separate PWMs were compiled (see below). From each PWM, a sequence logo [15] was generated to graphically represent the specificity at each amino acid position in each ligand set.

**Figure 1:**
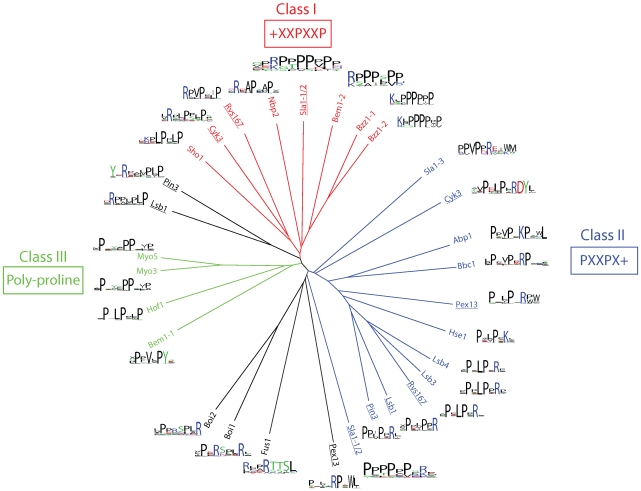
Endogenous specificity map for the yeast SH3 domain family. PWMs were generated using phage-derived binding peptides, and a PWM-based scoring algorithm was used to search the yeast proteome for closely matching sequences, which were subsequently aligned in an unrooted clustergram. The specificity profile for each SH3 domain is represented next to the name. The SH3 domain specificity classes are colored as follows: I (red), II (blue), and III (green). Specificity profiles that could not be assigned to any class are shown in black. Underlined names indicate domains that exhibit two distinct specificity profiles.

To compare the binding specificities for the yeast SH3 domain family on a global scale, we clustered all domains in an unrooted tree based on their specificities (Figure 1 and Figure S1). We generated a set of 10,000 random peptides from the yeast proteome and used these to score each phage-derived PWM. The match of each PWM with each peptide was calculated using an information score yielding a 10,000-dimensional profile vector for each PWM. This profile vector describes the binding specificity in a cellular context by sampling the sequences that the domains would be exposed to in the cell. The similarity between PWMs was computed as the Pearson correlation between these vectors. PWMs were then clustered according to this similarity measure using a complete linkage algorithm. Hence, the tree represents natural yeast SH3 domain specificity as it clusters binding profiles based on endogenous protein ligands. Overall, our results are consistent with previous findings [2]; in addition, this higher resolution analysis reveals that each domain exhibits specificity across multiple ligand positions, including the core motif and flanking positions.

Our specificity map reveals that the majority of yeast SH3 domains have specificities that can be defined as class I or II, with eight and 12 representatives, respectively. Notably, the SH3 domains from Cyk3p and Rvs167p, and a protein fragment containing the two N-terminal domains from Sla1p (Sla1-1/2) exhibit dual specificity for both ligand classes (Figure 1). Furthermore, the specificity map uncovered many specificity profiles that do not cluster with either of the canonical classes. For instance, the SH3 domains of Bem1p, Hof1p, Myo3p, and Myo5p comprise a distinct cluster, which we denote as class III, and are characterized by their preference for poly-proline ligands, without the requirement for flanking charged residues. The SH3 domains of the paralogs Pin3p and Lsb1p exhibit dual specificity, recognizing class II ligands and a ligand set (+XXXPXP) that resembles class I ligands, but with different residue spacings, thus was left unclassified. The SH3 domain of Pex13p also exhibits dual specificity for class II ligands and for a second motif characterized by a positively charged residue located between proline residues, which does not fit any defined class. The specificity profiles for the paralogs Boi1p and Boi2p (PXXXPX+) resemble class II, but with proline spacings that differ from the canonical binding motif, and have also been left unclassified. Finally, as observed previously [2], the SH3 domain of Fus1p exhibits a unique specificity profile that does not include prolines.

**Figure 2:**
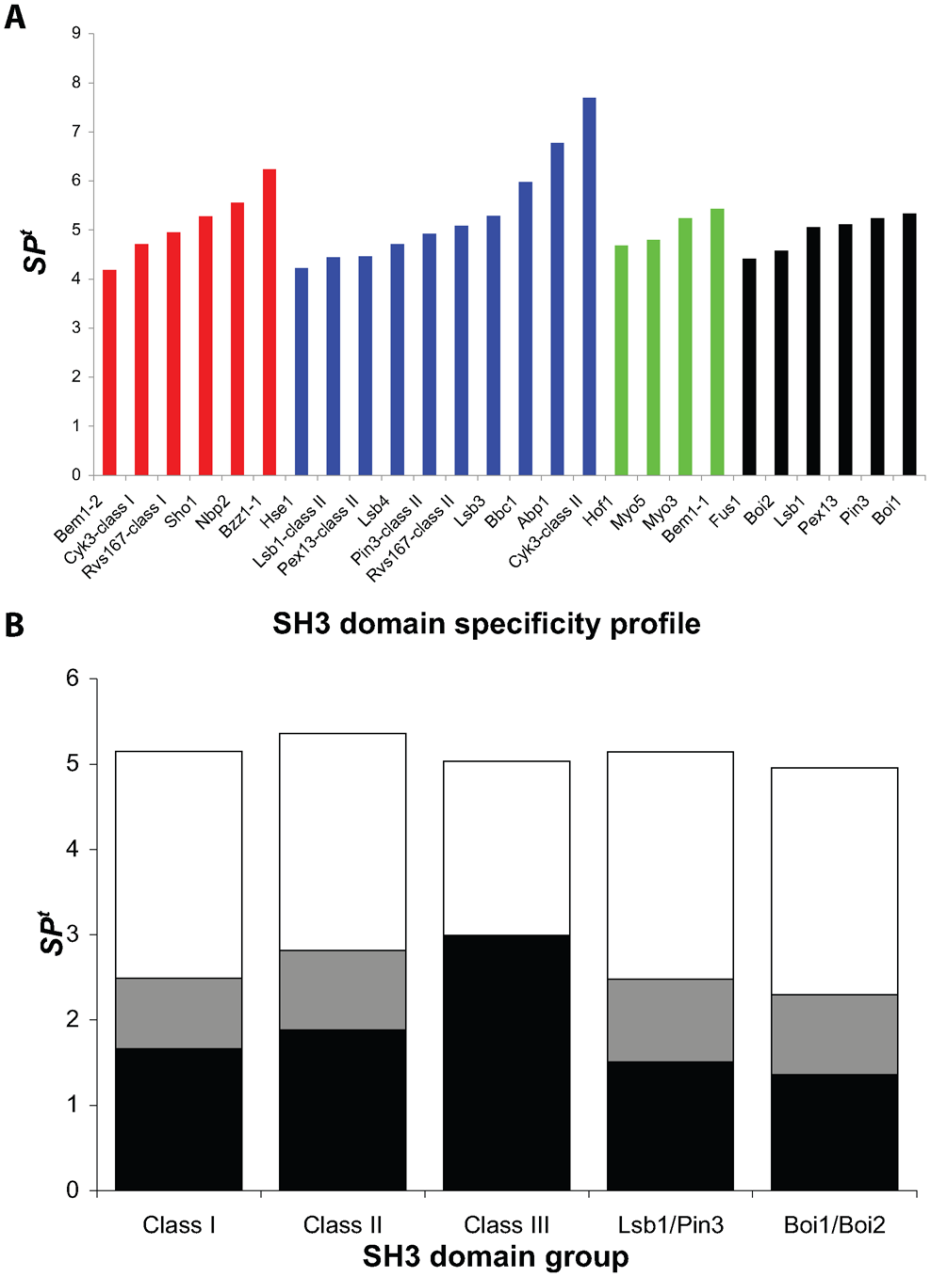
The intrinsic specificities of yeast SH3 domains. (A) *SP^t^* scores for yeast SH3 domain specificity profiles containing ten or more peptides. The values were determined for the PWMs shown in Figure 1, and the bars are colored according to the specificity class, as in Figure 1. (B) Contributions to *SP^t^* from different elements of SH3 domain specificity profiles. Contributions from proline or positively charged residues within the core binding motifs are colored black or grey, respectively, whereas contributions from positions outside of the core binding motifs are colored white. The core binding motifs for the various groups were defined as follows: class I (+XXPXXP), class II (PXXPX+), class III (poly-proline), unclassified Lsb1/Pin3 (+XXXPXP), and unclassified Boi1/Boi2 (PXXXPX+).

To compare the intrinsic specificities of yeast SH3 domains, we quantified the specificities using a specificity potential (*SP*) score, which was applied previously to the PDZ domain family [3]. The *SP* value summarizes the specificity observed in each column of a PWM as a numerical value ranging from zero (least specific) to one (most specific; Table S3). We had sufficient peptide data (*n*>10) to calculate reliable *SP* scores for 26 distinct specificity profiles. By summing the *SP* score across all PWM columns, we calculated a total *SP* (*SP^t^*) score for each SH3 domain specificity profile. Most yeast SH3 domains exhibit similar intrinsic specificities with *SP^t^* values ranging from four to six (Figure 2A). Furthermore, domains that recognize more than one class of ligands do so with approximately the same level of specificity for each class. This analysis reveals that the Cyk3p SH3 domain [16]–[18] has an unusually high *SP^t^* value for class II ligands, which stems from its strong preference for an Asp-Tyr motif downstream of the Arg residue of the canonical class II motif (Figure 1).

To assess the specificity contribution from different elements in the binding profiles, we quantified separately the *SP* scores for the positions within or outside the core motif for the various specificity profiles (Figure 2B). The core positions for classes I and II only contribute roughly half of the *SP^t^* value, with the other half being contributed by other positions that define distinct specificity niches. Analogously, residues outside the core positions contribute approximately the same level of specificity for the unique sets of ligands recognized by Lsb1/Pin3 and Boi1/Boi2 (Figure 2B). For class III domains, we found that recognition of proline accounts for approximately 60% of the *SP^t^*. Taken together, these results highlight the importance of residues outside the core positions for mediating specificity in SH3 domain–ligand interactions.

### Phage-Derived Specificity Profiles Correlate with Ligand Affinities

**Figure 3:**
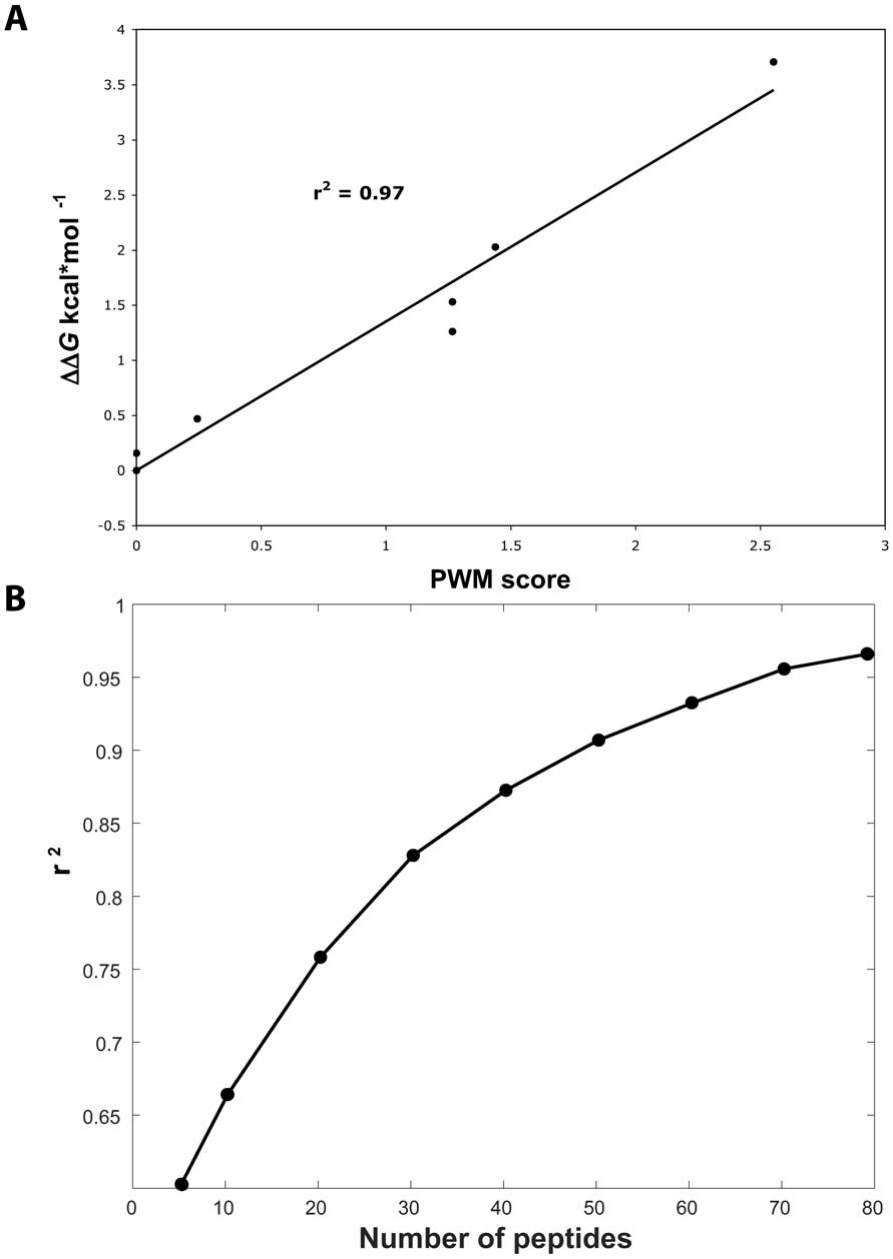
Phage-derived specificity profiles correlate with binding affinities. (A) In vitro affinity data for binding to the SH3 domain of Sho1p were used to calculate the differences in Gibbs free energy (ΔΔ*G*) for various peptides targeted relative to a reference peptide (IRSKPLPPLPV; *y*-axis), and these were plotted against the score match to the phage-derived PWM (*x*-axis). (B) A plot of the correlation (*r* ^2^) observed in (A) (*y*-axis) and the number of peptides used to derive the PWM (*x*-axis).

Phage display generally selects high-affinity ligands through an iterative panning process, and high-resolution PWMs have been used to predict preferences in selectivity that reflect binding affinities for PDZ domain–ligand interactions [3],[19]. To assess the accuracy of our phage-derived data for SH3 domains, we examined the SH3 domain of Sho1p and determined the correlation of PWM scores derived from phage display to differences in Gibbs free energy (ΔΔ*G*) derived from previous in vitro binding assays with synthetic peptides [20] (Table S4). We observed an excellent correlation between the two datasets (*r* ^2^ = 0.97; *p* = 7.8×10^−5^; Figure 3A), and a significant correlation was also observed for similar datasets for the SH3 domain of Abp1p [21] (*r* ^2^ = 0.73; *p* = 2.1×10^−4^; Figure S2 and Table S5). For the SH3 domain of Sho1p, the correlation between binding affinity and PWM score match is proportional to the number of peptides used to generate the PWM, and good correlation is observed for datasets containing >30 peptides (*r* ^2^>0.8). Notably, 22 of our SH3 domain specificity profiles are derived from 30 or more ligands, suggesting that the majority of our phage-derived data can predict accurately the relative in vitro affinities of peptide ligands for SH3 domains.

### Identification of Natural SH3 Domain Ligands by Peptide Array Screening

The use of synthesized peptide arrays offers an alternative and independent approach to query PRM–ligand interactions. In an ideal scenario, unique peptides representing the entire proteome of the organism would be spotted onto an array and assayed individually for interactions with a PRM of interest. However, in practice, a filtering step is required to generate an array of manageable size. In a strategy dubbed WISE (whole interactome scanning experiment), natural ligands for PRMs are identified by computationally scanning the proteome for sequence patterns similar to known ligands, and these are tested for interactions using synthetic peptide array (SPOT) technology. Proteome scanning can use regular expressions (REs), which describe discrete text patterns, or PWMs, which describe probabilistic positional frequency-based patterns. Although both methods rely heavily on the quality of the information they are based upon, REs run a higher risk of missing candidate ligands (higher false-negative rate), whereas PWMs often fail to catch strict position-specific rules. Following identification of putative natural ligands by either filtering method, the peptides are tested for interactions by SPOT.

We used the WISE approach to generate a yeast SH3 domain interaction network independently by creating a set of 15 REs based on SH3 domain specificity profiles identified in this study and previously [2], and searching for matches in the yeast proteome (Text S1). The stringency of the REs was set very low in order to maximize the number of putative ligands tested on the array. Although this approach potentially identifies a number of false positives, the goal is to capture as many interactions as possible, thus minimizing the number of false negatives. This analysis identified 2,953 peptides within 1,693 proteins (almost one-third of all yeast ORFs; Table S6). This defined set of peptides was synthesized on cellulose membranes according to a modified SPOT synthesis approach [22]. Subsequently, peptide arrays were screened for binding individually with 26 SH3 domains. In total, we identified 295 peptides that showed a positive signal with at least one SH3 domain (Table S7).

Peptides identified by either PWMs or REs address the ability of a domain to bind to a ligand outside of its protein and cellular context, but the peptides are identified by independent computational approaches with different strengths and weaknesses. To address this, we used the PWMs to define a set of peptides of similar size to the one defined by the REs. Interestingly, this analysis revealed only an approximately 30% overlap between the peptide sets defined by REs and PWMs. To examine the PWM-defined peptides experimentally, we tested in the SPOT assay the ten peptides with the highest PWM score for each SH3 domain. Of the 230 PWM high-scoring peptides, 113 were not included in the original WISE interactome, and approximately 55 of these gave a significant SPOT signal with at least one SH3 domain (Table S8). The 55 peptides predicted by PWM but missed by RE that yielded a significant SPOT signal can be regarded as false-negative interactions for the RE approach; therefore, the false-negative hit rate for the RE-defined peptides appears to be approximately 20%. Notably, of the 230 PWM high-scoring peptides, 69 did not generate a SPOT signal, which suggests that the PWM false-positive rate is on the order of approximately 30%.

The SPOT approach is semiquantitative, so we also examined the correlation between interaction signals and dissociation constants, but we found that, as reported previously [22], it was much poorer than that observed with the phage-display score (unpublished data). Thus, although SPOT assays can be used to validate PWM-predicted interactions, further development of the method is required to obtain highly accurate quantitative signals. Taken together, our SPOT analysis of the yeast SH3 interactome yields a weighted graph of more than 5,000 edges, which served as an additional source of semiquantitative information to be integrated into a map of yeast SH3 domain interactions.

### Yeast Two-Hybrid Screens

To complement the phage display and SPOT experiments, we performed large-scale yeast two-hybrid screens. We screened 22 yeast SH3 domain baits against a novel yeast activation domain ORFeome library [23], which tests for interactions with full-length proteins, using an array-based approach as described previously [24] and repeating each screen twice (Table S9). In addition, 26 SH3 domain baits were screened against a randomly fragmented genomic library (gDNA), which tests for interactions with protein fragments [25] (Table S9). In total, we identified 801 unique interactions, consisting of 241 and 587 interactions from the ORFeome or gDNA library screens, respectively (Table S10). Only 26 interactions were identified in both screens (10.7% or 4.4% of the interactions identified by the ORFeome or gDNA screens, respectively). Using the ORFeome screen, we identified an average of 11.0 interactions per SH3 domain, whereas we detected an average of 22.6 interactions per SH3 domain in the gDNA screen. One major reason for the difference in these numbers is that we sequenced approximately 200 positive single colonies from each gDNA library screen in an attempt to saturate the system. Furthermore, although we repeated the ORFeome screening twice, this is not expected to achieve complete saturation according to a recent analysis [23]. In total, we sequenced 3,965 yeast two-hybrid–positive colonies, and some interactions were captured multiple times (593 interactions were captured at least twice) by each screening technique (Table S10).

To assess the potential of identifying biologically relevant interactions, we examined the number of literature-validated interactions that were identified by each approach. To do so, we curated a comprehensive “gold-standard” set of 42 SH3 domain interactions from the literature (see below). Within this gold-standard set, only five were identified by the ORFeome screen, whereas 28 were identified by the gDNA screen. Thus, with yeast SH3 domains, gDNA two-hybrid screening has a 2.5-fold lower false-negative rate than ORFeome analysis (Figure S3), which may reflect both that our screening of the gDNA library was more extensive and that it contains gene fragments corresponding to protein domains, which often behave better in the two-hybrid system [26]. Taken together, these results highlight the complementary nature of ORFeome and gDNA screening methods to experimentally identify protein interactions for PRMs.

**Figure 4:**
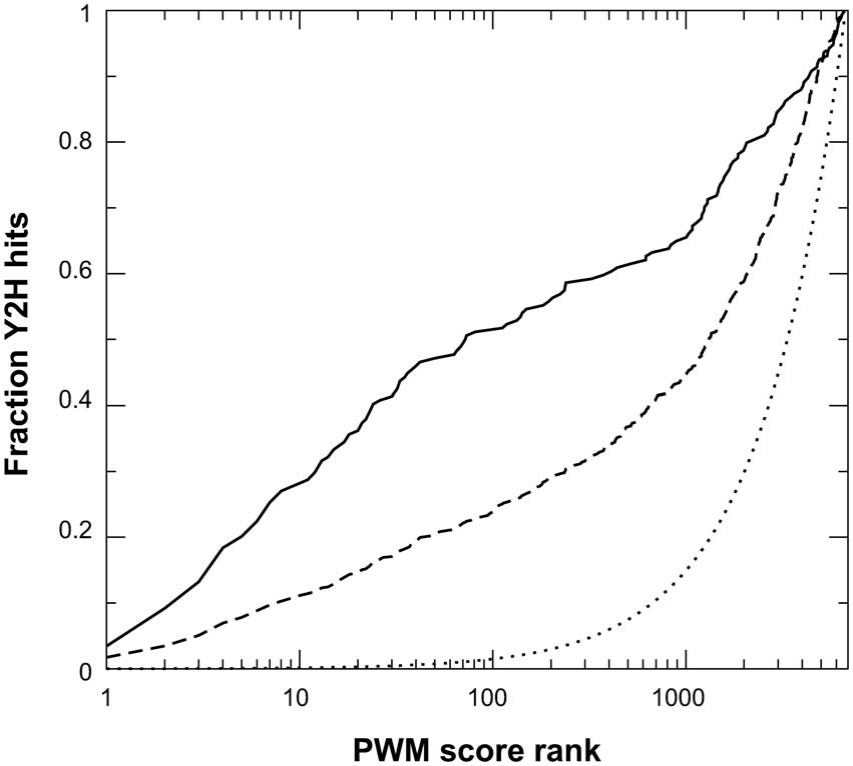
Yeast two-hybrid hits contain high-scoring PWM matches. The phage-derived PWM for each SH3 domain was used to search the yeast proteome for matching peptides based on a PWM scoring algorithm, which ranks each peptide based on how closely it resembles the PWM. The fraction of yeast two-hybrid (Y2H) hits containing sequences with a rank score higher than the value on the *x*-axis is shown for: yeast two-hybrid interactions observed at least six times (solid line), yeast two-hybrid interactions observed at least one time (dashed line), and randomly chosen interactions (dotted line).

As yeast two-hybrid and phage display potentially query different regions in interaction space, we sought to determine the overlap between the two methods. The phage-derived PWMs were used to search the yeast proteome for matching peptide ligands based on a PWM-scoring algorithm. For each SH3 domain, the yeast proteins were ranked according to their associated PWM score. Subsequently, the fraction of yeast two-hybrid hits containing predicted ligands with a rank higher than a defined threshold (*x* = 1, 2,…*N*, where *N* is the size of the yeast proteome) was determined. We find that approximately 10% of two-hybrid positives rank among the top ten hits predicted by the PWM of the associated SH3 domain (Figure 4, dashed line). The fraction of yeast two-hybrid hits with peptide sequences ranked among the top ten PWM-predicted ligands is increased to more than 25% when considering interactions that are captured at least six times, suggesting that these interactions have a higher likelihood of representing bona fide SH3 domain ligands (Figure 4, solid line). The high fraction of yeast two-hybrid positives with high-scoring PWM matches, compared to those predicted for random interactions, suggests that the detailed binding specificity uncovered by phage-derived PWMs was recapitulated using the yeast two-hybrid system.

### Generation of a High-Confidence SH3 Domain Protein–Protein Interaction Network Using Bayesian Integration

Each experimental method has different strengths and biases, and the integration of data from independent techniques increases the accuracy of the resulting dataset substantially [27]. We generated a yeast SH3 domain protein–protein interaction network and used a statistical approach based on Bayesian networks [27] to assign each interaction a probability score. This score is based on the confidence level of the experimental data that defined the interaction benchmarked by the gold-standard set (see Materials and Methods and Table S11). A Bayesian networks formalism was chosen for the machine learning because it has been shown previously to perform well at integrating heterogeneous biological data [27],[28].

The gold-standard set represents a list of manually curated interactions known to be mediated by a specific SH3 domain, compiled through an exhaustive literature search. Each interaction in the gold-standard set is supported by multiple experiments reported in one or more focused studies, which show the direct binding of the SH3 domain to its target, and its functional relevance.

Each technique utilized in our analysis encompasses a quantitative measure: first, the phage-derived PWMs accurately represent relative binding affinities; second, interactions identified by SPOT peptide arrays can be binned and ranked based on intensity (see Materials and Methods); and third, interactions captured multiple times by yeast two-hybrid can be assigned a higher score than those captured only once. Furthermore, the different methods have complementary features. Whereas the phage display and SPOT peptide array signals correlate with and predict binding affinity, the yeast two-hybrid system identifies putative in vivo interactors of SH3 domains. We therefore integrated these datasets into a Bayesian model to identify highly likely SH3 domain–ligand interactions.

**Figure 5:**
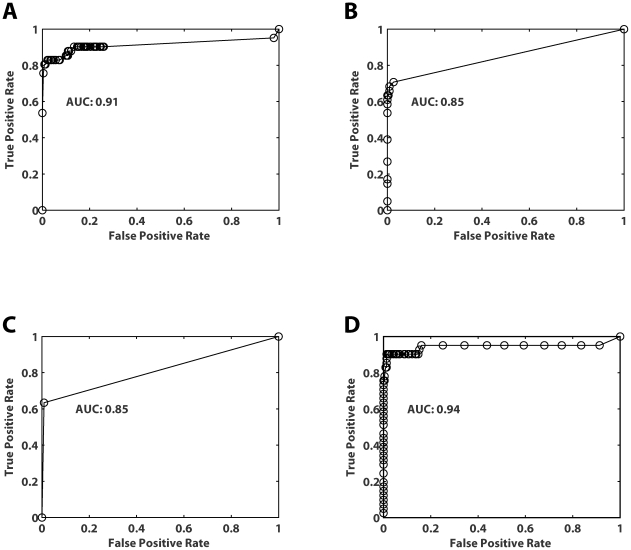
Performance analysis of independent screening methods used to identify SH3-mediated interactions. (A–C) ROCs were plotted against a gold-standard set of interactions (Table S11) for all three techniques used in this analysis, and the area under the curve (AUC) was calculated: (A) phage display; (B) SPOT arrays; (C) yeast two-hybrid. (D) Bayesian model integrating the results from the three independent techniques.

All interactions in the gold-standard set were mapped specifically to an SH3 domain and, where applicable, to the peptide sequence within the interacting partner (see Materials and Methods and Table S12). We generated a negative set using random protein pairs under the constraint of never sharing or being in “adjacent” cellular compartments (see Materials and Methods). To determine the sensitivity of each technique individually, we plotted their respective receiver-operating characteristic (ROC) curve, a standard assessment of accuracy, and examined the area under the curve (AUC; Figure 5). The phage-derived PWMs were found to exhibit the highest AUC (0.91; Figure 5A and Figure S4), with the SPOT peptide array and yeast two-hybrid exhibiting a lesser value (0.85 in both cases; Figure 5B and 5C, respectively). Remarkably, the Bayesian network, which integrates the data from all three techniques, results in an AUC of 0.94 (Figure 5D; *p* = 1.2×10^−10^; Figure S5), suggesting that our probabilistic interaction network captures the vast majority of literature-validated interactions. The entire set of yeast SH3 domain–ligand interactions predicted by our Bayesian model is represented as a network diagram and summarized in table format (Figure S6 and Table S12).

### Specificity in the SH3 Domain Network

To assess how profile specificity translates into specificity at the level of the network, we computed for each SH3 domain, the fraction of its interactors in the Bayesian network that are targeted by at least one other domain (Figure S7). Our results show that many proteins (61%) are targeted by only one SH3 domain. The other proteins (39%) are predicted to bind to more than one SH3 domain. Furthermore, important differences between SH3 domains can be observed, some of them having very unique specificity (e.g., Fus1p SH3 domain), whereas others share most of their interactors with other domains. The latter is especially true for SH3 domains from paralogous proteins such as Boi1p/Boi2p, Lsb1p/Pin3p, Lsb3p/Lsb4p, and Myo3p/Myo5p.

To study specificity further, we also distinguished the different predicted binding sites on each protein (binding sites are predicted by the best PWM hit in the protein sequence), since a protein can be targeted by multiple SH3 domains but at different binding sites. Interestingly, the fraction of binding sites targeted by more than one SH3 domain is lower than the fraction of proteins targeted by more than one SH3 domain (29% against 39%), revealing that some proteins have multiple unique binding sites recognized by individual SH3 domains (Figure S7, grey bars). However, cases of possible competition are not completely removed by distinguishing the different binding sites.

To assess the contribution of SH3 domains from the same protein and highly similar SH3 domains, we merged Bzz1-1 and Bzz1-2, Sla1-1/2 and Sla1-3, and the four pairs of close paralogs (Boi1p/Boi2p, Lsb1p/Pin3p, Lsb3p/Lsb4p, and Myo3p/Myo5p), treating each of them as a single protein since they have highly similar specificity profiles. In this case, we found that 33% of all interactors are targeted by more than one SH3-containing protein in our network (Figure S8). As previously, we distinguished the different binding sites in each protein target and found that 23% of binding sites are targeted by more than one SH3-containing protein (Figure S8). Thus, the majority of interactions are expected to be insulated from competition effects, due to sequence differences among binding sites alone, though some competition among domains is likely.

### Biological Process Enrichment in the Network

As one approach to assessing the biological relevance of interactions identified by the Bayesian model, we examined biological process annotation associated with the putative SH3 domain ligands, defined by Gene Ontology (GO). We found a significant number of overrepresented biological processes known to be associated with yeast SH3 domain biology such as establishment of cell polarity and endocytosis (*p* = 3×10^−7^ and *p* = 9×10^−8^, respectively). Moreover, from a recently published set of approximately 60 known and putative endocytosis proteins, 29 were found to be connected with at least one SH3 domain in our interaction network [29] (Figure S6). In addition, by searching for highly interconnected nodes in the Bayesian interaction network, we identified a core of 31 proteins that engage in at least six interactions with each other (k-core = 6; Figure S9). Consistent with the GO term enrichment analysis described above, 14 of the proteins that emerge from the k-core analysis (e.g., Las17p, Myo3p, and Vrp1p) have well-defined roles in endocytosis with a GO enrichment *p*-value of 5×10^−8^ [29],[30]. Hence, we decided to focus on the SH3-mediated interactions underlying endocytosis in more detail.

### SH3 Domain Interactions in Endocytosis

Endocytosis is a complex cellular process in which a dynamic array of protein interactions are sequentially coordinated to drive endocytic site initiation, membrane invagination, and vesicle scission [31]. Live-cell imaging analyses uncovered a detailed spatiotemporal map for the dynamic recruitment of numerous proteins to endocytic sites in budding yeast [31],[32]. These studies proposed the existence of four dynamic protein modules that cooperate to drive vesicle formation: (1) the endocytic coat module, (2) the Wiskott-Aldrich syndrome protein (WASP)-myosin (WASP/Myo) module, (3) the scission (or amphiphysin) module, and (4) the actin module.

Proteins in the endocytic modules arrive sequentially at sites of endocytosis with precisely defined dynamics and their assembly drives the steps of endocytic internalization. The first step in the endocytic pathway is the recruitment to the plasma membrane of the coat module proteins, which include clathrin, Sla1p, Pan1p, End3p, and Sla2p. The assembly of the coat module occurs prior to and independent of actin assembly. However, the subsequent movement of the coat proteins into the cell, and subsequent coat disassembly, are dependent upon actin polymerization. One to two minutes following coat module assembly, Las17p (the yeast ortholog of WASP) is recruited, which activates the Arp2/3 complex to promote actin assembly. The SH3 protein Sla1p is thought to inhibit the actin polymerizing function of Las17p. This inhibition appears to be relieved by the recruitment of members of the WASP/Myo complex, including Vrp1p and the SH3 proteins Bbc1p, Myo3p/Myo5p, and Bzz1p. Actin polymerization triggered by the WASP-myosin complex leads to recruitment of the actin module proteins, which include actin, Cap1p, Cap2p, Sac6p, Abp1p (SH3 protein), and the Arp2/3 complex, leading to further actin polymerization. As the vesicle begins its movement into the cell, the scission module, consisting of Rvs161p and the SH3 protein Rvs167p, is recruited. Although the exact scission mechanism is unclear, the scission module promotes the release of the nascent endocytic vesicle [29],[31]. In contrast to components of the coat module, proteins of the WASP/Myo module remain immotile at the plasma membrane as actin is being polymerized, and disassemble as the nascent vesicle is internalized [29],[31].

**Figure 6:**
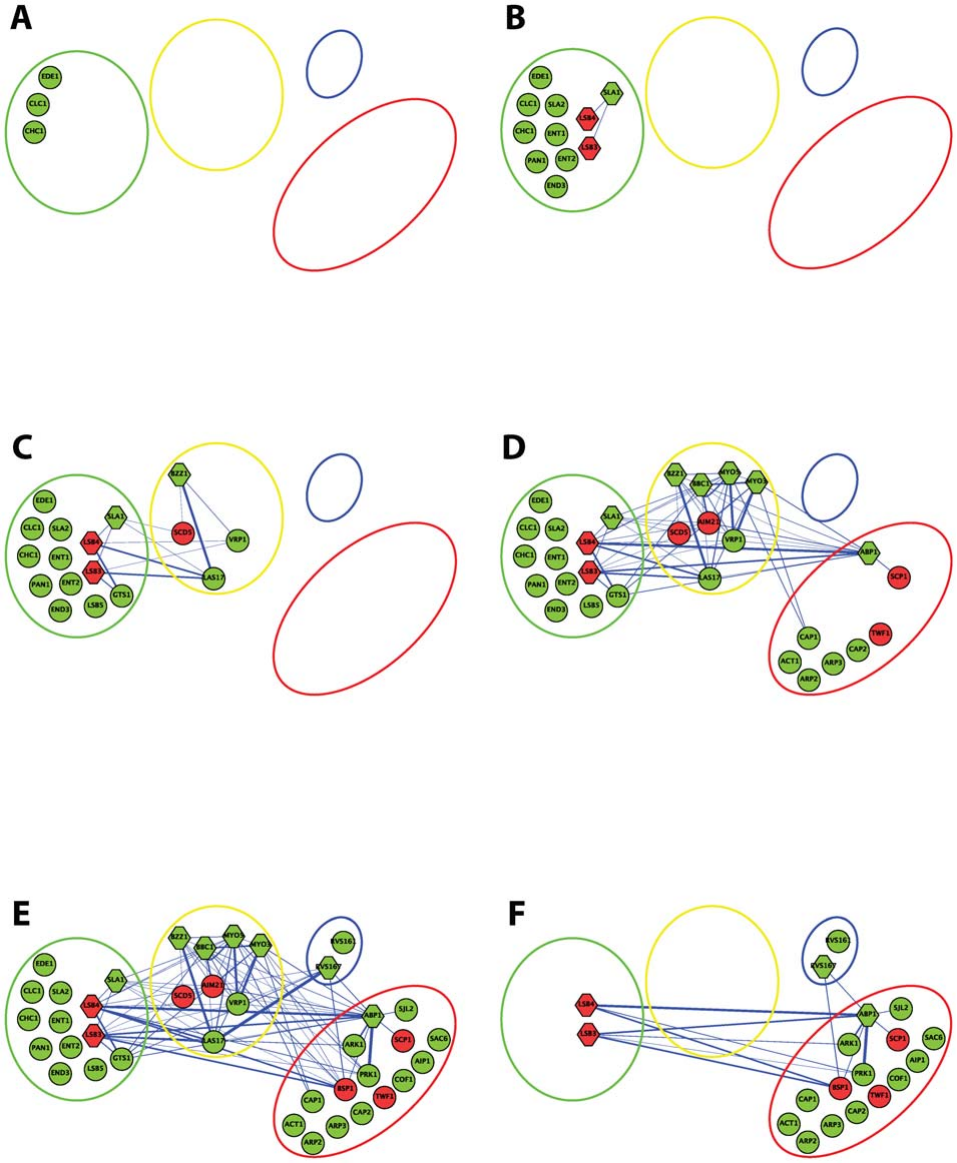
Yeast SH3 domain interaction network underlying endocytosis. SH3 domain–mediated interactions predicted by the Bayesian model are shown for endocytosis proteins. Interacting proteins were divided amongst their respective modules (coat, WASP/Myo, scission, and actin, represented as green, yellow, blue, and red circles, respectively) and are highlighted in green when known to be colocalized within a given module [29] at each time frame (in seconds) in endocytosis prior to vesicle internalization: (A) −120 to −40, (B) −40 to −35, (C) −35 to −25, (D) −25 to −15, (E) −15 to −10, and (F) −5 to 0. Disassembly of the endocytic vesicle and its concomitant internalization into the cytosol of the cell is taken as time 0 s, and therefore, all time points prior to this step are shown with negative time scales. Previously uncharacterized endocytosis proteins analyzed in this study are highlighted in red and assigned to their predicted endocytic module based on their SH3 domain interaction score (Table S13). The thickness of the edge is proportional to the Bayesian probability score for each interaction.

Spatiotemporal characterization of protein dynamics by live-cell imaging has provided a detailed view of endocytosis, but our understanding of this pathway is far from complete. It has been established that numerous proteins arrive at sites of endocytosis in a choreographed manner, but it is not known how the sequential recruitment, assembly, and functions of endocytic proteins are achieved. Our Bayesian interaction network contains 29 of the 60 or so known yeast endocytosis proteins, including ten that contain SH3 domains. To gain insights into the roles of SH3-mediated interactions in endocytosis, we screened for putative ligands for these ten SH3 domains using our Bayesian scoring algorithm (Table S13). The interacting proteins were grouped with the respective protein modules described above, and the putative SH3-mediated interactions at each stage of endocytosis were determined (Figure 6 and Table S13). This analysis uncovered a vast array of putative SH3 domain–mediated interactions, with 53 connections among 19 known or putative endocytic proteins, and suggested that interactions are likely to become more prevalent as additional proteins are recruited to the endocytic site (Figure 6). Furthermore, the interaction network suggests that the majority of SH3 domain–mediated interactions are established 35 to 15 s prior to vesicle internalization (Figure 6C to 6E). This timing suggests that SH3 domains play a particularly important role at the stages encompassing assembly of the WASP/Myo module, actin polymerization, membrane invagination, and vesicle scission.

The network allows us to map potential interactions onto the temporal order of protein recruitment at the site of endocytosis, and these interactions likely mediate assembly of protein modules and coordinate activities between the modules (Figure 6). We therefore examined in greater detail the relationships between SH3 domain–mediated interactions and protein dynamics during endocytosis. For each protein, we summed Bayesian probability scores (or interaction scores) based on interactions with proteins from within its corresponding module compared to interactions with proteins from external modules (Table S13). This analysis revealed that proteins have the highest total interaction score for interactions occurring within the same module. This was the case for 11 of 13 endocytic proteins for which a suitable Bayesian probability score and dynamic data were available (Table S13). For instance, the network identified a large number of interactions between members of the WASP/Myo module (Bbc1p, Bzz1p, Las17p, Vrp1p, Myo3p, and Myo5p), which arrive following the coat module, 35 to 25 s prior to vesicle internalization (Figure 6C and 6D). Summing their Bayesian probability scores across all modules revealed that each of these proteins has the highest combined interaction score for interactions within the WASP/Myo module. This finding provides support for the conclusion that the SH3 domain–mediated interactions are required for the assembly of this module, and that interactions between these proteins are established upon their temporal recruitment to the endocytic site.

Subsequent to the formation of an SH3 domain–mediated network within the WASP/Myo module, the network analysis points to the formation of an SH3 domain–mediated network within the actin module (e.g., Abp1p, Ark1p, Prk1p, and Sjl2p [33]), at 25 to 10 s prior to vesicle internalization (Figure 6D and 6E). Interestingly, proteins from the actin module also appear likely to engage in many interactions with members of both the WASP/Myo and actin modules, suggesting extensive cross-talk between these two modules (Figure 6). However, the interaction scores for proteins within the same module were higher than those for proteins in different modules, underscoring the predictive potential of using interaction scores to place endocytic components into their respective modules (Table S13).

Our network analysis, which incorporates both SH3 domain–mediated interactions and dynamics of endocytic proteins, suggests that members from the same endocytic module engage in tighter SH3 domain–mediated interactions and have similar spatiotemporal dynamics. This raises the possibility of predicting the dynamics of putative endocytic proteins based on their SH3 domain interaction profile. Thus, an uncharacterized endocytic protein is predicted to be part of the module within which it registered the highest interaction scores. For example, if an uncharacterized protein is implicated as a member of the WASP/Myo module because it has high scores with SH3 domains within the WASP/Myo module, then we predict that its dynamics will follow a similar pattern to those of other proteins in that module. Analogously, an uncharacterized SH3 domain protein would be predicted to be part of the module containing its best-predicted binding partners.

To test our hypothesis, we quantitatively examined the protein dynamics of five uncharacterized endocytosis proteins (Scd5p, Aim21p, Scp1p, Bsp1p, and Lsb3p), each of which had a high SH3 interaction score with at least one of the established endocytic modules (Table S13). We predicted that Scd5p, a protein first identified as a suppressor of defects in cells depleted of clathrin heavy chain (Chc1p) [34], arrives with and is part of the late coat module (with Sla1p, and Sla2p, etc., but not with the early coat protein, clathrin) and/or WASP/Myo module. We also predicted that Aim21p, a fungal-specific protein, is a component of the WASP/Myo module, and that Scp1p, a conserved member of the Calponin/transgelin family of actin-associated proteins [35], is part of the actin module.

Two closely related SH3 domain proteins, Lsb3p and its paralog Lsb4p, had high interaction scores across several modules, most significantly with early (e.g., coat and WASP/Myo) and late (actin) modules (Figure 6 and Table S13). Notably, we observed that the score for a particular module did not exceed the median interaction score across all other modules by more than 2-fold. This unique pattern of interactions suggests that Lsb3p and Lsb4p may play a role to cluster and to coordinate the activities of several module components at the site of endocytosis. In addition, Bsp1p, an adapter that links the yeast synaptojanins, Inp52p and Inp53p, to the cortical actin cytoskeleton and participates in actin contractile ring function [36], showed a similar interaction profile, and therefore, we speculated that it too might be a cross-module protein together with Lsb3p and Lsb4p.

**Figure 7:**
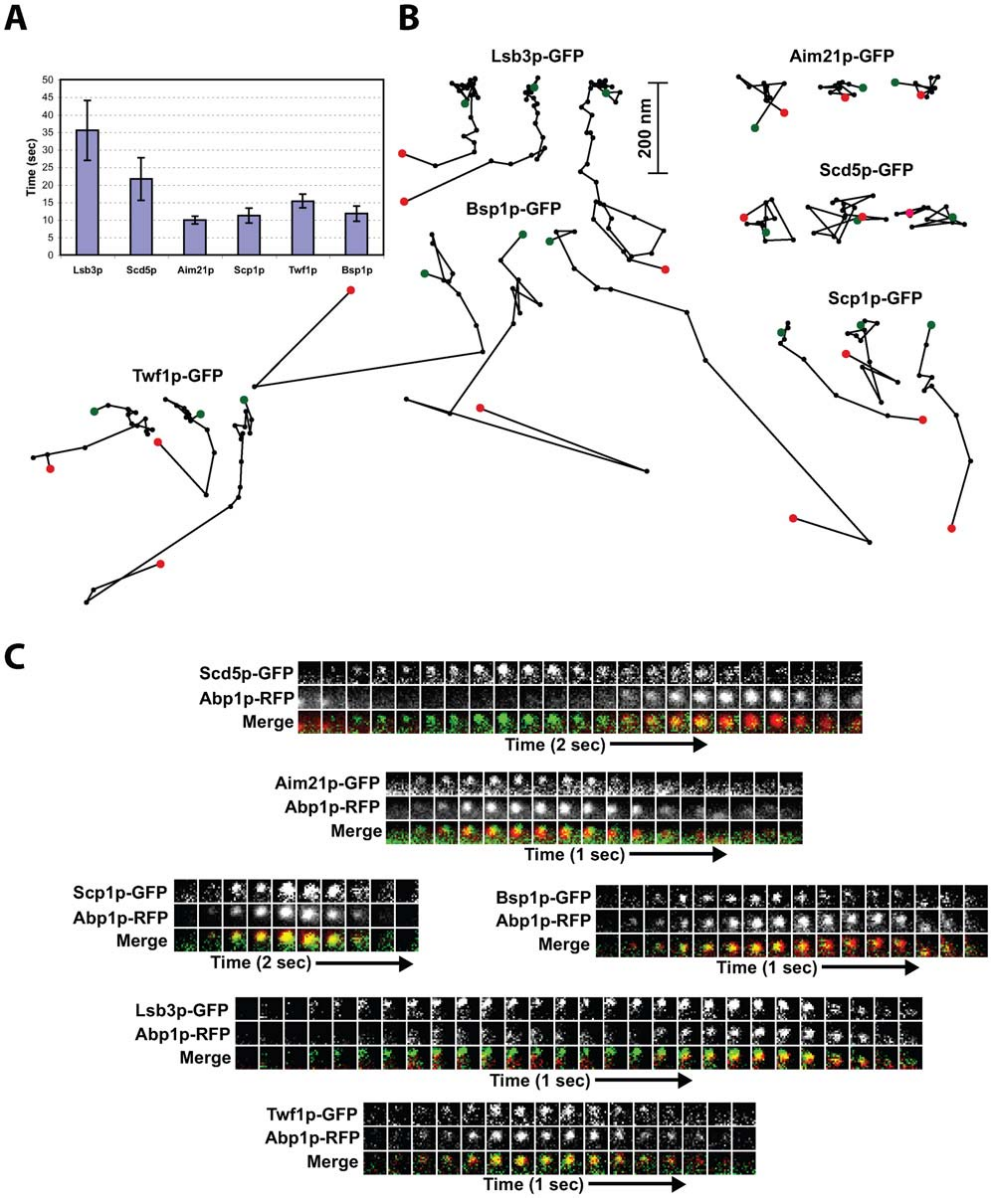
Protein dynamics at sites of endocytosis. (A) Lifetimes for different GFP-tagged endocytic proteins±standard deviation (*n* = 35 patches). All movies were taken with 1-s frame intervals. (B) Particle tracking for endocytic proteins. Positions of the centers of patches were determined for each frame of a movie (1 frame/s) from a medial focal plane of a cell, and consecutive positions were connected by lines. Green and red dots indicate initial and final positions, respectively, for each patch. All traces are oriented so that the cell surface is up and the cell interior is down. (C) Dynamic localization of GFP-tagged proteins compared to Abp1p-RFP in living cells. Time series showing composition of individual patches from two-color movies. Upper and middle rows show two separate channels, and lower panel shows merged images.

To test our predictions in vivo, each protein was C-terminally tagged with GFP and expressed from its endogenous locus in yeast cells. The dynamics of each protein were analyzed individually and in tandem with Abp1p-RFP. The dynamic localization analysis validated our approach for assigning proteins to endocytic modules based on their interaction scores (Figure 7 and Table S13).

In agreement with earlier observations [37], we found that Scd5p-GFP patches had a lifetime of 22±6 s (Figure 7A). Simultaneous, two-color analysis with Abp1p-RFP, a marker for actin polymerization, revealed that Scd5p-GFP arrives prior to actin polymerization (Figure 7B). However, Scd5p-GFP patches were immotile throughout their lifetime, like proteins of the WASP/Myo module (Figure 7C). These dynamics establish Scd5p as a component of the WASP/Myo module with similar dynamics to Bzz1p, suggesting that it might participate in late coat formation and/or coordinate this module with the WASP/Myo module. Moreover, Scd5p was recently reported to have a role in phospho-regulation of the endocytic coat complexes and its spatial dynamics may have a role in this essential function [37].

For Aim21p-GFP, we observed that it is located in immotile patches with a lifetime of 10±1 s, similar to the patch dynamics reported for Bbc1p [29] (Figure 7A and 7B). Furthermore, Aim21p-GFP arrives when actin begins to polymerize, as revealed by two-color analysis with Abp1p-RFP (Figure 7C). Thus, as predicted, Aim21p localizes as a component of the WASP/Myo module (Figure 6D and Table S13).

Scp1p is expected to be part of the actin module as it is predicted to bind the SH3 domain of Abp1p. Indeed, Scp1p-GFP formed patches with a lifetime of 15±2 s (Figure 7A) and colocalized with Abp1p (Figure 7B and 7C) [30]. These patch dynamics are indicative of proteins in the actin module. Scp1p patches had shorter lifetimes than Abp1p. However, Scp1p-GFP exhibited weak fluorescence intensity, which likely accounted for this lifetime decrease. Two-color analysis revealed strong colocalization between Scp1p and Abp1p with the fluorescence intensity of the patches peaking together (Figure 7C; unpublished data).

As mentioned above, Lsb3p and Lsb4p scored highly across all modules, predicting a long lifetime at the patch. As expected, Lsb3p-GFP patches were long-lived with a lifetime of 36±9 s (Figure 7A). Lsb3p-GFP patches arrived at the cell cortex as an immotile patch, but showed an initial slow movement into the cell to a depth of about 200 nm. The initial slow movement was then followed by a fast, more randomly directed movement further into the cell (Figure 7B). Two-color simultaneous imaging with Abp1p-RFP revealed that, like Sla1p, Lsb3p-GFP arrived early at endocytic sites but persisted late with the actin module proteins (Figure 7C) [30]. These dynamics are consistent with the prediction that the Lsb3p and Lsb4p SH3 domains interact with Sla1p and several actin module proteins (Figure 6). Thus, Lsb3p and Lsb4p appear to provide continuity in the context of a continuously evolving endocytic protein composition.

Finally, we analyzed the dynamics of Bsp1p, which our model suggested interacts with proteins in all modules, similar to Lsb3p and Lsb4p. However, in contrast to the Lsb proteins, Bsp1p-GFP patches were short-lived with a lifetime of 13±2 s (Figure 7A). Bsp1p-GFP colocalized well with Abp1p-RFP and displayed an Abp1p-like motility behavior (Figure 7B and 7C) [30], suggesting that Bsp1p functions within the actin module. Two-color analysis revealed that Bsp1p consistently arrived approximately 1 to 2 s after Abp1p-RFP, in a manner similar to descriptions for Cof1p, Ark1p, or Prk1p [38],[39]. Moreover, unlike other patch proteins, Bsp1p-GFP had an additional stable localization to the bud neck as described previously [40]. Bsp1p is not well characterized, and further studies are necessary to understand the nature of the discrepancy between its predicted interactions with proteins of multiple modules and its appearance only late in the pathway during the burst of actin assembly.

Our SH3 domain network contains only approximately half of the 60 proteins implicated in endocytosis and, as underscored by the Bsp1p example, a number of SH3-independent interactions must control endocytosis protein localization. To emphasis this point, we also analyzed the dynamics of yeast twinfilin (Twf1p), a highly conserved actin monomer-sequestering protein involved in regulation of the cortical actin cytoskeleton [41], which was not predicted to bind to any SH3 domain. Similar to Scp1p and Bsp1p, Twf1p localized to the patch with a lifetime of 15±2 s (Figure 7A). The patches were initially immotile at the cell surface but subsequently underwent a highly motile phase, after which the patch moved long range into the center of the cell (Figure 7B), in a manner characteristic of proteins comprising the actin module.

**Figure 8:**
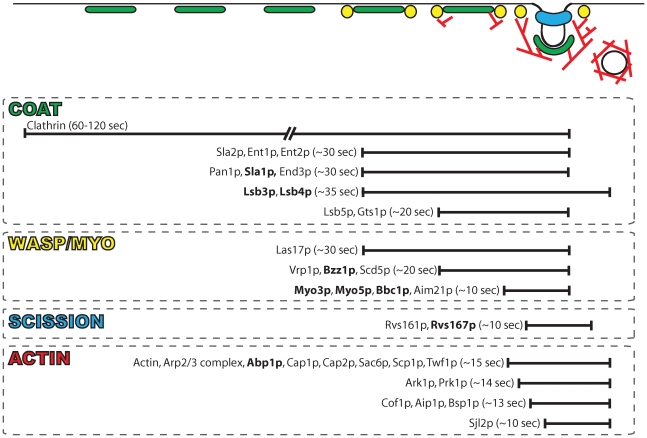
Spatiotemporal map of yeast endocytosis. The spatiotemporal localization of the constituent proteins for each protein module involved in endocytosis is represented as their lifetime (in seconds with representative bars drawn to scale) at the site of endocytic internalization. SH3 domain–containing proteins are shown in bold. The diagram at the top, in which the line represents the plasma membrane, graphically illustrates the dynamic recruitment of the four endocytic modules colored as follows: coat (green), WASP/Myo (yellow), scission (blue), and actin (red). The circle at the far right represents the nascent endocytic vesicle, which is released from the plasma membrane and internalized into the cell by an actin-based mechanism.

In summary, SH3 domain interactions are powerful predictors of spatiotemporal localization of yeast SH3 domain proteins. The putative SH3 domain–mediated interaction networks allowed us to accurately predict the dynamics of several previously uncharacterized proteins in the endocytic pathway and provided a detailed spatiotemporal map of the endocytic pathway (Figure 8).

## Discussion

We generated a specificity map for the SH3 domain family of budding yeast based on 1,871 unique peptide ligands isolated against 25 of the 27 domains. This map reveals that SH3 domains have a high level of intrinsic specificity and different domains recognize distinct sets of ligands. Notably, specificity was observed for ligand positions outside of the core positions, suggesting that SH3 domains utilize multiple features of their peptide ligands to achieve binding selectivity.

A major challenge in functional proteomics is the development of accurate protein interaction networks. We have integrated the data from three independent screening techniques (phage display, peptide arrays, and yeast two-hybrid) into a Bayesian model to generate a yeast SH3 domain interaction map. Each technique has a semiquantitative measure that was captured by the probabilistic model. Our interaction map captures a significant proportion of literature-validated interactions and therefore serves as an accurate reference for additional in-depth studies of yeast SH3 domain biology. Proper interpretation and use of our interaction map requires consideration of additional factors such as cellular concentration, localization, and competition from other SH3 domains to identify physiologically relevant interactions.

Applying our model to proteins involved in endocytosis revealed that there is a significant connection between the time at which a protein arrives at the endocytic patch and its predicted SH3 domain interactions. This correlation was used to accurately predict the spatiodynamics of several uncharacterized endocytic proteins. We found that Scd5p and Aim21p are both components of the WASP/Myo module, which drives vesicle internalization by nucleating actin filament assembly and generating myosin motor-based forces on the actin filaments [29]. Future studies will reveal how these proteins contribute to the function of the WASP/Myo module, but the presence of Scd5p in the WASP/Myo module may be important for its role in phospho-regulation of the endocytic machinery [37]. We also found that Scp1p, Bsp1p, and Twf1p are components of the actin module. Both Scp1p and Twf1p are known actin-binding proteins and may play a role in modulating actin dynamics at endocytic sites [35],[41]. The novel dynamics observed for Lsb3p indicate that it is present across all modules. Based on conserved SH3 predictions and sequence homology, we propose that Lsb4p has similar dynamics. Their numerous predicted interactions, and their dynamics and association with multiple endocytic modules, suggest that Lsb3p and Lsb4p may play an important role in coordinating the activities of the various endocytic modules.

The SH3 interaction predictions did not agree with the dynamics of Sla1p and Bsp1p. Sla1p appears in the coat module, whereas its predicted interactions are more consistent with it being a component of the WASP/Myo module. This may be explained by its established essential role in regulating the WASP/Myo module [29]. Perhaps Sla1p integrates its cargo adaptor role [42] with its roles in actin assembly to prevent premature actin nucleation, and perhaps its departure from the cell surface with the coat proteins separates it from the WASP/Myo proteins, further relieving its inhibition of actin polymerization. Unlike Sla1p, Bsp1p is less well studied and lacks any obvious homology with other proteins. Bsp1p has been linked to the actin module protein Sjl2p, which regulates phosphatidylinositol 4,5-bisphosphate levels [36]. Furthermore, Bsp1p plays a role in actomyosin ring function [40], but it is unclear how this relates to its role at endocytic sites [40]. The delayed recruitment of Bsp1p to the actin module also suggests a role in endocytic site disassembly alongside Sjl2p, Ark1p, Prk1p, and Cof1p [38],[39].

Given the conserved nature of endocytosis from yeast to human [31], it will be of great interest to examine SH3 domain interaction networks in more complex organisms. We emphasize the feasibility of the approach presented here, given the recent discovery that orthologous protein modules generally have very similar specificity profiles [3]. Recent studies of PDZ domains have shown that PRMs are more specific than previously appreciated [3],[43], and we show that the same holds true for SH3 domains. The intrinsic specificity observed at the level of the protein domain itself suggests that there is significant selective pressure driving the domain into a specificity niche not utilized by other domains. As postulated previously [20], an interplay between positive specificity selection acting on the protein interaction module and negative selection acting upon its cognate ligands would ensure high specificity without the requirement of a high-affinity interaction. It appears that such specific interactions have evolved and are necessary to create the dynamic and intricate signaling pathways required for cellular functions.

## Materials and Methods

### Cloning and Protein Expression

For cloning, the SH3 domain boundaries were defined as the union of the domain regions identified by BLAST [12], PFAM [13], and SMART [14], plus an additional ten amino acids (where applicable) on either side as described previously [3]. DNA fragments encoding the identified domains were amplified from *S. cerevisiae* genomic DNA by the polymerase chain reaction (PCR) and cloned into a vector designed for the expression and purification of SH3 domains fused to the C-terminus of glutathione *S*-transferase, as described [44]. All plasmid constructs were verified by DNA sequencing.

### Selection of Peptide Ligands

Phage-displayed peptide libraries (>10^10^ unique members) fused to the N-terminus of the gene-8 major coat protein of M13 filamentous phage were used to select peptide ligands for the collection of purified GST-SH3 fusion proteins. All domains were first screened using a random decapeptide library (X_10_, where X is any amino acid). Domains that failed to select peptides with the decapeptide library were subsequently screened using a biased peptide library (X_6_-PXXP-X_6_, where P is proline). Three SH3 domain proteins (Cyk3, Lsb4, and Sla1-1/2) were also tested using a biased library containing a fixed positive charge (X_7_-R/K-X_7_, where R and K are arginine or lysine, respectively). Phage display selections were carried out as described [44]. Individual binding clones were tested for positive interactions with cognate yeast SH3 domains by phage ELISA as described [44]. The sequencing of approximately 3,000 clones resulted in the isolation of 1,871 unique peptide sequences, which were manually aligned by an expert (RT). The phage library used to select peptides for each domain is indicated in Tables S1 and S2. As some peptide files contain peptides selected from different libraries (Cyk3-class II, Lsb4, and Sla1-1/2-class II), the library from which each peptide was isolated is also indicated in each sequence file.

### Specificity Potential

For each SH3 domain, the set of peptide ligands was used to create a binding profile statistical model as a PWM. The specificity potential (*SP*) for a given column (position) of a PWM was calculated as is done for the letter height in a sequence logo [15], except normalized to range from 0 to 1 instead of 0 to 4.32 (log 20). A *SP* value of one means the given PDZ domain is completely specific for a single amino acid at that position, and a value of zero means that there is no preferred amino acid at that position. We have also included a *p*-value to assess the statistical significance of these scores. The *p*-values were computed by statistical sampling: for each PWM, we generated 10^7^ sequences of *N* randomly chosen amino acids, with *N* equal to the number of different peptides used to build the PWM. For each sequence, we computed the *SP* score, and from the distribution of *SP* scores, we computed the *p*-value of the *SP* scores for each column in the initial PWM (Table S3).

### SPOT Array Analysis

Peptide arrays were semi-automatically prepared on cellulose-(3-amino-2-hydroxy-propyl)-ether membranes [22] (CAPE membranes) using a SPOT robot (Intavis) and the standard SPOT synthesis protocol [45]. Array design was generated using the in-house software LISA. To exclude false-positive spots in the incubation experiment, all cysteine residues were replaced by serine. The CAPE membranes were used because of the better signal to noise ratio in the incubation experiments.

The peptide arrays were incubated with the GST-SH3 fusion proteins, as described [22]. Analysis and quantification of membrane-bound GST-SH3 fusion proteins was carried out using a chemiluminescence substrate and a Lumi-Imager (Roche Diagnostics). Analysis and quantification of SPOT signal intensities (*SI*) were executed with the software Genespotter (MicroDiscovery) following previously described rules [46].

### Yeast Two-Hybrid Plasmid Construction Using Homologous Recombination Cloning

DNA fragments encoding SH3 domains were amplified by PCR from a *S. cerevisiae* genomic DNA library, using sequence specific primers fused to common sequences used for homologous recombination cloning. Specifically, the forward primer was composed of the bait-specific primer and a 23-nucleotide common sequence (CGACCCCGGGAATTCAGATCTAC), which is homologous to the upstream sequence of the SpeI site on pPC97 [23]. The reverse primer was composed of the bait-specific primer and a 23-nucleotide common sequence (CGGGGACAAGGCAAGCTAAACTA), which is homologous to the 5′ of the *KanMX6* cassette. The *KanMX6* cassette was amplified by PCR with the forward primer (TTTAGCTTGCCTTGTCCC) and the reverse primer (ATAGATCTCTGCAGGTCGACGGATCCCCGGGAATTGCCATTTTTCGACACTGGATGGC), using a *KanMX6* cassette carrying plasmid, p2076, as template. Along with the PCR-amplified *KanMX6* cassette and the SpeI-cut pPC97, bait coding sequence PCR product was transformed into Y8930 (*MATα trp1-901 leu2-3,112 ura3-52 his3-200 gal4Δ gal80Δ LYS2::GAL1-HIS3 GAL2-ADE2 met2::GAL7-lacZ cyhR*), which was generated from PJ69-4α [47]. G418 positive yeast transformants were selected on SD-Leu+G418 medium, and yeast DNA was purified and transformed into *Escherichia coli* DH5-α. Constructed plasmid was purified from kanamycin-positive clone, verified by DNA sequencing, and transformed into Y8930 for Y2H screening.

### Array-Based ORFeome Y2H Screening

The whole library was assembled in an 1,536-spot array format on agar plates with each clone represented twice. ORFeome Y2H screening was performed as described [24] with some modifications. The optimal concentration of 3-amino-1,2,4-triazole (3-AT) was tested for each bait before performing the screen. SD-Leu-Trp-His+3-AT selective medium was used for screening. Plates were incubated at 30°C for 5 to 10 d before scoring positive colonies.

### gDNA Library Y2H Screening

All pOBD plasmids were taken from Tong et al. [2], and the pBDC plasmids were cloned by homologous recombination as described above (Table S9) and verified by sequencing. All bait plasmids were transformed into Y8930. A genomic DNA library [25] was transformed into Y8800 (same genotype as Y8930 except opposite mating type). Screening was performed by mating methods as described previously [48] on SD-Leu-Trp-His+3AT plates. Up to 192 positive single colonies were picked from each screen. The identity of each positive colony was determined by colony-PCR and sequencing.

### Literature Curation of Interactions Mediated by Yeast SH3 Domains

To compile a comprehensive list of yeast SH3 domain–ligand interactions supported by one or more experiments (referred to as the gold-standard set), we used a combination of automatic text mining and database searches to retrieve abstracts from the literature. The DOMINO database, specialized in domain–peptide interactions, already contained 22 entries for yeast SH3 domains, curated according to the MIMix standards from 14 papers [49],[50]. A text-mining approach looking for co-occurrence in the abstract of names of yeast proteins together with “SH3” and a list of nouns and verbs indicating interactions yielded only two papers containing relevant information after manual inspection. An additional 19 papers were captured by manual searching PubMed (http://www.ncbi.nlm.nih.gov/sites/entrez) and the *Saccharomyces* Genome Database (SGD; http://www.yeastgenome.org/) [51],[52]. These 21 new papers, which were not already present in DOMINO, were read, and the information supporting interactions mediated by SH3 domains was captured in a MIMix format. A total of 56 new interactions were added to the DOMINO database by this curation effort. Since some interactions are supported by more than one report, this amounts to a total of 41 nonredundant interactions mediated by SH3 domains and supported by at least one experiment. One paper reported a yeast two-hybrid interaction between Las17p and a protein fragment encoding both SH3 domains of Bzz1p. As the SH3 domains were not tested individually for interaction with Las17p, we counted the interaction twice to account for the two SH3 domains, thus resulting in 42 total SH3-mediated interactions [53]. The Bzz1 domains were tested individually by GST pull-down and Western blot analysis, and both domains interact with Las17p (A. Soulard and B. Winsor, personal communication). The curated gold-standard list is contained in Table S11.

### PWM Scores Matches for Affinity Correlation

The peptides from phage display were converted into position weight matrices (PWMs) by calculating the probability of occurrence for each amino acid at each position. Despite the large number of peptide sequences, we still substantially undersampled sequence space, and hence added pseudocounts. We scaled the number of pseudocounts added by the entropy of each position [54]. Each matrix was used to scan the yeast proteome to identify the best matches. We used the MOTIPS analysis pipeline to identify possible binders for each domain. Only the proteome-scanning module of the pipeline was utilized, which performs a highly optimized search in the yeast proteome for optimal matches to a given PWM. It works in an analogous fashion to earlier proteome scanners (e.g., the Scansite server) [55].

### Data Integration

We employed the Bayesian Network algorithm as implemented in the WEKA 3.4.13 Java libraries [56]. All pre- and post-processing of the data was carried out using custom code written in Perl and Java. Bayesian networks can efficiently integrate different types of data and accurately estimate the probability of interactions based on different experiments [27]. The different data sources were first preprocessed as follows: the Y2H hits were put in one of two bins, depending on whether the associated clone was found once or more than once and given scores of one or two, respectively. The resulting discrete data were then fed directly into the learning algorithm. In the preprocessing step, the SPOT peptide binding data was discretized into four bins. The discretized data were then used as one feature of the learning algorithm.

To ensure a reliable set of gold-standard true-positive interactions for efficient machine learning, we used the curated list of 42 bona fide domain–peptide interactions for the yeast SH3 domains deposited in the DOMINO database, as described above [49]. We generated the true-negative set by using the “random with constraints” logic. Specifically, we included only pairs of proteins where protein A is annotated to localize to the cell membrane and where protein B is annotated to localize to the nucleus. Proteins with overlapping annotations were excluded as well. Although the first member of each gold-standard negative set was chosen to be one of the proteins containing SH3 domains, its interacting partner was under no such constraint. Since the proportion of real interactions is very low in the space of possible interactions, one can use random domain–ligand pairs to get a set likely to contain only negatives. However, we improved upon this set by filtering out only those pairs that do not occur in known interaction databases and are annotated to occur in nonadjacent cellular compartments. Specifically, we included only pairs of proteins where protein A is annotated to localize to the cell membrane and where protein B is annotated to localize to the nucleus. Proteins with overlapping annotations were excluded as well.

Performance of each data source was evaluated using the AUC (area under the curve) in the ROC curve. This corresponds to an evaluation of how well each data source corresponds to the gold-standard data. Finally, the performance of the Bayesian data integration was assessed using the AUC in a ROC curve analysis with 10-fold cross-validation. Ten-fold cross-validation corresponds to splitting the gold standard into a training (9/10) and a testing (1/10) set ten times in a rotating fashion and evaluating its accuracy for each split. Using the gold-standard set, we classified the discretized input data into the “True” (interacting) and “False” (noninteracting) labels as well as a probability score of the interaction. We report all interactions assigned a probability score of >0.6 (Table S12). The networks were created using Cytoscape 2.6 [57]. On the basis of affinity data for the Sho1p and Abp1p SH3 domains, we estimate that this cutoff represents a dissociation constant of approximately *K*_d_ = 1.5 µM.

### Imaging of Endocytosis

Yeast strains were grown at 25°C in standard rich medium (YPD) or synthetic medium (SD) supplemented with appropriate amino acids. GFP tags were integrated chromosomally to generate C-terminal fusions of each protein, as described [58]. All strains expressing fluorescent fusion proteins had growth properties similar to the corresponding untagged strains.

For microscopy, cells were grown in SD medium without tryptophan (to minimize autofluorescence) at 25°C until early log phase. Cells were attached to coverslips coated with concanavalin A, which were sealed to slides with vacuum grease (Dow Corning). Imaging was done at room temperature using an Olympus IX81 or IX71 microscope equipped with 100× NA 1.4 objectives, and Orca II cameras (Hamamatsu). Simultaneous two-color imaging was done using an image splitter (Optical Insight) to separate the red and green emission signals to two sides of the camera sensor using a 565-nm dichroic mirror, and 530/30-nm and 630/50-nm emission filters. To excite GFP or RFP, we used a 488-nm Argon ion laser (Melles Griot) or a mercury lamp filtered through a 575/20-nm filter, respectively. The excitation beams from these two light sources were combined using a beam splitter. After each experiment, images of immobilized microbeads that fluoresce at both green and red wavelengths were captured. These images were used to align the cell images.

Image analysis was done as described [30]. Tracking of patches was done from single-color GFP movies to achieve the best signal-to-noise ratio. ImageJ (http://rsbweb.nih.gov/ij/) was used for general manipulation of images and movies.

## Supporting Information

Figure S1 **Yeast SH3 domain specificity map with distances.** PWMs were generated using phage-derived binding peptides, and a PWM-based scoring algorithm was used to search the yeast proteome for closely matching sequences, which were subsequently aligned in an unrooted clustergram. The specificity profile for each SH3 domain is represented next to the name. The SH3 domain specificity classes are colored as follows: I (red), II (blue), and III (green). Specificity profiles that could not be assigned to any class are shown in black. Underlined names indicate domains that exhibit two distinct specificity profiles. The distances on the map represent Euclidean distances based on the feature vector from the endogenous peptides.
(0.92 MB EPS)

Figure S2 **Phage-derived specificity profiles correlate with binding affinities.** In vitro affinity data for binding to the SH3 domain of Abp1p were used to calculate the differences in Gibbs free energy (ΔΔ*G*) for various peptides targeted relative to a reference peptide (KKTKPPVPPKPSHLKPK; *y*-axis), and these were plotted against the score match to the phage-derived PWM (*x*-axis).
(0.30 MB EPS)

Figure S3 **Gold-standard interactions identified by yeast two-hybrid.** The number of yeast two-hybrid interactions identified in the ORFeome (blue line) and gDNA (red) screening approaches were examined for interactions recapitulated in the gold-standard set (green).
(1.51 MB EPS)

Figure S4 **Performance analysis following redundancy reduction.** ROCs were plotted against a gold-standard set of interactions following exclusion of all domains with highly similar binding profiles. The Bayesian model was retrained and its accuracy assessed by 10-fold cross-validation.
(0.25 MB EPS)

Figure S5 **Bayesian model is unbiased toward the experimental technique used in the gold-standard interactions.** ROCs were plotted following bootstrapping of the gold standard positive set to exclude interactions that were only found by one specific technique. The plot denoted by “All” represents the ROC curve for the Bayesian model incorporating all interactions from the gold-standard set, whereas each of the other plots is based on the exclusion of interaction data from the gold-standard set based on a given experimental technique: mutagenesis (Mutag), coimmunoprecipitation (CoIP), overlay assay (Overlay), fluorescence titration (Fluor), pull-down (Pulld), alanine substitution analysis (Subst), and yeast two-hybrid (Twohy). *p*-values are calculated based on 10-fold cross-validation.
(0.32 MB EPS)

Figure S6 **Yeast SH3 domain interaction network.** Network representation of SH3 domain mediated interactions predicted by the Bayesian model. SH3 domains are represented as diamonds with their associated interactors shown in magenta circles. Although some SH3 domains recognize more than one class of peptides, each SH3 domain is represented as a single node, therefore putative binding ligands conforming to different specificity classes are shown binding to a unique domain. Domains with putative roles in endocytosis as described in Kaksonen et al. [29] are highlighted in green.
(0.96 MB EPS)

Figure S7 **Some proteins are targeted by multiple SH3 domains.** For each SH3 domain, the fraction of interactors targeted by other SH3 domains was calculated at the level of the full-length protein (black bars) and by considering each binding site individually (grey bars).
(0.97 MB EPS)

Figure S8 **Paralogous SH3 domains account for some cross-reactivity in the interaction network.** For each SH3-containing protein, the fraction of interactors targeted by other SH3 domains was calculated at the level of the full-length protein (black bars) and by considering each binding site individually (grey bars), following merging of four pairs of closely related SH3 domains from Boi1p/Boi2p, Lsb1p/Pin3p, Lsb3p/Lsb4p, and Myo3p/Myo5p.
(0.37 MB EPS)

Figure S9 **Six-core subgraph network.** An interconnected core of 31 proteins, where each protein has at least six interactions (k-core = 6) is shown. SH3 domains are represented in diamonds with their associated interactors shown in magenta circles. Although some SH3 domains recognize more than one class of peptides, each SH3 domain is represented as a single node; therefore, putative binding ligands conforming to different specificity classes are shown binding to a unique domain. Domains with putative roles in endocytosis as described in Kaksonen et al. [29] are highlighted in green. The network is bipartite, where interactions predicted to bind to a given SH3 domain and the SH3 domain protein itself are displayed as unique nodes (e.g., Bbc1p in green circle).
(0.43 MB EPS)

Table S1 **Yeast SH3 domains used in phage-display analysis.** SH3 domains are named according to the gene name in which they were identified. SH3 domains from proteins with more than one domain are numbered from the N-terminus and demarcated from the protein name with a dash. The listed amino acid ranges indicate the length of the constructs used in this analysis and not necessarily the SH3 domain boundaries defined by computational analysis. For each domain, we list whether a stable GST fusion protein was isolated. All domains were initially screened with a random decamer peptide library (X_10_, where X is any amino acid). Domains that failed to select peptides with the X_10_ library, were screened with a biased library (X_6_-PXXP-X_6_, where P is proline). The Cyk3p and Lsb4p domains were also screened with a biased library containing a fixed charged amino acid (X_7_-R/K-X_7_, where R and K are arginine or lysine, respectively). For each construct, we list whether a stable GST fusion protein was isolated and if the latter selected peptides in the phage display analysis. Domains retested based on a fungal species sequence alignment are denoted by an asterisk (*).
(0.02 MB PDF)

Table S2 **Summary of analyzed SH3 domains with boundaries identified from fungal species alignments.** The domain boundaries for three SH3 domains were extended based on fungal species alignments. SH3 domains are named according to the gene name in which they were identified. SH3 domains from proteins with more than one domain are numbered from the N-terminus and demarcated from the protein name with a dash. The listed amino acid ranges indicate the length of the constructs used in this analysis and not necessarily the SH3 domain boundaries defined by computational analysis. Sla1-1/2 indicates the construct encoding the first two N-terminal SH3 domains from Sla1p in tandem. For each domain, we list whether a stable GST fusion protein was isolated. All domains were initially screened with a random decamer peptide library (X_10_, where X is any amino acid). Domains that failed to select peptides with the X_10_ library, were screened with a biased library (X_6_-PXXP-X_6_, where P is proline). The Sla1-1/2 construct was also screened with a biased library containing a fixed-charged amino acid (X_7_-R/K-X_7_, where R and K are arginine or lysine, respectively). The library or libraries used to select peptides for each domain are indicated. For each construct, we list whether a stable GST fusion protein was isolated and if the latter selected peptides in the phage display analysis.
(0.02 MB PDF)

Table S3 **Position-specific** ***SP*** **scores.** *SP* scores are shown for every position in the PWM for each SH3 domain. A *p*-value based on randomized peptides (see Materials and Methods) is also represented for each position.
(0.04 MB PDF)

Table S4 **Sho1p-SH3 peptide-ligand affinities with associated PWM scores and ΔΔ** ***G*** **values.** Sho1p-SH3 ligand affinities were taken from Zarrinpar et al. [20], and for each ligand a PWM score was calculated based on the phage-derived specificity profile. ΔΔ*G* values were taken as −RT ln (*K*_d_ Peptide 1/*K*_d_ Peptide 2) and were calculated relative to a reference peptide (IRSKPLPPLPV).
(0.03 MB PDF)

Table S5 **Abp1p-SH3 peptide-ligand affinities and associated PWM scores and ΔΔ** ***G*** **values.** Abp1p-SH3 ligand affinities were taken from Stollar et al. [21], and for each ligand, a PWM score was calculated based on the phage-derived specificity profile. ΔΔ*G* values were taken as −RT ln (*K*_d_ Peptide 1/*K*_d_ Peptide 2) and were calculated relative to a reference peptide (KKTKPPVPPKPSHLKPK).
(0.03 MB PDF)

Table S6 **Predicted yeast SH3 domain ligands based on regular expressions.** A set of 15 regular expression patterns were used to scan the yeast proteome for predicted SH3 domain ligands. This analysis identified 2,953 peptides within 1,693 proteins. The regular expression pattern, matching ligand sequence, location within the ORF (Start and End), including the common gene name are shown for each predicted ligand.
(0.35 MB PDF)

Table S7 **SPOT intensities for yeast SH3 domain ligands predicted by regular expressions.** The peptides predicted by the set of regular expressions (Table S6) were tested against 26 GST-SH3 fusion proteins by SPOT. From the panel of 2,953 predicted ligands, 295 showed a positive signal with at least one SH3 domain (numbered from 1 to 295). These ligands were re-arrayed and retested. The signal intensity of each predicted ligand against each GST-SH3 fusion protein is reported. Each array also contains a control peptide (LASDLIVPRR that reacts with the anti-GST antibody), which has been spotted in pentuplicate at the top left, top right, and bottom right of the array. In addition, GST was tested as a control to identify nonspecific interactions. The blot from the SPOT experiment is shown for each domain.
(1.09 MB PDF)

Table S8 **SPOT intensities for the top ten highest scoring yeast SH3 domain ligands based on PWM matches.** SPOT intensities were measured for the ten best-predicted matches for each SH3 domain specificity profile based on a PWM scoring algorithm. For domains that recognized more than one set of ligands, the ten best-predicted ligands from each PWM is shown. The peptides were arrayed in the order indicated, where the 11th peptide represents a synthetic, phage-optimized peptide. The peptides were arrayed in duplicate, and the signal intensity from each SPOT experiment is shown. For domains that recognized two sets of ligands, the first row of the array represents peptide ligands predicted by the first PWM, whereas the second row contains peptide ligands predicted by the second PWM. In these cases, rows 3 and 4 of the array are duplicates of rows 1 and 2, respectively. The specificity profile used to generate each PWM shown below the blot from the SPOT experiment. Sla1-1/2-W41S and Sla1-1/2-W108S represent the two point mutations made in the Sla1-1/2 construct to determine peptide ligand interactions for each SH3 domain individually.
(0.62 MB PDF)

Table S9 **Summary of yeast SH3 domains analyzed by yeast two-hybrid.** Yeast SH3 domains were cloned into the pPC97 and pBDC/pOBD yeast two-hybrid expression vectors for use in the ORFeome and fragmented gDNA screening approaches, respectively. SH3 domains are named according to the gene name in which they were identified. SH3 domains from proteins with more than one domain are numbered from the N-terminus and demarcated from the protein name with a dash. The listed amino acid ranges indicate the length of the constructs used in this analysis and not necessarily the SH3 domain boundaries defined by computational analysis. Sla1-1/2-W41S and Sla1-1/2-W108S represent the two point mutations made in the Sla1-1/2 construct to determine the binding partners for each SH3 domain individually. Each domain was tested in both screening techniques. In most cases, the addition of a competitor of *HIS3* gene product, 3-amino-1,2,4-triazole (3-AT) was added to reduce the level of basal transcription as indicated. The number of isolated hits is indicated for each SH3 domain. The SH3 domains that could not be cloned into either expression vector are also indicated.
(0.03 MB PDF)

Table S10 **Yeast SH3 domain interactors isolated by yeast two-hybrid.** Interacting ORFs isolated by ORFeome or gDNA screening are shown for each SH3 domain, including the number of times a given interacting ORF was captured. The ORF and gene name encoded in each activation domain (AD) isolated from a positive yeast two-hybrid colony are indicated. SH3 domains are named according to the gene name in which they were identified. SH3 domains from proteins with more than one domain are numbered from the N-terminus and demarcated from the protein name with a dash. Sla1-1/2-W41S and Sla1-1/2-W108S represent the two point mutations made in the Sla1-1/2 construct to determine the binding partners for each SH3 domain individually.
(0.11 MB PDF)

Table S11 **Gold-standard yeast SH3 domain interactions.** The literature was manually curated for yeast SH3 domain-mediated interactions. The PubMed Identifier (PMID) is shown for the paper in which the interaction was identified. Each row represents a unique SH3 domain-ligand interaction with their associated UniProt identification numbers (SPID). The amino acid (A.A.) range and sequence for the interacting protein (or protein fragment) is shown along with the techniques used to identify it (Method 1, 2, or 3, where applicable). The biological relevance of the SH3-mediated interaction as described in the paper is also reported. The list contains redundant interactions as some SH3 domain mediated interactions were reported in more than one paper. A brief description of the experimental techniques listed is provided as follows: 1) Overlay assay: the SH3 domain protein is run on SDS gel and transferred to nitrocellulose. The membrane is then probed with the interactor (or vice versa); 2) Spot synthesis overlay: peptides are chemically synthesized on array format, and the membrane is probed with GST-SH3 fusion protein; 3) Phage-display/two-hybrid: random nonapeptides are selected with phage display and compared with yeast two-hybrid analysis to map the peptide; 4) Alanine scanning: each amino acid of the binding peptide is substituted with Ala and the effect of the mutation on SH3 binding is analyzed; 5) Mutagenesis analysis: each amino acid of the binding peptide is mutated with any other amino acid and the effects of the mutation on SH3 binding are analyzed; 6) Co-IP: the SH3 domain is immunoprecipitated and the presence of interactors in the complex is revealed by western blot analysis with specific antibodies; and 7) Affinity coprecipitation: the SH3 domain is precipitated with an affinity column (GST column) and the presence of interactors in the complex is revealed in Western blot analysis with specific antibodies.
(0.09 MB PDF)

Table S12 **Predicted yeast SH3 domain interactions based on Bayesian networks.** The yeast proteome was scanned for putative SH3 domain interactions based on our Bayesian model. The Bayesian model assigns a probability score based on how well a given interaction scored across all three independent techniques, where higher scores represent higher confidence predictions. To obtain a wider spectrum of scores, the probability scores were scaled by taking −log(1 − Probability score). The best matching peptide based on a PWM scoring algorithm from each predicted gene is shown.
(0.11 MB PDF)

Table S13 **Protein dynamics and interaction scores for endocytosis proteins in yeast.** The modular localization is represented for all proteins with characterized dynamics during endocytosis. The proteins are separated into their known endocytic modules. For each protein, the total predicted SH3-mediated interaction score (taken by summing all associated Bayesian probability scores) for every endocytic module (abbreviations for the respective modules are as follows: C, coat; W/M, WASP/Myo; S, scission; A, actin) is calculated. The time frame represents the point at which the protein appears (START) and is no longer observed (END) during endocytosis, with the lifetime taken as the difference between the two time points. Each protein was predicted to be part of the module for which it obtained the highest interaction score (highlighted in yellow). Cross-module proteins were considered to be proteins with interaction scores for a particular module that did not exceed the median interaction score across all modules by more than 2-fold. We could not predict the localization of Rvs167p because its corresponding scission module is only comprised of two proteins, itself and Rvs161p, which is not predicted to bind to any SH3 domain proteins required during endocytosis. To reduce the effect of weak interactions, predictions were only made for proteins that had module interactions scores of five and higher. Based on this criterion, the modular localization of some proteins could not be predicted (N/A).
(0.03 MB PDF)

Text S1 **Supporting** **Materials and Methods** **.**
(0.05 MB DOC)

## Abbreviations

AUC: area under the curve
gDNA: genomic DNA
PRM: peptide recognition module
PWM: position weight matrix
RE: regular expression
ROC: receiver-operating characteristic
*SP*: specificity potential
*SP*^t^: total specificity potential
WISE: whole interactome scanning experiment
ΔΔG: difference in Gibbs free energy
