# Supplementary material for: Bayesian Modeling of the Yeast SH3 Domain Interactome Predicts Spatiotemporal Dynamics of Endocytosis Proteins
Source: PLoS Biol. 2009 Oct 20;7(10):e1000218. doi: 10.1371/journal.pbio.1000218 (PMC2756588; doi:10.1371/journal.pbio.1000218)
Supplement: Table S1 — Yeast SH3 domains used in phage-display analysis. SH3 domains are named according to the gene name in which they were identified. SH3 domains from proteins with more than one domain are numbered from the N-terminus and demarcated from the protein name with a dash. The listed amino acid ranges indicate the length of the constructs used in this analysis and not necessarily the SH3 domain boundaries defined by computational analysis. For each domain, we list whether a stable GST fusion protein was isolated. All domains were initially screened with a random decamer peptide library (X10, where X is any amino acid). Domains that failed to select peptides with the X10 library, were screened with a biased library (X6-PXXP-X6, where P is proline). The Cyk3p and Lsb4p domains were also screened with a biased library containing a fixed charged amino acid (X7-R/K-X7, where R and K are arginine or lysine, respectively). For each construct, we list whether a stable GST fusion protein was isolated and if the latter selected peptides in the phage display analysis. Domains retested based on a fungal species sequence alignment are denoted by an asterisk (*). (0.02 MB PDF) [file pbio.1000218.s010.pdf]

Table S1. Yeast SH3 domains used in phage display analysis

SH3 domain	ORF	SH3 domain boundaries		Purified	Library
		Start	End		
Abp1	YCR088W	535	592	YES	X10
Bbc1	YJL020C	1	78	YES	X10
Bem1 -1	YBR200W	55	141	YES	X10
Bem1-2*	YBR200W	139	227	NO	NO
Boi1	YBL085W	1	86	YES	X10
Boi2	YER114C	32	116	YES	X10
Bud14*	YAR014C	247	328	NO	NO
Bzz1-1	YHR114W	478	572	YES	X10
Bzz1-2	YHR114W	568	633	YES	X10
Cdc25	YLR310C	50	136	YES	NO
Cyk3	YDL117W	1	83	YES	PXXP, R/K
Fus1	YCL027W	432	512	YES	X10
Hof1	YMR032W	590	669	YES	X10
Myo3	YKL129C	1119	1195	YES	X10
Myo5	YMR109W	1072	1160	YES	X10
Nbp2	YDR162C	103	180	YES	PXXP
Pex13	YLR191W	295	386	YES	X10
Rvs167	YDR388W	419	482	YES	X10
Sho1	YER118C	287	367	YES	X10
Sla1-1*	YBL007C	1	79	NO	NO
Sla1-2*	YBL007C	60	140	YES	NO
Sla1-3	YBL007C	344	424	YES	X10
Lsb1	YGR136W	45	122	YES	X10
Lsb3	YFR024C-A	385	451	YES	X10
Hse1	YHL002W	208	285	YES	X10
Pin3	YPR154W	53	122	YES	X10
Lsb4	YHR016C	402	468	YES	PXXP, R/K
