# Supplementary material for: Bayesian Modeling of the Yeast SH3 Domain Interactome Predicts Spatiotemporal Dynamics of Endocytosis Proteins
Source: PLoS Biol. 2009 Oct 20;7(10):e1000218. doi: 10.1371/journal.pbio.1000218 (PMC2756588; doi:10.1371/journal.pbio.1000218)
Supplement: Table S2 — Summary of analyzed SH3 domains with boundaries identified from fungal species alignments. The domain boundaries for three SH3 domains were extended based on fungal species alignments. SH3 domains are named according to the gene name in which they were identified. SH3 domains from proteins with more than one domain are numbered from the N-terminus and demarcated from the protein name with a dash. The listed amino acid ranges indicate the length of the constructs used in this analysis and not necessarily the SH3 domain boundaries defined by computational analysis. Sla1-1/2 indicates the construct encoding the first two N-terminal SH3 domains from Sla1p in tandem. For each domain, we list whether a stable GST fusion protein was isolated. All domains were initially screened with a random decamer peptide library (X10, where X is any amino acid). Domains that failed to select peptides with the X10 library, were screened with a biased library (X6-PXXP-X6, where P is proline). The Sla1-1/2 construct was also screened with a biased library containing a fixed-charged amino acid (X7-R/K-X7, where R and K are arginine or lysine, respectively). The library or libraries used to select peptides for each domain are indicated. For each construct, we list whether a stable GST fusion protein was isolated and if the latter selected peptides in the phage display analysis. (0.02 MB PDF) [file pbio.1000218.s011.pdf]

Table S2. Summary of analyzed SH3 domains with boundaries identified from fungal species alignments

		SH3 domain boundaries			
**SH3 domain**	**ORF**	**Start**	**End**	**Purified**	**Library**
Bem1-2	YBR200W	155	261	YES	PXXP
Bud14	YAR014C	237	347	YES	NO
Sla1-1/2	YBL007C	1	150	YES	PXXP,R/K
Sla1-1/2-W41S	YBL007C	1	150	YES	PXXP,R/K
Sla1-1/2-W108S	YBL007C	1	150	YES	PXXP,R/K
