# Supplementary material for: Bayesian Modeling of the Yeast SH3 Domain Interactome Predicts Spatiotemporal Dynamics of Endocytosis Proteins
Source: PLoS Biol. 2009 Oct 20;7(10):e1000218. doi: 10.1371/journal.pbio.1000218 (PMC2756588; doi:10.1371/journal.pbio.1000218)
Supplement: Table S3 — Position-specific SP scores. SP scores are shown for every position in the PWM for each SH3 domain. A p-value based on randomized peptides (see Materials and Methods) is also represented for each position. (0.04 MB PDF) [file pbio.1000218.s012.pdf]

Table S3

Table S3. Position-specific *SP* scores

Domain	Position	Specificity score	*p*-value
Abp1	0	0.99535	<0.000001
Abp1	1	0.33885	<0.000001
Abp1	2	0.58262	<0.000001
Abp1	3	0.82347	<0.000001
Abp1	4	0.10517	<0.000001
Abp1	5	0.99535	<0.000001
Abp1	6	0.92272	<0.000001
Abp1	7	0.1583	<0.000001
Abp1	8	0.43119	<0.000001
Abp1	9	0.92272	<0.000001
Bbc1	0	0.46649	<0.000001
Bbc1	1	0.87379	<0.000001
Bbc1	2	0.26762	<0.000001
Bbc1	3	0.60896	<0.000001
Bbc1	4	0.81122	<0.000001
Bbc1	5	0.33352	<0.000001
Bbc1	6	0.98092	<0.000001
Bbc1	7	0.9973	<0.000001
Bbc1	8	0.1607	<0.000001
Bbc1	9	0.17532	<0.000001
Bbc1	10	0.2961	<0.000001
Bem1-1	0	0.40893	<0.000001
Bem1-1	1	0.87283	<0.000001
Bem1-1	2	0.36514	<0.000001
Bem1-1	3	0.87283	<0.000001
Bem1-1	4	0.21549	<0.000001
Bem1-1	5	0.99365	<0.000001
Bem1-1	6	0.92944	<0.000001
Bem1-1	7	0.17709	<0.000001
Bem1-2	0	0.48266	<0.000001
Bem1-2	1	0.29364	<0.000001
Bem1-2	2	0.53048	<0.000001
Bem1-2	3	0.41048	<0.000001
Bem1-2	4	0.17207	0.000003
Bem1-2	5	0.48266	<0.000001
Bem1-2	6	0.30663	<0.000001
Boi1	0	0.32049	<0.000001
Boi1	1	0.88473	<0.000001
Boi1	2	0.25591	<0.000001
Boi1	3	0.77151	<0.000001
Boi1	4	0.60292	<0.000001
Boi1	5	0.40695	<0.000001
Boi1	6	0.40411	<0.000001
Boi1	7	0.92485	<0.000001
Boi1	8	0.28389	<0.000001
Boi2	0	0.27193	<0.000001

Table S3

Domain	Position	Specificity score	*p*-value
Boi2	1	0.62812	<0.000001
Boi2	2	0.25535	<0.000001
Boi2	3	0.23722	<0.000001
Boi2	4	0.38895	<0.000001
Boi2	5	0.4197	<0.000001
Boi2	6	0.38723	<0.000001
Boi2	7	0.88327	<0.000001
Bzz1-1	0	0.7473	<0.000001
Bzz1-1	1	0.22082	<0.000001
Bzz1-1	2	0.60152	<0.000001
Bzz1-1	3	0.99354	<0.000001
Bzz1-1	4	0.99354	<0.000001
Bzz1-1	5	0.64835	<0.000001
Bzz1-1	6	0.51358	<0.000001
Bzz1-1	7	0.99342	<0.000001
Cyk3-ClassI	0	0.14314	0.000149
Cyk3-ClassI	1	0.97697	<0.000001
Cyk3-ClassI	2	0.12669	0.000392
Cyk3-ClassI	3	0.2366	<0.000001
Cyk3-ClassI	4	0.45374	<0.000001
Cyk3-ClassI	5	0.21431	<0.000001
Cyk3-ClassI	6	0.14173	0.000149
Cyk3-ClassI	7	0.64491	<0.000001
Cyk3-ClassI	8	0.10775	0.001706
Cyk3-ClassII	0	0.24103	<0.000001
Cyk3-ClassII	1	0.31062	<0.000001
Cyk3-ClassII	2	0.99289	<0.000001
Cyk3-ClassII	3	0.26188	<0.000001
Cyk3-ClassII	4	0.60627	<0.000001
Cyk3-ClassII	5	0.91848	<0.000001
Cyk3-ClassII	6	0.26102	<0.000001
Cyk3-ClassII	7	0.81065	<0.000001
Cyk3-ClassII	8	0.91848	<0.000001
Cyk3-ClassII	9	0.99289	<0.000001
Cyk3-ClassII	10	0.4766	<0.000001
Fus1	0	0.2743	<0.000001
Fus1	1	0.09204	0.01048
Fus1	2	0.14877	0.000098
Fus1	3	0.61371	<0.000001
Fus1	4	0.33631	<0.000001
Fus1	5	0.29259	<0.000001
Fus1	6	0.41669	<0.000001
Fus1	7	0.41669	<0.000001
Hof1	0	0.10758	<0.000001
Hof1	1	0.94779	<0.000001
Hof1	2	0.08239	<0.000001
Hof1	3	0.82295	<0.000001
Hof1	4	0.97166	<0.000001

Table S3

Domain	Position	Specificity score	*p*-value
Hof1	5	0.23872	<0.000001
Hof1	6	0.36416	<0.000001
Hof1	7	0.8691	<0.000001
Hse1	0	0.98839	<0.000001
Hse1	1	0.13928	0.000002
Hse1	2	0.38979	<0.000001
Hse1	3	0.98839	<0.000001
Hse1	4	0.15462	<0.000001
Hse1	5	0.76764	<0.000001
Hse1	6	0.12083	0.000021
Lsb1-ClassII	0	0.16684	<0.000001
Lsb1-ClassII	1	0.98753	<0.000001
Lsb1-ClassII	2	0.17072	<0.000001
Lsb1-ClassII	3	0.2747	<0.000001
Lsb1-ClassII	4	0.71189	<0.000001
Lsb1-ClassII	5	0.3195	<0.000001
Lsb1-ClassII	6	0.98753	<0.000001
Lsb1-Unique	0	0.19525	<0.000001
Lsb1-Unique	1	0.99275	<0.000001
Lsb1-Unique	2	0.42348	<0.000001
Lsb1-Unique	3	0.41832	<0.000001
Lsb1-Unique	4	0.27386	<0.000001
Lsb1-Unique	5	0.3833	<0.000001
Lsb1-Unique	6	0.63022	<0.000001
Lsb1-Unique	7	0.99275	<0.000001
Lsb3	0	0.28122	<0.000001
Lsb3	1	0.93697	<0.000001
Lsb3	2	0.31397	<0.000001
Lsb3	3	0.95633	<0.000001
Lsb3	4	0.9222	<0.000001
Lsb3	5	0.36992	<0.000001
Lsb3	6	0.90295	<0.000001
Lsb3	7	0.32857	<0.000001
Lsb4	0	0.19232	<0.000001
Lsb4	1	0.997	<0.000001
Lsb4	2	0.15482	<0.000001
Lsb4	3	0.91403	<0.000001
Lsb4	4	0.97218	<0.000001
Lsb4	5	0.14495	<0.000001
Lsb4	6	0.84223	<0.000001
Lsb4	7	0.24938	<0.000001
Myo3	0	0.17404	<0.000001
Myo3	1	0.99612	<0.000001
Myo3	2	0.10183	<0.000001
Myo3	3	0.31835	<0.000001
Myo3	4	0.20945	<0.000001
Myo3	5	0.82629	<0.000001
Myo3	6	0.99612	<0.000001

Table S3

Domain	Position	Specificity score	*p*-value
Myo3	7	0.10261	<0.000001
Myo3	8	0.38105	<0.000001
Myo3	9	0.60772	<0.000001
Myo5	0	0.17649	<0.000001
Myo5	1	0.93358	<0.000001
Myo5	2	0.12772	<0.000001
Myo5	3	0.26999	<0.000001
Myo5	4	0.2186	<0.000001
Myo5	5	0.74542	<0.000001
Myo5	6	0.85644	<0.000001
Myo5	7	0.0946	<0.000001
Myo5	8	0.34315	<0.000001
Myo5	9	0.43362	<0.000001
Nbp2	0	0.20629	<0.000001
Nbp2	1	0.99376	<0.000001
Nbp2	2	0.17705	<0.000001
Nbp2	3	0.88985	<0.000001
Nbp2	4	0.99376	<0.000001
Nbp2	5	0.16037	<0.000001
Nbp2	6	0.54591	<0.000001
Nbp2	7	0.87554	<0.000001
Nbp2	8	0.1471	<0.000001
Pex13-ClassII	0	0.7672	<0.000001
Pex13-ClassII	1	0.09952	<0.000001
Pex13-ClassII	2	0.4068	<0.000001
Pex13-ClassII	3	0.87024	<0.000001
Pex13-ClassII	4	0.07954	0.000002
Pex13-ClassII	5	0.75324	<0.000001
Pex13-ClassII	6	0.49372	<0.000001
Pex13-ClassII	7	0.50742	<0.000001
Pex13-Unique	0	0.39864	<0.000001
Pex13-Unique	1	0.0583	0.052163
Pex13-Unique	2	0.24	<0.000001
Pex13-Unique	3	0.07186	0.007649
Pex13-Unique	4	0.99086	<0.000001
Pex13-Unique	5	0.99086	<0.000001
Pex13-Unique	6	0.10197	0.000065
Pex13-Unique	7	0.99086	<0.000001
Pex13-Unique	8	0.61586	<0.000001
Pin3-ClassII	0	0.99483	<0.000001
Pin3-ClassII	1	0.56226	<0.000001
Pin3-ClassII	2	0.45585	<0.000001
Pin3-ClassII	3	0.9011	<0.000001
Pin3-ClassII	4	0.28972	<0.000001
Pin3-ClassII	5	0.99483	<0.000001
Pin3-ClassII	6	0.28958	<0.000001
Pin3-Unique	0	0.73537	<0.000001
Pin3-Unique	1	0.0971	0.000025

Table S3

Domain	Position	Specificity score	*p*-value
Pin3-Unique	2	0.91634	<0.000001
Pin3-Unique	3	0.25363	<0.000001
Pin3-Unique	4	0.19441	<0.000001
Pin3-Unique	5	0.31927	<0.000001
Pin3-Unique	6	0.56413	<0.000001
Pin3-Unique	7	0.52471	<0.000001
Pin3-Unique	8	0.73537	<0.000001
Rvs167-ClassI	0	0.98704	<0.000001
Rvs167-ClassI	1	0.42252	<0.000001
Rvs167-ClassI	2	0.57618	<0.000001
Rvs167-ClassI	3	0.82329	<0.000001
Rvs167-ClassI	4	0.10805	0.000217
Rvs167-ClassI	5	0.38862	<0.000001
Rvs167-ClassI	6	0.82329	<0.000001
Rvs167-ClassII	0	0.22297	<0.000001
Rvs167-ClassII	1	0.99735	<0.000001
Rvs167-ClassII	2	0.25816	<0.000001
Rvs167-ClassII	3	0.99735	<0.000001
Rvs167-ClassII	4	0.99735	<0.000001
Rvs167-ClassII	5	0.27182	<0.000001
Rvs167-ClassII	6	0.99735	<0.000001
Rvs167-ClassII	7	0.14662	<0.000001
Sho1	0	0.24573	<0.000001
Sho1	1	0.4223	<0.000001
Sho1	2	0.41005	<0.000001
Sho1	3	0.85509	<0.000001
Sho1	4	0.99572	<0.000001
Sho1	5	0.30103	<0.000001
Sho1	6	0.63905	<0.000001
Sho1	7	0.92156	<0.000001
