# Supplementary material for: Bayesian Modeling of the Yeast SH3 Domain Interactome Predicts Spatiotemporal Dynamics of Endocytosis Proteins
Source: PLoS Biol. 2009 Oct 20;7(10):e1000218. doi: 10.1371/journal.pbio.1000218 (PMC2756588; doi:10.1371/journal.pbio.1000218)
Supplement: Table S4 — Sho1p-SH3 peptide-ligand affinities with associated PWM scores and ΔΔ G values. Sho1p-SH3 ligand affinities were taken from Zarrinpar et al. [20], and for each ligand a PWM score was calculated based on the phage-derived specificity profile. ΔΔG values were taken as −RT ln (K d Peptide 1/K d Peptide 2) and were calculated relative to a reference peptide (IRSKPLPPLPV). (0.03 MB PDF) [file pbio.1000218.s013.pdf]

Table S4

Table S4. Sho1p-SH3 peptide ligand affinities and associated PWM and ΔΔ*G* values

Peptide	Ligand sequence	Kd (μM)	PWM score	ΔΔ*G*
1	IRSKPLPPLPV	1	0.0	0.0
2	IVNKPLPPLPV	1.3	0.0	0.2
3	IVNRPLPPLPV	2.2	0.2	0.5
4	IRSKPLPLTPN	8.3	1.3	1.3
5	IRSRALPPLPV	13	1.3	1.5
6	IKSRVLPPLPV	30	1.4	2.0
7	IVNKPLAPLPV	>50	2.6	3.7
