# Supplementary material for: Bayesian Modeling of the Yeast SH3 Domain Interactome Predicts Spatiotemporal Dynamics of Endocytosis Proteins
Source: PLoS Biol. 2009 Oct 20;7(10):e1000218. doi: 10.1371/journal.pbio.1000218 (PMC2756588; doi:10.1371/journal.pbio.1000218)
Supplement: Table S5 — Abp1p-SH3 peptide-ligand affinities and associated PWM scores and ΔΔ G values. Abp1p-SH3 ligand affinities were taken from Stollar et al. [21], and for each ligand, a PWM score was calculated based on the phage-derived specificity profile. ΔΔG values were taken as −RT ln (K d Peptide 1/K d Peptide 2) and were calculated relative to a reference peptide (KKTKPPVPPKPSHLKPK). (0.03 MB PDF) [file pbio.1000218.s014.pdf]

Table S5. Abp1p-SH3 peptide ligand affinities and associated PWM and ΔΔ*G* values

Peptide	Ligand Sequence	Kd (μM)	PWM	ΔΔ*G*
1	KKTKPPVPPKPSHLKPK	0.12	0.27	0.00
2	KSRPPRPPPKPLHLRTE	0.21	1.24	0.33
3	KKTKPTPPPKPSHLKPK	0.27	0.86	0.48
4	KKTKPTPAPKPSHLKPK	0.81	1.70	1.14
5	KKPRPPVKSKPKHLQDG	0.88	1.52	1.19
6	KKTKPTAPPKPSHLKPK	0.96	1.25	1.24
7	KKTKPTPPPKPSALKPK	0.96	1.28	1.24
8	KKTKPTPPPKASHLKPK	1.75	1.44	1.60
9	KSGPPPRPKKPSTLKTK	4.43	1.77	2.15
10	KKTKPTPPPKPSHAKPK	5.7	1.67	2.30
11	KPEKPPVVKKPHYLSVA	6.1	1.68	2.34
12	KKTKATPPPKPSHLKPK	10.5	1.95	2.67
13	KKTKPTPPPAPSHLKPK	31.1	1.95	3.31
