# Supplementary material for: Bayesian Modeling of the Yeast SH3 Domain Interactome Predicts Spatiotemporal Dynamics of Endocytosis Proteins
Source: PLoS Biol. 2009 Oct 20;7(10):e1000218. doi: 10.1371/journal.pbio.1000218 (PMC2756588; doi:10.1371/journal.pbio.1000218)
Supplement: Table S6 — Predicted yeast SH3 domain ligands based on regular expressions. A set of 15 regular expression patterns were used to scan the yeast proteome for predicted SH3 domain ligands. This analysis identified 2,953 peptides within 1,693 proteins. The regular expression pattern, matching ligand sequence, location within the ORF (Start and End), including the common gene name are shown for each predicted ligand. (0.35 MB PDF) [file pbio.1000218.s015.pdf]

Table S6. Predicted yeast SH3 domain ligands based on regular expressions

Motif	Motif Match	Start	End	ORF	Common name
...R..[FLIYM].[FLIYM]P.....	VSGRKIIFLPTVGED	615	629	YAL001C	TFC3
...R..[FLIYM].[FLIYM]P.....	GQYRRTIVIPRRFFT	126	140	YAL009W	SPO7
......P.[ILMVPYAFTR]P.[RKW]...	TENGNIPVFPAKFGI	473	487	YAL010C	MDM10
......P.[ILMVPYAFTR]P.[RKW]...	SVTTTRPAPPSKAKD	331	345	YAL011W	SWC1
...[KR][KR][AHPKRLG]P[PLV].......	AILRKRPLVKTETGP	11	25	YAL015C	NTG1
.[FPLWA].[WYLMFHP].[AVLIMFHPR]P..P.....	MLQFLPPDDPRSSLD	322	336	YAL015C	NTG1
...[RK].[AVLIMFHRTP]P..P.....	ILNRCVPKRPTIDDI	1334	1348	YAL017W	PSK1
.[FPLWA].[WYLMFHP].[AVLIMFHPR]P..P.....	LFNLDHPMVPENLVE	293	307	YAL018C	YAL018C
...PPPP...	SAEPTPAPAPAPVER	451	465	YAL019W	FUN30
..R[YFLEP]..[AVLIMFHWRTP]P[GSDLIAV]P.....	DWRPVEVPAPVVDVA	79	93	YAL020C	ATS1
...R..[FLIYM].[FLIYM]P.....	RDNRPEIPLPHERRF	459	473	YAL021C	CCR4
...[RK].[AVLIMFHRTP]P..P.....	AVDKIMPPTPAKKVE	309	323	YAL024C	LTE1
...[RK].[AVLIMFHRTP]P..P.....	LNDRQIPLSPDQMIL	388	402	YAL026C	DRS2
...R..[FLIYM].[FLIYM]P.....	VIKRENLSLPGKMEI	1416	1430	YAL029C	MYO4
...R..[FLIYM].[FLIYM]P.....	YVLRNVLRIPEEYLD	86	100	YAL034W-A	MTW1
...[RK].[AVLIMFHRTP]P..P.....	LVSKYRPNCPIILVT	410	424	YAL038W	CDC19
..[KRP]......P.[KR]P..	NARKSEELYPGRPLA	66	80	YAL038W	CDC19
...[RK].[AVLIMFHRTP]P..P.....	RSKRLFPVRPMATAH	536	550	YAL040C	CLN3
....RP[AVLIMFHRKTP][AVLIMFHRTP][AVLIMFHRTP]..[LP]...	LFPVRPMATAHPCSA	540	554	YAL040C	CLN3
...[RK].[AVLIMFHRTP]P..P.....	RLFKCTPGLPKLWTS	357	371	YAL048C	GON1
...[RK].[AVLIMFHRTP]P..P.....	DPGKNTPGLPEETAA	611	625	YAL048C	GON1
...R..[FLIYM].[FLIYM]P.....	FAARGDISIPAVKYA	214	228	YAL049C	YAL049C
......P.[ILMVPYAFTR]P.[RKW]...	VSKQPAPRIPSKDAI	97	111	YAL051W	OAF1
......P.[ILMVPYAFTR]P.[RKW]...	FVEENFPIVPLKSVA	635	649	YAL051W	OAF1
...[RK].[AVLIMFHRTP]P..P.....	TSVKIVPQRPISDRL	44	58	YAL054C	ACS1
..[KRP]......P.[KR]P..	VTPLAGGVTPMKPGS	479	493	YAL054C	ACS1
...PPPP...	FEPPITPPLMIPDVS	238	252	YAL058W	CNE1
...R..[FLIYM].[FLIYM]P.....	RDERDPLMIPHPDGT	276	290	YAL058W	CNE1
...[RK].[AVLIMFHRTP]P..P.....	WGPKPVPFQPMDVTL	293	307	YAL060W	BDH1
......P.[ILMVPYAFTR]P.[RKW]...	RASATVPSAPYRKQI	8	22	YAR002W	NUP60
......P.[ILMVPYAFTR]P.[RKW]...	NATKISPSAPSKDSF	347	361	YAR002W	NUP60
..[KRP]......P.[KR]P..	SIKLWDLSKPSKPLK	95	109	YAR003W	SWD1
...PPPP...	DLPLPDLPPESPTEF	571	585	YAR009C	YAR009C
.[FPLWA].[WYLMFHP].[AVLIMFHPR]P..P.....	IADLPLPDLPPESPT	569	583	YAR009C	YAR009C
.[FPLWA].[WYLMFHP].[AVLIMFHPR]P..P.....	LPKLNVPLNPKGKKL	934	948	YAR009C	YAR009C
...R..[FLIYM].[FLIYM]P.....	GSVRKVIHIPTKKLL	37	51	YAR018C	KIN3
...R..[FLIYM].[FLIYM]P.....	SKSRLLIELPEGFFT	607	621	YAR019C	CDC15
...[RK].[AVLIMFHRTP]P..P.....	PNLKRLPIPPLQDTL	2	16	YAR035W	YAT1
.....[GP]P.[IVL].P[FWY]...	TLNALPPHLIPYLAP	1089	1103	YAR042W	SWH1
......P.[ILMVPYAFTR]P.[RKW]...	TPRLSLPRLPNKHHW	113	127	YAR068W	YAR068W
..[KRP]....P...[KR]P..	KKPASKAPAEKKPAA	7	21	YBL002W	HTB2
......P.[ILMVPYAFTR]P.[RKW]...	SEMSNSPQIPKKKVK	1822	1836	YBL004W	UTP20
[FPLWA]..[WYLMFHP].[AVLIMFHPP]P..P.....	LHQLGAPSIPENLAN	2281	2295	YBL004W	UTP20
...PPPP...	ADLPLPDLPPEPPTE	1129	1143	YBL005W-B	YBL005W-B
...PPPP...	PLPDLPPEPPTELSD	1132	1146	YBL005W-B	YBL005W-B
.[FPLWA].[WYLMFHP].[AVLIMFHPR]P..P.....	IADLPLPDLPPEPPT	1128	1142	YBL005W-B	YBL005W-B
.[FPLWA].[WYLMFHP].[AVLIMFHPR]P..P.....	LPLPDLPPEPPTELS	1131	1145	YBL005W-B	YBL005W-B
.[FPLWA].[WYLMFHP].[AVLIMFHPR]P..P.....	IPKLNVPLNPKGRKL	1493	1507	YBL005W-B	YBL005W-B
....P.R.A[VP].....	KVSKPKRVAVHGYLG	54	68	YBL006C	LDB7
...PPPP...	EEGPPPAMPARPTAT	190	204	YBL007C	SLA1
...PPPP...	ELPPIKPPRPTSTTS	619	633	YBL007C	SLA1
......P.[ILMVPYAFTR]P.[RKW]...	DEEGPPPAMPARPTA	189	203	YBL007C	SLA1
..[KRP]......P.[KR]P..	QEKELPPIKPPRPTS	616	630	YBL007C	SLA1
.[FPLWA].[WYLMFHP].[AVLIMFHPR]P..P.....	GPPPAMPARPTATTE	192	206	YBL007C	SLA1
...[RK].[AVLIMFHRTP]P..P.....	LVLRNVPILPILNGQ	777	791	YBL008W	HIR1
...R..[FLIYM].[FLIYM]P.....	AYQRSIIKLPVTKGL	65	79	YBL008W-A	YBL008W-A
...R..[FLIYM].[FLIYM]P.....	QTLRERLYLPQNRVQ	249	263	YBL013W	FMT1
..[KRP]....P...[KR]P..	TTKKQENPQWLRPVD	33	47	YBL014C	RRN6
...[RK].[AVLIMFHRTP]P..P.....	EYIKSLPMYPAAPLE	258	272	YBL016W	FUS3
......P.[ILMVPYAFTR]P.[RKW]...	ELFLRRPIFPGRDYR	215	229	YBL016W	FUS3
...[RK].[AVLIMFHRTP]P..P.....	TKKKTVPVKPLQLVK	685	699	YBL017C	PEP1
..[KRP]......P.[KR]P..	CDKTKKKTVPVKPLQ	682	696	YBL017C	PEP1
.[FPLWA].[WYLMFHP].[AVLIMFHPR]P..P.....	GLKLDAPSSPHACPG	1366	1380	YBL017C	PEP1
.....[GP]P.[IVL].P[FWY]...	GVGNVGPNLLPYKEC	1126	1140	YBL017C	PEP1
......P.[ILMVPYAFTR]P.[RKW]...	IQFIINPDTPHRRIF	261	275	YBL019W	APN2
[FPLWA]..[WYLMFHP].[AVLIMFHPP]P..P.....	FTIWFIPLVPVFWGK	45	59	YBL029C-A	YBL029C-A
...PPPP...	LSNSPSPPPPSSSSL	154	168	YBL029W	YBL029W
...PPPP...	VKTPLPPAPAPKKES	7	21	YBL030C	PET9

Table S6

Motif	Motif Match	Start	End	ORF	Common name
....RP[AS]....Y...	MKRSRPSRSIPYTTP	134	148	YBL031W	SHE1
...R..[FLIYM].[FLIYM]P.....	SNTRIELKIPELYVG	257	271	YBL032W	HEK2
......P.[ILMVPYAFTR]P.[RKW]...	KSTDDTPSIPSKISS	1310	1324	YBL034C	STU1
...[RK].[AVLIMFHRTP]P..P.....	AVGRIVPDSPTYDKF	296	310	YBL035C	POL12
.[FPLWA].[WYLMFHP].[AVLIMFHPR]P..P.....	LFGLSIPKTPTLKKR	103	117	YBL035C	POL12
...R..[FLIYM].[FLIYM]P.....	SLIRKALQLPKRNFK	502	516	YBL035C	POL12
..[KRP]....P...[KR]P..	FDKKNETPRKRKPTD	314	328	YBL046W	YBL046W
.[FPLWA].[WYLMFHP].[AVLIMFHPR]P..P.....	VATPSIPQQPIPLKN	1305	1319	YBL047C	EDE1
..[KRP]......P.[KR]P..	SRKESKFGTPEKPLS	442	456	YBL052C	SAS3
.[FPLWA].[WYLMFHP].[AVLIMFHPR]P..P.....	TLNYAFPIKPISNDE	391	405	YBL058W	SHP1
...PPPP...	KTPVPVPDQPLPKVL	1039	1053	YBL063W	KIP1
...R..[FLIYM].[FLIYM]P.....	KKIRLIFTYPSTVGR	184	198	YBL064C	PRX1
...[RK].[AVLIMFHRTP]P..P.....	FVTRYLPYFPIMYSN	355	369	YBL066C	SEF1
......P.[ILMVPYAFTR]P.[RKW]...	FKLQLTPLLPDKYFD	690	704	YBL066C	SEF1
...R..[FLIYM].[FLIYM]P.....	ITDRKLYPLPLYNHI	820	834	YBL066C	SEF1
......P.[ILMVPYAFTR]P.[RKW]...	VETPNEPGAPSRLSF	282	296	YBL067C	UBP13
......P.[ILMVPYAFTR]P.[RKW]...	NVTDRPPDVPRKIIV	298	312	YBL067C	UBP13
..R[YFLEP]..[AVLIMFHWRTP]P[GSDLIAV]P.....	RNRYWGTPIPLWVSD	460	474	YBL076C	ILS1
...R..[FLIYM].[FLIYM]P.....	YVIRKRIMLPPEKAI	62	76	YBL078C	ATG8
...[RK].[AVLIMFHRTP]P..P.....	SINRSIPSAPYGAYN	85	99	YBL081W	YBL081W
...[RK].[AVLIMFHRTP]P..P.....	YCFKLVPPQPGSKKG	850	864	YBL085W	BOI1
..[KRP]....P...[KR]P..	YQKTFKLPWDPRPGE	310	324	YBL086C	YBL086C
....RP[AVLIMFHRKTP][AVLIMFHRTP][AVLIMFHRTP]..[LP]...	SKAKRPKFLLDLQIK	7	21	YBL086C	YBL086C
...R..[FLIYM].[FLIYM]P.....	TKFRISLGLPVGAIM	9	23	YBL087C	RPL23A
...[RK].[AVLIMFHRTP]P..P.....	RTYKVVPLGPKAGII	2502	2516	YBL088C	TEL1
...R..[FLIYM].[FLIYM]P.....	TMKRLLYKLPYDSLY	2304	2318	YBL088C	TEL1
......P.[ILMVPYAFTR]P.[RKW]...	LCYCGFPLLPSKRGV	162	176	YBL095W	YBL095W
...R..[FLIYM].[FLIYM]P.....	SKGRTKIDMPIKNRK	451	465	YBL097W	BRN1
...PPPP...	VSPQPASVPPPQNGQ	70	84	YBL101W-A	YBL101W-A
...PPPP...	VSPQPASVPPPQNGQ	70	84	YBL101W-B	YBL101W-B
.[FPLWA].[WYLMFHP].[AVLIMFHPR]P..P.....	LPKLNVPLNPKGKKL	1508	1522	YBL101W-B	YBL101W-B
....RP[AVLIMFHRKTP][AVLIMFHRTP][AVLIMFHRTP]..[LP]...	ELYIRPPPHLGLNDK	1374	1388	YBL101W-B	YBL101W-B
...[KR][KR][AHPKRLG]P[PLV].......	NAARKLPPGTKDHRY	933	947	YBL106C	SRO77
...[RK].[AVLIMFHRTP]P..P.....	RVTRKRPREPKSTND	335	349	YBL111C	YBL111C
..[KRP]......P.[KR]P..	TERLKRDLCPRKPTE	229	243	YBL111C	YBL111C
....RP[AVLIMFHRKTP][AVLIMFHRTP][AVLIMFHRTP]..[LP]...	GKKVRPLLVLLLSRA	83	97	YBR003W	COQ1
......P.[ILMVPYAFTR]P.[RKW]...	NDISLPPTPPYKAHK	350	364	YBR007C	YBR007C
.[FPLWA].[WYLMFHP].[AVLIMFHPR]P..P.....	LARYIFPPNPVFHFK	427	441	YBR007C	YBR007C
...PPPP...	ASPQPASVPPPQNGP	70	84	YBR012W-A	YBR012W-A
...PPPP...	SVPPPQNGPYPQQCM	76	90	YBR012W-A	YBR012W-A
...PPPP...	ASPQPASVPPPQNGP	70	84	YBR012W-B	YBR012W-B
...PPPP...	SVPPPQNGPYPQQCM	76	90	YBR012W-B	YBR012W-B
...PPPP...	LPLPDLPPEPPTELS	1132	1146	YBR012W-B	YBR012W-B
.[FPLWA].[WYLMFHP].[AVLIMFHPR]P..P.....	IADLPLPDLPPEPPT	1129	1143	YBR012W-B	YBR012W-B
.[FPLWA].[WYLMFHP].[AVLIMFHPR]P..P.....	IPKLNVPLNPNGRKL	1494	1508	YBR012W-B	YBR012W-B
...[RK].[AVLIMFHRTP]P..P.....	LGERFMPMAPEVYNR	619	633	YBR017C	KAP104
......P.[ILMVPYAFTR]P.[RKW]...	QLQGVYPSRPEKTYD	68	82	YBR026C	ETR1
[FPLWA]..[WYLMFHP].[AVLIMFHPP]P..P.....	LKTLAFPINPSDINQ	54	68	YBR026C	ETR1
...[KR][KR][AHPKRLG]P[PLV].......	WTQRRGPLVVYAEDN	199	213	YBR031W	RPL4A
.[FPLWA].[WYLMFHP].[AVLIMFHPR]P..P.....	PAVFSAPIRPDIVHT	23	37	YBR031W	RPL4A
..[KRP]......P.[KR]P..	KSKFTDMLDPEKPNV	383	397	YBR033W	EDS1
....RP[AVLIMFHRKTP][AVLIMFHRTP][AVLIMFHRTP]..[LP]...	PLQYRPRTHSYPMNS	115	129	YBR033W	EDS1
......P.[ILMVPYAFTR]P.[RKW]...	ILLPFIPKFPSRESV	299	313	YBR036C	CSG2
......P.[ILMVPYAFTR]P.[RKW]...	DSAHNSPVAPNRYAA	81	95	YBR038W	CHS2
......P.[ILMVPYAFTR]P.[RKW]...	ILYVTVPKLPFKLAV	178	192	YBR040W	FIG1
..[KRP]....P...[KR]P..	EYKYSRPPYNFKPLY	498	512	YBR043C	YBR043C
......P.[ILMVPYAFTR]P.[RKW]...	YSPEYSPLVPSKMCA	353	367	YBR044C	TCM62
...R..[FLIYM].[FLIYM]P.....	LMNRNDMGYPPAAAN	32	46	YBR050C	REG2
...[RK].[AVLIMFHRTP]P..P.....	SFRKILPQEPEIWII	390	404	YBR055C	PRP6
...[RK].[AVLIMFHRTP]P..P.....	SGTRLVPNCPLLWVS	693	707	YBR055C	PRP6
......P.[ILMVPYAFTR]P.[RKW]...	KHLDSLPFAPLRKVY	136	150	YBR056W	YBR056W
......P.[ILMVPYAFTR]P.[RKW]...	DPFVPPPNVPKKDKR	634	648	YBR058C	UBP14
......P.[ILMVPYAFTR]P.[RKW]...	PDPALKPKTPSKAPR	62	76	YBR060C	ORC2
......P.[ILMVPYAFTR]P.[RKW]...	SPEPPEPATPSKKSL	166	180	YBR060C	ORC2
...[KR][KR][AHPKRLG]P[PLV].......	HILKKLPLNESFLKN	185	199	YBR065C	ECM2
...R..[FLIYM].[FLIYM]P.....	DHERYKLNLPKEDEH	36	50	YBR074W	YBR074W
...[RK].[AVLIMFHRTP]P..P.....	AGNREPPSTPSTLPK	875	889	YBR079C	RPG1
...[KR][KR][AHPKRLG]P[PLV].......	VALKRKPPQDRRLLI	649	663	YBR080C	SEC18
...[RK].[AVLIMFHRTP]P..P.....	SKVRGRPMYPRPAED	423	437	YBR083W	TEC1
..[KRP]....P...[KR]P..	LSKVRGRPMYPRPAE	422	436	YBR083W	TEC1
......P.[ILMVPYAFTR]P.[RKW]...	TRRKKDPNAPKRRLS	18	32	YBR089C-A	NHP6B
...[KR][KR][AHPKRLG]P[PLV].......	RNQRRGPPGERRILT	82	96	YBR090C	YBR090C

Table S6

Motif	Motif Match	Start	End	ORF	Common name
.[FPLWA].[WYLMFHP].[AVLIMFHPR]P..P.....	AAKPFVPLDPYAYSV	587	601	YBR094W	YBR094W
..[KRP]......P.[KR]P..	PAKQKTPPSPAKPKQ	23	37	YBR102C	EXO84
...PPPP...	AASPEPTPAPPVSRN	25	39	YBR105C	VID24
...[KR][KR][AHPKRLG]P[PLV].......	NAAKKLPPQQLPTTP	248	262	YBR105C	VID24
...PPPP...	SLKDPKSFPPPPLKP	79	93	YBR108W	YBR108W
...PPPP...	DPKSFPPPPLKPGQK	82	96	YBR108W	YBR108W
...PPPP...	SFPPPPLKPGQKTYT	85	99	YBR108W	YBR108W
...PPPP...	ILPPRNNVEPPPPPS	547	561	YBR108W	YBR108W
...PPPP...	PRNNVEPPPPPSRGN	550	564	YBR108W	YBR108W
...PPPP...	NVEPPPPPSRGNFER	553	567	YBR108W	YBR108W
...PPPP...	KKAPPPVVKPKPRNF	775	789	YBR108W	YBR108W
...[RK].[AVLIMFHRTP]P..P.....	KDPKSFPPPPLKPGQ	81	95	YBR108W	YBR108W
...[RK].[AVLIMFHRTP]P..P.....	APERAVPILPPRNNV	540	554	YBR108W	YBR108W
...[RK].[AVLIMFHRTP]P..P.....	FIRKRAPTPPAPSRS	722	736	YBR108W	YBR108W
......P.[ILMVPYAFTR]P.[RKW]...	QQQQGQPLPPPRGQV	284	298	YBR108W	YBR108W
......P.[ILMVPYAFTR]P.[RKW]...	PPVGGQPPVPVRMQP	376	390	YBR108W	YBR108W
......P.[ILMVPYAFTR]P.[RKW]...	RNNVEPPPPPSRGNF	551	565	YBR108W	YBR108W
......P.[ILMVPYAFTR]P.[RKW]...	RKRAPTPPAPSRSEK	724	738	YBR108W	YBR108W
...[KR][KR][AHPKRLG]P[PLV].......	QSTKKAPPPVVKPKP	772	786	YBR108W	YBR108W
..[KRP]......P.[KR]P..	EDPISNFLPPPKPFR	581	595	YBR108W	YBR108W
..[KRP]......P.[KR]P..	TQPIQNFQPPPKPFR	651	665	YBR108W	YBR108W
....P.R.A[VP].....	FHLNPKRCAVLYDRP	214	228	YBR110W	ALG1
.[FPLWA].[WYLMFHP].[AVLIMFHPR]P..P.....	CFRYILPQPPAPLQE	209	223	YBR112C	CYC8
......P.[ILMVPYAFTR]P.[RKW]...	DLKNAPPYVPQRSKQ	162	176	YBR114W	RAD16
...[RK].[AVLIMFHRTP]P..P.....	YNPKTVPFVPISGWN	181	195	YBR118W	TEF2
...[RK].[AVLIMFHRTP]P..P.....	ALVKFVPSKPMCVEA	398	412	YBR118W	TEF2
....RP[AS]....Y...	NLPSRPANKENYTRL	8	22	YBR119W	MUD1
...R..[FLIYM].[FLIYM]P.....	DNARSVLSFPPLVAP	531	545	YBR121C	GRS1
......P.[ILMVPYAFTR]P.[RKW]...	STRITLPRRPAKKIQ	19	33	YBR122C	MRPL36
...[RK].[AVLIMFHRTP]P..P.....	IVKKNVPKPPPLVFE	401	415	YBR123C	TFC1
.[FPLWA].[WYLMFHP].[AVLIMFHPR]P..P.....	EFTLDLPRIPSLELP	28	42	YBR123C	TFC1
...[RK].[AVLIMFHRTP]P..P.....	RHLKLVPSTPMDIPW	259	273	YBR128C	ATG14
......P.[ILMVPYAFTR]P.[RKW]...	VQQDSLPKLPFRSWG	487	501	YBR132C	AGP2
...[RK].[AVLIMFHRTP]P..P.....	VQIKKVPTDPSSSQE	26	40	YBR136W	MEC1
...[KR][KR][AHPKRLG]P[PLV].......	EKGKRLPVPEIVPFR	2249	2263	YBR136W	MEC1
......P.[ILMVPYAFTR]P.[RKW]...	QCIGAKPKIPSKLYQ	121	135	YBR138C	YBR138C
...R..[FLIYM].[FLIYM]P.....	FFDRLLLVLPIESNL	23	37	YBR140C	IRA1
...R..[FLIYM].[FLIYM]P.....	VTARAFIEIPLSYIA	1092	1106	YBR140C	IRA1
..[KRP]......P.[KR]P..	EKKVTFDIEPYKPIN	104	118	YBR143C	SUP45
...R..[FLIYM].[FLIYM]P.....	RLLRKYIKLPSQNNN	60	74	YBR146W	MRPS9
...R..[FLIYM].[FLIYM]P.....	MKDRESIMYPLQVIE	190	204	YBR146W	MRPS9
.[FPLWA].[WYLMFHP].[AVLIMFHPR]P..P.....	DPVHLPPANPINETV	110	124	YBR147W	YBR147W
...PPPP...	PAMPPVPSNFPPVPT	46	60	YBR150C	TBS1
.[FPLWA].[WYLMFHP].[AVLIMFHPR]P..P.....	PAVPAMPPVPSNFPP	43	57	YBR150C	TBS1
[FPLWA]..[WYLMFHP].[AVLIMFHPP]P..P.....	LMGPAVPAMPPVPSN	40	54	YBR150C	TBS1
...[RK].[AVLIMFHRTP]P..P.....	SAMKLVPSIPPATIE	119	133	YBR154C	RPB5
...PPPP...	YVPGPGDPELPPQLS	16	30	YBR155W	CNS1
....RP[AVLIMFHRKTP][AVLIMFHRTP][AVLIMFHRTP]..[LP]...	LNVTRPFLFKSLHFK	252	266	YBR158W	AMN1
...R..[FLIYM].[FLIYM]P.....	KIRRSSLMIPNPQQF	262	276	YBR159W	YBR159W
......P.[ILMVPYAFTR]P.[RKW]...	YLPDFKPSFPQWRRK	241	255	YBR160W	CDC28
...[RK].[AVLIMFHRTP]P..P.....	IKTRSVPKIPSIESV	321	335	YBR163W	DEM1
.[FPLWA].[WYLMFHP].[AVLIMFHPR]P..P.....	ELLLSLPTGPESKEL	661	675	YBR172C	SMY2
...PPPP...	LKPPIGRPPKFPKSP	412	426	YBR182C	SMP1
...R..[FLIYM].[FLIYM]P.....	HVRRSILALPLGVLL	224	238	YBR183W	YPC1
...[KR][KR][AHPKRLG]P[PLV].......	RTIRKLPLMCLSEYF	524	538	YBR186W	PCH2
...[RK].[AVLIMFHRTP]P..P.....	SHYRNIPYNPATFKR	91	105	YBR194W	SOY1
...[RK].[AVLIMFHRTP]P..P.....	NRARYLPQNPDIIAG	133	147	YBR195C	MSI1
.[FPLWA].[WYLMFHP].[AVLIMFHPR]P..P.....	FPYLFRPIFPFLIVL	33	47	YBR196C-A	YBR196C-A
...R..[FLIYM].[FLIYM]P.....	SPARDILPLPPKTAL	415	429	YBR198C	TAF5
...[RK].[AVLIMFHRTP]P..P.....	WSKRIMPYIPGPVPY	346	360	YBR200W	BEM1
...R..[FLIYM].[FLIYM]P.....	ECLRKVIILPDCSHN	338	352	YBR204C	YBR204C
......P.[ILMVPYAFTR]P.[RKW]...	IEETPAPNLPEKTRL	869	883	YBR208C	DUR1,2
...[RK].[AVLIMFHRTP]P..P.....	EMVKLTPKNPFYKLP	194	208	YBR214W	SDS24
[FPLWA]..[WYLMFHP].[AVLIMFHPP]P..P.....	ANVPTVPGTPGPIET	147	161	YBR218C	PYC2
[FPLWA]..[WYLMFHP].[AVLIMFHPP]P..P.....	LEDFAFPDTPTIVKA	344	358	YBR221C	PDB1
[FPLWA]..[WYLMFHP].[AVLIMFHPP]P..P.....	AVPFTLPVIPYDLAE	518	532	YBR223C	TDP1
......P.[ILMVPYAFTR]P.[RKW]...	SANIHTPTIPKRSLT	506	520	YBR227C	MCX1
..[KRP]....P...[KR]P..	AIKSIYSPSDKRPFL	581	595	YBR229C	ROT2
...[KR][KR][AHPKRLG]P[PLV].......	RPLKRPPLLEQIISG	215	229	YBR231C	AOR1
..[KRP]....P...[KR]P..	VSRFYQIPGTHRPSS	2	16	YBR235W	YBR235W
..[KRP]....P...[KR]P..	KVKQYIEPESVKPN*	423	437	YBR236C	ABD1
..[KRP]....P...[KR]P..	EFRKSDEPVSVKPSK	77	91	YBR237W	PRP5

Table S6

Motif	Motif Match	Start	End	ORF	Common name
...[RK].[AVLIMFHRTP]P..P.....	NFKRLLPRDPSEKSS	261	275	YBR239C	YBR239C
......P.[ILMVPYAFTR]P.[RKW]...	LPMNIQPDLPPRKIM	87	101	YBR239C	YBR239C
...R..[FLIYM].[FLIYM]P.....	LRWRHKFFLPAIAAI	151	165	YBR243C	ALG7
....RP[AVLIMFHRKTP][AVLIMFHRTP][AVLIMFHRTP]..[LP]...	ITKERPRIVQELKDL	230	244	YBR260C	RGD1
...[RK].[AVLIMFHRTP]P..P.....	MAKKLLPYKPHNSIG	209	223	YBR270C	YBR270C
..[KRP]......P.[KR]P..	FSKMAKKLLPYKPHN	206	220	YBR270C	YBR270C
...R..[FLIYM].[FLIYM]P.....	KCFRDSIILPYYESF	468	482	YBR270C	YBR270C
....P.R.A[VP].....	YLSEPSRYAPSIDVL	451	465	YBR270C	YBR270C
......P.[ILMVPYAFTR]P.[RKW]...	KDKIAHPVRPHRAIT	628	642	YBR276C	PPS1
...R..[FLIYM].[FLIYM]P.....	IHPRPFFLFPSDLQI	508	522	YBR276C	PPS1
......P.[ILMVPYAFTR]P.[RKW]...	QNSLPVPQLPPKLLV	14	28	YBR279W	PAF1
...PPPP...	AMPAETPPPVEPLKT	97	111	YBR284W	YBR284W
..[KRP]......P.[KR]P..	QAPTGLVLEPSKPYK	657	671	YBR284W	YBR284W
..[KRP]....P...[KR]P..	NIPKPHIPYFMKPHV	82	96	YBR286W	APE3
...PPPP...	TSPPPQTHQSPPPPP	70	84	YBR289W	SNF5
...PPPP...	PPQTHQSPPPPPQQS	73	87	YBR289W	SNF5
...PPPP...	THQSPPPPPQQSQPI	76	90	YBR289W	SNF5
...PPPP...	SPPPPPQQSQPIANQ	79	93	YBR289W	SNF5
...PPPP...	SATSTPPPPPAPHNL	94	108	YBR289W	SNF5
...PPPP...	STPPPPPAPHNLHPQ	97	111	YBR289W	SNF5
...PPPP...	SVPTPSLPIAPPVAP	868	882	YBR289W	SNF5
...[RK].[AVLIMFHRTP]P..P.....	TTYKSRPDRPKPVSP	758	772	YBR289W	SNF5
.[FPLWA].[WYLMFHP].[AVLIMFHPR]P..P.....	VPTPSLPIAPPVAPH	869	883	YBR289W	SNF5
....RP[AVLIMFHRKTP][AVLIMFHRTP][AVLIMFHRTP]..[LP]...	SRPDRPKPVSPPCYI	762	776	YBR289W	SNF5
......P.[ILMVPYAFTR]P.[RKW]...	LYDVHGPGIPMKSLE	378	392	YBR297W	MAL33
.....[GP]P.[IVL].P[FWY]...	NAGFTGPDVKPWFFL	454	468	YBR299W	MAL32
....RP[AVLIMFHRKTP][AVLIMFHRTP][AVLIMFHRTP]..[LP]...	SEEFRPFIRRLPEFK	105	119	YCL001W	RER1
...R..[FLIYM].[FLIYM]P.....	LSQRFPLFYPLNDAR	66	80	YCL002C	YCL002C
...PPPP...	IAPIPMPKPNPTHIS	139	153	YCL005W	YCL005W
...PPPP...	QNTPPLPPKPKSPHL	175	189	YCL008C	STP22
...PPPP...	PKSPHLKPPLPPPPP	184	198	YCL008C	STP22
...PPPP...	PHLKPPLPPPPPPQP	187	201	YCL008C	STP22
...PPPP...	KPPLPPPPPPQPASN	190	204	YCL008C	STP22
...PPPP...	LPPPPPPQPASNALD	193	207	YCL008C	STP22
......P.[ILMVPYAFTR]P.[RKW]...	PPQDQAPSLPPKPNT	155	169	YCL008C	STP22
......P.[ILMVPYAFTR]P.[RKW]...	QQEQNTPPLPPKPKS	172	186	YCL008C	STP22
.[FPLWA].[WYLMFHP].[AVLIMFHPR]P..P.....	SPHLKPPLPPPPPPQ	186	200	YCL008C	STP22
.[FPLWA].[WYLMFHP].[AVLIMFHPR]P..P.....	HLKPPLPPPPPPQPA	188	202	YCL008C	STP22
.[FPLWA].[WYLMFHP].[AVLIMFHPR]P..P.....	PLPPPPPPQPASNAL	192	206	YCL008C	STP22
[FPLWA]..[WYLMFHP].[AVLIMFHPP]P..P.....	LKPPLPPPPPPQPAS	189	203	YCL008C	STP22
......P.[ILMVPYAFTR]P.[RKW]...	SGMMALPRTPLKTST	273	287	YCL009C	ILV6
......P.[ILMVPYAFTR]P.[RKW]...	ELLLIPPGFPTKNYP	182	196	YCL010C	SGF29
..[KRP]......P.[KR]P..	VTKETKEILPVKPTK	802	816	YCL014W	BUD3
..[KRP]....P...[KR]P..	NNPSNSIPKIEKPPA	1345	1359	YCL014W	BUD3
[FPLWA]..[WYLMFHP].[AVLIMFHPP]P..P.....	WKSLFPPFFPCDIDI	284	298	YCL016C	DCC1
....RP[AVLIMFHRKTP][AVLIMFHRTP][AVLIMFHRTP]..[LP]...	YVRRRPRVRLEPLLS	310	324	YCL017C	NFS1
......P.[ILMVPYAFTR]P.[RKW]...	MALQHEPPLPIWSLD	177	191	YCL018W	LEU2
...PPPP...	VSPQPASVPPPQNGQ	70	84	YCL019W	YCL019W
.[FPLWA].[WYLMFHP].[AVLIMFHPR]P..P.....	LPKLNVPLNPKGKKL	1508	1522	YCL019W	YCL019W
....RP[AVLIMFHRKTP][AVLIMFHRTP][AVLIMFHRTP]..[LP]...	ELYIRPPPHLGLNDK	1374	1388	YCL019W	YCL019W
...PPPP...	VSPQPASVPPPQNGQ	70	84	YCL020W	YCL020W
...[RK].[AVLIMFHRTP]P..P.....	SRSKPLPLTPNSKYN	416	430	YCL027W	FUS1
...[RK].[AVLIMFHRTP]P..P.....	ASDKGLPMIPAFVTG	57	71	YCL040W	GLK1
[FPLWA]..[WYLMFHP].[AVLIMFHPP]P..P.....	LQSLRLPTTPTERVQ	392	406	YCL040W	GLK1
...PPPP...	KYPLSTEPPTPPSVP	52	66	YCL042W	YCL042W
...PPPP...	LSTEPPTPPSVPNSA	55	69	YCL042W	YCL042W
...R..[FLIYM].[FLIYM]P.....	WRLRRLYKLPPPRRI	73	87	YCL044C	YCL044C
..[KRP]......P.[KR]P..	KARNVEYKKPQKPIP	307	321	YCL061C	MRC1
.[FPLWA].[WYLMFHP].[AVLIMFHPR]P..P.....	ALSPKIPIIPTELIG	213	227	YCL061C	MRC1
.[FPLWA].[WYLMFHP].[AVLIMFHPR]P..P.....	SASFFRPSNPTFGTS	290	304	YCL063W	VAC17
....RP[AVLIMFHRKTP][AVLIMFHRTP][AVLIMFHRTP]..[LP]...	GERSRPLVISILSCA	31	45	YCL069W	YCL069W
....RP[AVLIMFHRKTP][AVLIMFHRTP][AVLIMFHRTP]..[LP]...	NISYRPLLLRRLVAK	195	209	YCL069W	YCL069W
......P.[ILMVPYAFTR]P.[RKW]...	AKFAKSPLLPFKLLS	327	341	YCL073C	YCL073C
....P.R.A[VP].....	PWGSPERDAVVESYR	530	544	YCL073C	YCL073C
...R..[FLIYM].[FLIYM]P.....	NTGRPDISYPVSLLS	195	209	YCL074W	YCL074W
[FPLWA]..[WYLMFHP].[AVLIMFHPP]P..P.....	LTKMNAPQKPEDIPV	47	61	YCR004C	YCP4
....RP[AS]....Y...	GNTTRPAILLSYLVV	824	838	YCR017C	CWH43
...[RK].[AVLIMFHRTP]P..P.....	RWARDTPRAPGLHWK	148	162	YCR020C	PET18
.[FPLWA].[WYLMFHP].[AVLIMFHPR]P..P.....	FFEFFLPFIPHYAFL	8	22	YCR024C-B	YCR024C-B
...PPPP...	DTPPLPPHATPKNVD	415	429	YCR030C	SYP1
...[RK].[AVLIMFHRTP]P..P.....	SKTKPLPVEPASPSI	485	499	YCR030C	SYP1
....RP[AVLIMFHRKTP][AVLIMFHRTP][AVLIMFHRTP]..[LP]...	QVDSRPLHIRAPALP	513	527	YCR030C	SYP1

Table S6

Motif	Motif Match	Start	End	ORF	Common name
...[RK].[AVLIMFHRTP]P..P.....	KIPKHLPADPIFSSV	1500	1514	YCR032W	BPH1
...[RK].[AVLIMFHRTP]P..P.....	EVWRKVPMKPIFEKT	1850	1864	YCR032W	BPH1
..[KRP]......P.[KR]P..	SNKWRSIGTPSRPPF	219	233	YCR033W	SNT1
[FPLWA]..[WYLMFHP].[AVLIMFHPP]P..P.....	LTTLPPPRLPSIQFP	1027	1041	YCR033W	SNT1
...PPPP...	NVPTPSPSPKPQHRR	331	345	YCR034W	FEN1
[FPLWA]..[WYLMFHP].[AVLIMFHPP]P..P.....	AVHLYFPILPHCGDC	256	270	YCR034W	FEN1
......P.[ILMVPYAFTR]P.[RKW]...	YSMVFLPTAPSKHMD	475	489	YCR042C	TAF2
...[RK].[AVLIMFHRTP]P..P.....	TKRKTIPVYPPVQRI	21	35	YCR046C	IMG1
......P.[ILMVPYAFTR]P.[RKW]...	SPDHSSPIAPSKAKR	61	75	YCR065W	HCM1
[FPLWA]..[WYLMFHP].[AVLIMFHPP]P..P.....	LHLPFPPGLPSYMEG	364	378	YCR068W	ATG15
...R..[FLIYM].[FLIYM]P.....	PSKRLHLPFPPGLPS	360	374	YCR068W	ATG15
......P.[ILMVPYAFTR]P.[RKW]...	DLQEQLPFIPKKSWS	106	120	YCR071C	IMG2
......P.[ILMVPYAFTR]P.[RKW]...	VAAGRIPQLPNRDEM	1265	1279	YCR073C	SSK22
...R..[FLIYM].[FLIYM]P.....	ERLRSLLAFPVYLIK	385	399	YCR073C	SSK22
...R..[FLIYM].[FLIYM]P.....	PSTRISLTIPVICHS	234	248	YCR073W-A	SOL2
...PPPP...	TAPPPAMAPSPQSTM	112	126	YCR077C	PAT1
...PPPP...	QPGPSQFAPPPPPPG	187	201	YCR077C	PAT1
...PPPP...	PSQFAPPPPPPGVNV	190	204	YCR077C	PAT1
...PPPP...	FAPPPPPPGVNVNMN	193	207	YCR077C	PAT1
...R..[FLIYM].[FLIYM]P.....	KNPRRQLQIPRQQPS	402	416	YCR077C	PAT1
...PPPP...	PSRSSAAPPPPPRRA	511	525	YCR088W	ABP1
...PPPP...	SSAAPPPPPRRATPE	514	528	YCR088W	ABP1
...PPPP...	APPPPPRRATPEKKP	517	531	YCR088W	ABP1
......P.[ILMVPYAFTR]P.[RKW]...	EDEAAQPPLPSRNVA	443	457	YCR088W	ABP1
......P.[ILMVPYAFTR]P.[RKW]...	EQEEIAPSLPSRNSI	468	482	YCR088W	ABP1
......P.[ILMVPYAFTR]P.[RKW]...	EAEEAAPQLPSRSSA	502	516	YCR088W	ABP1
......P.[ILMVPYAFTR]P.[RKW]...	SRSSAAPPPPPRRAT	512	526	YCR088W	ABP1
......P.[ILMVPYAFTR]P.[RKW]...	RSSAAPPPPPRRATP	513	527	YCR088W	ABP1
.[FPLWA].[WYLMFHP].[AVLIMFHPR]P..P.....	LFTFDMPTDPSSFHT	174	188	YCR091W	KIN82
......P.[ILMVPYAFTR]P.[RKW]...	DDLCREPEVPQRLLT	247	261	YCR095C	YCR095C
...[KR][KR][AHPKRLG]P[PLV].......	NKAKKLPLENNRTHS	217	231	YCR095C	YCR095C
......P.[ILMVPYAFTR]P.[RKW]...	LNLKWEPSTPQRNGP	155	169	YCR102C	YCR102C
...[RK].[AVLIMFHRTP]P..P.....	QKLKLLPEEPFQRDS	75	89	YDL001W	RMD1
......P.[ILMVPYAFTR]P.[RKW]...	KVKERDPNMPKRPTN	85	99	YDL002C	NHP10
...R..[FLIYM].[FLIYM]P.....	TRPRVTLNLPGGERL	163	177	YDL013W	HEX3
....RP[AVLIMFHRKTP][AVLIMFHRTP][AVLIMFHRTP]..[LP]...	ERTTRPRVTLNLPGG	160	174	YDL013W	HEX3
...PPPP...	AAAPKHAPPPVPNET	769	783	YDL019C	OSH2
.[FPLWA].[WYLMFHP].[AVLIMFHPR]P..P.....	APKHAPPPVPNETDN	771	785	YDL019C	OSH2
[FPLWA]..[WYLMFHP].[AVLIMFHPP]P..P.....	PQPHLLPWLPPTDTR	1188	1202	YDL019C	OSH2
......P.[ILMVPYAFTR]P.[RKW]...	IMKVSDPYVPTRNTF	139	153	YDL020C	RPN4
..[KRP]......P.[KR]P..	AKKAPIDYSPSRPLP	108	122	YDL025C	YDL025C
...[RK].[AVLIMFHRTP]P..P.....	DSMKNLPLNPSKKLE	310	324	YDL027C	YDL027C
...[RK].[AVLIMFHRTP]P..P.....	FYHRPAPKPPVTKKV	401	415	YDL028C	MPS1
......P.[ILMVPYAFTR]P.[RKW]...	GGSSMYPGLPSRLEK	304	318	YDL029W	ARP2
......P.[ILMVPYAFTR]P.[RKW]...	HKQMKAPKMPDKHRD	920	934	YDL031W	DBP10
......P.[ILMVPYAFTR]P.[RKW]...	GRRSASPYRPQRLPA	10	24	YDL033C	YDL033C
..[KRP]....P...[KR]P..	LAKYAGLPTAEKPDS	211	225	YDL033C	YDL033C
.[FPLWA].[WYLMFHP].[AVLIMFHPR]P..P.....	FLKHYLPSSPGDIIT	242	256	YDL033C	YDL033C
...R..[FLIYM].[FLIYM]P.....	WYFRSYFKLPLLHLL	311	325	YDL035C	GPR1
...R..[FLIYM].[FLIYM]P.....	KNLRAIFIYPLSYIG	618	632	YDL035C	GPR1
.[FPLWA].[WYLMFHP].[AVLIMFHPR]P..P.....	PPVYRLPPLPRLKVK	5	19	YDL045W-A	MRP10
......P.[ILMVPYAFTR]P.[RKW]...	KEVNLDPNPPVRGEN	58	72	YDL046W	NPC2
...PPPP...	STLNSPPPPPLTTSY	70	84	YDL048C	STP4
...PPPP...	NSPPPPPLTTSYSSY	73	87	YDL048C	STP4
...[RK].[AVLIMFHRTP]P..P.....	HKKKNLPKFPKNKKK	239	253	YDL051W	LHP1
....RP[AVLIMFHRKTP][AVLIMFHRTP][AVLIMFHRTP]..[LP]...	GTRLRPLTLTVPKPL	11	25	YDL055C	PSA1
...R..[FLIYM].[FLIYM]P.....	DRIRMFIRFPKFLLE	555	569	YDL060W	TSR1
...R..[FLIYM].[FLIYM]P.....	TVFREPIVIPRIPRL	122	136	YDL066W	IDP1
....RP[AVLIMFHRKTP][AVLIMFHRTP][AVLIMFHRTP]..[LP]...	VSGGRPKRTIHPPKS	288	302	YDL070W	BDF2
....RP[AVLIMFHRKTP][AVLIMFHRTP][AVLIMFHRTP]..[LP]...	SRYRRPLTLLLLKPF	28	42	YDL072C	YDL072C
.....[GP]P.[IVL].P[FWY]...	YLYYNPPMVGPFIRV	611	625	YDL077C	VAM6
.[FPLWA].[WYLMFHP].[AVLIMFHPR]P..P.....	LFTPLLPSTPVGTIE	133	147	YDL085W	NDE2
...[KR][KR][AHPKRLG]P[PLV].......	LLGRKRPVMERVVDI	19	33	YDL090C	RAM1
...[KR][KR][AHPKRLG]P[PLV].......	VTIKRGPLRSFLITK	15	29	YDL093W	PMT5
...R..[FLIYM].[FLIYM]P.....	FFRRSELYLPNSCKA	198	212	YDL093W	PMT5
....RP[AVLIMFHRKTP][AVLIMFHRTP][AVLIMFHRTP]..[LP]...	QGPVRPFIVTDPSAE	23	37	YDL095W	PMT1
......P.[ILMVPYAFTR]P.[RKW]...	NASDSGPQRPKRTRA	70	84	YDL106C	PHO2
......P.[ILMVPYAFTR]P.[RKW]...	SKFVSTPPVPKKFQE	6	20	YDL107W	MSS2
.[FPLWA].[WYLMFHP].[AVLIMFHPR]P..P.....	ELMLRIPYLPGQNDV	197	211	YDL108W	KIN28
[FPLWA]..[WYLMFHP].[AVLIMFHPP]P..P.....	FKELPPPSDPSSIKI	290	304	YDL108W	KIN28
..[KRP]....P...[KR]P..	IRPDGRLPHQFRPIE	21	35	YDL111C	RRP42
......P.[ILMVPYAFTR]P.[RKW]...	FPMTLIPPIPEKQSI	207	221	YDL113C	ATG20

Table S6

Motif	Motif Match	Start	End	ORF	Common name
...PPPP...	ESMNNPLPPLPPLPD	178	192	YDL117W	CYK3
...PPPP...	NNPLPPLPPLPDLDN	181	195	YDL117W	CYK3
......P.[ILMVPYAFTR]P.[RKW]...	SNKVVIPPVPSRYSD	92	106	YDL117W	CYK3
.[FPLWA].[WYLMFHP].[AVLIMFHPR]P..P.....	NPLPPLPPLPDLDNM	182	196	YDL117W	CYK3
...[RK].[AVLIMFHRTP]P..P.....	AQLRKTPLEPGLELT	669	683	YDL126C	CDC48
..[KRP]....P...[KR]P..	NRPDQIDPAILRPGR	634	648	YDL126C	CDC48
....RP[AVLIMFHRKTP][AVLIMFHRTP][AVLIMFHRTP]..[LP]...	LQQWRPPLTSLLLDN	251	265	YDL133W	YDL133W
...PPPP...	ILTCLPVPPPPVRPS	235	249	YDL140C	RPO21
...PPPP...	CLPVPPPPVRPSISF	238	252	YDL140C	RPO21
......P.[ILMVPYAFTR]P.[RKW]...	LTCLPVPPPPVRPSI	236	250	YDL140C	RPO21
.[FPLWA].[WYLMFHP].[AVLIMFHPR]P..P.....	LPVPPPPVRPSISFN	239	253	YDL140C	RPO21
...[RK].[AVLIMFHRTP]P..P.....	LLRRTIPKRPFYHVL	13	27	YDL142C	CRD1
.[FPLWA].[WYLMFHP].[AVLIMFHPR]P..P.....	TLALSVPSGPQIIPV	141	155	YDL142C	CRD1
...PPPP...	KRKAKAPPPPPPPPP	469	483	YDL146W	YDL146W
...PPPP...	AKAPPPPPPPPPSRK	472	486	YDL146W	YDL146W
...PPPP...	PPPPPPPPPSRKCGT	475	489	YDL146W	YDL146W
...[RK].[AVLIMFHRTP]P..P.....	RKAKAPPPPPPPPPS	470	484	YDL146W	YDL146W
......P.[ILMVPYAFTR]P.[RKW]...	APPPPPPPPPSRKCG	474	488	YDL146W	YDL146W
.[FPLWA].[WYLMFHP].[AVLIMFHPR]P..P.....	KAPPPPPPPPPSRKC	473	487	YDL146W	YDL146W
......P.[ILMVPYAFTR]P.[RKW]...	RIEQLFPVRPVRVRH	195	209	YDL150W	RPC53
..[KRP]......P.[KR]P..	RSKRIEQLFPVRPVR	192	206	YDL150W	RPC53
......P.[ILMVPYAFTR]P.[RKW]...	ATKSASPTLPTRRSR	59	73	YDL156W	YDL156W
......P.[ILMVPYAFTR]P.[RKW]...	KYCTLRPGIPLKPML	381	395	YDL164C	CDC9
.[FPLWA].[WYLMFHP].[AVLIMFHPR]P..P.....	TLRPGIPLKPMLAKP	384	398	YDL164C	CDC9
......P.[ILMVPYAFTR]P.[RKW]...	PAHVIRPGVPTKFTK	1272	1286	YDL171C	GLT1
....RP[AVLIMFHRKTP][AVLIMFHRTP][AVLIMFHRTP]..[LP]...	SPEQRPRIILFPHTT	146	160	YDL174C	DLD1
.[FPLWA].[WYLMFHP].[AVLIMFHPR]P..P.....	ILIPLIPNVPGFYLS	207	221	YDL183C	YDL183C
...R..[FLIYM].[FLIYM]P.....	GRPREVIKLPRGRET	315	329	YDL185W	TFP1
...PPPP...	YNPAGPAPGPAPSPM	319	333	YDL189W	RBS1
...PPPP...	AGPAPGPAPSPMVMG	322	336	YDL189W	RBS1
...PPPP...	MTSPPPVFNKPPTGP	1093	1107	YDL195W	SEC31
...PPPP...	VFNKPPTGPPPISMK	1099	1113	YDL195W	SEC31
...[RK].[AVLIMFHRTP]P..P.....	FKTKFAPEAPDLFAC	304	318	YDL195W	SEC31
...[RK].[AVLIMFHRTP]P..P.....	KTNKNVPVLPTPGMP	767	781	YDL195W	SEC31
...[RK].[AVLIMFHRTP]P..P.....	ELARVTPLTPKEYSK	1180	1194	YDL195W	SEC31
[FPLWA]..[WYLMFHP].[AVLIMFHPP]P..P.....	LNSFAPPPNPYATAT	848	862	YDL195W	SEC31
...[RK].[AVLIMFHRTP]P..P.....	LTLKTLPRVPTDSPQ	462	476	YDL197C	ASF2
......P.[ILMVPYAFTR]P.[RKW]...	RVPTDSPQLPSKDKS	469	483	YDL197C	ASF2
...[RK].[AVLIMFHRTP]P..P.....	NTFKTLPTKPEMQSQ	182	196	YDL202W	MRPL11
......P.[ILMVPYAFTR]P.[RKW]...	RPLNNNPVPPQKINQ	195	209	YDL203C	YDL203C
......P.[ILMVPYAFTR]P.[RKW]...	TLYSHQPYLPIKNKN	326	340	YDL203C	YDL203C
..[KRP]....P...[KR]P..	DVKKEQAPEGKRPNS	31	45	YDL213C	NOP6
......P.[ILMVPYAFTR]P.[RKW]...	YFTQLLPNVPDKVLV	388	402	YDL220C	CDC13
......P.[ILMVPYAFTR]P.[RKW]...	KSEIFGPSFPFRSHI	167	181	YDL224C	WHI4
......P.[ILMVPYAFTR]P.[RKW]...	PKFLNSPDLPERTKL	442	456	YDL225W	SHS1
...[RK].[AVLIMFHRTP]P..P.....	FDLKNIPMMPAGEPV	463	477	YDL229W	SSB1
...[RK].[AVLIMFHRTP]P..P.....	TDIKLTPTDPLVGPV	194	208	YDL230W	PTP1
...[RK].[AVLIMFHRTP]P..P.....	TSLRVLPIAPKIFND	394	408	YDL231C	BRE4
...R..[FLIYM].[FLIYM]P.....	AIYRPIIHFPRDIRK	129	143	YDL231C	BRE4
...PPPP...	MYQGPPQPPPQAVPM	1	15	YDL233W	YDL233W
...PPPP...	GPPQPPPQAVPMPYI	4	18	YDL233W	YDL233W
.[FPLWA].[WYLMFHP].[AVLIMFHPR]P..P.....	EFEFIIPVFPVICST	198	212	YDL233W	YDL233W
...PPPP...	DGLPPPPQQLPANEN	472	486	YDL234C	GYP7
...[KR][KR][AHPKRLG]P[PLV].......	DYWRKLPLFEGLAYG	122	136	YDL237W	YDL237W
..[KRP]......P.[KR]P..	RMKIMMSMEPKKPIT	710	724	YDL240W	LRG1
......P.[ILMVPYAFTR]P.[RKW]...	VINGNTPDAPKRGFL	41	55	YDL245C	HXT15
......P.[ILMVPYAFTR]P.[RKW]...	DRVAIEPGVPSRYSD	88	102	YDL246C	SOR2
[FPLWA]..[WYLMFHP].[AVLIMFHPP]P..P.....	LGIFFAPESPWWLVK	285	299	YDL247W	MPH2
...[KR][KR][AHPKRLG]P[PLV].......	FFSKKKPPIIENKTV	350	364	YDR004W	RAD57
...PPPP...	INNPSPSPPPSSKQP	187	201	YDR006C	SOK1
...PPPP...	PSPSPPPSSKQPPSA	190	204	YDR006C	SOK1
...PPPP...	ATPTPVPFPPINLQG	361	375	YDR006C	SOK1
...[KR][KR][AHPKRLG]P[PLV].......	WTQRRGPLVVYAEDN	199	213	YDR012W	RPL4B
.[FPLWA].[WYLMFHP].[AVLIMFHPR]P..P.....	PAVFSAPIRPDIVHT	23	37	YDR012W	RPL4B
...[KR][KR][AHPKRLG]P[PLV].......	AYGKRGPVLRTPFRS	3	17	YDR014W	RAD61
...[RK].[AVLIMFHRTP]P..P.....	DKFRIPPKSPNIEDK	930	944	YDR017C	KCS1
...[RK].[AVLIMFHRTP]P..P.....	VLRKYIPGEPEFLPF	430	444	YDR023W	SES1
...[RK].[AVLIMFHRTP]P..P.....	SYIKRTPKIPEKDVK	220	234	YDR026C	YDR026C
...R..[FLIYM].[FLIYM]P.....	EAARSMLSIPVSERK	489	503	YDR026C	YDR026C
...[RK].[AVLIMFHRTP]P..P.....	QKKKALPKQPKASDS	872	886	YDR028C	REG1
....RP[AVLIMFHRKTP][AVLIMFHRTP][AVLIMFHRTP]..[LP]...	VASPRPAATPNLSKD	287	301	YDR033W	MRH1
...PPPP...	VSPQPASVPPPQNGQ	70	84	YDR034C-C	YDR034C-C

Table S6

Motif	Motif Match	Start	End	ORF	Common name
...PPPP...	VSPQPASVPPPQNGQ	70	84	YDR034C-D	YDR034C-D
.[FPLWA].[WYLMFHP].[AVLIMFHPR]P..P.....	LPKLNVPLNPKGKKL	1508	1522	YDR034C-D	YDR034C-D
....RP[AVLIMFHRKTP][AVLIMFHRTP][AVLIMFHRTP]..[LP]...	ELYIRPPPHLGLNDK	1374	1388	YDR034C-D	YDR034C-D
...R..[FLIYM].[FLIYM]P.....	NTIREVLLFPTLKPD	565	579	YDR037W	KRS1
[FPLWA]..[WYLMFHP].[AVLIMFHPP]P..P.....	PDSYKPPKNPIVFCH	18	32	YDR058C	TGL2
.[FPLWA].[WYLMFHP].[AVLIMFHPR]P..P.....	KPWYEIPLDPQVGQN	217	231	YDR060W	MAK21
......P.[ILMVPYAFTR]P.[RKW]...	LASNSPPVYPKRIRH	19	33	YDR061W	YDR061W
...R..[FLIYM].[FLIYM]P.....	LFVRSLIKMPQILIL	467	481	YDR061W	YDR061W
....RP[AVLIMFHRKTP][AVLIMFHRTP][AVLIMFHRTP]..[LP]...	HMYIRPLIIEDLKQI	11	25	YDR071C	PAA1
..[KRP]....P...[KR]P..	FKRAGLIPIRLRPDI	45	59	YDR072C	IPT1
...R..[FLIYM].[FLIYM]P.....	LNQRNLISLPFNFML	19	33	YDR072C	IPT1
......P.[ILMVPYAFTR]P.[RKW]...	NIVEIAPLIPKKPAL	594	608	YDR080W	VPS41
...[RK].[AVLIMFHRTP]P..P.....	KPQKIAPKFPGTVRQ	418	432	YDR091C	RLI1
...R..[FLIYM].[FLIYM]P.....	ERARASLDLPGINHA	1587	1601	YDR093W	DNF2
...PPPP...	ASPQPASVPPPQNGP	70	84	YDR098C-A	YDR098C-A
...PPPP...	SVPPPQNGPYPQQCM	76	90	YDR098C-A	YDR098C-A
...PPPP...	ASPQPASVPPPQNGP	70	84	YDR098C-B	YDR098C-B
...PPPP...	SVPPPQNGPYPQQCM	76	90	YDR098C-B	YDR098C-B
.[FPLWA].[WYLMFHP].[AVLIMFHPR]P..P.....	IADLPLPDLPPESPT	1128	1142	YDR098C-B	YDR098C-B
.[FPLWA].[WYLMFHP].[AVLIMFHPR]P..P.....	LPKLNVPLNPKGKKL	1493	1507	YDR098C-B	YDR098C-B
...PPPP...	RFPPYSPLLPPFGLS	271	285	YDR103W	STE5
...[RK].[AVLIMFHRTP]P..P.....	PQSRFPPYSPLLPPF	268	282	YDR103W	STE5
.....[GP]P.[IVL].P[FWY]...	LSDNIPPKVAPFGYP	148	162	YDR103W	STE5
...[RK].[AVLIMFHRTP]P..P.....	QIARQIPYQPWYLRT	516	530	YDR107C	YDR107C
......P.[ILMVPYAFTR]P.[RKW]...	NLQLSTPSAPAKINN	602	616	YDR109C	YDR109C
...[KR][KR][AHPKRLG]P[PLV].......	KRTKRKPVQVLLSEV	360	374	YDR110W	FOB1
....RP[AVLIMFHRKTP][AVLIMFHRTP][AVLIMFHRTP]..[LP]...	AEAKRPATASPLYMP	62	76	YDR116C	MRPL1
...R..[FLIYM].[FLIYM]P.....	SGWRNAFLIPVPFCL	402	416	YDR119W	YDR119W
.[FPLWA].[WYLMFHP].[AVLIMFHPR]P..P.....	SLKYMRPPMPSSAYP	799	813	YDR122W	KIN1
[FPLWA]..[WYLMFHP].[AVLIMFHPP]P..P.....	LVAYGLPVSPDEKWF	328	342	YDR127W	ARO1
......P.[ILMVPYAFTR]P.[RKW]...	IIDLDDPFTPPRWLH	49	63	YDR128W	YDR128W
...R..[FLIYM].[FLIYM]P.....	IFLRISINFPLNYPN	473	487	YDR128W	YDR128W
.[FPLWA].[WYLMFHP].[AVLIMFHPR]P..P.....	PPSHSAPYIPRSSEY	185	199	YDR132C	YDR132C
.[FPLWA].[WYLMFHP].[AVLIMFHPR]P..P.....	VLIFNPPYVPAECVP	118	132	YDR140W	YDR140W
..[KRP]....P...[KR]P..	AIRKENLPNLEKPIV	1608	1622	YDR150W	NUM1
......P.[ILMVPYAFTR]P.[RKW]...	YSPRPSPWLPSKPNC	77	91	YDR151C	CTH1
...[KR][KR][AHPKRLG]P[PLV].......	EPCRRAPLQLPQLVN	187	201	YDR151C	CTH1
.[FPLWA].[WYLMFHP].[AVLIMFHPR]P..P.....	RPSPWLPSKPNCYHP	80	94	YDR151C	CTH1
...R..[FLIYM].[FLIYM]P.....	PCRRAPLQLPQLVNK	188	202	YDR151C	CTH1
....P.R.A[VP].....	TKLEPCRRAPLQLPQ	184	198	YDR151C	CTH1
.[FPLWA].[WYLMFHP].[AVLIMFHPR]P..P.....	TAGLVAPLKPLIEKF	158	172	YDR158W	HOM2
..R[YFLEP]..[AVLIMFHWRTP]P[GSDLIAV]P.....	SLRFKNRPAPSVEQV	261	275	YDR158W	HOM2
...PPPP...	AAPPLPSDVRPPHIL	247	261	YDR159W	SAC3
....RP[AVLIMFHRKTP][AVLIMFHRTP][AVLIMFHRTP]..[LP]...	KVFARPAAAAAPPLP	238	252	YDR159W	SAC3
...[KR][KR][AHPKRLG]P[PLV].......	SVQRKAPPVFEICSK	658	672	YDR160W	SSY1
...R..[FLIYM].[FLIYM]P.....	NFFRKKLGIPRKLKR	175	189	YDR160W	SSY1
...R..[FLIYM].[FLIYM]P.....	YNKRQSITLPDDYIV	97	111	YDR162C	NBP2
......P.[ILMVPYAFTR]P.[RKW]...	QGYPRQPQRPQRYHP	27	41	YDR171W	HSP42
......PR.P.R...	RGQQGYPRQPQRPQR	24	38	YDR171W	HSP42
......P.[ILMVPYAFTR]P.[RKW]...	RKIERDPNAPKKPLT	97	111	YDR174W	HMO1
....RP[AVLIMFHRKTP][AVLIMFHRTP][AVLIMFHRTP]..[LP]...	DNSLRPFMFDELPSQ	126	140	YDR175C	RSM24
...PPPP...	KGVQSPPKPPPSNEI	130	144	YDR176W	NGG1
...PPPP...	QSPPKPPPSNEISGT	133	147	YDR176W	NGG1
......P.[ILMVPYAFTR]P.[RKW]...	LTIPFLPVLPQKPGG	32	46	YDR178W	SDH4
.[FPLWA].[WYLMFHP].[AVLIMFHPR]P..P.....	PFLPVLPQKPGGVRG	35	49	YDR178W	SDH4
...R..[FLIYM].[FLIYM]P.....	PLRRTVMSLPGNHDI	172	186	YDR182W	CDC1
......P.[ILMVPYAFTR]P.[RKW]...	DHGGRHPDMPTRVKN	216	230	YDR188W	CCT6
.[FPLWA].[WYLMFHP].[AVLIMFHPR]P..P.....	ELGPKVPFCPLVGSE	95	109	YDR190C	RVB1
...PPPP...	ELGLVPDIPPPPALV	415	429	YDR192C	NUP42
.[FPLWA].[WYLMFHP].[AVLIMFHPR]P..P.....	GLVPDIPPPPALVA*	417	431	YDR192C	NUP42
...[RK].[AVLIMFHRTP]P..P.....	DLNKRPPREPIEFDL	472	486	YDR195W	REF2
...R..[FLIYM].[FLIYM]P.....	RRLRDKYKLPIVDAD	19	33	YDR196C	YDR196C
...[KR][KR][AHPKRLG]P[PLV].......	LEKRRRPPPQLQHSP	4	18	YDR200C	VPS64
...[KR][KR][AHPKRLG]P[PLV].......	EKRRRPPPQLQHSPY	5	19	YDR200C	VPS64
..[KRP]......P.[KR]P..	MTKTPETSPPKRPMG	29	43	YDR200C	VPS64
...R..[FLIYM].[FLIYM]P.....	KCQRRGLILPAAAMY	16	30	YDR204W	COQ4
......P.[ILMVPYAFTR]P.[RKW]...	LQESKGPRTPAKEIL	307	321	YDR206W	EBS1
...PPPP...	HTNEQYIPPPPPKYI	478	492	YDR207C	UME6
...PPPP...	EQYIPPPPPKYINSK	481	495	YDR207C	UME6
......P.[ILMVPYAFTR]P.[RKW]...	TNEQYIPPPPPKYIN	479	493	YDR207C	UME6
....RP[AS]....Y...	ATSSRPSTGFFYGDL	453	467	YDR207C	UME6
...PPPP...	ASPQPASVPPPQNGP	70	84	YDR210C-C	YDR210C-C

Table S6

Motif	Motif Match	Start	End	ORF	Common name
...PPPP...	SVPPPQNGPYPQQCM	76	90	YDR210C-C	YDR210C-C
...PPPP...	ASPQPASVPPPQNGP	70	84	YDR210C-D	YDR210C-D
...PPPP...	SVPPPQNGPYPQQCM	76	90	YDR210C-D	YDR210C-D
.[FPLWA].[WYLMFHP].[AVLIMFHPR]P..P.....	IADLPLPDLPPESPT	1128	1142	YDR210C-D	YDR210C-D
.[FPLWA].[WYLMFHP].[AVLIMFHPR]P..P.....	LPKLNVPLNPKGKKL	1493	1507	YDR210C-D	YDR210C-D
...PPPP...	VSPQPASVPPPQNGQ	70	84	YDR210W-A	YDR210W-A
...PPPP...	VSPQPASVPPPQNGQ	70	84	YDR210W-B	YDR210W-B
.[FPLWA].[WYLMFHP].[AVLIMFHPR]P..P.....	LPKLNVPLNPKGKKL	1508	1522	YDR210W-B	YDR210W-B
....RP[AVLIMFHRKTP][AVLIMFHRTP][AVLIMFHRTP]..[LP]...	ELYIRPPPHLGLNDK	1374	1388	YDR210W-B	YDR210W-B
...[RK].[AVLIMFHRTP]P..P.....	DLVKKPPFIPSPTFS	209	223	YDR219C	YDR219C
..[KRP]....P...[KR]P..	VRRKPHSPNKKKPKE	11	25	YDR223W	YDR223W
..[KRP]....P...[KR]P..	KKPASKAPAEKKPAA	7	21	YDR224C	HTB1
...[RK].[AVLIMFHRTP]P..P.....	QLMKNLPKIPLLNDI	356	370	YDR228C	PCF11
....P.R.A[VP].....	FDVSPDRYAPHLSEF	29	43	YDR229W	IVY1
[FPLWA]..[WYLMFHP].[AVLIMFHPP]P..P.....	PMLHNVPHDPSLPGQ	159	173	YDR231C	COX20
[FPLWA]..[WYLMFHP].[AVLIMFHPP]P..P.....	FHELGIPVIPNPSHI	405	419	YDR232W	HEM1
...R..[FLIYM].[FLIYM]P.....	NVLRKILEIPLHSFS	166	180	YDR235W	PRP42
...PPPP...	DLPIPAQPGPPFPLV	37	51	YDR236C	FMN1
..R[YFLEP]..[AVLIMFHWRTP]P[GSDLIAV]P.....	FKREVDLPIPAQPGP	32	46	YDR236C	FMN1
...PPPP...	KIKRSRPPPPPMDMK	145	159	YDR239C	YDR239C
...PPPP...	RSRPPPPPMDMKSIT	148	162	YDR239C	YDR239C
......P.[ILMVPYAFTR]P.[RKW]...	TAETSAPDIPPRSPN	51	65	YDR239C	YDR239C
.....P.R.P.R...	SAPDIPPRSPNRNAH	55	69	YDR239C	YDR239C
....P.R.A[VP].....	YQTQPQRHAVNKPSN	331	345	YDR240C	SNU56
....RP[AVLIMFHRKTP][AVLIMFHRTP][AVLIMFHRTP]..[LP]...	YDSNRPAILPPLYGI	113	127	YDR242W	AMD2
...[RK].[AVLIMFHRTP]P..P.....	KMSRSPPRPPSLKII	238	252	YDR243C	PRP28
[FPLWA]..[WYLMFHP].[AVLIMFHPP]P..P.....	ASFFLLPSCPGNTIQ	9	23	YDR246W-A	YDR246W-A
..[KRP]......P.[KR]P..	QMRMDPNQQPEKPAL	84	98	YDR258C	HSP78
....RP[AVLIMFHRKTP][AVLIMFHRTP][AVLIMFHRTP]..[LP]...	SFNARPRVANKLLSD	39	53	YDR258C	HSP78
...PPPP...	ASPQPASVPPPQNGP	70	84	YDR261C-C	YDR261C-C
...PPPP...	SVPPPQNGPYPQQCM	76	90	YDR261C-C	YDR261C-C
...PPPP...	ASPQPASVPPPQNGP	70	84	YDR261C-D	YDR261C-D
...PPPP...	SVPPPQNGPYPQQCM	76	90	YDR261C-D	YDR261C-D
.[FPLWA].[WYLMFHP].[AVLIMFHPR]P..P.....	IADLPLPDLPPESPT	1128	1142	YDR261C-D	YDR261C-D
.[FPLWA].[WYLMFHP].[AVLIMFHPR]P..P.....	IPKLNVPLNPKGRKL	1493	1507	YDR261C-D	YDR261C-D
...PPPP...	VSPQPASVPPPQNGQ	70	84	YDR261W-A	YDR261W-A
...PPPP...	VSPQPASVPPPQNGQ	70	84	YDR261W-B	YDR261W-B
.[FPLWA].[WYLMFHP].[AVLIMFHPR]P..P.....	LPKLNVPLNPKGKKL	1508	1522	YDR261W-B	YDR261W-B
....RP[AVLIMFHRKTP][AVLIMFHRTP][AVLIMFHRTP]..[LP]...	ELYIRPPPHLGLNDK	1374	1388	YDR261W-B	YDR261W-B
...[RK].[AVLIMFHRTP]P..P.....	SWCKERPECPLCRQH	312	326	YDR265W	PEX10
...PPPP...	LNLPQLPPPKPKVQI	571	585	YDR266C	YDR266C
..[KRP]....P...[KR]P..	PKPKVQIPGLNRPQI	579	593	YDR266C	YDR266C
[FPLWA]..[WYLMFHP].[AVLIMFHPP]P..P.....	LKSLNLPQLPPPKPK	568	582	YDR266C	YDR266C
...R..[FLIYM].[FLIYM]P.....	IRERDDYDLPSISSL	333	347	YDR266C	YDR266C
...PPPP...	KRPDMPPPSIIPRKN	343	357	YDR277C	MTH1
...[RK].[AVLIMFHRTP]P..P.....	APPRIFPKAPIYGTQ	1061	1075	YDR283C	GCN2
......P.[ILMVPYAFTR]P.[RKW]...	QLLTESPLMPLWRKM	187	201	YDR284C	DPP1
..[KRP]......P.[KR]P..	IFPPIDDPLPFKPLM	246	260	YDR284C	DPP1
[FPLWA]..[WYLMFHP].[AVLIMFHPP]P..P.....	LVVWNPPILPQHERK	496	510	YDR291W	YDR291W
....RP[AS]....Y...	TKRKRPARLIFYDSK	935	949	YDR291W	YDR291W
....P.R.A[VP].....	KVYGPVRLAVRTYEH	77	91	YDR294C	DPL1
...[RK].[AVLIMFHRTP]P..P.....	HVLKYAPDEPNSLSG	1195	1209	YDR301W	CFT1
...[RK].[AVLIMFHRTP]P..P.....	FYRRDIPDVPITTGA	828	842	YDR310C	SUM1
......P.[ILMVPYAFTR]P.[RKW]...	SSSRRGPGRPRKDAS	201	215	YDR310C	SUM1
...PPPP...	ASPQPASVPPPQNGP	70	84	YDR316W-A	YDR316W-A
...PPPP...	SVPPPQNGPYPQQCM	76	90	YDR316W-A	YDR316W-A
...PPPP...	ASPQPASVPPPQNGP	70	84	YDR316W-B	YDR316W-B
...PPPP...	SVPPPQNGPYPQQCM	76	90	YDR316W-B	YDR316W-B
.[FPLWA].[WYLMFHP].[AVLIMFHPR]P..P.....	IADLPLPDLPPESPT	1128	1142	YDR316W-B	YDR316W-B
.[FPLWA].[WYLMFHP].[AVLIMFHPR]P..P.....	IPKLNVPLNPKGRKL	1493	1507	YDR316W-B	YDR316W-B
......P.[ILMVPYAFTR]P.[RKW]...	IIADYLPPVPEKNAG	271	285	YDR322W	MRPL35
...R..[FLIYM].[FLIYM]P.....	AAVREKYGLPPGRVF	348	362	YDR322W	MRPL35
...[RK].[AVLIMFHRTP]P..P.....	IEARVHPEPPNKPGI	404	418	YDR330W	YDR330W
......P.[ILMVPYAFTR]P.[RKW]...	FASSLAPVYPLKSLL	662	676	YDR332W	YDR332W
...[RK].[AVLIMFHRTP]P..P.....	KDHRLAPNGPGMHPQ	254	268	YDR333C	YDR333C
...[RK].[AVLIMFHRTP]P..P.....	WRPRIVPILPYITRL	780	794	YDR335W	MSN5
...R..[FLIYM].[FLIYM]P.....	GCIRWKIWYPTGDDY	517	531	YDR335W	MSN5
...R..[FLIYM].[FLIYM]P.....	ELNRMALLMPESLKK	619	633	YDR335W	MSN5
....RP[AVLIMFHRKTP][AVLIMFHRTP][AVLIMFHRTP]..[LP]...	QDLWRPRIVPILPYI	777	791	YDR335W	MSN5
...[RK].[AVLIMFHRTP]P..P.....	QRIRKIPGIPLMSVG	162	176	YDR339C	YDR339C
...R..[FLIYM].[FLIYM]P.....	TMERGDLLIPIPRLR	63	77	YDR341C	YDR341C
...[RK].[AVLIMFHRTP]P..P.....	KVNKVAPDHPFIQQE	275	289	YDR345C	HXT3

Table S6

Motif	Motif Match	Start	End	ORF	Common name
...R..[FLIYM].[FLIYM]P.....	SKIRKFLGIPTVFNV	194	208	YDR354W	TRP4
...[RK].[AVLIMFHRTP]P..P.....	DLVKRFPEQPPLRYS	106	120	YDR358W	GGA1
..[KRP]......P.[KR]P..	NTPRPNPTQPRKPLD	773	787	YDR359C	VID21
....RP[AVLIMFHRKTP][AVLIMFHRTP][AVLIMFHRTP]..[LP]...	PISPRPPVPNALAHY	203	217	YDR359C	VID21
....RP[AVLIMFHRKTP][AVLIMFHRTP][AVLIMFHRTP]..[LP]...	NHYLRPPAVPSLRYL	628	642	YDR359C	VID21
...PPPP...	ASPQPASVPPPQNGP	70	84	YDR365W-A	YDR365W-A
...PPPP...	SVPPPQNGPYPQQCM	76	90	YDR365W-A	YDR365W-A
...PPPP...	ASPQPASVPPPQNGP	70	84	YDR365W-B	YDR365W-B
...PPPP...	SVPPPQNGPYPQQCM	76	90	YDR365W-B	YDR365W-B
.[FPLWA].[WYLMFHP].[AVLIMFHPR]P..P.....	IADLPLPDLPPESPT	1128	1142	YDR365W-B	YDR365W-B
.[FPLWA].[WYLMFHP].[AVLIMFHPR]P..P.....	IPKLNVPLNPKGRKL	1493	1507	YDR365W-B	YDR365W-B
....RP[AVLIMFHRKTP][AVLIMFHRTP][AVLIMFHRTP]..[LP]...	YRKRRPKLICIPYTS	56	70	YDR370C	YDR370C
...R..[FLIYM].[FLIYM]P.....	SKLRDNINIPTLTLM	56	70	YDR372C	VPS74
...[KR][KR][AHPKRLG]P[PLV].......	NLLRRAPVKDVKLIA	208	222	YDR376W	ARH1
...[KR][KR][AHPKRLG]P[PLV].......	KNYKKAPPPSSGYDK	290	304	YDR376W	ARH1
...[RK].[AVLIMFHRTP]P..P.....	HFYKSLPQGPAPAIK	35	49	YDR377W	ATP17
..[KRP]....P...[KR]P..	TPPLESGPLFKRPSL	561	575	YDR379W	RGA2
..[KRP]....P...[KR]P..	MAKSQNKPKREKPAK	195	209	YDR381W	YRA1
...[KR][KR][AHPKRLG]P[PLV].......	TTLKRPPLSSRPYSY	57	71	YDR389W	SAC7
..[KRP]....P...[KR]P..	TSKMLKPPKQRRPHS	475	489	YDR389W	SAC7
..[KRP]....P...[KR]P..	KRRTSLFPWLHKPGI	506	520	YDR389W	SAC7
...[RK].[AVLIMFHRTP]P..P.....	CTIRSTPSQPIHCIV	177	191	YDR390C	UBA2
......P.[ILMVPYAFTR]P.[RKW]...	FDGYMQPIIPGKTEC	148	162	YDR390C	UBA2
...[KR][KR][AHPKRLG]P[PLV].......	NGSKKRPVDTEISEA	601	615	YDR390C	UBA2
...R..[FLIYM].[FLIYM]P.....	VLIREKYSYPQDISL	463	477	YDR390C	UBA2
[FPLWA]..[WYLMFHP].[AVLIMFHPP]P..P.....	LTLLKMPEVPNSVSP	824	838	YDR395W	SXM1
...[RK].[AVLIMFHRTP]P..P.....	NFPKNIPTIPFYVVF	28	42	YDR402C	DIT2
...[RK].[AVLIMFHRTP]P..P.....	WEEKLTPAGPLCPLN	464	478	YDR402C	DIT2
......P.[ILMVPYAFTR]P.[RKW]...	RIECILPAFPCKSSN	213	227	YDR403W	DIT1
...[RK].[AVLIMFHRTP]P..P.....	QAKRAPPSSPYVVNY	497	511	YDR406W	PDR15
...[KR][KR][AHPKRLG]P[PLV].......	KQAKRAPPSSPYVVN	496	510	YDR406W	PDR15
....RP[AVLIMFHRKTP][AVLIMFHRTP][AVLIMFHRTP]..[LP]...	ELAARPKLLVFLDEP	1038	1052	YDR406W	PDR15
...[RK].[AVLIMFHRTP]P..P.....	LKIKILPEQPQLELL	839	853	YDR407C	TRS120
..[KRP]....P...[KR]P..	CVRIIKLPTVIKPNE	928	942	YDR407C	TRS120
......P.[ILMVPYAFTR]P.[RKW]...	SRLIVNPFIPRRPYS	856	870	YDR409W	SIZ1
...[RK].[AVLIMFHRTP]P..P.....	VYEKTIPLFPPEIQY	607	621	YDR416W	SYF1
......P.[ILMVPYAFTR]P.[RKW]...	DINSHLPLIPEKIKS	200	214	YDR419W	RAD30
...R..[FLIYM].[FLIYM]P.....	KLSRGRYGLPLSSRP	376	390	YDR419W	RAD30
......P.[ILMVPYAFTR]P.[RKW]...	STLYSNPQTPLRSLA	1401	1415	YDR420W	HKR1
......P.[ILMVPYAFTR]P.[RKW]...	LAQNHIPPFPLRFSF	529	543	YDR421W	ARO80
..[KRP]......P.[KR]P..	VEPTLDEELPKRPEL	628	642	YDR422C	SIP1
.[FPLWA].[WYLMFHP].[AVLIMFHPR]P..P.....	NLQPLHPLHPIINDN	452	466	YDR422C	SIP1
[FPLWA]..[WYLMFHP].[AVLIMFHPP]P..P.....	LSRYPVPDLPIYLNS	771	785	YDR422C	SIP1
...PPPP...	LAPPLSPTKPSPLHS	253	267	YDR425W	SNX41
......P.[ILMVPYAFTR]P.[RKW]...	LPTIIIPPIPSKHSL	168	182	YDR425W	SNX41
...R..[FLIYM].[FLIYM]P.....	NSLREELLFPFAPIP	205	219	YDR429C	TIF35
...PPPP...	QPPEPQPYYPPPPPG	91	105	YDR432W	NPL3
...PPPP...	EPQPYYPPPPPGEHM	94	108	YDR432W	NPL3
...PPPP...	PYYPPPPPGEHMHGR	97	111	YDR432W	NPL3
[FPLWA]..[WYLMFHP].[AVLIMFHPP]P..P.....	PQAPDAPQEPQVPQE	29	43	YDR432W	NPL3
[FPLWA]..[WYLMFHP].[AVLIMFHPP]P..P.....	PQPYYPPPPPGEHMH	95	109	YDR432W	NPL3
...[KR][KR][AHPKRLG]P[PLV].......	EMKRKKPVRPVDIDE	386	400	YDR436W	PPZ2
...[RK].[AVLIMFHRTP]P..P.....	DLGKDIPFFPSSEKP	542	556	YDR443C	SSN2
...R..[FLIYM].[FLIYM]P.....	NGKRKYLDIPIDEMT	569	583	YDR443C	SSN2
......P.[ILMVPYAFTR]P.[RKW]...	TLWDLPPKFPEKPVH	183	197	YDR444W	YDR444W
......P.[ILMVPYAFTR]P.[RKW]...	YILEILPQAPAKKVF	376	390	YDR444W	YDR444W
.[FPLWA].[WYLMFHP].[AVLIMFHPR]P..P.....	DLPPKFPEKPVHLVI	186	200	YDR444W	YDR444W
...[RK].[AVLIMFHRTP]P..P.....	ESFRERPLEPPRKPM	139	153	YDR448W	ADA2
..[KRP]......P.[KR]P..	SFRERPLEPPRKPMA	140	154	YDR448W	ADA2
..[KRP]....P...[KR]P..	NDRNNADPEKKKPAR	2434	2448	YDR457W	TOM1
..[KRP]....P...[KR]P..	KARHVLYPKGVKPQD	220	234	YDR463W	STP1
...[RK].[AVLIMFHRTP]P..P.....	IVERGPPYPPDLRLT	650	664	YDR464W	SPP41
...[KR][KR][AHPKRLG]P[PLV].......	FKPKKRPPQEKKKTK	366	380	YDR464W	SPP41
..[KRP]......P.[KR]P..	DLRLTKSGKPKKPYR	660	674	YDR464W	SPP41
.[FPLWA].[WYLMFHP].[AVLIMFHPR]P..P.....	HPPWTIPSQPPFALP	793	807	YDR464W	SPP41
.[FPLWA].[WYLMFHP].[AVLIMFHPR]P..P.....	HALLTFPERPISQSY	5	19	YDR465C	RMT2
...PPPP...	RQPTPSPPLPQMEFP	829	843	YDR466W	YDR466W
...[RK].[AVLIMFHRTP]P..P.....	APNRIPPKVPVINDN	430	444	YDR466W	YDR466W
..R[YFLEP]..[AVLIMFHWRTP]P[GSDLIAV]P.....	SIRYVAHPLPEKINE	213	227	YDR473C	PRP3
...R..[FLIYM].[FLIYM]P.....	EQKRDFFNYPTILFP	209	223	YDR475C	JIP4
..[KRP]....P...[KR]P..	DLPEYLLPPDLKPHP	309	323	YDR477W	SNF1
...R..[FLIYM].[FLIYM]P.....	SVKRYQIMLPEQLKS	43	57	YDR478W	SNM1

Table S6

Motif	Motif Match	Start	End	ORF	Common name
…[RK].[AVLIMFHRTP]P..P…..	KFIKLIPMRPVLILL	287	301	YDR479C	PEX29
..[KRP]….P…[KR]P..	QGPPLRRPEVPKPVP	210	224	YDR479C	PEX29
.[FPLWA].[WYLMFHP].[AVLIMFHPR]P..P…..	GPPLRRPEVPKPVPE	211	225	YDR479C	PEX29
….RP[AVLIMFHRKTP][AVLIMFHRTP][AVLIMFHRTP]..[LP]…	PQQLRPVPLSQLLSL	349	363	YDR488C	PAC11
…[RK].[AVLIMFHRTP]P..P…..	GTAKILPEEPSNTAD	269	283	YDR490C	PKH1
…..[GP]P.[IVL].P[FWY]…	VWDDNPPEIQPYKIN	401	415	YDR490C	PKH1
…R..[FLIYM].[FLIYM]P…..	VLWRRIFKLPVKLGL	290	304	YDR498C	SEC20
……P.[ILMVPYAFTR]P.[RKW]…	SFVINQPSTPKKLKR	313	327	YDR501W	PLM2
.[FPLWA].[WYLMFHP].[AVLIMFHPR]P..P…..	GAHLDLPKNPNDVSI	347	361	YDR506C	YDR506C
…[RK].[AVLIMFHRTP]P..P…..	SNTKWTPITPSVIIS	75	89	YDR515W	SLF1
…[RK].[AVLIMFHRTP]P..P…..	TVDKGLPMIPTYVTS	57	71	YDR516C	EMI2
……P.[ILMVPYAFTR]P.[RKW]…	PSAFTAPPVPTKKKS	313	327	YDR517W	GRH1
.[FPLWA].[WYLMFHP].[AVLIMFHPR]P..P…..	NLKYGLPQLPEEEYA	321	335	YDR518W	EUG1
…[KR][KR][AHPKRLG]P[PLV]…….	NLPKRKPPKLQGPFS	228	242	YDR523C	SPS1
…[RK].[AVLIMFHRTP]P..P…..	LHEKYFPDLPKEVDK	253	267	YDR527W	YDR527W
.[FPLWA].[WYLMFHP].[AVLIMFHPR]P..P…..	GAKYLAPVGPWDDYS	195	209	YDR533C	HSP31
……P.[ILMVPYAFTR]P.[RKW]…	TRASTSPPRPKRIVV	45	59	YDR538W	PAD1
…[RK].[AVLIMFHRTP]P..P…..	RVTRKRPREPKSTND	757	771	YDR545W	YRF1-1
……P.[ILMVPYAFTR]P.[RKW]…	VCKLRSPNTPRRLRK	309	323	YDR545W	YRF1-1
..[KRP]……P.[KR]P..	TERLKRDLCPRKPIE	229	243	YDR545W	YRF1-1
…[RK].[AVLIMFHRTP]P..P…..	GKLKHIPRRPYEIER	27	41	YEL007W	YEL007W
…[RK].[AVLIMFHRTP]P..P…..	RKQRSIPLSPIVPES	210	224	YEL009C	GCN4
…R..[FLIYM].[FLIYM]P…..	FDYRLAMALPDMWIK	436	450	YEL011W	GLC3
.[FPLWA].[WYLMFHP].[AVLIMFHPR]P..P…..	TAPYMIPSFPTQTFP	115	129	YEL016C	YEL016C
…[RK].[AVLIMFHRTP]P..P…..	PIPKSVPLHPKSGKY	7	21	YEL019C	MMS21
.[FPLWA].[WYLMFHP].[AVLIMFHPR]P..P…..	NPSPLIPDNPGRLLS	308	322	YEL023C	YEL023C
…R..[FLIYM].[FLIYM]P…..	FPIRTSIVYPSEYPE	952	966	YEL025C	YEL025C
…PPPP…	NQPQPPVPEPIAHLF	868	882	YEL031W	SPF1
[FPLWA]..[WYLMFHP].[AVLIMFHPP]P..P…..	AKPYVLPFFPLYATF	25	39	YEL031W	SPF1
[FPLWA]..[WYLMFHP].[AVLIMFHPP]P..P…..	PIAHLFPPGPKNPHY	877	891	YEL031W	SPF1
..[KRP]….P…[KR]P..	HDKDVIVPNVWRPLP	221	235	YEL036C	ANP1
..R[YFLEP]..[AVLIMFHWRTP]P[GSDLIAV]P…..	ASRPGNAPVPDGQKY	218	232	YEL038W	UTR4
……P.[ILMVPYAFTR]P.[RKW]…	TQKYQYPQTPSKVDI	240	254	YEL040W	UTR2
……P.[ILMVPYAFTR]P.[RKW]…	THQLSSPCLPPKVNA	322	336	YEL042W	GDA1
……P.[ILMVPYAFTR]P.[RKW]…	LDVPVGPKVPAKEPS	871	885	YEL043W	YEL043W
…R..[FLIYM].[FLIYM]P…..	VGERNEIGFPSRFKS	36	50	YEL044W	IES6
…[RK].[AVLIMFHRTP]P..P…..	TRARYMPQDPNIVAT	122	136	YEL056W	HAT2
……P.[ILMVPYAFTR]P.[RKW]…	QSGNLKPERPSKVSF	536	550	YEL062W	NPR2
.[FPLWA].[WYLMFHP].[AVLIMFHPR]P..P…..	LLALFIPALPFIINT	194	208	YEL065W	SIT1
.[FPLWA].[WYLMFHP].[AVLIMFHPR]P..P…..	TAYMYAPDDPRAVIE	111	125	YEL070W	YEL070W
……P.[ILMVPYAFTR]P.[RKW]…	VCKLRSPNTPRRLRK	93	107	YEL076C	YEL076C
..[KRP]……P.[KR]P..	TERLKRDLCPRKPIE	13	27	YEL076C	YEL076C
……P.[ILMVPYAFTR]P.[RKW]…	VCKLRSPNTPRRLRK	93	107	YEL076C-A	YEL076C-A
..[KRP]……P.[KR]P..	TERLKRDLCPRKPIE	13	27	YEL076C-A	YEL076C-A
…[RK].[AVLIMFHRTP]P..P…..	RVTRKRPREPKSTND	182	196	YEL077C	YEL077C
[FPLWA]..[WYLMFHP].[AVLIMFHPP]P..P…..	FVFFIAPHLPVDLEA	400	414	YER003C	PMI40
…R..[FLIYM].[FLIYM]P…..	AILRRELPFPATDKV	31	45	YER004W	FMP52
……P.[ILMVPYAFTR]P.[RKW]…	YLKSSFPTIPLRASS	229	243	YER006W	NUG1
…..[GP]P.[IVL].P[FWY]…	SLQKIGPNIRPYEDK	666	680	YER008C	SEC3
……P.[ILMVPYAFTR]P.[RKW]…	LNEHGVPVVPSREVT	437	451	YER014W	HEM14
…[KR][KR][AHPKRLG]P[PLV]…….	NTKRKRPLEANDFVV	152	166	YER015W	FAA2
.[FPLWA].[WYLMFHP].[AVLIMFHPR]P..P…..	PFTFLFPFLPTIILL	224	238	YER017C	AFG3
…[RK].[AVLIMFHRTP]P..P…..	FIVKTIPVPPRDFMI	297	311	YER025W	GCD11
.[FPLWA].[WYLMFHP].[AVLIMFHPR]P..P…..	MAWLTPPQLPPHLEN	342	356	YER027C	GAL83
…[RK].[AVLIMFHRTP]P..P…..	SFDRPPPLTPEKNLY	206	220	YER032W	FIR1
…[RK].[AVLIMFHRTP]P..P…..	KKSRVLPPLPFPLYD	836	850	YER032W	FIR1
..[KRP]……P.[KR]P..	ETKSVPSIEPFKPLS	605	619	YER032W	FIR1
…..P.R.P.R…	TSIEIPKRSPLRFTS	520	534	YER032W	FIR1
…PPPP…	KNTGPPPPLPPPLFP	115	129	YER033C	ZRG8
…PPPP…	GPPPPLPPPLFPSSS	118	132	YER033C	ZRG8
…PPPP…	AVLNSPPLPPPARSQ	826	840	YER033C	ZRG8
…PPPP…	NSPPLPPPARSQSLK	829	843	YER033C	ZRG8
.[FPLWA].[WYLMFHP].[AVLIMFHPR]P..P…..	PPPLPPPLFPSSSTS	120	134	YER033C	ZRG8
..[KRP]….P…[KR]P..	VPPTQSLPNGKKPNF	35	49	YER035W	EDC2
……P.[ILMVPYAFTR]P.[RKW]…	ADGLVQPVVPDKVFS	365	379	YER036C	YER036C
…PPPP…	VDPPKLPDPDPARKV	40	54	YER037W	PHM8
…[RK].[AVLIMFHRTP]P..P…..	YGSKFIPFGPKCQVT	416	430	YER038C	KRE29
…R..[FLIYM].[FLIYM]P…..	SFQRQGMNIPRRKMS	562	576	YER040W	GLN3
…PPPP…	TTAPALPSLPPPPLL	334	348	YER047C	SAP1
…PPPP…	PALPSLPPPPLLNVD	337	351	YER047C	SAP1
…[RK].[AVLIMFHRTP]P..P…..	TKIRRRPQQPLTDFT	14	28	YER047C	SAP1
…R..[FLIYM].[FLIYM]P…..	FVRRQYIPLPEDQTR	782	796	YER047C	SAP1

Table S6

Motif	Motif Match	Start	End	ORF	Common name
...[RK].[AVLIMFHRTP]P..P.....	RRSKSLPTTPGIRSG	190	204	YER054C	GIP2
...R..[FLIYM].[FLIYM]P.....	LTDRLLFAIPKKGRL	7	21	YER055C	HIS1
....P.R.A[VP].....	LKVTPGRRAPTISKI	241	255	YER055C	HIS1
...R..[FLIYM].[FLIYM]P.....	LKGRNGLGYPINEQD	154	168	YER062C	HOR2
...[RK].[AVLIMFHRTP]P..P.....	PNGRTLPPVPTQVRS	179	193	YER064C	YER064C
...R..[FLIYM].[FLIYM]P.....	TILRSEFEYPDTFSA	75	89	YER064C	YER064C
...PPPP...	IQPPLNVSTPPPGIF	535	549	YER068W	MOT2
...R..[FLIYM].[FLIYM]P.....	APLRVPITLPNGFTY	26	40	YER073W	ALD5
[FPLWA]..[WYLMFHP].[AVLIMFHPP]P..P.....	FVTLHVPATPETEKM	253	267	YER081W	SER3
..[KRP]......P.[KR]P..	NERIISFEFPERPGA	493	507	YER086W	ILV1
......P.[ILMVPYAFTR]P.[RKW]...	NSKSNFPDFPLKKYL	330	344	YER087W	YER087W
...R..[FLIYM].[FLIYM]P.....	RSRRSSFAYPQQVAI	277	291	YER088C	DOT6
......P.[ILMVPYAFTR]P.[RKW]...	LFDIHSPRIPSKDEF	701	715	YER091C	MET6
...[RK].[AVLIMFHRTP]P..P.....	LKIKPLPYKPRGRHS	585	599	YER093C	TSC11
...R..[FLIYM].[FLIYM]P.....	FKTREFMSLPQALET	98	112	YER093C-A	YER093C-A
...R..[FLIYM].[FLIYM]P.....	QNVRFKLIFPEVVQE	146	160	YER100W	UBC6
...[RK].[AVLIMFHRTP]P..P.....	LPTKLIPSIPGGKLI	313	327	YER105C	NUP157
...[RK].[AVLIMFHRTP]P..P.....	VSPRRAPKPPSYPSP	437	451	YER114C	BOI2
...[RK].[AVLIMFHRTP]P..P.....	YCFKLLPPQPGSKKG	842	856	YER114C	BOI2
...R..[FLIYM].[FLIYM]P.....	TKFRISLGLPVGAIM	9	23	YER117W	RPL23B
...[RK].[AVLIMFHRTP]P..P.....	SKIRPTPRKPSRMAT	5	19	YER118C	SHO1
...R..[FLIYM].[FLIYM]P.....	LKGRISYLLPPQSHD	178	192	YER119C	AVT6
...[RK].[AVLIMFHRTP]P..P.....	VNEKEVPAEPETQPP	162	176	YER120W	SCS2
...[KR][KR][AHPKRLG]P[PLV].......	TASRKKPVLNSQDKK	220	234	YER122C	GLO3
...R..[FLIYM].[FLIYM]P.....	LDLRTNLYIPDILDN	288	302	YER124C	DSE1
.[FPLWA].[WYLMFHP].[AVLIMFHPR]P..P.....	QPSPIRPVLPQQKSS	668	682	YER129W	PAK1
...[RK].[AVLIMFHRTP]P..P.....	RKNRAPPQRPRFNRD	92	106	YER131W	RPS26B
......P.[ILMVPYAFTR]P.[RKW]...	EPLPPVPKAPSRRSS	940	954	YER132C	PMD1
.[FPLWA].[WYLMFHP].[AVLIMFHPR]P..P.....	LPVLNIPLPPQEKIP	924	938	YER132C	PMD1
...PPPP...	ASPQPASVPPPQNGP	70	84	YER137C-A	YER137C-A
...PPPP...	SVPPPQNGPYPQQCM	76	90	YER137C-A	YER137C-A
...PPPP...	ASPQPASVPPPQNGP	70	84	YER138C	YER138C
...PPPP...	SVPPPQNGPYPQQCM	76	90	YER138C	YER138C
.[FPLWA].[WYLMFHP].[AVLIMFHPR]P..P.....	IADLPLPDLPPESPT	1128	1142	YER138C	YER138C
.[FPLWA].[WYLMFHP].[AVLIMFHPR]P..P.....	IPKLNVPLNPKGRKL	1493	1507	YER138C	YER138C
...R..[FLIYM].[FLIYM]P.....	LEDRLGLPLPAFVVL	388	402	YER140W	YER140W
......P.[ILMVPYAFTR]P.[RKW]...	ASQLRKPRAPMRNVI	446	460	YER141W	COX15
......P.[ILMVPYAFTR]P.[RKW]...	TTSSDKPLTPTKTSS	338	352	YER143W	DDI1
.[FPLWA].[WYLMFHP].[AVLIMFHPR]P..P.....	VFSPRIPPLPQQNLS	413	427	YER144C	UBP5
...PPPP...	SMYPKTSSPPPPTPT	13	27	YER151C	UBP3
...PPPP...	PKTSSPPPPTPTNMQ	16	30	YER151C	UBP3
...PPPP...	SSPPPPTPTNMQIPI	19	33	YER151C	UBP3
...R..[FLIYM].[FLIYM]P.....	GQFRSVLDIPNNKES	703	717	YER151C	UBP3
......P.[ILMVPYAFTR]P.[RKW]...	TPSDELPSPPLRMVH	234	248	YER152C	YER152C
...[KR][KR][AHPKRLG]P[PLV].......	VMPRRLPVEGRLLCE	70	84	YER153C	PET122
[FPLWA]..[WYLMFHP].[AVLIMFHPP]P..P.....	LISHLFPTNPEFSKK	651	665	YER155C	BEM2
...PPPP...	NRIPVLPPPRSPNRP	358	372	YER158C	YER158C
...PPPP...	PVLPPPRSPNRPTLS	361	375	YER158C	YER158C
.[FPLWA].[WYLMFHP].[AVLIMFHPR]P..P.....	IPVLPPPRSPNRPTL	360	374	YER158C	YER158C
...R..[FLIYM].[FLIYM]P.....	PSRRDSLSIPRAVDA	23	37	YER158C	YER158C
..[KRP]......P.[KR]P..	QVPVTTQLPPIKPEH	4	18	YER159C	BUR6
...PPPP...	ASPQPASVPPPQNGP	70	84	YER159C-A	YER159C-A
...PPPP...	SVPPPQNGPYPQQCM	76	90	YER159C-A	YER159C-A
...PPPP...	ASPQPASVPPPQNGP	70	84	YER160C	YER160C
...PPPP...	SVPPPQNGPYPQQCM	76	90	YER160C	YER160C
...PPPP...	ADLPLPDLPPEPPTE	1129	1143	YER160C	YER160C
...PPPP...	PLPDLPPEPPTELSD	1132	1146	YER160C	YER160C
.[FPLWA].[WYLMFHP].[AVLIMFHPR]P..P.....	IADLPLPDLPPEPPT	1128	1142	YER160C	YER160C
.[FPLWA].[WYLMFHP].[AVLIMFHPR]P..P.....	LPLPDLPPEPPTELS	1131	1145	YER160C	YER160C
.[FPLWA].[WYLMFHP].[AVLIMFHPR]P..P.....	IPKLNVPLNPKGRKL	1493	1507	YER160C	YER160C
...[RK].[AVLIMFHRTP]P..P.....	TDHRGTPANPGRVAT	44	58	YER163C	YER163C
...[RK].[AVLIMFHRTP]P..P.....	IGSKKLPTGPSKKRQ	1277	1291	YER164W	CHD1
...[RK].[AVLIMFHRTP]P..P.....	KRMKALPKGPAALIN	1317	1331	YER164W	CHD1
......P.[ILMVPYAFTR]P.[RKW]...	RLSPNSPTPPLKSKV	1334	1348	YER164W	CHD1
..[KRP]......P.[KR]P..	YPPGYDPTDPNRPKV	1423	1437	YER166W	DNF1
...R..[FLIYM].[FLIYM]P.....	ERARTSLDLPGVTNA	1547	1561	YER166W	DNF1
..[KRP]....P...[KR]P..	VPKSTHDPSLAKPPS	235	249	YER167W	BCK2
..[KRP]....P...[KR]P..	QLPNPAMPLLHRPTL	386	400	YER169W	RPH1
.[FPLWA].[WYLMFHP].[AVLIMFHPR]P..P.....	TLAFPRPNGPSGLNP	528	542	YER169W	RPH1
.[FPLWA].[WYLMFHP].[AVLIMFHPR]P..P.....	NLQYNPPKVPGLDDI	156	170	YER170W	ADK2
...PPPP...	KLPKKFPPPTPLLEN	1312	1326	YER172C	BRR2
...[RK].[AVLIMFHRTP]P..P.....	LPKKFPPPTPLLENI	1313	1327	YER172C	BRR2

Table S6

Motif	Motif Match	Start	End	ORF	Common name
...[KR][KR][AHPKRLG]P[PLV].......	KLSKRLPLRFPEHTS	1904	1918	YER172C	BRR2
...R..[FLIYM].[FLIYM]P.....	LSKRLPLRFPEHTSS	1905	1919	YER172C	BRR2
...[KR][KR][AHPKRLG]P[PLV].......	RQPRKAPVISESLSD	639	653	YER173W	RAD24
.[FPLWA].[WYLMFHP].[AVLIMFHPR]P..P.....	YADPIFPDYPEFDDL	150	164	YER175C	TMT1
......P.[ILMVPYAFTR]P.[RKW]...	AKLQDSPDMPEKECV	307	321	YER179W	DMC1
...[RK].[AVLIMFHRTP]P..P.....	RVTRKRPREPKSTND	643	657	YER190W	YRF1-2
......P.[ILMVPYAFTR]P.[RKW]...	VCKLRSPNTPRRLRK	195	209	YER190W	YRF1-2
..[KRP]......P.[KR]P..	TERLKRDLCPRKPTE	115	129	YER190W	YRF1-2
...PPPP...	VSPQPASVPPPQNGQ	70	84	YFL002W-A	YFL002W-A
.[FPLWA].[WYLMFHP].[AVLIMFHPR]P..P.....	LPKLNVPLNPKGKKL	1508	1522	YFL002W-A	YFL002W-A
....RP[AVLIMFHRKTP][AVLIMFHRTP][AVLIMFHRTP]..[LP]...	ELYIRPPPHLGLNDK	1374	1388	YFL002W-A	YFL002W-A
...PPPP...	VSPQPASVPPPQNGQ	70	84	YFL002W-B	YFL002W-B
...R..[FLIYM].[FLIYM]P.....	VYTRTAFQIPGDDKI	403	417	YFL004W	VTC2
.[FPLWA].[WYLMFHP].[AVLIMFHPR]P..P.....	AFPYVFPLPPWIPKQ	2071	2085	YFL007W	BLM3
...PPPP...	GTTWPRPKGPPPGVN	43	57	YFL010C	WWM1
..[KRP]......P.[KR]P..	ETREASLDIPYKPII	30	44	YFL011W	HXT10
.[FPLWA].[WYLMFHP].[AVLIMFHPR]P..P.....	EASLDIPYKPIIAYW	33	47	YFL011W	HXT10
...[RK].[AVLIMFHRTP]P..P.....	NRKKVTPQLPKVTPA	530	544	YFL013C	IES1
...[RK].[AVLIMFHRTP]P..P.....	QLPKVTPAAPTETEE	537	551	YFL013C	IES1
..[KRP]....P...[KR]P..	LLRRNPSPSIVKPGS	256	270	YFL021W	GAT1
[FPLWA]..[WYLMFHP].[AVLIMFHPP]P..P.....	LTSPNMPLSPLLLAT	766	780	YFL033C	RIM15
...PPPP...	YKPPSTPKINPPQSP	1024	1038	YFL034W	YFL034W
......P.[ILMVPYAFTR]P.[RKW]...	QDEMVLPPAPPKNSD	761	775	YFL036W	RPO41
......P.[ILMVPYAFTR]P.[RKW]...	LLPLRLPEIPPKGDF	1324	1338	YFL036W	RPO41
[FPLWA]..[WYLMFHP].[AVLIMFHPP]P..P.....	AIVWRLPKMPKIKIT	6	20	YFL040W	YFL040W
...[RK].[AVLIMFHRTP]P..P.....	FDLKGLPRQPEPLPK	551	565	YFL041W	FET5
...PPPP...	ESFSLPPPQPKPKRV	79	93	YFL044C	YFL044C
...PPPP...	SLPPPQPKPKRVLKS	82	96	YFL044C	YFL044C
....RP[AS]....Y...	DIRNRPAFGFHYWNS	217	231	YFL055W	AGP3
....RP[AVLIMFHRKTP][AVLIMFHRTP][AVLIMFHRTP]..[LP]...	VNAPRPRAVVPPPNS	20	34	YFL061W	YFL061W
..[KRP]......P.[KR]P..	TERLKRDLCPRKPIE	60	74	YFL065C	YFL065C
...R..[FLIYM].[FLIYM]P.....	HKDRIVFCLPFFPAL	35	49	YFL068W	YFL068W
......P.[ILMVPYAFTR]P.[RKW]...	SISSLDPKLPSWGNK	174	188	YFR002W	NIC96
....P.R.A[VP].....	LDGDPYRLAVYKLIG	402	416	YFR002W	NIC96
...[RK].[AVLIMFHRTP]P..P.....	KPKRKRPAPPKKKAP	79	93	YFR013W	IOC3
...[KR][KR][AHPKRLG]P[PLV].......	SAKKKGPLTTWENLK	274	288	YFR013W	IOC3
...R..[FLIYM].[FLIYM]P.....	DHDRVSMAMPLQTDQ	27	41	YFR013W	IOC3
....RP[AVLIMFHRKTP][AVLIMFHRTP][AVLIMFHRTP]..[LP]...	DPSKRPTAAELLEDP	283	297	YFR014C	CMK1
[FPLWA]..[WYLMFHP].[AVLIMFHPP]P..P.....	ARPLSVPGSPKVRSN	647	661	YFR015C	GSY1
...PPPP...	LFISTPPPPPKMAIP	388	402	YFR019W	FAB1
...PPPP...	STPPPPPKMAIPATK	391	405	YFR019W	FAB1
......P.[ILMVPYAFTR]P.[RKW]...	KLFISTPPPPPKMAI	387	401	YFR019W	FAB1
...[KR][KR][AHPKRLG]P[PLV].......	KSTKKKPLLFDIRLK	7	21	YFR022W	YFR022W
...R..[FLIYM].[FLIYM]P.....	RNQRDNLGLPPSASS	515	529	YFR022W	YFR022W
......PR.P.R...	AIQNVQPRKPSRVHS	631	645	YFR022W	YFR022W
....RP[AVLIMFHRKTP][AVLIMFHRTP][AVLIMFHRTP]..[LP]...	SGRIRPPPAVDPLFR	187	201	YFR024C-A	LSB3
......P.[ILMVPYAFTR]P.[RKW]...	LAQVDPPFMPFRDAG	117	131	YFR028C	CDC14
...[RK].[AVLIMFHRTP]P..P.....	TDTKLFPGNPNLMCI	525	539	YFR029W	PTR3
...[RK].[AVLIMFHRTP]P..P.....	VENKLRPDLPVKNFV	638	652	YFR030W	MET10
[FPLWA]..[WYLMFHP].[AVLIMFHPP]P..P.....	PSVMKLPPSPKQPVI	872	886	YFR030W	MET10
....RP[AVLIMFHRKTP][AVLIMFHRTP][AVLIMFHRTP]..[LP]...	LLQFRPAPMYILDEV	1100	1114	YFR031C	SMC2
...[RK].[AVLIMFHRTP]P..P.....	YRRKCIPFAPHQITA	170	184	YFR032C	YFR032C
[FPLWA]..[WYLMFHP].[AVLIMFHPP]P..P.....	LYYHEVPISPIGNAG	70	84	YFR032C-B	YFR032C-B
...[RK].[AVLIMFHRTP]P..P.....	EFAKFAPDLPVLKYY	288	302	YFR038W	YFR038W
...R..[FLIYM].[FLIYM]P.....	LLIRWLILMPLVGSR	25	39	YFR042W	YFR042W
......P.[ILMVPYAFTR]P.[RKW]...	YVAYTLPLLPGKGGF	225	239	YFR048W	RMD8
[FPLWA]..[WYLMFHP].[AVLIMFHPP]P..P.....	FTIPVFPQEPVDINT	433	447	YFR051C	RET2
......P.[ILMVPYAFTR]P.[RKW]...	KKGGNIPMIPGWVME	58	72	YFR053C	HXK1
......P.[ILMVPYAFTR]P.[RKW]...	FSPHSTPVTPRRLFT	168	182	YGL003C	CDH1
...[RK].[AVLIMFHRTP]P..P.....	QYTKGRPLAPKGAEW	254	268	YGL009C	LEU1
......P.[ILMVPYAFTR]P.[RKW]...	AYMGLEPNTPLKSIK	337	351	YGL009C	LEU1
..[KRP]......P.[KR]P..	DIPVKSDDTPAKPSS	528	542	YGL009C	LEU1
....P.R.A[VP].....	FDNVPKRKAVTTTFD	759	773	YGL009C	LEU1
....RP[AVLIMFHRKTP][AVLIMFHRTP][AVLIMFHRTP]..[LP]...	FEKRRPALIDNLVQS	118	132	YGL010W	YGL010W
...[RK].[AVLIMFHRTP]P..P.....	TGRKTFPFLPYQILK	365	379	YGL012W	ERG4
.[FPLWA].[WYLMFHP].[AVLIMFHPR]P..P.....	DFFMGAPLNPRWGIL	211	225	YGL012W	ERG4
...PPPP...	PHNPCPFFPPPDFND	85	99	YGL014W	PUF4
...PPPP...	ILPPQQNTPPPPWLY	430	444	YGL014W	PUF4
...PPPP...	NTPPPPWLYSTPPPF	436	450	YGL014W	PUF4
...PPPP...	STPPPFNAMVPPHLL	445	459	YGL014W	PUF4
.[FPLWA].[WYLMFHP].[AVLIMFHPR]P..P.....	QPNPIMPGHPHNISS	316	330	YGL014W	PUF4
...[RK].[AVLIMFHRTP]P..P.....	CHSKYIPLKPIQDMI	315	329	YGL017W	ATE1

Table S6

Motif	Motif Match	Start	End	ORF	Common name
...R..[FLIYM].[FLIYM]P.....	HALRNWLGLPIDIRN	99	113	YGL022W	STT3
...[RK].[AVLIMFHRTP]P..P.....	RDGRGVPLGPRNHDY	23	37	YGL023C	PIB2
..[KRP]......P.[KR]P..	AAPIAAPKKPRKPRQ	204	218	YGL025C	PGD1
......P.[ILMVPYAFTR]P.[RKW]...	YLTSALPILPKREVV	16	30	YGL028C	SCW11
...R..[FLIYM].[FLIYM]P.....	NGDRKCYQLPPAGRG	229	243	YGL040C	HEM2
...PPPP...	MTDQRGPPPPHPQQA	1	15	YGL049C	TIF4632
...PPPP...	QRGPPPPHPQQANGY	4	18	YGL049C	TIF4632
...PPPP...	TMPPPQSPSNCPASP	463	477	YGL056C	SDS23
...[RK].[AVLIMFHRTP]P..P.....	EIVKLTPKNPFYKLP	181	195	YGL056C	SDS23
.[FPLWA].[WYLMFHP].[AVLIMFHPR]P..P.....	SATMPPPQSPSNCPA	461	475	YGL056C	SDS23
...PPPP...	SVPIMPTLPPRPYIT	535	549	YGL060W	YBP2
......P.[ILMVPYAFTR]P.[RKW]...	NSVPIMPTLPPRPYI	534	548	YGL060W	YBP2
[FPLWA]..[WYLMFHP].[AVLIMFHPP]P..P.....	PIMPTLPPRPYITIN	537	551	YGL060W	YBP2
...R..[FLIYM].[FLIYM]P.....	LLRRLLYLMPESTSF	467	481	YGL060W	YBP2
..[KRP]......P.[KR]P..	NRKGEKDAAPAKPPN	172	186	YGL061C	DUO1
..[KRP]....P...[KR]P..	RVRKTHVPASKRPSG	212	226	YGL061C	DUO1
[FPLWA]..[WYLMFHP].[AVLIMFHPP]P..P.....	ANVPTVPGTPGPIET	146	160	YGL062W	PYC1
...[RK].[AVLIMFHRTP]P..P.....	GPRKYIPRAPAQGLL	288	302	YGL063W	PUS2
..[KRP]......P.[KR]P..	SEKIMPFLSPPRPLS	429	443	YGL064C	MRH4
....P.R.A[VP].....	LILPPVRDAVKEIIS	104	118	YGL064C	MRH4
...[RK].[AVLIMFHRTP]P..P.....	AFIKILPNNPWPFLF	458	472	YGL065C	ALG2
......P.[ILMVPYAFTR]P.[RKW]...	NNNAGSPIRPYKQRY	523	537	YGL073W	HSF1
......P.[ILMVPYAFTR]P.[RKW]...	MVHILSPGYPNKSFN	650	664	YGL073W	HSF1
....RP[AVLIMFHRKTP][AVLIMFHRTP][AVLIMFHRTP]..[LP]...	SHKSRPAFVNKLWSM	168	182	YGL073W	HSF1
...[RK].[AVLIMFHRTP]P..P.....	KNRKNLPTIPIRLCG	140	154	YGL085W	YGL085W
...R..[FLIYM].[FLIYM]P.....	KNERTFFDFPVMDST	210	224	YGL095C	VPS45
...PPPP...	PLLPPLPGQPPLINI	325	339	YGL099W	LSG1
......P.[ILMVPYAFTR]P.[RKW]...	AKDLIVPRRPEWNEG	131	145	YGL099W	LSG1
..[KRP]....P...[KR]P..	APKKWKAPKGPKPTH	6	20	YGL099W	LSG1
[FPLWA]..[WYLMFHP].[AVLIMFHPP]P..P.....	PKKWKAPKGPKPTHR	7	21	YGL099W	LSG1
[FPLWA]..[WYLMFHP].[AVLIMFHPP]P..P.....	PNEPLLPPLPGQPPL	322	336	YGL099W	LSG1
.[FPLWA].[WYLMFHP].[AVLIMFHPR]P..P.....	LLIHSLPRKPLLMTS	358	372	YGL104C	VPS73
.[FPLWA].[WYLMFHP].[AVLIMFHPR]P..P.....	RLDMRVPVWPMALRF	239	253	YGL111W	NSA1
.[FPLWA].[WYLMFHP].[AVLIMFHPR]P..P.....	EPLPQVPRLPTFTTH	123	137	YGL112C	TAF6
...R..[FLIYM].[FLIYM]P.....	VIVREKLPFPSGSAT	159	173	YGL114W	YGL114W
....RP[AS]....Y...	IHTRRPSSTSQYLIR	596	610	YGL116W	CDC20
...[RK].[AVLIMFHRTP]P..P.....	GRCRLFPHCPLGRSC	260	274	YGL122C	NAB2
...[KR][KR][AHPKRLG]P[PLV].......	LAAKRKPVQTGIVLC	326	340	YGL122C	NAB2
..[KRP]....P...[KR]P..	AAPNSNVPTNERPFA	485	499	YGL122C	NAB2
......P.[ILMVPYAFTR]P.[RKW]...	GYPECHPELPNKDVK	155	169	YGL125W	MET13
.[FPLWA].[WYLMFHP].[AVLIMFHPR]P..P.....	AAGMDVPIIPGIMPI	206	220	YGL125W	MET13
...[RK].[AVLIMFHRTP]P..P.....	MESRVAPEIPGLIQP	5	19	YGL130W	CEG1
......P.[ILMVPYAFTR]P.[RKW]...	VNGFRFPRLPQKKKE	103	117	YGL130W	CEG1
...R..[FLIYM].[FLIYM]P.....	LYKRKPILLPDPKPL	3	17	YGL133W	ITC1
.[FPLWA].[WYLMFHP].[AVLIMFHPR]P..P.....	NASFELPLKPKFLQS	31	45	YGL134W	PCL10
.[FPLWA].[WYLMFHP].[AVLIMFHPR]P..P.....	EFRFIFPLQPLFILI	393	407	YGL142C	GPI10
...[RK].[AVLIMFHRTP]P..P.....	LVNKLLPPHPFADKP	140	154	YGL143C	MRF1
......P.[ILMVPYAFTR]P.[RKW]...	ASSILLPPLPERAYI	456	470	YGL144C	ROG1
....RP[AVLIMFHRKTP][AVLIMFHRTP][AVLIMFHRTP]..[LP]...	INSDRPKIVRELIKM	328	342	YGL150C	INO80
......P.[ILMVPYAFTR]P.[RKW]...	LYEAMPPTLPHRDWK	81	95	YGL153W	PEX14
...R..[FLIYM].[FLIYM]P.....	GGERTEYVLPKTSDG	153	167	YGL156W	AMS1
.[FPLWA].[WYLMFHP].[AVLIMFHPR]P..P.....	PLYWLLPSHPYTIAS	298	312	YGL160W	YGL160W
...PPPP...	AIVPPPQNYTPPLFE	94	108	YGL162W	SUT1
...[RK].[AVLIMFHRTP]P..P.....	MARRRLPDRPPNGIG	1	15	YGL163C	RAD54
....RP[AVLIMFHRKTP][AVLIMFHRTP][AVLIMFHRTP]..[LP]...	GAGERPRLVPRPINV	15	29	YGL163C	RAD54
..[KRP]....P...[KR]P..	DFRNPLLPIDNRPSP	266	280	YGL170C	SPO74
...[KR][KR][AHPKRLG]P[PLV].......	FLEKKRPLQVALLSK	220	234	YGL171W	ROK1
...PPPP...	FGQPISFPPPPPMTN	1438	1452	YGL173C	KEM1
...PPPP...	PISFPPPPPMTNVSD	1441	1455	YGL173C	KEM1
......P.[ILMVPYAFTR]P.[RKW]...	AATTSTPPLPRRRAT	244	258	YGL181W	GTS1
...R..[FLIYM].[FLIYM]P.....	EQIRKEFGIPEEFKE	201	215	YGL183C	MND1
...[RK].[AVLIMFHRTP]P..P.....	RKNRAPPQRPRFNRE	92	106	YGL189C	RPS26A
......P.[ILMVPYAFTR]P.[RKW]...	FSCLRDPVIPIKLAA	2561	2575	YGL195W	GCN1
.[FPLWA].[WYLMFHP].[AVLIMFHPR]P..P.....	VAAFKLPRGPNCVLP	2199	2213	YGL195W	GCN1
.[FPLWA].[WYLMFHP].[AVLIMFHPR]P..P.....	IPMFLRPFIPQLQRT	2284	2298	YGL195W	GCN1
...PPPP...	QTREPNEPPPPCPAM	868	882	YGL197W	MDS3
...PPPP...	EPNEPPPPCPAMSTG	871	885	YGL197W	MDS3
...[RK].[AVLIMFHRTP]P..P.....	WHPKTIPKQPMEITT	261	275	YGL197W	MDS3
......P.[ILMVPYAFTR]P.[RKW]...	IRMPSNPALPKKLLN	342	356	YGL197W	MDS3
[FPLWA]..[WYLMFHP].[AVLIMFHPP]P..P.....	LGVLNVPLPPQTREP	858	872	YGL197W	MDS3
...R..[FLIYM].[FLIYM]P.....	KRNRLKILLPFLEQS	857	871	YGL206C	CHC1
....P.R.A[VP].....	FQGVPNRSAVFCMPT	834	848	YGL207W	SPT16

Table S6

Motif	Motif Match	Start	End	ORF	Common name
...[RK].[AVLIMFHRTP]P..P.....	NTPRSVPNSPNDGYL	202	216	YGL209W	MIG2
...R..[FLIYM].[FLIYM]P.....	RRERAKFEIPEEQEE	316	330	YGL209W	MIG2
...PPPP...	QQHQQPPILPPPGLM	79	93	YGL215W	CLG1
...PPPP...	QQPPILPPPGLMYTS	82	96	YGL215W	CLG1
...PPPP...	LLPAAPQLPPPHVHN	406	420	YGL215W	CLG1
.[FPLWA].[WYLMFHP].[AVLIMFHPR]P..P.....	SLLPAAPQLPPPHVH	405	419	YGL215W	CLG1
......P.[ILMVPYAFTR]P.[RKW]...	IRDLLKPETPSKRLV	246	260	YGL216W	KIP3
...R..[FLIYM].[FLIYM]P.....	VSIRDKYVIPEKISL	405	419	YGL219C	MDM34
..[KRP]....P...[KR]P..	IPPPQTLPNGQKPNF	52	66	YGL222C	EDC1
......P.[ILMVPYAFTR]P.[RKW]...	LQDILKPDIPLRNML	135	149	YGL224C	SDT1
...[RK].[AVLIMFHRTP]P..P.....	IIPRIFPSTPGTIDS	771	785	YGL233W	SEC15
...R..[FLIYM].[FLIYM]P.....	ILARAILTIPRVLDK	455	469	YGL241W	KAP114
..[KRP]....P...[KR]P..	YYRLDALPKDGKPYV	677	691	YGL245W	YGL245W
....RP[AVLIMFHRKTP][AVLIMFHRTP][AVLIMFHRTP]..[LP]...	HADNRPVLIFCPTRA	368	382	YGL251C	HFM1
...R..[FLIYM].[FLIYM]P.....	VSDRRALQLPAVGLF	299	313	YGL252C	RTG2
......P.[ILMVPYAFTR]P.[RKW]...	KKGGNIPMIPGWVMD	58	72	YGL253W	HXK2
[FPLWA]..[WYLMFHP].[AVLIMFHPP]P..P.....	LQELSLPSRPVMPSF	428	442	YGR002C	GOD1
...R..[FLIYM].[FLIYM]P.....	LGYRAHISLPDDTSL	130	144	YGR012W	YGR012W
...[RK].[AVLIMFHRTP]P..P.....	HDPKFIPNKPYRQFN	336	350	YGR019W	UGA1
...[RK].[AVLIMFHRTP]P..P.....	IPSKDHPYDPEKDSV	94	108	YGR020C	VMA7
...[RK].[AVLIMFHRTP]P..P.....	LWAKFFPEKPLNIKH	112	126	YGR024C	THG1
.[FPLWA].[WYLMFHP].[AVLIMFHPR]P..P.....	IADLPLPDLPPESPT	1128	1142	YGR027W-B	YGR027W-B
.[FPLWA].[WYLMFHP].[AVLIMFHPR]P..P.....	IPKLNVPLNPKGRKL	1493	1507	YGR027W-B	YGR027W-B
[FPLWA]..[WYLMFHP].[AVLIMFHPP]P..P.....	AIEMALPNDPYPAWT	157	171	YGR032W	GSC2
....RP[AVLIMFHRKTP][AVLIMFHRTP][AVLIMFHRTP]..[LP]...	NGRRRPKFRVQLSGN	1080	1094	YGR032W	GSC2
...R..[FLIYM].[FLIYM]P.....	DTIRSGYGMPSAHSQ	96	110	YGR036C	CAX4
...PPPP...	ASPQPASVPPPQNGP	70	84	YGR038C-A	YGR038C-A
...PPPP...	SVPPPQNGPYPQQCM	76	90	YGR038C-A	YGR038C-A
...PPPP...	ASPQPASVPPPQNGP	70	84	YGR038C-B	YGR038C-B
...PPPP...	SVPPPQNGPYPQQCM	76	90	YGR038C-B	YGR038C-B
.[FPLWA].[WYLMFHP].[AVLIMFHPR]P..P.....	IADLPLPDLPPESPT	1128	1142	YGR038C-B	YGR038C-B
.[FPLWA].[WYLMFHP].[AVLIMFHPR]P..P.....	IPKLNVPLNPKGRKL	1493	1507	YGR038C-B	YGR038C-B
...R..[FLIYM].[FLIYM]P.....	VTHRLRISIPGITGR	204	218	YGR038W	ORM1
......P.[ILMVPYAFTR]P.[RKW]...	EMVSGKPLFPGRDYH	218	232	YGR040W	KSS1
..[KRP]......P.[KR]P..	RAKEYIANLPMRPPL	257	271	YGR040W	KSS1
...[RK].[AVLIMFHRTP]P..P.....	GGGRIVPGVPPGAAK	517	531	YGR054W	YGR054W
...R..[FLIYM].[FLIYM]P.....	VNGRKFFKYPSPIAD	506	520	YGR056W	RSC1
....RP[AVLIMFHRKTP][AVLIMFHRTP][AVLIMFHRTP]..[LP]...	VRRGRPPVIDLPYVL	225	239	YGR056W	RSC1
...PPPP...	QKPAGRPIPPAPTHY	100	114	YGR058W	YGR058W
...[RK].[AVLIMFHRTP]P..P.....	PAGRPIPPAPTHYNN	102	116	YGR058W	YGR058W
...[RK].[AVLIMFHRTP]P..P.....	LLIKTVPGFPLLENL	139	153	YGR061C	ADE6
...[RK].[AVLIMFHRTP]P..P.....	FALKNTPITPGSCLI	463	477	YGR061C	ADE6
......P.[ILMVPYAFTR]P.[RKW]...	IGFFVIPGVPSKCYS	307	321	YGR065C	VHT1
.[FPLWA].[WYLMFHP].[AVLIMFHPR]P..P.....	PFMYLLPRFPSHIIL	183	197	YGR065C	VHT1
...R..[FLIYM].[FLIYM]P.....	SSLRDNILYPYLIYS	644	658	YGR067C	YGR067C
......P.[ILMVPYAFTR]P.[RKW]...	MLLFISPHLPMKGRL	468	482	YGR068C	YGR068C
.[FPLWA].[WYLMFHP].[AVLIMFHPR]P..P.....	TAHLALPQPPPNYHE	523	537	YGR068C	YGR068C
...R..[FLIYM].[FLIYM]P.....	SSHRKILELPENGVT	172	186	YGR068C	YGR068C
...R..[FLIYM].[FLIYM]P.....	LMKRGNYELPFNTML	227	241	YGR068C	YGR068C
....RP[AVLIMFHRKTP][AVLIMFHRTP][AVLIMFHRTP]..[LP]...	TVFQRPIPLPLLFIC	732	746	YGR070W	ROM1
..[KRP]....P...[KR]P..	RRRDFGAPANKRPRR	130	144	YGR074W	SMD1
......P.[ILMVPYAFTR]P.[RKW]...	KAINANPFLPNKHPV	35	49	YGR076C	MRPL25
...[RK].[AVLIMFHRTP]P..P.....	KNARGIPQAPFIENV	10	24	YGR078C	PAC10
......P.[ILMVPYAFTR]P.[RKW]...	LDHGSNPDLPNKSNL	304	318	YGR080W	TWF1
......P.[ILMVPYAFTR]P.[RKW]...	EISLIDPSTPDKAAK	350	364	YGR083C	GCD2
...PPPP...	REPLQPRPPVKPLSK	85	99	YGR084C	MRP13
......P.[ILMVPYAFTR]P.[RKW]...	TREPLQPRPPVKPLS	84	98	YGR084C	MRP13
..[KRP]....P...[KR]P..	RDPKTREPLQPRPPV	80	94	YGR084C	MRP13
.[FPLWA].[WYLMFHP].[AVLIMFHPR]P..P.....	PLQPRPPVKPLSKQT	87	101	YGR084C	MRP13
...PPPP...	SQLQNPPPPPSTTKG	16	30	YGR086C	PIL1
...PPPP...	QNPPPPPSTTKGRFF	19	33	YGR086C	PIL1
[FPLWA]..[WYLMFHP].[AVLIMFHPP]P..P.....	AECLGVPWEPVDLEG	548	562	YGR088W	CTT1
...R..[FLIYM].[FLIYM]P.....	FFLRDAIKFPVFIHS	153	167	YGR088W	CTT1
...[KR][KR][AHPKRLG]P[PLV].......	NSPKKLPPKFYERAT	82	96	YGR092W	DBF2
[FPLWA]..[WYLMFHP].[AVLIMFHPP]P..P.....	LRSMIPPFTPQLDSE	485	499	YGR092W	DBF2
......P.[ILMVPYAFTR]P.[RKW]...	GKFIQHPFLPRKIPI	405	419	YGR094W	VAS1
.[FPLWA].[WYLMFHP].[AVLIMFHPR]P..P.....	ALKLIHPFMPFISEE	896	910	YGR094W	VAS1
......P.[ILMVPYAFTR]P.[RKW]...	NWKSNDPIPPPRKVS	454	468	YGR097W	ASK10
..[KRP]....P...[KR]P..	ESKMSLAPHEYKPVQ	219	233	YGR097W	ASK10
...R..[FLIYM].[FLIYM]P.....	RKRRTHLRLPLIRSN	1189	1203	YGR098C	ESP1
...R..[FLIYM].[FLIYM]P.....	LLSRFHYQLPLQVTI	1428	1442	YGR098C	ESP1
...[RK].[AVLIMFHRTP]P..P.....	GESRKAPLIPLLKQT	448	462	YGR099W	TEL2

Table S6

Motif	Motif Match	Start	End	ORF	Common name
......P.[ILMVPYAFTR]P.[RKW]...	FVIPVYPSLPERSKV	41	55	YGR099W	TEL2
..[KRP]....P...[KR]P..	ELRGLDDPTIVKPKF	548	562	YGR099W	TEL2
[FPLWA]..[WYLMFHP].[AVLIMFHPP]P..P.....	LIKFVIPVYPSLPER	38	52	YGR099W	TEL2
...R..[FLIYM].[FLIYM]P.....	EKFRLKYKLPANENI	29	43	YGR100W	MDR1
.....[GP]P.[IVL].P[FWY]...	PGEALPPHLSPWGDA	449	463	YGR103W	NOP7
...[KR][KR][AHPKRLG]P[PLV].......	NNQRRKPLLLNIGDH	1372	1386	YGR109W-B	YGR109W-B
....RP[AVLIMFHRKTP][AVLIMFHRTP][AVLIMFHRTP]..[LP]...	IKSHRPRLHGLLQPL	1148	1162	YGR109W-B	YGR109W-B
...[RK].[AVLIMFHRTP]P..P.....	ELLKTLPFYPTPSES	92	106	YGR110W	YGR110W
...[RK].[AVLIMFHRTP]P..P.....	LDEKRIPSTPVDYHA	197	211	YGR124W	ASN2
...R..[FLIYM].[FLIYM]P.....	DTARNLMSLPTNTPR	835	849	YGR125W	YGR125W
.....P.R.P.R...	TIYVPPLRRPPRDDL	239	253	YGR127W	YGR127W
.[FPLWA].[WYLMFHP].[AVLIMFHPR]P..P.....	TFLLTHPLFPLSRTR	524	538	YGR128C	UTP8
...R..[FLIYM].[FLIYM]P.....	RRARRQMGIPTISQV	160	174	YGR131W	YGR131W
..[KRP]....P...[KR]P..	DGKILPLPKADKPIP	1092	1106	YGR134W	CAF130
...PPPP...	QQAPLPYPPPFTNYY	169	183	YGR136W	LSB1
......P.[ILMVPYAFTR]P.[RKW]...	TNAKKDPFPPPKDLL	418	432	YGR140W	CBF2
...[RK].[AVLIMFHRTP]P..P.....	PPDRNLPSHPSSNNM	155	169	YGR143W	SKN1
...[RK].[AVLIMFHRTP]P..P.....	VCDRLIPTSPQKTNI	11	25	YGR146C	YGR146C
....RP[AVLIMFHRKTP][AVLIMFHRTP][AVLIMFHRTP]..[LP]...	FFTIRPPFFPKLALV	34	48	YGR146C-A	YGR146C-A
[FPLWA]..[WYLMFHP].[AVLIMFHPP]P..P.....	LVKFKLPFLPISDLN	549	563	YGR150C	YGR150C
......P.[ILMVPYAFTR]P.[RKW]...	ARFLPAPHRPEKYKE	64	78	YGR154C	YGR154C
...R..[FLIYM].[FLIYM]P.....	KGYRTIITLPEKMSN	96	110	YGR155W	CYS4
...PPPP...	TEVPIPRRPPPPQAA	136	150	YGR156W	PTI1
...PPPP...	PIPRRPPPPQAANNT	139	153	YGR156W	PTI1
...PPPP...	FAPPGPFMPPAQGPS	328	342	YGR156W	PTI1
.[FPLWA].[WYLMFHP].[AVLIMFHPR]P..P.....	VPIPRRPPPPQAANN	138	152	YGR156W	PTI1
..[KRP]....P...[KR]P..	DRRRLLGPAAAKPMA	5	19	YGR158C	MTR3
....RP[AVLIMFHRKTP][AVLIMFHRTP][AVLIMFHRTP]..[LP]...	TRAMRPAVARDPRRL	32	46	YGR161C	RTS3
...PPPP...	ASPQPASVPPPQNGP	70	84	YGR161C-C	YGR161C-C
...PPPP...	SVPPPQNGPYPQQCM	76	90	YGR161C-C	YGR161C-C
...PPPP...	ASPQPASVPPPQNGP	70	84	YGR161C-D	YGR161C-D
...PPPP...	SVPPPQNGPYPQQCM	76	90	YGR161C-D	YGR161C-D
...PPPP...	ADLPLPDLPPEPPTE	1129	1143	YGR161C-D	YGR161C-D
...PPPP...	PLPDLPPEPPTELSD	1132	1146	YGR161C-D	YGR161C-D
.[FPLWA].[WYLMFHP].[AVLIMFHPR]P..P.....	IADLPLPDLPPEPPT	1128	1142	YGR161C-D	YGR161C-D
.[FPLWA].[WYLMFHP].[AVLIMFHPR]P..P.....	LPLPDLPPEPPTELS	1131	1145	YGR161C-D	YGR161C-D
.[FPLWA].[WYLMFHP].[AVLIMFHPR]P..P.....	IPKLNVPLNPNGRKL	1493	1507	YGR161C-D	YGR161C-D
...PPPP...	VSPQPASVPPPQNGQ	70	84	YGR161W-A	YGR161W-A
...PPPP...	VSPQPASVPPPQNGQ	70	84	YGR161W-B	YGR161W-B
.[FPLWA].[WYLMFHP].[AVLIMFHPR]P..P.....	LPKLNVPLNPKGKKL	1508	1522	YGR161W-B	YGR161W-B
....RP[AVLIMFHRKTP][AVLIMFHRTP][AVLIMFHRTP]..[LP]...	ELYIRPPPHLGLNDK	1374	1388	YGR161W-B	YGR161W-B
...PPPP...	RAPPPKEEPAAPTST	922	936	YGR162W	TIF4631
......P.[ILMVPYAFTR]P.[RKW]...	DESATTPAIPTKSDE	361	375	YGR162W	TIF4631
......P.[ILMVPYAFTR]P.[RKW]...	RRRIAYPFYPFKKLG	29	43	YGR165W	MRPS35
..[KRP]....P...[KR]P..	TVPTNHVPNYIKPDL	78	92	YGR165W	MRPS35
...R..[FLIYM].[FLIYM]P.....	HFSRRRIAYPFYPFK	26	40	YGR165W	MRPS35
......P.[ILMVPYAFTR]P.[RKW]...	SNDYKIPVVPPRETY	504	518	YGR166W	KRE11
......P.[ILMVPYAFTR]P.[RKW]...	LPTGAPPTTPSRPRT	88	102	YGR170W	PSD2
...R..[FLIYM].[FLIYM]P.....	QNARLLIAYPLLIFY	224	238	YGR172C	YIP1
...R..[FLIYM].[FLIYM]P.....	MEERGFLGLPMALLE	460	474	YGR175C	ERG1
......P.[ILMVPYAFTR]P.[RKW]...	IIPLGNPKRPNWRLI	153	167	YGR177C	ATF2
.[FPLWA].[WYLMFHP].[AVLIMFHPR]P..P.....	QPMFYHPSMPQMMPV	623	637	YGR178C	PBP1
......P.[ILMVPYAFTR]P.[RKW]...	LSKVHDPNIPLRPKE	640	654	YGR184C	UBR1
.....P.R.P.R...	KLINTPGRKPERIVF	307	321	YGR188C	BUB1
...[KR][KR][AHPKRLG]P[PLV].......	TANKKAPPGYVIDSN	548	562	YGR196C	FYV8
...R..[FLIYM].[FLIYM]P.....	EFYRYGYIMPIHNAV	453	467	YGR197C	SNG1
...[RK].[AVLIMFHRTP]P..P.....	SNVRIPPTVPSKIID	4	18	YGR198W	YGR198W
......P.[ILMVPYAFTR]P.[RKW]...	SKPLQIPPLPLKLLT	304	318	YGR204W	ADE3
...R..[FLIYM].[FLIYM]P.....	LVSRLHYWFPQTVVK	194	208	YGR212W	YGR212W
...R..[FLIYM].[FLIYM]P.....	SLARLVLYLPNMWKF	817	831	YGR217W	CCH1
...[RK].[AVLIMFHRTP]P..P.....	TSFKGRPKPPKTLKH	456	470	YGR221C	TOS2
....RP[AVLIMFHRKTP][AVLIMFHRTP][AVLIMFHRTP]..[LP]...	SFKGRPKPPKTLKHV	457	471	YGR221C	TOS2
....P.R.A[VP].....	DVRRPIREAVNNRRK	2	16	YGR223C	YGR223C
...[KR][KR][AHPKRLG]P[PLV].......	LRIKRKPVQTFFEFI	324	338	YGR227W	DIE2
......P.[ILMVPYAFTR]P.[RKW]...	DFALPPPIIPLRKYG	690	704	YGR233C	PHO81
.[FPLWA].[WYLMFHP].[AVLIMFHPR]P..P.....	NANWMMPMFPSFGFI	725	739	YGR250C	YGR250C
.[FPLWA].[WYLMFHP].[AVLIMFHPR]P..P.....	GFIPQVPPVPYIIPP	737	751	YGR250C	YGR250C
...R..[FLIYM].[FLIYM]P.....	QVSRENYFIPLQYPN	693	707	YGR250C	YGR250C
...[RK].[AVLIMFHRTP]P..P.....	IFQKQLPKMPKEYIA	123	137	YGR252W	GCN5
...[RK].[AVLIMFHRTP]P..P.....	GWQRFLPTGPIAHLP	240	254	YGR255C	COQ6
...R..[FLIYM].[FLIYM]P.....	RLSRLLLSLPPESFT	269	283	YGR255C	COQ6
......P.[ILMVPYAFTR]P.[RKW]...	KEKSPIPEMPSWFSS	580	594	YGR258C	RAD2

Table S6

Motif	Motif Match	Start	End	ORF	Common name
....RP[AVLIMFHRKTP][AVLIMFHRTP][AVLIMFHRTP]..[LP]...	GPTARPVRLESLEDK	11	25	YGR258C	RAD2
......P.[ILMVPYAFTR]P.[RKW]...	LVHSKFPELPSKVHN	138	152	YGR264C	MES1
...R..[FLIYM].[FLIYM]P.....	WFYRYEIKLPMSNYE	110	124	YGR266W	YGR266W
...PPPP...	RLQSQPPRPPRPAAN	22	36	YGR268C	HUA1
...PPPP...	SQPPRPPRPAANLAQ	25	39	YGR268C	HUA1
...PPPP...	YTPTSSQPRPPPRPQ	67	81	YGR268C	HUA1
...PPPP...	TSSQPRPPPRPQQNP	70	84	YGR268C	HUA1
...PPPP...	QPRPPPRPQQNPSLP	73	87	YGR268C	HUA1
......P.[ILMVPYAFTR]P.[RKW]...	ERLQSQPPRPPRPAA	21	35	YGR268C	HUA1
......P.[ILMVPYAFTR]P.[RKW]...	TPTSSQPRPPPRPQQ	68	82	YGR268C	HUA1
..[KRP]....P...[KR]P..	NRPDAVDPALRRPGR	562	576	YGR270W	YTA7
..R[YFLEP]..[AVLIMFHWRTP]P[GSDLIAV]P.....	FDREFYFPLPDVKAR	577	591	YGR270W	YTA7
...R..[FLIYM].[FLIYM]P.....	NLDRSKISLPDFDDE	28	42	YGR271W	SLH1
....RP[AVLIMFHRKTP][AVLIMFHRTP][AVLIMFHRTP]..[LP]...	LLKLRPLPTSALQNP	1115	1129	YGR271W	SLH1
...R..[FLIYM].[FLIYM]P.....	ENSRVLIVLPYTPPS	4	18	YGR277C	YGR277C
...[RK].[AVLIMFHRTP]P..P.....	LHSKKIPEVPQTDDE	90	104	YGR281W	YOR1
......P.[ILMVPYAFTR]P.[RKW]...	LYDVHGPGVPVKTLE	383	397	YGR288W	MAL13
.....[GP]P.[IVL].P[FWY]...	NAGFTGPDVKPWFLL	454	468	YGR292W	MAL12
....RP[AVLIMFHRKTP][AVLIMFHRTP][AVLIMFHRTP]..[LP]...	GTIARPLFLVVLFFI	256	270	YGR295C	COS6
...[RK].[AVLIMFHRTP]P..P.....	RVTRKRPREPKSTND	821	835	YGR296W	YRF1-3
......P.[ILMVPYAFTR]P.[RKW]...	VCKLRSPNTPRRLRK	373	387	YGR296W	YRF1-3
..[KRP]......P.[KR]P..	TERLKRDLCPRKPTE	293	307	YGR296W	YRF1-3
......P.[ILMVPYAFTR]P.[RKW]...	APYLVQPNVPEKHNM	141	155	YHL002W	HSE1
...PPPP...	KFIPSRPAPKPPSSA	469	483	YHL007C	STE20
...PPPP...	EEQPLPPIPPTKSKT	532	546	YHL007C	STE20
...[RK].[AVLIMFHRTP]P..P.....	ANGKFIPSRPAPKPP	466	480	YHL007C	STE20
...[RK].[AVLIMFHRTP]P..P.....	IPSRPAPKPPSSASA	471	485	YHL007C	STE20
......P.[ILMVPYAFTR]P.[RKW]...	LKQTHAPTTPNRTSP	504	518	YHL007C	STE20
....P.R.A[VP].....	GKFIPSRPAPKPPSS	468	482	YHL007C	STE20
...R..[FLIYM].[FLIYM]P.....	ERDRKRLSLPEYEAR	266	280	YHL008C	YHL008C
...[KR][KR][AHPKRLG]P[PLV].......	NLVKKGPVRLPTKVL	49	63	YHL015W	RPS20
...[KR][KR][AHPKRLG]P[PLV].......	EAAKRHPVDGNYQGE	231	245	YHL016C	DUR3
......P.[ILMVPYAFTR]P.[RKW]...	LVRNIQPNIPLRSYR	779	793	YHL023C	RMD11
...PPPP...	MPMGPMPMGPPPPNA	484	498	YHL024W	RIM4
...PPPP...	GPMPMGPPPPNAASL	487	501	YHL024W	RIM4
...PPPP...	MMVYPMSPPPPSGLD	652	666	YHL024W	RIM4
......P.[ILMVPYAFTR]P.[RKW]...	AELQEKPLTPLKYTK	157	171	YHL025W	SNF6
......P.[ILMVPYAFTR]P.[RKW]...	PLQSKIPMLPSRRTM	502	516	YHL027W	RIM101
......P.[ILMVPYAFTR]P.[RKW]...	ASPFHDPILPRRTST	523	537	YHL028W	WSC4
...[RK].[AVLIMFHRTP]P..P.....	LSLRYAPNLPPVIRN	1328	1342	YHL035C	VMR1
..[KRP]....P...[KR]P..	EVPMFVNPANAKPLP	181	195	YHL038C	CBP2
...R..[FLIYM].[FLIYM]P.....	DFSRLTLKLPSTLVS	302	316	YHL039W	YHL039W
......P.[ILMVPYAFTR]P.[RKW]...	YRFARDPILPYRLVK	342	356	YHL040C	ARN1
......P.[ILMVPYAFTR]P.[RKW]...	SRLALVPFAPFKLLK	337	351	YHL047C	ARN2
..[KRP]......P.[KR]P..	TERLKRDLCPRKPTE	229	243	YHL049C	YHL049C
..[KRP]....P...[KR]P..	KSRHFTYPEGVKPQD	264	278	YHR006W	STP2
[FPLWA]..[WYLMFHP].[AVLIMFHPP]P..P.....	PINFVFPNLPLEHYR	238	252	YHR007C	ERG11
...[RK].[AVLIMFHRTP]P..P.....	NVEREAPMFPVPYYI	145	159	YHR014W	SPO13
...R..[FLIYM].[FLIYM]P.....	PIQRPELLYPNVNVY	193	207	YHR014W	SPO13
...[RK].[AVLIMFHRTP]P..P.....	TFFKLPPNLPEAESQ	95	109	YHR017W	YSC83
...R..[FLIYM].[FLIYM]P.....	LGYRTFFKLPPNLPE	91	105	YHR017W	YSC83
.[FPLWA].[WYLMFHP].[AVLIMFHPR]P..P.....	NPNFKAPKAPFMRLQ	384	398	YHR019C	DED81
[FPLWA]..[WYLMFHP].[AVLIMFHPP]P..P.....	LSKLVLPPPPVSITQ	82	96	YHR021W-A	ECM12
...R..[FLIYM].[FLIYM]P.....	FFQRYRILYPENSTT	726	740	YHR023W	MYO1
.[FPLWA].[WYLMFHP].[AVLIMFHPR]P..P.....	PLKFLRPTYPDLCSI	88	102	YHR027C	RPN1
....RP[AVLIMFHRKTP][AVLIMFHRTP][AVLIMFHRTP]..[LP]...	NAGIRPKFILALNDE	905	919	YHR027C	RPN1
..[KRP]......P.[KR]P..	NLPRHDADFPPRPQE	430	444	YHR030C	SLT2
...R..[FLIYM].[FLIYM]P.....	VPERFPIDIPRENVG	629	643	YHR031C	RRM3
[FPLWA]..[WYLMFHP].[AVLIMFHPP]P..P.....	LDALNIPHKPKQLSE	58	72	YHR033W	YHR033W
...R..[FLIYM].[FLIYM]P.....	VLCRDRIAFPKMKKK	150	164	YHR034C	PIH1
...[RK].[AVLIMFHRTP]P..P.....	LINRFMPHFPSSPSP	43	57	YHR036W	YHR036W
.[FPLWA].[WYLMFHP].[AVLIMFHPR]P..P.....	RFMPHFPSSPSPLRN	46	60	YHR036W	YHR036W
..[KRP]......P.[KR]P..	KPPKHIRNEPVKPFR	23	37	YHR037W	PUT2
....RP[AVLIMFHRKTP][AVLIMFHRTP][AVLIMFHRTP]..[LP]...	RLNCRPVTVPRLFNR	7	21	YHR038W	RRF1
.[FPLWA].[WYLMFHP].[AVLIMFHPR]P..P.....	FPNPELPDAPPVVGV	463	477	YHR042W	NCP1
...R..[FLIYM].[FLIYM]P.....	HVRRSNFRLPSNPST	520	534	YHR042W	NCP1
...R..[FLIYM].[FLIYM]P.....	INFRDFIGLPFDKCD	68	82	YHR064C	SSZ1
...[KR][KR][AHPKRLG]P[PLV].......	LLCRRLPVSKATKHL	23	37	YHR067W	RMD12
...[RK].[AVLIMFHRTP]P..P.....	LVRKVAPTKPMYCAS	478	492	YHR070W	TRM5
.[FPLWA].[WYLMFHP].[AVLIMFHPR]P..P.....	LLPYSLPMHPGRWWV	219	233	YHR072W	ERG7
...R..[FLIYM].[FLIYM]P.....	EISRGVIILPDTEDD	521	535	YHR073W	OSH3
....RP[AVLIMFHRKTP][AVLIMFHRTP][AVLIMFHRTP]..[LP]...	DPLKRPTAMKVLRHP	964	978	YHR079C	IRE1

Table S6

Motif	Motif Match	Start	End	ORF	Common name
...PPPP...	DGLPPQPVPPSSAPV	370	384	YHR082C	KSP1
...R..[FLIYM].[FLIYM]P.....	KKSRKPLGIPTPNTH	491	505	YHR082C	KSP1
.[FPLWA].[WYLMFHP].[AVLIMFHPR]P..P.....	AFTPAAPSMPISYDN	331	345	YHR084W	STE12
...R..[FLIYM].[FLIYM]P.....	GTLRNPYLIPDYPQP	242	256	YHR085W	IPI1
......P.[ILMVPYAFTR]P.[RKW]...	KNGMLIPNFPNKKLK	125	139	YHR086W	NAM8
..[KRP]......P.[KR]P..	FLPKPKVVGPPKPKN	122	136	YHR089C	GAR1
...R..[FLIYM].[FLIYM]P.....	SMDRGDLLLPLPKIK	101	115	YHR091C	MSR1
...[RK].[AVLIMFHRTP]P..P.....	KVNKCPPDHPYIQYE	278	292	YHR094C	HXT1
[FPLWA]..[WYLMFHP].[AVLIMFHPP]P..P.....	PGEHFAPMGPNGQRS	251	265	YHR098C	SFB3
..[KRP]....P...[KR]P..	TLRLLYNPSLCKPSL	9	23	YHR100C	YHR100C
...[RK].[AVLIMFHRTP]P..P.....	NNNKMRPHLPPLSSG	536	550	YHR102W	KIC1
[FPLWA]..[WYLMFHP].[AVLIMFHPP]P..P.....	LNCLQIPHLPPSVQW	145	159	YHR105W	YPT35
...[RK].[AVLIMFHRTP]P..P.....	ELVKRFPGHPPLRYS	110	124	YHR108W	GGA2
[FPLWA]..[WYLMFHP].[AVLIMFHPP]P..P.....	FDMLLLPFIPSIDDG	354	368	YHR109W	CTM1
...R..[FLIYM].[FLIYM]P.....	QTIRSRYEIPENIQY	123	137	YHR109W	CTM1
...PPPP...	CYRCFYPTPPPPNAV	208	222	YHR111W	UBA4
...PPPP...	CFYPTPPPPNAVTSC	211	225	YHR111W	UBA4
...R..[FLIYM].[FLIYM]P.....	DLNRPLLKIPTLAIH	159	173	YHR113W	YHR113W
...PPPP...	DRGPAPEVPPPRRST	562	576	YHR114W	BZZ1
[FPLWA]..[WYLMFHP].[AVLIMFHPP]P..P.....	LKFPTLPEVPTFFAE	474	488	YHR117W	TOM71
...[KR][KR][AHPKRLG]P[PLV].......	LNKRKKPVMFARSAI	933	947	YHR119W	SET1
[FPLWA]..[WYLMFHP].[AVLIMFHPP]P..P.....	LHGYGHPNPPNEKSR	371	385	YHR135C	YCK1
...[RK].[AVLIMFHRTP]P..P.....	KLRKFHPPEPYKGKG	184	198	YHR147C	MRPL6
...[KR][KR][AHPKRLG]P[PLV].......	ICRRRLPVIMHRLKM	106	120	YHR148W	IMP3
.[FPLWA].[WYLMFHP].[AVLIMFHPR]P..P.....	KLSLLPPTDPFRRKH	60	74	YHR148W	IMP3
...[KR][KR][AHPKRLG]P[PLV].......	GYVRKHPLQRSIYPL	274	288	YHR150W	PEX28
...R..[FLIYM].[FLIYM]P.....	LKSRKWFKLPVFTAG	380	394	YHR158C	KEL1
...R..[FLIYM].[FLIYM]P.....	PSDRITFTLPVLKDA	209	223	YHR163W	SOL3
......P.[ILMVPYAFTR]P.[RKW]...	QNPSSTPIVPNRLKT	155	169	YHR164C	DNA2
......P.[ILMVPYAFTR]P.[RKW]...	RDANDDPVIPYKLSK	1034	1048	YHR164C	DNA2
...PPPP...	MSGLPPPPPGFEEDS	1	15	YHR165C	PRP8
...PPPP...	EDSDLALPPPPPPPP	13	27	YHR165C	PRP8
...PPPP...	DLALPPPPPPPPGYE	16	30	YHR165C	PRP8
...PPPP...	LPPPPPPPPGYEIEE	19	33	YHR165C	PRP8
...PPPP...	VNEDTFLPPPPPPPS	43	57	YHR165C	PRP8
...PPPP...	DTFLPPPPPPPSNFE	46	60	YHR165C	PRP8
...PPPP...	LPPPPPPPSNFEINA	49	63	YHR165C	PRP8
...PPPP...	VDFTLPPPPPPPGLD	67	81	YHR165C	PRP8
...PPPP...	TLPPPPPPPGLDELE	70	84	YHR165C	PRP8
.[FPLWA].[WYLMFHP].[AVLIMFHPR]P..P.....	LALPPPPPPPPGYEI	17	31	YHR165C	PRP8
.[FPLWA].[WYLMFHP].[AVLIMFHPR]P..P.....	ALPPPPPPPPGYEIE	18	32	YHR165C	PRP8
.[FPLWA].[WYLMFHP].[AVLIMFHPR]P..P.....	TFLPPPPPPPSNFEI	47	61	YHR165C	PRP8
.[FPLWA].[WYLMFHP].[AVLIMFHPR]P..P.....	DFTLPPPPPPPGLDE	68	82	YHR165C	PRP8
[FPLWA]..[WYLMFHP].[AVLIMFHPP]P..P.....	FTLPPPPPPPGLDEL	69	83	YHR165C	PRP8
.[FPLWA].[WYLMFHP].[AVLIMFHPR]P..P.....	RASLQIPTIPSNSSD	358	372	YHR172W	SPC97
...R..[FLIYM].[FLIYM]P.....	KVVRASLQIPTIPSN	355	369	YHR172W	SPC97
......P.[ILMVPYAFTR]P.[RKW]...	PFEENVPLYPSRRNI	78	92	YHR176W	FMO
....P.R.A[VP].....	PGNIPVRTAVQLPLT	226	240	YHR177W	GON3
.....P.R.P.R...	AHCSYPGRAPRRTKK	47	61	YHR178W	STB5
......P.[ILMVPYAFTR]P.[RKW]...	FKTSSTPTTPERPKR	755	769	YHR182W	YHR182W
......P.[ILMVPYAFTR]P.[RKW]...	SSTPTTPERPKRKSG	758	772	YHR182W	YHR182W
...[RK].[AVLIMFHRTP]P..P.....	DTTKVTPIVPVPIHV	383	397	YHR188C	GPI16
...R..[FLIYM].[FLIYM]P.....	PALRSNLRIPSIAAA	27	41	YHR194W	MDM31
[FPLWA]..[WYLMFHP].[AVLIMFHPP]P..P.....	LNNWILPSTPHIQIL	534	548	YHR197W	RIX1
[FPLWA]..[WYLMFHP].[AVLIMFHPP]P..P.....	FELLCHPKVPVGMVY	603	617	YHR197W	RIX1
[FPLWA]..[WYLMFHP].[AVLIMFHPP]P..P.....	PKKMGPPQWPFSTQY	85	99	YHR199C	FMP34
...PPPP...	FSPPEFPPLSPLERS	376	390	YHR204W	MNL1
...[RK].[AVLIMFHRTP]P..P.....	HPEKLTPYTPMTGIG	261	275	YHR204W	MNL1
.[FPLWA].[WYLMFHP].[AVLIMFHPR]P..P.....	RWNFSPPEFPPLSPL	373	387	YHR204W	MNL1
...[KR][KR][AHPKRLG]P[PLV].......	EHLKKKPLYTHRSSS	276	290	YHR205W	SCH9
..[KRP]......P.[KR]P..	ALKQKKIPPPFKPHL	678	692	YHR205W	SCH9
..[KRP]......P.[KR]P..	KLKITRNPNPSKPRP	27	41	YHR208W	BAT1
...PPPP...	ASPQPASVPPPQNGP	70	84	YHR214C-B	YHR214C-B
...PPPP...	SVPPPQNGPYPQQCM	76	90	YHR214C-B	YHR214C-B
...PPPP...	DLPLPDLPPEPPTEL	1168	1182	YHR214C-B	YHR214C-B
.[FPLWA].[WYLMFHP].[AVLIMFHPR]P..P.....	IADLPLPDLPPEPPT	1166	1180	YHR214C-B	YHR214C-B
.[FPLWA].[WYLMFHP].[AVLIMFHPR]P..P.....	LPLPDLPPEPPTELS	1169	1183	YHR214C-B	YHR214C-B
.[FPLWA].[WYLMFHP].[AVLIMFHPR]P..P.....	IPKLNVPLNPKGRKL	1531	1545	YHR214C-B	YHR214C-B
...PPPP...	ASPQPASVPPPQNGP	70	84	YHR214C-C	YHR214C-C
...PPPP...	SVPPPQNGPYPQQCM	76	90	YHR214C-C	YHR214C-C
...[RK].[AVLIMFHRTP]P..P.....	RVTRKRPREPKSTND	271	285	YHR218W	YHR218W
..[KRP]......P.[KR]P..	TERLKRDLCPRKPTE	165	179	YHR218W	YHR218W

Table S6

Motif	Motif Match	Start	End	ORF	Common name
...[KR][KR][AHPKRLG]P[PLV].......	KRGRKLPPPSSDLKK	889	903	YIL002C	INP51
.[FPLWA].[WYLMFHP].[AVLIMFHPR]P..P.....	KLIHEHPCGPLKKLF	141	155	YIL002C	INP51
....RP[AVLIMFHRKTP][AVLIMFHRTP][AVLIMFHRTP]..[LP]...	KNEDRPLPVLLLREG	180	194	YIL007C	NAS2
...[RK].[AVLIMFHRTP]P..P.....	DEVKLHPPEPKDLAC	252	266	YIL009W	FAA3
...[KR][KR][AHPKRLG]P[PLV].......	VRVKKRPLIRPLNSS	24	38	YIL009W	FAA3
......P.[ILMVPYAFTR]P.[RKW]...	LAPISTPEVPKKKIK	25	39	YIL010W	DOT5
...R..[FLIYM].[FLIYM]P.....	LYLRYHYMIPGDRKL	267	281	YIL017C	VID28
......P.[ILMVPYAFTR]P.[RKW]...	LQDSPFPSAPLRKLE	89	103	YIL034C	CAP2
[FPLWA]..[WYLMFHP].[AVLIMFHPP]P..P.....	LTNFEVPSLPTFHKR	448	462	YIL037C	PRM2
...[RK].[AVLIMFHRTP]P..P.....	RRSKSLPITPKSIFN	158	172	YIL045W	PIG2
...[RK].[AVLIMFHRTP]P..P.....	KDNRLLPSWPKRGFS	207	221	YIL047C	SYG1
...R..[FLIYM].[FLIYM]P.....	LKGRNGLGFPINEQD	175	189	YIL053W	RHR2
...PPPP...	VTPQPPSASTPPSQF	175	189	YIL055C	YIL055C
...PPPP...	PTPVPVPVGVPPLAP	208	222	YIL055C	YIL055C
...PPPP...	PVGVPPLAPPPHGPF	214	228	YIL055C	YIL055C
...PPPP...	VPPLAPPPHGPFSTS	217	231	YIL055C	YIL055C
[FPLWA]..[WYLMFHP].[AVLIMFHPP]P..P.....	PSMPSIPLVPQEKDD	552	566	YIL056W	YIL056W
...R..[FLIYM].[FLIYM]P.....	TILRTEFGYPVSCSK	69	83	YIL056W	YIL056W
......P.[ILMVPYAFTR]P.[RKW]...	AERNYAPRLPRRETS	228	242	YIL061C	SNP1
......P.[ILMVPYAFTR]P.[RKW]...	EPSDEPPSFPKRWGW	82	96	YIL067C	YIL067C
[FPLWA]..[WYLMFHP].[AVLIMFHPP]P..P.....	FVTLHVPATPETEKM	253	267	YIL074C	SER33
......P.[ILMVPYAFTR]P.[RKW]...	DKVASMPRVPLKIVL	65	79	YIL078W	THS1
...[KR][KR][AHPKRLG]P[PLV].......	NNQRRKPLLLNIGDH	1398	1412	YIL080W	YIL080W
....RP[AVLIMFHRKTP][AVLIMFHRTP][AVLIMFHRTP]..[LP]...	IKSHRPRLHGLLQPL	1174	1188	YIL080W	YIL080W
...[KR][KR][AHPKRLG]P[PLV].......	NNQRRKPLLLNIGDH	1398	1412	YIL082W-A	YIL082W-A
....RP[AVLIMFHRKTP][AVLIMFHRTP][AVLIMFHRTP]..[LP]...	IKSHRPRLHGLLQPL	1174	1188	YIL082W-A	YIL082W-A
......P.[ILMVPYAFTR]P.[RKW]...	RELTGQPPAPFRLRS	312	326	YIL084C	SDS3
..[KRP]....P...[KR]P..	ISRWVNFPWDAKPKL	295	309	YIL085C	KTR7
....P.R.A[VP].....	LLFHPLRIAVNNVII	284	298	YIL088C	AVT7
..[KRP]....P...[KR]P..	QFRDDSLPPKSKPKS	357	371	YIL091C	YIL091C
...PPPP...	DKSRPPRPPPKPLHL	745	759	YIL095W	PRK1
...PPPP...	RPPRPPPKPLHLRTE	748	762	YIL095W	PRK1
...[RK].[AVLIMFHRTP]P..P.....	GKDKSRPPRPPPKPL	743	757	YIL095W	PRK1
......P.[ILMVPYAFTR]P.[RKW]...	KDKSRPPRPPPKPLH	744	758	YIL095W	PRK1
...PPPP...	NVPSSPIAPAPPTFP	577	591	YIL101C	XBP1
...PPPP...	SSPIAPAPPTFPQPY	580	594	YIL101C	XBP1
...PPPP...	IAPAPPTFPQPYGDD	583	597	YIL101C	XBP1
[FPLWA]..[WYLMFHP].[AVLIMFHPP]P..P.....	PRHYNVPSSPIAPAP	573	587	YIL101C	XBP1
[FPLWA]..[WYLMFHP].[AVLIMFHPP]P..P.....	PSSPIAPAPPTFPQP	579	593	YIL101C	XBP1
[FPLWA]..[WYLMFHP].[AVLIMFHPP]P..P.....	PIAPAPPTFPQPYGD	582	596	YIL101C	XBP1
...R..[FLIYM].[FLIYM]P.....	NAKRIALQMPEGLLI	91	105	YIL103W	YIL103W
...R..[FLIYM].[FLIYM]P.....	TGIRRALSYPLHRNY	371	385	YIL104C	SHQ1
......P.[ILMVPYAFTR]P.[RKW]...	DPNFLLPNLPMRTFK	441	455	YIL105C	LIT2
....P.R.A[VP].....	VLTTPKRHAPPPEQL	82	96	YIL106W	MOB1
....RP[AVLIMFHRKTP][AVLIMFHRTP][AVLIMFHRTP]..[LP]...	HHKRRPTTIDVPGLT	151	165	YIL107C	PFK26
......P.[ILMVPYAFTR]P.[RKW]...	DAPASKPSVPPRNYF	574	588	YIL108W	YIL108W
...PPPP...	LTLPPPPLVIPPERM	154	168	YIL109C	SEC24
.[FPLWA].[WYLMFHP].[AVLIMFHPR]P..P.....	PAQFMPPQDPAAAGM	26	40	YIL109C	SEC24
......P.[ILMVPYAFTR]P.[RKW]...	LKSVENPFLPAKEER	907	921	YIL115C	NUP159
..[KRP]....P...[KR]P..	TDPASAKPVFGKPAF	496	510	YIL115C	NUP159
...R..[FLIYM].[FLIYM]P.....	DWLRERIEIPEPYQY	226	240	YIL125W	KGD1
....RP[AVLIMFHRKTP][AVLIMFHRTP][AVLIMFHRTP]..[LP]...	HKVLRPFLLRRLKKE	687	701	YIL126W	STH1
...[RK].[AVLIMFHRTP]P..P.....	LVTKERPRFPEDDNR	689	703	YIL129C	TAO3
...[RK].[AVLIMFHRTP]P..P.....	VNFRRIPTGPDSPPT	137	151	YIL131C	FKH1
[FPLWA]..[WYLMFHP].[AVLIMFHPP]P..P.....	FQKLVPPTLPENVRI	51	65	YIL132C	CSM2
...R..[FLIYM].[FLIYM]P.....	ASIRTLLEFPKEQLL	157	171	YIL132C	CSM2
....RP[AS]....Y...	EPISRPSHIHEYKIT	162	176	YIL143C	SSL2
...[KR][KR][AHPKRLG]P[PLV].......	SDIRRAPVVAASPPP	179	193	YIL151C	YIL151C
.[FPLWA].[WYLMFHP].[AVLIMFHPR]P..P.....	IPTMDVPKSPYYVNR	117	131	YIL151C	YIL151C
...PPPP...	TSMPPPGVPPSGNNI	355	369	YIL153W	RRD1
...[RK].[AVLIMFHRTP]P..P.....	IPLKQVPENPMPTEA	375	389	YIL155C	GUT2
...R..[FLIYM].[FLIYM]P.....	SDGRVMFFLPWQGKV	354	368	YIL155C	GUT2
....RP[AVLIMFHRKTP][AVLIMFHRTP][AVLIMFHRTP]..[LP]...	SQGDRPLVHNDPSYM	28	42	YIL155C	GUT2
...PPPP...	PIRLRKRPPPPPPVS	508	522	YIL156W	UBP7
...PPPP...	LRKRPPPPPPVSMPT	511	525	YIL156W	UBP7
...PPPP...	RPPPPPPVSMPTTPE	514	528	YIL156W	UBP7
...PPPP...	MPTTPEIPPPLPPKI	523	537	YIL156W	UBP7
...PPPP...	TPEIPPPLPPKIMVH	526	540	YIL156W	UBP7
...[RK].[AVLIMFHRTP]P..P.....	IRLRKRPPPPPPVSM	509	523	YIL156W	UBP7
...[RK].[AVLIMFHRTP]P..P.....	RLRKRPPPPPPVSMP	510	524	YIL156W	UBP7
......P.[ILMVPYAFTR]P.[RKW]...	ESSTKVPEPPSWKPP	491	505	YIL156W	UBP7
......P.[ILMVPYAFTR]P.[RKW]...	PPSWKPPDLPIRLRK	499	513	YIL156W	UBP7

Table S6

Motif	Motif Match	Start	End	ORF	Common name
......P.[ILMVPYAFTR]P.[RKW]...	TTPEIPPPLPPKIMV	525	539	YIL156W	UBP7
......P.[ILMVPYAFTR]P.[RKW]...	SSISRKPPIPAKQHV	544	558	YIL156W	UBP7
..[KRP]......P.[KR]P..	REPERKTSVPVRPSV	222	236	YIL156W	UBP7
...R..[FLIYM].[FLIYM]P.....	NQLRPDLKIPDDQQD	689	703	YIL156W	UBP7
...PPPP...	VKLPQLPPPPPPPPP	763	777	YIL159W	BNR1
...PPPP...	PQLPPPPPPPPPPPL	766	780	YIL159W	BNR1
...PPPP...	PPPPPPPPPPPLPQS	769	783	YIL159W	BNR1
...PPPP...	PPPPPPPPLPQSLLT	772	786	YIL159W	BNR1
...PPPP...	SCIAAPAPPPLPDLF	796	810	YIL159W	BNR1
...PPPP...	AAPAPPPLPDLFKTK	799	813	YIL159W	BNR1
...PPPP...	TCGAVPPPPPPPPLP	814	828	YIL159W	BNR1
...PPPP...	AVPPPPPPPPLPESL	817	831	YIL159W	BNR1
...PPPP...	PPPPPPPLPESLSMN	820	834	YIL159W	BNR1
...PPPP...	DLVTPPAPPLPNGLL	841	855	YIL159W	BNR1
.[FPLWA].[WYLMFHP].[AVLIMFHPR]P..P.....	LPQLPPPPPPPPPPP	765	779	YIL159W	BNR1
.[FPLWA].[WYLMFHP].[AVLIMFHPR]P..P.....	QLPPPPPPPPPPPLP	767	781	YIL159W	BNR1
.[FPLWA].[WYLMFHP].[AVLIMFHPR]P..P.....	LPPPPPPPPPPPLPQ	768	782	YIL159W	BNR1
.[FPLWA].[WYLMFHP].[AVLIMFHPR]P..P.....	PPPPPPPPPPPLPQSL	770	784	YIL159W	BNR1
.[FPLWA].[WYLMFHP].[AVLIMFHPR]P..P.....	IAAPAPPPLPDLFKT	798	812	YIL159W	BNR1
.[FPLWA].[WYLMFHP].[AVLIMFHPR]P..P.....	GAVPPPPPPPPLPES	816	830	YIL159W	BNR1
.[FPLWA].[WYLMFHP].[AVLIMFHPR]P..P.....	PPPPPPPPLPESLSM	819	833	YIL159W	BNR1
[FPLWA]..[WYLMFHP].[AVLIMFHPP]P..P.....	LVTPPAPPLPNGLLS	842	856	YIL159W	BNR1
......P.[ILMVPYAFTR]P.[RKW]...	NIPLDYPKAPIKLTM	157	171	YIL161W	YIL161W
....RP[AVLIMFHRKTP][AVLIMFHRTP][AVLIMFHRTP]..[LP]...	ETSDRPLVHFTPNKG	24	38	YIL162W	SUC2
...[RK].[AVLIMFHRTP]P..P.....	TGKRKLPGWPSADDN	35	49	YIL165C	YIL165C
......P.[ILMVPYAFTR]P.[RKW]...	EYDLIMPIYPYRLVS	522	536	YIL173W	VTH1
...[RK].[AVLIMFHRTP]P..P.....	RVTRKRPREPKSTND	628	642	YIL177C	YIL177C
......P.[ILMVPYAFTR]P.[RKW]...	VCKLRSPNTPRRLRK	309	323	YIL177C	YIL177C
..[KRP]......P.[KR]P..	TERLKRDLCPRKPIE	229	243	YIL177C	YIL177C
...PPPP...	PDTFPYYPPYPPNQS	172	186	YIR001C	SGN1
.[FPLWA].[WYLMFHP].[AVLIMFHPR]P..P.....	TFPYYPPYPPNQSPN	174	188	YIR001C	SGN1
...PPPP...	ERPKRRAPPPVPKKP	382	396	YIR003W	YIR003W
...PPPP...	KRRAPPPVPKKPSSR	385	399	YIR003W	YIR003W
...[RK].[AVLIMFHRTP]P..P.....	PKRRAPPPVPKKPSS	384	398	YIR003W	YIR003W
......P.[ILMVPYAFTR]P.[RKW]...	MPSEVTPKVPERPSR	1	15	YIR003W	YIR003W
......P.[ILMVPYAFTR]P.[RKW]...	EVTPKVPERPSRRKT	4	18	YIR003W	YIR003W
......P.[ILMVPYAFTR]P.[RKW]...	PTPAGTPNVPTRRPI	39	53	YIR003W	YIR003W
...[KR][KR][AHPKRLG]P[PLV].......	RPKRRAPPPVPKKPS	383	397	YIR003W	YIR003W
.[FPLWA].[WYLMFHP].[AVLIMFHPR]P..P.....	RAPPPVPKKPSSRIA	387	401	YIR003W	YIR003W
....P.R.A[VP].....	PSERPKRRAPPPVPK	380	394	YIR003W	YIR003W
....RP[AVLIMFHRKTP][AVLIMFHRTP][AVLIMFHRTP]..[LP]...	IPSERPKRRAPPPVP	379	393	YIR003W	YIR003W
...PPPP...	GTMPPPNPAQQPQLQ	127	141	YIR006C	PAN1
...PPPP...	SSPIPIAPIPPSVTQ	1339	1353	YIR006C	PAN1
...PPPP...	VTQEPPVPLAPPLPA	1351	1365	YIR006C	PAN1
...PPPP...	EPPVPLAPPLPAVDG	1354	1368	YIR006C	PAN1
...PPPP...	ALAGGVLPPPPPLPT	1393	1407	YIR006C	PAN1
...PPPP...	GGVLPPPPPLPTQQA	1396	1410	YIR006C	PAN1
...PPPP...	LPPPPPLPTQQASTS	1399	1413	YIR006C	PAN1
...PPPP...	IPSIPPAGIPPPPPL	1465	1479	YIR006C	PAN1
.[FPLWA].[WYLMFHP].[AVLIMFHPR]P..P.....	SPIPIAPIPPSVTQE	1340	1354	YIR006C	PAN1
.[FPLWA].[WYLMFHP].[AVLIMFHPR]P..P.....	PPVPLAPPLPAVDGF	1355	1369	YIR006C	PAN1
[FPLWA]..[WYLMFHP].[AVLIMFHPP]P..P.....	FQEPPIPSAPAIATA	1369	1383	YIR006C	PAN1
...PPPP...	IQPPVMKPPPNLQNS	418	432	YIR007W	YIR007W
...[RK].[AVLIMFHRTP]P..P.....	AKSKSRPNTPGSDTS	740	754	YIR007W	YIR007W
.[FPLWA].[WYLMFHP].[AVLIMFHPR]P..P.....	RLALKRPYHPHLARS	202	216	YIR008C	PRI1
...[RK].[AVLIMFHRTP]P..P.....	ERRKALPKEPKKLLP	419	433	YIR010W	DSN1
[FPLWA]..[WYLMFHP].[AVLIMFHPP]P..P.....	ASAMPAPYMPYYYYY	140	154	YIR010W	DSN1
..[KRP]......P.[KR]P..	GLPRNADSQPARPRT	33	47	YIR013C	GAT4
...[RK].[AVLIMFHRTP]P..P.....	KNWKLPPRLPHRAAQ	26	40	YIR018W	YAP5
.[FPLWA].[WYLMFHP].[AVLIMFHPR]P..P.....	TLAPSAPVTPATNAV	1174	1188	YIR019C	MUC1
...[KR][KR][AHPKRLG]P[PLV].......	YDTKKAPLRTFLVSR	40	54	YIR021W	MRS1
......P.[ILMVPYAFTR]P.[RKW]...	SNNTPLPFAPNKSSK	843	857	YIR023W	DAL81
.[FPLWA].[WYLMFHP].[AVLIMFHPR]P..P.....	FFKLIHPFLPILHER	323	337	YIR023W	DAL81
....P.R.A[VP].....	FKKKPQRGAVFAHCQ	104	118	YIR026C	YVH1
......P.[ILMVPYAFTR]P.[RKW]...	AENLIQPTAPIRDPT	45	59	YIR029W	DAL2
.....P.R.P.R...	IQPTAPIRDPTRFVH	49	63	YIR029W	DAL2
......P.[ILMVPYAFTR]P.[RKW]...	IYESLDPDRPEKDCV	168	182	YIR032C	DAL3
..[KRP]....P...[KR]P..	IRRYLRVPNELKPSQ	4	18	YJL004C	SYS1
......P.[ILMVPYAFTR]P.[RKW]...	ELDPMSPGPPSKKDS	492	506	YJL005W	CYR1
..[KRP]....P...[KR]P..	TTPTIETPISCKPSL	370	384	YJL005W	CYR1
[FPLWA]..[WYLMFHP].[AVLIMFHPP]P..P.....	FKFLFHPVTPSHFTP	769	783	YJL005W	CYR1
[FPLWA]..[WYLMFHP].[AVLIMFHPP]P..P.....	AGGPRAPQGPRPGNW	550	564	YJL008C	CCT8

Table S6

Motif	Motif Match	Start	End	ORF	Common name
...[KR][KR][AHPKRLG]P[PLV].......	VDIRKPPLPTNIEIT	483	497	YJL012C	VTC4
...R..[FLIYM].[FLIYM]P.....	FYNRTAFQLPGDARV	358	372	YJL012C	VTC4
.[FPLWA].[WYLMFHP].[AVLIMFHPR]P..P.....	SLMFGLPKLPSVDYF	420	434	YJL016W	YJL016W
...R..[FLIYM].[FLIYM]P.....	VSSRSFLELPPELFS	34	48	YJL016W	YJL016W
...R..[FLIYM].[FLIYM]P.....	SRIRQQMYLPPWFIQ	568	582	YJL019W	MPS3
...PPPP...	KVPPHPVPSAPSAPP	688	702	YJL020C	BBC1
...PPPP...	PHPVPSAPSAPPVPS	691	705	YJL020C	BBC1
...PPPP...	APSVPSAPPVPPAPP	706	720	YJL020C	BBC1
...PPPP...	VPSAPPVPPAPPALS	709	723	YJL020C	BBC1
...PPPP...	APPVPPAPPALSAPS	712	726	YJL020C	BBC1
...PPPP...	ALSAPSVPPVPPVPP	721	735	YJL020C	BBC1
...PPPP...	APSVPPVPPVPPVSS	724	738	YJL020C	BBC1
...PPPP...	VPPVPPVPPVSSAPP	727	741	YJL020C	BBC1
...PPPP...	VPPVPPVSSAPPALS	730	744	YJL020C	BBC1
...PPPP...	ALSAPSIPPVPPTPP	742	756	YJL020C	BBC1
...PPPP...	APSIPPVPPTPPAPP	745	759	YJL020C	BBC1
...PPPP...	IPPVPPTPPAPPAPP	748	762	YJL020C	BBC1
...PPPP...	VPPTPPAPPAPPAPL	751	765	YJL020C	BBC1
...PPPP...	TPPAPPAPPAPLALP	754	768	YJL020C	BBC1
...PPPP...	APPAPPAPLALPKHN	757	771	YJL020C	BBC1
...PPPP...	YHPMPNTAPPLPRAP	790	804	YJL020C	BBC1
...PPPP...	TAPPLPRAPPVPPAT	796	810	YJL020C	BBC1
...PPPP...	PLPRAPPVPPATFEF	799	813	YJL020C	BBC1
...[RK].[AVLIMFHRTP]P..P.....	ELPRAIPVMPFVDPS	472	486	YJL020C	BBC1
.[FPLWA].[WYLMFHP].[AVLIMFHPR]P..P.....	VPPHPVPSAPSAPPV	689	703	YJL020C	BBC1
.[FPLWA].[WYLMFHP].[AVLIMFHPR]P..P.....	HPVPSAPSAPPVPSA	692	706	YJL020C	BBC1
.[FPLWA].[WYLMFHP].[AVLIMFHPR]P..P.....	PPVPSAPSVPSAPPV	701	715	YJL020C	BBC1
.[FPLWA].[WYLMFHP].[AVLIMFHPR]P..P.....	SAPPVPPAPPALSAP	711	725	YJL020C	BBC1
.[FPLWA].[WYLMFHP].[AVLIMFHPR]P..P.....	PPALSAPSVPPVPPV	719	733	YJL020C	BBC1
.[FPLWA].[WYLMFHP].[AVLIMFHPR]P..P.....	PPALSAPSIPPVPPT	740	754	YJL020C	BBC1
.[FPLWA].[WYLMFHP].[AVLIMFHPR]P..P.....	PPTPPAPPAPPAPLA	752	766	YJL020C	BBC1
.[FPLWA].[WYLMFHP].[AVLIMFHPR]P..P.....	PPAPPAPPAPLALPK	755	769	YJL020C	BBC1
.[FPLWA].[WYLMFHP].[AVLIMFHPR]P..P.....	PPLPRAPPVPPATFE	798	812	YJL020C	BBC1
[FPLWA]..[WYLMFHP].[AVLIMFHPP]P..P.....	PKYYVPPGIPTNDTS	200	214	YJL020C	BBC1
[FPLWA]..[WYLMFHP].[AVLIMFHPP]P..P.....	PSAPSAPPVPSAPSV	695	709	YJL020C	BBC1
[FPLWA]..[WYLMFHP].[AVLIMFHPP]P..P.....	PSAPPVPSAPSVPSA	698	712	YJL020C	BBC1
[FPLWA]..[WYLMFHP].[AVLIMFHPP]P..P.....	PSAPSVPSAPPVPPA	704	718	YJL020C	BBC1
[FPLWA]..[WYLMFHP].[AVLIMFHPP]P..P.....	PSVPSAPPVPPAPPA	707	721	YJL020C	BBC1
[FPLWA]..[WYLMFHP].[AVLIMFHPP]P..P.....	PSAPPVPPAPPALSA	710	724	YJL020C	BBC1
[FPLWA]..[WYLMFHP].[AVLIMFHPP]P..P.....	LSAPSVPPVPPVPPV	722	736	YJL020C	BBC1
[FPLWA]..[WYLMFHP].[AVLIMFHPP]P..P.....	PSVPPVPPVPPVSSA	725	739	YJL020C	BBC1
[FPLWA]..[WYLMFHP].[AVLIMFHPP]P..P.....	LSAPSIPPVPPTPPA	743	757	YJL020C	BBC1
[FPLWA]..[WYLMFHP].[AVLIMFHPP]P..P.....	PSIPPVPPTPPAPPA	746	760	YJL020C	BBC1
[FPLWA]..[WYLMFHP].[AVLIMFHPP]P..P.....	PVPPTPPAPPAPPAP	750	764	YJL020C	BBC1
[FPLWA]..[WYLMFHP].[AVLIMFHPP]P..P.....	PTPPAPPAPPAPLAL	753	767	YJL020C	BBC1
[FPLWA]..[WYLMFHP].[AVLIMFHPP]P..P.....	AYSLGLPGAPKIKMK	478	492	YJL033W	HCA4
......P.[ILMVPYAFTR]P.[RKW]...	FPTCIIPPLPDKKVF	93	107	YJL036W	SNX4
..[KRP]......P.[KR]P..	KAKDKARGNPEKPPY	12	26	YJL036W	SNX4
..[KRP]......P.[KR]P..	SQPEPTTNEPAKPAL	252	266	YJL041W	NSP1
..[KRP]....P...[KR]P..	SLKLNSKPVELKPVS	617	631	YJL041W	NSP1
...PPPP...	KGRPIPPHPDAPKLP	331	345	YJL042W	MHP1
...PPPP...	PIPPHPDAPKLPSAF	334	348	YJL042W	MHP1
...[RK].[AVLIMFHRTP]P..P.....	FDAKGRPIPPHPDAP	328	342	YJL042W	MHP1
...[RK].[AVLIMFHRTP]P..P.....	AKGRPIPPHPDAPKL	330	344	YJL042W	MHP1
.[FPLWA].[WYLMFHP].[AVLIMFHPR]P..P.....	RPIPPHPDAPKLPSA	333	347	YJL042W	MHP1
.[FPLWA].[WYLMFHP].[AVLIMFHPR]P..P.....	PPHPDAPKLPSAFRK	336	350	YJL042W	MHP1
...R..[FLIYM].[FLIYM]P.....	CHLREILPIPATLKQ	646	660	YJL042W	MHP1
......P.[ILMVPYAFTR]P.[RKW]...	IADTLQPGLPHKPLA	458	472	YJL045W	YJL045W
.[FPLWA].[WYLMFHP].[AVLIMFHPR]P..P.....	TLQPGLPHKPLASNI	461	475	YJL045W	YJL045W
..[KRP]......P.[KR]P..	LGPISTSKCPKKPSH	234	248	YJL051W	YJL051W
......P.[ILMVPYAFTR]P.[RKW]...	HLESKPPQLPPKCSS	340	354	YJL056C	ZAP1
...R..[FLIYM].[FLIYM]P.....	MCFRDSIVLPCYENF	466	480	YJL058C	BIT61
[FPLWA]..[WYLMFHP].[AVLIMFHPP]P..P.....	ADVPIPPIDPQELEA	60	74	YJL062W-A	YJL062W-A
...[RK].[AVLIMFHRTP]P..P.....	VPSKAIPPLPQLDFL	99	113	YJL066C	MPM1
...R..[FLIYM].[FLIYM]P.....	TPHRHGYYYPSYFTD	195	209	YJL070C	YJL070C
.[FPLWA].[WYLMFHP].[AVLIMFHPR]P..P.....	RPMFVIPPSPTHVNI	84	98	YJL071W	ARG2
...R..[FLIYM].[FLIYM]P.....	LGLRPMFVIPPSPTH	81	95	YJL071W	ARG2
....RP[AVLIMFHRKTP][AVLIMFHRTP][AVLIMFHRTP]..[LP]...	MLGLRPMFVIPPSPT	80	94	YJL071W	ARG2
...PPPP...	LPPPTQPQSPPIRIS	244	258	YJL076W	NET1
...[KR][KR][AHPKRLG]P[PLV].......	RIAKKRPPTGTTTTT	184	198	YJL076W	NET1
...[KR][KR][AHPKRLG]P[PLV].......	DINRKPPVTTPRITS	348	362	YJL076W	NET1
......PR.P.R...	RVVVNTPREPVRSSS	1012	1026	YJL076W	NET1

Table S6

Motif	Motif Match	Start	End	ORF	Common name
...R..[FLIYM].[FLIYM]P.....	KLARAPIEIPLEKVC	1053	1067	YJL080C	SCP160
...[RK].[AVLIMFHRTP]P..P.....	SYLRLLPWWPSLANE	457	471	YJL083W	YJL083W
......P.[ILMVPYAFTR]P.[RKW]...	ILAVDFPIFPRRFAK	141	155	YJL091C	GWT1
......P.[ILMVPYAFTR]P.[RKW]...	PEFWISPDTPLKFPL	633	647	YJL093C	TOK1
...R..[FLIYM].[FLIYM]P.....	KRKRFNLLLPKPYLT	720	734	YJL094C	KHA1
...PPPP...	VNIPSPSSSPPPIPK	472	486	YJL095W	BCK1
...PPPP...	PSPSSSPPPIPKTAN	475	489	YJL095W	BCK1
...[RK].[AVLIMFHRTP]P..P.....	APKREAPKPPANTSP	803	817	YJL095W	BCK1
...[RK].[AVLIMFHRTP]P..P.....	GKSKSAPPIPEDTLP	1393	1407	YJL095W	BCK1
......P.[ILMVPYAFTR]P.[RKW]...	ASSRTEPSTPSRPVP	1075	1089	YJL095W	BCK1
....P.R.A[VP].....	PELAPKREAPKPPAN	800	814	YJL095W	BCK1
....RP[AS]....Y...	KMTFRPSPEVVYQNL	1025	1039	YJL095W	BCK1
...R..[FLIYM].[FLIYM]P.....	TVKRDSFNIPILVMG	595	609	YJL103C	YJL103C
[FPLWA]..[WYLMFHP].[AVLIMFHPP]P..P.....	PKVLFHPHWPLYIDS	402	416	YJL105W	SET4
...R..[FLIYM].[FLIYM]P.....	HRRRRVFSIPSLKSI	159	173	YJL106W	IME2
...[RK].[AVLIMFHRTP]P..P.....	KSDKNAPSVPQVDAS	13	27	YJL117W	PHO86
....RP[AVLIMFHRKTP][AVLIMFHRTP][AVLIMFHRTP]..[LP]...	RRRRRPHRIERPLSN	151	165	YJL118W	YJL118W
....RP[AVLIMFHRKTP][AVLIMFHRTP][AVLIMFHRTP]..[LP]...	NSINRPKLTSNLHHK	5	19	YJL122W	YJL122W
......P.[ILMVPYAFTR]P.[RKW]...	GIFDLKPAYPGRSAH	169	183	YJL127C	SPT10
[FPLWA]..[WYLMFHP].[AVLIMFHPP]P..P.....	ATMYPIPAYPDLLPL	54	68	YJL127C	SPT10
...[RK].[AVLIMFHRTP]P..P.....	IVNKPLPPLPVAGSS	90	104	YJL128C	PBS2
...[RK].[AVLIMFHRTP]P..P.....	NPNRRAPRRPLSTQH	181	195	YJL128C	PBS2
..[KRP]......P.[KR]P..	PRRPLSTQHPTRPNV	187	201	YJL128C	PBS2
....P.R.A[VP].....	QLLNPNRRAPRRPLS	178	192	YJL128C	PBS2
.[FPLWA].[WYLMFHP].[AVLIMFHPR]P..P.....	KAIMGLPLTPYPVEK	1287	1301	YJL130C	URA2
...[RK].[AVLIMFHRTP]P..P.....	NSTRPIPAIPMDLPD	9	23	YJL133W	MRS3
.[FPLWA].[WYLMFHP].[AVLIMFHPR]P..P.....	YWCLPRPRAPPLHRI	130	144	YJL134W	LCB3
...PPPP...	VVPPARAPAPNPFQY	130	144	YJL141C	YAK1
...PPPP...	AYPPSTSPPLQPPFK	241	255	YJL141C	YAK1
.[FPLWA].[WYLMFHP].[AVLIMFHPR]P..P.....	ELFLGIPIFPGASEY	562	576	YJL141C	YAK1
.[FPLWA].[WYLMFHP].[AVLIMFHPR]P..P.....	QAKPMAPPLPEAPSS	139	153	YJL143W	TIM17
[FPLWA]..[WYLMFHP].[AVLIMFHPP]P..P.....	PMAPPLPEAPSSQPL	142	156	YJL143W	TIM17
...[RK].[AVLIMFHRTP]P..P.....	FVKKIRPGNPLTKLS	280	294	YJL149W	YJL149W
...R..[FLIYM].[FLIYM]P.....	NFLRLLIGLPSQNEL	512	526	YJL153C	INO1
...[RK].[AVLIMFHRTP]P..P.....	GPLKYAPHLPFSKSE	590	604	YJL176C	SWI3
[FPLWA]..[WYLMFHP].[AVLIMFHPP]P..P.....	ANTPSIPTNPVDNEN	126	140	YJL176C	SWI3
......P.[ILMVPYAFTR]P.[RKW]...	PAKLRIPETPVKKSP	188	202	YJL187C	SWE1
......P.[ILMVPYAFTR]P.[RKW]...	NLFDDAPATPPRPLK	15	29	YJL194W	CDC6
......P.[ILMVPYAFTR]P.[RKW]...	DDAPATPPRPLKRKK	18	32	YJL194W	CDC6
......P.[ILMVPYAFTR]P.[RKW]...	PSFNAKPPLPIKAVT	437	451	YJL201W	ECM25
.[FPLWA].[WYLMFHP].[AVLIMFHPR]P..P.....	RLVMKLPQAPYSLVI	54	68	YJL201W	ECM25
...R..[FLIYM].[FLIYM]P.....	LMDRLVMKLPQAPYS	51	65	YJL201W	ECM25
...PPPP...	LQLPDDYNPPPPSRN	664	678	YJL204C	RCY1
...PPPP...	YNPPPPSRNSPIRDI	670	684	YJL204C	RCY1
......P.[ILMVPYAFTR]P.[RKW]...	LPDDYNPPPPSRNSP	666	680	YJL204C	RCY1
...[KR][KR][AHPKRLG]P[PLV].......	YSLRRKPLEEDSRTQ	420	434	YJL206C	YJL206C
...R..[FLIYM].[FLIYM]P.....	TSLRSILNLPQGEAD	673	687	YJL206C	YJL206C
....RP[AVLIMFHRKTP][AVLIMFHRTP][AVLIMFHRTP]..[LP]...	RFYHRPTIISILDSI	202	216	YJL206C	YJL206C
.[FPLWA].[WYLMFHP].[AVLIMFHPR]P..P.....	VAAFVLPNEPISNET	249	263	YJL208C	NUC1
......P.[ILMVPYAFTR]P.[RKW]...	EYDLIMPIYPYRLVS	522	536	YJL222W	VTH2
...[RK].[AVLIMFHRTP]P..P.....	RVTRKRPREPKSTND	628	642	YJL225C	YJL225C
......P.[ILMVPYAFTR]P.[RKW]...	VCKLRSPNTPRRLRK	309	323	YJL225C	YJL225C
..[KRP]......P.[KR]P..	TERLKRDLCPRKPIE	229	243	YJL225C	YJL225C
..[KRP]....P...[KR]P..	IIPIAKTPLNARPIV	478	492	YJR001W	AVT1
...[RK].[AVLIMFHRTP]P..P.....	NKTKLLPQSPILNPN	604	618	YJR005W	APL1
...PPPP...	PTPLPQNPFPAPIAT	193	207	YJR006W	HYS2
...[RK].[AVLIMFHRTP]P..P.....	PSDKSLPQQPFHKSL	305	319	YJR006W	HYS2
..[KRP]......P.[KR]P..	VVKILRESNPPRPKQ	380	394	YJR010W	MET3
...R..[FLIYM].[FLIYM]P.....	NVYRYGYMMPVHNVI	441	455	YJR015W	YJR015W
....RP[AS]....Y...	AKVLRPANGLIYRIA	337	351	YJR015W	YJR015W
...[RK].[AVLIMFHRTP]P..P.....	ERAKKAPSLPEGQEI	377	391	YJR016C	ILV3
...[RK].[AVLIMFHRTP]P..P.....	EGPRGAPGMPEMLKP	464	478	YJR016C	ILV3
...[RK].[AVLIMFHRTP]P..P.....	FVTKYLPAAPVGSKG	25	39	YJR019C	TES1
...PPPP...	ASPQPASVPPPQNGP	70	84	YJR026W	YJR026W
...PPPP...	SVPPPQNGPYPQQCM	76	90	YJR026W	YJR026W
...PPPP...	ASPQPASVPPPQNGP	70	84	YJR027W	YJR027W
...PPPP...	SVPPPQNGPYPQQCM	76	90	YJR027W	YJR027W
.[FPLWA].[WYLMFHP].[AVLIMFHPR]P..P.....	IADLPLPDLPPESPT	1128	1142	YJR027W	YJR027W
.[FPLWA].[WYLMFHP].[AVLIMFHPR]P..P.....	IPKLNVPLNPKGRKL	1493	1507	YJR027W	YJR027W
.[FPLWA].[WYLMFHP].[AVLIMFHPR]P..P.....	IADLPLPDLPPESPT	1128	1142	YJR029W	YJR029W
.[FPLWA].[WYLMFHP].[AVLIMFHPR]P..P.....	IPKLNVPLNPKGRKL	1493	1507	YJR029W	YJR029W
...R..[FLIYM].[FLIYM]P.....	NHSRSLFSLPGNQQS	523	537	YJR030C	YJR030C

Table S6

Motif	Motif Match	Start	End	ORF	Common name
...[RK].[AVLIMFHRTP]P..P.....	EWRRPHPNIPDAKLN	270	284	YJR035W	RAD26
..[KRP]....P...[KR]P..	SQRSSDLPEWRRPHP	262	276	YJR035W	RAD26
..[KRP]......P.[KR]P..	TKRPATSTPPRKPSP	300	314	YJR043C	POL32
......P.[ILMVPYAFTR]P.[RKW]...	SNNIIKPYLPFKISK	66	80	YJR054W	YJR054W
......P.[ILMVPYAFTR]P.[RKW]...	KAYFREPDLPRKGLL	473	487	YJR054W	YJR054W
...[RK].[AVLIMFHRTP]P..P.....	RSLRKRPTSPSISGS	49	63	YJR059W	PTK2
..[KRP]....P...[KR]P..	YNPYGISPNHARPDT	184	198	YJR059W	PTK2
...[RK].[AVLIMFHRTP]P..P.....	DMDRNVPLAPNLNVN	22	36	YJR066W	TOR1
.....[GP]P.[IVL].P[FWY]...	IAYEVGPDIAPYVKQ	415	429	YJR066W	TOR1
......P.[ILMVPYAFTR]P.[RKW]...	HDLENYPCPPYKIII	124	138	YJR068W	RFC2
...R..[FLIYM].[FLIYM]P.....	LRERIAMTFPYDSHV	114	128	YJR075W	HOC1
...PPPP...	TLPNRKPNPPPNRSQ	184	198	YJR083C	ACF4
...PPPP...	STPTSGPPLLPPRNT	211	225	YJR083C	ACF4
......P.[ILMVPYAFTR]P.[RKW]...	TPTSGPPLLPPRNTM	212	226	YJR083C	ACF4
..[KRP]......P.[KR]P..	ESKSPRVTTPLKPKR	41	55	YJR083C	ACF4
...[RK].[AVLIMFHRTP]P..P.....	QLNRLLPNLPEEERK	139	153	YJR090C	GRR1
......P.[ILMVPYAFTR]P.[RKW]...	NNNSTRPQMPSRTRE	47	61	YJR090C	GRR1
...PPPP...	KEQCPFPLPPPNVNE	523	537	YJR091C	JSN1
...[RK].[AVLIMFHRTP]P..P.....	LDTKTIPELPFCMSS	118	132	YJR092W	BUD4
...[RK].[AVLIMFHRTP]P..P.....	EEDKMTPSTPVRSIS	848	862	YJR092W	BUD4
......P.[ILMVPYAFTR]P.[RKW]...	EHVPLLPPLPRWEEI	459	473	YJR092W	BUD4
...PPPP...	SMSGTPNPPAPPMHP	277	291	YJR093C	FIP1
...PPPP...	GTPNPPAPPMHPSFP	280	294	YJR093C	FIP1
...PPPP...	NPPAPPMHPSFPPLP	283	297	YJR093C	FIP1
...PPPP...	APPMHPSFPPLPMFG	286	300	YJR093C	FIP1
...PPPP...	SFPPFPMPGMMPPMN	301	315	YJR093C	FIP1
.[FPLWA].[WYLMFHP].[AVLIMFHPR]P..P.....	PAPPMHPSFPPLPMF	285	299	YJR093C	FIP1
[FPLWA]..[WYLMFHP].[AVLIMFHPP]P..P.....	PNPPAPPMHPSFPPL	282	296	YJR093C	FIP1
[FPLWA]..[WYLMFHP].[AVLIMFHPP]P..P.....	PMHPSFPPLPMFGSF	288	302	YJR093C	FIP1
......P.[ILMVPYAFTR]P.[RKW]...	TSKVLEPSRPFWGLV	541	555	YJR103W	URA8
.[FPLWA].[WYLMFHP].[AVLIMFHPR]P..P.....	VLNFSAPFIPHVFKD	189	203	YJR105W	ADO1
[FPLWA]..[WYLMFHP].[AVLIMFHPP]P..P.....	ASGLLPPIQPGFDIS	231	245	YJR106W	ECM27
.[FPLWA].[WYLMFHP].[AVLIMFHPR]P..P.....	GLGYTLPELPNPITK	345	359	YJR109C	CPA2
......P.[ILMVPYAFTR]P.[RKW]...	EHYEFCPTYPSKLFV	186	200	YJR110W	YJR110W
...[RK].[AVLIMFHRTP]P..P.....	EALKSVPIPPRKNAT	99	113	YJR113C	RSM7
......P.[ILMVPYAFTR]P.[RKW]...	KTILDHPNIPWKLII	5	19	YJR117W	STE24
...[RK].[AVLIMFHRTP]P..P.....	ENIKITPQKPFWFSD	474	488	YJR122W	CAF17
......P.[ILMVPYAFTR]P.[RKW]...	PGDPTTPGYPSKDSD	338	352	YJR126C	VPS70
......P.[ILMVPYAFTR]P.[RKW]...	LKHGWKPLRPIKLIS	475	489	YJR126C	VPS70
.[FPLWA].[WYLMFHP].[AVLIMFHPR]P..P.....	RLSFFFPLDPAYIRN	36	50	YJR127C	ZMS1
...R..[FLIYM].[FLIYM]P.....	ALFRLSFFFPLDPAY	33	47	YJR127C	ZMS1
......P.[ILMVPYAFTR]P.[RKW]...	GRVEDTPDLPQKTGI	1226	1240	YJR137C	ECM17
....RP[AVLIMFHRKTP][AVLIMFHRTP][AVLIMFHRTP]..[LP]...	EYLSRPITADLLKHT	1045	1059	YJR140C	HIR3
..[KRP]....P...[KR]P..	EVRKVYIPKVVKPLP	526	540	YJR143C	PMT4
......P.[ILMVPYAFTR]P.[RKW]...	QHQQRQPKRPKRYSL	213	227	YJR147W	HMS2
[FPLWA]..[WYLMFHP].[AVLIMFHPP]P..P.....	LIFLWIPDDPSKARF	264	278	YJR152W	DAL5
......P.[ILMVPYAFTR]P.[RKW]...	VINGNTPDAPKRGFL	41	55	YJR158W	HXT16
......P.[ILMVPYAFTR]P.[RKW]...	DRVAIEPGVPSRYSD	88	102	YJR159W	SOR1
[FPLWA]..[WYLMFHP].[AVLIMFHPP]P..P.....	LGIFFAPESPWWLVK	285	299	YJR160C	MPH3
.[FPLWA].[WYLMFHP].[AVLIMFHPR]P..P.....	SAPYEAPKAPELHLR	161	175	YKL001C	MET14
......P.[ILMVPYAFTR]P.[RKW]...	IMQNVFPAAPPWYKI	193	207	YKL004W	AUR1
...[KR][KR][AHPKRLG]P[PLV].......	ALRRRLPVTRSKINW	237	251	YKL007W	CAP1
...R..[FLIYM].[FLIYM]P.....	PTMRNKFEIPTKIKA	163	177	YKL009W	MRT4
......P.[ILMVPYAFTR]P.[RKW]...	VSILENPSTPDKTEE	721	735	YKL010C	UFD4
...R..[FLIYM].[FLIYM]P.....	LTRRFPFLFPFDTRM	1045	1059	YKL010C	UFD4
...R..[FLIYM].[FLIYM]P.....	SYERMLILFPDELVD	1342	1356	YKL010C	UFD4
.[FPLWA].[WYLMFHP].[AVLIMFHPR]P..P.....	YFDFSLPILPRLLVP	155	169	YKL014C	URB1
...[RK].[AVLIMFHRTP]P..P.....	ILERLTPLVPNNLLL	268	282	YKL017C	HCS1
...R..[FLIYM].[FLIYM]P.....	VANRLLIDLPTVDAT	494	508	YKL017C	HCS1
...[KR][KR][AHPKRLG]P[PLV].......	QFGRKLPLRTPYANP	92	106	YKL020C	SPT23
..[KRP]......P.[KR]P..	ELPKYEDLFPKKPKN	938	952	YKL020C	SPT23
...PPPP...	ANNVPPPMQPPPIES	199	213	YKL025C	PAN3
...PPPP...	VPPPMQPPPIESSNL	202	216	YKL025C	PAN3
..[KRP]......P.[KR]P..	RRKQLKKQGPKRPSS	352	366	YKL032C	IXR1
.[FPLWA].[WYLMFHP].[AVLIMFHPR]P..P.....	TLPPKRPSGPFIQFT	431	445	YKL032C	IXR1
....RP[AVLIMFHRKTP][AVLIMFHRTP][AVLIMFHRTP]..[LP]...	YDENRPRPAFIPDEG	508	522	YKL034W	TUL1
......P.[ILMVPYAFTR]P.[RKW]...	INSNTVPSTPSRSNS	134	148	YKL038W	RGT1
.[FPLWA].[WYLMFHP].[AVLIMFHPR]P..P.....	DFNYRPPNPPANNPT	942	956	YKL038W	RGT1
...[RK].[AVLIMFHRTP]P..P.....	VAVKIIPKKPWNAQQ	114	128	YKL048C	ELM1
...[RK].[AVLIMFHRTP]P..P.....	EMHRAAPETPRSISN	41	55	YKL050C	YKL050C
......P.[ILMVPYAFTR]P.[RKW]...	SSMVPSPIVPNKMRK	211	225	YKL052C	ASK1
...[RK].[AVLIMFHRTP]P..P.....	DKGKHVPSKPVSNTT	146	160	YKL054C	DEF1

Table S6

Motif	Motif Match	Start	End	ORF	Common name
...PPPP...	GTPVPPVPLPFGIPP	400	414	YKL059C	MPE1
...PPPP...	VPPVPLPFGIPPFPM	403	417	YKL059C	MPE1
.[FPLWA].[WYLMFHP].[AVLIMFHPR]P..P.....	PLPFGIPPFPMFPMP	407	421	YKL059C	MPE1
..[KRP]......P.[KR]P..	MSPVGNPEGPEKPNK	312	326	YKL060C	FBA1
...[RK].[AVLIMFHRTP]P..P.....	GHIKTHPNSPTPGIK	453	467	YKL064W	MNR2
..[KRP]....P...[KR]P..	STPSYKKPAGLRPSD	128	142	YKL064W	MNR2
....P.R.A[VP].....	GRLRPKRIAPWHLIQ	350	364	YKL064W	MNR2
...[KR][KR][AHPKRLG]P[PLV].......	KLSKKPPLGTLFSSK	16	30	YKL070W	YKL070W
..[KRP]......P.[KR]P..	SEKPEETPNPLKPWV	65	79	YKL077W	YKL077W
...[RK].[AVLIMFHRTP]P..P.....	VRCRVLPATPRQIRM	104	118	YKL086W	SRX1
...[RK].[AVLIMFHRTP]P..P.....	QKKKPTPTRPYNYVP	340	354	YKL089W	MIF2
..[KRP]......P.[KR]P..	RKKQKKKPTPTRPYN	337	351	YKL089W	MIF2
..[KRP]....P...[KR]P..	DKKDLDSPIEVKPEI	848	862	YKL092C	BUD2
......P.[ILMVPYAFTR]P.[RKW]...	NKENEGPEYPTKIEK	774	788	YKL101W	HSL1
..[KRP]......P.[KR]P..	LQKKNDRPSPLKPIQ	858	872	YKL101W	HSL1
.[FPLWA].[WYLMFHP].[AVLIMFHPR]P..P.....	MPLPQLPKSPSRYSL	619	633	YKL101W	HSL1
......P.[ILMVPYAFTR]P.[RKW]...	EENAGQPEIPLKSNN	222	236	YKL104C	GFA1
...[RK].[AVLIMFHRTP]P..P.....	EASKCFPKAPQASTT	878	892	YKL105C	YKL105C
...R..[FLIYM].[FLIYM]P.....	TKRRLQIAYPSLQKT	227	241	YKL108W	SLD2
..[KRP]....P...[KR]P..	TSKHWVLPPRPRPGR	65	79	YKL109W	HAP4
...PPPP...	TATANIPIPPPPPPM	1102	1116	YKL129C	MYO3
...PPPP...	ANIPIPPPPPPMGQP	1105	1119	YKL129C	MYO3
...PPPP...	PIPPPPPPMGQPKDP	1108	1122	YKL129C	MYO3
...PPPP...	PPPPPMGQPKDPKFE	1111	1125	YKL129C	MYO3
...[KR][KR][AHPKRLG]P[PLV].......	KDSKRRPPTAGDKII	570	584	YKL129C	MYO3
......P.[ILMVPYAFTR]P.[RKW]...	PQSLARPPPPKRIRT	29	43	YKL139W	CTK1
...R..[FLIYM].[FLIYM]P.....	FPTRTNIKIPILLIY	351	365	YKL140W	TGL1
..[KRP]....P...[KR]P..	EGKNEETPQNEKPPA	179	193	YKL144C	RPC25
..[KRP]....P...[KR]P..	NRPNTLDPALLRPGR	356	370	YKL145W	RPT1
...[KR][KR][AHPKRLG]P[PLV].......	GLLKKPPLLVNNEAV	64	78	YKL146W	AVT3
......P.[ILMVPYAFTR]P.[RKW]...	VADTLQPGLPHKPLP	464	478	YKL148C	SDH1
.[FPLWA].[WYLMFHP].[AVLIMFHPR]P..P.....	TLQPGLPHKPLPSDL	467	481	YKL148C	SDH1
...R..[FLIYM].[FLIYM]P.....	ESMRHLMLLPHGGYV	87	101	YKL149C	DBR1
...R..[FLIYM].[FLIYM]P.....	DTRRFTFKLPTEDSE	67	81	YKL150W	MCR1
...PPPP...	DVPPPPIDASSPFSQ	118	132	YKL152C	GPM1
...[KR][KR][AHPKRLG]P[PLV].......	ELVKRLPPGLSMLGS	873	887	YKL157W	APE2
...PPPP...	MFLISPPASPPPEFD	109	123	YKL159C	RCN1
...PPPP...	ISPPASPPPEFDFSK	112	126	YKL159C	RCN1
......P.[ILMVPYAFTR]P.[RKW]...	SPYQSTPTPPAKRLF	38	52	YKL165C	MCD4
...R..[FLIYM].[FLIYM]P.....	KLPRNFLNLPYTQRK	313	327	YKL171W	YKL171W
....RP[AS]....Y...	MEQIRPSELKEYIFG	151	165	YKL176C	LST4
...[RK].[AVLIMFHRTP]P..P.....	NIAKAHPNFPIALQW	718	732	YKL182W	FAS1
...[RK].[AVLIMFHRTP]P..P.....	PMQKPVPFVPVLDRR	1010	1024	YKL182W	FAS1
...PPPP...	TPPVTPPMSPPTNRT	286	300	YKL185W	ASH1
......P.[ILMVPYAFTR]P.[RKW]...	ASSSPSPSTPTKSGK	421	435	YKL185W	ASH1
...[KR][KR][AHPKRLG]P[PLV].......	ETSKRLPLFSQPSSS	813	827	YKL188C	PXA2
.[FPLWA].[WYLMFHP].[AVLIMFHPR]P..P.....	CAIFYLPQRPYMGNR	547	561	YKL188C	PXA2
...R..[FLIYM].[FLIYM]P.....	STFREQIIYPDSIEQ	562	576	YKL188C	PXA2
...PPPP...	ATVLASSPPPPPPAT	571	585	YKL198C	PTK1
...PPPP...	LASSPPPPPPATHVP	574	588	YKL198C	PTK1
...PPPP...	SPPPPPPATHVPAEA	577	591	YKL198C	PTK1
...PPPP...	EEPPATPAPSAPSAP	610	624	YKL198C	PTK1
.[FPLWA].[WYLMFHP].[AVLIMFHPR]P..P.....	TPAPSAPSAPSARVR	615	629	YKL198C	PTK1
...PPPP...	GKSETSTPPPPPPGL	445	459	YKL204W	EAP1
...PPPP...	ETSTPPPPPPGLIAH	448	462	YKL204W	EAP1
...PPPP...	TPPPPPPGLIAHQGP	451	465	YKL204W	EAP1
...PPPP...	NFPQRMMPPPPGLVQ	475	489	YKL204W	EAP1
...PPPP...	LPQQQYMPPPPPPGF	529	543	YKL204W	EAP1
...PPPP...	QQYMPPPPPPGFFPM	532	546	YKL204W	EAP1
...PPPP...	MPPPPPPGFFPMHPN	535	549	YKL204W	EAP1
...[RK].[AVLIMFHRTP]P..P.....	FPQRMMPPPPGLVQF	476	490	YKL204W	EAP1
......P.[ILMVPYAFTR]P.[RKW]...	PVMGVPPNFPQRMMP	468	482	YKL204W	EAP1
.[FPLWA].[WYLMFHP].[AVLIMFHPR]P..P.....	DLRPVIPRGPSSITD	271	285	YKL204W	EAP1
.[FPLWA].[WYLMFHP].[AVLIMFHPR]P..P.....	GFFPMHPNFPNGPMP	542	556	YKL204W	EAP1
[FPLWA]..[WYLMFHP].[AVLIMFHPP]P..P.....	PMHPNFPNGPMPPLP	545	559	YKL204W	EAP1
....RP[AVLIMFHRKTP][AVLIMFHRTP][AVLIMFHRTP]..[LP]...	PTDLRPVIPRGPSSI	269	283	YKL204W	EAP1
[FPLWA]..[WYLMFHP].[AVLIMFHPP]P..P.....	PIIFEIPFNPIYKFN	1002	1016	YKL205W	LOS1
......P.[ILMVPYAFTR]P.[RKW]...	ILFNSMPNVPVRLKL	149	163	YKL208W	CBT1
...[KR][KR][AHPKRLG]P[PLV].......	EIVKKGPPALLTMVI	1230	1244	YKL209C	STE6
...R..[FLIYM].[FLIYM]P.....	LLDRFILDLPDGLET	483	497	YKL209C	STE6
..[KRP]....P...[KR]P..	GLKYAQSPKFSKPDP	605	619	YKL212W	SAC1
......P.[ILMVPYAFTR]P.[RKW]...	YREREEPPLPKRIRI	54	68	YKL214C	YRA2

Table S6

Motif	Motif Match	Start	End	ORF	Common name
......P.[ILMVPYAFTR]P.[RKW]...	ELNLSCPNVPGKPQV	128	142	YKL216W	URA1
[FPLWA]..[WYLMFHP].[AVLIMFHPP]P..P.....	LSCPNVPGKPQVAYD	131	145	YKL216W	URA1
......P.[ILMVPYAFTR]P.[RKW]...	RLTVKKPARPWRAKP	447	461	YKL220C	FRE2
..[KRP]....P...[KR]P..	TVKKPARPWRAKPGQ	449	463	YKL220C	FRE2
...R..[FLIYM].[FLIYM]P.....	GYVRGYLTLPTIGSK	203	217	YKL220C	FRE2
...[RK].[AVLIMFHRTP]P..P.....	KTGKPLPTQPSSSKA	558	572	YKR001C	VPS1
......P.[ILMVPYAFTR]P.[RKW]...	KMINNTPIVPEKFSN	331	345	YKR004C	ECM9
...[KR][KR][AHPKRLG]P[PLV].......	TKAKKLPPNEQGSVK	302	316	YKR009C	FOX2
......P.[ILMVPYAFTR]P.[RKW]...	KNVVSPPFFPEKELN	451	465	YKR010C	TOF2
..[KRP]....P...[KR]P..	NLKIDCAPAYLKPNC	56	70	YKR011C	YKR011C
...[RK].[AVLIMFHRTP]P..P.....	ALPKRRPSPPLQSSL	210	224	YKR019C	IRS4
[FPLWA]..[WYLMFHP].[AVLIMFHPP]P..P.....	ADSHAIPVDPPSYFD	674	688	YKR021W	YKR021W
...R..[FLIYM].[FLIYM]P.....	IKKRNKIRLPSGSPE	3	17	YKR022C	YKR022C
......P.[ILMVPYAFTR]P.[RKW]...	INSEKFPKLPHKLVH	333	347	YKR024C	DBP7
...[RK].[AVLIMFHRTP]P..P.....	AYERPLPDLPSTIKP	748	762	YKR027W	FMP50
..[KRP]....P...[KR]P..	ERPLPDLPSTIKPLA	750	764	YKR027W	FMP50
......P.[ILMVPYAFTR]P.[RKW]...	NPLYTTPKTPPRPKT	954	968	YKR028W	SAP190
......P.[ILMVPYAFTR]P.[RKW]...	LNRNNGPKIPQKNTI	578	592	YKR029C	SET3
...[RK].[AVLIMFHRTP]P..P.....	YDRKVIPRMPWHDVQ	862	876	YKR031C	SPO14
...[KR][KR][AHPKRLG]P[PLV].......	HGKRRAPLLLAKLDV	234	248	YKR031C	SPO14
[FPLWA]..[WYLMFHP].[AVLIMFHPP]P..P.....	ARTLKIPNEPSPGYN	197	211	YKR038C	KAE1
.[FPLWA].[WYLMFHP].[AVLIMFHPR]P..P.....	PATLPLPGTPTLAAV	169	183	YKR041W	YKR041W
.[FPLWA].[WYLMFHP].[AVLIMFHPR]P..P.....	CFTLLFPSGPTWWLF	585	599	YKR050W	TRK2
...R..[FLIYM].[FLIYM]P.....	TSSRASLALPFQLRL	7	21	YKR050W	TRK2
...[RK].[AVLIMFHRTP]P..P.....	AGQRTFPEDPNYPVV	376	390	YKR051W	YKR051W
.[FPLWA].[WYLMFHP].[AVLIMFHPR]P..P.....	YWCLPRPKSPPVDRI	131	145	YKR053C	YSR3
...R..[FLIYM].[FLIYM]P.....	EVMRPDIVIPTIDTI	2384	2398	YKR054C	DYN1
...[RK].[AVLIMFHRTP]P..P.....	LDEKNLPRLPTPFAR	37	51	YKR055W	RHO4
[FPLWA]..[WYLMFHP].[AVLIMFHPP]P..P.....	LQQPLAPALPVNDLI	829	843	YKR064W	YKR064W
......P.[ILMVPYAFTR]P.[RKW]...	VQEMQGPKIPWRCGR	183	197	YKR066C	CCP1
...[RK].[AVLIMFHRTP]P..P.....	NAAKPIPHVPQASRR	9	23	YKR067W	GPT2
..[KRP]....P...[KR]P..	INPLGITPLGPKPDI	353	367	YKR076W	ECM4
...[KR][KR][AHPKRLG]P[PLV].......	PVPKKKPVTSDRVEK	93	107	YKR077W	YKR077W
[FPLWA]..[WYLMFHP].[AVLIMFHPP]P..P.....	AIKLGVPKGPLFAKL	212	226	YKR079C	YKR079C
...R..[FLIYM].[FLIYM]P.....	VAPRCILTFPATMDE	134	148	YKR082W	NUP133
...[RK].[AVLIMFHRTP]P..P.....	LKKKKTPKKPIAAAN	78	92	YKR084C	HBS1
..[KRP]......P.[KR]P..	VQRYYKTTVPTKPKK	139	153	YKR084C	HBS1
..[KRP]......P.[KR]P..	YYKTTVPTKPKKPHD	142	156	YKR084C	HBS1
.[FPLWA].[WYLMFHP].[AVLIMFHPR]P..P.....	TFDMNRPLLPGTPFI	512	526	YKR084C	HBS1
....RP[AVLIMFHRKTP][AVLIMFHRTP][AVLIMFHRTP]..[LP]...	FDMNRPLLPGTPFIL	513	527	YKR084C	HBS1
...R..[FLIYM].[FLIYM]P.....	SDPRRGLDMPVQSTK	79	93	YKR085C	MRPL20
...PPPP...	MFPSPTPPLVSPTAV	1	15	YKR091W	SRL3
...[RK].[AVLIMFHRTP]P..P.....	FIRRYTPLKPITKIF	411	425	YKR093W	PTR2
..[KRP]......P.[KR]P..	VYPFIRRYTPLKPIT	408	422	YKR093W	PTR2
....RP[AVLIMFHRKTP][AVLIMFHRTP][AVLIMFHRTP]..[LP]...	DTLRRPILPSMLTNE	663	677	YKR096W	YKR096W
..[KRP]......P.[KR]P..	IKRKRAYSLPLRPLP	131	145	YKR098C	UBP11
...R..[FLIYM].[FLIYM]P.....	KKLRPDLNIPDDQQD	375	389	YKR098C	UBP11
...[RK].[AVLIMFHRTP]P..P.....	NENKKLPPAPTVFGL	237	251	YKR103W	NFT1
....RP[AVLIMFHRKTP][AVLIMFHRTP][AVLIMFHRTP]..[LP]...	GERSRPLVISILSCA	155	169	YKR105C	YKR105C
....RP[AVLIMFHRKTP][AVLIMFHRTP][AVLIMFHRTP]..[LP]...	NISYRPLLLRRLVAK	319	333	YKR105C	YKR105C
......P.[ILMVPYAFTR]P.[RKW]...	AKFAKSPLLPFKLLS	327	341	YKR106W	YKR106W
....P.R.A[VP].....	PWGSPERDAVVESYR	530	544	YKR106W	YKR106W
...[KR][KR][AHPKRLG]P[PLV].......	DYFRKHPVYRTISTK	296	310	YLL001W	DNM1
.[FPLWA].[WYLMFHP].[AVLIMFHPR]P..P.....	LAVYNIPLFPDDPKA	277	291	YLL002W	RTT109
.[FPLWA].[WYLMFHP].[AVLIMFHPR]P..P.....	DPPFHLPSPPVDSTN	73	87	YLL003W	SFI1
..[KRP]....P...[KR]P..	NIKVLLNPKLGKPVK	14	28	YLL007C	YLL007C
...[KR][KR][AHPKRLG]P[PLV].......	SDKKKRPPILMLHGL	216	230	YLL012W	YLL012W
...PPPP...	YPNPMMFMPPPPLSA	382	396	YLL013C	PUF3
...PPPP...	FMPPPPLSAPQQQQQ	388	402	YLL013C	PUF3
.[FPLWA].[WYLMFHP].[AVLIMFHPR]P..P.....	NPMMFMPPPPLSAPQ	384	398	YLL013C	PUF3
...[RK].[AVLIMFHRTP]P..P.....	PRFKNFPTLPSKINS	60	74	YLL015W	BPT1
...R..[FLIYM].[FLIYM]P.....	LEIRPFYTMPDPANP	450	464	YLL018C	DPS1
......PR.P.R...	RRASLFPRDPKRLRP	543	557	YLL018C	DPS1
......P.[ILMVPYAFTR]P.[RKW]...	SLSALRPTPPERGSF	8	22	YLL018C-A	COX19
...PPPP...	DAPRPPLPQPMKQEV	127	141	YLL021W	SPA2
......P.[ILMVPYAFTR]P.[RKW]...	ELAKNSPLAPIKKNV	1082	1096	YLL021W	SPA2
......P.[ILMVPYAFTR]P.[RKW]...	ANNQGPPNLPARDKS	6	20	YLL023C	YLL023C
......P.[ILMVPYAFTR]P.[RKW]...	GDRPYPPSLPSRDLY	110	124	YLL028W	TPO1
....RP[AVLIMFHRKTP][AVLIMFHRTP][AVLIMFHRTP]..[LP]...	GVGFRPHLMNFPLRA	639	653	YLL029W	YLL029W
...R..[FLIYM].[FLIYM]P.....	DALRFDFAIPVNESH	89	103	YLL031C	GPI13
...R..[FLIYM].[FLIYM]P.....	AICRISLPLPMNTND	88	102	YLL032C	YLL032C
..[KRP]....P...[KR]P..	NRPDMIDPAMLRPGR	677	691	YLL034C	YLL034C

Table S6

Motif	Motif Match	Start	End	ORF	Common name
...R..[FLIYM].[FLIYM]P.....	PCNRLLLLYPGNQIT	253	267	YLL036C	PRP19
[FPLWA]..[WYLMFHP].[AVLIMFHPP]P..P.....	FTDPNAPEMPADVLR	1037	1051	YLL040C	VPS13
....RP[AS]....Y...	DIRIRPASEDIYDWS	2032	2046	YLL040C	VPS13
....RP[AVLIMFHRKTP][AVLIMFHRTP][AVLIMFHRTP]..[LP]...	PQSARPTPTYVPQYS	88	102	YLL043W	FPS1
...[KR][KR][AHPKRLG]P[PLV].......	NMLKKKPLKKPLKRF	65	79	YLL046C	RNP1
...[RK].[AVLIMFHRTP]P..P.....	LSLRYAPNLPRVIKN	1386	1400	YLL048C	YBT1
...R..[FLIYM].[FLIYM]P.....	NYIRGHFVLPTFLVD	207	221	YLL051C	FRE6
...[KR][KR][AHPKRLG]P[PLV].......	GIKRRAPVTHIHPLV	286	300	YLL057C	JLP1
...[RK].[AVLIMFHRTP]P..P.....	GYPRYFPQPPVQRLC	45	59	YLL058W	YLL058W
...[KR][KR][AHPKRLG]P[PLV].......	SDQKKKPLLCDTVGI	261	275	YLL058W	YLL058W
...R..[FLIYM].[FLIYM]P.....	VDVRKRLGLPETYPG	314	328	YLL063C	AYT1
...[RK].[AVLIMFHRTP]P..P.....	RVTRKRPREPKSTND	166	180	YLL066C	YLL066C
..[KRP]......P.[KR]P..	TERLKRDLCPRKPIE	60	74	YLL066C	YLL066C
...[RK].[AVLIMFHRTP]P..P.....	RVTRKRPREPKSTND	166	180	YLL067C	YLL067C
..[KRP]......P.[KR]P..	TERLKRDLCPRKPIE	60	74	YLL067C	YLL067C
[FPLWA]..[WYLMFHP].[AVLIMFHPP]P..P.....	PQKPIAPSNPHSFKQ	674	688	YLR006C	SSK1
....RP[AVLIMFHRKTP][AVLIMFHRTP][AVLIMFHRTP]..[LP]...	NKYTRPLIRKGLNFT	221	235	YLR006C	SSK1
.....[GP]P.[IVL].P[FWY]...	PIALLPPMVTPWGTT	51	65	YLR017W	MEU1
....RP[AVLIMFHRKTP][AVLIMFHRTP][AVLIMFHRTP]..[LP]...	KKKKRPARHSRPLSI	60	74	YLR020C	YLR020C
......P.[ILMVPYAFTR]P.[RKW]...	IHRAARPTRPDKARR	36	50	YLR029C	RPL15A
...[KR][KR][AHPKRLG]P[PLV].......	RGNRKRPVPKGATYG	68	82	YLR029C	RPL15A
......P.[ILMVPYAFTR]P.[RKW]...	EYVQETPIIPKRIIH	8	22	YLR030W	YLR030W
...[KR][KR][AHPKRLG]P[PLV].......	TFFKKRPLSRKNSIK	102	116	YLR030W	YLR030W
[FPLWA]..[WYLMFHP].[AVLIMFHPP]P..P.....	FRDMDLPQHPVIHDC	46	60	YLR030W	YLR030W
...[RK].[AVLIMFHRTP]P..P.....	ELLRIIPEMPKDLVM	81	95	YLR032W	RAD5
....RP[AS]....Y...	DIIDRPSMCLEYTTS	66	80	YLR035C	MLH2
...PPPP...	ADLPLPDLPPEPPTE	529	543	YLR035C-A	YLR035C-A
...PPPP...	PLPDLPPEPPTELSD	532	546	YLR035C-A	YLR035C-A
.[FPLWA].[WYLMFHP].[AVLIMFHPR]P..P.....	IADLPLPDLPPEPPT	528	542	YLR035C-A	YLR035C-A
.[FPLWA].[WYLMFHP].[AVLIMFHPR]P..P.....	LPLPDLPPEPPTELS	531	545	YLR035C-A	YLR035C-A
.[FPLWA].[WYLMFHP].[AVLIMFHPR]P..P.....	IPKLNVPLNPKGRKL	893	907	YLR035C-A	YLR035C-A
[FPLWA]..[WYLMFHP].[AVLIMFHPP]P..P.....	PEYLSLPFEPYCFYH	321	335	YLR054C	OSW2
.[FPLWA].[WYLMFHP].[AVLIMFHPR]P..P.....	QFDLKMPSKPIHNLK	489	503	YLR055C	SPT8
...[RK].[AVLIMFHRTP]P..P.....	SQWKNHPDWPFWVNK	727	741	YLR057W	YLR057W
....RP[AVLIMFHRKTP][AVLIMFHRTP][AVLIMFHRTP]..[LP]...	SQYLRPRTIQNLQSM	27	41	YLR059C	REX2
...[RK].[AVLIMFHRTP]P..P.....	TVPRNRPMAPFGTII	2	16	YLR064W	YLR064W
.[FPLWA].[WYLMFHP].[AVLIMFHPR]P..P.....	NLLPFLPLIPLNLKN	137	151	YLR064W	YLR064W
.[FPLWA].[WYLMFHP].[AVLIMFHPR]P..P.....	EFSHYAPTAPHVQKE	735	749	YLR069C	MEF1
...R..[FLIYM].[FLIYM]P.....	VSYRESITIPADFDY	543	557	YLR069C	MEF1
......P.[ILMVPYAFTR]P.[RKW]...	DRVALEPGIPDRFSP	89	103	YLR070C	XYL2
..[KRP]....P...[KR]P..	DTKRLGTPESVKPAN	1030	1044	YLR071C	RGR1
.[FPLWA].[WYLMFHP].[AVLIMFHPR]P..P.....	VLSLGRPNLPTHNFK	206	220	YLR071C	RGR1
......P.[ILMVPYAFTR]P.[RKW]...	KDYRFNPAIPLRIYL	12	26	YLR073C	YLR073C
...R..[FLIYM].[FLIYM]P.....	GLRRARYKFPGQQKI	151	165	YLR075W	RPL10
...[RK].[AVLIMFHRTP]P..P.....	NLLREIPQVPAFQLG	142	156	YLR077W	FMP25
..[KRP]....P...[KR]P..	CAKPMNIPEVLKPGQ	535	549	YLR077W	FMP25
...[RK].[AVLIMFHRTP]P..P.....	KIIKDVPGTPSDKVI	165	179	YLR079W	SIC1
...[RK].[AVLIMFHRTP]P..P.....	QIARQIPFQPWYLKT	511	525	YLR083C	EMP70
.....P.R.P.R...	SYSQSPPRSPGRSPT	105	119	YLR086W	SMC4
.....P.R.P.R...	SPPRSPGRSPTRRLE	109	123	YLR086W	SMC4
......P.[ILMVPYAFTR]P.[RKW]...	FQIVTKPVVPKKTKK	316	330	YLR087C	CSF1
...[KR][KR][AHPKRLG]P[PLV].......	KPIKKLPPGSRRIYT	459	473	YLR087C	CSF1
.[FPLWA].[WYLMFHP].[AVLIMFHPR]P..P.....	IPTLPLPTLPDTIDY	1131	1145	YLR087C	CSF1
...R..[FLIYM].[FLIYM]P.....	PYHRCPFLLPLFYQD	1100	1114	YLR087C	CSF1
...[RK].[AVLIMFHRTP]P..P.....	TGSKTVPQAPQAPQT	763	777	YLR095C	IOC2
...[KR][KR][AHPKRLG]P[PLV].......	PTDKRKPLSECSTFI	640	654	YLR095C	IOC2
...PPPP...	FTRPPIPAALPPSDM	829	843	YLR096W	KIN2
.[FPLWA].[WYLMFHP].[AVLIMFHPR]P..P.....	SLKFTRPPIPAALPP	826	840	YLR096W	KIN2
....RP[AVLIMFHRKTP][AVLIMFHRTP][AVLIMFHRTP]..[LP]...	LKFTRPPIPAALPPS	827	841	YLR096W	KIN2
......P.[ILMVPYAFTR]P.[RKW]...	GKLFQLPSLPPWKTP	10	24	YLR102C	APC9
...[KR][KR][AHPKRLG]P[PLV].......	LLYKRGPPFQHAEFC	287	301	YLR105C	SEN2
...R..[FLIYM].[FLIYM]P.....	NQKRYKYPLPIHPVD	7	21	YLR105C	SEN2
...[RK].[AVLIMFHRTP]P..P.....	RHLKNIPRIPIFCIL	2507	2521	YLR106C	MDN1
.[FPLWA].[WYLMFHP].[AVLIMFHPR]P..P.....	LLLLFVPDSPYDPAI	2904	2918	YLR106C	MDN1
...PPPP...	KKPGFPPLLPPSYIA	163	177	YLR108C	YLR108C
......P.[ILMVPYAFTR]P.[RKW]...	EMIEGKPLFPGKDHV	209	223	YLR113W	HOG1
..R[YFLEP]..[AVLIMFHWRTP]P[GSDLIAV]P.....	VLRFLGFPLPIFEKG	269	283	YLR114C	YLR114C
...[RK].[AVLIMFHRTP]P..P.....	SKHKMFPFNPAKIKK	532	546	YLR115W	CFT2
...[KR][KR][AHPKRLG]P[PLV].......	QNSRKRPLKDGAKTT	568	582	YLR115W	CFT2
...PPPP...	PGIAPPPLQSPPESE	409	423	YLR116W	MSL5
...PPPP...	IAPPPGLSGPPGFSN	442	456	YLR116W	MSL5
...PPPP...	NKPTPPGLQGPPGL*	463	477	YLR116W	MSL5

Table S6

Motif	Motif Match	Start	End	ORF	Common name
.[FPLWA].[WYLMFHP].[AVLIMFHPR]P..P.....	QPKFSLPPPPGMTTV	424	438	YLR116W	MSL5
......P.[ILMVPYAFTR]P.[RKW]...	MDVVPSPGLPEKVNE	31	45	YLR119W	SRN2
......P.[ILMVPYAFTR]P.[RKW]...	WKLFFQPEYPVRMEP	191	205	YLR128W	YLR128W
..[KRP]......P.[KR]P..	WQRKKLGEAPIKPQG	776	790	YLR129W	DIP2
[FPLWA]..[WYLMFHP].[AVLIMFHPP]P..P.....	LRDLDIPLVPSPKTG	70	84	YLR131C	ACE2
....RP[AVLIMFHRKTP][AVLIMFHRTP][AVLIMFHRTP]..[LP]...	IAEIRPLPRHALPLS	181	195	YLR141W	RRN5
...[RK].[AVLIMFHRTP]P..P.....	TIIKLFPYIPIPVIK	91	105	YLR142W	PUT1
...[RK].[AVLIMFHRTP]P..P.....	NIIKYVPWGPPLETK	426	440	YLR142W	PUT1
....P.R.A[VP].....	LKKYPERKAPFMVST	241	255	YLR142W	PUT1
......P.[ILMVPYAFTR]P.[RKW]...	DLSKYGPLPPSRACV	385	399	YLR143W	YLR143W
...PPPP...	AIPPPVPNRPGGTTN	7	21	YLR144C	ACF2
...PPPP...	TTNRGPPPLPPRANV	19	33	YLR144C	ACF2
...PPPP...	RGPPPLPPRANVQPP	22	36	YLR144C	ACF2
......P.[ILMVPYAFTR]P.[RKW]...	SRQAIPPPVPNRPGG	4	18	YLR144C	ACF2
......P.[ILMVPYAFTR]P.[RKW]...	KKNSSRPMPPIRGPK	85	99	YLR147C	SMD3
...R..[FLIYM].[FLIYM]P.....	SLSRRRFSLPSMPNV	476	490	YLR149C	YLR149C
...PPPP...	LTDVPPQPNSPPDNV	37	51	YLR154W-C	YLR154W-C
....RP[AS]....Y...	ISCMRPANQEIYPLR	57	71	YLR156W	YLR156W
...PPPP...	ASPQPASVPPPQNGP	70	84	YLR157C-A	YLR157C-A
...PPPP...	SVPPPQNGPYPQQCM	76	90	YLR157C-A	YLR157C-A
...PPPP...	ASPQPASVPPPQNGP	70	84	YLR157C-B	YLR157C-B
...PPPP...	SVPPPQNGPYPQQCM	76	90	YLR157C-B	YLR157C-B
.[FPLWA].[WYLMFHP].[AVLIMFHPR]P..P.....	IADLPLPDLPPESPT	1128	1142	YLR157C-B	YLR157C-B
.[FPLWA].[WYLMFHP].[AVLIMFHPR]P..P.....	IPKLNVPLNPKGRKL	1493	1507	YLR157C-B	YLR157C-B
....RP[AS]....Y...	ISCMRPANQEIYPL*	57	71	YLR157W-A	YLR157W-A
....RP[AS]....Y...	ISCMRPANQEIYPLR	57	71	YLR159W	YLR159W
....RP[AS]....Y...	ISCMRPANQEIYPLR	57	71	YLR161W	YLR161W
......P.[ILMVPYAFTR]P.[RKW]...	VFVSMSPLPPARANK	48	62	YLR162W	YLR162W
......P.[ILMVPYAFTR]P.[RKW]...	FTIPFLPKIPQKPGG	24	38	YLR164W	YLR164W
.[FPLWA].[WYLMFHP].[AVLIMFHPR]P..P.....	PFLPKIPQKPGGVSG	27	41	YLR164W	YLR164W
[FPLWA]..[WYLMFHP].[AVLIMFHPP]P..P.....	ARSFTIPFLPKIPQK	21	35	YLR164W	YLR164W
...PPPP...	VNKPPGIPSQPPDCR	19	33	YLR165C	PUS5
...[KR][KR][AHPKRLG]P[PLV].......	AIERKLPLYSTTKNT	200	214	YLR174W	IDP2
...R..[FLIYM].[FLIYM]P.....	HLIRDKLVLPYLDVD	24	38	YLR174W	IDP2
...R..[FLIYM].[FLIYM]P.....	TVFREPIIIPRIPRL	106	120	YLR174W	IDP2
...PPPP...	SHKPSPTPTQPPAQP	136	150	YLR176C	RFX1
...PPPP...	PSPTPTQPPAQPATQ	139	153	YLR176C	RFX1
......P.[ILMVPYAFTR]P.[RKW]...	TLVMTDPDAPSKTDH	90	104	YLR178C	TFS1
......P.[ILMVPYAFTR]P.[RKW]...	ALLMTDPDAPSRTEH	74	88	YLR179C	YLR179C
[FPLWA]..[WYLMFHP].[AVLIMFHPP]P..P.....	LTRPSAPSEPAAAEH	262	276	YLR181C	VTA1
...[RK].[AVLIMFHRTP]P..P.....	LNKRVLPSKPKKSVK	351	365	YLR183C	TOS4
...[KR][KR][AHPKRLG]P[PLV].......	GSIRKHPLIFTDTSM	268	282	YLR183C	TOS4
..[KRP]......P.[KR]P..	KPKLNKRVLPSKPKK	348	362	YLR183C	TOS4
.[FPLWA].[WYLMFHP].[AVLIMFHPR]P..P.....	SLLFALPTLPHIYYL	397	411	YLR187W	YLR187W
..[KRP]......P.[KR]P..	VFKNVSFTYPTRPKH	433	447	YLR188W	MDL1
...R..[FLIYM].[FLIYM]P.....	GSFRDLIAIPNSELN	655	669	YLR188W	MDL1
...[RK].[AVLIMFHRTP]P..P.....	LKTKSHPEVPRLDKR	153	167	YLR189C	ATG26
..[KRP]......P.[KR]P..	GPKPSISLVPDKPSE	15	29	YLR189C	ATG26
..[KRP]....P...[KR]P..	LKKSGKLPTWVKPFL	56	70	YLR193C	YLR193C
...PPPP...	LDPPPSPEDPTPENR	46	60	YLR203C	MSS51
.[FPLWA].[WYLMFHP].[AVLIMFHPR]P..P.....	ALGLDPPPSPEDPTP	43	57	YLR203C	MSS51
......P.[ILMVPYAFTR]P.[RKW]...	FIVNYQPKPPIREGN	94	108	YLR213C	CRR1
......P.[ILMVPYAFTR]P.[RKW]...	SKEYRYPQTPMRLEI	292	306	YLR213C	CRR1
......P.[ILMVPYAFTR]P.[RKW]...	VHDVVLPKFPDKSFV	236	250	YLR215C	CDC123
...[KR][KR][AHPKRLG]P[PLV].......	TRRKKRPPAVKNAEA	319	333	YLR219W	MSC3
[FPLWA]..[WYLMFHP].[AVLIMFHPP]P..P.....	ADWYEVPKVPLSAFR	814	828	YLR223C	IFH1
...PPPP...	ASPQPASVPPPQNGP	70	84	YLR227W-A	YLR227W-A
...PPPP...	SVPPPQNGPYPQQCM	76	90	YLR227W-A	YLR227W-A
...PPPP...	ASPQPASVPPPQNGP	70	84	YLR227W-B	YLR227W-B
...PPPP...	SVPPPQNGPYPQQCM	76	90	YLR227W-B	YLR227W-B
...PPPP...	ADLPLPDPPPEPPTE	1129	1143	YLR227W-B	YLR227W-B
...PPPP...	PLPDPPPEPPTELSD	1132	1146	YLR227W-B	YLR227W-B
.[FPLWA].[WYLMFHP].[AVLIMFHPR]P..P.....	IADLPLPDPPPEPPT	1128	1142	YLR227W-B	YLR227W-B
.[FPLWA].[WYLMFHP].[AVLIMFHPR]P..P.....	LPLPDPPPEPPTELS	1131	1145	YLR227W-B	YLR227W-B
.[FPLWA].[WYLMFHP].[AVLIMFHPR]P..P.....	IPKLNVPLNPKGRKL	1493	1507	YLR227W-B	YLR227W-B
...R..[FLIYM].[FLIYM]P.....	ESLRDEFNIPTFKSM	13	27	YLR231C	BNA5
......P.[ILMVPYAFTR]P.[RKW]...	KDFILTPDFPERRRL	280	294	YLR233C	EST1
[FPLWA]..[WYLMFHP].[AVLIMFHPP]P..P.....	LKEMTHPIKPSAQTL	28	42	YLR239C	LIP2
......P.[ILMVPYAFTR]P.[RKW]...	TLPIYTPYIPFRNSR	72	86	YLR240W	VPS34
......P.[ILMVPYAFTR]P.[RKW]...	KVKASYPLTPRRYVP	87	101	YLR244C	MAP1
...R..[FLIYM].[FLIYM]P.....	SQLRNNYQIPQEYYN	142	156	YLR246W	ERF2
...PPPP...	ASPQPASVPPPQNGP	70	84	YLR256W-A	YLR256W-A

Table S6

Motif	Motif Match	Start	End	ORF	Common name
...PPPP...	SVPPPQNGPYPQQCM	76	90	YLR256W-A	YLR256W-A
[FPLWA]..[WYLMFHP].[AVLIMFHPP]P..P.....	ARPLSVPGSPRDLRS	647	661	YLR258W	GSY2
..[KRP]......P.[KR]P..	DKRNLRSNAPTKPKS	599	613	YLR263W	RED1
...[KR][KR][AHPKRLG]P[PLV].......	EKGKKRPPICKKICF	219	233	YLR267W	BOP2
...R..[FLIYM].[FLIYM]P.....	FFTRLQFKYPILDEQ	314	328	YLR278C	YLR278C
...PPPP...	EPEDFLPPPPKPHFA	16	30	YLR285W	NNT1
...PPPP...	DFLPPPPKPHFAEYQ	19	33	YLR285W	NNT1
..[KRP]......P.[KR]P..	EEPEDFLPPPPKPHF	15	29	YLR285W	NNT1
..[KRP]......P.[KR]P..	TPKVEKTEKPKKPKG	18	32	YLR287C-A	RPS30A
...PPPP...	RIPPPTGRPDKPFRA	223	237	YLR289W	GUF1
...[RK].[AVLIMFHRTP]P..P.....	SADKGFPLIPSGYIN	163	177	YLR290C	YLR290C
[FPLWA]..[WYLMFHP].[AVLIMFHPP]P..P.....	ATLYWAPVSPLKSIN	1421	1435	YLR305C	STT4
.[FPLWA].[WYLMFHP].[AVLIMFHPR]P..P.....	WPGLDPPYIPLDFID	64	78	YLR308W	CDA2
.[FPLWA].[WYLMFHP].[AVLIMFHPR]P..P.....	SAYLHVPRNPSKSRR	158	172	YLR310C	CDC25
...R..[FLIYM].[FLIYM]P.....	DKVRSILQLPKYGIN	48	62	YLR312W-A	MRPL15
......P.[ILMVPYAFTR]P.[RKW]...	GNRCNEPHLPPKWVQ	149	163	YLR318W	EST2
...[KR][KR][AHPKRLG]P[PLV].......	LESRRKPVFNTVVEV	528	542	YLR320W	MMS22
...PPPP...	YNRPPVTAPPAPAYT	214	228	YLR326W	YLR326W
....RP[AVLIMFHRKTP][AVLIMFHRTP][AVLIMFHRTP]..[LP]...	KMYNRPPVTAPPAPA	212	226	YLR326W	YLR326W
...[RK].[AVLIMFHRTP]P..P.....	KIPKSIPIIPYVLAD	27	41	YLR328W	NMA1
...[KR][KR][AHPKRLG]P[PLV].......	LCRRRGPPIQNFQIF	177	191	YLR329W	REC102
..[KRP]....P...[KR]P..	SIPSKNTPDASKPSF	355	369	YLR335W	NUP2
..[KRP]....P...[KR]P..	ATKVDATPEESKPIN	584	598	YLR335W	NUP2
...R..[FLIYM].[FLIYM]P.....	VMNRRKIAMPKRRMA	35	49	YLR335W	NUP2
...PPPP...	MAGAPAPPPPPPPPA	1	15	YLR337C	VRP1
...PPPP...	APAPPPPPPPPALGG	4	18	YLR337C	VRP1
...PPPP...	PPPPPPPPALGGSAP	7	21	YLR337C	VRP1
...PPPP...	PIVPSSPAPPLPLSG	169	183	YLR337C	VRP1
...PPPP...	VSNPPQAPPPPPTPT	232	246	YLR337C	VRP1
...PPPP...	PPQAPPPPPTPTIGL	235	249	YLR337C	VRP1
...PPPP...	APPPPPTPTIGLDSK	238	252	YLR337C	VRP1
...PPPP...	QPPLPSSAPPIPTSH	307	321	YLR337C	VRP1
...PPPP...	SAPPIPTSHAPPLPP	313	327	YLR337C	VRP1
...PPPP...	PIPTSHAPPLPPTAP	316	330	YLR337C	VRP1
...PPPP...	TSHAPPLPPTAPPPP	319	333	YLR337C	VRP1
...PPPP...	APPLPPTAPPPPSLP	322	336	YLR337C	VRP1
...PPPP...	LPPTAPPPPSLPNVT	325	339	YLR337C	VRP1
...PPPP...	TAPPPPSLPNVTSAP	328	342	YLR337C	VRP1
...PPPP...	KKATSAPAPPPPPLP	343	357	YLR337C	VRP1
...PPPP...	TSAPAPPPPPLPAAM	346	360	YLR337C	VRP1
...PPPP...	PAPPPPPLPAAMSSA	349	363	YLR337C	VRP1
...PPPP...	ATPVPPTLAPPLPNT	370	384	YLR337C	VRP1
...PPPP...	KASSMPAPPPPPPPP	391	405	YLR337C	VRP1
...PPPP...	SMPAPPPPPPPPPGA	394	408	YLR337C	VRP1
...PPPP...	APPPPPPPPPGAFST	397	411	YLR337C	VRP1
...PPPP...	PPPPPPPGAFSTSSA	400	414	YLR337C	VRP1
...PPPP...	SSIPLAPLPPPPPPS	418	432	YLR337C	VRP1
...PPPP...	PLAPLPPPPPPSVAT	421	435	YLR337C	VRP1
...PPPP...	PLPPPPPPSVATSVP	424	438	YLR337C	VRP1
...PPPP...	VATSVPSAPPPPPTL	433	447	YLR337C	VRP1
...PPPP...	SVPSAPPPPPTLTTN	436	450	YLR337C	VRP1
...PPPP...	SAPPPPPTLTTNKPS	439	453	YLR337C	VRP1
...PPPP...	MAPPLPPSAPPPPIT	565	579	YLR337C	VRP1
...PPPP...	PLPPSAPPPPITSLP	568	582	YLR337C	VRP1
...PPPP...	PSAPPPPITSLPTPT	571	585	YLR337C	VRP1
...PPPP...	APALPGHVPPPPVPP	607	621	YLR337C	VRP1
...PPPP...	LPGHVPPPPVPPVLS	610	624	YLR337C	VRP1
...PPPP...	HVPPPPVPPVLSDDS	613	627	YLR337C	VRP1
...PPPP...	SIPSPPPVAPTLSVR	676	690	YLR337C	VRP1
...PPPP...	SKNPTKSPPPPPSPS	697	711	YLR337C	VRP1
...PPPP...	PTKSPPPPPSPSTMD	700	714	YLR337C	VRP1
...PPPP...	SPPPPPSPSTMDTGT	703	717	YLR337C	VRP1
...[RK].[AVLIMFHRTP]P..P.....	NSVKATPVPPTLAPP	366	380	YLR337C	VRP1
...[RK].[AVLIMFHRTP]P..P.....	NPTKSPPPPPSPSTM	699	713	YLR337C	VRP1
.[FPLWA].[WYLMFHP].[AVLIMFHPR]P..P.....	PAPPPPPPPPALGGS	5	19	YLR337C	VRP1
.[FPLWA].[WYLMFHP].[AVLIMFHPR]P..P.....	GPSMSAPPIPGMGAP	76	90	YLR337C	VRP1
.[FPLWA].[WYLMFHP].[AVLIMFHPR]P..P.....	VAAPPIPNAPLSPAP	130	144	YLR337C	VRP1
.[FPLWA].[WYLMFHP].[AVLIMFHPR]P..P.....	PPIPNAPLSPAPAVP	133	147	YLR337C	VRP1
.[FPLWA].[WYLMFHP].[AVLIMFHPR]P..P.....	SPAPAVPSIPSSSAP	141	155	YLR337C	VRP1
.[FPLWA].[WYLMFHP].[AVLIMFHPR]P..P.....	SAAPPIPIVPSSPAP	163	177	YLR337C	VRP1
.[FPLWA].[WYLMFHP].[AVLIMFHPR]P..P.....	PPIPIVPSSPAPPLP	166	180	YLR337C	VRP1
.[FPLWA].[WYLMFHP].[AVLIMFHPR]P..P.....	HAPPLPPTAPPPPSL	321	335	YLR337C	VRP1

Table S6

Motif	Motif Match	Start	End	ORF	Common name
.[FPLWA].[WYLMFHP].[AVLIMFHPR]P..P.....	PLPPTAPPPPSLPNV	324	338	YLR337C	VRP1
.[FPLWA].[WYLMFHP].[AVLIMFHPR]P..P.....	APAPPPPPLPAAMSS	348	362	YLR337C	VRP1
.[FPLWA].[WYLMFHP].[AVLIMFHPR]P..P.....	MPAPPPPPPPPPGAF	395	409	YLR337C	VRP1
.[FPLWA].[WYLMFHP].[AVLIMFHPR]P..P.....	PAPPPPPPPPPGAFS	396	410	YLR337C	VRP1
.[FPLWA].[WYLMFHP].[AVLIMFHPR]P..P.....	LAPLPPPPPPSVATS	422	436	YLR337C	VRP1
.[FPLWA].[WYLMFHP].[AVLIMFHPR]P..P.....	LPTFSAPSLPQQSVS	660	674	YLR337C	VRP1
[FPLWA]..[WYLMFHP].[AVLIMFHPP]P..P.....	AGAPAPPPPPPPPAL	2	16	YLR337C	VRP1
[FPLWA]..[WYLMFHP].[AVLIMFHPP]P..P.....	PQAPPPPPTPTIGLD	236	250	YLR337C	VRP1
[FPLWA]..[WYLMFHP].[AVLIMFHPP]P..P.....	PTSHAPPLPPTAPPP	318	332	YLR337C	VRP1
[FPLWA]..[WYLMFHP].[AVLIMFHPP]P..P.....	PTAPPPPSLPNVTSA	327	341	YLR337C	VRP1
[FPLWA]..[WYLMFHP].[AVLIMFHPP]P..P.....	ASSMPAPPPPPPPPP	392	406	YLR337C	VRP1
[FPLWA]..[WYLMFHP].[AVLIMFHPP]P..P.....	FSSYGPPGTPGYDSY	93	107	YLR342W	FKS1
[FPLWA]..[WYLMFHP].[AVLIMFHPP]P..P.....	AIAMALPNEPYPAWT	138	152	YLR342W	FKS1
....RP[AVLIMFHRKTP][AVLIMFHRTP][AVLIMFHRTP]..[LP]...	NGRRRPKFRVQLSGN	1061	1075	YLR342W	FKS1
......P.[ILMVPYAFTR]P.[RKW]...	EANEKLPETPDRSKC	376	390	YLR343W	YLR343W
...R..[FLIYM].[FLIYM]P.....	ITHRLRISIPGITGR	198	212	YLR350W	ORM2
...R..[FLIYM].[FLIYM]P.....	VNGRKFFKYPSPIRH	546	560	YLR357W	RSC2
....RP[AVLIMFHRKTP][AVLIMFHRTP][AVLIMFHRTP]..[LP]...	VKRGRPPIIDLPYIQ	265	279	YLR357W	RSC2
......P.[ILMVPYAFTR]P.[RKW]...	IGTNTTPEIPSWATS	669	683	YLR362W	STE11
...R..[FLIYM].[FLIYM]P.....	KNVRHKYVFPLFYPT	161	175	YLR368W	MDM30
[FPLWA]..[WYLMFHP].[AVLIMFHPP]P..P.....	LDNFTLPGTPGFPLN	102	116	YLR370C	ARC18
....RP[AVLIMFHRKTP][AVLIMFHRTP][AVLIMFHRTP]..[LP]...	KVFQRPIPLPLLFAC	927	941	YLR371W	ROM2
....RP[AVLIMFHRKTP][AVLIMFHRTP][AVLIMFHRTP]..[LP]...	DNDMRPVILVRPRLH	175	189	YLR380W	CSR1
..R[YFLEP]..[AVLIMFHWRTP]P[GSDLIAV]P.....	RQRYWGTPIPIIHCD	449	463	YLR382C	NAM2
.....[GP]P.[IVL].P[FWY]...	YKMAQGPTLEPFDNG	426	440	YLR384C	IKI3
...PPPP...	SSTPAAPPTPPTPPT	94	108	YLR390W	ECM19
...PPPP...	PAAPPTPPTPPTPPQ	97	111	YLR390W	ECM19
.[FPLWA].[WYLMFHP].[AVLIMFHPR]P..P.....	AAPPTPPTPPTPPQQ	98	112	YLR390W	ECM19
...R..[FLIYM].[FLIYM]P.....	GNLRTDIPIPEYDED	69	83	YLR390W	ECM19
...R..[FLIYM].[FLIYM]P.....	IDVRTSLRLPPQLLD	87	101	YLR396C	VPS33
..[KRP]....P...[KR]P..	NRPNSVDPALRRPGR	390	404	YLR397C	AFG2
...PPPP...	VPKEPAPAPPPEPDM	127	141	YLR399C	BDF1
...PPPP...	EPAPAPPPEPDMNNL	130	144	YLR399C	BDF1
.[FPLWA].[WYLMFHP].[AVLIMFHPR]P..P.....	ELPMEVPKEPAPAPP	122	136	YLR399C	BDF1
....RP[AVLIMFHRKTP][AVLIMFHRTP][AVLIMFHRTP]..[LP]...	THNGRPKRTIHPPKS	284	298	YLR399C	BDF1
....RP[AVLIMFHRKTP][AVLIMFHRTP][AVLIMFHRTP]..[LP]...	KTHGRPVTIAGPMVR	34	48	YLR405W	DUS4
..[KRP]....P...[KR]P..	DGKTMTKPFVEKPVD	340	354	YLR410W	VIP1
[FPLWA]..[WYLMFHP].[AVLIMFHPP]P..P.....	LEAYNVPTPPRLEIS	285	299	YLR410W	VIP1
...PPPP...	VSPQPASVPPPQNGQ	70	84	YLR410W-A	YLR410W-A
...PPPP...	VSPQPASVPPPQNGQ	70	84	YLR410W-B	YLR410W-B
.[FPLWA].[WYLMFHP].[AVLIMFHPR]P..P.....	LPKLNVPLNPKGKKL	1508	1522	YLR410W-B	YLR410W-B
....RP[AVLIMFHRKTP][AVLIMFHRTP][AVLIMFHRTP]..[LP]...	ELYIRPPPHLGLNDK	1374	1388	YLR410W-B	YLR410W-B
...[RK].[AVLIMFHRTP]P..P.....	VNPRNLPSVPNGLVN	265	279	YLR418C	CDC73
...[RK].[AVLIMFHRTP]P..P.....	GKPRKTPRPPFPFFR	150	164	YLR422W	YLR422W
...PPPP...	VKERRPPPPPPLLYS	100	114	YLR425W	TUS1
...PPPP...	RRPPPPPPLLYSTES	103	117	YLR425W	TUS1
...[RK].[AVLIMFHRTP]P..P.....	KERRPPPPPPLLYST	101	115	YLR425W	TUS1
...[RK].[AVLIMFHRTP]P..P.....	EREKALPPIPFTTTL	197	211	YLR425W	TUS1
...R..[FLIYM].[FLIYM]P.....	ESQRKSIELPKLPPL	22	36	YLR425W	TUS1
....RP[AVLIMFHRKTP][AVLIMFHRTP][AVLIMFHRTP]..[LP]...	KLIERPIPIDYLSLE	759	773	YLR425W	TUS1
...[RK].[AVLIMFHRTP]P..P.....	LDWRKIPFLPKEIYE	488	502	YLR427W	MAG2
......P.[ILMVPYAFTR]P.[RKW]...	PWHQLEPLAPYKFFD	1687	1701	YLR430W	SEN1
......P.[ILMVPYAFTR]P.[RKW]...	RNASSSPFIPKKRKP	2215	2229	YLR430W	SEN1
......P.[ILMVPYAFTR]P.[RKW]...	HQLEYWPHRPQWPTP	881	895	YLR436C	ECM30
..[KRP]....P...[KR]P..	IKKSVSSPKVCKPSP	47	61	YLR453C	RIF2
...[KR][KR][AHPKRLG]P[PLV].......	FPVRRGPLFGLQSIS	1657	1671	YLR454W	FMP27
..[KRP]....P...[KR]P..	GDKYQVDPKTCKPIT	1837	1851	YLR454W	FMP27
[FPLWA]..[WYLMFHP].[AVLIMFHPP]P..P.....	LRTLFVPLIPSVTVE	1339	1353	YLR454W	FMP27
......P.[ILMVPYAFTR]P.[RKW]...	NISPACPTPPYRSRE	285	299	YLR457C	NBP1
......P.[ILMVPYAFTR]P.[RKW]...	LDLKWEPSTPQRKGP	163	177	YLR460C	YLR460C
......P.[ILMVPYAFTR]P.[RKW]...	VCKLRSPNTPRRLRK	93	107	YLR464W	YLR464W
..[KRP]......P.[KR]P..	TERLKRDLCPRKPIE	13	27	YLR464W	YLR464W
...[RK].[AVLIMFHRTP]P..P.....	RVTRKRPREPKSTND	343	357	YLR466W	YRF1-4
...[RK].[AVLIMFHRTP]P..P.....	RVTRKRPREPKSTND	757	771	YLR467W	YRF1-5
......P.[ILMVPYAFTR]P.[RKW]...	VCKLRSPNTPRRLRK	309	323	YLR467W	YRF1-5
..[KRP]......P.[KR]P..	TERLKRDLCPRKPIE	229	243	YLR467W	YRF1-5
..[KRP]....P...[KR]P..	LIPVQELPLLLKPNK	362	376	YML010W	SPT5
......P.[ILMVPYAFTR]P.[RKW]...	PKISKVPKAPTRETH	178	192	YML013W	SEL1
......P.[ILMVPYAFTR]P.[RKW]...	NSNVPDPSTPSKPNL	59	73	YML016C	PPZ1
[FPLWA]..[WYLMFHP].[AVLIMFHPP]P..P.....	WEPFSLPKDPKVVVI	283	297	YML018C	YML018C
..[KRP]......P.[KR]P..	PAPLTNTYIPYKPDA	158	172	YML020W	YML020W
...PPPP...	FSVKPPTPAPPSLKN	175	189	YML021C	UNG1

Table S6

Motif	Motif Match	Start	End	ORF	Common name
...R..[FLIYM].[FLIYM]P.....	LDDRGTIKLPPLNTS	38	52	YML027W	YOX1
...[RK].[AVLIMFHRTP]P..P.....	YRPKCIPCPPNGICY	512	526	YML034W	SRC1
...PPPP...	NKPGWVVYPPPPKPS	220	234	YML035C	AMD1
...PPPP...	GWVVYPPPPKPSYNS	223	237	YML035C	AMD1
...PPPP...	VYPPPPKPSYNSDTK	226	240	YML035C	AMD1
[FPLWA]..[WYLMFHP].[AVLIMFHPP]P..P.....	WVVYPPPPKPSYNSD	224	238	YML035C	AMD1
[FPLWA]..[WYLMFHP].[AVLIMFHPP]P..P.....	PSLWEAPQNPPYSYY	588	602	YML035C	AMD1
...PPPP...	ASPQPASVPPPQNGP	70	84	YML039W	YML039W
...PPPP...	SVPPPQNGPYPQQCM	76	90	YML039W	YML039W
.[FPLWA].[WYLMFHP].[AVLIMFHPR]P..P.....	IADLPLPDLPPESPT	1128	1142	YML039W	YML039W
.[FPLWA].[WYLMFHP].[AVLIMFHPR]P..P.....	IPKLNVPLNPKGRKL	1493	1507	YML039W	YML039W
...PPPP...	ASPQPASVPPPQNGP	70	84	YML040W	YML040W
...PPPP...	SVPPPQNGPYPQQCM	76	90	YML040W	YML040W
...PPPP...	ASPQPASVPPPQNGP	70	84	YML045W	YML045W
...PPPP...	SVPPPQNGPYPQQCM	76	90	YML045W	YML045W
.[FPLWA].[WYLMFHP].[AVLIMFHPR]P..P.....	IADLPLPDLPPESPT	1128	1142	YML045W	YML045W
.[FPLWA].[WYLMFHP].[AVLIMFHPR]P..P.....	IPKLNVPLNPKGRKL	1493	1507	YML045W	YML045W
...PPPP...	ASPQPASVPPPQNGP	70	84	YML045W-A	YML045W-A
...PPPP...	SVPPPQNGPYPQQCM	76	90	YML045W-A	YML045W-A
...R..[FLIYM].[FLIYM]P.....	FRPRTILSFPNNPKS	924	938	YML049C	RSE1
...[RK].[AVLIMFHRTP]P..P.....	FNTKKIPELPSQFVM	377	391	YML051W	GAL80
..[KRP]......P.[KR]P..	HYKKNIEICPLKPVS	113	127	YML053C	YML053C
...[RK].[AVLIMFHRTP]P..P.....	DLTRFLPNHPGGQDV	115	129	YML054C	CYB2
...[RK].[AVLIMFHRTP]P..P.....	AEFRRVPLPPMAEVP	29	43	YML058W	SML1
......P.[ILMVPYAFTR]P.[RKW]...	PPVSKDPNRPTKFAF	383	397	YML061C	PIF1
...[KR][KR][AHPKRLG]P[PLV].......	AGKRRLPLVRFKASD	651	665	YML061C	PIF1
..[KRP]......P.[KR]P..	KSRSTGFKNPLRPAL	151	165	YML061C	PIF1
.[FPLWA].[WYLMFHP].[AVLIMFHPR]P..P.....	TLNHIIPRRPFICSF	8	22	YML061C	PIF1
..[KRP]......P.[KR]P..	KKRGRKPKDPSKPRQ	328	342	YML065W	ORC1
...[KR][KR][AHPKRLG]P[PLV].......	VASRKAPLEELKFNH	187	201	YML067C	ERV41
...R..[FLIYM].[FLIYM]P.....	NQARKPFLLPATELS	39	53	YML069W	POB3
..[KRP]......P.[KR]P..	EFKKDLEEGPTKPKS	276	290	YML074C	FPR3
....RP[AVLIMFHRKTP][AVLIMFHRTP][AVLIMFHRTP]..[LP]...	DKIGRPTRIVAPMVD	24	38	YML080W	DUS1
....RP[AVLIMFHRKTP][AVLIMFHRTP][AVLIMFHRTP]..[LP]...	IYADRPPALSAPPCV	19	33	YML081W	YML081W
....RP[AVLIMFHRKTP][AVLIMFHRTP][AVLIMFHRTP]..[LP]...	RMHSRPLVATMLKHW	976	990	YML081W	YML081W
...R..[FLIYM].[FLIYM]P.....	CPERDVFLFPSGMAS	300	314	YML082W	YML082W
.[FPLWA].[WYLMFHP].[AVLIMFHPR]P..P.....	LPDLKLPPSPVPSTV	114	128	YML083C	YML083C
[FPLWA]..[WYLMFHP].[AVLIMFHPP]P..P.....	ATAPSPPLAPVAIKL	100	114	YML083C	YML083C
...R..[FLIYM].[FLIYM]P.....	SIERIVFDYPRVAAY	106	120	YML088W	UFO1
.[FPLWA].[WYLMFHP].[AVLIMFHPR]P..P.....	VASLFLPYQPQFELD	5	19	YML100W	TSL1
.[FPLWA].[WYLMFHP].[AVLIMFHPR]P..P.....	TLHYQIPDNPNSKAF	432	446	YML100W	TSL1
....RP[AVLIMFHRKTP][AVLIMFHRTP][AVLIMFHRTP]..[LP]...	AAKQRPLLAKQPSNL	254	268	YML100W	TSL1
..[KRP]....P...[KR]P..	NRPAIRIPSLKKPAL	307	321	YML102W	CAC2
...R..[FLIYM].[FLIYM]P.....	VKNRPAIRIPSLKKP	305	319	YML102W	CAC2
....RP[AVLIMFHRKTP][AVLIMFHRTP][AVLIMFHRTP]..[LP]...	NNFYRPLLRSVLVLL	1305	1319	YML103C	NUP188
...[RK].[AVLIMFHRTP]P..P.....	VFFKSLPDLPKPQPR	42	56	YML104C	MDM1
...[RK].[AVLIMFHRTP]P..P.....	ESLREIPYGPDFDNI	201	215	YML105C	SEC65
.[FPLWA].[WYLMFHP].[AVLIMFHPR]P..P.....	SPLPNIPPTPFDTAY	58	72	YML117W	NAB6
....RP[AVLIMFHRKTP][AVLIMFHRTP][AVLIMFHRTP]..[LP]...	AITKRPLILKGPIRA	306	320	YML118W	NGL3
.[FPLWA].[WYLMFHP].[AVLIMFHPR]P..P.....	LFTPLLPSAPVGTVD	89	103	YML120C	NDI1
.[FPLWA].[WYLMFHP].[AVLIMFHPR]P..P.....	YFRLTIPESPRYQLD	265	279	YML123C	PHO84
...PPPP...	LKPPAPKEPPNLSED	367	381	YML127W	RSC9
.[FPLWA].[WYLMFHP].[AVLIMFHPR]P..P.....	RLKPPAPKEPPNLSE	366	380	YML127W	RSC9
....RP[AVLIMFHRKTP][AVLIMFHRTP][AVLIMFHRTP]..[LP]...	CQRIRPLVLHKLADI	550	564	YML127W	RSC9
...R..[FLIYM].[FLIYM]P.....	FIYRSYLDLPRNIWE	514	528	YML130C	ERO1
...[RK].[AVLIMFHRTP]P..P.....	RVTRKRPREPKSTND	335	349	YML133C	YML133C
..[KRP]......P.[KR]P..	TERLKRDLCPRKPIE	229	243	YML133C	YML133C
....RP[AVLIMFHRKTP][AVLIMFHRTP][AVLIMFHRTP]..[LP]...	YHQNRPFLPHSLSPG	407	421	YMR001C	CDC5
[FPLWA]..[WYLMFHP].[AVLIMFHPP]P..P.....	ASTMAAPVHPQQQQQ	22	36	YMR002W	YMR002W
...[RK].[AVLIMFHRTP]P..P.....	YPFRMIPELPPKRIG	187	201	YMR004W	MVP1
.[FPLWA].[WYLMFHP].[AVLIMFHPR]P..P.....	GLNLSFPYVPDVNTF	457	471	YMR008C	PLB1
[FPLWA]..[WYLMFHP].[AVLIMFHPP]P..P.....	AGMLMVPESPRFLVE	242	256	YMR011W	HXT2
...[RK].[AVLIMFHRTP]P..P.....	VVKKNFPVSPNYTAP	409	423	YMR015C	ERG5
....RP[AVLIMFHRKTP][AVLIMFHRTP][AVLIMFHRTP]..[LP]...	TLRYRPPVLMVPYVV	396	410	YMR015C	ERG5
...R..[FLIYM].[FLIYM]P.....	IIVRSGIPFPLLCSF	70	84	YMR030W-A	YMR030W-A
...[RK].[AVLIMFHRTP]P..P.....	FILKLFPHEPIEGKS	293	307	YMR034C	YMR034C
...PPPP...	ADLPLPDLPPEPPTE	1129	1143	YMR045C	YMR045C
...PPPP...	PLPDLPPEPPTELSD	1132	1146	YMR045C	YMR045C
.[FPLWA].[WYLMFHP].[AVLIMFHPR]P..P.....	IADLPLPDLPPEPPT	1128	1142	YMR045C	YMR045C
.[FPLWA].[WYLMFHP].[AVLIMFHPR]P..P.....	LPLPDLPPEPPTELS	1131	1145	YMR045C	YMR045C
.[FPLWA].[WYLMFHP].[AVLIMFHPR]P..P.....	IPKLNVPLNPKGRKL	1493	1507	YMR045C	YMR045C
......P.[ILMVPYAFTR]P.[RKW]...	IIYANLPNRPKRGEG	1032	1046	YMR047C	NUP116

Table S6

Motif	Motif Match	Start	End	ORF	Common name
…[RK].[AVLIMFHRTP]P..P…..	MDAKFHPFKPQLFVC	643	657	YMR049C	ERB1
…[KR][KR][AHPKRLG]P[PLV]…….	NFHKKLPLFSSAADD	730	744	YMR049C	ERB1
..[KRP]….P…[KR]P..	LIPELPSPKDLRPFP	412	426	YMR049C	ERB1
…PPPP…	ASPQPASVPPPQNGP	70	84	YMR050C	YMR050C
…PPPP…	SVPPPQNGPYPQQCM	76	90	YMR050C	YMR050C
.[FPLWA].[WYLMFHP].[AVLIMFHPR]P..P…..	IADLPLPDLPPESPT	1128	1142	YMR050C	YMR050C
.[FPLWA].[WYLMFHP].[AVLIMFHPR]P..P…..	IPKLNVPLNPKGRKL	1493	1507	YMR050C	YMR050C
…PPPP…	ASPQPASVPPPQNGP	70	84	YMR051C	YMR051C
…PPPP…	SVPPPQNGPYPQQCM	76	90	YMR051C	YMR051C
……P.[ILMVPYAFTR]P.[RKW]…	DNHPAFPEYPMRRDT	445	459	YMR058W	FET3
…[KR][KR][AHPKRLG]P[PLV]…….	YRLKRAPLNYASHIP	22	36	YMR066W	SOV1
……P.[ILMVPYAFTR]P.[RKW]…	SNNSPLPVLPRRIST	246	260	YMR068W	AVO2
…[KR][KR][AHPKRLG]P[PLV]…….	EVKKKGPPLGAGKKP	200	214	YMR068W	AVO2
[FPLWA]..[WYLMFHP].[AVLIMFHPP]P..P…..	LGFYRAPGSPAPASP	243	257	YMR069W	NAT4
…R..[FLIYM].[FLIYM]P…..	ILEREIIYIPEDDTD	63	77	YMR069W	NAT4
…PPPP…	HSAPAPAPGPPHHHH	229	243	YMR070W	MOT3
…[RK].[AVLIMFHRTP]P..P…..	FDEKLPPKKPAGPFI	110	124	YMR072W	ABF2
.[FPLWA].[WYLMFHP].[AVLIMFHPR]P..P…..	FLCLDPPIDPNNLPK	284	298	YMR075W	RCO1
.[FPLWA].[WYLMFHP].[AVLIMFHPR]P..P…..	ILRLYAPDAPYTDAQ	83	97	YMR076C	PDS5
.[FPLWA].[WYLMFHP].[AVLIMFHPR]P..P…..	IFEYLLPFEPDNDKR	472	486	YMR076C	PDS5
……P.[ILMVPYAFTR]P.[RKW]…	MTNLRKPSAPKRMSS	49	63	YMR086W	YMR086W
……P.[ILMVPYAFTR]P.[RKW]…	SIRSNSPSPPEKINN	651	665	YMR086W	YMR086W
[FPLWA]..[WYLMFHP].[AVLIMFHPP]P..P…..	FHKPEAPMVPVTKVV	563	577	YMR086W	YMR086W
…PPPP…	LLPPPEEAEAPPLEQ	10	24	YMR088C	YMR088C
…PPPP…	GNNSRNIPPPPPPPP	148	162	YMR089C	YTA12
…PPPP…	SRNIPPPPPPPPPKP	151	165	YMR089C	YTA12
…PPPP…	IPPPPPPPPPKPPLN	154	168	YMR089C	YTA12
…PPPP…	PPPPPPPKPPLNDPS	157	171	YMR089C	YTA12
…PPPP…	PPPPKPPLNDPSNPV	160	174	YMR089C	YTA12
…[RK].[AVLIMFHRTP]P..P…..	NNSRNIPPPPPPPPP	149	163	YMR089C	YTA12
……P.[ILMVPYAFTR]P.[RKW]…	NIPPPPPPPPPKPPL	153	167	YMR089C	YTA12
.[FPLWA].[WYLMFHP].[AVLIMFHPR]P..P…..	PPPPPPPPPKPPLNDP	156	170	YMR089C	YTA12
..[KRP]……P.[KR]P..	LYKSNDVELPGKPSY	39	53	YMR094W	CTF13
…[KR][KR][AHPKRLG]P[PLV]…….	AKSRRLPVKKGHEAG	81	95	YMR101C	SRT1
[FPLWA]..[WYLMFHP].[AVLIMFHPP]P..P…..	LKGYIPPYKPIVKSE	609	623	YMR104C	YPK2
…[RK].[AVLIMFHRTP]P..P…..	EVDKKVPVLPMVAGG	644	658	YMR108W	ILV2
…PPPP…	RHSKKPAPPPPGMQN	1006	1020	YMR109W	MYO5
…PPPP…	KKPAPPPPGMQNKAA	1009	1023	YMR109W	MYO5
…PPPP…	SGRQANIPPPPPPPP	1066	1080	YMR109W	MYO5
…PPPP…	QANIPPPPPPPPPSS	1069	1083	YMR109W	MYO5
…PPPP…	IPPPPPPPPPSSKPK	1072	1086	YMR109W	MYO5
…PPPP…	PPPPPPPPSSKPKEPM	1075	1089	YMR109W	MYO5
…[RK].[AVLIMFHRTP]P..P…..	HSKKPAPPPPGMQNK	1007	1021	YMR109W	MYO5
…[KR][KR][AHPKRLG]P[PLV]…….	RESKRRPPTAGDKII	571	585	YMR109W	MYO5
..[KRP]….P…[KR]P..	PPPPPPPPPPSSKPKE	1073	1087	YMR109W	MYO5
[FPLWA]..[WYLMFHP].[AVLIMFHPP]P..P…..	ANIPPPPPPPPPSSK	1070	1084	YMR109W	MYO5
..[KRP]……P.[KR]P..	PIPESPANSPTRPQM	52	66	YMR115W	FMP24
…[RK].[AVLIMFHRTP]P..P…..	SQRKKRPISPHLTVY	68	82	YMR118C	YMR118C
…R..[FLIYM].[FLIYM]P…..	LKQRFFLMFPKSIIW	398	412	YMR119W	ASI1
……P.[ILMVPYAFTR]P.[RKW]…	IHRAARPTRPDKARR	36	50	YMR121C	RPL15B
…[KR][KR][AHPKRLG]P[PLV]…….	RGNRKRPVPKGATYG	68	82	YMR121C	RPL15B
…..P.R.P.R…	LLQSIPERDPKRNVS	650	664	YMR124W	YMR124W
……P.[ILMVPYAFTR]P.[RKW]…	NYRDFRPRMPKRQRI	15	29	YMR125W	STO1
…[RK].[AVLIMFHRTP]P..P…..	IPRRYIPITPADLPH	80	94	YMR126C	DLT1
……P.[ILMVPYAFTR]P.[RKW]…	IINFPFPTPPDRVAL	862	876	YMR128W	ECM16
.[FPLWA].[WYLMFHP].[AVLIMFHPR]P..P…..	LPHMSRPLGPDEVLE	98	112	YMR131C	RRB1
…R..[FLIYM].[FLIYM]P…..	MLRRFQMNLPNDVEF	106	120	YMR133W	REC114
…R..[FLIYM].[FLIYM]P…..	SNNRSNITLPHDSIQ	198	212	YMR133W	REC114
……P.[ILMVPYAFTR]P.[RKW]…	RNTHQIPSAPQRLVS	111	125	YMR136W	GAT2
…R..[FLIYM].[FLIYM]P…..	KPTRVKLVLPSFKII	206	220	YMR137C	PSO2
…R..[FLIYM].[FLIYM]P…..	SQPRKNYHLPSDDLK	247	261	YMR140W	SIP5
.[FPLWA].[WYLMFHP].[AVLIMFHPR]P..P…..	LFTPLLPSTPVGTIE	148	162	YMR145C	NDE1
.[FPLWA].[WYLMFHP].[AVLIMFHPR]P..P…..	NWRFGHPYPPNNFIE	306	320	YMR152W	YIM1
..[KRP]……P.[KR]P..	DGKPTATPSPFRPLE	198	212	YMR153W	NUP53
…[RK].[AVLIMFHRTP]P..P…..	FLIKFTPKFPQSIDH	10	24	YMR156C	TPP1
..[KRP]……P.[KR]P..	SHRLWLYAAPKRPKT	127	141	YMR156C	TPP1
.[FPLWA].[WYLMFHP].[AVLIMFHPR]P..P…..	LWLYAAPKRPKTFAA	130	144	YMR156C	TPP1
…[RK].[AVLIMFHRTP]P..P…..	LRERIIPILPANSKS	160	174	YMR164C	MSS11
[FPLWA]..[WYLMFHP].[AVLIMFHPP]P..P…..	LTDLSPPGTPPTMAT	273	287	YMR165C	SMP2
..[KRP]….P…[KR]P..	RNRASTLPSYPKPMT	515	529	YMR172W	HOT1
…R..[FLIYM].[FLIYM]P…..	QVNRSPISFPNASTD	356	370	YMR172W	HOT1
…[RK].[AVLIMFHRTP]P..P…..	KKQKAIPSSPCGMFN	685	699	YMR179W	SPT21

Table S6

Motif	Motif Match	Start	End	ORF	Common name
...PPPP...	YFPHPMPSAPIPLPH	139	153	YMR182C	RGM1
...PPPP...	PSAPIPLPHQPPPLP	145	159	YMR182C	RGM1
...PPPP...	PIPLPHQPPPLPIYS	148	162	YMR182C	RGM1
.[FPLWA].[WYLMFHP].[AVLIMFHPR]P..P.....	PLPHQPPPLPIYSYM	150	164	YMR182C	RGM1
......P.[ILMVPYAFTR]P.[RKW]...	ITSLHDPLVPIKSYG	736	750	YMR185W	YMR185W
....RP[AVLIMFHRKTP][AVLIMFHRTP][AVLIMFHRTP]..[LP]...	TFVNRPILVTCLNGL	317	331	YMR185W	YMR185W
...R..[FLIYM].[FLIYM]P.....	LEFRAILFIPKRAPF	311	325	YMR186W	HSC82
...R..[FLIYM].[FLIYM]P.....	QTERGLLSIPLTSSI	224	238	YMR187C	YMR187C
...[KR][KR][AHPKRLG]P[PLV].......	NILKRRPLKLEAPSK	102	116	YMR189W	GCV2
......P.[ILMVPYAFTR]P.[RKW]...	PKTNSPPTTPSKDEC	42	56	YMR190C	SGS1
....RP[AVLIMFHRKTP][AVLIMFHRTP][AVLIMFHRTP]..[LP]...	FNTFRPHMASSLVEN	131	145	YMR190C	SGS1
......P.[ILMVPYAFTR]P.[RKW]...	WSFVKEPSFPSRSAF	344	358	YMR191W	SPG5
......P.[ILMVPYAFTR]P.[RKW]...	LKRITSPPLPPRADC	119	133	YMR192W	APP2
......P.[ILMVPYAFTR]P.[RKW]...	QAPLDRPQLPPRQVV	156	170	YMR192W	APP2
......P.[ILMVPYAFTR]P.[RKW]...	SSSSTPPTLPPRRIE	197	211	YMR192W	APP2
...R..[FLIYM].[FLIYM]P.....	KGVRSSMFLPGFFTT	441	455	YMR192W	APP2
...R..[FLIYM].[FLIYM]P.....	SNWRGPIWFPTSFLI	823	837	YMR196W	YMR196W
......P.[ILMVPYAFTR]P.[RKW]...	IVVSQQPTAPMKKTL	450	464	YMR198W	CIK1
...R..[FLIYM].[FLIYM]P.....	LAYRPPMMLPTETLN	175	189	YMR200W	ROT1
...PPPP...	STKPTPPPAPEASAE	148	162	YMR205C	PFK2
...R..[FLIYM].[FLIYM]P.....	QYKRMIIKYPHGGEG	84	98	YMR210W	YMR210W
...[RK].[AVLIMFHRTP]P..P.....	TVIKTHPRIPKSEYQ	95	109	YMR211W	DML1
...R..[FLIYM].[FLIYM]P.....	EILRRNMNMPDGKLQ	236	250	YMR218C	TRS130
....RP[AVLIMFHRKTP][AVLIMFHRTP][AVLIMFHRTP]..[LP]...	RAKVRPLVRQWLPNL	88	102	YMR218C	TRS130
...[RK].[AVLIMFHRTP]P..P.....	TAEKSAPTSPEVYEI	1472	1486	YMR219W	ESC1
......P.[ILMVPYAFTR]P.[RKW]...	REEDSIPSRPQRRKS	255	269	YMR221C	FMP42
...R..[FLIYM].[FLIYM]P.....	EYLREYLNLPEHIVP	74	88	YMR230W	RPS10B
...R..[FLIYM].[FLIYM]P.....	FMCRLTLDIPVVFFK	929	943	YMR231W	PEP5
.[FPLWA].[WYLMFHP].[AVLIMFHPR]P..P.....	NLSPQIPNSPHEGCK	98	112	YMR232W	FUS2
..[KRP]....P...[KR]P..	VERNFTDPGDGKPGW	135	149	YMR238W	DFG5
...[RK].[AVLIMFHRTP]P..P.....	KATKWPPKLPEIQDL	194	208	YMR239C	RNT1
...R..[FLIYM].[FLIYM]P.....	QELRAIMNLPEGQLP	296	310	YMR240C	CUS1
......P.[ILMVPYAFTR]P.[RKW]...	ADKQFVPIAPQRLNM	1012	1026	YMR247C	YMR247C
..[KRP]....P...[KR]P..	VNPIGITPLGPKPDI	349	363	YMR251W	YMR251W
....RP[AVLIMFHRKTP][AVLIMFHRTP][AVLIMFHRTP]..[LP]...	KTRLRPAFIQQLWSC	441	455	YMR257C	PET111
...R..[FLIYM].[FLIYM]P.....	FCIRCQLEFPAILEE	716	730	YMR259C	YMR259C
.[FPLWA].[WYLMFHP].[AVLIMFHPR]P..P.....	VASLFLPYTPQFEAD	5	19	YMR261C	TPS3
.[FPLWA].[WYLMFHP].[AVLIMFHPR]P..P.....	TLHYQIPDNPNSKAF	399	413	YMR261C	TPS3
...[KR][KR][AHPKRLG]P[PLV].......	HHNRRKPLEVYFKAT	375	389	YMR265C	YMR265C
..[KRP]......P.[KR]P..	LPRLKWSAIPVRPFT	219	233	YMR265C	YMR265C
...[RK].[AVLIMFHRTP]P..P.....	DGIKELPEFPIKKYH	738	752	YMR266W	RSN1
......P.[ILMVPYAFTR]P.[RKW]...	GDTMKIPPAPWWKRF	805	819	YMR266W	RSN1
.[FPLWA].[WYLMFHP].[AVLIMFHPR]P..P.....	CPELWIPRDPFGFSK	866	880	YMR266W	RSN1
....RP[AVLIMFHRKTP][AVLIMFHRTP][AVLIMFHRTP]..[LP]...	LDLNRPLLMQILRSD	148	162	YMR272C	SCS7
...[KR][KR][AHPKRLG]P[PLV].......	FPPKRKPLLRPQRSD	10	24	YMR275C	BUL1
...PPPP...	MSPLGAPPPPPHKDH	202	216	YMR280C	CAT8
...PPPP...	LGAPPPPPHKDHLCD	205	219	YMR280C	CAT8
......P.[ILMVPYAFTR]P.[RKW]...	SPLGAPPPPPHKDHL	203	217	YMR280C	CAT8
......P.[ILMVPYAFTR]P.[RKW]...	SASHRGPRRPQKNRY	1391	1405	YMR280C	CAT8
..[KRP]......P.[KR]P..	SGPSASHRGPRRPQK	1388	1402	YMR280C	CAT8
.[FPLWA].[WYLMFHP].[AVLIMFHPR]P..P.....	QLSLIIPKNPYFLNM	500	514	YMR280C	CAT8
.[FPLWA].[WYLMFHP].[AVLIMFHPR]P..P.....	TALFTHPEGPNCTNT	1042	1056	YMR280C	CAT8
...[RK].[AVLIMFHRTP]P..P.....	LQDRVIPIFPHYPLI	56	70	YMR283C	RIT1
..[KRP]....P...[KR]P..	SIKEEKKPFDKKPKF	586	600	YMR284W	YKU70
...R..[FLIYM].[FLIYM]P.....	VKCRGFLRMPPGNEM	475	489	YMR285C	NGL2
...R..[FLIYM].[FLIYM]P.....	TKNRVLLRIPHKLPA	205	219	YMR287C	MSU1
[FPLWA]..[WYLMFHP].[AVLIMFHPP]P..P.....	AKSYGFPVPPKVNIT	474	488	YMR290C	HAS1
...[RK].[AVLIMFHRTP]P..P.....	YGFRVRPDFPVFGAA	131	145	YMR295C	YMR295C
......P.[ILMVPYAFTR]P.[RKW]...	EKLEKLPAIPRKFIV	275	289	YMR296C	LCB1
...R..[FLIYM].[FLIYM]P.....	MVTRSEILIPKGCDS	221	235	YMR299C	YMR299C
.[FPLWA].[WYLMFHP].[AVLIMFHPR]P..P.....	ELNPTIPYIPGIEYE	1041	1055	YMR306W	FKS3
...[RK].[AVLIMFHRTP]P..P.....	IKVKRIPYSPKSIRR	640	654	YMR309C	NIP1
...PPPP...	LEPEPLMSPVPPSSA	490	504	YMR313C	TGL3
.[FPLWA].[WYLMFHP].[AVLIMFHPR]P..P.....	RAKYLAPIHPWDDYS	195	209	YMR322C	SNO4
...R..[FLIYM].[FLIYM]P.....	VALRRDLKLPSPHFE	109	123	YNL005C	MRP7
...[RK].[AVLIMFHRTP]P..P.....	AALKYHPDKPTGDTE	29	43	YNL007C	SIS1
..[KRP]......P.[KR]P..	YRKAALKYHPDKPTG	26	40	YNL007C	SIS1
...[KR][KR][AHPKRLG]P[PLV].......	ALKRKLPLFFTTKNT	200	214	YNL009W	IDP3
...R..[FLIYM].[FLIYM]P.....	TVFREPIIIPKIPRL	106	120	YNL009W	IDP3
..[KRP]......P.[KR]P..	SAPQLTVPEPSKPCR	543	557	YNL018C	YNL018C
...PPPP...	DKKTKPTPPPKPSHL	604	618	YNL020C	ARK1
...PPPP...	TKPTPPPKPSHLKPK	607	621	YNL020C	ARK1

Table S6

Motif	Motif Match	Start	End	ORF	Common name
...PPPP...	PKPSHLKPKPPPKPL	613	627	YNL020C	ARK1
...PPPP...	SHLKPKPPPKPLLLA	616	630	YNL020C	ARK1
......P.[ILMVPYAFTR]P.[RKW]...	KDKKTKPTPPPKPSH	603	617	YNL020C	ARK1
......P.[ILMVPYAFTR]P.[RKW]...	KPSHLKPKPPPKPLL	614	628	YNL020C	ARK1
.[FPLWA].[WYLMFHP].[AVLIMFHPR]P..P.....	HLKPKPPPKPLLLAG	617	631	YNL020C	ARK1
..[KRP]......P.[KR]P..	KTRSSLHFKPMRPPQ	750	764	YNL023C	FAP1
.....P.R.P.R...	SLHFKPMRPPQRHFI	754	768	YNL023C	FAP1
[FPLWA]..[WYLMFHP].[AVLIMFHPP]P..P.....	PSTHAAPVTPIISIQ	322	336	YNL027W	CRZ1
..[KRP]......P.[KR]P..	SAPQLTVPEPSKPCR	543	557	YNL034W	YNL034W
...[KR][KR][AHPKRLG]P[PLV].......	IITKKKPVLEPSDYF	151	165	YNL040W	YNL040W
...PPPP...	QQQPLSQPLPPPPQQ	256	270	YNL042W	BOP3
...PPPP...	PLSQPLPPPPQQQQD	259	273	YNL042W	BOP3
..[KRP]....P...[KR]P..	GTKSQESPLNKKPTS	210	224	YNL042W	BOP3
......P.[ILMVPYAFTR]P.[RKW]...	ETWLYKPGMPPRPHF	491	505	YNL045W	YNL045W
[FPLWA]..[WYLMFHP].[AVLIMFHPP]P..P.....	LYKPGMPPRPHFITA	494	508	YNL045W	YNL045W
....RP[AS]....Y...	IEQRRPSRSPEYDQS	45	59	YNL045W	YNL045W
....RP[AVLIMFHRKTP][AVLIMFHRTP][AVLIMFHRTP]..[LP]...	GMPPRPHFITALADN	498	512	YNL045W	YNL045W
.....[GP]P.[IVL].P[FWY]...	LTGLVGPLLSPFSAV	150	164	YNL046W	YNL046W
......P.[ILMVPYAFTR]P.[RKW]...	ISNFVPPNLPMRRFK	418	432	YNL047C	LIT1
...R..[FLIYM].[FLIYM]P.....	LAQRNPIPYPIDAVT	52	66	YNL047C	LIT1
...[RK].[AVLIMFHRTP]P..P.....	KNKKKVPLNPLAKAG	248	262	YNL050C	YNL050C
...PPPP...	KCPPKVSPRPTPPSL	79	93	YNL053W	MSG5
...PPPP...	NTPPAPLLPLPSLSQ	229	243	YNL054W	VAC7
...PPPP...	ADLPLPDLPPEPPTK	1123	1137	YNL054W-B	YNL054W-B
...PPPP...	PLPDLPPEPPTKLSD	1126	1140	YNL054W-B	YNL054W-B
.[FPLWA].[WYLMFHP].[AVLIMFHPR]P..P.....	IADLPLPDLPPEPPT	1122	1136	YNL054W-B	YNL054W-B
.[FPLWA].[WYLMFHP].[AVLIMFHPR]P..P.....	LPLPDLPPEPPTKLS	1125	1139	YNL054W-B	YNL054W-B
...R..[FLIYM].[FLIYM]P.....	SLYRSGYPMPLNYSF	18	32	YNL056W	YNL056W
......P.[ILMVPYAFTR]P.[RKW]...	LMALKYPYFPTKMSY	226	240	YNL059C	ARP5
..[KRP]....P...[KR]P..	LLKKGVNPKAKRPSN	602	616	YNL061W	NOP2
...[RK].[AVLIMFHRTP]P..P.....	LPPRIVPAIPKKATV	353	367	YNL064C	YDJ1
......PR.P.R...	AGGAQRPRGPQRGKD	101	115	YNL064C	YDJ1
...R..[FLIYM].[FLIYM]P.....	RLNRGNYVMPNLVDA	116	130	YNL075W	IMP4
...R..[FLIYM].[FLIYM]P.....	FSRRQSMDIPSKNRN	513	527	YNL076W	MKS1
...PPPP...	TQSYPPTLFPPPLGT	10	24	YNL078W	NIS1
......P.[ILMVPYAFTR]P.[RKW]...	VSHSQSPKRPKKYPK	292	306	YNL080C	YNL080C
...R..[FLIYM].[FLIYM]P.....	SGMRHTLHLPVRGQH	89	103	YNL081C	SWS2
...R..[FLIYM].[FLIYM]P.....	YYNRQELALPKRMCS	386	400	YNL082W	PMS1
.[FPLWA].[WYLMFHP].[AVLIMFHPR]P..P.....	EPEWYLPILPMILVN	814	828	YNL088W	TOP2
[FPLWA]..[WYLMFHP].[AVLIMFHPP]P..P.....	WSTYIPPFNPLEIIK	837	851	YNL088W	TOP2
...[RK].[AVLIMFHRTP]P..P.....	GQERFAPLAPMYYRN	70	84	YNL093W	YPT53
...PPPP...	VRTRRRPPPPIPST	463	477	YNL094W	APP1
...PPPP...	RRRPPPPPIPSTQKP	466	480	YNL094W	APP1
...PPPP...	PPPPPIPSTQKPSLT	469	483	YNL094W	APP1
...PPPP...	VAPPPLPNRQLPNLD	505	519	YNL094W	APP1
...[RK].[AVLIMFHRTP]P..P.....	FLSKKVPNTPVVVDL	197	211	YNL094W	APP1
...[RK].[AVLIMFHRTP]P..P.....	RTRRRPPPPPIPSTQ	464	478	YNL094W	APP1
......P.[ILMVPYAFTR]P.[RKW]...	AKRVAPPPLPNRQLP	502	516	YNL094W	APP1
..[KRP]....P...[KR]P..	RPPPPPIPSTQKPSL	468	482	YNL094W	APP1
...R..[FLIYM].[FLIYM]P.....	GLCRELFFLPREGKT	388	402	YNL102W	POL1
[FPLWA]..[WYLMFHP].[AVLIMFHPP]P..P.....	WRIPYLPLPDPKDIGR	391	405	YNL104C	LEU4
...PPPP...	KLNVLPPPPPTSRHN	982	996	YNL106C	INP52
...PPPP...	VLPPPPPTSRHNKEP	985	999	YNL106C	INP52
...PPPP...	HSTPKPLPPVPALSL	1030	1044	YNL106C	INP52
...[RK].[AVLIMFHRTP]P..P.....	STPKPLPPVPALSLS	1031	1045	YNL106C	INP52
......P.[ILMVPYAFTR]P.[RKW]...	GKIVPRPCPPIRRKS	1067	1081	YNL106C	INP52
......P.[ILMVPYAFTR]P.[RKW]...	CKKKSKPKVPAKNPE	1154	1168	YNL106C	INP52
..[KRP]......P.[KR]P..	PEPAKASTKPEKPPV	1103	1117	YNL106C	INP52
..[KRP]....P...[KR]P..	STKPEKPPVVKKPHY	1109	1123	YNL106C	INP52
...PPPP...	NWLPKGPYPPPPTGI	16	30	YNL108C	YNL108C
...PPPP...	PKGPYPPPPTGIDND	19	33	YNL108C	YNL108C
...PPPP...	ANPIPTPVPPNFNAP	385	399	YNL118C	DCP2
...PPPP...	PVPPNFNAPPNPMAF	391	405	YNL118C	DCP2
.[FPLWA].[WYLMFHP].[AVLIMFHPR]P..P.....	PPNFNAPPNPMAFGV	393	407	YNL118C	DCP2
...[RK].[AVLIMFHRTP]P..P.....	EAKRKFPEAPEVPNF	456	470	YNL121C	TOM70
...PPPP...	NGMPYPPYSPFPQPT	367	381	YNL124W	NAF1
...PPPP...	PYPPYSPFPQPTNFQ	370	384	YNL124W	NAF1
...PPPP...	QSQPPLPYGVPPMNQ	427	441	YNL124W	NAF1
...PPPP...	PMYIQPPPQAPPQGN	445	459	YNL124W	NAF1
...PPPP...	IQPPPQAPPQGNGNF	448	462	YNL124W	NAF1
[FPLWA]..[WYLMFHP].[AVLIMFHPP]P..P.....	ANNYPFPQQPNGMPY	357	371	YNL124W	NAF1
.[FPLWA].[WYLMFHP].[AVLIMFHPR]P..P.....	TPAPSPPSSPQLMHL	520	534	YNL127W	FAR11

Table S6

Motif	Motif Match	Start	End	ORF	Common name
...[RK].[AVLIMFHRTP]P..P.....	EPIRYAPGDPIEKWL	472	486	YNL132W	KRE33
.[FPLWA].[WYLMFHP].[AVLIMFHPR]P..P.....	LFVLLPPIDPKDGGR	564	578	YNL132W	KRE33
....P.R.A[VP].....	SLDEPIRYAPGDPIE	469	483	YNL132W	KRE33
[FPLWA]..[WYLMFHP].[AVLIMFHPP]P..P.....	FGTFTLPADPEYKEA	308	322	YNL134C	YNL134C
..[KRP]......P.[KR]P..	QDPKKPFFTPWKPRP	415	429	YNL137C	NAM9
..[KRP]....P...[KR]P..	PKKPFFTPWKPRPFL	417	431	YNL137C	NAM9
...R..[FLIYM].[FLIYM]P.....	PKARKLINLPWQKNY	396	410	YNL137C	NAM9
...PPPP...	ATAAPAPPPPPPAPP	271	285	YNL138W	SRV2
...PPPP...	APAPPPPPPAPPASV	274	288	YNL138W	SRV2
...PPPP...	PPPPPPAPPASVFEI	277	291	YNL138W	SRV2
...PPPP...	SGPPPRPKKPSTLKT	352	366	YNL138W	SRV2
......P.[ILMVPYAFTR]P.[RKW]...	GSKSGPPPRPKKPST	349	363	YNL138W	SRV2
.[FPLWA].[WYLMFHP].[AVLIMFHPR]P..P.....	TAAPAPPPPPPAPPA	272	286	YNL138W	SRV2
.[FPLWA].[WYLMFHP].[AVLIMFHPR]P..P.....	PAPPPPPPAPPASVF	275	289	YNL138W	SRV2
.[FPLWA].[WYLMFHP].[AVLIMFHPR]P..P.....	APPPPPPAPPASVFE	276	290	YNL138W	SRV2
......P.[ILMVPYAFTR]P.[RKW]...	TYSRNEPVIPTKPSS	1276	1290	YNL139C	RLR1
......P.[ILMVPYAFTR]P.[RKW]...	HKRSELPTRPSKSKT	1444	1458	YNL139C	RLR1
...R..[FLIYM].[FLIYM]P.....	NEEREDIKLPSSALI	1175	1189	YNL139C	RLR1
...R..[FLIYM].[FLIYM]P.....	SSQRSQLRFPEKPFQ	1492	1506	YNL139C	RLR1
....RP[AS]....Y...	ELPTRPSKSKTYNDR	1448	1462	YNL139C	RLR1
...R..[FLIYM].[FLIYM]P.....	LAKRNDIILPEGFPK	31	45	YNL141W	AAH1
....RP[AVLIMFHRKTP][AVLIMFHRTP][AVLIMFHRTP]..[LP]...	GSFVRPRAVEILSKD	221	235	YNL148C	ALF1
...PPPP...	NYNQPPLPPIPTRDD	325	339	YNL152W	YNL152W
...[RK].[AVLIMFHRTP]P..P.....	MAMRPIPPLPTESEY	156	170	YNL152W	YNL152W
......P.[ILMVPYAFTR]P.[RKW]...	YNQPPLPPIPTRDDM	326	340	YNL152W	YNL152W
..[KRP]....P...[KR]P..	SRKNSMSPTRKRPPP	392	406	YNL152W	YNL152W
[FPLWA]..[WYLMFHP].[AVLIMFHPP]P..P.....	LNSPKLPPLPTTSNS	374	388	YNL152W	YNL152W
[FPLWA]..[WYLMFHP].[AVLIMFHPP]P..P.....	LHGYGHPNPPNEKSK	378	392	YNL154C	YCK2
......P.[ILMVPYAFTR]P.[RKW]...	EITHSEPAIPYRETF	743	757	YNL163C	RIA1
...[RK].[AVLIMFHRTP]P..P.....	RHNRQHPPYPHSGSP	15	29	YNL165W	YNL165W
...[RK].[AVLIMFHRTP]P..P.....	SYSRSRPTRPLGYLP	294	308	YNL165W	YNL165W
...R..[FLIYM].[FLIYM]P.....	LLQRGFFAFPSEESL	108	122	YNL165W	YNL165W
...[KR][KR][AHPKRLG]P[PLV].......	GGSKRLPPLLLSPSI	115	129	YNL167C	SKO1
....RP[AVLIMFHRKTP][AVLIMFHRTP][AVLIMFHRTP]..[LP]...	PGQQRPTIISPPILT	99	113	YNL167C	SKO1
...[KR][KR][AHPKRLG]P[PLV].......	NANRRPPVDSNNRTK	363	377	YNL175C	NOP13
...PPPP...	KARGNPPPVPPPRNV	544	558	YNL176C	YNL176C
...PPPP...	GNPPPVPPPRNVTAM	547	561	YNL176C	YNL176C
......P.[ILMVPYAFTR]P.[RKW]...	ARGNPPPVPPPRNVT	545	559	YNL176C	YNL176C
.[FPLWA].[WYLMFHP].[AVLIMFHPR]P..P.....	QAAPSPPVGPALGSK	18	32	YNL185C	MRPL19
......P.[ILMVPYAFTR]P.[RKW]...	KNELFGPSFPNKTTV	175	189	YNL197C	WHI3
[FPLWA]..[WYLMFHP].[AVLIMFHPP]P..P.....	ASFLPVPEKPNLIYL	126	140	YNL206C	RTT106
...[RK].[AVLIMFHRTP]P..P.....	FDLKNIPMMPAGEPV	463	477	YNL209W	SSB2
..[KRP]......P.[KR]P..	IARNVRTFKPFKPNN	179	193	YNL213C	YNL213C
....RP[AVLIMFHRKTP][AVLIMFHRTP][AVLIMFHRTP]..[LP]...	NLTNRPKRPGVPTPG	576	590	YNL216W	RAP1
....RP[AVLIMFHRKTP][AVLIMFHRTP][AVLIMFHRTP]..[LP]...	KWVRRPKLMTPLTFS	142	156	YNL217W	YNL217W
...R..[FLIYM].[FLIYM]P.....	CTGREWYHFPSSFLL	399	413	YNL219C	ALG9
....RP[AVLIMFHRKTP][AVLIMFHRTP][AVLIMFHRTP]..[LP]...	HMMRRPAVIKALRGE	340	354	YNL229C	URE2
......P.[ILMVPYAFTR]P.[RKW]...	KKDVTQPITPVKKRR	261	275	YNL230C	ELA1
...[KR][KR][AHPKRLG]P[PLV].......	SSPKKGPLSIKPEPV	234	248	YNL230C	ELA1
...[RK].[AVLIMFHRTP]P..P.....	SKKKEAPEDPKNLIN	6	20	YNL231C	PDR16
..[KRP]......P.[KR]P..	VYKIYNHVMPLKPVD	156	170	YNL236W	SIN4
......P.[ILMVPYAFTR]P.[RKW]...	TDPDSDPNTPKKLSS	658	672	YNL238W	KEX2
...[KR][KR][AHPKRLG]P[PLV].......	DLFKRLPVPAPPMDS	105	119	YNL238W	KEX2
[FPLWA]..[WYLMFHP].[AVLIMFHPP]P..P.....	FKRLPVPAPPMDSSL	107	121	YNL238W	KEX2
..[KRP]....P...[KR]P..	DLKSRVLPHLKKPHG	60	74	YNL241C	ZWF1
...PPPP...	RTPARTPTPTPPVVA	289	303	YNL243W	SLA2
.....P.R.P.R...	EPSVTPARTPARTPT	282	296	YNL243W	SLA2
.[FPLWA].[WYLMFHP].[AVLIMFHPR]P..P.....	VFQFQMPTTPISTKM	492	506	YNL257C	SIP3
.[FPLWA].[WYLMFHP].[AVLIMFHPR]P..P.....	EADYVIPVLPGSHLN	2032	2046	YNL262W	POL2
..[KRP]....P...[KR]P..	NDRFSHTPQQQRPMQ	17	31	YNL263C	YIF1
...PPPP...	VLSSQPPPPPPPPPP	1234	1248	YNL271C	BNI1
...PPPP...	SQPPPPPPPPPPVPA	1237	1251	YNL271C	BNI1
...PPPP...	PPPPPPPPPVPAKLF	1240	1254	YNL271C	BNI1
...PPPP...	PPPPPPVPAKLFGES	1243	1257	YNL271C	BNI1
...PPPP...	TTGDSPAPPPPPPPP	1273	1287	YNL271C	BNI1
...PPPP...	DSPAPPPPPPPPPPP	1276	1290	YNL271C	BNI1
...PPPP...	APPPPPPPPPPPPMA	1279	1293	YNL271C	BNI1
...PPPP...	PPPPPPPPPPMALFG	1282	1296	YNL271C	BNI1
...PPPP...	PPPPPPPMALFGKPK	1285	1299	YNL271C	BNI1
...PPPP...	KPKGETPPPPPLPSV	1297	1311	YNL271C	BNI1
...PPPP...	GETPPPPPLPSVLSS	1300	1314	YNL271C	BNI1
...PPPP...	GVIPPAPPMMPASQI	1318	1332	YNL271C	BNI1

Table S6

Motif	Motif Match	Start	End	ORF	Common name
...[RK].[AVLIMFHRTP]P..P.....	VLIKTLPVLPEARKK	1680	1694	YNL271C	BNI1
......P.[ILMVPYAFTR]P.[RKW]...	PPPPPPPPVPAKLFG	1241	1255	YNL271C	BNI1
.[FPLWA].[WYLMFHP].[AVLIMFHPR]P..P.....	QPPPPPPPPPPVPAK	1238	1252	YNL271C	BNI1
.[FPLWA].[WYLMFHP].[AVLIMFHPR]P..P.....	PPPPPPPPPPVPAKL	1239	1253	YNL271C	BNI1
.[FPLWA].[WYLMFHP].[AVLIMFHPR]P..P.....	SPAPPPPPPPPPPPP	1277	1291	YNL271C	BNI1
.[FPLWA].[WYLMFHP].[AVLIMFHPR]P..P.....	PAPPPPPPPPPPPPM	1278	1292	YNL271C	BNI1
.[FPLWA].[WYLMFHP].[AVLIMFHPR]P..P.....	PPPPPPPPPPPPMAL	1280	1294	YNL271C	BNI1
.[FPLWA].[WYLMFHP].[AVLIMFHPR]P..P.....	PPPPPPPPPPPMALF	1281	1295	YNL271C	BNI1
..[KRP]......P.[KR]P..	QVPMPILQQPNRPYQ	948	962	YNL278W	CAF120
.[FPLWA].[WYLMFHP].[AVLIMFHPR]P..P.....	SLLFGLPVLPHIYYL	379	393	YNL278W	CAF120
.[FPLWA].[WYLMFHP].[AVLIMFHPR]P..P.....	SAGYYRPPAPQLQNS	1021	1035	YNL278W	CAF120
[FPLWA]..[WYLMFHP].[AVLIMFHPP]P..P.....	PQAYHLPGNPYSTGN	922	936	YNL278W	CAF120
....RP[AS]....Y...	GWNNRPSPSNIYQRP	901	915	YNL278W	CAF120
..[KRP]....P...[KR]P..	LEKRGRVPLQARPTK	230	244	YNL284C	MRPL10
...PPPP...	ADLPLPDLPPEPPTK	1129	1143	YNL284C-B	YNL284C-B
...PPPP...	PLPDLPPEPPTKLSD	1132	1146	YNL284C-B	YNL284C-B
.[FPLWA].[WYLMFHP].[AVLIMFHPR]P..P.....	IADLPLPDLPPEPPT	1128	1142	YNL284C-B	YNL284C-B
.[FPLWA].[WYLMFHP].[AVLIMFHPR]P..P.....	LPLPDLPPEPPTKLS	1131	1145	YNL284C-B	YNL284C-B
.[FPLWA].[WYLMFHP].[AVLIMFHPR]P..P.....	IPKLNVPLNPKGRKL	1493	1507	YNL284C-B	YNL284C-B
......P.[ILMVPYAFTR]P.[RKW]...	LKEFNGPEPPIKRLK	145	159	YNL286W	CUS2
....P.R.A[VP].....	HQNDPLRQAVYLAIK	76	90	YNL287W	SEC21
[FPLWA]..[WYLMFHP].[AVLIMFHPP]P..P.....	PGSMSMPGVPTSMGP	27	41	YNL288W	CAF40
...[KR][KR][AHPKRLG]P[PLV].......	SSHKRKPVIPWDASI	202	216	YNL295W	YNL295W
...PPPP...	GPSPSPSPSPSPLNP	394	408	YNL298W	CLA4
...PPPP...	PSPSPSPSPLNPYRP	397	411	YNL298W	CLA4
...[RK].[AVLIMFHRTP]P..P.....	QPQRTAPKPPISAPR	452	466	YNL298W	CLA4
...[RK].[AVLIMFHRTP]P..P.....	QTMRQAPKRPDADVA	494	508	YNL298W	CLA4
..[KRP]......P.[KR]P..	PSPSPSPLNPYRPHH	399	413	YNL298W	CLA4
..[KRP]......P.[KR]P..	STPQTMRQAPKRPDA	491	505	YNL298W	CLA4
....P.R.A[VP].....	AHFQPQRTAPKPPIS	449	463	YNL298W	CLA4
..[KRP]....P...[KR]P..	SNPAYDGPLFSKPLF	267	281	YNL307C	MCK1
......P.[ILMVPYAFTR]P.[RKW]...	HPPSNVPTTPSRELN	304	318	YNL309W	STB1
......P.[ILMVPYAFTR]P.[RKW]...	SYIHQLPFIPRWQLD	499	513	YNL313C	YNL313C
......P.[ILMVPYAFTR]P.[RKW]...	PLDPSAPKVPFKTLD	110	124	YNL315C	ATP11
...[RK].[AVLIMFHRTP]P..P.....	FWTRARPIDPNAYDD	376	390	YNL317W	PFS2
...[KR][KR][AHPKRLG]P[PLV].......	YHCRRHPLALGFKYV	440	454	YNL321W	YNL321W
...R..[FLIYM].[FLIYM]P.....	AVDRCDITIPCGITD	198	212	YNL325C	FIG4
...[RK].[AVLIMFHRTP]P..P.....	DLFKPLPEPPTELGR	3	17	YNL331C	AAD14
....RP[AVLIMFHRKTP][AVLIMFHRTP][AVLIMFHRTP]..[LP]...	VNAPRPRAVVPPPNS	20	34	YNL335W	YNL335W
...[RK].[AVLIMFHRTP]P..P.....	RVTRKRPREPKSTND	821	835	YNL339C	YRF1-6
......P.[ILMVPYAFTR]P.[RKW]...	VCKLRSPNTPRRLRK	373	387	YNL339C	YRF1-6
..[KRP]......P.[KR]P..	TERLKRDLCPRKPTE	293	307	YNL339C	YRF1-6
......P.[ILMVPYAFTR]P.[RKW]...	ITRYDFPTVPARKFV	575	589	YNR006W	VPS27
...R..[FLIYM].[FLIYM]P.....	EFIRDCLVIPKEEWL	841	855	YNR011C	PRP2
....RP[AS]....Y...	EKAQRPSKYVLYQQL	821	835	YNR011C	PRP2
...R..[FLIYM].[FLIYM]P.....	LTFRELMHLPPKQWL	406	420	YNR013C	PHO91
......P.[ILMVPYAFTR]P.[RKW]...	GHASENPLLPEKLSQ	151	165	YNR016C	ACC1
....RP[AVLIMFHRKTP][AVLIMFHRTP][AVLIMFHRTP]..[LP]...	SMHLRPIATPYPVKE	1477	1491	YNR016C	ACC1
..[KRP]....P...[KR]P..	ILKQDLIPSIGKPKT	300	314	YNR027W	BUD17
...[RK].[AVLIMFHRTP]P..P.....	LKKKYAPDPPITHNV	36	50	YNR028W	CPR8
......P.[ILMVPYAFTR]P.[RKW]...	VAAGHTPQFPTKDEV	1513	1527	YNR031C	SSK2
...R..[FLIYM].[FLIYM]P.....	PSNRISLTIPVICHS	238	252	YNR034W	SOL1
...PPPP...	KYIPAPKLVPPPPRT	115	129	YNR039C	ZRG17
...PPPP...	PAPKLVPPPPRTRSP	118	132	YNR039C	ZRG17
...PPPP...	KLVPPPPRTRSPVRG	121	135	YNR039C	ZRG17
...[KR][KR][AHPKRLG]P[PLV].......	SRSRKKPVTNFTRTN	33	47	YNR045W	PET494
..[KRP]....P...[KR]P..	FFPNVNSPSVNKPVP	370	384	YNR045W	PET494
...R..[FLIYM].[FLIYM]P.....	YNERTQLFFPNVNSP	363	377	YNR045W	PET494
..[KRP]......P.[KR]P..	ATPRRASSRPTRPSA	147	161	YNR049C	MSO1
.[FPLWA].[WYLMFHP].[AVLIMFHPR]P..P.....	GPGLLAPYSPEINDP	413	427	YNR050C	LYS9
...[KR][KR][AHPKRLG]P[PLV].......	SKNKKKPVFSTVNKD	401	415	YNR051C	BRE5
...PPPP...	QSMPPIFLPPPNYLF	145	159	YNR052C	POP2
...R..[FLIYM].[FLIYM]P.....	VGFRLALKFPSRITG	109	123	YNR064C	YNR064C
.[FPLWA].[WYLMFHP].[AVLIMFHPR]P..P.....	TAYMYAPDDPRAVIE	111	125	YNR073C	YNR073C
...PPPP...	IFPPPYQPLPSHIKL	154	168	YOL006C	TOP1
...[RK].[AVLIMFHRTP]P..P.....	NLSKDAPVPPAPEGH	317	331	YOL006C	TOP1
...[RK].[AVLIMFHRTP]P..P.....	TIFKRPPKQPGHQLF	478	492	YOL006C	TOP1
......P.[ILMVPYAFTR]P.[RKW]...	VQSSSLPSPPAKKSA	69	83	YOL006C	TOP1
..[KRP]......P.[KR]P..	ISKWTIMPHPNRPNA	136	150	YOL008W	YOL008W
[FPLWA]..[WYLMFHP].[AVLIMFHPP]P..P.....	WTIMPHPNRPNAAMV	139	153	YOL008W	YOL008W
...R..[FLIYM].[FLIYM]P.....	SSARAGLQFPVGRIK	25	39	YOL012C	HTZ1
....RP[AVLIMFHRKTP][AVLIMFHRTP][AVLIMFHRTP]..[LP]...	NPADRPTATELLDDP	293	307	YOL016C	CMK2

Table S6

Motif	Motif Match	Start	End	ORF	Common name
......P.[ILMVPYAFTR]P.[RKW]...	NTLNWSPNIPLRYSD	126	140	YOL017W	ESC8
...[RK].[AVLIMFHRTP]P..P.....	LYQRTTPAGPDPSNV	468	482	YOL019W	YOL019W
.....P.R.P.R...	QREVIPHRNPSRLLN	364	378	YOL019W	YOL019W
...[KR][KR][AHPKRLG]P[PLV].......	LLTKRAPVVTIMGHV	140	154	YOL023W	IFM1
...[KR][KR][AHPKRLG]P[PLV].......	YSRKKLPVVLSKMTL	55	69	YOL024W	YOL024W
...[RK].[AVLIMFHRTP]P..P.....	EEAKSVPPIPADQAA	551	565	YOL027C	MDM38
..[KRP]....P...[KR]P..	LSKNWELPQRLKPGR	50	64	YOL028C	YAP7
......P.[ILMVPYAFTR]P.[RKW]...	IKEDIHPSLPVRTRF	35	49	YOL033W	MSE1
...[KR][KR][AHPKRLG]P[PLV].......	ELKKKAPVIAYPPSL	97	111	YOL045W	PSK2
...PPPP...	DQPAVPPSPPAEPEE	322	336	YOL047C	YOL047C
...PPPP...	AVPPSPPAEPEEPTV	325	339	YOL047C	YOL047C
...R..[FLIYM].[FLIYM]P.....	FYSRRCFLFPCKQAA	140	154	YOL052C	SPE2
....RP[AVLIMFHRKTP][AVLIMFHRTP][AVLIMFHRTP]..[LP]...	LGENRPKLVVDPVLV	116	130	YOL055C	THI20
[FPLWA]..[WYLMFHP].[AVLIMFHPP]P..P.....	AYYLGLPVTPEDMAL	182	196	YOL057W	YOL057W
...R..[FLIYM].[FLIYM]P.....	GFSRIPIFLPNEPNN	286	300	YOL060C	MAM3
...R..[FLIYM].[FLIYM]P.....	YHVRDNINLPFKVTP	331	345	YOL062C	APM4
...[RK].[AVLIMFHRTP]P..P.....	YLTRPLPSTPNEDSR	349	363	YOL070C	YOL070C
......P.[ILMVPYAFTR]P.[RKW]...	ETNDYNPTIPPRSKD	271	285	YOL070C	YOL070C
...R..[FLIYM].[FLIYM]P.....	VYERGNFKFPSYELS	97	111	YOL076W	MDM20
...[RK].[AVLIMFHRTP]P..P.....	LFERILPILPVESNL	30	44	YOL081W	IRA2
...R..[FLIYM].[FLIYM]P.....	EGFRIFFDIPSKKEL	797	811	YOL081W	IRA2
...R..[FLIYM].[FLIYM]P.....	ATPRMGIVYPGIEIL	572	586	YOL084W	PHM7
......P.[ILMVPYAFTR]P.[RKW]...	ITKILYPDIPGKNYF	26	40	YOL087C	YOL087C
...[KR][KR][AHPKRLG]P[PLV].......	YLTKRLPVTKIIKAS	809	823	YOL087C	YOL087C
...PPPP...	VKRTPPLPPVPEGMS	13	27	YOL093W	TRM10
...[RK].[AVLIMFHRTP]P..P.....	EKVKRTPPLPPVPEG	11	25	YOL093W	TRM10
...[RK].[AVLIMFHRTP]P..P.....	KVKRTPPLPPVPEGM	12	26	YOL093W	TRM10
...PPPP...	SSKVPMPPYTPPMSP	628	642	YOL100W	PKH2
...PPPP...	VPMPPYTPPMSPPMT	631	645	YOL100W	PKH2
.[FPLWA].[WYLMFHP].[AVLIMFHPR]P..P.....	MPPYTPPMSPPMTPY	633	647	YOL100W	PKH2
.[FPLWA].[WYLMFHP].[AVLIMFHPR]P..P.....	TPPMSPPMTPYDTYQ	637	651	YOL100W	PKH2
.....[GP]P.[IVL].P[FWY]...	VWSKTPPEIKPYKIN	453	467	YOL100W	PKH2
...PPPP...	ASPQPASVPPPQNGP	70	84	YOL103W-A	YOL103W-A
...PPPP...	SVPPPQNGPYPQQCM	76	90	YOL103W-A	YOL103W-A
...PPPP...	ASPQPASVPPPQNGP	70	84	YOL103W-B	YOL103W-B
...PPPP...	SVPPPQNGPYPQQCM	76	90	YOL103W-B	YOL103W-B
.[FPLWA].[WYLMFHP].[AVLIMFHPR]P..P.....	IADLPLPDLPPESPT	1128	1142	YOL103W-B	YOL103W-B
.[FPLWA].[WYLMFHP].[AVLIMFHPR]P..P.....	IPKLNVPLNPKGRKL	1493	1507	YOL103W-B	YOL103W-B
....RP[AVLIMFHRKTP][AVLIMFHRTP][AVLIMFHRTP]..[LP]...	KSFVRPAHLLVLEDH	310	324	YOL104C	NDJ1
...[RK].[AVLIMFHRTP]P..P.....	RPHRLAPSAPATKNH	306	320	YOL113W	SKM1
....P.R.A[VP].....	STFRPHRLAPSAPAT	303	317	YOL113W	SKM1
.[FPLWA].[WYLMFHP].[AVLIMFHPR]P..P.....	SPPLNLPNGPKGPSQ	462	476	YOL123W	HRP1
...R..[FLIYM].[FLIYM]P.....	IGGRLAFWMPTANDA	356	370	YOL124C	YOL124C
...[RK].[AVLIMFHRTP]P..P.....	MSLKRFPQLPSNTKH	163	177	YOL125W	YOL125W
...R..[FLIYM].[FLIYM]P.....	SLFRFPFKIPTFRGI	8	22	YOL129W	VPS68
...PPPP...	KCLDSPPPVPPSSSQ	82	96	YOL131W	YOL131W
...PPPP...	DSPPPVPPSSSQGED	85	99	YOL131W	YOL131W
...PPPP...	NEVSSLYPPPPPYVK	7	21	YOL135C	MED7
...PPPP...	SSLYPPPPPYVKFFT	10	24	YOL135C	MED7
[FPLWA]..[WYLMFHP].[AVLIMFHPP]P..P.....	LDYLIPPPMPKNQQY	62	76	YOL135C	MED7
...[RK].[AVLIMFHRTP]P..P.....	GSPRHAPSRPDSIGR	1122	1136	YOL138C	YOL138C
......P.[ILMVPYAFTR]P.[RKW]...	DSFPVDPTTPVKLGP	9	23	YOL142W	RRP40
....RP[AVLIMFHRKTP][AVLIMFHRTP][AVLIMFHRTP]..[LP]...	NEKTRPHILNLLGRA	763	777	YOL145C	CTR9
.[FPLWA].[WYLMFHP].[AVLIMFHPR]P..P.....	NLSLSVPLNPYEHRD	324	338	YOL148C	SPT20
..[KRP]......P.[KR]P..	EYKGLITIIPSKPDL	550	564	YOL152W	FRE7
..[KRP]....P...[KR]P..	ASPQCKKPQVYRPSF	61	75	YOL153C	YOL153C
...[RK].[AVLIMFHRTP]P..P.....	LYYRHRPQKPVRTVI	145	159	YOL164W	YOL164W
..[KRP]......P.[KR]P..	SLKLYYRHRPQKPVR	142	156	YOL164W	YOL164W
...[RK].[AVLIMFHRTP]P..P.....	EYSKYRPLTPSSEVY	402	416	YOR001W	RRP6
..[KRP]......P.[KR]P..	ASPMYDFLYPFRPVG	19	33	YOR002W	ALG6
...[RK].[AVLIMFHRTP]P..P.....	RKLRTVPGVPLIHLT	128	142	YOR004W	YOR004W
...PPPP...	LFRPIPPPVNPVGDI	352	366	YOR014W	RTS1
...R..[FLIYM].[FLIYM]P.....	LFGRLRLNIPTVLQT	62	76	YOR018W	ROD1
...R..[FLIYM].[FLIYM]P.....	WEVRALLNIPASLTK	335	349	YOR018W	ROD1
..[KRP]....P...[KR]P..	RERLRANPTDKKPRK	518	532	YOR023C	AHC1
......P.[ILMVPYAFTR]P.[RKW]...	DSIGTPPTTPLRTAQ	376	390	YOR025W	HST3
...[KR][KR][AHPKRLG]P[PLV].......	LSNRKLPLTSFSSHW	164	178	YOR025W	HST3
..[KRP]....P...[KR]P..	DVPNTAGPHIARPVT	108	122	YOR028C	CIN5
....RP[AVLIMFHRKTP][AVLIMFHRTP][AVLIMFHRTP]..[LP]...	PHIARPVTINNLIPP	115	129	YOR028C	CIN5
...PPPP...	IQPPDLIPGPPGHKL	31	45	YOR030W	DFG16
[FPLWA]..[WYLMFHP].[AVLIMFHPP]P..P.....	PIHLQPPVPPVYKNN	89	103	YOR032C	HMS1
......P.[ILMVPYAFTR]P.[RKW]...	LKKFVLPCLPRKNTY	340	354	YOR034C	AKR2

Table S6

Motif	Motif Match	Start	End	ORF	Common name
[FPLWA]..[WYLMFHP].[AVLIMFHPP]P..P.....	FIDYEFPHLPNELKR	178	192	YOR037W	CYC2
......P.[ILMVPYAFTR]P.[RKW]...	EEEEEHPPLPARRKS	77	91	YOR042W	CUE5
......P.[ILMVPYAFTR]P.[RKW]...	QPVRKNPEAPARRRQ	152	166	YOR042W	CUE5
......P.[ILMVPYAFTR]P.[RKW]...	DDEDVPPQLPTRTKS	334	348	YOR042W	CUE5
.[FPLWA].[WYLMFHP].[AVLIMFHPR]P..P.....	SAELWVPGLPFTFDL	302	316	YOR048C	RAT1
..R[YFLEP]..[AVLIMFHWRTP]P[GSDLIAV]P.....	GHRFSLLPSPMAYSF	94	108	YOR049C	RSB1
.[FPLWA].[WYLMFHP].[AVLIMFHPR]P..P.....	ELEPEHPVYPLQQSL	250	264	YOR051C	YOR051C
..[KRP]....P...[KR]P..	CFKIFPLPKDGKPKH	307	321	YOR056C	NOB1
...[RK].[AVLIMFHRTP]P..P.....	QESKTVPQSPKKPLS	190	204	YOR058C	ASE1
...R..[FLIYM].[FLIYM]P.....	VKPRQLFPIPLNKVD	757	771	YOR058C	ASE1
.[FPLWA].[WYLMFHP].[AVLIMFHPR]P..P.....	SAQLKPPSTPKSSLQ	272	286	YOR066W	YOR066W
......P.[ILMVPYAFTR]P.[RKW]...	VFFVILPQIPPKLTF	371	385	YOR067C	ALG8
......P.[ILMVPYAFTR]P.[RKW]...	NGGFGTPVAPTRTLS	176	190	YOR070C	GYP1
...PPPP...	TVPSNPLPLPLPGPS	289	303	YOR073W	SGO1
...PPPP...	SNPLPLPLPGPSATL	292	306	YOR073W	SGO1
...R..[FLIYM].[FLIYM]P.....	NIERLILSMPTLTTT	324	338	YOR080W	DIA2
..[KRP]......P.[KR]P..	RLKKSPLKVPSRPQF	656	670	YOR081C	STC2
...PPPP...	FGTPSPPSPPGITKS	55	69	YOR083W	WHI5
......P.[ILMVPYAFTR]P.[RKW]...	HPRFVSPRIPSRIVK	889	903	YOR086C	YOR086C
...R..[FLIYM].[FLIYM]P.....	YSNRQKLDLPELLQY	845	859	YOR086C	YOR086C
...[RK].[AVLIMFHRTP]P..P.....	RIRKEHPGEPNVIRN	335	349	YOR090C	PTC5
......P.[ILMVPYAFTR]P.[RKW]...	EISQMRPYTPLRSPN	46	60	YOR093C	YOR093C
..[KRP]......P.[KR]P..	IEPAYRNGGPVKPKL	940	954	YOR093C	YOR093C
..[KRP]......P.[KR]P..	GVRNIMIPFPNRPRI	1314	1328	YOR093C	YOR093C
...R..[FLIYM].[FLIYM]P.....	ISLRSYLDIPPVDLY	1360	1374	YOR093C	YOR093C
...[RK].[AVLIMFHRTP]P..P.....	SSLRKPPNGPSSVGL	145	159	YOR097C	YOR097C
...[KR][KR][AHPKRLG]P[PLV].......	KRKRRLPVSEDTNTK	467	481	YOR098C	NUP1
.[FPLWA].[WYLMFHP].[AVLIMFHPR]P..P.....	KPSFSFPGKPVDVQA	611	625	YOR098C	NUP1
....RP[AVLIMFHRKTP][AVLIMFHRTP][AVLIMFHRTP]..[LP]...	ENTERPPLLPILPIQ	93	107	YOR098C	NUP1
....P.R.A[VP].....	EGRSPSRVAPLVYEE	173	187	YOR104W	PIN2
[FPLWA]..[WYLMFHP].[AVLIMFHPP]P..P.....	WRIPYLPLDPKDIGR	390	404	YOR108W	LEU9
...PPPP...	IKKPVLRPPPPPAHK	958	972	YOR109W	INP53
...PPPP...	PVLRPPPPPAHKSVS	961	975	YOR109W	INP53
.[FPLWA].[WYLMFHP].[AVLIMFHPR]P..P.....	KPVLRPPPPPAHKSV	960	974	YOR109W	INP53
...R..[FLIYM].[FLIYM]P.....	NSMRPPLLIPAATTK	228	242	YOR113W	AZF1
...R..[FLIYM].[FLIYM]P.....	LAKRLEIPIPAMSEY	103	117	YOR114W	YOR114W
...[RK].[AVLIMFHRTP]P..P.....	YIWRYLPAPPVCIRP	256	270	YOR116C	RPO31
...[RK].[AVLIMFHRTP]P..P.....	FQDRSLPHFPKNSKT	827	841	YOR116C	RPO31
...[RK].[AVLIMFHRTP]P..P.....	RLKKNLPSLPTPVID	139	153	YOR127W	RGA1
...R..[FLIYM].[FLIYM]P.....	AHLRSILDLPMPKNF	292	306	YOR128C	ADE2
...[KR][KR][AHPKRLG]P[PLV].......	SRDKRAPPAVQTSKR	119	133	YOR141C	ARP8
...PPPP...	ASPQPASVPPPQNGP	70	84	YOR142W-A	YOR142W-A
...PPPP...	SVPPPQNGPYPQQCM	76	90	YOR142W-A	YOR142W-A
...PPPP...	ASPQPASVPPPQNGP	70	84	YOR142W-B	YOR142W-B
...PPPP...	SVPPPQNGPYPQQCM	76	90	YOR142W-B	YOR142W-B
.[FPLWA].[WYLMFHP].[AVLIMFHPR]P..P.....	IADLPLPDLPPESPT	1128	1142	YOR142W-B	YOR142W-B
.[FPLWA].[WYLMFHP].[AVLIMFHPR]P..P.....	IPKLNVPLNPNGRKL	1493	1507	YOR142W-B	YOR142W-B
.[FPLWA].[WYLMFHP].[AVLIMFHPR]P..P.....	ELRFLVPLVPLLVTN	315	329	YOR149C	SMP3
...[RK].[AVLIMFHRTP]P..P.....	IIGKTTPISPDEEEL	911	925	YOR151C	RPB2
....RP[AVLIMFHRKTP][AVLIMFHRTP][AVLIMFHRTP]..[LP]...	LTRLRPFVTEQPQGD	187	201	YOR152C	YOR152C
...[RK].[AVLIMFHRTP]P..P.....	QSKRARPSSPYTVSY	487	501	YOR153W	PDR5
...R..[FLIYM].[FLIYM]P.....	NRLRKRLNLPSEISI	300	314	YOR155C	ISN1
...PPPP...	EGDLPPIPPVDPNSE	622	636	YOR156C	NFI1
.[FPLWA].[WYLMFHP].[AVLIMFHPR]P..P.....	DLPPIPPVDPNSEAE	624	638	YOR156C	NFI1
....RP[AVLIMFHRKTP][AVLIMFHRTP][AVLIMFHRTP]..[LP]...	IDPWRPKAIKILIAK	86	100	YOR156C	NFI1
...PPPP...	NEKYERPPQPPPAYD	4	18	YOR161C	YOR161C
...PPPP...	YERPPQPPPAYDPNH	7	21	YOR161C	YOR161C
...[RK].[AVLIMFHRTP]P..P.....	EFFRLTPNQPVGLIK	629	643	YOR168W	GLN4
..[KRP]......P.[KR]P..	DNKVEEGSKPKKPKT	669	683	YOR168W	GLN4
..[KRP]......P.[KR]P..	LDKTCPETAPHKPYV	119	133	YOR176W	HEM15
...[RK].[AVLIMFHRTP]P..P.....	SPAKARPSFPHNDLI	10	24	YOR178C	GAC1
...PPPP...	PTTKHKAPPPPPPTA	178	192	YOR181W	LAS17
...PPPP...	KHKAPPPPPPTAETF	181	195	YOR181W	LAS17
...PPPP...	APPPPPPTAETFDSD	184	198	YOR181W	LAS17
...PPPP...	RNNRPVPPPPPMRTT	319	333	YOR181W	LAS17
...PPPP...	RPVPPPPPMRTTTEG	322	336	YOR181W	LAS17
...PPPP...	SGVRLPAPPPPPPRRG	337	351	YOR181W	LAS17
...PPPP...	RLPAPPPPPRRGPAP	340	354	YOR181W	LAS17
...PPPP...	APPPPPRRGPAPPPP	343	357	YOR181W	LAS17
...PPPP...	PPPRRGPAPPPPPHR	346	360	YOR181W	LAS17
...PPPP...	RRGPAPPPPPHRHVT	349	363	YOR181W	LAS17
...PPPP...	PAPPPPPHRHVTSNT	352	366	YOR181W	LAS17

Table S6

Motif	Motif Match	Start	End	ORF	Common name
...PPPP...	ATGRRGPAPPPPPRA	379	393	YOR181W	LAS17
...PPPP...	RRGPAPPPPPRASRP	382	396	YOR181W	LAS17
...PPPP...	PAPPPPPRASRPTPN	385	399	YOR181W	LAS17
...PPPP...	NSNMSSPPPPPVTTF	421	435	YOR181W	LAS17
...PPPP...	MSSPPPPPVTTFNTL	424	438	YOR181W	LAS17
...PPPP...	ATNVPVAPPPPPASL	463	477	YOR181W	LAS17
...PPPP...	VPVAPPPPPASLGQS	466	480	YOR181W	LAS17
...PPPP...	PSTTSAAPPPPPAFL	496	510	YOR181W	LAS17
...PPPP...	TSAAPPPPPAFLTQQ	499	513	YOR181W	LAS17
...PPPP...	PQSGGAPAPPPPPQM	514	528	YOR181W	LAS17
...PPPP...	GGAPAPPPPPQMPAT	517	531	YOR181W	LAS17
...PPPP...	PAPPPPPQMPATSTS	520	534	YOR181W	LAS17
...[RK].[AVLIMFHRTP]P..P.....	TKHKAPPPPPPTAET	180	194	YOR181W	LAS17
...[RK].[AVLIMFHRTP]P..P.....	QQNRPLPQLPNRNNR	308	322	YOR181W	LAS17
......P.[ILMVPYAFTR]P.[RKW]...	NNRPVPPPPPMRTTT	320	334	YOR181W	LAS17
......P.[ILMVPYAFTR]P.[RKW]...	GVRLPAPPPPPRRGP	338	352	YOR181W	LAS17
......P.[ILMVPYAFTR]P.[RKW]...	VRLPAPPPPPRRGPA	339	353	YOR181W	LAS17
......P.[ILMVPYAFTR]P.[RKW]...	GRRGPAPPPPPRASR	381	395	YOR181W	LAS17
..[KRP]....P...[KR]P..	QAPSMGIPQQNRPLP	300	314	YOR181W	LAS17
.[FPLWA].[WYLMFHP].[AVLIMFHPR]P..P.....	HPKHSLPPLPNQFAP	234	248	YOR181W	LAS17
.[FPLWA].[WYLMFHP].[AVLIMFHPR]P..P.....	PFPFPIPEIPSTQSA	271	285	YOR181W	LAS17
[FPLWA]..[WYLMFHP].[AVLIMFHPP]P..P.....	PSAPIPPTLPSTTSA	487	501	YOR181W	LAS17
...R..[FLIYM].[FLIYM]P.....	TVARLYIAYPDKNEW	27	41	YOR181W	LAS17
....RP[AVLIMFHRKTP][AVLIMFHRTP][AVLIMFHRTP]..[LP]...	NRNNRPVPPPPPMRT	318	332	YOR181W	LAS17
..[KRP]......P.[KR]P..	TPKVEKTEKPKKPKG	18	32	YOR182C	RPS30B
[FPLWA]..[WYLMFHP].[AVLIMFHPP]P..P.....	LHELGVPITPIAFDY	246	260	YOR184W	SER1
...PPPP...	YYNGPPPMRAPPMMS	1045	1059	YOR188W	MSB1
...[RK].[AVLIMFHRTP]P..P.....	DMAKPLPTPPTAEIR	3	17	YOR188W	MSB1
..[KRP]....P...[KR]P..	NNPPQMVPKGVRPNQ	1010	1024	YOR188W	MSB1
...[RK].[AVLIMFHRTP]P..P.....	VNEKRTPALPSNLSS	261	275	YOR191W	RIS1
......P.[ILMVPYAFTR]P.[RKW]...	NKILKRPILPSKNMD	341	355	YOR191W	RIS1
......P.[ILMVPYAFTR]P.[RKW]...	DIDTGFPPTPLKREG	812	826	YOR191W	RIS1
...PPPP...	VSPQPASVPPPQNGQ	70	84	YOR192C-A	YOR192C-A
...PPPP...	VSPQPASVPPPQNGQ	70	84	YOR192C-B	YOR192C-B
.[FPLWA].[WYLMFHP].[AVLIMFHPR]P..P.....	LPKLNVPLNPKGKKL	1508	1522	YOR192C-B	YOR192C-B
....RP[AVLIMFHRKTP][AVLIMFHRTP][AVLIMFHRTP]..[LP]...	ELYIRPPPHLGLNDK	1374	1388	YOR192C-B	YOR192C-B
...PPPP...	FSQQYAPPPGPPPMA	76	90	YOR197W	MCA1
...PPPP...	QYAPPPGPPPMAYNR	79	93	YOR197W	MCA1
...R..[FLIYM].[FLIYM]P.....	RPTRFTIMYPGSGRY	15	29	YOR197W	MCA1
.[FPLWA].[WYLMFHP].[AVLIMFHPR]P..P.....	IAIFGVPEDPNFQSS	104	118	YOR204W	DED1
...[KR][KR][AHPKRLG]P[PLV].......	LKFKKKPLHQKQLSQ	320	334	YOR208W	PTP2
.[FPLWA].[WYLMFHP].[AVLIMFHPR]P..P.....	VLALAIPEEPNSENS	225	239	YOR212W	STE4
...[KR][KR][AHPKRLG]P[PLV].......	EGEKKLPLPAKRKAS	65	79	YOR217W	RFC1
......P.[ILMVPYAFTR]P.[RKW]...	ISKKLGPDTPIKVAG	613	627	YOR219C	STE13
...[RK].[AVLIMFHRTP]P..P.....	SGYKYFPQFPQLKRI	766	780	YOR227W	YOR227W
...[RK].[AVLIMFHRTP]P..P.....	TRPKILPKIPTGAEL	1149	1163	YOR227W	YOR227W
..[KRP]......P.[KR]P..	AVPTKTIASPQRPLS	53	67	YOR227W	YOR227W
...R..[FLIYM].[FLIYM]P.....	CHLREILPIPSTLRQ	485	499	YOR227W	YOR227W
.....P.R.P.R...	PPQQLPSRKPKRGNV	320	334	YOR227W	YOR227W
......P.[ILMVPYAFTR]P.[RKW]...	QDVEGKPLRPKWIYQ	120	134	YOR229W	WTM2
...[RK].[AVLIMFHRTP]P..P.....	NTMKKRPAPPSLPSL	78	92	YOR231W	MKK1
....RP[AVLIMFHRKTP][AVLIMFHRTP][AVLIMFHRTP]..[LP]...	TMKKRPAPPSLPSLS	79	93	YOR231W	MKK1
.[FPLWA].[WYLMFHP].[AVLIMFHPR]P..P.....	SFIPMAPRTPFVTPS	16	30	YOR232W	MGE1
...[RK].[AVLIMFHRTP]P..P.....	HLQKERPKPPNKGDV	330	344	YOR233W	KIN4
...[KR][KR][AHPKRLG]P[PLV].......	INTRKAPPKFYVHYV	43	57	YOR244W	ESA1
..[KRP]......P.[KR]P..	SKKENKVGSPEKPLS	328	342	YOR244W	ESA1
...[RK].[AVLIMFHRTP]P..P.....	NYSKIFPGIPISLMT	212	226	YOR245C	DGA1
......P.[ILMVPYAFTR]P.[RKW]...	VPYSFAPFFPTKRRG	497	511	YOR254C	SEC63
.[FPLWA].[WYLMFHP].[AVLIMFHPR]P..P.....	YLKYGHPDGPQSTSH	192	206	YOR254C	SEC63
...[KR][KR][AHPKRLG]P[PLV].......	GSSKKKPLLIARFPK	72	86	YOR255W	OSW1
...PPPP...	IETNITPPPEPPSYE	46	60	YOR256C	YOR256C
...PPPP...	NITPPPEPPSYEFDI	49	63	YOR256C	YOR256C
..[KRP]....P...[KR]P..	NRPDTLDPALLRPGR	328	342	YOR259C	RPT4
...R..[FLIYM].[FLIYM]P.....	RELREVIELPLKNPE	191	205	YOR259C	RPT4
..[KRP]....P...[KR]P..	RLPKALLPIGNRPMI	63	77	YOR260W	GCD1
...[RK].[AVLIMFHRTP]P..P.....	DDLKYIPPIPQDFED	27	41	YOR264W	DSE3
..[KRP]....P...[KR]P..	QRKSLRRPTLSKPAV	128	142	YOR264W	DSE3
[FPLWA]..[WYLMFHP].[AVLIMFHPP]P..P.....	FTDMQVPKGPVCPQS	97	111	YOR264W	DSE3
....RP[AVLIMFHRKTP][AVLIMFHRTP][AVLIMFHRTP]..[LP]...	VFVSRPAPIVMPVKA	159	173	YOR264W	DSE3
...[RK].[AVLIMFHRTP]P..P.....	TSVKLHPNLPIVFVA	159	173	YOR269W	PAC1
...[KR][KR][AHPKRLG]P[PLV].......	TIRRRAPLSLLESHT	273	287	YOR272W	YTM1
......P.[ILMVPYAFTR]P.[RKW]...	ETGELLPVVPEKFLI	412	426	YOR273C	TPO4

Table S6

Motif	Motif Match	Start	End	ORF	Common name
...PPPP...	QNNPPKPQKPVPLNV	415	429	YOR290C	SNF2
....RP[AVLIMFHRKTP][AVLIMFHRTP][AVLIMFHRTP]..[LP]...	HKVLRPFLLRRLKKD	984	998	YOR290C	SNF2
...R..[FLIYM].[FLIYM]P.....	EYLREYLNLPEHIVP	74	88	YOR293W	RPS10A
......P.[ILMVPYAFTR]P.[RKW]...	EEGHLDPEAPDKLLE	106	120	YOR301W	RAX1
.[FPLWA].[WYLMFHP].[AVLIMFHPR]P..P.....	TPQPKAPRAPKVIHG	774	788	YOR304W	ISW2
[FPLWA]..[WYLMFHP].[AVLIMFHPP]P..P.....	PSLYQIPEPPRGGFQ	22	36	YOR307C	SLY41
......P.[ILMVPYAFTR]P.[RKW]...	KIGLVIPSTPSKKCK	20	34	YOR315W	YOR315W
...[RK].[AVLIMFHRTP]P..P.....	AVTRSIPRGPDKNSL	265	279	YOR322C	YOR322C
......P.[ILMVPYAFTR]P.[RKW]...	VTTSVLPQTPLKDVM	1089	1103	YOR326W	MYO2
...R..[FLIYM].[FLIYM]P.....	FVLRYYILIPHEQWD	714	728	YOR326W	MYO2
...[RK].[AVLIMFHRTP]P..P.....	QSKRARPSSPYTVSF	507	521	YOR328W	PDR10
...PPPP...	NQQQPQHLPPPPPPR	658	672	YOR329C	SCD5
...PPPP...	QPQHLPPPPPPRAQQ	661	675	YOR329C	SCD5
...PPPP...	HLPPPPPPRAQQQQQ	664	678	YOR329C	SCD5
..[KRP]......P.[KR]P..	TVPQQLPLEPLKPTA	401	415	YOR329C	SCD5
[FPLWA]..[WYLMFHP].[AVLIMFHPP]P..P.....	PQHLPPPPPPRAQQQ	662	676	YOR329C	SCD5
......P.[ILMVPYAFTR]P.[RKW]...	AKCQKLPGFPEWYRQ	480	494	YOR330C	MIP1
...[KR][KR][AHPKRLG]P[PLV].......	RMVRRRPLRVQFCAR	39	53	YOR330C	MIP1
...R..[FLIYM].[FLIYM]P.....	RYARKYMNYPIGNFF	337	351	YOR335C	ALA1
...[RK].[AVLIMFHRTP]P..P.....	GLHKWLPDIPLFELQ	753	767	YOR336W	KRE5
...[KR][KR][AHPKRLG]P[PLV].......	QGLKRAPLGLFRMSG	1218	1232	YOR336W	KRE5
...[KR][KR][AHPKRLG]P[PLV].......	GEKKKKPLVKGSLYP	233	247	YOR337W	TEA1
...[KR][KR][AHPKRLG]P[PLV].......	SYKRKHPVKSDSLLP	252	266	YOR337W	TEA1
.[FPLWA].[WYLMFHP].[AVLIMFHPR]P..P.....	FLIPSPPLSPKLRQC	78	92	YOR338W	YOR338W
...[RK].[AVLIMFHRTP]P..P.....	KLIKITPDTPFGFTW	103	117	YOR340C	RPA43
...PPPP...	VSPQPASVPPPQNGQ	70	84	YOR343W-A	YOR343W-A
...PPPP...	VSPQPASVPPPQNGQ	70	84	YOR343W-B	YOR343W-B
.[FPLWA].[WYLMFHP].[AVLIMFHPR]P..P.....	LPKLNVPLNPKGKKL	1508	1522	YOR343W-B	YOR343W-B
....RP[AVLIMFHRKTP][AVLIMFHRTP][AVLIMFHRTP]..[LP]...	ELYIRPPPHLGLNDK	1374	1388	YOR343W-B	YOR343W-B
...[RK].[AVLIMFHRTP]P..P.....	RRCKDAPIEPPKYMG	670	684	YOR346W	REV1
...[RK].[AVLIMFHRTP]P..P.....	LLSKYRPSCPIILVT	412	426	YOR347C	PYK2
...[RK].[AVLIMFHRTP]P..P.....	RYLKDHPNLPTPEPK	204	218	YOR353C	SOG2
......P.[ILMVPYAFTR]P.[RKW]...	HPKVMVPHLPGKILL	101	115	YOR357C	GRD19
...R..[FLIYM].[FLIYM]P.....	MIQREEMNMPRQYSE	27	41	YOR358W	HAP5
...[RK].[AVLIMFHRTP]P..P.....	DIIKDHPHIPNGQIF	471	485	YOR360C	PDE2
....RP[AVLIMFHRKTP][AVLIMFHRTP][AVLIMFHRTP]..[LP]...	NFAWRPRPASILSNA	657	671	YOR361C	PRT1
......P.[ILMVPYAFTR]P.[RKW]...	QLSTKKPRPPVKSKP	150	164	YOR367W	SCP1
..[KRP]....P...[KR]P..	STKKPRPPVKSKPKH	152	166	YOR367W	SCP1
...PPPP...	SSPPTSPKHTPPLNP	691	705	YOR371C	GPB1
.[FPLWA].[WYLMFHP].[AVLIMFHPR]P..P.....	SPKHTPPLNPSKKCA	696	710	YOR371C	GPB1
[FPLWA]..[WYLMFHP].[AVLIMFHPP]P..P.....	PSQPLFPNIPHSRKE	646	660	YOR371C	GPB1
...R..[FLIYM].[FLIYM]P.....	VLKRHNLWMPMITRR	208	222	YOR371C	GPB1
...[KR][KR][AHPKRLG]P[PLV].......	KVLKKPPLNTISPGQ	283	297	YOR373W	NUD1
[FPLWA]..[WYLMFHP].[AVLIMFHPP]P..P.....	AGYFVIPHTPMPSRD	218	232	YOR378W	YOR378W
...R..[FLIYM].[FLIYM]P.....	RYVRRYLTIPTIWGK	203	217	YOR381W	FRE3
......P.[ILMVPYAFTR]P.[RKW]...	KENIRYPVTPNKASL	129	143	YOR384W	FRE5
...R..[FLIYM].[FLIYM]P.....	AFLRGHYVLPALVHN	200	214	YOR384W	FRE5
...PPPP...	GDHPKGPPPPPPPDE	154	168	YOR389W	YOR389W
...PPPP...	PKGPPPPPPPDEKDR	157	171	YOR389W	YOR389W
...PPPP...	PPPPPPPDEKDRGSQ	160	174	YOR389W	YOR389W
...[RK].[AVLIMFHRTP]P..P.....	DHPKGPPPPPPPDEK	155	169	YOR389W	YOR389W
.[FPLWA].[WYLMFHP].[AVLIMFHPR]P..P.....	GAKYLAPIHPWDDYS	195	209	YOR391C	HSP33
...[RK].[AVLIMFHRTP]P..P.....	RVTRKRPREPKSTND	185	199	YOR396W	YOR396W
...PPPP...	AELQAPPPPPSSTKS	16	30	YPL004C	LSP1
...PPPP...	QAPPPPPSSTKSKFF	19	33	YPL004C	LSP1
.[FPLWA].[WYLMFHP].[AVLIMFHPR]P..P.....	AAELQAPPPPPSSTK	15	29	YPL004C	LSP1
......P.[ILMVPYAFTR]P.[RKW]...	TDEVCPPSFPSRRCE	875	889	YPL006W	NCR1
...[RK].[AVLIMFHRTP]P..P.....	EFSRPIPPTPSGFVV	64	78	YPL009C	YPL009C
[FPLWA]..[WYLMFHP].[AVLIMFHPP]P..P.....	AVLLNPPVAPTAEHV	185	199	YPL012W	RRP12
....RP[AVLIMFHRKTP][AVLIMFHRTP][AVLIMFHRTP]..[LP]...	EELMRPKVPELLLKL	959	973	YPL012W	RRP12
...PPPP...	SLTSLPPPPPPQFSE	217	231	YPL014W	YPL014W
...PPPP...	SLPPPPPPQFSEMLR	220	234	YPL014W	YPL014W
[FPLWA]..[WYLMFHP].[AVLIMFHPP]P..P.....	LTSLPPPPPPQFSEM	218	232	YPL014W	YPL014W
...R..[FLIYM].[FLIYM]P.....	NLARLKLPYPEAVFD	54	68	YPL015C	HST2
...[RK].[AVLIMFHRTP]P..P.....	KHDKLHPNYPSVKSL	130	144	YPL019C	VTC3
...R..[FLIYM].[FLIYM]P.....	TYNRTAFQIPGDQSI	421	435	YPL019C	VTC3
.[FPLWA].[WYLMFHP].[AVLIMFHPR]P..P.....	VLYPLIPNEPDDIET	59	73	YPL022W	RAD1
..[KRP]....P...[KR]P..	LDRLKKNPYAHKPFG	708	722	YPL029W	SUV3
....RP[AVLIMFHRKTP][AVLIMFHRTP][AVLIMFHRTP]..[LP]...	WIVKRPKFLNLPKNE	42	56	YPL029W	SUV3
...R..[FLIYM].[FLIYM]P.....	NMARGLIRIPETQTK	66	80	YPL038W	MET31
...PPPP...	YHHRETPPPPPSNGY	202	216	YPL055C	LGE1
...PPPP...	RETPPPPPSNGYYAK	205	219	YPL055C	LGE1

Table S6

Motif	Motif Match	Start	End	ORF	Common name
......P.[ILMVPYAFTR]P.[RKW]...	LEEDLNPDAPPKPQL	40	54	YPL057C	SUR1
.[FPLWA].[WYLMFHP].[AVLIMFHPR]P..P.....	DLNPDAPPKPQLIPK	43	57	YPL057C	SUR1
...[RK].[AVLIMFHRTP]P..P.....	LFMKGTPEFPKCGFS	49	63	YPL059W	GRX5
...PPPP...	QEPPFPDLLPPPPPP	172	186	YPL063W	TIM50
...PPPP...	PFPDLLPPPPPPPYQ	175	189	YPL063W	TIM50
...PPPP...	DLLPPPPPPPYQRPL	178	192	YPL063W	TIM50
...PPPP...	PPPPPPPYQRPLTLV	181	195	YPL063W	TIM50
..[KRP]....P...[KR]P..	LLPPPPPPPYQRPLT	179	193	YPL063W	TIM50
.[FPLWA].[WYLMFHP].[AVLIMFHPR]P..P.....	FPDLLPPPPPPPYQR	176	190	YPL063W	TIM50
[FPLWA]..[WYLMFHP].[AVLIMFHPP]P..P.....	PDLLPPPPPPPYQRP	177	191	YPL063W	TIM50
...R..[FLIYM].[FLIYM]P.....	LKPRKRMILPAWIKD	212	226	YPL064C	CWC27
..R[YFLEP]..[AVLIMFHWRTP]P[GSDLIAV]P.....	VERYTYLPSPSYFRP	173	187	YPL066W	YPL066W
....RP[AVLIMFHRKTP][AVLIMFHRTP][AVLIMFHRTP]..[LP]...	DSKTRPVTTSKLKAS	153	167	YPL068C	YPL068C
...[KR][KR][AHPKRLG]P[PLV].......	SSFRRAPVVNNEVNK	425	439	YPL072W	UBP16
......P.[ILMVPYAFTR]P.[RKW]...	LQIRYKPTPPLKKRY	332	346	YPL074W	YTA6
...[RK].[AVLIMFHRTP]P..P.....	TVTKTAPSFPQSSSK	605	619	YPL075W	GCR1
...[KR][KR][AHPKRLG]P[PLV].......	VQKRKLPLPGSIASA	406	420	YPL075W	GCR1
...[RK].[AVLIMFHRTP]P..P.....	MSAKNTPKHPVDITE	210	224	YPL082C	MOT1
...PPPP...	TANSPAPPLPPLDSK	712	726	YPL084W	BRO1
...[RK].[AVLIMFHRTP]P..P.....	EFNRNTPPQPSLLDI	527	541	YPL084W	BRO1
......P.[ILMVPYAFTR]P.[RKW]...	SVVGGPPLLPQKSAA	728	742	YPL084W	BRO1
......P.[ILMVPYAFTR]P.[RKW]...	SDLPQGPGIPPRTYE	766	780	YPL084W	BRO1
......P.[ILMVPYAFTR]P.[RKW]...	TPTMAAPPVPPKQSQ	787	801	YPL084W	BRO1
[FPLWA]..[WYLMFHP].[AVLIMFHPP]P..P.....	ANSPAPPLPPLDSKA	713	727	YPL084W	BRO1
...PPPP...	KIIESSAPPPPPIVK	2071	2085	YPL085W	SEC16
...PPPP...	ESSAPPPPPIVKRKD	2074	2088	YPL085W	SEC16
...[RK].[AVLIMFHRTP]P..P.....	TANKYAPVSPTVQQK	798	812	YPL085W	SEC16
......P.[ILMVPYAFTR]P.[RKW]...	PPTGILPLAPLRPLD	912	926	YPL085W	SEC16
.[FPLWA].[WYLMFHP].[AVLIMFHPR]P..P.....	PLAPLRPLDPLQAAT	918	932	YPL085W	SEC16
...R..[FLIYM].[FLIYM]P.....	YIRRTYLALPLGVLL	224	238	YPL087W	YDC1
[FPLWA]..[WYLMFHP].[AVLIMFHPP]P..P.....	PSTMDFPKLPSFQNS	235	249	YPL089C	RLM1
...R..[FLIYM].[FLIYM]P.....	NNGRMVIKLPNANAP	390	404	YPL089C	RLM1
...[RK].[AVLIMFHRTP]P..P.....	DDVKRTPFIPESVKN	370	384	YPL093W	NOG1
[FPLWA]..[WYLMFHP].[AVLIMFHPP]P..P.....	FSIYSIPSKPGECVL	475	489	YPL100W	ATG21
...R..[FLIYM].[FLIYM]P.....	ESNRSCLGFPDEFPN	450	464	YPL100W	ATG21
.[FPLWA].[WYLMFHP].[AVLIMFHPR]P..P.....	ELTFIAPTQPVSTIL	255	269	YPL101W	ELP4
...R..[FLIYM].[FLIYM]P.....	YNYRVPIVLPIENDE	390	404	YPL104W	MSD1
...R..[FLIYM].[FLIYM]P.....	ESIRDVIAFPKSITG	616	630	YPL104W	MSD1
...[KR][KR][AHPKRLG]P[PLV].......	FPKRKGPLPKSVLAA	169	183	YPL107W	YPL107W
..[KRP]......P.[KR]P..	TKKIAGVQVPAKPQE	68	82	YPL107W	YPL107W
...[RK].[AVLIMFHRTP]P..P.....	GLNKDVPHCPESLKW	172	186	YPL111W	CAR1
...PPPP...	TINESLPPPPAPPTF	361	375	YPL115C	BEM3
...PPPP...	ESLPPPPAPPTFFSP	364	378	YPL115C	BEM3
...PPPP...	PPPPAPPTFFSPTSS	367	381	YPL115C	BEM3
......P.[ILMVPYAFTR]P.[RKW]...	VPDLPLPTLPDRQLF	567	581	YPL115C	BEM3
.[FPLWA].[WYLMFHP].[AVLIMFHPR]P..P.....	APGLWFPNSPPPLIL	102	116	YPL128C	TBF1
...R..[FLIYM].[FLIYM]P.....	PQQREVYVLPGETAL	159	173	YPL132W	COX11
...R..[FLIYM].[FLIYM]P.....	TIRRDRYNIPICIVG	426	440	YPL133C	RDS2
...[RK].[AVLIMFHRTP]P..P.....	YMYKYLPRFPNLKRI	765	779	YPL137C	YPL137C
...[RK].[AVLIMFHRTP]P..P.....	ANPKILPKIPSGAVL	1166	1180	YPL137C	YPL137C
..[KRP]......P.[KR]P..	KSRRKTNTKPSRPST	18	32	YPL137C	YPL137C
...R..[FLIYM].[FLIYM]P.....	CHLREILPIPSTLRQ	479	493	YPL137C	YPL137C
...PPPP...	IKNRPVPPPLPPLVL	82	96	YPL140C	MKK2
...PPPP...	RPVPPPLPPLVLTQK	85	99	YPL140C	MKK2
...[RK].[AVLIMFHRTP]P..P.....	TTIKNRPVPPPLPPL	80	94	YPL140C	MKK2
..R[YFLEP]..[AVLIMFHWRTP]P[GSDLIAV]P.....	KNRPVPPPLPPLVLT	83	97	YPL140C	MKK2
....RP[AVLIMFHRKTP][AVLIMFHRTP][AVLIMFHRTP]..[LP]...	TIKNRPVPPPLPPLV	81	95	YPL140C	MKK2
..[KRP]....P...[KR]P..	SKRKPSPPSQRRPKK	761	775	YPL141C	YPL141C
...R..[FLIYM].[FLIYM]P.....	KKKRKRIFIPPKDND	146	160	YPL147W	PXA1
...R..[FLIYM].[FLIYM]P.....	GTLRDQIIYPMSSDE	692	706	YPL147W	PXA1
..[KRP]....P...[KR]P..	DGKVSPPPIRNRPLS	616	630	YPL150W	YPL150W
...[KR][KR][AHPKRLG]P[PLV].......	MSAKKPPVSDTNNNG	749	763	YPL153C	RAD53
.[FPLWA].[WYLMFHP].[AVLIMFHPR]P..P.....	MLKLARPFIPPLSRN	1	15	YPL159C	PET20
.[FPLWA].[WYLMFHP].[AVLIMFHPR]P..P.....	QALLLAPIAPHFAEY	866	880	YPL160W	CDC60
[FPLWA]..[WYLMFHP].[AVLIMFHPP]P..P.....	PGGMSIPGAPQGAMV	515	529	YPL169C	MEX67
.[FPLWA].[WYLMFHP].[AVLIMFHPR]P..P.....	IAKLVAPEMPLTEVR	243	257	YPL173W	MRPL40
...[RK].[AVLIMFHRTP]P..P.....	TEAKCLPKIPSIPIS	344	358	YPL176C	YPL176C
...R..[FLIYM].[FLIYM]P.....	DASRGGFAIPFAERV	149	163	YPL178W	CBC2
[FPLWA]..[WYLMFHP].[AVLIMFHPP]P..P.....	AGSFGAPSSPTSGIP	200	214	YPL179W	PPQ1
..[KRP]......P.[KR]P..	DFKSPLENDPNRPHI	114	128	YPL188W	POS5
......P.[ILMVPYAFTR]P.[RKW]...	VLSSNSPSPPLWNTW	58	72	YPL189W	GUP2
...PPPP...	GRYQTSIPPPPPQQQ	637	651	YPL190C	NAB3

Table S6

Motif	Motif Match	Start	End	ORF	Common name
...PPPP...	QTSIPPPPPQQQIPQ	640	654	YPL190C	NAB3
...PPPP...	IPPPPPQQQIPQGYG	643	657	YPL190C	NAB3
...PPPP...	RYQAGPPPQPPSQTP	658	672	YPL190C	NAB3
...PPPP...	AGPPPQPPSQTPMDQ	661	675	YPL190C	NAB3
...PPPP...	SAPPMSHQPPPPQQQ	757	771	YPL190C	NAB3
...[KR][KR][AHPKRLG]P[PLV].......	NDQRKPPLSDAQRRM	277	291	YPL190C	NAB3
.[FPLWA].[WYLMFHP].[AVLIMFHPR]P..P.....	APPLPVPNGPAVGPP	574	588	YPL190C	NAB3
.[FPLWA].[WYLMFHP].[AVLIMFHPR]P..P.....	SLWFCHPGDPILMQM	350	364	YPL194W	DDC1
...R..[FLIYM].[FLIYM]P.....	KSKRIPIIIPENYKI	412	426	YPL195W	APL5
......P.[ILMVPYAFTR]P.[RKW]...	SSSCILPSTPTRPLS	311	325	YPL202C	AFT2
......P.[ILMVPYAFTR]P.[RKW]...	NPLPKEPRLPKRKVA	64	78	YPL212C	PUS1
.[FPLWA].[WYLMFHP].[AVLIMFHPR]P..P.....	QAPYYFPSHPITDSY	197	211	YPL230W	YPL230W
[FPLWA]..[WYLMFHP].[AVLIMFHPP]P..P.....	LDSFSPPFSPSTTVA	299	313	YPL230W	YPL230W
...R..[FLIYM].[FLIYM]P.....	LEFRAILFIPKRAPF	315	329	YPL240C	HSP82
...[RK].[AVLIMFHRTP]P..P.....	EKRKFTPIEPSLLGP	333	347	YPL242C	IQG1
......P.[ILMVPYAFTR]P.[RKW]...	SVSKLKPSLPNKLVG	74	88	YPL242C	IQG1
..[KRP]......P.[KR]P..	PLKPKMLPIPSKPTL	529	543	YPL243W	SRP68
[FPLWA]..[WYLMFHP].[AVLIMFHPP]P..P.....	PKMLPIPSKPTLFDL	532	546	YPL243W	SRP68
....RP[AVLIMFHRKTP][AVLIMFHRTP][AVLIMFHRTP]..[LP]...	RSVKRPRRAPRPVVS	14	28	YPL245W	YPL245W
...R..[FLIYM].[FLIYM]P.....	RIFRTDYSIPTLYTP	146	160	YPL246C	RBD2
...[RK].[AVLIMFHRTP]P..P.....	KYNRQHPANPAAADD	222	236	YPL247C	YPL247C
...[RK].[AVLIMFHRTP]P..P.....	ETARKVPPIPTQIIN	248	262	YPL249C	GYP5
......P.[ILMVPYAFTR]P.[RKW]...	IPTTSSPPLPPRQNV	276	290	YPL249C	GYP5
......P.[ILMVPYAFTR]P.[RKW]...	VATSTSPKLPPRGKQ	290	304	YPL249C	GYP5
[FPLWA]..[WYLMFHP].[AVLIMFHPP]P..P.....	LRELFLPGMPGLMLM	558	572	YPL249C	GYP5
.[FPLWA].[WYLMFHP].[AVLIMFHPR]P..P.....	SPAPIVPREPLRNEP	115	129	YPL255W	BBP1
...PPPP...	ASPQPASVPPPQNGP	70	84	YPL257W-A	YPL257W-A
...PPPP...	SVPPPQNGPYPQQCM	76	90	YPL257W-A	YPL257W-A
...PPPP...	ASPQPASVPPPQNGP	70	84	YPL257W-B	YPL257W-B
...PPPP...	SVPPPQNGPYPQQCM	76	90	YPL257W-B	YPL257W-B
.[FPLWA].[WYLMFHP].[AVLIMFHPR]P..P.....	IADLPLPDLPPESPT	1128	1142	YPL257W-B	YPL257W-B
.[FPLWA].[WYLMFHP].[AVLIMFHPR]P..P.....	IPKLNVPLNPKGRKL	1493	1507	YPL257W-B	YPL257W-B
....RP[AVLIMFHRKTP][AVLIMFHRTP][AVLIMFHRTP]..[LP]...	LGENRPKLVVDPVLV	116	130	YPL258C	THI21
...R..[FLIYM].[FLIYM]P.....	GGARERMPLPLVHAF	60	74	YPL262W	FUM1
....RP[AVLIMFHRKTP][AVLIMFHRTP][AVLIMFHRTP]..[LP]...	INQPRPPRVSILMFQ	142	156	YPL266W	DIM1
...[RK].[AVLIMFHRTP]P..P.....	RRTRLRPPTPLSQLL	617	631	YPL269W	KAR9
..[KRP]......P.[KR]P..	EFKDVSFSYPTRPSV	447	461	YPL270W	MDL2
....RP[AVLIMFHRKTP][AVLIMFHRTP][AVLIMFHRTP]..[LP]...	NMLSRPRLAKLPSIR	16	30	YPL270W	MDL2
..[KRP]....P...[KR]P..	FNKTRFVPGSNKPLW	132	146	YPL272C	YPL272C
...PPPP...	GDHPKGPPPPPPPPD	154	168	YPL277C	YPL277C
...PPPP...	PKGPPPPPPPPDEKG	157	171	YPL277C	YPL277C
...PPPP...	PPPPPPPPDEKGRGS	160	174	YPL277C	YPL277C
...[RK].[AVLIMFHRTP]P..P.....	DHPKGPPPPPPPPDE	155	169	YPL277C	YPL277C
.[FPLWA].[WYLMFHP].[AVLIMFHPR]P..P.....	GAKYLAPIHPWDDYS	195	209	YPL280W	HSP32
...[RK].[AVLIMFHRTP]P..P.....	RVTRKRPREPKSTND	821	835	YPL283C	YRF1-7
......P.[ILMVPYAFTR]P.[RKW]...	VCKLRSPNTPRRLRK	373	387	YPL283C	YRF1-7
..[KRP]......P.[KR]P..	TERLKRDLCPRKPTE	293	307	YPL283C	YRF1-7
...[RK].[AVLIMFHRTP]P..P.....	VNHRYPPMAPTTVAT	509	523	YPR008W	HAA1
....RP[AS]....Y...	KPKGRPSTTCDYCKQ	34	48	YPR008W	HAA1
...[RK].[AVLIMFHRTP]P..P.....	LVLRSTPVIPDMKVA	331	345	YPR019W	CDC54
...[RK].[AVLIMFHRTP]P..P.....	DFSKNAPYNPSEVPL	112	126	YPR022C	YPR022C
......P.[ILMVPYAFTR]P.[RKW]...	SSKDQKPLVPIRIYG	313	327	YPR023C	EAF3
...[RK].[AVLIMFHRTP]P..P.....	TLLKVVPQEPTAVQR	361	375	YPR029C	APL4
......P.[ILMVPYAFTR]P.[RKW]...	VQPTAAPATPPRHIC	47	61	YPR040W	TIP41
...PPPP...	NENEPYPFPLPPPSL	475	489	YPR042C	PUF2
...PPPP...	EPYPFPLPPPSLSDS	478	492	YPR042C	PUF2
......P.[ILMVPYAFTR]P.[RKW]...	MALFENPTIPKKSYS	288	302	YPR045C	YPR045C
....RP[AVLIMFHRKTP][AVLIMFHRTP][AVLIMFHRTP]..[LP]...	GGLVRPMTLRNLLKY	309	323	YPR048W	TAH18
......P.[ILMVPYAFTR]P.[RKW]...	TRKKKDPNAPKRALS	12	26	YPR052C	NHP6A
......P.[ILMVPYAFTR]P.[RKW]...	NAATQAPLAPSRNQE	305	319	YPR055W	SEC8
......P.[ILMVPYAFTR]P.[RKW]...	GATNNAPTLPKRKNP	474	488	YPR055W	SEC8
...[KR][KR][AHPKRLG]P[PLV].......	AKLKRKPVVPRLKAK	318	332	YPR056W	TFB4
......P.[ILMVPYAFTR]P.[RKW]...	ESPDETPFFPDKIQK	78	92	YPR060C	ARO7
..[KRP]....P...[KR]P..	MNPKSSTPKIPRPKN	1	15	YPR065W	ROX1
.[FPLWA].[WYLMFHP].[AVLIMFHPR]P..P.....	IPYYSAPHDPSTRHH	201	215	YPR065W	ROX1
[FPLWA]..[WYLMFHP].[AVLIMFHPP]P..P.....	PTHHHIPHIPNQNIP	332	346	YPR065W	ROX1
......P.[ILMVPYAFTR]P.[RKW]...	FAYSFVPFPPFKLLT	41	55	YPR071W	YPR071W
...[RK].[AVLIMFHRTP]P..P.....	YNPKTVPFVPISGWN	181	195	YPR080W	TEF1
...[RK].[AVLIMFHRTP]P..P.....	ALVKFVPSKPMCVEA	398	412	YPR080W	TEF1
...R..[FLIYM].[FLIYM]P.....	SESRGFFSFPLQIAP	489	503	YPR081C	GRS2
.....[GP]P.[IVL].P[FWY]...	MLQVDGPMLTPYDVL	63	77	YPR081C	GRS2
....P.R.A[VP].....	SEERPIRLAVLGGTS	6	20	YPR084W	YPR084W

Table S6

Motif	Motif Match	Start	End	ORF	Common name
....RP[AVLIMFHRKTP][AVLIMFHRTP][AVLIMFHRTP]..[LP]...	ASEERPIRLAVLGGT	5	19	YPR084W	YPR084W
[FPLWA]..[WYLMFHP].[AVLIMFHPP]P..P.....	ARMPGMPNMPGMPNM	452	466	YPR088C	SRP54
[FPLWA]..[WYLMFHP].[AVLIMFHPP]P..P.....	PGMPNMPGMPNMPGM	455	469	YPR088C	SRP54
[FPLWA]..[WYLMFHP].[AVLIMFHPP]P..P.....	PNMPGMPNMPGMPNM	458	472	YPR088C	SRP54
[FPLWA]..[WYLMFHP].[AVLIMFHPP]P..P.....	PNMPGMPNMPGMPKV	464	478	YPR088C	SRP54
[FPLWA]..[WYLMFHP].[AVLIMFHPP]P..P.....	PGMPNMPGMPKVTPQ	467	481	YPR088C	SRP54
.[FPLWA].[WYLMFHP].[AVLIMFHPR]P..P.....	AWDMVPPYSPLSEFL	161	175	YPR089W	YPR089W
.[FPLWA].[WYLMFHP].[AVLIMFHPR]P..P.....	IPYLVLPTIPEMTDL	760	774	YPR089W	YPR089W
...[RK].[AVLIMFHRTP]P..P.....	SNRRPVPRRPSQPLN	659	673	YPR091C	YPR091C
.[FPLWA].[WYLMFHP].[AVLIMFHPR]P..P.....	RPVPRRPSQPLNTLS	662	676	YPR091C	YPR091C
...[RK].[AVLIMFHRTP]P..P.....	RNSKHAPFIPVKPAL	155	169	YPR095C	SYT1
...[RK].[AVLIMFHRTP]P..P.....	NGDRLVPTLPTVSRI	192	206	YPR095C	SYT1
......P.[ILMVPYAFTR]P.[RKW]...	FPGKKCPRLPHRNKK	344	358	YPR097W	YPR097W
...[RK].[AVLIMFHRTP]P..P.....	KKEKKPPKIPKKVYT	436	450	YPR104C	FHL1
......P.[ILMVPYAFTR]P.[RKW]...	QPIVQTPHVPDRPPS	728	742	YPR104C	FHL1
[FPLWA]..[WYLMFHP].[AVLIMFHPP]P..P.....	ADCPVVPADPDILLA	180	194	YPR110C	RPC40
...[RK].[AVLIMFHRTP]P..P.....	ENGRLTPGLPRSYKP	43	57	YPR111W	DBF20
..[KRP]....P...[KR]P..	SAKEEENPVEVKPSS	621	635	YPR112C	MRD1
...[RK].[AVLIMFHRTP]P..P.....	DEERFLPNRPHYEIQ	1312	1326	YPR117W	YPR117W
...R..[FLIYM].[FLIYM]P.....	YDSRFRIAFPGLVLT	1985	1999	YPR117W	YPR117W
....RP[AVLIMFHRKTP][AVLIMFHRTP][AVLIMFHRTP]..[LP]...	LGENRPKLVIDPVLC	138	152	YPR121W	THI22
...[RK].[AVLIMFHRTP]P..P.....	GKFRYMPFSPAGTPF	472	486	YPR135W	CTF4
.[FPLWA].[WYLMFHP].[AVLIMFHPR]P..P.....	IADLPLPDLPPESPT	1128	1142	YPR137C-B	YPR137C-B
.[FPLWA].[WYLMFHP].[AVLIMFHPR]P..P.....	IPKLNVPLNPKGRKL	1493	1507	YPR137C-B	YPR137C-B
..[KRP]......P.[KR]P..	RKRSKGEVNPSKPTV	10	24	YPR137W	RRP9
...[RK].[AVLIMFHRTP]P..P.....	ASEKGAPFFPNKTFR	135	149	YPR144C	NOC4
...R..[FLIYM].[FLIYM]P.....	LFSRGDLTLPSSKKS	66	80	YPR144C	NOC4
...R..[FLIYM].[FLIYM]P.....	LMKRFNLEYPNFYMK	326	340	YPR144C	NOC4
...[RK].[AVLIMFHRTP]P..P.....	LDEKRIPSTPIDYMA	197	211	YPR145W	ASN1
...[RK].[AVLIMFHRTP]P..P.....	YDFRSLPKVPTTQYL	53	67	YPR151C	SUE1
...PPPP...	SNGPSNLPPPPQYKA	115	129	YPR154W	PIN3
...PPPP...	ASPQPASVPPPQNGP	70	84	YPR158C-C	YPR158C-C
...PPPP...	SVPPPQNGPYPQQCM	76	90	YPR158C-C	YPR158C-C
...PPPP...	ASPQPASVPPPQNGP	70	84	YPR158C-D	YPR158C-D
...PPPP...	SVPPPQNGPYPQQCM	76	90	YPR158C-D	YPR158C-D
...PPPP...	ADLPLPDPPPEPPTE	1129	1143	YPR158C-D	YPR158C-D
...PPPP...	PLPDPPPEPPTELSD	1132	1146	YPR158C-D	YPR158C-D
.[FPLWA].[WYLMFHP].[AVLIMFHPR]P..P.....	IADLPLPDPPPEPPT	1128	1142	YPR158C-D	YPR158C-D
.[FPLWA].[WYLMFHP].[AVLIMFHPR]P..P.....	LPLPDPPPEPPTELS	1131	1145	YPR158C-D	YPR158C-D
.[FPLWA].[WYLMFHP].[AVLIMFHPR]P..P.....	IPKLNVPLNPKGRKL	1493	1507	YPR158C-D	YPR158C-D
...PPPP...	ASPQPASVPPPQNGP	70	84	YPR158W-A	YPR158W-A
...PPPP...	SVPPPQNGPYPQQCM	76	90	YPR158W-A	YPR158W-A
...PPPP...	ASPQPASVPPPQNGP	70	84	YPR158W-B	YPR158W-B
...PPPP...	SVPPPQNGPYPQQCM	76	90	YPR158W-B	YPR158W-B
.[FPLWA].[WYLMFHP].[AVLIMFHPR]P..P.....	IADLPLPDLPPESPT	1129	1143	YPR158W-B	YPR158W-B
.[FPLWA].[WYLMFHP].[AVLIMFHPR]P..P.....	IPKLNVPLNPKGRKL	1494	1508	YPR158W-B	YPR158W-B
[FPLWA]..[WYLMFHP].[AVLIMFHPP]P..P.....	PSHPQFPISPSQGQH	607	621	YPR161C	SGV1
[FPLWA]..[WYLMFHP].[AVLIMFHPP]P..P.....	FQSLLHPELPIIITG	51	65	YPR169W	JIP5
...PPPP...	NKPKPTPPSPPAKRI	301	315	YPR171W	BSP1
...[RK].[AVLIMFHRTP]P..P.....	NLKKRPPTAPQRKIS	466	480	YPR171W	BSP1
......P.[ILMVPYAFTR]P.[RKW]...	PSFEKGPRMPSRGRP	141	155	YPR171W	BSP1
......P.[ILMVPYAFTR]P.[RKW]...	SSSSSPPPLPTRRDH	181	195	YPR171W	BSP1
......P.[ILMVPYAFTR]P.[RKW]...	DGNEEKPLLPTRPNK	200	214	YPR171W	BSP1
......P.[ILMVPYAFTR]P.[RKW]...	EEKPLLPTRPNKAEV	203	217	YPR171W	BSP1
......P.[ILMVPYAFTR]P.[RKW]...	KPKPTPPSPPAKRIP	302	316	YPR171W	BSP1
......P.[ILMVPYAFTR]P.[RKW]...	LTTTSKPSLPEKPQK	330	344	YPR171W	BSP1
......P.[ILMVPYAFTR]P.[RKW]...	AAHKTKPSIPPKKVE	351	365	YPR171W	BSP1
......P.[ILMVPYAFTR]P.[RKW]...	KTKQQKPAIPQKKSF	421	435	YPR171W	BSP1
...[KR][KR][AHPKRLG]P[PLV].......	NNLKKRPPTAPQRKI	465	479	YPR171W	BSP1
..[KRP]....P...[KR]P..	FEKGPRMPSRGRPRP	143	157	YPR171W	BSP1
..[KRP]....P...[KR]P..	IKPDAVVPERVKPAP	226	240	YPR171W	BSP1
...[RK].[AVLIMFHRTP]P..P.....	ETRKLTPCSPGDDGA	369	383	YPR173C	VPS4
...R..[FLIYM].[FLIYM]P.....	FERRIYIPLPDLAAR	290	304	YPR173C	VPS4
..[KRP]....P...[KR]P..	VLPVKIQPPLLRPLA	7	21	YPR175W	DPB2
...R..[FLIYM].[FLIYM]P.....	RFKRHRLEFPFNESE	549	563	YPR175W	DPB2
..[KRP]....P...[KR]P..	RDRLGTLPPDLRPIY	274	288	YPR179C	HDA3
.[FPLWA].[WYLMFHP].[AVLIMFHPR]P..P.....	LAKLPLPRDPRVAIG	189	203	YPR183W	DPM1
....RP[AS]....Y...	PPVHRPSMSSNYGPQ	398	412	YPR185W	ATG13
..[KRP]....P...[KR]P..	DHPKLKCPICSKPCV	218	232	YPR186C	PZF1
...[RK].[AVLIMFHRTP]P..P.....	KYFKKFPKDPLAMIL	307	321	YPR189W	SKI3
...R..[FLIYM].[FLIYM]P.....	VWQRSAYFFPNNLKV	1359	1373	YPR189W	SKI3
...R..[FLIYM].[FLIYM]P.....	YENRTLYDIPEDVAY	58	72	YPR194C	OPT2

Table S6

Motif	Motif Match	Start	End	ORF	Common name
......P.[ILMVPYAFTR]P.[RKW]...	LYDVHGPVIPMKALE	380	394	YPR196W	YPR196W
..[KRP]......P.[KR]P..	TERLKRDLCPRKPIE	60	74	YPR203W	YPR203W
...[RK].[AVLIMFHRTP]P..P.....	GMPRRIPDYPDAFAG	435	449	Q0045	COX1
...R..[FLIYM].[FLIYM]P.....	ILDRYKYMLPRSLSL	774	788	Q0055	AI2
...R..[FLIYM].[FLIYM]P.....	EKIRNMLNMPSNFEE	126	140	Q0255	Q0255
