# Supplementary material for: Bayesian Modeling of the Yeast SH3 Domain Interactome Predicts Spatiotemporal Dynamics of Endocytosis Proteins
Source: PLoS Biol. 2009 Oct 20;7(10):e1000218. doi: 10.1371/journal.pbio.1000218 (PMC2756588; doi:10.1371/journal.pbio.1000218)

Table S7. SPOT intensities for yeast SH3 domain ligands predicted by regular expressions

Peptide #	Sequence	Abp1	Bbc1	Bem1-1	Bem1-2	Boi1	Boi2	Bud14	Bzz1-1
	control	1006	614	8054	9277	1501	779	1653	1791
	control	846	756	3205	6314	1482	1161	974	3402
	control	970	729	4124	7295	1157	1838	993	4348
	control	935	1106	4545	7607	1311	1681	956	5390
	control	1109	972	4890	8231	1479	1881	716	4446
	no peptide	0	0	0	0	3	1	0	0
	no peptide	0	0	0	0	0	0	0	0
	no peptide	0	0	0	0	0	0	0	0
	no peptide	0	0	0	0	0	1	0	0
	no peptide	0	0	0	0	1	2	0	0
	no peptide	0	0	1	0	0	0	0	3
	no peptide	0	0	0	0	0	0	1	0
	no peptide	0	0	0	0	3	0	1	2
	no peptide	0	0	0	0	4	0	1	0
	no peptide	0	0	0	0	4	0	0	0
	no peptide	0	0	1	0	1	0	0	0
	no peptide	0	0	2	0	4	0	0	0
	no peptide	0	0	0	0	2	2	0	0
	no peptide	0	2	0	1	0	1	0	0
	no peptide	0	0	0	0	0	2	0	0
	control	728	892	1952	3410	1129	1013	1159	1642
	control	710	796	4815	3852	1304	1161	1026	4153
	control	446	682	4467	3950	1162	1022	1108	3852
	control	593	832	4282	3825	1386	1181	887	5408
	control	661	700	6193	2694	1436	1335	1082	3794
1	LKPPIGRPPKFPKSP	133	18	0	0	3	0	0	0
2	AAAPKHAPPPVPNET	0	0	0	84	0	0	0	0
3	APKHAPPPVPNETDN	0	0	0	0	0	0	0	0
4	GPPPPLPPPLFPSSS	6	0	0	0	0	0	0	0
5	TTAPALPSLPPPPLL	0	0	0	0	0	0	0	0
6	PALPSLPPPPLLNVD	0	0	0	0	0	0	1	2
7	PHNPSPFFPPPDFND	0	0	0	0	0	0	0	0
8	AKDLIVPRRPEWNEG	766	1047	3925	5582	1449	1641	480	2633
9	TEVPIPRRPPPPQAA	0	3	3	0	0	0	0	12
10	KFIPSRPAPKPPSSA	193	316	0	7698	0	0	0	0
11	IPSRPAPKPPSSASA	54	121	2	2679	0	0	0	6
12	PPPPPPPPPPPLPQS	0	0	0	0	0	0	0	2
13	AVPPPPPPPPLPESL	0	0	0	2	0	1	0	0
14	LPPPPPPPPPPLPQ	0	0	0	6	1	0	0	3
15	PPPPPPPPPPLPQSL	0	0	0	1	0	0	0	5
16	PPLPRAPPVPPATFE	27	3	2	59	1	0	0	2
17	VNIPSPSSSPPPIPK	1	0	0	49	0	0	1	1
18	PSPSSSPPPIPKTAN	0	0	1	0	1	1	0	1
19	VPPVPLPFGIPPFPM	25	3	0	2	0	1	2	2
20	ISPPASPPPEFDFSK	0	0	0	0	0	0	0	0
21	PIVPSSPAPPLPLSG	0	0	0	0	0	0	0	1
22	QPPLPSSAPPIPTSH	0	0	0	1	0	0	0	0
23	PPIPIVPSSPAPPLP	0	0	0	0	0	0	0	0
24	HAPPLPPTAPPPPSL	0	0	0	0	0	0	0	0
25	MPAPPPPPPPPPGAF	0	0	0	0	0	0	0	0
26	VPKEPAPAPPPEPDM	0	0	0	0	0	0	0	0
27	GDHPKGPPPPPPPDE	0	1	0	3	4	1	1	1
28	DHPKGPPPPPPPDEK	0	0	8	0	0	0	1	72
29	TPRLSLPRLPNKHHW	37	2	0	3	4	0	9	0
30	SRSKPLPLTPNSKYN	109	5	0	1	2	0	5	2
31	QLMKNLPKIPLLNDI	76	0	5	0	1	1	0	1
32	RRSKSLPTTPGIRSG	229	19	1	0	3	1	20	0
33	KNRKNLPTIPIRLSG	333	25	4	16	3	2	3	0
34	RRSKSLPITPKSIFN	117	15	0	0	0	0	10	0
35	IVNKPLPPLPVAGSS	301	0	0	0	1	0	0	0
36	NENKKLPPAPTVFGL	9	0	2	0	2	0	2	3
37	EREKALPPIPFTTTL	0	0	1	0	0	0	0	0
38	DLFKPLPEPPTELGR	0	0	0	0	0	0	0	0
39	AATTSTPPLPRRRAT	109	103	0	0	0	2	1	0
40	PLQSKIPMLPSRRTM	124	29	1	0	0	0	0	0
41	SSSSTPPTLPPRRIE	19	11	0	0	0	0	0	0
42	TTNRGPPPLPPRANV	48	33	0	11	0	0	1	0
43	LKRITSPPLPPRADS	40	33	0	0	1	0	3	0
44	ISNFVPPNLPMRRFK	129	199	1	7	0	0	8	1

Table S7

Peptide #	Sequence	Abp1	Bbc1	Bem1-1	Bem1-2	Boi1	Boi2	Bud14	Bzz1-1
45	EEEEEHPPLPARRKS	0	9	0	3	0	0	0	4
46	DDEDVPPQLPTRTKS	0	5	0	3	2	1	1	0
47	QQNRPLPQLPNRNNR	228	40	3	1	1	1	4	4
48	NPLPKEPRLPKRKVA	131	221	0	1	4	2	26	6
49	VATSTSPKLPPRGKQ	23	23	6	0	0	1	0	2
50	VQPTAAPATPPRHIS	0	2	2	0	0	0	0	0
51	GATNNAPTLPKRKNP	36	6	0	9	12	58	0	0
52	SSSSSPPPLPTRRDH	0	3	8	13	4	4	0	6
53	KKAPPPVVKPKPRNF	899	2	0	0	0	3	3	10
54	QNTPPLPPKPKSPHL	16	0	0	16	3	0	0	0
55	ATKSASPTLPTRRSR	182	79	3	18	3	0	8	3
56	SKIRPTPRKPSRMAT	345	37	6	50	4	3	13	6
57	TSFKGRPKPPKTLKH	128	5	0	0	1	0	1	0
58	ALKQKKIPPPFKPHL	42	0	0	0	0	0	0	0
59	DKSRPPRPPPKPLHL	675	5	0	57	0	0	1	36
60	RPPRPPPKPLHLRTE	692	15	4	458	0	3	0	30
61	KDKSRPPRPPPKPLH	339	4	0	0	2	0	0	50
62	ERPKRRAPPPVPKKP	673	282	0	279	0	0	0	160
63	KRRAPPPVPKKPSSR	626	233	1	961	2	1	9	1318
64	PKRRAPPPVPKKPSS	648	354	0	389	2	1	1	650
65	RPKRRAPPPVPKKPS	555	487	0	497	3	1	6	480
66	RAPPPVPKKPSSRIA	673	116	0	592	0	0	3	435
67	LKHGWKPLRPIKLIS	75	14	0	7	0	0	8	0
68	NMLKKKPLKKPLKRF	303	160	0	30	11	1	33	0
69	FPPKRKPLLRPQRSD	232	266	1	19	6	1	19	0
70	DKKTKPTPPPKPSHL	410	0	0	1	3	2	1	1
71	TKPTPPPKPSHLKPK	631	1	0	0	0	0	1	0
72	KDKKTKPTPPPKPSH	190	0	1	5	0	0	4	0
73	GSKSGPPPRPKKPST	285	13	1	2	4	0	7	3
74	STKKPRPPVKSKPKH	397	3	0	2	0	1	3	1
75	VQKRKLPLPGSIASA	157	23	0	1	0	0	0	0
76	FPGKKSPRLPHRNKK	175	13	0	0	7	0	22	0
77	KKEKKPPKIPKKVYT	161	0	1	4	0	3	0	0
78	NLKKRPPTAPQRKIS	564	31	0	51	15	0	25	22
79	AAHKTKPSIPPKKVE	239	0	0	0	0	1	0	0
80	KTKQQKPAIPQKKSF	617	1	0	5	0	0	0	0
81	MTKTPETSPPKRPMG	75	0	0	3	0	0	0	3
82	QTREPNEPPPPSPAM	7	0	1	0	0	3	0	0
83	GKFIPSRPAPKPPSS	231	138	1	14622	3	4	0	2
84	DTFLPPPPPPPSNFE	0	0	0	0	1	3	0	3
85	VDFTLPPPPPPPGLD	0	0	0	0	2	0	0	1
86	DFTLPPPPPPPGLDE	0	0	0	1	0	0	0	0
87	FTLPPPPPPPGLDEL	0	0	0	0	0	0	0	7
88	DLPLPDLPPEPPTEL	0	0	3	0	2	1	0	0
89	VPPPMQPPPIESSNL	85	0	0	0	1	0	0	0
90	MFLISPPASPPPEFD	0	0	0	0	2	0	0	0
91	APPLPPTAPPPPSLP	0	0	0	89	0	0	0	0
92	NPTKSPPPPPSPSTM	28	0	1	0	0	0	2	124
93	PPIPNAPLSPAPAVP	0	0	0	0	0	0	2	0
94	MSPLGAPPPPPHKDH	0	0	0	0	0	0	0	0
95	SPLGAPPPPPHKDHL	0	0	0	0	1	0	0	0
96	RTRRRPPPPPIPSTQ	212	434	0	79	1	0	16	1474
97	GDHPKGPPPPPPPPD	0	0	0	0	0	0	1	331
98	TPEIPPPLPPKIMVH	2	0	0	0	0	0	0	0
99	RHLKLVPSTPMDIPW	37	1	189	90	0	0	8	6
100	IFLRISINFPLNYPN	212	192	8	3	24	3	18	6
101	PLRRTVMSLPGNHDI	24	0	1	6	0	0	2	1
102	NNQRRKPLLLNIGDH	56	11	0	25	5	1	17	0
103	NPNRRAPRRPLSTQH	151	294	3	476	28	0	17	0
104	NFLRLLIGLPSQNEL	55	156	1	0	122	6	22	2
105	NHSRSLFSLPGNQQS	53	35	3	353	7	2	7	2
106	ALFRLSFFFPLDPAY	142	218	0	27	192	17	12	0
107	QFDLKMPSKPIHNLK	41	0	0	0	0	2	0	0
108	RHLKNIPRIPIFSIL	49	25	823	4829	14	3	16	59
109	FLIKFTPKFPQSIDH	417	44	1	0	7	0	5	0
110	VGFRLALKFPSRITG	489	663	0	0	152	70	29	3
111	FVLRYYILIPHEQWD	0	15	2	13	6	1	1	1
112	APERAVPILPPRNNV	4	13	1	2	0	1	0	1
113	VQQDSLPKLPFRSWG	0	5	0	0	0	2	0	0
114	LPMNIQPDLPPRKIM	141	2	0	0	0	0	0	0

Table S7

Peptide #	Sequence	Abp1	Bbc1	Bem1-1	Bem1-2	Boi1	Boi2	Bud14	Bzz1-1
115	QQEQNTPPLPPKPKS	3	0	1	1	0	0	0	0
116	SSAAPPPPPRRATPE	23	8	1	1	1	0	0	0
117	APPPPPRRATPEKKP	77	14	0	3	0	1	0	1
118	EQEEIAPSLPSRNSI	0	0	1	1	0	1	0	0
119	EAEEAAPQLPSRSSA	0	0	0	0	1	1	0	2
120	LASNSPPVYPKRIRH	68	4	0	0	0	0	0	0
121	QGYPRQPQRPQRYHP	9	34	0	1	0	0	1	0
122	SAPDIPPRSPNRNAH	8	1	0	0	240	312	2	0
123	QRIRKIPGIPLMSVG	110	16	0	0	0	0	9	0
124	SSTPTTPERPKRKSG	22	559	1	0	0	2	0	1
125	AERNYAPRLPRRETS	50	18	1	0	0	0	12	0
126	EVTPKVPERPSRRKT	156	1074	0	13	0	2	3	1
127	TPTSGPPLLPPRNTM	25	0	0	0	2	0	0	0
128	ANNQGPPNLPARDKS	0	0	0	0	4	0	0	2
129	VAPPPLPNRQLPNLD	0	0	2	0	0	0	0	2
130	MAMRPIPPLPTESEY	6	0	0	0	0	1	0	0
131	YLTRPLPSTPNEDSR	0	0	0	12	0	1	1	0
132	NTMKKRPAPPSLPSL	205	15	0	56	7	2	21	60
133	RKRAPTPPAPSRSEK	274	105	1	0	1	0	6	27
134	FYHRPAPKPPVTKKV	114	45	0	4206	0	0	0	16
135	DRIRMFIRFPKFLLE	108	43	0	20	7	3	8	0
136	KMSRSPPRPPSLKII	326	98	0	567	0	0	5	2
137	NLPKRKPPKLQGPFS	202	31	0	81	2	1	12	9
138	LKVTPGRRAPTISKI	83	17	1	3	0	2	8	0
139	RKNRAPPQRPRFNRD	298	535	0	104	12	7	18	11
140	IHTRRPSSTSQYLIR	27	6	9	43	2	0	4	0
141	RKNRAPPQRPRFNRE	235	362	0	65	8	3	15	8
142	RLSRLLLSLPPESFT	57	47	0	11	7	2	50	0
143	APKREAPKPPANTSP	9	56	0	0	0	0	0	10
144	PELAPKREAPKPPAN	0	7	0	1	1	1	0	4
145	QLLNPNRRAPRRPLS	125	158	0	169	5	2	11	0
146	LEFRAILFIPKRAPF	92	22	3	1	10	4	13	0
147	VRTRRRPPPPIPST	107	115	0	109	1	1	8	1717
148	QPQRTAPKPPISAPR	42	55	0	45	0	1	2	2
149	STPQTMRQAPKRPDA	102	8	0	2	0	1	0	0
150	AHFQPQRTAPKPPIS	19	101	0	1861	0	0	1	1
151	RPVPRRPSQPLNTLS	125	423	0	75	0	1	11	0
152	IAPIPMPKPNPTHIS	44	0	0	0	0	1	0	0
153	IRMPSNPALPKKLLN	231	2	0	0	0	2	1	0
154	VFQFQMPTTPISTKM	32	0	0	5	0	0	0	1
155	QTMRQAPKRPDADVA	81	90	1	65	0	0	0	0
156	NSMRPPLLIPAATTK	87	0	5	6	0	0	13	0
157	PSTMDFPKLPSFQNS	99	0	0	8	0	0	1	0
158	PKMLPIPSKPTLFDL	173	0	0	0	0	0	0	0
159	TSIEIPKRSPLRFTS	60	12	0	0	3044	2738	5	0
160	GQYRRTIVIPRRFFT	184	258	0	17	30	6	13	3
161	RSKRLFPVRPMATAH	110	49	0	14	0	0	6	1
162	MKRSRPSRSIPYTTP	32	25	0	39	3	2	7	1
163	KKIRLIFTYPSTVGR	196	277	0	0	61	13	9	2
164	SKAKRPKFLLDLQIK	78	48	0	0	2	0	6	1
165	TKFRISLGLPVGAIM	289	43	0	0	1	0	6	0
166	RVTRKRPREPKSTND	167	217	2	0	10	2	9	0
167	WTQRRGPLVVYAEDN	0	0	0	3	1	0	0	0
168	FIRKRAPTPPAPSRS	259	68	0	800	3	0	10	52
169	KIRRSSLMIPNPQQF	105	13	0	3	2	1	5	1
170	IKTRSVPKIPSIESV	9	10	3	0	1	1	2	2
171	HVRRSILALPLGVLL	23	1	0	0	3	1	14	0
172	RTIRKLPLMSLSEYF	13	3	0	0	1	0	6	2
173	NRARYLPQNPDIIAG	5	2	0	0	3	0	11	0
174	NFKRLLPRDPSEKSS	41	51	0	11	4	2	12	1
175	LRWRHKFFLPAIAAI	0	18	0	1	1	0	10	1
176	YVRRRPRVRLEPLLS	101	273	0	15	13	17	26	0
177	KNPRRQLQIPRQQPS	92	46	2	1	0	0	6	2
178	SRYRRPLTLLLLKPF	37	140	0	10	16	0	15	0
179	LLGRKRPVMERVVDI	28	1	0	0	0	1	3	0
180	FFRRSELYLPNSSKA	22	19	3	15	3	0	10	1
181	LLRRTIPKRPFYHVL	18	184	0	108	7	2	13	0
182	NFFRKKLGIPRKLKR	99	101	1	13	14	2	43	0
183	RRLRDKYKLPIVDAD	18	14	0	2	6	0	26	0
184	TKRKRPARLIFYDSK	157	308	0	6	39	9	29	0

Table S7

Peptide #	Sequence	Abp1	Bbc1	Bem1-1	Bem1-2	Boi1	Boi2	Bud14	Bzz1-1
185	WRPRIVPILPYITRL	10	87	0	94	0	0	2	0
186	KRRTSLFPWLHKPGI	25	39	0	0	3	0	3	0
187	KLSRGRYGLPLSSRP	26	9	1	0	4	1	4	0
188	VSKLRSPNTPRRLRK	216	223	1	1	8	1	6	0
189	GKLKHIPRRPYEIER	4	13	0	0	0	0	0	0
190	RKQRSIPLSPIVPES	48	1	0	0	1	1	1	0
191	TKIRRRPQQPLTDFT	121	190	0	1	1	1	24	0
192	RSRRSSFAYPQQVAI	28	24	0	0	1	0	3	1
193	PSRRDSLSIPRAVDA	8	0	0	0	0	1	0	0
194	LSKRLPLRFPEHTSS	22	2	0	4	0	0	1	0
195	YRRKSIPFAPHQITA	12	2	0	3	0	1	0	0
196	RVRKTHVPASKRPSG	64	11	0	0	1	1	5	0
197	KRNRLKILLPFLEQS	64	46	0	0	9	3	8	0
198	NGRRRPKFRVQLSGN	209	227	0	6	16	5	23	1
199	RRRDFGAPANKRPRR	165	20	0	4	0	1	4	3
200	RKRRTHLRLPLIRSN	75	101	0	0	2	0	23	1
201	RRARRQMGIPTISQV	95	58	3	12	3	1	41	2
202	RRRIAYPFYPFKKLG	36	133	1	3	2	0	3	0
203	LRIKRKPVQTFFEFI	102	54	1	4	11	1	17	0
204	ISRRRLPVIMHRLKM	90	26	0	16	1	1	2	0
205	VRVKKRPLIRPLNSS	301	201	0	0	2	3	16	1
206	VNFRRIPTGPDSPPT	5	2	0	0	0	0	3	0
207	KRKRFNLLLPKPYLT	72	175	3	4	9	2	34	3
208	HRRRRVFSIPSLKSI	158	395	1	8	15	4	64	3
209	RRRRRPHRIERPLSN	133	515	1	5	9	0	24	2
210	RSLRKRPTSPSISGS	224	175	0	5	5	0	22	0
211	QHQQRQPKRPKRYSL	106	211	1	147	13	4	8	0
212	ALRRRLPVTRSKINW	134	245	0	7	13	1	6	0
213	GRLRPKRIAPWHLIQ	25	57	0	6	2	1	3	0
214	IKKRNKIRLPSGSPE	136	101	1	0	5	3	10	2
215	GLRRARYKFPGQQKI	47	79	0	2	3	0	9	2
216	SLSRRRFSLPSMPNV	70	98	0	53	1	1	15	3
217	AGKRRLPLVRFKASD	262	507	2	3	16	1	28	0
218	RKLRTVPGVPLIHLT	5	34	0	0	0	0	3	1
219	KRKRRLPVSEDTNTK	78	77	1	0	6	2	22	3
220	NRLRKRLNLPSEISI	36	74	0	3	12	3	7	6
221	QRKSLRRPTLSKPAV	78	37	0	1	2	0	2	4
222	TIRRRAPLSLLESHT	46	15	2	4	1	2	13	3
223	RMVRRRPLRVQFSAR	270	235	6	27	14	12	36	4
224	RSVKRPRRAPRPVVS	204	377	3	15	1	6	12	0
225	RRTRLRPPTPLSQLL	103	348	2	15	0	0	24	2
226	SNRRPVPRRPSQPLN	139	248	1	29	2	1	16	0
227	RFKRHRLEFPFNESE	36	36	1	4	0	0	4	0
228	SKSRLLIELPEGFFT	0	0	0	0	1	1	1	1
229	GKKVRPLLVLLLSRA	37	67	0	0	14	4	9	0
230	FHLNPKRSAVLYDRP	24	10	245	308	0	0	9	3
231	LLIRWLILMPLVGSR	166	339	0	4	23	4	29	3
232	ILARAILTIPRVLDK	189	35	1	0	0	0	10	0
233	VTHRLRISIPGITGR	225	30	2	4	0	0	12	0
234	GTIARPLFLVVLFFI	0	0	0	1	4	1	8	0
235	NAGIRPKFILALNDE	16	24	0	0	8	2	26	2
236	HHKRRPTTIDVPGLT	26	2	22	28	2	1	8	0
237	RLSFFFPLDPAYIRN	28	12	0	43	6	0	12	0
238	LTRRFPFLFPFDTRM	18	69	1	61	6	0	21	3
239	HGKRRAPLLLAKLDV	79	17	0	0	0	0	14	0
240	GERSRPLVISILSSA	16	0	0	0	2	0	14	3
241	LLLLFVPDSPYDPAI	0	0	0	0	1	0	3	0
242	VLRFLGFPLPIFEKG	19	30	0	2	1	1	17	0
243	ITHRLRISIPGITGR	118	24	0	6	1	1	8	1
244	NNFYRPLLRSVLVLL	14	37	0	3	6	2	14	3
245	LKQRFFLMFPKSIIW	63	23	0	1	2	1	27	0
246	SHRLWLYAAPKRPKT	47	34	0	0	1	0	5	2
247	KTRLRPAFIQQLWSS	31	17	1	0	1	0	4	0
248	HHNRRKPLEVYFKAT	10	7	43	6	0	2	10	3
249	YHSRRHPLALGFKYV	0	2	0	10	0	0	4	0
250	HKVLRPFLLRRLKKD	34	333	0	1	1	0	21	1
251	GRSRLFPHSPLGRSS	34	12	0	5	4	1	25	0
252	RRASLFPRDPKRLRP	95	165	0	7	7	8	10	1
253	SPAPIVPREPLRNEP	122	49	3187	2911	45	28	184	3071
254	VYTRTAFQIPGDDKI	0	0	0	0	0	0	0	0

Table S7

Peptide #	Sequence	Abp1	Bbc1	Bem1-1	Bem1-2	Boi1	Boi2	Bud14	Bzz1-1
255	LFMKGTPEFPKSGFS	85	1	0	0	0	0	1	0
256	PVLPPPRSPNRPTLS	133	151	1	6	849	1277	0	8
257	PSRRAPLQLPQLVNK	45	12	2	0	0	0	0	0
258	NSVPIMPTLPPRPYI	14	109	0	2	0	0	0	0
259	LYKRKPILLPDPKPL	43	2	0	0	0	0	1	0
260	MARRRLPDRPPNGIG	113	69	0	78	0	0	4	0
261	GAGERPRLVPRPINV	9	30	3	1	3	0	0	1
262	NLDRSKISLPDFDDE	23	15	3	0	7	2	20	3
263	DAPASKPSVPPRNYF	17	6	0	0	0	0	0	0
264	FYNRTAFQLPGDARV	3	0	0	1	0	0	4	2
265	AIPPPVPNRPGGTTN	7	196	1	1	1	0	2	2
266	SRQAIPPPVPNRPGG	24	247	1	3	0	0	0	3
267	NQARKPFLLPATELS	18	30	0	1	3	0	19	0
268	NRPAIRIPSLKKPAL	74	40	1	12	1	0	13	3
269	YYNRQELALPKRMSS	14	8	0	0	1	1	3	0
270	KWVRRPKLMTPLTFS	67	38	0	5	1	0	11	1
271	YSRKKLPVVLSKMTL	64	18	1	0	0	1	3	1
272	VKPRQLFPIPLNKVD	10	0	0	0	0	0	0	1
273	HPRFVSPRIPSRIVK	133	226	0	2	0	0	0	0
274	TYNRTAFQIPGDQSI	0	0	0	0	1	0	0	2
275	DGNEEKPLLPTRPNK	0	43	0	0	0	0	0	0
276	NNNKMRPHLPPLSSG	59	0	1	16	3	0	35	1
277	NILKRRPLKLEAPSK	92	272	0	0	7	3	19	2
278	FSRRQSMDIPSKNRN	127	7	7	0	0	1	6	1
279	NMLSRPRLAKLPSIR	42	54	0	18	5	2	33	0
280	NVLRKILEIPLHSFS	7	62	2	0	6	0	31	0
281	NIKVLLNPKLGKPVK	93	111	2	3	5	0	27	2
282	VSKLRSPNTPRRLRK	273	226	3	0	4	0	6	0
283	SPAPIVPREPLRNEP	106	40	3197	3314	64	50	188	3748
284	RHLKNIPRIPIFSIL	31	23	2167	2258	10	1	18	9
285	ATNVPVAPPPPPASL	0	0	4	0	0	0	0	1
286	VPVAPPPPPASLGQS	0	0	4	1	0	0	0	8
287	RNNRPVPPPPPMRTT	0	0	5	0	0	1	0	6
288	RPVPPPPPMRTTTEG	0	0	3	6	0	0	0	19
289	SGVRLPAPPPPPRRG	0	0	3	0	0	1	3	6
290	RLPAPPPPPRRGPAP	0	0	0	2	0	1	3	31
291	RRGPAPPPPPRASRP	0	0	3	11	0	0	8	45
292	PAPPPPPRASRPTPN	0	0	0	0	1	0	6	7
293	TKHKAPPPPPPTAET	0	0	3	0	0	1	0	762
294	LYEAMPPTLPHRDWK	0	0	0	1	0	2	0	0
295	QPWTDQFEKLEKEVS	0	0	0	0	0	0	2	5
	control	796	547	2367	2376	324	644	622	4513
	control	935	912	3415	1425	278	862	603	4735
	control	1016	1225	4037	4347	359	1002	868	5370
	control	1004	758	5018	4591	909	1612	800	6728
	control	992	922	3360	4500	776	1233	929	5297

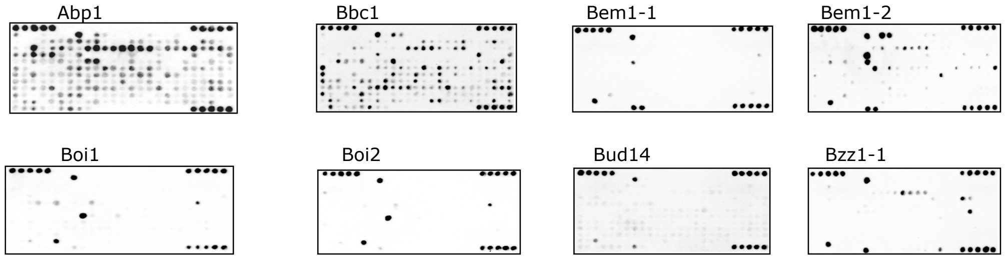

Table S7

Peptide #	Sequence	Bzz1-2	Cdc25	Cyk3	Fus1	Hof1	Hse1	Lsb1	Lsb3
	control	4259	1378	1542	1269	7526	6032	436	506
	control	6533	1266	928	1219	4293	3155	832	452
	control	4922	1172	1525	1397	4836	3053	728	337
	control	4571	1165	1213	1095	4086	2575	305	381
	control	3896	1112	1250	815	2859	2436	654	674
	no peptide	0	1	2	0	0	0	0	0
	no peptide	0	0	0	0	0	0	0	0
	no peptide	0	1	1	0	0	0	0	0
	no peptide	0	1	0	0	0	0	0	0
	no peptide	0	0	0	0	0	0	0	0
	no peptide	1	4	1	3	0	1	0	0
	no peptide	0	0	0	2	0	0	0	2
	no peptide	0	3	2	0	0	0	0	0
	no peptide	0	0	2	0	1	1	1	0
	no peptide	0	0	1	0	0	1	0	0
	no peptide	0	0	1	1	0	1	0	0
	no peptide	0	0	0	0	0	0	0	0
	no peptide	0	1	2	3	0	0	0	0
	no peptide	0	5	0	1	0	1	0	1
	no peptide	0	20	0	0	0	0	0	0
	control	5246	969	1535	1089	2962	1655	293	281
	control	2917	835	1158	1369	4783	1727	388	350
	control	2173	580	1231	1817	3557	1961	261	406
	control	5192	547	1264	1286	3802	2035	216	466
	control	6063	1012	1409	1200	4318	1692	380	349
1	LKPPIGRPPKFPKSP	0	0	0	0	0	1	0	3
2	AAAPKHAPPPVPNET	0	0	0	0	0	13	0	0
3	APKHAPPPVPNETDN	0	0	0	0	0	3	0	0
4	GPPPPLPPPLFPSSS	0	0	0	0	1686	0	64	0
5	TTAPALPSLPPPPLL	2	0	0	0	6110	0	0	0
6	PALPSLPPPPLLNVD	0	0	0	0	6722	0	0	0
7	PHNPSPFFPPPDFND	0	0	0	0	0	0	0	0
8	AKDLIVPRRPEWNEG	2661	347	2724	1227	5257	1129	636	591
9	TEVPIPRRPPPPQAA	0	0	0	0	0	0	0	46
10	KFIPSRPAPKPPSSA	10	0	0	0	0	2	692	41
11	IPSRPAPKPPSSASA	0	0	3	1	0	1	493	10
12	PPPPPPPPPPPLPQS	5	0	0	2	1	0	0	0
13	AVPPPPPPPPLPESL	1	0	2	0	1	0	0	0
14	LPPPPPPPPPPPLPQ	0	1	1	0	1	0	0	0
15	PPPPPPPPPPLPQSL	2	0	1	0	5	0	0	0
16	PPLPRAPPVPPATFE	4	1	0	0	183	0	7	0
17	VNIPSPSSSPPPIPK	0	0	0	0	0	8	0	0
18	PSPSSSPPPIPKTAN	3	1	0	0	0	0	0	0
19	VPPVPLPFGIPPFPM	0	8	2	0	84	0	6	4
20	ISPPASPPPEFDFSK	0	0	1	0	0	0	0	0
21	PIVPSSPAPPLPLSG	0	0	0	1	0	0	0	0
22	QPPLPSSAPPIPTSH	0	0	0	0	0	0	0	0
23	PPIPIVPSSPAPPLP	0	0	0	0	0	0	0	0
24	HAPPLPPTAPPPPSL	0	0	0	0	11	0	0	0
25	MPAPPPPPPPPPGAF	1	0	0	0	0	0	0	0
26	VPKEPAPAPPPEPDM	0	0	1	1	0	3	0	0
27	GDHPKGPPPPPPPDE	0	3	0	0	0	0	0	0
28	DHPKGPPPPPPPDEK	12	6	0	0	2	0	0	0
29	TPRLSLPRLPNKHHW	1	10	0	0	1596	30	0	0
30	SRSKPLPLTPNSKYN	4	2	1	0	0	1	0	0
31	QLMKNLPKIPLLNDI	7	0	0	0	11	1	6	21
32	RRSKSLPTTPGIRSG	1	17	0	63	0	5	8	1
33	KNRKNLPTIPIRLSG	4	5	1	24	26	2	1220	25
34	RRSKSLPITPKSIFN	0	15	0	26	0	0	0	0
35	IVNKPLPPLPVAGSS	2	0	0	0	1443	59	0	2
36	NENKKLPPAPTVFGL	0	4	1	0	25	0	0	1
37	EREKALPPIPFTTTL	0	0	0	0	354	1	16	4
38	DLFKPLPEPPTELGR	0	0	0	0	2	1	0	6
39	AATTSTPPLPRRRAT	0	4	1	0	5	2	243	877
40	PLQSKIPMLPSRRTM	0	0	2	0	186	3	396	635
41	SSSSTPPTLPPRRIE	0	2	0	0	5	9	1359	1091
42	TTNRGPPPLPPRANV	8	0	0	0	425	750	1107	892
43	LKRITSPPLPPRADS	6	0	0	0	520	38	892	566
44	ISNFVPPNLPMRRFK	19	0	0	0	5	72	63	588

Table S7

Peptide #	Sequence	Bzz1-2	Cdc25	Cyk3	Fus1	Hof1	Hse1	Lsb1	Lsb3
45	EEEEEHPPLPARRKS	3	0	0	0	0	0	32	683
46	DDEDVPPQLPTRTKS	0	0	0	0	0	2	58	556
47	QQNRPLPQLPNRNNR	0	2	0	2	112	23	176	484
48	NPLPKEPRLPKRKVA	29	22	4	2	5	191	19	282
49	VATSTSPKLPPRGKQ	0	0	1	0	2	0	561	695
50	VQPTAAPATPPRHIS	0	2	0	13	0	0	45	251
51	GATNNAPTLPKRKNP	0	1	0	2	0	1	64	425
52	SSSSSPPPLPTRRDH	3	15	1	1	16	4	463	485
53	KKAPPPVVKPKPRNF	360	15	5	1	10	0	4	0
54	QNTPPLPPKPKSPHL	2	0	0	0	1322	634	0	249
55	ATKSASPTLPTRRSR	1	22	1	2	0	2	56	834
56	SKIRPTPRKPSRMAT	16	35	6	12	8	19	10	124
57	TSFKGRPKPPKTLKH	11	2	2	1	1	2	0	0
58	ALKQKKIPPPFKPHL	0	0	0	0	0	0	0	0
59	DKSRPPRPPPKPLHL	1	0	0	0	0	36	4	216
60	RPPRPPPKPLHLRTE	38	0	0	0	3	539	266	306
61	KDKSRPPRPPPKPLH	0	2	0	0	0	20	7	162
62	ERPKRRAPPPVPKKP	791	4	0	0	0	57	326	188
63	KRRAPPPVPKKPSSR	4500	9	4	10	6	412	1854	389
64	PKRRAPPPVPKKPSS	3419	3	1	6	8	368	1736	346
65	RPKRRAPPPVPKKPS	2582	12	2	6	2	310	1454	298
66	RAPPPVPKKPSSRIA	590	2	0	0	4	857	1599	415
67	LKHGWKPLRPIKLIS	1	14	0	4	0	147	0	0
68	NMLKKKPLKKPLKRF	46	68	2	36	14	187	0	2
69	FPPKRKPLLRPQRSD	28	27	3	6	4	51	69	48
70	DKKTKPTPPPKPSHL	0	1	0	0	3	4	0	24
71	TKPTPPPKPSHLKPK	0	2	1	2	3	11	0	75
72	KDKKTKPTPPPKPSH	1	2	2	4	0	2	0	2
73	GSKSGPPPRPKKPST	11	0	3	0	0	38	0	55
74	STKKPRPPVKSKPKH	5	0	3	1	0	1	0	0
75	VQKRKLPLPGSIASA	0	18	0	0	0	0	0	1
76	FPGKKSPRLPHRNKK	5	35	3	0	1	8	5	155
77	KKEKKPPKIPKKVYT	18	3	1	3	4	12	0	0
78	NLKKRPPTAPQRKIS	324	203	2	8	28	157	8	45
79	AAHKTKPSIPPKKVE	0	0	0	1	0	0	0	0
80	KTKQQKPAIPQKKSF	9	3	0	0	0	6	0	4
81	MTKTPETSPPKRPMG	4	2	0	0	16	0	3	34
82	QTREPNEPPPPSPAM	0	0	1	0	0	0	0	2
83	GKFIPSRPAPKPPSS	52	4	2	0	0	0	360	21
84	DTFLPPPPPPPSNFE	0	0	0	0	0	0	0	0
85	VDFTLPPPPPPPGLD	1	0	0	0	143	0	0	0
86	DFTLPPPPPPPGLDE	0	0	0	0	6	0	0	0
87	FTLPPPPPPPGLDEL	0	0	2	0	116	0	0	0
88	DLPLPDLPPEPPTEL	0	2	0	0	566	0	0	0
89	VPPPMQPPPIESSNL	0	0	1	1	0	0	99	0
90	MFLISPPASPPPEFD	0	2	1	0	1	0	0	0
91	APPLPPTAPPPPSLP	0	2	0	0	51	12	0	0
92	NPTKSPPPPPSPSTM	253	3	1	1	0	0	0	0
93	PPIPNAPLSPAPAVP	1	2	0	0	0	0	0	0
94	MSPLGAPPPPPHKDH	1	0	0	0	0	0	0	0
95	SPLGAPPPPPHKDHL	0	0	0	0	0	0	0	0
96	RTRRRPPPPPIPSTQ	2961	3	3	13	8	63	800	56
97	GDHPKGPPPPPPPPD	0	0	0	0	0	0	0	0
98	TPEIPPPLPPKIMVH	0	2	0	0	2235	1541	0	96
99	RHLKLVPSTPMDIPW	411	14	0	1432	59	134	14	0
100	IFLRISINFPLNYPN	70	91	5	655	15	143	71	10
101	PLRRTVMSLPGNHDI	1	16	0	1	0	1	75	0
102	NNQRRKPLLLNIGDH	5	216	1	28	18	50	0	0
103	NPNRRAPRRPLSTQH	56	221	3	59	70	113	52	2
104	NFLRLLIGLPSQNEL	0	354	9	243	3	10	2	0
105	NHSRSLFSLPGNQQS	19	333	0	653	364	114	46	0
106	ALFRLSFFFPLDPAY	31	359	13	628	33	116	49	16
107	QFDLKMPSKPIHNLK	0	1	0	0	0	0	1	9
108	RHLKNIPRIPIFSIL	394	230	1	1870	5556	307	12	1
109	FLIKFTPKFPQSIDH	9	209	0	100	24	25	46	32
110	VGFRLALKFPSRITG	109	490	25	430	25	113	452	166
111	FVLRYYILIPHEQWD	2	59	0	42	5	2	2	0
112	APERAVPILPPRNNV	0	1	0	0	63	1	290	771
113	VQQDSLPKLPFRSWG	0	0	0	1	1568	0	399	646
114	LPMNIQPDLPPRKIM	0	0	0	0	0	3	285	941

Table S7

Peptide #	Sequence	Bzz1-2	Cdc25	Cyk3	Fus1	Hof1	Hse1	Lsb1	Lsb3
115	QQEQNTPPLPPKPKS	3	0	0	0	950	184	0	923
116	SSAAPPPPPRRATPE	1	2	0	0	1	0	266	706
117	APPPPPRRATPEKKP	0	3	1	0	0	0	30	432
118	EQEEIAPSLPSRNSI	1	1	1	0	2	0	20	998
119	EAEEAAPQLPSRSSA	2	2	0	1	0	0	62	956
120	LASNSPPVYPKRIRH	0	0	0	0	1	1	0	167
121	QGYPRQPQRPQRYHP	0	0	1	0	3	3	1	46
122	SAPDIPPRSPNRNAH	0	0	0	0	0	0	857	207
123	QRIRKIPGIPLMSVG	13	2	2	6	13	7	78	51
124	SSTPTTPERPKRKSG	1	1	1	0	0	0	316	442
125	AERNYAPRLPRRETS	1	11	0	0	0	4	352	371
126	EVTPKVPERPSRRKT	1	10	1	0	28	245	824	271
127	TPTSGPPLLPPRNTM	0	0	1	1	3	4	456	345
128	ANNQGPPNLPARDKS	0	0	0	0	0	0	461	478
129	VAPPPLPNRQLPNLD	0	0	2	0	1331	0	1379	743
130	MAMRPIPPLPTESEY	2	0	0	0	1557	0	174	131
131	YLTRPLPSTPNEDSR	0	0	0	0	0	6	8	139
132	NTMKKRPAPPSLPSL	1440	58	1	0	489	53	52	12
133	RKRAPTPPAPSRSEK	946	15	1	14	0	3	1289	8
134	FYHRPAPKPPVTKKV	186	0	0	0	0	78	63	5
135	DRIRMFIRFPKFLLE	0	77	2	39	21	17	4	6
136	KMSRSPPRPPSLKII	350	9	0	0	0	14	140	54
137	NLPKRKPPKLQGPFS	49	53	1	6	24	440	0	4
138	LKVTPGRRAPTISKI	1	13	1	0	0	0	0	0
139	RKNRAPPQRPRFNRD	91	40	12	26	8	23	670	68
140	IHTRRPSSTSQYLIR	7	10	1	233	11	22	0	0
141	RKNRAPPQRPRFNRE	77	30	4	14	6	25	390	30
142	RLSRLLLSLPPESFT	4	172	2	81	89	59	6	0
143	APKREAPKPPANTSP	0	3	0	0	0	0	0	0
144	PELAPKREAPKPPAN	4	0	1	0	0	0	0	0
145	QLLNPNRRAPRRPLS	9	16	1	1	3	20	56	43
146	LEFRAILFIPKRAPF	0	29	1	87	5	6	35	26
147	VRTRRRPPPPIPST	4026	0	1	0	6	29	171	38
148	QPQRTAPKPPISAPR	2	3	0	0	0	0	3	2
149	STPQTMRQAPKRPDA	2	0	1	0	0	0	24	45
150	AHFQPQRTAPKPPIS	0	5	0	0	0	0	17	0
151	RPVPRRPSQPLNTLS	13	37	1	0	13	34	94	138
152	IAPIPMPKPNPTHIS	0	0	1	0	0	0	2	0
153	IRMPSNPALPKKLLN	2	4	0	0	6	16	47	53
154	VFQFQMPTTPISTKM	0	0	0	0	0	0	1	14
155	QTMRQAPKRPDADVA	28	0	0	0	0	1	211	60
156	NSMRPPLLIPAATTK	0	37	0	0	2	5	387	25
157	PSTMDFPKLPSFQNS	0	0	2	0	307	0	44	21
158	PKMLPIPSKPTLFDL	0	0	0	0	0	0	2	15
159	TSIEIPKRSPLRFTS	0	13	0	0	0	0	0	5
160	GQYRRTIVIPRRFFT	0	156	4	48	8	11	19	16
161	RSKRLFPVRPMATAH	13	22	0	17	7	12	5	4
162	MKRSRPSRSIPYTTP	29	9	1	5	0	14	0	1
163	KKIRLIFTYPSTVGR	2	163	10	73	0	4	50	20
164	SKAKRPKFLLDLQIK	1	26	4	3	2	2	7	2
165	TKFRISLGLPVGAIM	2	108	0	32	0	8	44	28
166	RVTRKRPREPKSTND	11	28	4	34	3	12	9	5
167	WTQRRGPLVVYAEDN	0	1	0	2	3	1	0	0
168	FIRKRAPTPPAPSRS	522	43	2	13	6	76	1189	18
169	KIRRSSLMIPNPQQF	0	12	2	21	0	1	12	14
170	IKTRSVPKIPSIESV	2	2	0	0	38	0	266	257
171	HVRRSILALPLGVLL	0	20	2	16	0	0	47	0
172	RTIRKLPLMSLSEYF	0	6	1	24	20	2	15	3
173	NRARYLPQNPDIIAG	0	9	0	0	4	3	1	1
174	NFKRLLPRDPSEKSS	10	50	3	145	15	72	0	0
175	LRWRHKFFLPAIAAI	0	26	4	53	0	21	0	0
176	YVRRRPRVRLEPLLS	15	156	4	19	20	54	13	0
177	KNPRRQLQIPRQQPS	2	54	0	0	2	3	216	3
178	SRYRRPLTLLLLKPF	4	236	3	15	15	15	7	2
179	LLGRKRPVMERVVDI	4	9	0	0	0	9	4	4
180	FFRRSELYLPNSSKA	5	115	0	37	25	43	2	0
181	LLRRTIPKRPFYHVL	13	44	0	14	52	43	139	2
182	NFFRKKLGIPRKLKR	7	138	4	219	29	130	234	1
183	RRLRDKYKLPIVDAD	2	18	1	58	9	9	48	0
184	TKRKRPARLIFYDSK	5	61	7	29	4	11	4	6

Table S7

Peptide #	Sequence	Bzz1-2	Cdc25	Cyk3	Fus1	Hof1	Hse1	Lsb1	Lsb3
185	WRPRIVPILPYITRL	0	14	0	8	770	11	22	0
186	KRRTSLFPWLHKPGI	3	18	0	31	5	19	0	0
187	KLSRGRYGLPLSSRP	7	12	0	3	8	10	175	4
188	VSKLRSPNTPRRLRK	22	55	2	0	6	8	10	72
189	GKLKHIPRRPYEIER	2	0	0	0	1	1	5	0
190	RKQRSIPLSPIVPES	5	1	0	17	0	0	2	6
191	TKIRRRPQQPLTDFT	6	27	1	6	0	1	30	4
192	RSRRSSFAYPQQVAI	1	10	0	26	4	5	0	2
193	PSRRDSLSIPRAVDA	0	0	1	0	0	0	1	1
194	LSKRLPLRFPEHTSS	7	3	0	1	25	101	1	0
195	YRRKSIPFAPHQITA	5	2	0	2	5	9	8	0
196	RVRKTHVPASKRPSG	0	0	3	5	0	2	0	0
197	KRNRLKILLPFLEQS	0	28	14	169	6	25	34	2
198	NGRRRPKFRVQLSGN	0	87	8	75	24	53	114	8
199	RRRDFGAPANKRPRR	7	11	3	8	0	5	0	10
200	RKRRTHLRLPLIRSN	0	59	8	55	0	6	3	0
201	RRARRQMGIPTISQV	25	120	2	1	3	11	35	6
202	RRRIAYPFYPFKKLG	6	85	1	14	8	13	4	0
203	LRIKRKPVQTFFEFI	0	271	1	16	7	25	4	0
204	ISRRRLPVIMHRLKM	5	51	0	15	9	17	137	2
205	VRVKKRPLIRPLNSS	11	127	2	6	0	14	9	6
206	VNFRRIPTGPDSPPT	3	0	0	0	3	4	0	2
207	KRKRFNLLLPKPYLT	6	32	3	64	4	12	0	0
208	HRRRRVFSIPSLKSI	5	131	5	136	17	19	114	5
209	RRRRRPHRIERPLSN	5	28	5	43	4	16	0	8
210	RSLRKRPTSPSISGS	21	52	1	44	13	55	26	14
211	QHQQRQPKRPKRYSL	17	22	1	55	3	33	5	36
212	ALRRRLPVTRSKINW	7	46	2	25	14	35	46	4
213	GRLRPKRIAPWHLIQ	4	0	1	6	1	9	0	0
214	IKKRNKIRLPSGSPE	4	18	9	52	6	6	104	0
215	GLRRARYKFPGQQKI	1	15	0	14	6	12	71	0
216	SLSRRRFSLPSMPNV	17	35	1	27	77	97	1050	4
217	AGKRRLPLVRFKASD	2	55	9	23	7	9	18	16
218	RKLRTVPGVPLIHLT	0	0	0	2	2	0	0	2
219	KRKRRLPVSEDTNTK	1	16	8	150	0	4	1	8
220	NRLRKRLNLPSEISI	13	42	8	321	45	99	459	3
221	QRKSLRRPTLSKPAV	16	7	4	2	4	10	32	3
222	TIRRRAPLSLLESHT	8	13	3	3	4	16	29	0
223	RMVRRRPLRVQFSAR	16	94	12	92	10	41	60	35
224	RSVKRPRRAPRPVVS	3	20	12	19	6	8	94	52
225	RRTRLRPPTPLSQLL	0	24	7	17	5	17	215	2
226	SNRRPVPRRPSQPLN	60	35	0	0	18	9	576	183
227	RFKRHRLEFPFNESE	7	142	0	34	9	32	53	0
228	SKSRLLIELPEGFFT	0	66	0	0	0	0	0	0
229	GKKVRPLLVLLLSRA	0	132	0	4	0	0	0	6
230	FHLNPKRSAVLYDRP	38	25	0	309	83	40	0	0
231	LLIRWLILMPLVGSR	7	176	4	168	12	25	47	6
232	ILARAILTIPRVLDK	2	36	0	9	0	5	23	8
233	VTHRLRISIPGITGR	0	19	0	14	0	5	382	8
234	GTIARPLFLVVLFFI	0	18	1	9	0	0	0	0
235	NAGIRPKFILALNDE	6	22	1	0	1	2	0	0
236	HHKRRPTTIDVPGLT	13	42	0	180	6	3	1	3
237	RLSFFFPLDPAYIRN	3	28	1	159	48	91	0	0
238	LTRRFPFLFPFDTRM	3	21	3	100	116	126	0	0
239	HGKRRAPLLLAKLDV	1	7	0	0	0	0	0	0
240	GERSRPLVISILSSA	1	16	0	3	0	0	0	0
241	LLLLFVPDSPYDPAI	0	0	0	1	0	0	0	0
242	VLRFLGFPLPIFEKG	0	28	7	78	4	6	0	0
243	ITHRLRISIPGITGR	0	29	0	39	1	11	231	9
244	NNFYRPLLRSVLVLL	2	22	9	13	0	0	0	1
245	LKQRFFLMFPKSIIW	0	25	4	38	5	8	7	0
246	SHRLWLYAAPKRPKT	0	5	4	20	5	8	0	5
247	KTRLRPAFIQQLWSS	0	13	3	7	0	5	0	0
248	HHNRRKPLEVYFKAT	0	10	3	565	12	6	0	0
249	YHSRRHPLALGFKYV	0	6	3	5	0	0	0	0
250	HKVLRPFLLRRLKKD	0	67	3	52	0	11	0	0
251	GRSRLFPHSPLGRSS	9	34	2	16	14	9	0	0
252	RRASLFPRDPKRLRP	35	148	3	25	21	79	0	2
253	SPAPIVPREPLRNEP	4029	240	241	227	1551	2786	21	46
254	VYTRTAFQIPGDDKI	3	2	0	0	0	0	1124	0

Table S7

Peptide #	Sequence	Bzz1-2	Cdc25	Cyk3	Fus1	Hof1	Hse1	Lsb1	Lsb3
255	LFMKGTPEFPKSGFS	0	0	0	0	0	2	67	8
256	PVLPPPRSPNRPTLS	9	0	0	0	9	0	39	102
257	PSRRAPLQLPQLVNK	0	0	0	0	0	0	313	0
258	NSVPIMPTLPPRPYI	0	0	2	0	2750	368	176	242
259	LYKRKPILLPDPKPL	0	0	0	0	0	2	1211	0
260	MARRRLPDRPPNGIG	160	14	0	0	130	126	587	175
261	GAGERPRLVPRPINV	0	18	3	0	11	7	18	60
262	NLDRSKISLPDFDDE	3	35	4	4	8	3	84	0
263	DAPASKPSVPPRNYF	0	0	1	0	0	0	1307	125
264	FYNRTAFQLPGDARV	0	0	0	0	0	2	2058	2
265	AIPPPVPNRPGGTTN	0	0	2	0	1	22	948	453
266	SRQAIPPPVPNRPGG	0	0	1	1	1	22	1592	658
267	NQARKPFLLPATELS	0	16	3	0	5	4	576	0
268	NRPAIRIPSLKKPAL	0	5	0	1	13	20	467	0
269	YYNRQELALPKRMSS	4	0	3	6	1	2	819	24
270	KWVRRPKLMTPLTFS	0	0	1	54	8	16	355	5
271	YSRKKLPVVLSKMTL	0	0	1	2	2	1	888	1
272	VKPRQLFPIPLNKVD	0	0	0	0	0	0	880	0
273	HPRFVSPRIPSRIVK	0	2	0	0	0	9	379	41
274	TYNRTAFQIPGDQSI	0	0	0	0	0	0	705	0
275	DGNEEKPLLPTRPNK	0	0	0	0	23	1	174	493
276	NNNKMRPHLPPLSSG	62	22	0	1	1457	24	81	1
277	NILKRRPLKLEAPSK	18	297	4	37	14	302	2	0
278	FSRRQSMDIPSKNRN	26	16	0	0	0	8	200	22
279	NMLSRPRLAKLPSIR	11	108	1	12	178	216	59	2
280	NVLRKILEIPLHSFS	5	105	1	35	0	5	0	0
281	NIKVLLNPKLGKPVK	6	96	5	2	13	20	1	0
282	VSKLRSPNTPRRLRK	4	39	1	1	0	1	1	49
283	SPAPIVPREPLRNEP	3638	142	231	99	3191	3859	0	25
284	RHLKNIPRIPIFSIL	164	113	3	1461	3668	687	0	0
285	ATNVPVAPPPPPASL	3	0	0	0	0	0	0	0
286	VPVAPPPPPASLGQS	0	0	0	0	0	0	0	0
287	RNNRPVPPPPPMRTT	233	0	2	0	0	7	0	0
288	RPVPPPPPMRTTTEG	664	0	0	1	23	9	0	0
289	SGVRLPAPPPPPRRG	20	0	1	0	633	0	0	0
290	RLPAPPPPPRRGPAP	9	0	0	0	5	1	0	0
291	RRGPAPPPPPRASRP	27	0	0	0	2	10	0	0
292	PAPPPPPRASRPTPN	0	0	1	1	0	0	0	0
293	TKHKAPPPPPPTAET	190	0	0	0	1	0	0	1
294	LYEAMPPTLPHRDWK	2	0	0	0	3	2	0	0
295	QPWTDQFEKLEKEVS	1	4	0	0	1	0	0	0
	control	6151	379	1451	616	3419	1263	507	414
	control	3603	345	1881	826	2702	1572	426	307
	control	2888	504	1656	1581	2792	1179	287	382
	control	3056	548	1732	965	4458	1617	310	513
	control	4538	989	1512	803	6895	1725	535	621

Bzz1-2

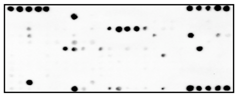

Cdc25

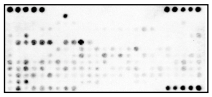

Cyk3

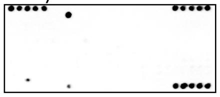

Fus1

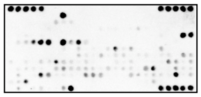

Hof1

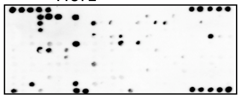

Hse1

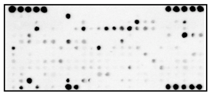

Lsb1

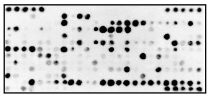

Lsb3

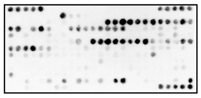

Table S7

Peptide #	Sequence	Lsb4	Nbp2	Myo3	Myo5	Pex13	Pin3	Rvs167	Sho1
	control	397	420	847	598	1341	2872	5701	1030
	control	407	505	659	674	1564	2607	2707	1199
	control	596	534	778	755	1222	3726	1769	1234
	control	445	357	469	661	992	2436	3017	2027
	control	599	223	636	570	819	2305	1552	1118
	no peptide	0	0	0	0	0	2	0	0
	no peptide	0	0	0	0	0	0	0	0
	no peptide	1	0	0	0	0	0	0	0
	no peptide	0	0	0	0	0	0	0	0
	no peptide	0	0	0	0	0	1	1	2
	no peptide	0	0	0	0	0	0	0	0
	no peptide	0	0	0	0	0	0	0	0
	no peptide	1	0	0	0	0	0	0	1
	no peptide	0	1	0	0	0	2	0	2
	no peptide	0	0	0	0	0	2	3	1
	no peptide	0	0	0	0	0	3	0	0
	no peptide	0	0	0	0	2	0	4	0
	no peptide	4	0	0	0	1	4	8	0
	no peptide	0	0	0	0	0	4	0	1
	no peptide	0	0	0	0	0	4	0	0
	control	366	297	466	396	1067	1211	2011	1464
	control	529	344	369	390	1215	2171	2673	1123
	control	468	272	426	287	1240	1180	4377	1025
	control	342	605	358	345	1441	832	2608	1012
	control	480	283	422	265	1594	1452	4287	902
1	LKPPIGRPPKFPKSP	0	0	397	117	4	0	0	24
2	AAAPKHAPPPVPNET	0	0	557	376	0	0	0	0
3	APKHAPPPVPNETDN	0	0	497	239	0	0	0	0
4	GPPPPLPPPLFPSSS	10	0	575	362	725	0	0	0
5	TTAPALPSLPPPPLL	0	0	728	651	4	0	0	0
6	PALPSLPPPPLLNVD	0	0	598	688	2	0	0	83
7	PHNPSPFFPPPDFND	0	0	319	244	70	0	0	0
8	AKDLIVPRRPEWNEG	768	1391	461	624	567	694	1953	1571
9	TEVPIPRRPPPPQAA	108	0	367	141	0	0	0	0
10	KFIPSRPAPKPPSSA	78	2508	527	339	221	8	7	47
11	IPSRPAPKPPSSASA	21	1231	575	447	873	7	1	0
12	PPPPPPPPPPPLPQS	0	0	557	489	3	0	0	0
13	AVPPPPPPPPLPESL	0	1	490	132	12	0	0	0
14	LPPPPPPPPPPPLPQ	0	0	230	325	3	0	0	0
15	PPPPPPPPPPLPQSL	0	0	275	263	28	0	0	0
16	PPLPRAPPVPPATFE	0	2	269	137	14	0	0	9
17	VNIPSPSSSPPPIPK	0	2	464	529	0	0	0	0
18	PSPSSSPPPIPKTAN	0	0	413	300	0	0	0	0
19	VPPVPLPFGIPPFPM	16	1	341	207	129	63	0	6
20	ISPPASPPPEFDFSK	0	0	233	91	0	0	0	0
21	PIVPSSPAPPLPLSG	0	0	399	434	0	0	0	0
22	QPPLPSSAPPIPTSH	0	0	334	242	0	0	0	0
23	PPIPIVPSSPAPPLP	0	0	425	318	0	0	0	0
24	HAPPLPPTAPPPPSL	0	0	465	279	0	0	0	0
25	MPAPPPPPPPPPGAF	0	0	163	49	0	1	0	0
26	VPKEPAPAPPPEPDM	0	0	149	93	4	17	0	0
27	GDHPKGPPPPPPPDE	0	0	371	227	0	2	0	0
28	DHPKGPPPPPPPDEK	0	1	431	298	0	6	0	0
29	TPRLSLPRLPNKHHW	0	0	0	0	45	7	2	35
30	SRSKPLPLTPNSKYN	2	0	0	0	21	19	1	1026
31	QLMKNLPKIPLLNDI	41	10	72	68	162	78	0	861
32	RRSKSLPTTPGIRSG	2	49	12	0	8	5	0	1351
33	KNRKNLPTIPIRLSG	103	47	5	1	13	15	86	1660
34	RRSKSLPITPKSIFN	0	0	0	0	8	4	0	1068
35	IVNKPLPPLPVAGSS	0	0	0	0	688	0	0	2244
36	NENKKLPPAPTVFGL	0	0	0	0	9	0	2	927
37	EREKALPPIPFTTTL	2	0	0	0	12	2	2	1484
38	DLFKPLPEPPTELGR	0	0	0	0	47	0	0	1318
39	AATTSTPPLPRRRAT	1355	0	31	0	364	35	2276	38
40	PLQSKIPMLPSRRTM	960	12	102	35	329	280	1569	21
41	SSSSTPPTLPPRRIE	907	0	99	11	365	58	5648	0
42	TTNRGPPPLPPRANV	743	0	129	206	470	48	4619	58
43	LKRITSPPLPPRADS	700	0	75	4	145	11	4037	65
44	ISNFVPPNLPMRRFK	452	1	59	37	671	217	2143	65

Peptide #	Sequence	Lsb4	Nbp2	Myo3	Myo5	Pex13	Pin3	Rvs167	Sho1
45	EEEEEHPPLPARRKS	590	1	0	0	155	0	222	0
46	DDEDVPPQLPTRTKS	897	0	0	0	97	2	1257	0
47	QQNRPLPQLPNRNNR	484	0	0	0	589	34	1312	1596
48	NPLPKEPRLPKRKVA	288	0	7	0	16	10	200	107
49	VATSTSPKLPPRGKQ	275	0	0	0	87	15	4095	1
50	VQPTAAPATPPRHIS	251	0	0	0	0	0	12	0
51	GATNNAPTLPKRKNP	570	0	0	0	27	0	2546	2
52	SSSSSPPPLPTRRDH	748	0	69	19	467	0	3538	0
53	KKAPPPVVKPKPRNF	0	0	0	0	4	1	1	21
54	QNTPPLPPKPKSPHL	231	1	0	0	309	0	8	72
55	ATKSASPTLPTRRSR	1182	2	12	0	249	20	1537	132
56	SKIRPTPRKPSRMAT	162	44	50	23	40	20	148	177
57	TSFKGRPKPPKTLKH	0	0	0	0	3	4	0	43
58	ALKQKKIPPPFKPHL	0	0	0	0	0	0	0	0
59	DKSRPPRPPPKPLHL	177	0	160	44	144	0	0	155
60	RPPRPPPKPLHLRTE	201	86	121	33	479	5	12	780
61	KDKSRPPRPPPKPLH	114	2	104	10	8	0	0	202
62	ERPKRRAPPPVPKKP	89	58	358	180	62	11	10	311
63	KRRAPPPVPKKPSSR	150	751	454	144	47	14	165	370
64	PKRRAPPPVPKKPSS	182	650	342	119	41	11	44	399
65	RPKRRAPPPVPKKPS	129	887	491	232	42	9	99	487
66	RAPPPVPKKPSSRIA	180	1049	233	15	395	37	45	509
67	LKHGWKPLRPIKLIS	4	2	0	0	17	3	0	55
68	NMLKKKPLKKPLKRF	1	66	24	1	18	34	0	228
69	FPPKRKPLLRPQRSD	81	9	30	0	16	19	25	154
70	DKKTKPTPPPKPSHL	0	0	0	0	4	0	0	115
71	TKPTPPPKPSHLKPK	5	1	0	0	266	0	0	199
72	KDKKTKPTPPPKPSH	0	2	0	0	0	0	1	50
73	GSKSGPPPRPKKPST	2	0	3	0	23	2	1	16
74	STKKPRPPVKSKPKH	0	0	0	0	0	0	12	12
75	VQKRKLPLPGSIASA	13	1	0	0	0	0	0	11
76	FPGKKSPRLPHRNKK	225	0	0	0	16	0	314	84
77	KKEKKPPKIPKKVYT	0	0	0	0	0	1	0	16
78	NLKKRPPTAPQRKIS	141	48	20	3	13	7	18	229
79	AAHKTKPSIPPKKVE	0	0	0	0	2	0	0	4
80	KTKQQKPAIPQKKSF	2	0	2	1	5	3	4	38
81	MTKTPETSPPKRPMG	36	0	455	499	8	24	0	5
82	QTREPNEPPPPSPAM	13	0	502	515	7	21	4	0
83	GKFIPSRPAPKPPSS	56	2885	553	593	362	12	5	22
84	DTFLPPPPPPPSNFE	0	0	518	453	8	0	0	0
85	VDFTLPPPPPPPGLD	0	0	491	367	2	0	0	0
86	DFTLPPPPPPPGLDE	0	3	345	306	0	0	0	0
87	FTLPPPPPPPGLDEL	0	0	455	234	13	0	0	0
88	DLPLPDLPPEPPTEL	0	0	262	101	0	0	0	0
89	VPPPMQPPPIESSNL	48	6	318	167	27	114	0	0
90	MFLISPPASPPPEFD	0	0	326	219	12	65	0	0
91	APPLPPTAPPPPSLP	0	0	537	559	4	0	0	0
92	NPTKSPPPPPSPSTM	4	0	436	223	43	109	0	2
93	PPIPNAPLSPAPAVP	0	0	483	443	1	0	0	0
94	MSPLGAPPPPPHKDH	0	0	245	108	0	0	0	0
95	SPLGAPPPPPHKDHL	0	0	327	81	0	0	2	1
96	RTRRRPPPPPIPSTQ	69	107	487	243	21	10	99	83
97	GDHPKGPPPPPPPPD	0	1	456	262	0	0	0	0
98	TPEIPPPLPPKIMVH	42	0	304	87	321	20	0	52
99	RHLKLVPSTPMDIPW	6	3	81	10	99	329	43	72
100	IFLRISINFPLNYPN	14	74	71	15	76	101	1	134
101	PLRRTVMSLPGNHDI	0	18	23	12	31	72	0	12
102	NNQRRKPLLLNIGDH	0	42	0	0	0	0	0	79
103	NPNRRAPRRPLSTQH	18	626	46	0	27	4	0	267
104	NFLRLLIGLPSQNEL	0	84	10	0	47	15	0	93
105	NHSRSLFSLPGNQQS	0	77	13	1	36	16	0	141
106	ALFRLSFFFPLDPAY	21	283	44	17	93	39	8	168
107	QFDLKMPSKPIHNLK	27	0	34	29	95	68	6	7
108	RHLKNIPRIPIFSIL	4	184	14	5	163	84	289	160
109	FLIKFTPKFPQSIDH	63	418	77	27	73	41	2	304
110	VGFRLALKFPSRITG	207	1105	164	69	127	150	6	718
111	FVLRYYILIPHEQWD	0	47	0	0	10	9	0	5
112	APERAVPILPPRNNV	1217	0	66	0	536	13	1338	7
113	VQQDSLPKLPFRSWG	1313	0	0	0	490	7	944	66
114	LPMNIQPDLPPRKIM	1362	6	102	35	35	175	2811	29

Table S7

Peptide #	Sequence	Lsb4	Nbp2	Myo3	Myo5	Pex13	Pin3	Rvs167	Sho1
115	QQEQNTPPLPPKPKS	776	0	0	0	79	0	0	14
116	SSAAPPPPPRRATPE	965	0	99	18	58	5	1794	3
117	APPPPPRRATPEKKP	424	0	92	14	68	8	525	21
118	EQEEIAPSLPSRNSI	517	0	0	0	417	0	31	0
119	EAEEAAPQLPSRSSA	529	0	0	0	6	2	75	0
120	LASNSPPVYPKRIRH	107	0	0	0	3	0	0	7
121	QGYPRQPQRPQRYHP	16	0	0	0	7	2	0	0
122	SAPDIPPRSPNRNAH	145	0	0	0	21	4	816	0
123	QRIRKIPGIPLMSVG	24	5	28	7	90	71	156	42
124	SSTPTTPERPKRKSG	363	0	0	0	8	6	0	0
125	AERNYAPRLPRRETS	137	0	0	0	76	18	306	32
126	EVTPKVPERPSRRKT	317	0	0	0	171	25	129	23
127	TPTSGPPLLPPRNTM	356	0	97	45	813	66	2945	21
128	ANNQGPPNLPARDKS	642	0	0	0	229	0	3317	0
129	VAPPPLPNRQLPNLD	813	0	112	59	563	5	5724	0
130	MAMRPIPPLPTESEY	278	0	7	27	235	4	1462	30
131	YLTRPLPSTPNEDSR	598	0	0	0	297	0	15	1324
132	NTMKKRPAPPSLPSL	63	120	99	115	80	57	9	95
133	RKRAPTPPAPSRSEK	29	283	20	0	6	7	9	99
134	FYHRPAPKPPVTKKV	3	314	37	12	143	19	0	8
135	DRIRMFIRFPKFLLE	7	52	18	2	38	30	0	71
136	KMSRSPPRPPSLKII	88	513	81	64	124	69	67	55
137	NLPKRKPPKLQGPFS	20	56	42	3	11	6	0	121
138	LKVTPGRRAPTISKI	7	85	80	6	3	14	0	72
139	RKNRAPPQRPRFNRD	75	490	61	3	10	9	2	262
140	IHTRRPSSTSQYLIR	2	0	1	1	11	25	0	28
141	RKNRAPPQRPRFNRE	49	240	51	5	14	12	4	178
142	RLSRLLLSLPPESFT	3	42	10	0	19	27	0	66
143	APKREAPKPPANTSP	1	139	277	126	7	2	0	0
144	PELAPKREAPKPPAN	2	281	497	262	0	0	0	0
145	QLLNPNRRAPRRPLS	35	1128	168	13	21	30	0	50
146	LEFRAILFIPKRAPF	18	53	34	9	59	68	0	103
147	VRTRRRPPPPIPST	39	35	296	188	8	11	45	46
148	QPQRTAPKPPISAPR	1	276	294	230	44	7	0	0
149	STPQTMRQAPKRPDA	33	53	47	47	116	192	0	4
150	AHFQPQRTAPKPPIS	4	750	413	104	13	5	0	1
151	RPVPRRPSQPLNTLS	163	71	5	0	348	26	4065	95
152	IAPIPMPKPNPTHIS	9	1	22	7	54	43	0	0
153	IRMPSNPALPKKLLN	56	15	72	81	352	115	0	79
154	VFQFQMPTTPISTKM	39	3	32	74	219	125	0	46
155	QTMRQAPKRPDADVA	115	989	44	103	73	61	10	21
156	NSMRPPLLIPAATTK	87	9	40	65	47	40	0	39
157	PSTMDFPKLPSFQNS	67	57	51	77	35	54	2	25
158	PKMLPIPSKPTLFDL	42	19	36	52	52	69	0	164
159	TSIEIPKRSPLRFTS	8	0	4	2	19	10	0	8
160	GQYRRTIVIPRRFFT	28	120	17	2	20	18	0	124
161	RSKRLFPVRPMATAH	15	46	2	2	11	12	9	90
162	MKRSRPSRSIPYTTP	6	0	5	2	10	34	6	54
163	KKIRLIFTYPSTVGR	28	258	41	12	12	31	0	253
164	SKAKRPKFLLDLQIK	5	0	6	0	5	4	0	114
165	TKFRISLGLPVGAIM	46	159	69	14	29	65	0	367
166	RVTRKRPREPKSTND	10	40	19	3	0	3	0	173
167	WTQRRGPLVVYAEDN	1	0	0	0	10	32	0	1
168	FIRKRAPTPPAPSRS	26	219	35	4	27	26	4	90
169	KIRRSSLMIPNPQQF	3	0	35	11	30	142	0	47
170	IKTRSVPKIPSIESV	166	12	5	0	32	15	1343	4
171	HVRRSILALPLGVLL	4	0	3	1	3	2	2	35
172	RTIRKLPLMSLSEYF	2	1	3	1	12	27	0	6
173	NRARYLPQNPDIIAG	0	1	0	0	0	2	0	10
174	NFKRLLPRDPSEKSS	0	12	6	0	7	7	0	51
175	LRWRHKFFLPAIAAI	0	0	0	0	3	4	0	9
176	YVRRRPRVRLEPLLS	3	16	5	1	2	2	0	102
177	KNPRRQLQIPRQQPS	8	0	4	1	3	3	2	99
178	SRYRRPLTLLLLKPF	2	28	8	0	16	2	7	78
179	LLGRKRPVMERVVDI	27	0	10	6	110	61	5	74
180	FFRRSELYLPNSSKA	0	14	0	0	10	1	5	62
181	LLRRTIPKRPFYHVL	2	100	0	0	12	0	31	26
182	NFFRKKLGIPRKLKR	1	72	3	0	14	7	3	107
183	RRLRDKYKLPIVDAD	0	7	0	0	0	0	1	19
184	TKRKRPARLIFYDSK	7	183	7	3	1	0	0	91

Table S7

Peptide #	Sequence	Lsb4	Nbp2	Myo3	Myo5	Pex13	Pin3	Rvs167	Sho1
185	WRPRIVPILPYITRL	6	1	0	0	10	8	36	3
186	KRRTSLFPWLHKPGI	0	57	0	0	4	0	0	61
187	KLSRGRYGLPLSSRP	0	2	1	0	3	7	3	31
188	VSKLRSPNTPRRLRK	206	0	8	2	3	5	4	267
189	GKLKHIPRRPYEIER	0	0	0	0	2	4	1	9
190	RKQRSIPLSPIVPES	9	0	4	2	2	7	0	49
191	TKIRRRPQQPLTDFT	8	2	14	2	3	11	1	120
192	RSRRSSFAYPQQVAI	0	0	8	0	4	8	2	64
193	PSRRDSLSIPRAVDA	0	0	3	2	1	11	0	13
194	LSKRLPLRFPEHTSS	4	2	0	0	3	0	0	29
195	YRRKSIPFAPHQITA	2	0	0	0	10	9	0	17
196	RVRKTHVPASKRPSG	1	0	1	0	0	1	0	124
197	KRNRLKILLPFLEQS	0	22	10	0	11	17	0	81
198	NGRRRPKFRVQLSGN	9	63	30	2	5	11	0	155
199	RRRDFGAPANKRPRR	4	2	16	2	2	9	0	90
200	RKRRTHLRLPLIRSN	0	9	5	0	0	3	0	47
201	RRARRQMGIPTISQV	16	2	10	20	19	33	2	79
202	RRRIAYPFYPFKKLG	0	21	2	0	5	0	0	25
203	LRIKRKPVQTFFEFI	12	46	8	0	13	5	2	173
204	ISRRRLPVIMHRLKM	21	5	7	2	22	27	21	112
205	VRVKKRPLIRPLNSS	16	9	8	1	4	1	7	188
206	VNFRRIPTGPDSPPT	2	0	0	0	6	0	2	19
207	KRKRFNLLLPKPYLT	1	53	0	0	3	0	3	98
208	HRRRRVFSIPSLKSI	10	176	6	0	4	7	3	123
209	RRRRRPHRIERPLSN	6	74	7	1	2	1	4	270
210	RSLRKRPTSPSISGS	5	198	9	2	5	8	2	295
211	QHQQRQPKRPKRYSL	101	21	2	2	6	2	0	115
212	ALRRRLPVTRSKINW	7	149	10	0	4	10	8	196
213	GRLRPKRIAPWHLIQ	0	10	0	0	8	5	8	83
214	IKKRNKIRLPSGSPE	6	24	7	0	2	4	4	122
215	GLRRARYKFPGQQKI	2	22	4	0	2	4	6	69
216	SLSRRRFSLPSMPNV	9	34	27	4	28	66	4	84
217	AGKRRLPLVRFKASD	18	125	22	2	4	8	6	260
218	RKLRTVPGVPLIHLT	5	0	8	0	4	3	43	6
219	KRKRRLPVSEDTNTK	4	8	9	0	0	4	1	54
220	NRLRKRLNLPSEISI	0	32	4	2	2	17	0	58
221	QRKSLRRPTLSKPAV	3	5	10	3	3	9	0	116
222	TIRRRAPLSLLESHT	1	11	0	0	3	11	3	52
223	RMVRRRPLRVQFSAR	25	104	50	10	17	70	3	259
224	RSVKRPRRAPRPVVS	34	67	11	1	0	2	9	60
225	RRTRLRPPTPLSQLL	1	11	6	0	4	9	0	39
226	SNRRPVPRRPSQPLN	333	46	3	0	129	12	3398	31
227	RFKRHRLEFPFNESE	0	14	0	0	13	15	0	41
228	SKSRLLIELPEGFFT	0	0	0	0	14	4	0	10
229	GKKVRPLLVLLLSRA	2	10	7	1	19	0	0	51
230	FHLNPKRSAVLYDRP	0	0	0	1	35	32	7	35
231	LLIRWLILMPLVGSR	25	215	34	10	78	47	1	150
232	ILARAILTIPRVLDK	38	17	17	3	56	107	0	137
233	VTHRLRISIPGITGR	22	72	13	11	20	46	0	180
234	GTIARPLFLVVLFFI	0	0	1	0	11	3	0	0
235	NAGIRPKFILALNDE	0	2	0	0	10	3	4	31
236	HHKRRPTTIDVPGLT	5	1	7	4	18	28	0	67
237	RLSFFFPLDPAYIRN	0	21	4	0	21	13	0	68
238	LTRRFPFLFPFDTRM	4	16	4	0	16	18	0	57
239	HGKRRAPLLLAKLDV	4	1	5	0	5	9	0	85
240	GERSRPLVISILSSA	4	2	5	0	39	26	0	38
241	LLLLFVPDSPYDPAI	0	1	0	0	0	0	0	0
242	VLRFLGFPLPIFEKG	0	41	1	0	4	3	0	43
243	ITHRLRISIPGITGR	5	32	37	4	32	69	0	191
244	NNFYRPLLRSVLVLL	0	3	0	0	4	5	2	17
245	LKQRFFLMFPKSIIW	0	9	12	0	21	54	0	53
246	SHRLWLYAAPKRPKT	0	1	6	0	8	13	0	39
247	KTRLRPAFIQQLWSS	0	4	0	0	7	15	1	22
248	HHNRRKPLEVYFKAT	1	0	3	1	16	20	2	43
249	YHSRRHPLALGFKYV	0	0	1	0	4	13	0	7
250	HKVLRPFLLRRLKKD	0	13	0	0	0	4	0	26
251	GRSRLFPHSPLGRSS	0	4	0	0	4	1	0	21
252	RRASLFPRDPKRLRP	4	0	7	1	2	11	0	67
253	SPAPIVPREPLRNEP	114	2	122	88	403	721	2592	138
254	VYTRTAFQIPGDDKI	0	0	0	0	0	29	0	0

Table S7

Peptide #	Sequence	Lsb4	Nbp2	Myo3	Myo5	Pex13	Pin3	Rvs167	Sho1
255	LFMKGTPEFPKSGFS	50	5	54	48	211	40	1	17
256	PVLPPPRSPNRPTLS	325	0	22	6	86	7	6	0
257	PSRRAPLQLPQLVNK	2	0	0	0	1	5	0	17
258	NSVPIMPTLPPRPYI	513	0	26	25	172	28	157	0
259	LYKRKPILLPDPKPL	0	1	0	1	1	7	0	15
260	MARRRLPDRPPNGIG	533	3	15	5	99	91	1594	393
261	GAGERPRLVPRPINV	105	2	0	1	24	5	4	4
262	NLDRSKISLPDFDDE	5	6	1	0	3	8	6	16
263	DAPASKPSVPPRNYF	231	2	0	0	91	23	58	0
264	FYNRTAFQLPGDARV	0	2	1	1	6	151	0	4
265	AIPPPVPNRPGGTTN	222	1	8	0	375	48	1432	0
266	SRQAIPPPVPNRPGG	373	1	63	21	133	55	833	14
267	NQARKPFLLPATELS	0	4	1	1	0	4	0	38
268	NRPAIRIPSLKKPAL	6	2	3	2	5	9	0	39
269	YYNRQELALPKRMSS	45	1	11	5	16	327	21	9
270	KWVRRPKLMTPLTFS	2	3	6	6	6	53	0	19
271	YSRKKLPVVLSKMTL	0	0	1	4	8	20	2	47
272	VKPRQLFPIPLNKVD	0	0	0	0	0	5	0	8
273	HPRFVSPRIPSRIVK	57	2	3	0	21	86	4	40
274	TYNRTAFQIPGDQSI	0	0	0	1	0	45	0	0
275	DGNEEKPLLPTRPNK	281	0	0	0	81	0	263	0
276	NNNKMRPHLPPLSSG	22	12	18	7	84	85	0	61
277	NILKRRPLKLEAPSK	1	0	4	0	2	1	0	65
278	FSRRQSMDIPSKNRN	84	2	44	83	61	195	0	60
279	NMLSRPRLAKLPSIR	10	0	9	11	21	40	0	38
280	NVLRKILEIPLHSFS	0	0	0	0	0	0	0	6
281	NIKVLLNPKLGKPVK	1	2	6	0	6	2	0	63
282	VSKLRSPNTPRRLRK	92	0	6	0	1	3	1	126
283	SPAPIVPREPLRNEP	89	8	104	90	875	289	3297	78
284	RHLKNIPRIPIFSIL	7	63	19	3	113	84	67	101
285	ATNVPVAPPPPPASL	0	0	0	0	0	0	0	0
286	VPVAPPPPPASLGQS	0	0	0	1	0	0	0	0
287	RNNRPVPPPPPMRTT	0	0	0	1	201	31	1869	0
288	RPVPPPPPMRTTTEG	0	0	0	2	185	59	7509	2
289	SGVRLPAPPPPPRRG	0	0	0	0	55	4	3627	0
290	RLPAPPPPPRRGPAP	0	1	0	0	75	8	1499	0
291	RRGPAPPPPPRASRP	1	0	0	0	17	17	3336	0
292	PAPPPPPRASRPTPN	0	0	0	1	56	6	514	0
293	TKHKAPPPPPPTAET	0	0	0	1	0	0	0	0
294	LYEAMPPTLPHRDWK	1	0	0	1	59	19	151	0
295	QPWTDQFEKLEKEVS	0	0	0	0	0	0	0	0
	control	216	349	578	520	1424	1476	1741	1393
	control	235	346	520	471	1404	2764	2363	1382
	control	177	449	289	294	920	1801	3274	1148
	control	194	565	468	458	1361	2210	5957	836
	control	270	408	404	551	1610	1452	6233	1226

Lsb4

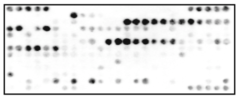

Nbp2

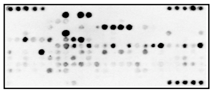

Myo3

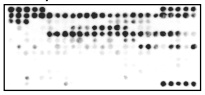

Myo5

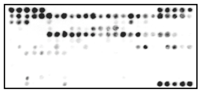

Pex13

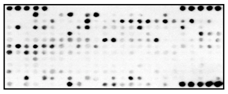

Pin3

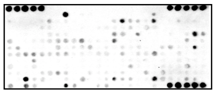

Rvs167

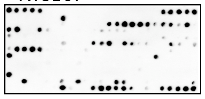

Sho1

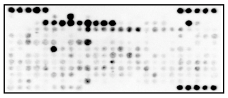

Table S7

Peptide #	Sequence	Sla1-2	Sla1-3	GST
	control	791	10174	1158
	control	727	9647	1556
	control	767	5978	956
	control	708	6607	1035
	control	842	3872	945
	no peptide	3	0	0
	no peptide	0	0	0
	no peptide	0	0	0
	no peptide	0	0	0
	no peptide	0	0	0
	no peptide	1	0	0
	no peptide	1	1	0
	no peptide	2	0	0
	no peptide	0	3	0
	no peptide	1	2	0
	no peptide	2	0	0
	no peptide	0	0	0
	no peptide	1	0	0
	no peptide	0	2	0
	no peptide	0	0	0
	control	637	5811	790
	control	538	4054	754
	control	720	6609	738
	control	811	4604	776
	control	789	4117	759
1	LKPPIGRPPKFPKSP	0	0	0
2	AAAPKHAPPPVPNET	0	0	0
3	APKHAPPPVPNETDN	0	0	0
4	GPPPPLPPPLFPSSS	0	0	0
5	TTAPALPSLPPPPLL	0	0	0
6	PALPSLPPPPLLNVD	0	0	0
7	PHNPSPFFPPPDFND	0	0	0
8	AKDLIVPRRPEWNEG	550	2734	446
9	TEVPIPRRPPPPQAA	0	0	3
10	KFIPSRPAPKPPSSA	0	199	0
11	IPSRPAPKPPSSASA	0	29	0
12	PPPPPPPPPPPLPQS	0	0	0
13	AVPPPPPPPPLPESL	0	3	1
14	LPPPPPPPPPPPLPQ	0	4	0
15	PPPPPPPPPPLPQSL	0	6	1
16	PPLPRAPPVPPATFE	1	1	0
17	VNIPSPSSSPPPIPK	0	3	0
18	PSPSSSPPPIPKTAN	0	0	1
19	VPPVPLPFGIPPFPM	1	0	0
20	ISPPASPPPEFDFSK	0	0	0
21	PIVPSSPAPPLPLSG	0	0	0
22	QPPLPSSAPPIPTSH	0	0	0
23	PPIPIVPSSPAPPLP	0	0	0
24	HAPPLPPTAPPPPSL	0	0	0
25	MPAPPPPPPPPPGAF	0	2	0
26	VPKEPAPAPPPEPDM	0	0	0
27	GDHPKGPPPPPPPDE	0	2	0
28	DHPKGPPPPPPPDEK	0	12	0
29	TPRLSLPRLPNKHHW	0	0	0
30	SRSKPLPLTPNSKYN	3	7	0
31	QLMKNLPKIPLLNDI	5	2	1
32	RRSKSLPTTPGIRSG	0	26	0
33	KNRKNLPTIPIRLSG	0	55	0
34	RRSKSLPITPKSIFN	1	21	2
35	IVNKPLPPLPVAGSS	0	0	0
36	NENKKLPPAPTVFGL	0	1	0
37	EREKALPPIPFTTTL	1	0	0
38	DLFKPLPEPPTELGR	0	0	0
39	AATTSTPPLPRRRAT	0	0	1
40	PLQSKIPMLPSRRTM	14	0	0
41	SSSSTPPTLPPRRIE	1	1	0
42	TTNRGPPPLPPRANV	0	1	0
43	LKRITSPPLPPRADS	0	0	0
44	ISNFVPPNLPMRRFK	39	3	1

Table S7

Peptide #	Sequence	Sla1-2	Sla1-3	GST
45	EEEEEHPPLPARRKS	0	1	0
46	DDEDVPPQLPTRTKS	0	1	1
47	QQNRPLPQLPNRNNR	0	10	0
48	NPLPKEPRLPKRKVA	2	45	2
49	VATSTSPKLPPRGKQ	0	13	0
50	VQPTAAPATPPRHIS	0	5	0
51	GATNNAPTLPKRKNP	92	1	0
52	SSSSSPPPLPTRRDH	0	7	1
53	KKAPPPVVKPKPRNF	2	0	3
54	QNTPPLPPKPKSPHL	1	0	2
55	ATKSASPTLPTRRSR	8	1	1
56	SKIRPTPRKPSRMAT	21	21	2
57	TSFKGRPKPPKTLKH	0	2	0
58	ALKQKKIPPPFKPHL	0	0	0
59	DKSRPPRPPPKPLHL	0	0	0
60	RPPRPPPKPLHLRTE	1	43	0
61	KDKSRPPRPPPKPLH	0	1	0
62	ERPKRRAPPPVPKKP	0	2607	0
63	KRRAPPPVPKKPSSR	0	4638	3
64	PKRRAPPPVPKKPSS	0	3503	2
65	RPKRRAPPPVPKKPS	2	2977	6
66	RAPPPVPKKPSSRIA	0	155	1
67	LKHGWKPLRPIKLIS	0	16	0
68	NMLKKKPLKKPLKRF	2	36	6
69	FPPKRKPLLRPQRSD	1	25	2
70	DKKTKPTPPPKPSHL	1	0	0
71	TKPTPPPKPSHLKPK	0	1	0
72	KDKKTKPTPPPKPSH	1	0	3
73	GSKSGPPPRPKKPST	1	4	2
74	STKKPRPPVKSKPKH	0	5	2
75	VQKRKLPLPGSIASA	0	4	0
76	FPGKKSPRLPHRNKK	0	12	1
77	KKEKKPPKIPKKVYT	0	12	3
78	NLKKRPPTAPQRKIS	3	161	2
79	AAHKTKPSIPPKKVE	0	0	0
80	KTKQQKPAIPQKKSF	0	0	1
81	MTKTPETSPPKRPMG	2	0	0
82	QTREPNEPPPPSPAM	0	0	0
83	GKFIPSRPAPKPPSS	0	492	0
84	DTFLPPPPPPPSNFE	0	0	0
85	VDFTLPPPPPPPGLD	1	0	0
86	DFTLPPPPPPPGLDE	0	0	1
87	FTLPPPPPPPGLDEL	0	0	1
88	DLPLPDLPPEPPTEL	0	0	1
89	VPPPMQPPPIESSNL	2	0	0
90	MFLISPPASPPPEFD	0	0	0
91	APPLPPTAPPPPSLP	0	3	1
92	NPTKSPPPPPSPSTM	0	1	1
93	PPIPNAPLSPAPAVP	0	0	0
94	MSPLGAPPPPPHKDH	0	0	1
95	SPLGAPPPPPHKDHL	0	0	1
96	RTRRRPPPPPIPSTQ	3	1976	1
97	GDHPKGPPPPPPPPD	0	0	0
98	TPEIPPPLPPKIMVH	0	0	0
99	RHLKLVPSTPMDIPW	3	290	0
100	IFLRISINFPLNYPN	12	199	0
101	PLRRTVMSLPGNHDI	3	0	0
102	NNQRRKPLLLNIGDH	0	138	0
103	NPNRRAPRRPLSTQH	4	607	0
104	NFLRLLIGLPSQNEL	0	0	0
105	NHSRSLFSLPGNQQS	1	124	2
106	ALFRLSFFFPLDPAY	4	54	1
107	QFDLKMPSKPIHNLK	2	0	0
108	RHLKNIPRIPIFSIL	0	992	0
109	FLIKFTPKFPQSIDH	10	13	0
110	VGFRLALKFPSRITG	52	124	0
111	FVLRYYILIPHEQWD	3	3	0
112	APERAVPILPPRNNV	0	0	0
113	VQQDSLPKLPFRSWG	0	0	0
114	LPMNIQPDLPPRKIM	6	0	0

Table S7

Peptide #	Sequence	Sla1-2	Sla1-3	GST
115	QQEQNTPPLPPKPKS	1	3	0
116	SSAAPPPPPRRATPE	0	0	1
117	APPPPPRRATPEKKP	0	0	1
118	EQEEIAPSLPSRNSI	0	0	2
119	EAEEAAPQLPSRSSA	0	2	0
120	LASNSPPVYPKRIRH	0	0	0
121	QGYPRQPQRPQRYHP	0	0	1
122	SAPDIPPRSPNRNAH	423	3	0
123	QRIRKIPGIPLMSVG	4	8	0
124	SSTPTTPERPKRKSG	0	0	2
125	AERNYAPRLPRRETS	0	5	0
126	EVTPKVPERPSRRKT	0	15	0
127	TPTSGPPLLPPRNTM	1	0	0
128	ANNQGPPNLPARDKS	1	0	1
129	VAPPPLPNRQLPNLD	0	0	0
130	MAMRPIPPLPTESEY	0	0	0
131	YLTRPLPSTPNEDSR	0	1	0
132	NTMKKRPAPPSLPSL	1	11	0
133	RKRAPTPPAPSRSEK	0	24	1
134	FYHRPAPKPPVTKKV	0	2038	0
135	DRIRMFIRFPKFLLE	0	24	0
136	KMSRSPPRPPSLKII	8	88	1
137	NLPKRKPPKLQGPFS	1	213	1
138	LKVTPGRRAPTISKI	0	6	0
139	RKNRAPPQRPRFNRD	3	353	4
140	IHTRRPSSTSQYLIR	0	21	0
141	RKNRAPPQRPRFNRE	4	379	4
142	RLSRLLLSLPPESFT	0	45	0
143	APKREAPKPPANTSP	0	12	0
144	PELAPKREAPKPPAN	0	0	0
145	QLLNPNRRAPRRPLS	3	9	2
146	LEFRAILFIPKRAPF	6	8	0
147	VRTRRRPPPPPIPST	4	1790	2
148	QPQRTAPKPPISAPR	0	9	0
149	STPQTMRQAPKRPDA	2	2	1
150	AHFQPQRTAPKPPIS	0	4	0
151	RPVPRRPSQPLNTLS	0	31	0
152	IAPIPMPKPNPTHIS	1	0	0
153	IRMPSNPALPKKLLN	18	0	0
154	VFQFQMPTTPISTKM	7	0	0
155	QTMRQAPKRPDADVA	19	42	1
156	NSMRPPLLIPAATTK	6	0	1
157	PSTMDFPKLPSFQNS	7	0	0
158	PKMLPIPSKPTLFDL	3	0	1
159	TSIEIPKRSPLRFTS	792	0	0
160	GQYRRTIVIPRRFFT	8	58	5
161	RSKRLFPVRPMATAH	0	36	0
162	MKRSRPSRSIPYTTP	6	35	0
163	KKIRLIFTYPSTVGR	6	8	0
164	SKAKRPKFLLDLQIK	2	19	2
165	TKFRISLGLPVGAIM	9	3	0
166	RVTRKRPREPKSTND	2	47	2
167	WTQRRGPLVVYAEDN	0	18	0
168	FIRKRAPTPPAPSRS	0	155	0
169	KIRRSSLMIPNPQQF	4	9	3
170	IKTRSVPKIPSIESV	1	6	0
171	HVRRSILALPLGVLL	0	0	0
172	RTIRKLPLMSLSEYF	1	19	0
173	NRARYLPQNPDIIAG	0	11	0
174	NFKRLLPRDPSEKSS	0	156	0
175	LRWRHKFFLPAIAAI	0	24	0
176	YVRRRPRVRLEPLLS	3	44	9
177	KNPRRQLQIPRQQPS	0	3	4
178	SRYRRPLTLLLLKPF	1	15	0
179	LLGRKRPVMERVVDI	5	4	0
180	FFRRSELYLPNSSKA	0	53	0
181	LLRRTIPKRPFYHVL	0	245	1
182	NFFRKKLGIPRKLKR	0	58	0
183	RRLRDKYKLPIVDAD	0	34	0
184	TKRKRPARLIFYDSK	2	33	1

Table S7

Peptide #	Sequence	Sla1-2	Sla1-3	GST
185	WRPRIVPILPYITRL	0	9	0
186	KRRTSLFPWLHKPGI	0	20	0
187	KLSRGRYGLPLSSRP	1	31	0
188	VSKLRSPNTPRRLRK	0	9	3
189	GKLKHIPRRPYEIER	0	16	1
190	RKQRSIPLSPIVPES	0	17	1
191	TKIRRRPQQPLTDFT	1	20	2
192	RSRRSSFAYPQQVAI	1	29	0
193	PSRRDSLSIPRAVDA	0	0	2
194	LSKRLPLRFPEHTSS	0	24	0
195	YRRKSIPFAPHQITA	0	28	1
196	RVRKTHVPASKRPSG	1	6	2
197	KRNRLKILLPFLEQS	0	49	5
198	NGRRRPKFRVQLSGN	5	221	3
199	RRRDFGAPANKRPRR	0	30	3
200	RKRRTHLRLPLIRSN	0	14	4
201	RRARRQMGIPTISQV	15	14	7
202	RRRIAYPFYPFKKLG	0	21	2
203	LRIKRKPVQTFFEFI	0	9	1
204	ISRRRLPVIMHRLKM	1	20	0
205	VRVKKRPLIRPLNSS	2	22	1
206	VNFRRIPTGPDSPPT	0	17	0
207	KRKRFNLLLPKPYLT	2	34	2
208	HRRRRVFSIPSLKSI	0	117	1
209	RRRRRPHRIERPLSN	0	25	1
210	RSLRKRPTSPSISGS	1	44	3
211	QHQQRQPKRPKRYSL	5	78	8
212	ALRRRLPVTRSKINW	0	73	2
213	GRLRPKRIAPWHLIQ	0	41	0
214	IKKRNKIRLPSGSPE	0	43	3
215	GLRRARYKFPGQQKI	1	50	3
216	SLSRRRFSLPSMPNV	2	388	0
217	AGKRRLPLVRFKASD	3	27	4
218	RKLRTVPGVPLIHLT	1	5	1
219	KRKRRLPVSEDTNTK	0	21	6
220	NRLRKRLNLPSEISI	1	141	1
221	QRKSLRRPTLSKPAV	3	22	6
222	TIRRRAPLSLLESHT	1	38	1
223	RMVRRRPLRVQFSAR	37	128	9
224	RSVKRPRRAPRPVVS	6	25	4
225	RRTRLRPPTPLSQLL	0	35	5
226	SNRRPVPRRPSQPLN	0	459	0
227	RFKRHRLEFPFNESE	0	97	0
228	SKSRLLIELPEGFFT	0	0	0
229	GKKVRPLLVLLLSRA	0	0	0
230	FHLNPKRSAVLYDRP	0	140	5
231	LLIRWLILMPLVGSR	3	7	2
232	ILARAILTIPRVLDK	6	4	0
233	VTHRLRISIPGITGR	4	0	1
234	GTIARPLFLVVLFFI	0	0	1
235	NAGIRPKFILALNDE	1	2	0
236	HHKRRPTTIDVPGLT	2	4	0
237	RLSFFFPLDPAYIRN	0	113	1
238	LTRRFPFLFPFDTRM	1	129	0
239	HGKRRAPLLLAKLDV	0	1	1
240	GERSRPLVISILSSA	1	0	0
241	LLLLFVPDSPYDPAI	0	2	1
242	VLRFLGFPLPIFEKG	0	0	0
243	ITHRLRISIPGITGR	4	2	0
244	NNFYRPLLRSVLVLL	0	4	1
245	LKQRFFLMFPKSIIW	0	17	0
246	SHRLWLYAAPKRPKT	3	16	0
247	KTRLRPAFIQQLWSS	0	33	0
248	HHNRRKPLEVYFKAT	0	14	1
249	YHSRRHPLALGFKYV	0	9	0
250	HKVLRPFLLRRLKKD	0	30	0
251	GRSRLFPHSPLGRSS	1	8	0
252	RRASLFPRDPKRLRP	7	158	5
253	SPAPIVPREPLRNEP	269	3538	1003
254	VYTRTAFQIPGDDKI	1	0	3

Table S7

Peptide #	Sequence	Sla1-2	Sla1-3	GST
255	LFMKGTPEFPKSGFS	5	0	0
256	PVLPPPRSPNRPTLS	442	6	1
257	PSRRAPLQLPQLVNK	0	0	1
258	NSVPIMPTLPPRPYI	0	0	1
259	LYKRKPILLPDPKPL	0	0	2
260	MARRRLPDRPPNGIG	1	36	0
261	GAGERPRLVPRPINV	1	0	0
262	NLDRSKISLPDFDDE	1	2	36
263	DAPASKPSVPPRNYF	0	12	0
264	FYNRTAFQLPGDARV	0	5	0
265	AIPPPVPNRPGGTTN	0	1	0
266	SRQAIPPPVPNRPGG	0	3	0
267	NQARKPFLLPATELS	1	2	2
268	NRPAIRIPSLKKPAL	0	14	1
269	YYNRQELALPKRMSS	2	7	3
270	KWVRRPKLMTPLTFS	0	44	1
271	YSRKKLPVVLSKMTL	4	12	2
272	VKPRQLFPIPLNKVD	0	0	0
273	HPRFVSPRIPSRIVK	0	15	0
274	TYNRTAFQIPGDQSI	0	1	0
275	DGNEEKPLLPTRPNK	0	5	0
276	NNNKMRPHLPPLSSG	0	0	0
277	NILKRRPLKLEAPSK	0	28	0
278	FSRRQSMDIPSKNRN	27	1	1
279	NMLSRPRLAKLPSIR	1	25	0
280	NVLRKILEIPLHSFS	0	0	0
281	NIKVLLNPKLGKPVK	0	8	0
282	VSKLRSPNTPRRLRK	0	7	1
283	SPAPIVPREPLRNEP	78	3054	337
284	RHLKNIPRIPIFSIL	0	402	4
285	ATNVPVAPPPPPASL	0	0	0
286	VPVAPPPPPASLGQS	0	0	0
287	RNNRPVPPPPPMRTT	1	0	0
288	RPVPPPPPMRTTTEG	0	2	0
289	SGVRLPAPPPPPRRG	3	0	0
290	RLPAPPPPPRRGPAP	0	1	0
291	RRGPAPPPPPRASRP	0	14	1
292	PAPPPPPRASRPTPN	2	0	1
293	TKHKAPPPPPPTAET	1	0	0
294	LYEAMPPTLPHRDWK	1	0	0
295	QPWTDQFEKLEKEVS	0	0	0
	control	653	3972	1003
	control	615	4864	978
	control	540	5475	1008
	control	712	6580	1095
	control	651	4938	1031

Sla1-2

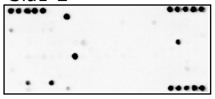

Sla1-3

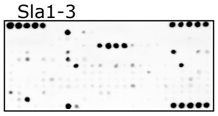

GST/Ab

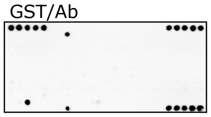

Supplement: Table S7 — SPOT intensities for yeast SH3 domain ligands predicted by regular expressions. The peptides predicted by the set of regular expressions (Table S6) were tested against 26 GST-SH3 fusion proteins by SPOT. From the panel of 2,953 predicted ligands, 295 showed a positive signal with at least one SH3 domain (numbered from 1 to 295). These ligands were re-arrayed and retested. The signal intensity of each predicted ligand against each GST-SH3 fusion protein is reported. Each array also contains a control peptide (LASDLIVPRR that reacts with the anti-GST antibody), which has been spotted in pentuplicate at the top left, top right, and bottom right of the array. In addition, GST was tested as a control to identify nonspecific interactions. The blot from the SPOT experiment is shown for each domain. (1.09 MB PDF) [file pbio.1000218.s016.pdf]
