# Supplementary material for: Bayesian Modeling of the Yeast SH3 Domain Interactome Predicts Spatiotemporal Dynamics of Endocytosis Proteins
Source: PLoS Biol. 2009 Oct 20;7(10):e1000218. doi: 10.1371/journal.pbio.1000218 (PMC2756588; doi:10.1371/journal.pbio.1000218)
Supplement: Table S8 — SPOT intensities for the top ten highest scoring yeast SH3 domain ligands based on PWM matches. SPOT intensities were measured for the ten best-predicted matches for each SH3 domain specificity profile based on a PWM scoring algorithm. For domains that recognized more than one set of ligands, the ten best-predicted ligands from each PWM is shown. The peptides were arrayed in the order indicated, where the 11th peptide represents a synthetic, phage-optimized peptide. The peptides were arrayed in duplicate, and the signal intensity from each SPOT experiment is shown. For domains that recognized two sets of ligands, the first row of the array represents peptide ligands predicted by the first PWM, whereas the second row contains peptide ligands predicted by the second PWM. In these cases, rows 3 and 4 of the array are duplicates of rows 1 and 2, respectively. The specificity profile used to generate each PWM shown below the blot from the SPOT experiment. Sla1-1/2-W41S and Sla1-1/2-W108S represent the two point mutations made in the Sla1-1/2 construct to determine peptide ligand interactions for each SH3 domain individually. (0.62 MB PDF) [file pbio.1000218.s017.pdf]

Table S8

Table S8. SPOT intensities for the top ten highest scoring yeast SH3 domain ligands based on PWM matches

SH3 domain	Peptide sequence	Signal Intensity 1	Signal Intensity 2	Signal Intensity Average
**Abp1**	TKPTPPPKPSHLKPKP	2005	1985	1995
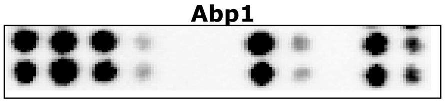	RPPRPPPKPLHLRTEI	2271	2595	2433
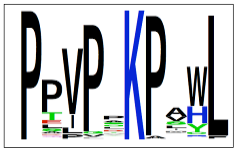	PRPPVKSKPKHLQDGT	2006	1619	1812
	EKPPVVKKPHYLSVAA	174	248	211
	LPPKFPEKPVHLVIMT	0	0	0
	NNPPKPQKPVPLNVLQ	0	0	0
	LKPKPPPKPLLLAGRK	2639	2713	2676
	SKPSLPEKPQKLRNAN	316	225	270
	QLPPGVKKPVHLLKNA	0	0	0
	GPPPRPKKPSTLKTKR	1722	1574	1648
	PPVPPKPAWL	531	605	568

SH3 domain	Peptide sequence	Signal Intensity 1	Signal Intensity 2	Signal Intensity Average
**Bbc1**	ESAPEVLPRPAALNSS	0	0	0
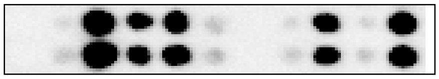	LPVPPPPVRPSISFNE	18	61	39
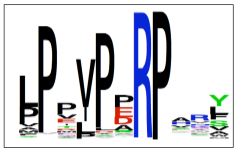	EVTPKVPERPSRRKTS	2846	3379	3112
	PIMPTLPPRPYITINE	785	724	755
	AIPPPVPNRPGGTTNR	1930	1747	1839
	TTPPPVLRRPSVTAAQ	77	95	86
	PPPPPPPPVPAKLFGE	0	0	0
	MPLPQLPKSPSRYSLS	28	56	42
	ELPPIKPPRPTSTTSV	1006	1117	1062
	LVPPALGPRPLLFTAS	10	44	27
	LPPVPPRPARY	1674	1592	1633

SH3 domain	Peptide sequence	Signal Intensity 1	Signal Intensity 2	Signal Intensity Average
**Bem1-1**	NNGVPPVSPYGQATIN	0	0	0
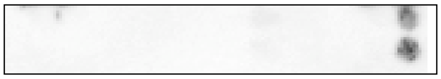	TFLGPPGTPYEGGKFV	6	0	3
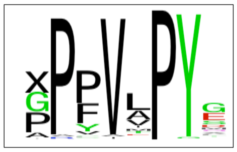	QESPAFVYPYSLWKYT	0	0	0
	SIVPPPVLPILDNIIR	0	0	0
	NFMGPEALPYDVQGTD	0	0	0
	IPSPPPVAPTLSVRTE	1	0	1
	KKKSPKVTPYERNTLR	6	0	3
	KIPIPPRAPYGGELVV	0	2	1
	DIETPFILPYLMEKAG	0	0	0
	NLNGPFVVPRDTGKFD	1	0	1
	GPPVLPYG	40	60	50

SH3 domain	Peptide sequence	Signal Intensity 1	Signal Intensity 2	Signal Intensity Average
**Bem1-2**	VKERRPPPPPPLLYST	143	162	152
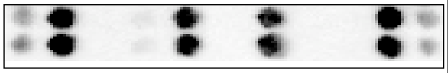	RLRKRPPPPPPVSMPT	804	850	827
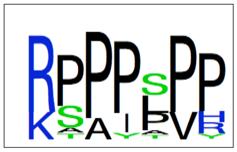	KNPTKSPPPPPSPSTM	0	0	0
	EKVKRTPPLPPVPEGM	29	27	28
	**RHSKKPAPPPPGMQNK**	**543**	**607**	**575**
	YDSNRPAILPPLYGIP	0	0	0
	RPKRRAPPPVPKKPSS	466	498	482
	TTKHKAPPPPPPTAET	0	0	0
	KRKAKAPPPPPPPPPS	0	0	0
	IKMSRSPPRPPSLKII	911	736	823
	RPPPSPP	135	116	126

Table S8

		Signal Intensity		
SH3 domain	Peptide sequence	1	2	Average
Boi1	AKLPPPRSSARLSKSL	3947	4538	4242
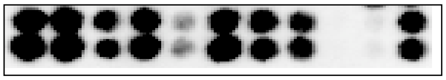	PVLPPPRSPNRPTLSD	3255	4368	3812
	REVIPHRNPSRLLNDI	2226	2776	2501
	GLTIPERSSLRRSKFN	3222	4530	3876
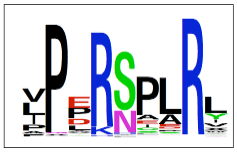	GTYVPERSTARAHDQL	472	481	476
	SIEIPKRSPLRFTSSP	3591	4715	4153
	APDIPPRSPNRNAHSR	2522	2734	2628
	SFVLPPRSAERKAHIK	1948	2177	2062
	QVALPSKNPERINDKN	3	0	2
	NWKLPPRLPHRAAQRR	66	51	58
	VPERSPLRL	2894	2799	2847

		Signal Intensity		
SH3 domain	Peptide sequence	1	2	Average
Boi2	SIEIPKRSPLRFTSSP	4036	4102	4069
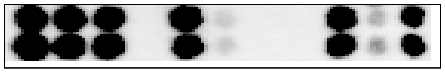	APDIPPRSPNRNAHSR	3626	3368	3497
	PVLPPPRSPNRPTLSD	3263	3535	3399
	HWGVPPPYLLRHAFNK	0	0	0
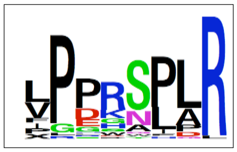	AKLPPPRSSARLSKSL	3573	3108	3341
	RSGLPPSQLARIWTLC	136	122	129
	ASLLPPRSSILREFLSL	0	0	0
	IPSVPLSNLLRIYQSA	0	0	0
	GLTIPERSSLRRSKFN	3013	3065	3039
	GTYVPERSTARAHDQL	192	295	244
	LPPRSPLR	2635	2317	2476

		Signal Intensity		
SH3 domain	Peptide sequence	1	2	Average
Bzz1-1	RKAKAPPPPPPPPPSR	612	533	572
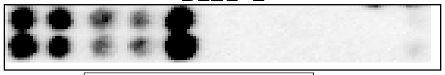	DHPKGPPPPPPPDEKD	280	189	234
	DHPKGPPPPPPPPDEK	181	191	186
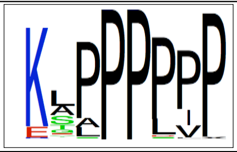	RLRKRPPPPPPVSMPT	1199	1568	1383
	KLPQLPPPPPPPPPPP	0	1	1
	LKPPLPPPPPPQPASN	0	0	0
	SDLALPPPPPPPPGYE	4	1	3
	QPQHLPPPPPPRAQQQ	0	0	0
	PLAPLPPPPPPSVATS	0	0	0
	KLPPPPPP	16	34	25

		Signal Intensity		
SH3 domain	Peptide sequence	1	2	Average
Bzz1-2	TRRRPPPPPIPSTQKP	1093	1245	1169
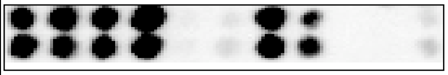	TKHKAPPPPPPTAETF	983	805	894
	RKAKAPPPPPPPPPSR	1381	1223	1302
	RLRKRPPPPPPVSMPT	1971	1586	1778
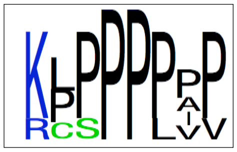	DHPKGPPPPPPPDEKD	25	19	22
	DHPKGPPPPPPPPDEK	38	68	53
	LRKRPPPPPPVSMPTT	1567	1567	1567
	VKERRPPPPPPLLYST	424	632	528
	LSSQPPPPPPPPPPVP	0	0	0
	QANIPPPPPPPPPSSK	0	0	0
	KLPPPPPP	37	104	70

Table S8

		Signal Intensity		
**SH3 domain**	**Peptide sequence**	**1**	**2**	**Average**
**Cyk3**	**Class I PWM**			
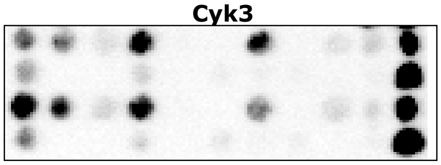	LAYERPLPDLPSTIKP	262	605	433
	NRNNRPVPPPPPMRTT	209	382	295
	PKKSRVLPPLPFPLYD	40	60	50
	TYLTRPLPSTPNEDSR	516	488	502
	LPPLRSLPVLVGKKKL	6	1	4
	SELVRKLPSGVSPQIM	0	0	0
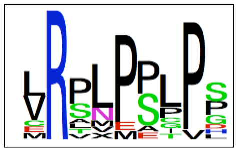	SMAMRPIPPLPTESEY	436	252	344
	YSYLRLLPWWPSLANE	0	0	0
**Class I profile**	PIRLRKRPPPPPPVSM	41	56	49
	TEFSRPIPPTPSGFVV	65	36	50
	LRPLPPLPS	796	970	883
	**Class II PWM**			
	SILLPPLPERAYIMDP	155	195	175
	ELVKPDVPIVDYLTRY	0	0	0
	VPWGPPLETKDYLLRR	0	0	0
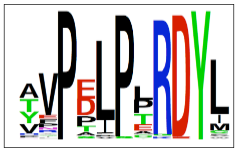	VPIMPTLPPRPYITIN	68	43	55
	QPPLPPIPTRDDMSNY	0	0	0
	KQVKPDIKKRDYLNKI	12	25	18
**Class II profile**	YRFNPAIPLRIYLKTC	9	7	8
	KSNFPDFPLKKYLKNN	3	10	6
	LFALPTLPHIYYLQVD	0	0	0
	FLRRPIFPGRDYRHQL	0	0	0
	AVPDLPPRDYL	1287	1338	1312

		Signal Intensity		
**SH3 domain**	**Peptide sequence**	**1**	**2**	**Average**
**Fus1**	TLQRQPRTISLFTNDI	146	371	258
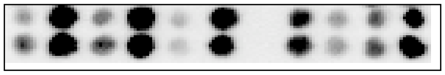	INGRRPRSSSLQSYTN	1784	1651	1717
	AAGRAIRTSSLYSTMI	282	472	377
	**SRVTRRTTSLVNNIL**	**1957**	**2133**	**2045**
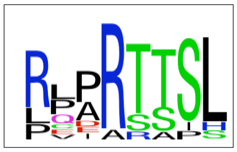	GRHPLSRTSSLIDSIG	127	90	109
	SLMRPRRSSSLFSNES	1364	1305	1334
	PSQRVARPTSLHPFNI	0	0	0
	DLERQMRSSSLDSFSP	753	777	765
	LRVVLPRTASLQSSNT	236	233	234
	KISLPKRSTSLLKSKR	419	467	
	RLPRTTSL	1105	1942	1523

		Signal Intensity		
**SH3 domain**	**Peptide sequence**	**1**	**2**	**Average**
**Hof1**	PEHVPLLPPLPRWEEI	1548	1508	1528
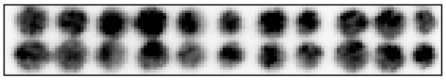	NNPLPPLPPLPDLDNM	1667	1389	1528
	APNEPLLPPLPGQPPL	2005	1200	1603
	GLNSPKLPPLPTTSNS	2060	1562	1811
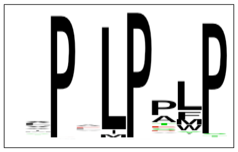	PTTAPALPSLPPPPLL	1720	1281	1500
	QNSTPVLPTLPQNVPI	1554	1550	1552
	KNELPSLPMLPSEATL	1975	1606	1791
	VVKLPQLPPPPPPPPP	1721	1456	1589
	RSKFPSLPSLPIFLSL	1694	1598	1646
	APALPSLPPPPLLNVD	1833	1679	1756
	LPALPPLP	1410	1636	1523

		Signal Intensity		
**SH3 domain**	**Peptide sequence**	**1**	**2**	**Average**
**Hse1**	SLPVPQLPPKLLVYPE	375	441	408
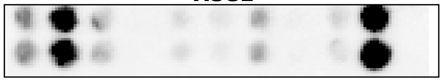	PEIPPPLPPKIMVHSQ	1721	1463	1592
	FVILPQIPPKLTFILT	235	216	225
	MPSNPALPKKLLNVPY	7	0	3
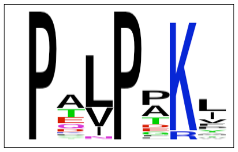	QDQAPSLPPKPNTQLQ	86	79	83
	PPPPPPVPAKLFGESL	67	34	51
	FRMIPELPPKRIGSQN	284	241	263
	PLQIPPLPLKLLTPVP	15	5	10
	YVTVPKLPFKLAVNKF	106	82	94
	ESKPPQLPPKCSSLRK	2667	2742	2704
	PALPPKL	0	0	0

Table S8

		Signal Intensity		
SH3 domain	Peptide sequence	1	2	Average
Lsb1	**Unique PWM**			
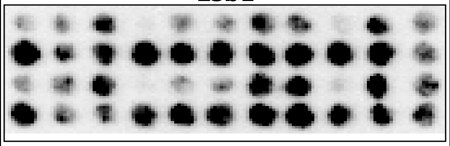	KLEDRLGLPLPAFVVL	91	149	120
	YLAYRPPMMLPTETLN	144	227	186
	SNSMRPPLLIPAATTK	380	482	431
	TLPSRLSPPLPFSVEP	6	5	6
	GFDYRLAMALPDMWIK	165	104	134
	LDAPRPPLPQPMKQEV	146	116	131
	KLIGRPPLLVPGMTPC	338	509	423
	RTSSRASLALPFQLRL	299	807	553
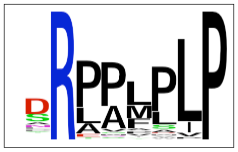 **Unique profile**	KDKSRPPRPPPKPLHL	38	25	32
	ANQARKPFLLPATELS	436	933	685
	DRPPLPLP	181	329	255
	**Class II PWM**			
	TNRGPPPLPPRANVQP	1144	712	928
	PQHLPPPPPPRAQQQQ	279	205	242
	VRLPAPPPPPRRGPAP	421	240	330
	RSSAAPPPPPRRATPE	945	457	701
	PTTSSPPLPPRQNVAT	818	867	842
	KRITSPPLPPRADCIE	677	555	616
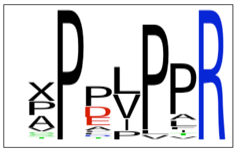 **Class II profile**	AETSAPDIPPRSPNRN	1018	947	983
	SSSTPPTLPPRRIEDP	994	1020	1007
	PTSGPPLLPPRNTMKN	910	577	743
	SSSSPPPLPTRRDHIK	937	661	799
	PPPLPPR	270	390	330

		Signal Intensity		
SH3 domain	Sequence	1	2	Average
Lsb3	NRGPPPLPPRANVQPP	2742	2670	2706
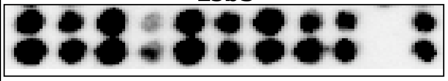	RITSPPLPPRADCIEE	1578	2335	1957
	TTSSPPLPPRQNVATS	2582	3074	2828
	SILLPPLPERAYIMDP	419	689	554
	TSGPPLLPPRNTMKNA	2435	3293	2864
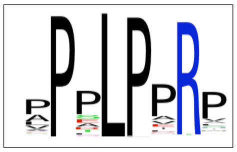	VPIMPTLPPRPYITIN	1500	1337	1418
	SSTPPTLPPRRIEDPL	2661	2710	2686
	RVAPPPLPNRQLPNLD	1152	1769	1460
	SWKPPDLPIRLRKRPP	1150	1982	1566
	TGPPPPLPPPLFPSSS	0	0	0
	PPPLPPRP	1357	1688	1522

Table S8

		Signal Intensity		
SH3 domain	Sequence	1	2	Average
Lsb4	NRGPPPLPPRANVQPP	1790	1666	1728
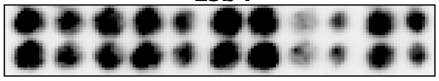	VPIMPTLPPRPYITIN	1067	1033	1050
	RITSPPLPPRADCIEE	1225	1164	1195
	TSGPPLLPPRNTMKNA	1416	1480	1448
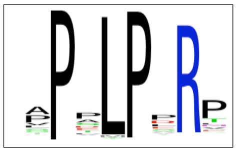	EEIAPSLPSRNSIPAP	846	796	821
	TTSSPPLPPRQNVATS	1782	1573	1678
	SSTPPTLPPRRIEDPL	1834	1647	1740
	SILLPPLPERAYIMDP	399	540	
	FLNSPDLPERTKLRNI	501	535	518
	SSSPPPLPTRRDHIKI	1440	1120	1280
	APPLPPRP	993	722	857

		Signal Intensity		
SH3 domain	Sequence	1	2	Average
Myo3	PPPPPPPPPPVPAKLF	1789	1406	1597
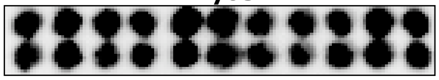	IPSPSSSPPPIPKTAN	3122	2187	2655
	PPPPPPPPPPLPQSLL	2240	1270	1755
	ASAPTTPAPALPPASP	2143	1708	1926
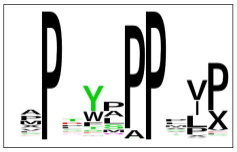	PNAPLSPAPAVPSIPS	3359	2255	2807
	SPAPPPPPPPPPPPPM	1991	1398	1694
	AKAPPPPPPPPPSRKC	1720	1396	1558
	MPAPPPPPPPPPGAFS	1705	1091	1398
	SQPPPPPPPPPPVPAK	2048	2093	2070
	QLPPPPPPPPPPPLPQ	2900	2249	2574
	APTYPPPPVP	2468	2058	2263

		Signal Intensity		
SH3 domain	Sequence	1	2	Average
Myo5	PPPPPPPPPPVPAKLF	977	1159	1068
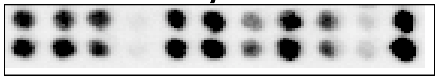	PPPPPPPPPPLPQSLL	857	1273	1065
	SSMPAPPPPPPPPPGA	862	730	796
	APAPPPPPPAPPASVF	51	36	43
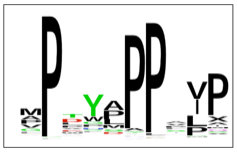	SQPPPPPPPPPPVPAK	1155	1475	1315
	QLPPPPPPPPPPPLPQ	1304	1871	1588
	MPAPPPPPPPPPGAFS	509	589	549
	AKAPPPPPPPPPSRKC	1285	1545	1415
	TSAPAPPPPPLPAAMS	664	582	623
	AGAPAPPPPPPPPALG	122	164	143
	MPTYAPPAVP	1759	2698	2228

		Signal Intensity		
SH3 domain	Sequence	1	2	Average
Nbp2	VFYHRPAPKPPVTKKV	2134	1658	1896
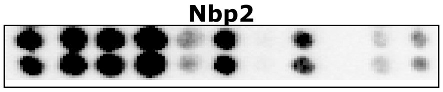	FRPHRLAPSAPATKNH	2974	2122	2548
	FQPQRTAPKPPISAPR	3695	3230	3462
	FIPSRPAPKPPSSASA	4123	3981	4052
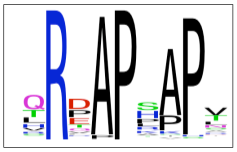	RFSSRRAPIAPSGKYY	464	298	381
	LAPKREAPKPPANTSP	2429	2347	
	VKERRPPPPPPLLYST	23	6	14
	FVSPRRAPKPPSYPSP	943	925	934
	TGESRKAPLIPLLKQT	0	0	0
	RLRKRPPPPPPVSMPT	168	186	177
	QRDAPSAPV	371	308	340

Table S8

SH3 domain	Sequence	Signal Intensity 1	Signal Intensity 2	Signal Intensity Average
**Pex13**	**Class II PWM**			
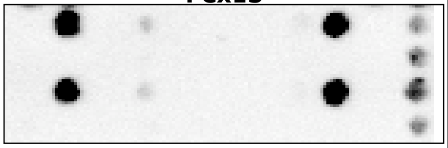	EEKPLLPTRPNKAEVT	0	2	1
	PIMPTLPPRPYITINE	769	637	703
	GPPPAMPARPTATTET	0	1	1
	TSSPSLPTRFTPCPVA	34	33	34
	DQAPSLPPKPNTQLQQ	1	1	1
	LPVPPPPVRPSISFNE	0	0	0
	TSKPSLPEKPQKLRNA	2	1	1
	YSLPMHPGRWWVHTRG	19	13	16
	DSLPKLPFRSWGQPYT	1008	806	907
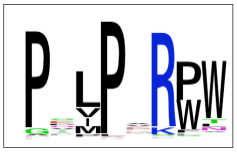 **Class II profile**	CILPSTPTRPLSQSKM	0	0	0
	PSLPARPW	101	306	203
	**Unique PWM**			
	TSYIEAIRPQWLSYPE	0	0	0
	LDVVSHSRPSWLPPKD	0	0	0
	GFNEVFSRPEWMILTC	0	0	0
	DVEGKPLRPKWIYQGE	4	4	4
	IRTVTSVRPEWLIEIA	0	0	0
	YETPVKVRPGWVIVSP	2	0	1
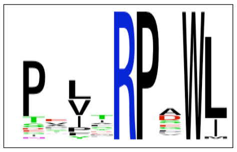 **Unique profile**	GVLLYRIRPKWLRGIL	0	0	0
	GILLYNIRPKWLRSIL	3	0	2
	LKAVSHFRPSWLPPKD	0	0	0
	LRPDHASRPLWISPSD	0	0	0
	PELTRPAWL	161	114	137

SH3 domain	Sequence	Signal Intensity 1	Signal Intensity 2	Signal Intensity Average
**Pin3**	**Unique PWM**			
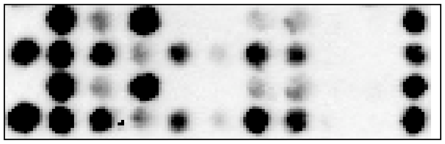	IDEYDNPLLPLPFNFN	0	0	0
	SDYYNRQELALPKRMC	1256	1358	
	LHEYAREGKPLPRAIE	189	131	160
	DSPYHRCPFLLPLFYQ	1173	890	1031
	HKLEDRLGLPLPAFVV	4	0	2
	LSSPARDILPLPPKTA	0	0	0
	GGITDRKLYPLPLYNH	73	104	88
	APWYFRSGNPLPHCVR	128	140	134
	GVEYLREYLNLPEHIV	2	4	3
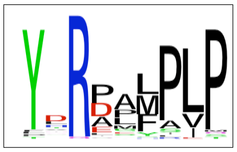 **Unique profile**	GVEYLREYLNLPEHIV	2	6	4
	YDRPALPLP	777	806	792
	**Class II PWM**			
	RITSPPLPPRADCIEE	940	1553	1246
	NRGPPPLPPRANVQPP	1355	1360	1358
	TTSSPPLPPRQNVATS	723	708	715
	SILLPPLPERAYIMDP	140	157	
	EREEPPLPKRIRISKI	330	310	320
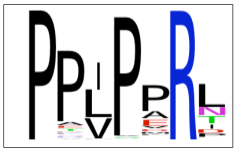 **Class II profile**	QPPLPPIPTRDDMSNY	41	30	36
	TSGPPLLPPRNTMKNA	486	858	672
	VPIMPTLPPRPYITIN	278	422	350
	EAAQPPLPSRNVASGA	4	3	4
	EEEHPPLPARRKSEEE	0	0	0
	PPIPPRL	449	994	722

Table S8

		Signal Intensity		
**SH3 domain**	**Sequence**	**1**	**2**	**Average**
**Rvs167**	**Class I PWM**			
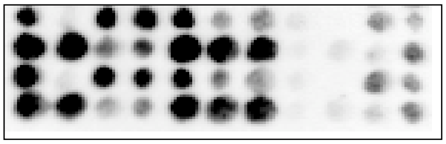	NRNNRPVPPPPPMRTT	1037	1580	1308
	LAYERPLPDLPSTIKP	35	0	18
	PQQNRPLPQLPNRNNR	1221	1265	1243
	SETARKVPPIPTQIIN	1197	921	1059
	NAPERAVPILPPRNNV	1361	1117	1239
	ILPPRIVPAIPKKATV	343	376	
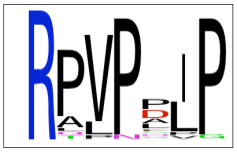	SMAMRPIPPLPTESEY	494	390	442
	RLRKRPPPPPPVSMPT	87	51	69
	VKERRPPPPPPLLYST	0	0	0
**Class I profile**	NNSTRPIPAIPMDLPD	427	603	515
	RPVPPIP	154	360	257
	**Class II PWM**			
	NRGPPPLPPRANVQPP	1618	1373	1495
	RITSPPLPPRADCIEE	1790	1290	1540
	SILLPPLPERAYIMDP	500	283	392
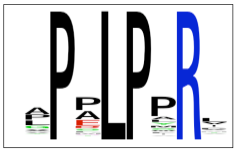	SWKPPDLPIRLRKRPP	677	394	535
	TTSSPPLPPRQNVATS	2461	1649	2055
**Class II profile**	TSGPPLLPPRNTMKNA	1692	990	1341
	SSTPPTLPPRRIEDPL	1319	1120	1219
	EEAAPQLPSRSSAAPP	40	35	37
	NFLLPNLPMRTFKEIV	93	90	91
	RPYPPSLPSRDLYEVT	101	151	126
	APPLPPRL	569	496	532

		Signal Intensity		
**SH3 domain**	**Sequence**	**1**	**2**	**Average**
**Sho1**	QIVNKPLPPLPVAGSS	852	648	750
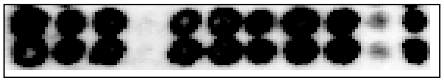	ESMNNPLPPLPPLPDL	1875	1798	1836
	LSRSKPLPLTPNSKYN	608	546	577
	DQDDEELPPLPVEAQT	4	0	2
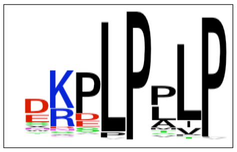	LAYERPLPDLPSTIKP	731	811	771
	PKKSRVLPPLPFPLYD	790	640	715
	KKEEQPLPPIPPTKSK	2106	1934	2020
	HEREKALPPIPFTTTL	1027	1434	1231
	HSTPKPLPPVPALSLS	1578	1721	1649
	HVPDLPLPTLPDRQLF	289	223	256
	DKPLPPLP	1607	2142	1874

		Signal Intensity		
**SH3 domain**	**Sequence**	**1**	**2**	**Average**
**Sla1-3**	DYKIPVVPPRETYFAD	31	35	33
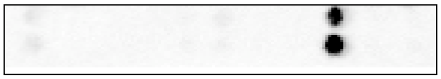	SWDKGPVPDREEFRNS	0	0	0
	EHGVPVVPSREVTINA	0	0	0
	PWVLLPVPPRLELLLP	1	4	3
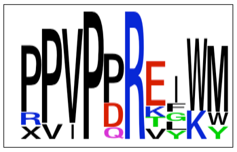	KVVIPPVPSRYSDERP	5	6	6
	QAIPPPVPNRPGGTTN	23	11	17
	VGGQPPVPVRMQPQPP	0	1	1
	IVKTIPVPPRDFMISP	2	1	1
	PASKPSVPPRNYFSKI	449	753	601
	RITSPPLPPRADCIEE	0	0	0
	PPVPPRE	2	6	4

Table S8

		Signal Intensity		
**SH3 domain**	**Sequence**	**1**	**2**	**Average**
**Sla1-1/2-W41S**	**Class I PWM**			
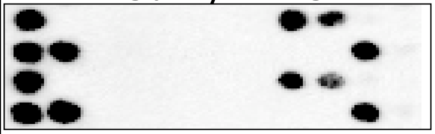	KAPPPPPPPPPSRKCG	1165	1481	1323
	NIPPPPPPPPPSSKPK	0	0	0
	PPPPPPPPPPPMALFG	0	0	0
	DHPKGPPPPPPPDEKD	0	1	0
	DHPKGPPPPPPPPDEK	0	0	0
	PAPPPPPPPPPGAFST	0	0	0
	NIPPPPPPPPPKPPLN	2	0	1
	TRRRPPPPPIPSTQKP	988	788	888
	LRKRPPPPPPVSMPTT	434	295	364
	NKPKPTPPSPPAKRIP	3	9	6
	PRPPPPPPPS	0	0	0
	**Class II PWM**			
	GRRGPAPPPPPRASRP	1398	1665	1531
	APPPPPPPPPSRKCGT	1387	1592	1490
	NIPPPPPPPPPKPPLN	2	0	1
	HPKGPPPPPPPDEKDR	8	0	4
	HPKGPPPPPPPPDEKG	0	0	0
	VLNSPPLPPPARSQSL	1	0	1
	GAPAPPPPPPPPALGG	0	0	0
	DSPAPPPPPPPPPPPP	0	0	0
	AAPAPPPPPPAPPASV	0	0	0
	GSITPPRPPPSRSSPK	1476	1317	1397
	GPPPPPPSR	11	12	12

		Signal Intensity		
**SH3 domain**	**Sequence**	**1**	**2**	**Average**
**Sla1-1/2-W108S**	**Class I PWM**			
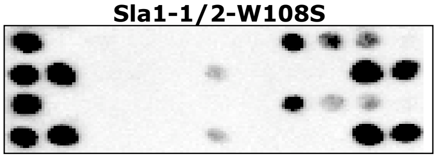	KAPPPPPPPPPSRKCG	1115	1367	1241
	NIPPPPPPPPPSSKPK	0	0	0
	PPPPPPPPPPPMALFG	1	0	0
	DHPKGPPPPPPPDEKD	0	0	0
	DHPKGPPPPPPPPDEK	0	1	0
	PAPPPPPPPPPGAFST	0	0	0
	NIPPPPPPPPPKPPLN	0	0	0
	TRRRPPPPPIPSTQKP	581	557	569
	LRKRPPPPPPVSMPTT	291	120	205
	NKPKPTPPSPPAKRIP	229	122	175
	PRPPPPPPPS	0	0	0
	**Class II PWM**			
	GRRGPAPPPPPRASRP	1267	1191	1229
	APPPPPPPPPSRKCGT	1428	1295	1362
	NIPPPPPPPPPKPPLN	2	0	1
	HPKGPPPPPPPDEKDR	6	0	3
	HPKGPPPPPPPPDEKG	0	0	0
	VLNSPPLPPPARSQSL	85	81	83
	GAPAPPPPPPPPALGG	0	0	0
	DSPAPPPPPPPPPPPP	2	0	1
	AAPAPPPPPPAPPASV	0	0	0
	GSITPPRPPPSRSSPK	2537	2480	2508
	GPPPPPPSR	788	1098	943

Table S8

		Signal Intensity		
**SH3 domain**	**Sequence**	**1**	**2**	**Average**
**Sla1-1/2**	**Class I PWM**			
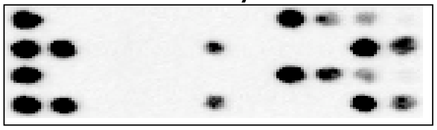	KAPPPPPPPPPSRKCG	1035	916	975
	NIPPPPPPPPPSSKPK	0	0	0
	PPPPPPPPPPPMALFG	0	0	0
	DHPKGPPPPPPPDEKD	1	0	0
	DHPKGPPPPPPPPDEK	0	0	0
	PAPPPPPPPPPGAFST	0	0	0
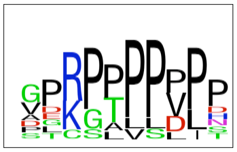 **Class I profile**	NIPPPPPPPPPKPPLN	0	0	0
	TRRRPPPPPIPSTQKP	916	951	933
	LRKRPPPPPPVSMPTT	210	373	291
	NKPKPTPPSPPAKRIP	125	141	133
	PRPPPPPPPS	3	11	7
	**Class II PWM**			
	GRRGPAPPPPPRASRP	1121	1123	1122
	APPPPPPPPPSRKCGT	1021	681	851
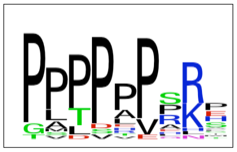 **Class II profile**	NIPPPPPPPPPKPPLN	2	0	1
	HPKGPPPPPPPDEKDR	3	0	2
	HPKGPPPPPPPPDEKG	0	0	0
	VLNSPPLPPPARSQSL	228	276	252
	GAPAPPPPPPPPALGG	0	0	0
	DSPAPPPPPPPPPPPP	0	0	0
	AAPAPPPPPPPAPPASV	0	0	0
	GSITPPRPPPPSRSSPK	1347	1058	1203
	GPPPPPPSR	394	282	338
