# Supplementary material for: Bayesian Modeling of the Yeast SH3 Domain Interactome Predicts Spatiotemporal Dynamics of Endocytosis Proteins
Source: PLoS Biol. 2009 Oct 20;7(10):e1000218. doi: 10.1371/journal.pbio.1000218 (PMC2756588; doi:10.1371/journal.pbio.1000218)
Supplement: Table S9 — Summary of yeast SH3 domains analyzed by yeast two-hybrid. Yeast SH3 domains were cloned into the pPC97 and pBDC/pOBD yeast two-hybrid expression vectors for use in the ORFeome and fragmented gDNA screening approaches, respectively. SH3 domains are named according to the gene name in which they were identified. SH3 domains from proteins with more than one domain are numbered from the N-terminus and demarcated from the protein name with a dash. The listed amino acid ranges indicate the length of the constructs used in this analysis and not necessarily the SH3 domain boundaries defined by computational analysis. Sla1-1/2-W41S and Sla1-1/2-W108S represent the two point mutations made in the Sla1-1/2 construct to determine the binding partners for each SH3 domain individually. Each domain was tested in both screening techniques. In most cases, the addition of a competitor of HIS3 gene product, 3-amino-1,2,4-triazole (3-AT) was added to reduce the level of basal transcription as indicated. The number of isolated hits is indicated for each SH3 domain. The SH3 domains that could not be cloned into either expression vector are also indicated. (0.03 MB PDF) [file pbio.1000218.s018.pdf]

Table S9. Summary of yeast SH3 domains analyzed by yeast two-hybrid

			SH3 domain boundaries					SH3 domain boundaries			
**SH3 Domain**	**ORF**	**ORFeome vector**	**Start**	**End**	**Screened**	**3-AT (mM)**	**gDNA vector**	**Start**	**End**	**Screened**	**3-AT (mM)**
Abp1	YCR088W	pPC97	535	592	No	>50	pOBD	351	592	Yes	1
Bbc1	YJL020C	pPC97	1	78	Yes	1	pOBD	1	90	Yes	1
Bem1 -1	YBR200W	pPC97	55	141	Yes	1	pOBD	51	240	Yes	25
Bem1-2	YBR200W	pPC97	155	261	No	>50	pOBD	1	551	Yes	1
Boi1	YBL085W	pPC97	1	86	Yes	1	pOBD	1	100	Yes	1
Boi2	YER114C	pPC97	32	116	Yes	3	pOBD	21	130	Yes	1
Bud14	YAR014C	pPC97	237	347	Yes	3	pBDC	237	347	Yes	25
Bzz1-1	YHR114W	pPC97	-	-	Not cloned	-	pOBD	476	564	Yes	1
Bzz1-2	YHR114W	pPC97	568	633	Yes	3	pOBD	562	633	Yes	1
Cdc25	YLR310C	pPC97	51	157	Yes	1	pBDC	51	157	Yes	3
Cyk3	YDL117W	pPC97	1	83	Yes	3	pOBD	1	90	Yes	3
Fus1	YCL027W	pPC97	432	512	Yes	1	pOBD	401	512	Yes	3
Hof1	YMR032W	pPC97	-	-	Not cloned	-	pOBD	576	669	Yes	3
Hse1	YHL002W	pPC97	208	285	Yes	3	pBDC	208	285	Yes	5
Lsb1	YGR136W	pPC97	45	122	Yes	3	pBDC	-	-	Not cloned	-
Lsb3	YFR024C-A	pPC97	385	451	Yes	3	pBDC	385	451	Yes	3
Lsb4	YHR016C	pPC97	402	468	Yes	1	pOBD	391	468	Yes	1
Myo3	YKL129C	pPC97	1119	1195	Yes	1	pOBD	1054	1271	Yes	1
Myo5	YMR109W	pPC97	1072	1160	Yes	1	pBDC	1072	1160	Yes	1
Nbp2	YDR162C	pPC97	103	180	Yes	1	pOBD	91	190	Yes	50
Pex13	YLR191W	pPC97	295	386	Yes	3	pOBD	286	386	Yes	3
Pin3	YPR154W	pPC97	53	122	Yes	1	pOBD	38	130	Yes	1
Rvs167	YDR388W	pPC97	419	482	Yes	1	pOBD	401	482	Yes	1
Sho1	YER118C	pPC97	287	367	No	>50	pOBD	281	368	Yes	25
Sla1-1/2	YBL007C	pPC97	-	-	Not cloned	-	pOBD	1	150	Yes	3
Sla1-1/2-W41S	YBL007C	pPC97	1	150	Yes	50	pBDC	1	150	Yes	50
Sla1-1/2-W108S	YBL007C	pPC97	1	150	Yes	10	pBDC	1	150	Yes	25
Sla1-3	YBL007C	pPC97	344	424	Yes	1	pOBD	336	435	Yes	1
