# Supplementary material for: Bayesian Modeling of the Yeast SH3 Domain Interactome Predicts Spatiotemporal Dynamics of Endocytosis Proteins
Source: PLoS Biol. 2009 Oct 20;7(10):e1000218. doi: 10.1371/journal.pbio.1000218 (PMC2756588; doi:10.1371/journal.pbio.1000218)
Supplement: Table S10 — Yeast SH3 domain interactors isolated by yeast two-hybrid. Interacting ORFs isolated by ORFeome or gDNA screening are shown for each SH3 domain, including the number of times a given interacting ORF was captured. The ORF and gene name encoded in each activation domain (AD) isolated from a positive yeast two-hybrid colony are indicated. SH3 domains are named according to the gene name in which they were identified. SH3 domains from proteins with more than one domain are numbered from the N-terminus and demarcated from the protein name with a dash. Sla1-1/2-W41S and Sla1-1/2-W108S represent the two point mutations made in the Sla1-1/2 construct to determine the binding partners for each SH3 domain individually. (0.11 MB PDF) [file pbio.1000218.s019.pdf]

# Table S10. Yeast SH3 domain interactors isolated by yeast two-hybrid

| SH3 Domain | AD ORF | AD gene name | Y2H library | Number of hits |
|---|---|---|---|---|
| Abp1 | YNL094W | APP1 | gDNA | 20 |
| Abp1 | YNL020C | ARK1 | gDNA | 1 |
| Abp1 | YPL115C | BEM3 | gDNA | 1 |
| Abp1 | YPL008W | CHL1 | gDNA | 1 |
| Abp1 | YOL089C | HAL9 | gDNA | 2 |
| Abp1 | YBR133C | HSL7 | gDNA | 1 |
| Abp1 | YDR277C | MTH1 | gDNA | 15 |
| Abp1 | YBL005W | PDR3 | gDNA | 2 |
| Abp1 | YIL095W | PRK1 | gDNA | 24 |
| Abp1 | YOR116C | RPO31 | gDNA | 1 |
| Abp1 | YDR303C | RSC3 | gDNA | 1 |
| Abp1 | YOR047C | STD1 | gDNA | 1 |
| Abp1 | YCR099C | YCR099C | gDNA | 2 |
| Bbc1 | YNR016C | ACC1 | gDNA | 2 |
| Bbc1 | YNL094W | APP1 | gDNA | 4 |
| Bbc1 | YIL159W | BNR1 | gDNA | 1 |
| Bbc1 | YJL194W | CDC6 | gDNA | 11 |
| Bbc1 | YMR162C | DNF3 | gDNA | 1 |
| Bbc1 | YLR206W | ENT2 | gDNA | 1 |
| Bbc1 | YKL109W | HAP4 | gDNA | 1 |
| Bbc1 | YDL240W | LRG1 | gDNA | 1 |
| Bbc1 | YDR386W | MUS81 | gDNA | 1 |
| Bbc1 | YMR109W | MYO5 | gDNA | 15 |
| Bbc1 | YBL027W | RPL19B | gDNA | 3 |
| Bbc1 | YCL031C | RRP7 | gDNA | 2 |
| Bbc1 | YER115C | SPR6 | gDNA | 1 |
| Bbc1 | YOR047C | STD1 | gDNA | 1 |
| Bbc1 | YIL144W | TID3 | gDNA | 7 |
| Bbc1 | YLR337C | VRP1 | gDNA | 10 |
| Bbc1 | YBR108W | YBR108W | gDNA | 1 |
| Bbc1 | YER172C-A | YER172C-A | gDNA | 1 |
| Bbc1 | YIR003W | YIR003W | gDNA | 1 |
| Bbc1 | YJL195C | YJL195C | gDNA | 1 |
| Bbc1 | YJR115W | YJR115W | gDNA | 1 |
| Bbc1 | YJR047C | ANB1 | ORFeome | 1 |
| Bbc1 | YGL190C | CDC55 | ORFeome | 6 |
| Bbc1 | YNL199C | GCR2 | ORFeome | 5 |
| Bbc1 | YER086W | ILV1 | ORFeome | 2 |
| Bbc1 | YGL035C | MIG1 | ORFeome | 2 |
| Bbc1 | YDR277C | MTH1 | ORFeome | 6 |
| Bbc1 | YPR072W | NOT5 | ORFeome | 1 |
| Bbc1 | YER059W | PCL6 | ORFeome | 1 |
| Bbc1 | YNL206C | RTT106 | ORFeome | 5 |
| Bbc1 | YNL025C | SSN8 | ORFeome | 6 |
| Bbc1 | YOR047C | STD1 | ORFeome | 6 |
| Bbc1 | YIL144W | TID3 | ORFeome | 2 |
| Bbc1 | YAL016W | TPD3 | ORFeome | 6 |

# Table S10

| SH3 Domain | AD ORF | AD gene name | Y2H library | Number of hits |
|---|---|---|---|---|
| Bbc1 | YGL250W | YGL250W | ORFeome | 1 |
| Bbc1 | YIR003W | YIR003W | ORFeome | 6 |
| Bbc1 | YJR023C | YJR023C | ORFeome | 1 |
| Bbc1 | YJR149W | YJR149W | ORFeome | 1 |
| Bem1-1 | YOL078W | AVO1 | gDNA | 5 |
| Bem1-1 | YMR287C | DSS1 | gDNA | 14 |
| Bem1-1 | YLL049W | LDB18 | gDNA | 1 |
| Bem1-1 | YDL028C | MPS1 | gDNA | 1 |
| Bem1-1 | YCL031C | RRP7 | gDNA | 2 |
| Bem1-1 | YOR129C | YOR129C | gDNA | 16 |
| Bem1-1 | YGL190C | CDC55 | ORFeome | 4 |
| Bem1-1 | YBR112C | CYC8 | ORFeome | 2 |
| Bem1-1 | YMR212C | EFR3 | ORFeome | 1 |
| Bem1-1 | YPL038W | MET31 | ORFeome | 6 |
| Bem1-1 | YDR277C | MTH1 | ORFeome | 6 |
| Bem1-1 | YKL040C | NFU1 | ORFeome | 1 |
| Bem1-1 | YNL025C | SSN8 | ORFeome | 2 |
| Bem1-1 | YOR047C | STD1 | ORFeome | 1 |
| Bem1-1 | YNR047W | YNR047W | ORFeome | 1 |
| Bem1-1 | YDR368W | YPR1 | ORFeome | 1 |
| Bem1-2 | YOL078W | AVO1 | gDNA | 2 |
| Bem1-2 | YBL085W | BOI1 | gDNA | 19 |
| Bem1-2 | YER114C | BOI2 | gDNA | 8 |
| Bem1-2 | YNL298W | CLA4 | gDNA | 1 |
| Bem1-2 | YMR287C | DSS1 | gDNA | 5 |
| Bem1-2 | YLR318W | EST2 | gDNA | 1 |
| Bem1-2 | YCL005W | LDB16 | gDNA | 1 |
| Bem1-2 | YBL005W | PDR3 | gDNA | 1 |
| Bem1-2 | YMR247C | RKR1 | gDNA | 1 |
| Bem1-2 | YHL007C | STE20 | gDNA | 9 |
| Bem1-2 | YDR306C | YDR306C | gDNA | 13 |
| Bem1-2 | YKR105C | YKR105C | gDNA | 1 |
| Bem1-2 | YNR071C | YNR071C | gDNA | 3 |
| Bem1-2 | YOR129C | YOR129C | gDNA | 6 |
| Boi1 | YNR016C | ACC1 | gDNA | 1 |
| Boi1 | YDR085C | AFR1 | gDNA | 36 |
| Boi1 | YER155C | BEM2 | gDNA | 12 |
| Boi1 | YLR314C | CDC3 | gDNA | 1 |
| Boi1 | YER164W | CHD1 | gDNA | 1 |
| Boi1 | YBR003W | COQ1 | gDNA | 1 |
| Boi1 | YDR036C | EHD3 | gDNA | 1 |
| Boi1 | YBR102C | EXO84 | gDNA | 5 |
| Boi1 | YJL155C | FBP26 | gDNA | 4 |
| Boi1 | YER032W | FIR1 | gDNA | 10 |
| Boi1 | YJR138W | IML1 | gDNA | 8 |
| Boi1 | YCL024W | KCC4 | gDNA | 3 |
| Boi1 | YGL201C | MCM6 | gDNA | 1 |
| Boi1 | YOL135C | MED7 | gDNA | 1 |
| Boi1 | YNL078W | NIS1 | gDNA | 15 |
| Boi1 | YDR329C | PEX3 | gDNA | 1 |
| Boi1 | YCL031C | RRP7 | gDNA | 3 |

# Table S10

| SH3 Domain | AD ORF | AD gene name | Y2H library | Number of hits |
|---|---|---|---|---|
| Boi1 | YER008C | SEC3 | gDNA | 4 |
| Boi1 | YIL144W | TID3 | gDNA | 1 |
| Boi1 | YLL040C | VPS13 | gDNA | 3 |
| Boi1 | YDR372C | VPS74 | gDNA | 1 |
| Boi1 | YBR108W | YBR108W | gDNA | 1 |
| Boi1 | YDR239C | YDR239C | gDNA | 8 |
| Boi1 | YER158C | YER158C | gDNA | 13 |
| Boi1 | YJR056C | YJR056C | gDNA | 1 |
| Boi1 | YJR061W | YJR061W | gDNA | 4 |
| Boi1 | YMR244W | YMR244W | gDNA | 1 |
| Boi1 | YNL234W | YNL234W | gDNA | 2 |
| Boi1 | YOL019W | YOL019W | gDNA | 7 |
| Boi1 | YOL070C | YOL070C | gDNA | 1 |
| Boi1 | YPL158C | YPL158C | gDNA | 11 |
| Boi1 | YML109W | ZDS2 | gDNA | 5 |
| Boi1 | YGL190C | CDC55 | ORFeome | 6 |
| Boi1 | YPL049C | DIG1 | ORFeome | 4 |
| Boi1 | YDR480W | DIG2 | ORFeome | 4 |
| Boi1 | YER124C | DSE1 | ORFeome | 6 |
| Boi1 | YOL156W | HXT11 | ORFeome | 3 |
| Boi1 | YPL038W | MET31 | ORFeome | 4 |
| Boi1 | YGL035C | MIG1 | ORFeome | 2 |
| Boi1 | YDR277C | MTH1 | ORFeome | 6 |
| Boi1 | YFR034C | PHO4 | ORFeome | 2 |
| Boi1 | YMR139W | RIM11 | ORFeome | 6 |
| Boi1 | YNL206C | RTT106 | ORFeome | 2 |
| Boi1 | YER081W | SER3 | ORFeome | 6 |
| Boi1 | YKL218C | SRY1 | ORFeome | 2 |
| Boi1 | YOR047C | STD1 | ORFeome | 2 |
| Boi1 | YJR056C | YJR056C | ORFeome | 2 |
| Boi1 | YMR124W | YMR124W | ORFeome | 6 |
| Boi1 | YOL070C | YOL070C | ORFeome | 6 |
| Boi2 | YDR085C | AFR1 | gDNA | 83 |
| Boi2 | YER032W | FIR1 | gDNA | 1 |
| Boi2 | YJR138W | IML1 | gDNA | 2 |
| Boi2 | YMR080C | NAM7 | gDNA | 3 |
| Boi2 | YNL078W | NIS1 | gDNA | 10 |
| Boi2 | YIL144W | TID3 | gDNA | 1 |
| Boi2 | YER158C | YER158C | gDNA | 9 |
| Boi2 | YMR244W | YMR244W | gDNA | 2 |
| Boi2 | YPL158C | YPL158C | gDNA | 8 |
| Boi2 | YML109W | ZDS2 | gDNA | 1 |
| Boi2 | YGL190C | CDC55 | ORFeome | 4 |
| Boi2 | YPL049C | DIG1 | ORFeome | 2 |
| Boi2 | YDR480W | DIG2 | ORFeome | 2 |
| Boi2 | YER124C | DSE1 | ORFeome | 4 |
| Boi2 | YPL038W | MET31 | ORFeome | 2 |
| Boi2 | YDR259C | YAP6 | ORFeome | 2 |
| Boi2 | YMR124W | YMR124W | ORFeome | 4 |
| Bud14 | YDR085C | AFR1 | gDNA | 15 |
| Bud14 | YGR225W | AMA1 | gDNA | 2 |

# Table S10

| SH3 Domain | AD ORF | AD gene name | Y2H library | Number of hits |
|---|---|---|---|---|
| Bud14 | YER032W | FIR1 | gDNA | 1 |
| Bud14 | YLR096W | KIN2 | gDNA | 3 |
| Bud14 | YDL240W | LRG1 | gDNA | 1 |
| Bud14 | YJL042W | MHP1 | gDNA | 7 |
| Bud14 | YOL033W | MSE1 | gDNA | 1 |
| Bud14 | YMR026C | PEX12 | gDNA | 1 |
| Bud14 | YLR273C | PIG1 | gDNA | 1 |
| Bud14 | YDR195W | REF2 | gDNA | 2 |
| Bud14 | YCL031C | RRP7 | gDNA | 6 |
| Bud14 | YOR329C | SCD5 | gDNA | 9 |
| Bud14 | YLR430W | SEN1 | gDNA | 1 |
| Bud14 | YER115C | SPR6 | gDNA | 1 |
| Bud14 | YIL144W | TID3 | gDNA | 1 |
| Bud14 | YJR115W | YJR115W | gDNA | 3 |
| Bud14 | YKR075C | YKR075C | gDNA | 5 |
| Bud14 | YMR018W | YMR018W | gDNA | 1 |
| Bud14 | YMR258C | YMR258C | gDNA | 4 |
| Bud14 | YOR062C | YOR062C | gDNA | 10 |
| Bud14 | YMR104C | YPK2 | gDNA | 1 |
| Bud14 | YGR037C | ACB1 | ORFeome | 4 |
| Bud14 | YDL070W | BDF2 | ORFeome | 4 |
| Bud14 | YLR245C | CDD1 | ORFeome | 4 |
| Bud14 | YLR098C | CHA4 | ORFeome | 4 |
| Bud14 | YHL048W | COS8 | ORFeome | 4 |
| Bud14 | YPL049C | DIG1 | ORFeome | 4 |
| Bud14 | YDR480W | DIG2 | ORFeome | 4 |
| Bud14 | YOR178C | GAC1 | ORFeome | 4 |
| Bud14 | YOR355W | GDS1 | ORFeome | 4 |
| Bud14 | YGL237C | HAP2 | ORFeome | 2 |
| Bud14 | YOR358W | HAP5 | ORFeome | 4 |
| Bud14 | YOR284W | HUA2 | ORFeome | 4 |
| Bud14 | YDR277C | MTH1 | ORFeome | 4 |
| Bud14 | YBR186W | PCH2 | ORFeome | 4 |
| Bud14 | YLR273C | PIG1 | ORFeome | 4 |
| Bud14 | YCL028W | RNQ1 | ORFeome | 4 |
| Bud14 | YNL206C | RTT106 | ORFeome | 4 |
| Bud14 | YFR040W | SAP155 | ORFeome | 4 |
| Bud14 | YNL333W | SNZ2 | ORFeome | 4 |
| Bud14 | YFL059W | SNZ3 | ORFeome | 4 |
| Bud14 | YNL025C | SSN8 | ORFeome | 4 |
| Bud14 | YOR047C | STD1 | ORFeome | 4 |
| Bud14 | YML052W | SUR7 | ORFeome | 4 |
| Bud14 | YIL144W | TID3 | ORFeome | 4 |
| Bud14 | YDR100W | TVP15 | ORFeome | 4 |
| Bud14 | YMR071C | TVP18 | ORFeome | 4 |
| Bud14 | YNL229C | URE2 | ORFeome | 4 |
| Bud14 | YOR229W | WTM2 | ORFeome | 4 |
| Bud14 | YDR259C | YAP6 | ORFeome | 4 |
| Bud14 | YDR010C | YDR010C | ORFeome | 2 |
| Bud14 | YDR107C | YDR107C | ORFeome | 4 |
| Bud14 | YNL044W | YIP3 | ORFeome | 4 |

# Table S10

| SH3 Domain | AD ORF | AD gene name | Y2H library | Number of hits |
|---|---|---|---|---|
| Bud14 | YLR108C | YLR108C | ORFeome | 4 |
| Bzz1-1 | YNL094W | APP1 | gDNA | 6 |
| Bzz1-1 | YOR181W | LAS17 | gDNA | 30 |
| Bzz1-1 | YDL146W | LDB17 | gDNA | 3 |
| Bzz1-1 | YMR109W | MYO5 | gDNA | 18 |
| Bzz1-1 | YCL031C | RRP7 | gDNA | 1 |
| Bzz1-1 | YIL156W | UBP7 | gDNA | 50 |
| Bzz1-1 | YGR114C | YGR114C | gDNA | 1 |
| Bzz1-1 | YOR389W | YOR389W | gDNA | 3 |
| Bzz1-2 | YNR016C | ACC1 | gDNA | 1 |
| Bzz1-2 | YLR127C | APC2 | gDNA | 1 |
| Bzz1-2 | YNL094W | APP1 | gDNA | 2 |
| Bzz1-2 | YDR270W | CCC2 | gDNA | 3 |
| Bzz1-2 | YGL238W | CSE1 | gDNA | 3 |
| Bzz1-2 | YIR023W | DAL81 | gDNA | 1 |
| Bzz1-2 | YGR227W | DIE2 | gDNA | 1 |
| Bzz1-2 | YMR162C | DNF3 | gDNA | 2 |
| Bzz1-2 | YBR177C | EHT1 | gDNA | 1 |
| Bzz1-2 | YNL084C | END3 | gDNA | 1 |
| Bzz1-2 | YMR232W | FUS2 | gDNA | 1 |
| Bzz1-2 | YLR081W | GAL2 | gDNA | 1 |
| Bzz1-2 | YOR109W | INP53 | gDNA | 4 |
| Bzz1-2 | YOR181W | LAS17 | gDNA | 22 |
| Bzz1-2 | YJL134W | LCB3 | gDNA | 1 |
| Bzz1-2 | YDL146W | LDB17 | gDNA | 4 |
| Bzz1-2 | YCL034W | LSB5 | gDNA | 4 |
| Bzz1-2 | YGL201C | MCM6 | gDNA | 1 |
| Bzz1-2 | YGL178W | MPT5 | gDNA | 2 |
| Bzz1-2 | YMR109W | MYO5 | gDNA | 18 |
| Bzz1-2 | YKR103W | NFT1 | gDNA | 7 |
| Bzz1-2 | YPR052C | NHP6A | gDNA | 1 |
| Bzz1-2 | YIL013C | PDR11 | gDNA | 3 |
| Bzz1-2 | YLR324W | PEX30 | gDNA | 1 |
| Bzz1-2 | YGR004W | PEX31 | gDNA | 2 |
| Bzz1-2 | YPL179W | PPQ1 | gDNA | 1 |
| Bzz1-2 | YBL027W | RPL19B | gDNA | 6 |
| Bzz1-2 | YCL031C | RRP7 | gDNA | 3 |
| Bzz1-2 | YHR172W | SPC97 | gDNA | 9 |
| Bzz1-2 | YLL005C | SPO75 | gDNA | 4 |
| Bzz1-2 | YPL032C | SVL3 | gDNA | 1 |
| Bzz1-2 | YBR069C | TAT1 | gDNA | 1 |
| Bzz1-2 | YMR313C | TGL3 | gDNA | 9 |
| Bzz1-2 | YIL144W | TID3 | gDNA | 1 |
| Bzz1-2 | YIL156W | UBP7 | gDNA | 16 |
| Bzz1-2 | YLR024C | UBR2 | gDNA | 2 |
| Bzz1-2 | YHL035C | VMR1 | gDNA | 2 |
| Bzz1-2 | YAL018C | YAL018C | gDNA | 3 |
| Bzz1-2 | YBR108W | YBR108W | gDNA | 1 |
| Bzz1-2 | YBR109W-A | YBR109W-A | gDNA | 1 |
| Bzz1-2 | YCR022C | YCR022C | gDNA | 1 |
| Bzz1-2 | YDR271C | YDR271C | gDNA | 3 |

# Table S10

| SH3 Domain | AD ORF | AD gene name | Y2H library | Number of hits |
|---|---|---|---|---|
| Bzz1-2 | YJL010C | YJL010C | gDNA | 1 |
| Bzz1-2 | YLR426W | YLR426W | gDNA | 1 |
| Bzz1-2 | YML002W | YML002W | gDNA | 1 |
| Bzz1-2 | YGR281W | YOR1 | gDNA | 1 |
| Bzz1-2 | YPL277C | YPL277C | gDNA | 1 |
| Bzz1-2 | YNL094W | APP1 | ORFeome | 4 |
| Bzz1-2 | YJL020C | BBC1 | ORFeome | 2 |
| Bzz1-2 | YGL190C | CDC55 | ORFeome | 4 |
| Bzz1-2 | YPL049C | DIG1 | ORFeome | 4 |
| Bzz1-2 | YDR480W | DIG2 | ORFeome | 3 |
| Bzz1-2 | YOR355W | GDS1 | ORFeome | 4 |
| Bzz1-2 | YDR277C | MTH1 | ORFeome | 3 |
| Bzz1-2 | YCL028W | RNQ1 | ORFeome | 2 |
| Bzz1-2 | YFR040W | SAP155 | ORFeome | 4 |
| Cdc25 | YPL100W | ATG21 | gDNA | 2 |
| Cdc25 | YER172C | BRR2 | gDNA | 7 |
| Cdc25 | YMR275C | BUL1 | gDNA | 2 |
| Cdc25 | YNL116W | DMA2 | gDNA | 1 |
| Cdc25 | YPR023C | EAF3 | gDNA | 1 |
| Cdc25 | YBR140C | IRA1 | gDNA | 1 |
| Cdc25 | YDR122W | KIN1 | gDNA | 3 |
| Cdc25 | YGL197W | MDS3 | gDNA | 43 |
| Cdc25 | YPL082C | MOT1 | gDNA | 8 |
| Cdc25 | YJR132W | NMD5 | gDNA | 1 |
| Cdc25 | YNL231C | PDR16 | gDNA | 1 |
| Cdc25 | YBL017C | PEP1 | gDNA | 16 |
| Cdc25 | YER132C | PMD1 | gDNA | 22 |
| Cdc25 | YPR108W | RPN7 | gDNA | 2 |
| Cdc25 | YCL031C | RRP7 | gDNA | 1 |
| Cdc25 | YER115C | SPR6 | gDNA | 1 |
| Cdc25 | YIL173W | VTH1 | gDNA | 13 |
| Cdc25 | YBR053C | YBR053C | gDNA | 1 |
| Cdc25 | YMR084W | YMR084W | gDNA | 1 |
| Cdc25 | YGR037C | ACB1 | ORFeome | 2 |
| Cdc25 | YPL049C | DIG1 | ORFeome | 4 |
| Cdc25 | YDR480W | DIG2 | ORFeome | 4 |
| Cdc25 | YER052C | HOM3 | ORFeome | 3 |
| Cdc25 | YPL038W | MET31 | ORFeome | 4 |
| Cdc25 | YLR019W | PSR2 | ORFeome | 4 |
| Cdc25 | YHR084W | STE12 | ORFeome | 4 |
| Cdc25 | YNL229C | URE2 | ORFeome | 4 |
| Cdc25 | YOR013W | YOR013W | ORFeome | 3 |
| Cyk3 | YNR016C | ACC1 | gDNA | 1 |
| Cyk3 | YJL155C | FBP26 | gDNA | 2 |
| Cyk3 | YJR138W | IML1 | gDNA | 18 |
| Cyk3 | YNL078W | NIS1 | gDNA | 32 |
| Cyk3 | YNL152W | YNL152W | gDNA | 22 |
| Cyk3 | YOL070C | YOL070C | gDNA | 9 |
| Cyk3 | YPL158C | YPL158C | gDNA | 14 |
| Cyk3 | YGR037C | ACB1 | ORFeome | 4 |
| Cyk3 | YDR211W | GCD6 | ORFeome | 2 |

# Table S10

| SH3 Domain | AD ORF | AD gene name | Y2H library | Number of hits |
|---|---|---|---|---|
| Cyk3 | YPL038W | MET31 | ORFeome | 4 |
| Cyk3 | YER081W | SER3 | ORFeome | 4 |
| Fus1 | YIL159W | BNR1 | gDNA | 90 |
| Fus1 | YPR023C | EAF3 | gDNA | 2 |
| Fus1 | YAL031C | GIP4 | gDNA | 45 |
| Fus1 | YPR008W | HAA1 | gDNA | 10 |
| Fus1 | YER149C | PEA2 | gDNA | 4 |
| Fus1 | YER118C | SHO1 | gDNA | 19 |
| Fus1 | YMR140W | SIP5 | gDNA | 4 |
| Fus1 | YKR101W | SIR1 | gDNA | 4 |
| Fus1 | YKL105C | YKL105C | gDNA | 1 |
| Fus1 | YMR103C | YMR103C | gDNA | 3 |
| Fus1 | YOL046C | YOL046C | gDNA | 1 |
| Fus1 | YLR291C | GCD7 | ORFeome | 4 |
| Fus1 | YPR008W | HAA1 | ORFeome | 4 |
| Fus1 | YPL038W | MET31 | ORFeome | 4 |
| Fus1 | YER149C | PEA2 | ORFeome | 4 |
| Fus1 | YNL267W | PIK1 | ORFeome | 2 |
| Hof1 | YJL084C | ALY2 | gDNA | 10 |
| Hof1 | YNL094W | APP1 | gDNA | 2 |
| Hof1 | YJL020C | BBC1 | gDNA | 9 |
| Hof1 | YPL115C | BEM3 | gDNA | 3 |
| Hof1 | YIL159W | BNR1 | gDNA | 54 |
| Hof1 | YPL084W | BRO1 | gDNA | 3 |
| Hof1 | YJR092W | BUD4 | gDNA | 4 |
| Hof1 | YDL117W | CYK3 | gDNA | 10 |
| Hof1 | YMR162C | DNF3 | gDNA | 3 |
| Hof1 | YOR264W | DSE3 | gDNA | 1 |
| Hof1 | YER032W | FIR1 | gDNA | 6 |
| Hof1 | YNL199C | GCR2 | gDNA | 2 |
| Hof1 | YKL101W | HSL1 | gDNA | 1 |
| Hof1 | YOR181W | LAS17 | gDNA | 4 |
| Hof1 | YGL099W | LSG1 | gDNA | 7 |
| Hof1 | YGL201C | MCM6 | gDNA | 3 |
| Hof1 | YPL140C | MKK2 | gDNA | 1 |
| Hof1 | YMR109W | MYO5 | gDNA | 3 |
| Hof1 | YNL078W | NIS1 | gDNA | 6 |
| Hof1 | YGR240C | PFK1 | gDNA | 1 |
| Hof1 | YHL027W | RIM101 | gDNA | 2 |
| Hof1 | YOR191W | RIS1 | gDNA | 1 |
| Hof1 | YBL027W | RPL19B | gDNA | 1 |
| Hof1 | YER047C | SAP1 | gDNA | 1 |
| Hof1 | YDR178W | SDH4 | gDNA | 4 |
| Hof1 | YDR104C | SPO71 | gDNA | 1 |
| Hof1 | YCL008C | STP22 | gDNA | 7 |
| Hof1 | YER144C | UBP5 | gDNA | 5 |
| Hof1 | YIL156W | UBP7 | gDNA | 4 |
| Hof1 | YAL069W | YAL069W | gDNA | 2 |
| Hof1 | YGL060W | YBP2 | gDNA | 9 |
| Hof1 | YBR108W | YBR108W | gDNA | 2 |
| Hof1 | YFL063W | YFL063W | gDNA | 2 |

# Table S10

| SH3 Domain | AD ORF | AD gene name | Y2H library | Number of hits |
|---|---|---|---|---|
| Hof1 | YJR162C | YJR162C | gDNA | 1 |
| Hof1 | YNL152W | YNL152W | gDNA | 10 |
| Hof1 | YOL036W | YOL036W | gDNA | 1 |
| Hof1 | YOL070C | YOL070C | gDNA | 5 |
| Hof1 | YPL158C | YPL158C | gDNA | 1 |
| Hse1 | YNR016C | ACC1 | gDNA | 1 |
| Hse1 | YNL094W | APP1 | gDNA | 3 |
| Hse1 | YML099C | ARG81 | gDNA | 5 |
| Hse1 | YKL213C | DOA1 | gDNA | 18 |
| Hse1 | YKR009C | FOX2 | gDNA | 2 |
| Hse1 | YOR181W | LAS17 | gDNA | 1 |
| Hse1 | YPL144W | POC4 | gDNA | 1 |
| Hse1 | YCL031C | RRP7 | gDNA | 2 |
| Hse1 | YHL034C | SBP1 | gDNA | 27 |
| Hse1 | YOR076C | SKI7 | gDNA | 3 |
| Hse1 | YBL007C | SLA1 | gDNA | 1 |
| Hse1 | YCL008C | STP22 | gDNA | 14 |
| Hse1 | YIL144W | TID3 | gDNA | 2 |
| Hse1 | YBL067C | UBP13 | gDNA | 2 |
| Hse1 | YIL156W | UBP7 | gDNA | 32 |
| Hse1 | YBR108W | YBR108W | gDNA | 1 |
| Hse1 | YMR084W | YMR084W | gDNA | 4 |
| Hse1 | YOR227W | YOR227W | gDNA | 1 |
| Hse1 | YMR089C | YTA12 | gDNA | 1 |
| Hse1 | YGR037C | ACB1 | ORFeome | 4 |
| Hse1 | YLR144C | ACF2 | ORFeome | 2 |
| Hse1 | YCR048W | ARE1 | ORFeome | 2 |
| Hse1 | YBR112C | CYC8 | ORFeome | 4 |
| Hse1 | YOR355W | GDS1 | ORFeome | 4 |
| Hse1 | YPL038W | MET31 | ORFeome | 4 |
| Hse1 | YDR277C | MTH1 | ORFeome | 3 |
| Hse1 | YFR034C | PHO4 | ORFeome | 3 |
| Hse1 | YNR052C | POP2 | ORFeome | 1 |
| Hse1 | YIL156W | UBP7 | ORFeome | 4 |
| Lsb1 | YGR037C | ACB1 | ORFeome | 3 |
| Lsb1 | YLR144C | ACF2 | ORFeome | 4 |
| Lsb1 | YPR081C | GRS2 | ORFeome | 4 |
| Lsb1 | YOR197W | MCA1 | ORFeome | 4 |
| Lsb1 | YPL038W | MET31 | ORFeome | 4 |
| Lsb1 | YNL293W | MSB3 | ORFeome | 4 |
| Lsb3 | YCR088W | ABP1 | gDNA | 3 |
| Lsb3 | YJR083C | ACF4 | gDNA | 9 |
| Lsb3 | YNL094W | APP1 | gDNA | 7 |
| Lsb3 | YNL065W | AQR1 | gDNA | 1 |
| Lsb3 | YBR068C | BAP2 | gDNA | 1 |
| Lsb3 | YJL194W | CDC6 | gDNA | 3 |
| Lsb3 | YGR218W | CRM1 | gDNA | 1 |
| Lsb3 | YGL238W | CSE1 | gDNA | 1 |
| Lsb3 | YGR227W | DIE2 | gDNA | 1 |
| Lsb3 | YMR162C | DNF3 | gDNA | 1 |
| Lsb3 | YJL201W | ECM25 | gDNA | 8 |

# Table S10

| SH3 Domain | AD ORF | AD gene name | Y2H library | Number of hits |
|---|---|---|---|---|
| Lsb3 | YDR414C | ERD1 | gDNA | 1 |
| Lsb3 | YMR232W | FUS2 | gDNA | 1 |
| Lsb3 | YGL195W | GCN1 | gDNA | 1 |
| Lsb3 | YMR192W | GYL1 | gDNA | 3 |
| Lsb3 | YPL249C | GYP5 | gDNA | 12 |
| Lsb3 | YGR268C | HUA1 | gDNA | 1 |
| Lsb3 | YOR109W | INP53 | gDNA | 1 |
| Lsb3 | YDL146W | LDB17 | gDNA | 7 |
| Lsb3 | YGL201C | MCM6 | gDNA | 1 |
| Lsb3 | YPL038W | MET31 | gDNA | 1 |
| Lsb3 | YMR109W | MYO5 | gDNA | 2 |
| Lsb3 | YGR240C | PFK1 | gDNA | 1 |
| Lsb3 | YGL144C | ROG1 | gDNA | 1 |
| Lsb3 | YCL031C | RRP7 | gDNA | 1 |
| Lsb3 | YPR055W | SEC8 | gDNA | 3 |
| Lsb3 | YBL007C | SLA1 | gDNA | 4 |
| Lsb3 | YOR247W | SRL1 | gDNA | 5 |
| Lsb3 | YNR031C | SSK2 | gDNA | 1 |
| Lsb3 | YHR178W | STB5 | gDNA | 1 |
| Lsb3 | YCL008C | STP22 | gDNA | 7 |
| Lsb3 | YDR310C | SUM1 | gDNA | 1 |
| Lsb3 | YBR069C | TAT1 | gDNA | 1 |
| Lsb3 | YIL156W | UBP7 | gDNA | 8 |
| Lsb3 | YDR027C | VPS54 | gDNA | 1 |
| Lsb3 | YGL060W | YBP2 | gDNA | 4 |
| Lsb3 | YCR018C-A | YCR018C-A | gDNA | 1 |
| Lsb3 | YDL012C | YDL012C | gDNA | 1 |
| Lsb3 | YDL027C | YDL027C | gDNA | 2 |
| Lsb3 | YDR271C | YDR271C | gDNA | 1 |
| Lsb3 | YNL176C | YNL176C | gDNA | 1 |
| Lsb3 | YOL107W | YOL107W | gDNA | 2 |
| Lsb3 | YPR097W | YPR097W | gDNA | 2 |
| Lsb3 | YDR545W | YRF1-1 | gDNA | 1 |
| Lsb3 | YLR144C | ACF2 | ORFeome | 4 |
| Lsb3 | YNL094W | APP1 | ORFeome | 4 |
| Lsb3 | YFR021W | ATG18 | ORFeome | 4 |
| Lsb3 | YPR171W | BSP1 | ORFeome | 4 |
| Lsb3 | YOR042W | CUE5 | ORFeome | 4 |
| Lsb3 | YLR206W | ENT2 | ORFeome | 4 |
| Lsb3 | YDR261C | EXG2 | ORFeome | 4 |
| Lsb3 | YPR081C | GRS2 | ORFeome | 4 |
| Lsb3 | YGL181W | GTS1 | ORFeome | 4 |
| Lsb3 | YGR268C | HUA1 | ORFeome | 4 |
| Lsb3 | YOR181W | LAS17 | ORFeome | 4 |
| Lsb3 | YHL004W | MRP4 | ORFeome | 1 |
| Lsb3 | YFR040W | SAP155 | ORFeome | 4 |
| Lsb3 | YCR030C | SYP1 | ORFeome | 4 |
| Lsb3 | YAL016W | TPD3 | ORFeome | 4 |
| Lsb3 | YDR100W | TVP15 | ORFeome | 4 |
| Lsb3 | YFL010C | WWM1 | ORFeome | 4 |
| Lsb3 | YGR241C | YAP1802 | ORFeome | 4 |

# Table S10

| SH3 Domain | AD ORF | AD gene name | Y2H library | Number of hits |
|---|---|---|---|---|
| Lsb3 | YDL072C | YET3 | ORFeome | 3 |
| Lsb4 | YCR088W | ABP1 | gDNA | 22 |
| Lsb4 | YLR144C | ACF2 | gDNA | 7 |
| Lsb4 | YJR083C | ACF4 | gDNA | 4 |
| Lsb4 | YNL094W | APP1 | gDNA | 5 |
| Lsb4 | YPR171W | BSP1 | gDNA | 1 |
| Lsb4 | YDR270W | CCC2 | gDNA | 1 |
| Lsb4 | YJL194W | CDC6 | gDNA | 4 |
| Lsb4 | YGR218W | CRM1 | gDNA | 1 |
| Lsb4 | YOR042W | CUE5 | gDNA | 1 |
| Lsb4 | YGR227W | DIE2 | gDNA | 1 |
| Lsb4 | YJL201W | ECM25 | gDNA | 3 |
| Lsb4 | YNL084C | END3 | gDNA | 1 |
| Lsb4 | YDL161W | ENT1 | gDNA | 2 |
| Lsb4 | YMR192W | GYL1 | gDNA | 3 |
| Lsb4 | YPL249C | GYP5 | gDNA | 22 |
| Lsb4 | YKL109W | HAP4 | gDNA | 1 |
| Lsb4 | YOR284W | HUA2 | gDNA | 1 |
| Lsb4 | YOR109W | INP53 | gDNA | 2 |
| Lsb4 | YOR181W | LAS17 | gDNA | 3 |
| Lsb4 | YDL146W | LDB17 | gDNA | 3 |
| Lsb4 | YCL034W | LSB5 | gDNA | 1 |
| Lsb4 | YKL129C | MYO3 | gDNA | 1 |
| Lsb4 | YMR109W | MYO5 | gDNA | 2 |
| Lsb4 | YPR052C | NHP6A | gDNA | 1 |
| Lsb4 | YPR055W | SEC8 | gDNA | 8 |
| Lsb4 | YBL007C | SLA1 | gDNA | 8 |
| Lsb4 | YLL005C | SPO75 | gDNA | 2 |
| Lsb4 | YOR247W | SRL1 | gDNA | 9 |
| Lsb4 | YCL008C | STP22 | gDNA | 1 |
| Lsb4 | YMR313C | TGL3 | gDNA | 1 |
| Lsb4 | YIL156W | UBP7 | gDNA | 11 |
| Lsb4 | YGL060W | YBP2 | gDNA | 11 |
| Lsb4 | YBR108W | YBR108W | gDNA | 1 |
| Lsb4 | YBR239C | YBR239C | gDNA | 1 |
| Lsb4 | YLR426W | YLR426W | gDNA | 1 |
| Lsb4 | YGR037C | ACB1 | ORFeome | 3 |
| Lsb4 | YLR144C | ACF2 | ORFeome | 4 |
| Lsb4 | YNR007C | ATG3 | ORFeome | 3 |
| Lsb4 | YPR171W | BSP1 | ORFeome | 4 |
| Lsb4 | YGL190C | CDC55 | ORFeome | 4 |
| Lsb4 | YOR042W | CUE5 | ORFeome | 4 |
| Lsb4 | YDR480W | DIG2 | ORFeome | 2 |
| Lsb4 | YNL084C | END3 | ORFeome | 4 |
| Lsb4 | YLR206W | ENT2 | ORFeome | 4 |
| Lsb4 | YDR261C | EXG2 | ORFeome | 3 |
| Lsb4 | YPR081C | GRS2 | ORFeome | 4 |
| Lsb4 | YGL181W | GTS1 | ORFeome | 4 |
| Lsb4 | YGR268C | HUA1 | ORFeome | 4 |
| Lsb4 | YPL038W | MET31 | ORFeome | 4 |
| Lsb4 | YFR040W | SAP155 | ORFeome | 4 |

# Table S10

| SH3 Domain | AD ORF | AD gene name | Y2H library | Number of hits |
|---|---|---|---|---|
| Lsb4 | YPR055W | SEC8 | ORFeome | 1 |
| Lsb4 | YCR030C | SYP1 | ORFeome | 3 |
| Lsb4 | YDR100W | TVP15 | ORFeome | 4 |
| Lsb4 | YFL010C | WWM1 | ORFeome | 3 |
| Lsb4 | YGR241C | YAP1802 | ORFeome | 4 |
| Myo3 | YJL020C | BBC1 | gDNA | 27 |
| Myo3 | YJL095W | BCK1 | gDNA | 4 |
| Myo3 | YNL084C | END3 | gDNA | 2 |
| Myo3 | YMR109W | MYO5 | gDNA | 1 |
| Myo3 | YDL019C | OSH2 | gDNA | 39 |
| Myo3 | YOR247W | SRL1 | gDNA | 23 |
| Myo3 | YIL156W | UBP7 | gDNA | 24 |
| Myo3 | YLR337C | VRP1 | gDNA | 22 |
| Myo3 | YJL020C | BBC1 | ORFeome | 2 |
| Myo3 | YGL190C | CDC55 | ORFeome | 3 |
| Myo3 | YPL038W | MET31 | ORFeome | 6 |
| Myo3 | YDR277C | MTH1 | ORFeome | 4 |
| Myo3 | YER047C | SAP1 | ORFeome | 6 |
| Myo3 | YOR184W | SER1 | ORFeome | 1 |
| Myo3 | YNL025C | SSN8 | ORFeome | 2 |
| Myo3 | YOR047C | STD1 | ORFeome | 2 |
| Myo3 | YIL156W | UBP7 | ORFeome | 5 |
| Myo3 | YDR010C | YDR010C | ORFeome | 5 |
| Myo5 | YNR016C | ACC1 | gDNA | 1 |
| Myo5 | YJL020C | BBC1 | gDNA | 6 |
| Myo5 | YJL095W | BCK1 | gDNA | 4 |
| Myo5 | YIL159W | BNR1 | gDNA | 13 |
| Myo5 | YEL015W | EDC3 | gDNA | 2 |
| Myo5 | YGR223C | HSV2 | gDNA | 1 |
| Myo5 | YOR181W | LAS17 | gDNA | 1 |
| Myo5 | YGL201C | MCM6 | gDNA | 1 |
| Myo5 | YNR059W | MNT4 | gDNA | 1 |
| Myo5 | YMR109W | MYO5 | gDNA | 3 |
| Myo5 | YKR082W | NUP133 | gDNA | 1 |
| Myo5 | YDL019C | OSH2 | gDNA | 26 |
| Myo5 | YDR466W | PKH3 | gDNA | 2 |
| Myo5 | YER047C | SAP1 | gDNA | 2 |
| Myo5 | YDL139C | SCM3 | gDNA | 1 |
| Myo5 | YOR247W | SRL1 | gDNA | 16 |
| Myo5 | YIL156W | UBP7 | gDNA | 28 |
| Myo5 | YLR337C | VRP1 | gDNA | 16 |
| Myo5 | YGR210C | YGR210C | gDNA | 1 |
| Myo5 | YIL092W | YIL092W | gDNA | 1 |
| Myo5 | YOR227W | YOR227W | gDNA | 3 |
| Myo5 | YML109W | ZDS2 | gDNA | 1 |
| Myo5 | YGR037C | ACB1 | ORFeome | 1 |
| Myo5 | YNR007C | ATG3 | ORFeome | 2 |
| Myo5 | YJL020C | BBC1 | ORFeome | 3 |
| Myo5 | YGL190C | CDC55 | ORFeome | 4 |
| Myo5 | YDR024W | FYV1 | ORFeome | 1 |
| Myo5 | YPR159W | KRE6 | ORFeome | 1 |

# Table S10

| SH3 Domain | AD ORF | AD gene name | Y2H library | Number of hits |
|---|---|---|---|---|
| Myo5 | YNL307C | MCK1 | ORFeome | 1 |
| Myo5 | YPL038W | MET31 | ORFeome | 6 |
| Myo5 | YDR277C | MTH1 | ORFeome | 6 |
| Myo5 | YNL206C | RTT106 | ORFeome | 2 |
| Myo5 | YER047C | SAP1 | ORFeome | 6 |
| Myo5 | YNL025C | SSN8 | ORFeome | 2 |
| Myo5 | YOR047C | STD1 | ORFeome | 2 |
| Myo5 | YIL156W | UBP7 | ORFeome | 2 |
| Myo5 | YLR337C | VRP1 | ORFeome | 2 |
| Myo5 | YDR010C | YDR010C | ORFeome | 6 |
| Nbp2 | YNL094W | APP1 | gDNA | 8 |
| Nbp2 | YNL298W | CLA4 | gDNA | 18 |
| Nbp2 | YMR299C | DYN3 | gDNA | 1 |
| Nbp2 | YDL028C | MPS1 | gDNA | 25 |
| Nbp2 | YJL128C | PBS2 | gDNA | 5 |
| Nbp2 | YCL031C | RRP7 | gDNA | 1 |
| Nbp2 | YOL113W | SKM1 | gDNA | 7 |
| Nbp2 | YHL007C | STE20 | gDNA | 1 |
| Nbp2 | YOL028C | YAP7 | gDNA | 2 |
| Nbp2 | YOL070C | YOL070C | gDNA | 1 |
| Nbp2 | YML109W | ZDS2 | gDNA | 3 |
| Nbp2 | YGL071W | AFT1 | ORFeome | 2 |
| Nbp2 | YGL190C | CDC55 | ORFeome | 6 |
| Nbp2 | YER086W | ILV1 | ORFeome | 6 |
| Nbp2 | YPL038W | MET31 | ORFeome | 2 |
| Nbp2 | YGL035C | MIG1 | ORFeome | 6 |
| Nbp2 | YDR277C | MTH1 | ORFeome | 6 |
| Nbp2 | YAL016W | TPD3 | ORFeome | 4 |
| Pex13 | YNR016C | ACC1 | gDNA | 1 |
| Pex13 | YNL094W | APP1 | gDNA | 5 |
| Pex13 | YJL020C | BBC1 | gDNA | 8 |
| Pex13 | YIL159W | BNR1 | gDNA | 25 |
| Pex13 | YPL084W | BRO1 | gDNA | 28 |
| Pex13 | YER086W | ILV1 | gDNA | 1 |
| Pex13 | YLR096W | KIN2 | gDNA | 1 |
| Pex13 | YOR181W | LAS17 | gDNA | 4 |
| Pex13 | YGL197W | MDS3 | gDNA | 5 |
| Pex13 | YGR239C | PEX21 | gDNA | 4 |
| Pex13 | YDR244W | PEX5 | gDNA | 3 |
| Pex13 | YIL095W | PRK1 | gDNA | 2 |
| Pex13 | YCL031C | RRP7 | gDNA | 3 |
| Pex13 | YER047C | SAP1 | gDNA | 1 |
| Pex13 | YGR279C | SCW4 | gDNA | 1 |
| Pex13 | YER008C | SEC3 | gDNA | 4 |
| Pex13 | YMR066W | SOV1 | gDNA | 5 |
| Pex13 | YNL138W | SRV2 | gDNA | 1 |
| Pex13 | YCL008C | STP22 | gDNA | 2 |
| Pex13 | YIL144W | TID3 | gDNA | 2 |
| Pex13 | YIL156W | UBP7 | gDNA | 6 |
| Pex13 | YGL060W | YBP2 | gDNA | 6 |
| Pex13 | YLL032C | YLL032C | gDNA | 1 |

# Table S10

| SH3 Domain | AD ORF | AD gene name | Y2H library | Number of hits |
|---|---|---|---|---|
| Pex13 | YNL152W | YNL152W | gDNA | 16 |
| Pex13 | YGR037C | ACB1 | ORFeome | 4 |
| Pex13 | YML035C | AMD1 | ORFeome | 4 |
| Pex13 | YFR021W | ATG18 | ORFeome | 4 |
| Pex13 | YKR027W | BCH2 | ORFeome | 2 |
| Pex13 | YGL190C | CDC55 | ORFeome | 4 |
| Pex13 | YPL049C | DIG1 | ORFeome | 4 |
| Pex13 | YDR480W | DIG2 | ORFeome | 4 |
| Pex13 | YOR355W | GDS1 | ORFeome | 4 |
| Pex13 | YGL237C | HAP2 | ORFeome | 2 |
| Pex13 | YDR277C | MTH1 | ORFeome | 4 |
| Pex13 | YGL153W | PEX14 | ORFeome | 4 |
| Pex13 | YNL206C | RTT106 | ORFeome | 2 |
| Pex13 | YFR040W | SAP155 | ORFeome | 4 |
| Pex13 | YKL108W | SLD2 | ORFeome | 4 |
| Pex13 | YDR477W | SNF1 | ORFeome | 4 |
| Pex13 | YNL025C | SSN8 | ORFeome | 3 |
| Pex13 | YML052W | SUR7 | ORFeome | 3 |
| Pex13 | YAL016W | TPD3 | ORFeome | 4 |
| Pin3 | YLR144C | ACF2 | gDNA | 7 |
| Pin3 | YNL094W | APP1 | gDNA | 8 |
| Pin3 | YPR171W | BSP1 | gDNA | 1 |
| Pin3 | YMR162C | DNF3 | gDNA | 2 |
| Pin3 | YDR096W | GIS1 | gDNA | 1 |
| Pin3 | YMR192W | GYL1 | gDNA | 1 |
| Pin3 | YPL249C | GYP5 | gDNA | 10 |
| Pin3 | YOR181W | LAS17 | gDNA | 7 |
| Pin3 | YGL201C | MCM6 | gDNA | 1 |
| Pin3 | YDR505C | PSP1 | gDNA | 2 |
| Pin3 | YOR191W | RIS1 | gDNA | 2 |
| Pin3 | YCL031C | RRP7 | gDNA | 4 |
| Pin3 | YDL139C | SCM3 | gDNA | 1 |
| Pin3 | YGR002C | SWC4 | gDNA | 1 |
| Pin3 | YPL258C | THI21 | gDNA | 4 |
| Pin3 | YIL156W | UBP7 | gDNA | 5 |
| Pin3 | YGR094W | VAS1 | gDNA | 2 |
| Pin3 | YJL012C | VTC4 | gDNA | 2 |
| Pin3 | YAL063C-A | YAL063C-A | gDNA | 1 |
| Pin3 | YBR108W | YBR108W | gDNA | 11 |
| Pin3 | YDL159W-A | YDL159W-A | gDNA | 2 |
| Pin3 | YDR154C | YDR154C | gDNA | 3 |
| Pin3 | YIL108W | YIL108W | gDNA | 7 |
| Pin3 | YJL056C | ZAP1 | gDNA | 2 |
| Pin3 | YLR144C | ACF2 | ORFeome | 4 |
| Pin3 | YGL190C | CDC55 | ORFeome | 4 |
| Pin3 | YPR081C | GRS2 | ORFeome | 4 |
| Pin3 | YOR197W | MCA1 | ORFeome | 4 |
| Pin3 | YPL038W | MET31 | ORFeome | 4 |
| Rvs167 | YLR144C | ACF2 | gDNA | 8 |
| Rvs167 | YJR083C | ACF4 | gDNA | 10 |
| Rvs167 | YNL094W | APP1 | gDNA | 3 |

# Table S10

| SH3 Domain | AD ORF | AD gene name | Y2H library | Number of hits |
|---|---|---|---|---|
| Rvs167 | YPR171W | BSP1 | gDNA | 3 |
| Rvs167 | YJL194W | CDC6 | gDNA | 2 |
| Rvs167 | YMR162C | DNF3 | gDNA | 2 |
| Rvs167 | YMR287C | DSS1 | gDNA | 4 |
| Rvs167 | YMR192W | GYL1 | gDNA | 9 |
| Rvs167 | YPL249C | GYP5 | gDNA | 18 |
| Rvs167 | YFR013W | IOC3 | gDNA | 1 |
| Rvs167 | YOR181W | LAS17 | gDNA | 6 |
| Rvs167 | YGL201C | MCM6 | gDNA | 1 |
| Rvs167 | YMR109W | MYO5 | gDNA | 3 |
| Rvs167 | YGR159C | NSR1 | gDNA | 3 |
| Rvs167 | YGR058W | PEF1 | gDNA | 2 |
| Rvs167 | YGR240C | PFK1 | gDNA | 1 |
| Rvs167 | YBL027W | RPL19B | gDNA | 1 |
| Rvs167 | YHR065C | RRP3 | gDNA | 2 |
| Rvs167 | YCL031C | RRP7 | gDNA | 7 |
| Rvs167 | YOR329C | SCD5 | gDNA | 1 |
| Rvs167 | YDL139C | SCM3 | gDNA | 1 |
| Rvs167 | YPR055W | SEC8 | gDNA | 15 |
| Rvs167 | YBR150C | TBS1 | gDNA | 3 |
| Rvs167 | YIL156W | UBP7 | gDNA | 9 |
| Rvs167 | YLR337C | VRP1 | gDNA | 1 |
| Rvs167 | YGL060W | YBP2 | gDNA | 11 |
| Rvs167 | YBR108W | YBR108W | gDNA | 7 |
| Rvs167 | YBR239C | YBR239C | gDNA | 2 |
| Rvs167 | YER172C-A | YER172C-A | gDNA | 1 |
| Rvs167 | YGL226W | YGL226W | gDNA | 1 |
| Rvs167 | YJL195C | YJL195C | gDNA | 1 |
| Rvs167 | YJR115W | YJR115W | gDNA | 1 |
| Rvs167 | YMR003W | YMR003W | gDNA | 1 |
| Rvs167 | YNL176C | YNL176C | gDNA | 1 |
| Rvs167 | YML109W | ZDS2 | gDNA | 1 |
| Rvs167 | YLR144C | ACF2 | ORFeome | 4 |
| Rvs167 | YGL190C | CDC55 | ORFeome | 4 |
| Rvs167 | YPR081C | GRS2 | ORFeome | 4 |
| Rvs167 | YNL206C | RTT106 | ORFeome | 4 |
| Rvs167 | YOR047C | STD1 | ORFeome | 4 |
| Sho1 | YNL331C | AAD14 | gDNA | 19 |
| Sho1 | YKR027W | BCH2 | gDNA | 2 |
| Sho1 | YDL117W | CYK3 | gDNA | 4 |
| Sho1 | YNL112W | DBP2 | gDNA | 1 |
| Sho1 | YHR143W | DSE2 | gDNA | 1 |
| Sho1 | YCL027W | FUS1 | gDNA | 10 |
| Sho1 | YLR096W | KIN2 | gDNA | 14 |
| Sho1 | YOR181W | LAS17 | gDNA | 19 |
| Sho1 | YGL201C | MCM6 | gDNA | 3 |
| Sho1 | YJL128C | PBS2 | gDNA | 3 |
| Sho1 | YOR127W | RGA1 | gDNA | 13 |
| Sho1 | YCL031C | RRP7 | gDNA | 1 |
| Sho1 | YBL007C | SLA1 | gDNA | 1 |
| Sho1 | YHL007C | STE20 | gDNA | 1 |

# Table S10

| SH3 Domain | AD ORF | AD gene name | Y2H library | Number of hits |
|---|---|---|---|---|
| Sho1 | YIL144W | TID3 | gDNA | 1 |
| Sho1 | YLR425W | TUS1 | gDNA | 5 |
| Sho1 | YIL156W | UBP7 | gDNA | 1 |
| Sho1 | YER082C | UTP7 | gDNA | 1 |
| Sho1 | YDL073W | YDL073W | gDNA | 8 |
| Sho1 | YJR115W | YJR115W | gDNA | 1 |
| Sho1 | YNL152W | YNL152W | gDNA | 1 |
| Sla1-1/2 | YBL037W | APL3 | gDNA | 1 |
| Sla1-1/2 | YNL094W | APP1 | gDNA | 1 |
| Sla1-1/2 | YPR171W | BSP1 | gDNA | 2 |
| Sla1-1/2 | YNL116W | DMA2 | gDNA | 1 |
| Sla1-1/2 | YJL201W | ECM25 | gDNA | 9 |
| Sla1-1/2 | YLR206W | ENT2 | gDNA | 1 |
| Sla1-1/2 | YNL106C | INP52 | gDNA | 1 |
| Sla1-1/2 | YFR013W | IOC3 | gDNA | 1 |
| Sla1-1/2 | YOR181W | LAS17 | gDNA | 8 |
| Sla1-1/2 | YDL146W | LDB17 | gDNA | 23 |
| Sla1-1/2 | YGL201C | MCM6 | gDNA | 1 |
| Sla1-1/2 | YOR211C | MGM1 | gDNA | 1 |
| Sla1-1/2 | YMR109W | MYO5 | gDNA | 14 |
| Sla1-1/2 | YBL105C | PKC1 | gDNA | 4 |
| Sla1-1/2 | YDR382W | RPP2B | gDNA | 1 |
| Sla1-1/2 | YCL031C | RRP7 | gDNA | 7 |
| Sla1-1/2 | YOR329C | SCD5 | gDNA | 1 |
| Sla1-1/2 | YCR030C | SYP1 | gDNA | 2 |
| Sla1-1/2 | YLR154W-C | TAR1 | gDNA | 1 |
| Sla1-1/2 | YBR150C | TBS1 | gDNA | 1 |
| Sla1-1/2 | YIL144W | TID3 | gDNA | 2 |
| Sla1-1/2 | YIL156W | UBP7 | gDNA | 5 |
| Sla1-1/2 | YBR108W | YBR108W | gDNA | 10 |
| Sla1-1/2 | YNL176C | YNL176C | gDNA | 3 |
| Sla1-1/2 | YML109W | ZDS2 | gDNA | 1 |
| Sla1-1/2-W108S | YDR423C | CAD1 | gDNA | 5 |
| Sla1-1/2-W108S | YNL161W | CBK1 | gDNA | 1 |
| Sla1-1/2-W108S | YNR041C | COQ2 | gDNA | 1 |
| Sla1-1/2-W108S | YKL054C | DEF1 | gDNA | 3 |
| Sla1-1/2-W108S | YJL201W | ECM25 | gDNA | 1 |
| Sla1-1/2-W108S | YLR088W | GAA1 | gDNA | 1 |
| Sla1-1/2-W108S | YML075C | HMG1 | gDNA | 1 |
| Sla1-1/2-W108S | YLR095C | IOC2 | gDNA | 11 |
| Sla1-1/2-W108S | YOR181W | LAS17 | gDNA | 2 |
| Sla1-1/2-W108S | YLR106C | MDN1 | gDNA | 3 |
| Sla1-1/2-W108S | YKL074C | MUD2 | gDNA | 1 |
| Sla1-1/2-W108S | YKR022C | NTR2 | gDNA | 1 |
| Sla1-1/2-W108S | YDR382W | RPP2B | gDNA | 4 |
| Sla1-1/2-W108S | YCL031C | RRP7 | gDNA | 1 |
| Sla1-1/2-W108S | YDL139C | SCM3 | gDNA | 25 |
| Sla1-1/2-W108S | YPL027W | SMA1 | gDNA | 2 |
| Sla1-1/2-W108S | YAL011W | SWC3 | gDNA | 1 |
| Sla1-1/2-W108S | YPR048W | TAH18 | gDNA | 1 |
| Sla1-1/2-W108S | YPL157W | TGS1 | gDNA | 6 |

# Table S10

| SH3 Domain | AD ORF | AD gene name | Y2H library | Number of hits |
|---|---|---|---|---|
| Sla1-1/2-W108S | YOL028C | YAP7 | gDNA | 2 |
| Sla1-1/2-W108S | YML109W | ZDS2 | gDNA | 5 |
| Sla1-1/2-W108S | YCR100C | | gDNA | 3 |
| Sla1-1/2-W108S | YDL206W | | gDNA | 1 |
| Sla1-1/2-W108S | YDR520C | | gDNA | 7 |
| Sla1-1/2-W108S | YJR115W | | gDNA | 5 |
| Sla1-1/2-W108S | YNL285W | | gDNA | 1 |
| Sla1-1/2-W108S | YDR277C | MTH1 | ORFeome | 1 |
| Sla1-1/2-W108S | YOR047C | STD1 | ORFeome | 1 |
| Sla1-1/2-W108S | YCL008C | STP22 | ORFeome | 4 |
| Sla1-1/2-W108S | YLR363W-A | | ORFeome | 4 |
| Sla1-1/2-W41S | YNL094W | APP1 | gDNA | 6 |
| Sla1-1/2-W41S | YLL018C | DPS1 | gDNA | 1 |
| Sla1-1/2-W41S | YOR388C | FDH1 | gDNA | 2 |
| Sla1-1/2-W41S | YER027C | GAL83 | gDNA | 10 |
| Sla1-1/2-W41S | YLR095C | IOC2 | gDNA | 7 |
| Sla1-1/2-W41S | YOR181W | LAS17 | gDNA | 14 |
| Sla1-1/2-W41S | YMR109W | MYO5 | gDNA | 12 |
| Sla1-1/2-W41S | YDL002C | NHP10 | gDNA | 2 |
| Sla1-1/2-W41S | YLR315W | NKP2 | gDNA | 1 |
| Sla1-1/2-W41S | YGR240C | PFK1 | gDNA | 1 |
| Sla1-1/2-W41S | YCL031C | RRP7 | gDNA | 2 |
| Sla1-1/2-W41S | YDL139C | SCM3 | gDNA | 50 |
| Sla1-1/2-W41S | YIL076W | SEC28 | gDNA | 1 |
| Sla1-1/2-W41S | YOR047C | STD1 | gDNA | 1 |
| Sla1-1/2-W41S | YBR150C | TBS1 | gDNA | 2 |
| Sla1-1/2-W41S | YER151C | UBP3 | gDNA | 2 |
| Sla1-1/2-W41S | YIL173W | VTH1 | gDNA | 1 |
| Sla1-1/2-W41S | YML109W | ZDS2 | gDNA | 2 |
| Sla1-1/2-W41S | YDR520C | | gDNA | 4 |
| Sla1-1/2-W41S | YML116W | ATR1 | ORFeome | 1 |
| Sla1-1/2-W41S | YGL237C | HAP2 | ORFeome | 1 |
| Sla1-1/2-W41S | YGL035C | MIG1 | ORFeome | 2 |
| Sla1-1/2-W41S | YDR277C | MTH1 | ORFeome | 1 |
| Sla1-1/2-W41S | YJL145W | SFH5 | ORFeome | 1 |
| Sla1-1/2-W41S | YOR047C | STD1 | ORFeome | 9 |
| Sla1-3 | YGL238W | CSE1 | gDNA | 2 |
| Sla1-3 | YPR030W | CSR2 | gDNA | 7 |
| Sla1-3 | YGR227W | DIE2 | gDNA | 2 |
| Sla1-3 | YHL016C | DUR3 | gDNA | 4 |
| Sla1-3 | YBL047C | EDE1 | gDNA | 2 |
| Sla1-3 | YOR109W | INP53 | gDNA | 1 |
| Sla1-3 | YER110C | KAP123 | gDNA | 1 |
| Sla1-3 | YOR322C | LDB19 | gDNA | 40 |
| Sla1-3 | YBR136W | MEC1 | gDNA | 6 |
| Sla1-3 | YIR033W | MGA2 | gDNA | 4 |
| Sla1-3 | YKR095W | MLP1 | gDNA | 1 |
| Sla1-3 | YDL028C | MPS1 | gDNA | 11 |
| Sla1-3 | YPL022W | RAD1 | gDNA | 2 |
| Sla1-3 | YPL012W | RRP12 | gDNA | 2 |
| Sla1-3 | YDR388W | RVS167 | gDNA | 1 |

# Table S10

| SH3 Domain | AD ORF | AD gene name | Y2H library | Number of hits |
|---|---|---|---|---|
| Sla1-3 | YLR430W | SEN1 | gDNA | 1 |
| Sla1-3 | YBR250W | SPO23 | gDNA | 2 |
| Sla1-3 | YLL005C | SPO75 | gDNA | 2 |
| Sla1-3 | YBL088C | TEL1 | gDNA | 1 |
| Sla1-3 | YMR313C | TGL3 | gDNA | 8 |
| Sla1-3 | YML097C | VPS9 | gDNA | 1 |
| Sla1-3 | YCL045C | YCL045C | gDNA | 1 |
| Sla1-3 | YDL203C | YDL203C | gDNA | 5 |
| Sla1-3 | YDR271C | YDR271C | gDNA | 2 |
| Sla1-3 | YLR064W | YLR064W | gDNA | 1 |
| Sla1-3 | YMR147W | YMR147W | gDNA | 1 |
| Sla1-3 | YNL144C | YNL144C | gDNA | 3 |
| Sla1-3 | YGL215W | CLG1 | ORFeome | 3 |
| Sla1-3 | YGR268C | HUA1 | ORFeome | 4 |
| Sla1-3 | YNL229C | URE2 | ORFeome | 2 |
| Sla1-3 | YFL010C | WWM1 | ORFeome | 4 |
