# Supplementary material for: Bayesian Modeling of the Yeast SH3 Domain Interactome Predicts Spatiotemporal Dynamics of Endocytosis Proteins
Source: PLoS Biol. 2009 Oct 20;7(10):e1000218. doi: 10.1371/journal.pbio.1000218 (PMC2756588; doi:10.1371/journal.pbio.1000218)
Supplement: Table S11 — Gold-standard yeast SH3 domain interactions. The literature was manually curated for yeast SH3 domain-mediated interactions. The PubMed Identifier (PMID) is shown for the paper in which the interaction was identified. Each row represents a unique SH3 domain-ligand interaction with their associated UniProt identification numbers (SPID). The amino acid (A.A.) range and sequence for the interacting protein (or protein fragment) is shown along with the techniques used to identify it (Method 1, 2, or 3, where applicable). The biological relevance of the SH3-mediated interaction as described in the paper is also reported. The list contains redundant interactions as some SH3 domain mediated interactions were reported in more than one paper. A brief description of the experimental techniques listed is provided as follows: 1) Overlay assay: the SH3 domain protein is run on SDS gel and transferred to nitrocellulose. The membrane is then probed with the interactor (or vice versa); 2) Spot synthesis overlay: peptides are chemically synthesized on array format, and the membrane is probed with GST-SH3 fusion protein; 3) Phage-display/two-hybrid: random nonapeptides are selected with phage display and compared with yeast two-hybrid analysis to map the peptide; 4) Alanine scanning: each amino acid of the binding peptide is substituted with Ala and the effect of the mutation on SH3 binding is analyzed; 5) Mutagenesis analysis: each amino acid of the binding peptide is mutated with any other amino acid and the effects of the mutation on SH3 binding are analyzed; 6) Co-IP: the SH3 domain is immunoprecipitated and the presence of interactors in the complex is revealed by western blot analysis with specific antibodies; and 7) Affinity coprecipitation: the SH3 domain is precipitated with an affinity column (GST column) and the presence of interactors in the complex is revealed in Western blot analysis with specific antibodies. (0.09 MB PDF) [file pbio.1000218.s020.pdf]

Table S11. Gold standard yeast SH3 domain interactions

PMID	SH3 domain protein name	SPID domain protein	Ligand name	SPID ligand	A.A range	Sequence	Method 1	Method 2	Method 3	Biological Relevance
9190214	Rvs167	P39743	Abp1	P15891	444-537	DDDEDEAAQPPLPSRNVASG<br>APVQKEEPEQEEIAPSLPSRN<br>SIPAPKQEEAPEQAPEEEIEEE<br>AEEAAPQLPSRSSAAPPPPPR<br>RATPEKKPKENPWATAEYDYD<br>AAEDNELTFVENDKIINIEFVDD<br>DWWLGELEKDG	overlay assay [(1)]			actin reorganization in response to environmental condition
10388809	Rvs167	P39743	Abp1	P15891	444-537	DDDEDEAAQPPLPSRNVASG<br>APVQKEEPEQEEIAPSLPSRN<br>SIPAPKQEEAPEQAPEEEIEEE<br>AEEAAPQLPSRSSAAPPPPPR<br>RATPEKKPKENPWATAEYDYD<br>AAEDNELTFVENDKIINIEFVDD<br>DWWLGELEKDG	two hybrid			actin reorganization in response to environmental condition
11668184	Abp1	P15891	Ark1	P53974	604-631	DKKTKPTPPPKPSHLKPKPPP<br>KPLLLAG	phage display	pull down	two hybrid	localization of Ark in actin cortical patches
14668868	Abp1	P15891	Ark1	P53974	605-620	KKTKPTPPPKPSHLK	fluorescence titration			localization of Ark in actin cortical patches
14737190	Abp1	P15891	Ark1	P53974	602-622	SKDKKTKPTPPPKP	spot synthesis overlay [(2)]	surface plasmon resonance	CoIP [(6)]	localization of Ark in actin cortical patches
17409071	Abp1	P15891	Ark1	P53974	605-621	KKTKPTPPPKPSHLKPK	isothermal titration calor	pull down		localization of Ark in actin cortical patches
11901111	Myo5	Q04439	Bbc1	P47068	799-1157	PPLPRAPPVPPATFEFDSEPTA<br>THSHTAPSPPPHQNVTASTPS<br>MMSTQQRVPTSVLSGAEKES<br>RTLPPHVPSLTNRPVDSFHES<br>DTTPKVASIRRSTTHDVGE<br>ISNNVKIEFNAQERWWINKSA<br>PPAISNLKLNFLMEIDDHFISKR<br>LHQKWVVRDFYFLFEN<br>YSQLRFSLTFNSTSPEKTVTTL<br>QERFPSPVETQSARILDEYAQ<br>RFNAKVVEKSHSLINSH<br>IGAKNFVSQIVSEFKDEVIQPI<br>GARTFGATILSYKPEEGIEQLM<br>KSLQKIKPGDILVIRK<br>AKFEAHKKIGKNEIINVGMDSA<br>APYSSVVTDYDFTKNKFRVIE<br>NHEGKIIQNSYKLSHMK<br>SGKLKVFRIVARGYVGW*	two hybrid	overlay assay [(1)]	CoIP [(6)]	actin reorganization

Table S11

PMID	SH3 domain protein name	SPID domain protein	Ligand name	SPID ligand	A.A range	Sequence	Method 1	Method 2	Method 3	Biological Relevance
11901111	Myo3	P36006	Bbc1	P47068	799-1157	PPLPRAPPVPPATFEFDSEPTA<br>THSHTAPSPPPHQNVTASTPS<br>MMSTQQRVPTSVLSGAEKES<br>RTLPPHVPSLTNRPVDSFESD<br>TTPKVASIRRSTTHDVGE<br>ISNNVKIEFNAQERWWINKSA<br>PPAISNLKLNFLMEIDDHFISKR<br>LHQKWVVRDFYFLFEN<br>YSQLRFSLTFNSTSPEKTVTTL<br>QERFPSPVETQSARILDEYAQ<br>RFNAKVVEKSHSLINSH<br>IGAKNFVSQIVSEFKDEVIQPI<br>GARTFGATILSYKPEEGIEQLM<br>KSLQKIKPGDILVIRK<br>AKFEAHKKIGKNEIINVGMDSA<br>APYSSVVTDYDFTKNKFRVIE<br>NHEGKIIQNSYKLSHMK<br>SGKLKVFRIVARGYVGW*	two hybrid	overlay assay (1)		actin reorganization
15020407	Fus1	P11710	Bni1	P41832	1 – 1219	MLKNSGSKHSNSKESHSNSS<br>SGIFQNLKRLANSNATNSNTG<br>SPTYASQQQHSPVGNEVSTS<br>PASSSSFRKLNAPSRSTSTEA<br>RPLNKKSTLNTQNLSQYMNG<br>KLSGDVPVSSQHARSHSMQS<br>KYSYSKRNSSQASNKLTRQHT<br>GQSHSASSLLSQGSLTNLSKF<br>TTPDGKIYLEMPSDPYEVEVL<br>FEDIMYKRNIFQSLSEDKQEAL<br>MGYSIEKKWLIVKQDLQNELK<br>KMRANTTSSSTASRTSMASD<br>HHPILTANSSLSSPKSVLMTSA<br>SSPTSTVYSNSLNHSTTLSSV<br>GTSTSKGKKLVSGSLKKQPSL<br>NNIYRGGAENNTSASTLPGDR<br>TNRPPIHYVQRILADKLTSDEM<br>KDLWVTLRTEQLDWVDAFIDH<br>QGHIAMANVLMNSIYKTAPRE<br>NLTKELLEKENSFFKCFRVLS<br>MLSQGLYEFSTHRLMTDTVAE<br>GLFSTKLATRKMATEIFVCMLE<br>KKNKSRFEAVLTSLDKKFRIGQ<br>NLHMIQNFKKMPQYFSHLTLE<br>SHLKIIQAWLFAVEQTLDGRGK<br>MGSLVGASDEFKNGGGENAIL<br>EYCQWTMVFINHLCSCSDNIN<br>QRMLLRTKLENCGILRIMNKIK<br>LLDYDKVIDQIELYDNNKLDDF<br>NVKLEANNKAFNVDLHDPLSL<br>LKNLWDICKGTENEKLLVSLVQ<br>HLFLSSSKLIEENQNSSKLTKQ<br>LKLMDSLVTNVSVASTSDEET<br>NMNMAIQRLYDAMQTDEVAR	two hybrid			Bn1p localization during mating

Table S11

PMID	SH3 domain protein name	SPID domain protein	Ligand name	SPID ligand	A.A range	Sequence	Method 1	Method 2	Method 3	Biological Relevance
15020407	Fus1	P11710	Bnr1	P40450	1 – 758	MDSSPNKKTYRYPRRSLSLHA RDRVSEARKLEELNLNDGLVA AGLQLVGVALEKQGTGSHIYM KQKNFSANDVSSSPMVSEEV NGSEMDFNPKCMPQDASLVE RMFDELLKDGTFFWGAAYKN LQNISLRRKWLLICKIRSSNHW GKKKVTSSTTYSTHLATNELA ENAHFLDGLVRNLSTGGMKLS KALYKLEKFLRKQSFLQLFLKD EIYLTTLIEKTLPLISKELQFVYL RCFKILMNNPLARIRALHSEPL IRWFTELLTDQNSNLKCQLLS MELLLLLLTYVEGSTGCELIWD QLSILFTDWLEWFDKILADDIAI HSSLYLNWNQLKIDYSTTFLLL INSILQGFNNKTALEILNFLKKN NIHNTITFLELAYKDDPNSVVIM EQIKQFKSKESAIFDSMIKTTN DTNSLHPTKDIARIESEPLCLE NCLLLKAKDSPVEAPINEIIQSL WKILDSQKPYSESIKLLKLINSL LFYLIDSFQVSTNPSFDETLES AENVDYVFQDSVNKLLDSLQS DEIARRAVTEIDDLNAKISHLN EKLNLVENHDKDHLIAKLDESE SLISLKTKEIENLKLQLKATKKR LDQITTHQRLYDQPPSLASSN LSIAGSIIKNNSHGNIIFQNLAK KQQQQQKISLPKRSTSLLKSK RVTSLSSYLTDANNENESQNE SEDKSKDSLFQRSTSTINFNIP SMKNITNMQNVSLNSILSELEF SNSLGTQPNYQSSPVLSSVSS	two hybrid			actin nucleation
18280496	Fus1	P11710	Bnr1	P40450	639 - 650	KISLPKRSTSLL	phage display	fluorescence titration	alanine scanning (4)	actin nucleation
9774458	Hof1	Q05080	Bnr1p	P40450	765-806	PQLPPPPPPPPPPLPQSLLT EAEAKPDGVSCIAAPAPPPLP	two hybrid	pull down		actin cytoskeleton reorganization
8666672	Bem1_2	P29366	Boi1	P38041	394-414	PGRAPKPPSYPSPVQPPQSR	CoIP (6)	pull down	two hybrid	bud formation
16802101	Bem1 _2	P29366	Boi1	P38041	371-423	PAEQILDMTEVPNLFADKDIFE SPGRAPKPPSYPSPVQPPQS PSFNNRYT	phage display	ELISA		bud formation
8666671	Bem1_2	P29366	Boi2	P39969	436-464	VSPRRAPKPPSYPSPAQPPKS PLLNNTR	two hybrid	pull down		bud formation
8666672	Bem1_2	P29366	Boi2	P39969	424-487	QVVEEMAGNENLFVSPRRAP KPPSYPSPAQPPKSPLLNNTR TSPSPAQLYSWQSPTLSFSGP KRT	pull down	two hybrid		bud formation
16802101	Bem1 _2	P29366	Boi2	P39969	355-458	PANVDQRASYRGHVRKTSQS LEDLPSQQNFIPTPRNTRNSS ASKHRPKSLVFDSQEANANIA PDVQIPQVVEEMAGNENLFVS PRRAPKPPSYPSPAQPPKSP	phage display	ELISA		bud formation

Table S11

PMID	SH3 domain protein name	SPID domain protein	Ligand name	SPID ligand	A.A range	Sequence	Method 1	Method 2	Method 3	Biological Relevance
10048790	Cdc25	P04821	Cyr1	P08678	1769-1960	SVRMGIHWGCPVPELDLVTQ RMDYLGPMVNKAARVQGVAD GGQIAMSSDFYSEFNKIMKYH ERVVKGKESLKEVYGEEIIGEV LEREIAMLESIGWAFFDFGEH KLKGLETKELVTIAYPKILASRH EFASEDEQSKLINETMLFRLRV IS NRLESIMSALSGGFIELDSRTE GSYIKFNPKVENGIMQSI	HIS-pull down	GST-pull down		activation of adenylyl cyclase activity of Cyr1
18508771	Hse1	P38753	Doa1	P36037	433-445	FILKNTNGISLD	pull down			sorting ub-protein in MVBs
15020407	Sho1	P40073	Fus1	P11710	416-425	SRSKPLPLTP	two hybrid	pull down		prevents Sho1p from signaling during mating
15473003	Rvs167	P39743	Gyp5	Q12344	1 – 446	MSSDKSIEKNTDTIASEVHEG DNHSNNLGSMEEEIKSTPSDQ YEEIAIIPTEPLHSDKELNDKQ QSLGHEAPTNVSREEPIGISG DEDTQITEQNVNEQRQETRE PSSEIDLNEPLDVEKDVTTDV QAPNGLNIEKEYDAVKENEKV YADTKEVVSSPENREVTGKNS GGEKSSSSKFLDDESGTTTAA NANDISISSEVTPERSSENDN NQIHITNEVAAGINLNENKEQK AAIEDGPVTAENLSSETARKVP PIPTQIINEKGDNSSENEVSAIP TTSSPPLPPRQNVATSTSPKL PPRGKQREQPPKTKNAVPPP LEEEMKSEKFRKNFEETKRNS YHHVPLTGSKTAQLESTAEINL IASRYRKTSHHLNKEGEETRE SLQEGQSFLKSTFTSFLENLS EYNEVENVNEEDREMFKIDW SFWTQVVNDYATVASNEPENL EA	two hybrid			vescicle trafficking from ER to Golgi

Table S11

PMID	SH3 domain protein name	SPID domain protein	Ligand name	SPID ligand	A.A range	Sequence	Method 1	Method 2	Method 3	Biological Relevance
15802519	Rvs167	P39743	Gyp5	Q12344	1-441	MSSDKSIEKNTDTIASEVHEGDNHSNNLGSMEEEIKSTPSDQYEEIAIIPTEPLHSDKELNDKQQSLGHEAPTNVSREEPIGISGDEDTQITEQNVNEQRQETREPSSEIDLNEPLDVEKDVTTDVQAPNGLNIEKEYDAVKENEKVYADTKEVVSSPENREVTGKNSGGEKSSSSKFLDDESGTTTAANANDISISSEVTPERSSENDNNQIHITNEVAAGINLNENKEQKAAIEDGPVTAENLSSETARKVPPIPTQIINEKGDNSSENEVSAIPTTSSPPLPPRQNVATSTSPKLPPRGKQREQPPKTKNAVPPPLEEEMKSEKFRKNFEETKRNSYHHVPLTGSKTAQLESTAEINLIASRYRKTSHHLNKEGEETRESLQEGQSFLKSTFTSFLENLSEYNE	overlay assay [1]	CoIP [6]	pull down	vescicle trafficking from ER to Golgi
11743162	Bbc1	P47068	Las17	Q12446	339-366	RLPAPPPPPRRGPAPPPPPHRHVTSNT	phage display/two hybrid [3]	ELISA	CoIP [6]	negative regulation of Las17
11743162	Lsb1	P53281	Las17	Q12446	306-336	PQQNRPLPQLPNRNNRPVPPPPPMRTTTEG	phage display/two hybrid [3]	ELISA	CoIP [6]	actin polymerization
11743162	Lsb1	P53281	Las17	Q12446	339-366	RLPAPPPPPRRGPAPPPPPHRHVTSNT	phage display/two hybrid [3]	ELISA	CoIP [6]	actin polymerization
11743162	Bzz1	P38822	Las17	Q12446	153-190	HGPRGESLIDNQRKRYNYEDVDTIPTTKHKAPPPPPP	phage display/two hybrid [3]	ELISA	CoIP [6]	actin polymerization in early step of endocytosis
11743162	Bzz1	P38822	Las17	Q12446	339-366	RLPAPPPPPRRGPAPPPPPHRHVTSNT	phage display/two hybrid [3]	ELISA	CoIP [6]	actin polymerization in early step of endocytosis
11743162	Pin3	Q06449	Las17	Q12446	339-366	RLPAPPPPPRRGPAPPPPPHRHVTSNT	phage display/two hybrid [3]	ELISA	CoIP [6]	unknown
11743162	Lsb3	P43603	Las17	Q12446	306-336	PQQNRPLPQLPNRNNRPVPPPPPMRTTTEG	phage display/two hybrid [3]	ELISA	CoIP [6]	actin polymerization during endocytosis
11743162	Lsb1	P53281	Las17	Q12446	374-403	LLPQATGRRGPAPPPPPRASRPTPNVTMQ	phage display/two hybrid [3]	ELISA	CoIP [6]	actin polymerization
11743162	Pin3	Q06449	Las17	Q12446	306-336	PQQNRPLPQLPNRNNRPVPPPPPMRTTTEG	phage display/two hybrid [3]	ELISA	CoIP [6]	unknown
11743162	Ysc84	P32793	Las17	Q12446	306-336	PQQNRPLPQLPNRNNRPVPPPPPMRTTTEG	phage display/two hybrid [3]	ELISA	CoIP [6]	activation of Ysc84 actin binding capability
11743162	Ysc84	P32793	Las17	Q12446	339-366	RLPAPPPPPRRGPAPPPPPHRHVTSNT	phage display/two hybrid [3]	ELISA	CoIP [6]	activation of Ysc84 actin binding capability
11743162	Lsb3	P43603	Las17	Q12446	339-366	RLPAPPPPPRRGPAPPPPPHRHVTSNT	phage display/two hybrid [3]	ELISA	CoIP [6]	actin polymerization during endocytosis
12391157	Bzz1	P38822	Las17	Q12446	318-544	NRNNRPVPPPPPMRTTTEGSGVRLPAPPPPPRRGPAPPPPPHRHVTSNTLNSAGGNSLLPQATGRRGPAPPPPPRASRPTPNVTMQQNPQQYNNSNRPFGYQTNSNMSSPPPPPVTTFNTLTPQMTAATGQPAVPLPQNTQAPSQATNVPVAPPPPPASLGQSQIPQSAPSAPIPPTLPSTTSAAPPPPPAFLTQQPQSGGAPAPPPPPQMPA	two hybrid	pull down	CoIP [6]	localization of Bzz1 in actin cortical patches

Table S11

PMID	SH3 domain protein name	SPID domain protein	Ligand name	SPID ligand	A.A range	Sequence	Method 1	Method 2	Method 3	Biological Relevance
17522383	Myo5	Q04439	Pan1	P32521	1468-1480	PPAGIPPPPPLP	two hybrid	pull down	CoIP (6)	late stage endocytosis
17522383	Myo5	Q04439	Pan1	P32521	1232-1480	STGLPSTTMGHNPYFKDASA SSTSTFDARAAEMQRRIQRGL DEDEDDGWSDEDESNNRVAV DNKVEEAKIGHPDHARAPPVT AAPLPSVTPVPPAVPVPQANT SNEKSSPIPIAPIPPSVTQEPP VPLAPPLPAVDGFQEPPIPSAP AIATAVQKSGSSTPALAGGVLP PPPPLPTQQASTSEPIIAHVDN YNGAEKGTGAYGSDSDDDVL SIPESVGTDEEEEGAQPVSTA GIPSIPPAGIPPPPPLP	two hybrid	pull down	CoIP (6)	late stage endocytosis
17522383	Myo3	P36006	Pan1	P32521	1232-1480	STGLPSTTMGHNPYFKDASA SSTSTFDARAAEMQRRIQRGL DEDEDDGWSDEDESNNRVAV DNKVEEAKIGHPDHARAPPVT AAPLPSVTPVPPAVPVPQANT SNEKSSPIPIAPIPPSVTQEPP VPLAPPLPAVDGFQEPPIPSAP AIATAVQKSGSSTPALAGGVLP PPPPLPTQQASTSEPIIAHVDN YNGAEKGTGAYGSDSDDDVL SIPESVGTDEEEEGAQPVSTA GIPSIPPAGIPPPPPLP	two hybrid	pull down	CoIP (6)	late stage endocytosis
17522383	Myo3	P36006	Pan1	P32521	1468-1480	PPAGIPPPPPLP	two hybrid	pull down	CoIP (6)	late stage endocytosis
14668868	Sho1	P40073	PBS2	P08018	92-106	NKPLPPLPVAGSSKV	fluorescence titration	protein array	growth assay	activation of high osmolarity stress response pathway
14685261	Nbp2	Q12163	Pbs2	P08018	180-199	LNPNRRAPRRPLSTQHPTRP	affinity coprecipitation (7)	pull down		negative regulation of high osmolarity response pathway
10087260	Pex13	P80667	Pex14	P53112	86-94	PPTLPHRDW	two hybrid	CoIP (6)		peroxisomal localization of Pex14
12453410	Pex13	P80667	Pex14	P53112	83-96	PPTLPHRDWKDY	NMR titration	X-ray diffraction	CD spectroscopy	protein import in peroxisome
12595255	Pex13	P80667	Pex14	P53112	81-96	YEAMPPTLPHRDWDY	spot synthesis overlay (2)	NMR titration		protein import in peroxisome
11071920	Pex13	P80667	Pex5	P35056	204-219	WTDQFEKLEKEVSENL	two hybrid	pull down		protein import in peroxisome
11101511	Pex13	P80667	Pex5	P35056	203-227	EQQPWTDQFEKLEKEVSENL DINDEIEK	two hybrid	pull down	alanine scanning (4)	protein import in peroxisome
12453410	Pex13	P80667	Pex5	P35056	198-214	GVNEQEQQPWTDQFEKLEKE	NMR titration	TOCSY/NOESY	CD spectroscopy	protein import in peroxisome
12595255	Pex13	P80667	Pex5	P35056	204-216	QQPWTDQFEKLEKEVS	spot synthesis overlay (2)	NMR titration	mutagenesis analysis (5)	protein import in peroxisome
11668184	Abp1	P15891	Prk1	P40494	748-760	RPPRPPPKPLHLR	phage display	pull down	two hybrid	localization of Prk in actin cortical patches
14737190	Abp1	P15891	Prk1	P40494	743-756	GKDKSRPPRPPPKP	spot synthesis overlay (2)	surface plasmon resonance	CoIP (6)	localization of Prk in actin cortical patches
14737190	Abp1	P15891	Scp1	Q08873	151 – 164	LSTKKPRPPVKSKP	spot synthesis overlay (2)	surface plasmon resonance	CoIP (6)	localization of Scp1 in actin cortical patches
17409071	Abp1	P15891	Scp1	Q08873	154 - 170	KKPRPPVKSKPKHLQDG	isothermal titration calor	pull down		localization of Scp1 in actin cortical patches
11668184	Abp1	P15891	Sjl2	P50942	1115-1121	PPVVKKP	phage display			recruit Sjl2 at cortical actin patches

Table S11

PMID	SH3 domain protein name	SPID domain protein	Ligand name	SPID ligand	A.A range	Sequence	Method 1	Method 2	Method 3	Biological Relevance
15798181	Abp1	P15891	Sjl2	P50942	1115-1121	PPVVKKP	CoIP [6]	cosedimentation analysis	colocalization analysis	recruit Sjl2 at cortical actin patches
12388763	Ysc84	P32793	Sla1p	P32790	118-511	KEDQAPDEDEEGPPPAMPAR PTATTETTDATAAAVRSRTRLS YSDNDNDDEEDDYYYNSNSN NVGNHEYNTEYHSWNVTEIE GRKKKKAKLSIGNNKINFIPQK GTPHEWSIDKLVSYDNEKKHM FLEFVDPYRSLELHTGNTTTC EEIMNIIGEYKGASRDPGLREV EMASKSKKRGIVQYDFMAES QDELTIKSGDKVYILDDKKSKD WWMCQLVDSGKSGLVPAQFI EPVRDKKHTESTASGIIKSIKK NFTKSPSRSRSRSRSKSNAN ASWKDDELQNDVVGSAAGKR SRKSSLSSHKKNSSATKDFPN PKKSRLWVDRSGTFKVD	two-hybrid	pull down		actin dinamics and endocytosis
12388763	Lsb3c	P43603	Sla1p	P32790	118-511	KEDQAPDEDEEGPPPAMPAR PTATTETTDATAAAVRSRTRLS YSDNDNDDEEDDYYYNSNSN NVGNHEYNTEYHSWNVTEIE GRKKKKAKLSIGNNKINFIPQK GTPHEWSIDKLVSYDNEKKHM FLEFVDPYRSLELHTGNTTTC EEIMNIIGEYKGASRDPGLREV EMASKSKKRGIVQYDFMAES QDELTIKSGDKVYILDDKKSKD WWMCQLVDSGKSGLVPAQFI EPVRDKKHTESTASGIIKSIKK NFTKSPSRSRSRSRSKSNAN ASWKDDELQNDVVGSAAGKR SRKSSLSSHKKNSSATKDFPN PKKSRLWVDRSGTFKVD	two-hybrid			actin dinamics

Table S11

PMID	SH3 domain protein name	SPID domain protein	Ligand name	SPID ligand	A.A range	Sequence	Method 1	Method 2	Method 3	Biological Relevance
12734398	Sla1_2	P32790	Sla2	P33338	310-768	VSQRTTSTPTGYLQTMPTGATTGMMIPTATGAANAIFPQATAQMQPDFWANQQAQFANEQNRLEQERVQQLQQQQAQQELFQQQLQKAQQDMMNMQLQQQNQHQNDLILTNQYEKDQALLQQYDQRVQQLESEITTMDSTASKQLANKDEQLTALQDQLDVWERKYESLAKLYSQLRQEHLNLLPRFKKLQLKVNSAQESIQKKEQLEHKLKQKDLQMAELVKDRDRARLELERSINNAEADSAAATAAAETMTQDKMNPILDAILESGINTIQESVYNLDSPLSWSGPLTPPTFLLSLLESTSENATEFATSFNNLIVDGLAHGDQTEVIHCVSDFSTSMATLVTNSKAYAVTTLPQEQSDQILTLVKRCAREAQYFFEDLMSENLNQVGDEEKTDIVINANVDMQEKLQELSLAIEPLLNIQSVKSNKETNPHSELVATADKIVKSSEHLRVDVP	two-hybrid	GST-pull down	HIS-pull down	sla1p and actin localization at endocytic sites
8552082	Abp1	P15891	Srv2	P17555	350-367	SKSGPPPRPKKPSTLKT	overlay assay (1)	pull down	competition assay	association of Srv2 with actin cytoskeleton
9190214	Abp1	P15891	Srv2	P17555	337-376	PELRQSSTVSSTGSKSGPPPRPKKPSTLKTKRPPRKELVG	overlay assay (1)	colocalization analysis		association of Srv2 with actin cytoskeleton
14737190	Abp1	P15891	Srv2	P17555	348 – 361	TGSKSGPPPRPKKP	spot synthesis overlay (2)	surface plasmon resonance	CoIP (6)	association of Srv2 with actin cytoskeleton
17409071	Abp1	P15891	Srv2	P17555	351 – 367	KSGPPPRPKKPSTLKTK	isothermal titration calor	pull down		association of Srv2 with actin cytoskeleton
16778768	Sho1	P40073	Ste11	P23561	340-413	RRPLSAESNNIGDILLKHSNAVDMALLQGLDQTRLSSKLDTTKIPKLAHKRPEDNDAISNQLELLSVESGEEED	CoIP (6)	affinity coprecipitation (7)		HOG MAPK pathway
15743816	Bem1	P29366	Ste20	Q03497	469-484	NGKFIPSRPAPKPPSSAS	two hybrid	affinity coprecipitation (7)		localization of Ste20 at buds and mating projections
15743816	Nbp2	Q12163	Ste20	Q03497	469-484	NGKFIPSRPAPKPPSSAS	two hybrid	affinity coprecipitation (7)		activation of ste20 kinase
18280496	Fus1	P11710	Ste5	P32917	111 – 122	LPQHPHRTSSLP	phage display	fluorescence titration	alanine scanning (4)	mating pathway
16778768	Sho1	P40073	Ste50	P25344	149-235	TSSSSSPINTHGVSTTVPSSNNTIIPSSDGVSLSQTDYFDTVHNRQSPSRRESPVTVFRQPSLSHSKSLHKDSKNKVPQISTNQSHPSAVS	CoIP (6)	affinity coprecipitation (7)		Hog MAPK pathway

Table S11

PMID	SH3 domain protein name	SPID domain protein	Ligand name	SPID ligand	A.A range	Sequence	Method 1	Method 2	Method 3	Biological Relevance
9628892	Myo5	Q04439	Vrp1	P37370	1-200	MAGAPAPPPPPPPPPALGGSA<br>PKPAKSVMQGRDALLGDIRKG<br>MKLKKAETNDRSAPIVGGGVV<br>SSASGSSGTVSSKGPSMSAP<br>PIPGMGAPQLGDILAGGIPKLK<br>HINNNASTKPSPSASAPPIPGA<br>VPSVAAPPIPNAPLSPAPVPSI<br>PSSSAPPIPDIPSSAAPPIPIVP<br>SSPAPPLP<br>LSGASAPKVPQNRPHMPSVR	two hybrid	CoIP [6]	colocalization analysis	Actin Polarization
9628892	Myo5	Q04439	Vrp1	P37370	195-817	HMPSVRPAHRSHQRKSSNISL<br>PSVSAPPLPSASLPTHVSNPP<br>QAPPPPPTPTIGLDSKNIKPTD<br>NAVSPPSSEVPAGGLPFLAEIN<br>ARRSERGAVEGVSSTKIQTEN<br>HKSPSQPPLPSSAPPIPTSHA<br>PPLPPTAPPPPSLPNVTSAPK<br>KATSAPAPPPPPLPAAMSSAS<br>TNSVKATPVPPTLAPPLPNTTS<br>VPPNKASSMPAPPPPPPPPP<br>GAFSTSSALSASSIPLAPLPPP<br>PPPSVATSVPSAPPPPPTLTTN<br>KPSASSKQSKISSSSSSSAVT<br>PGGPLPFLAEIQKKRDDRFVV<br>GGDTGYTTQDKQEDVIGSSK<br>DDNVRPSPISPSINPPKQSSQ<br>NGMSFLDEIESKLHKQTSSNA<br>FNAPPPHTDAMAPPLPPSAPP<br>PPITSLPTPTASGDDHTNDKS<br>ETVLGMKKAKAPALPGHVPPP<br>PVPPVLSDDSKNNVPAASLLH<br>DVLPSSNLEKPPSPPVAAAPP<br>LPTFSAPSLPQQSVSTSIPSPP<br>PVAPTLSVRTETESISKNPTKS<br>PPPPPSPSTMDTGTSNSPSK<br>NLKQRLFSTGGSTLQHKHNTH<br>TNQPDVDVGRYTIGGSNSIVG<br>AKSGNERIVIDDSRFKWTNVS<br>QMPKPRPFQNKTKLYPSGKG<br>SSVPLDLTLFT	two hybrid	CoIP [6]	colocalization analysis	Actin Polarization
10648568	Myo3	P36006	Vrp1	P37370	211-437	QRKSSNISLPSVSAPPLPSASL<br>PTHVSNPPQAPPPPPTPTIGL<br>DSKNIKPTDNAVSPPSSEVPA<br>GGLPFLAEINARRSERGAVEG<br>VSSTKIQTENHKSPSQPPLPS<br>SAPPIPTSHAPPLPPTAPPPPS<br>LPNVTSAPKKATSAPAPPPPP<br>LPAAMSSASTNSVKATPVPPT<br>LAPPLPNTTSVPPNKASSMPA<br>PPPPPPPPPGAFSTSSALSAS<br>SIPLAPLPPPPPPSVATSVPSA<br>PP	two hybrid	pull down	CoIP [6]	actin assembly

Table S11

PMID	SH3 domain protein name	SPID domain protein	Ligand name	SPID ligand	A.A range	Sequence	Method 1	Method 2	Method 3	Biological Relevance
11260524	Hof1	Q05080	Vrp1	P37370	1-267	MAGAPAPPPPPPPPALGGSA PKPAKSVMQGRDALLGDIRKG MKLKKAETNDRSAPIVGGGVV SSASGSSGTVSSKGPSMSAP PIPGMGAPQLGDILAGGIPKLK HINNNASTKPSPSASAPPIPGA VPSVAAPPIPNAPLSPAPAVPSI PSSSAPPIPDIPSSAAPPIPIVP SSPAPPLPLSGASAPKVPQNR PHMPSVRPAHRSHQRKSSNIS LPSVSAPPLPSASLPTHVSNP PQAPPPPPTPTIGLDSKNIKPT DNAVSPPSSEVPAGGLP	two hybrid	GST-pull down	HIS-pull down	localization of Hof1 to the actomyosin ring at the bud neck
15941409	Hof1	Q05080	Vrp1	P37370	145-174	AVPSIPSSSAPPIPDIPSSAAPP IPIVPSS	two hybrid	pull down	alanine scanning (4)	regulatation of cytokinesis
15941409	Myo3	P36006	Vrp1	P37370	168-174	PPIPIVPSS	two hybrid	alanine scanning (4)		actin filament assembly
14737190	Abp1	P15891	Yir003W	P40563	383 – 396	RPKRRAPPPVPKKP	spot synthesis overlay (2)	surface plasmon resonance	CoIP (6)	unknown
11668184	Abp1	P15891	Ynl094	P53933	467-504	RRPPPPPIPSTQKPSLTEEQT ESIRMSRRNKDENNAKR	phage display			actin filament assembly
14737190	Abp1	P15891	Ynl094	P53933	467 - 483	RRPPPPPIPSTQKPSLT	spot synthesis overlay (2)	surface plasmon resonance	CoIP (6)	actin filament assembly
