# Supplementary material for: Bayesian Modeling of the Yeast SH3 Domain Interactome Predicts Spatiotemporal Dynamics of Endocytosis Proteins
Source: PLoS Biol. 2009 Oct 20;7(10):e1000218. doi: 10.1371/journal.pbio.1000218 (PMC2756588; doi:10.1371/journal.pbio.1000218)
Supplement: Table S12 — Predicted yeast SH3 domain interactions based on Bayesian networks. The yeast proteome was scanned for putative SH3 domain interactions based on our Bayesian model. The Bayesian model assigns a probability score based on how well a given interaction scored across all three independent techniques, where higher scores represent higher confidence predictions. To obtain a wider spectrum of scores, the probability scores were scaled by taking −log(1 − Probability score). The best matching peptide based on a PWM scoring algorithm from each predicted gene is shown. (0.11 MB PDF) [file pbio.1000218.s021.pdf]

Table S12. Predicted yeast SH3 domain interactions based on Bayesian networks

SH3 domain	Predicted gene name	Probability score	Rescaled score	Best PWM match
Abp1	CLN3	0.876841404	2.094282357	SQPQVNPKMRFLIFD
Abp1	AIM3	0.99811599	6.274353058	SFPPPPLKPGQKTYT
Abp1	STP22	0.957402648	3.155963183	NTPPLPPKPKSPHLK
Abp1	TSR1	0.835917034	1.80738309	QSPAIFFKPSPTDAK
Abp1	MTC5	0.835917034	1.80738309	QWSPHPAKPYWIVST
Abp1	CTH1	0.957402648	3.155963183	PSPWLPSKPNCYHPL
Abp1	MTH1	0.820753441	1.718992998	SPPPATSKNQVLQRR
Abp1	RAD30	0.957402648	3.155963183	HLPLIPEKIKSLKFE
Abp1	SNX41	0.833065933	1.79015635	APPLSPTKPSPLHSI
Abp1	FIR1	0.957402648	3.155963183	PPPLTPEKNLYLTPE
Abp1	GIP2	0.876841404	2.094282357	FAPPVYKKSGELLKS
Abp1	MDS3	0.835917034	1.80738309	RMPSNPALPKKLLNV
Abp1	GSC2	0.835917034	1.80738309	RPPIYSLKQARLRKR
Abp1	TOS2	0.835917034	1.80738309	SKLSVPSKHIDLSNK
Abp1	DIE2	0.996349278	5.612830456	TLPFGPVKSIWLSAF
Abp1	RIM101	0.876841404	2.094282357	ILPPLQSKIPMLPSR
Abp1	PIG2	0.876841404	2.094282357	PSPPIYKKSGELVKS
Abp1	PRK1	0.999998414	13.3540484	RPPRPPPKPLHLRTE
Abp1	AIM21	0.999616496	7.86615938	APPPVPKKPSSRIAA
Abp1	BBC1	0.991037831	4.714743048	SAPPVPPAPPALSAP
Abp1	GPI13	0.833065933	1.79015635	SQPPDVLKPQTLLGR
Abp1	RPL15A	0.833065933	1.79015635	ARPTRPDKARRLGYK
Abp1	VRP1	0.957402648	3.155963183	PAPPPPPPPPALGGS
Abp1	YTA12	0.833065933	1.79015635	PPPPPPPKPPLNDPS
Abp1	RPL15B	0.833065933	1.79015635	ARPTRPDKARRLGYK
Abp1	BUL1	0.835917034	1.80738309	LIPEDVKKPLEKLDL
Abp1	ARK1	0.999984454	11.07167998	TKPTPPPKPSHLKPK
Abp1	APP1	0.999188998	7.117239524	RRPPPPPIPSTQKPS
Abp1	INP52	0.999255819	7.203226043	EKPPVVKKPHYLSVA
Abp1	SRV2	0.999616496	7.86615938	GPPPRPKKPSTLKTK
Abp1	SIP3	0.957402648	3.155963183	VPPKSPEKSGWLYMK
Abp1	CLA4	0.876841404	2.094282357	YLNEDPLKALYLIAT
Abp1	HAL9	0.820753441	1.718992998	ILPDLIDKKEDLDKI
Abp1	ASE1	0.957402648	3.155963183	TVPQSPKKPLSLLSK
Abp1	RPO31	0.961583486	3.259267849	IPPPAIMKPYYLWTG
Abp1	LAS17	0.835917034	1.80738309	SLPPLPNQFAPLPDP
Abp1	SNF2	0.833065933	1.79015635	NNPPKPQKPVPLNVL
Abp1	MIP1	0.835917034	1.80738309	DKPDFYLKDPWLSQL
Abp1	SCP1	0.999616496	7.86615938	PRPPVKSKPKHLQDG
Abp1	VTC3	0.833065933	1.79015635	QLPPGVKKPVHLLKN
Abp1	SRP68	0.876841404	2.094282357	KMLPIPSKPTLFDLA
Abp1	FHL1	0.876841404	2.094282357	KPPKIPKKVYTLEEI
Abp1	BSP1	0.999255819	7.203226043	SKPSLPEKPQKLRNA
Bbc1	SLA1	0.999616496	7.86615938	KELPPIKPPRPTSTT

Table S12

SH3 domain	Predicted gene name	Probability score	Rescaled score	Best PWM match
Bbc1	AIM3	0.994340487	5.174417433	NVEPPPPPSRGNFER
Bbc1	RPO21	0.991037831	4.714743048	CLPVPPPPVRPSISF
Bbc1	CRD1	0.876841404	2.094282357	IVRYPSAEVRPSQLS
Bbc1	CMR1	0.835917034	1.80738309	IGSMAINPMRPYEIA
Bbc1	MTC5	0.835917034	1.80738309	ITAADELKARPKRYV
Bbc1	HSP42	0.957402648	3.155963183	QGYPRQPQRPQRYHP
Bbc1	PRP28	0.876841404	2.094282357	LIKMSRSPPRPPSLK
Bbc1	HXT7	0.833065933	1.79015635	EPVVEIPKRPASAYV
Bbc1	SAC7	0.991037831	4.714743048	TLKRPPLSSRPYSYN
Bbc1	SAP1	0.996349278	5.612830456	APALPSLPPPPLLNV
Bbc1	ILV1	0.864848328	2.001357636	RIISFEFPERPGALT
Bbc1	MIG1	0.864848328	2.001357636	RYSNTVILPRPRSLT
Bbc1	YBP2	0.999255819	7.203226043	VPIMPTLPPRPYITI
Bbc1	LSG1	0.876841404	2.094282357	EPLLPPLPGQPPLIN
Bbc1	HUA1	0.833065933	1.79015635	LQSQPPRPPRPAANL
Bbc1	STE20	0.835917034	1.80738309	SANGKFIPSRPAPKP
Bbc1	TID3	0.820753441	1.718992998	QQHLSNRDPRPLRDK
Bbc1	BNR1	0.961583486	3.259267849	LPQLPPPPPPPPPPP
Bbc1	AIM21	0.999984454	11.07167998	EVTPKVPERPSRRKT
Bbc1	BBC1	0.833065933	1.79015635	APSVPPVPPVPPVSS
Bbc1	BCK1	0.957402648	3.155963183	SRTEPSTPSRPVPPD
Bbc1	PBS2	0.876841404	2.094282357	RPLSTQHPTRPNVAP
Bbc1	CDC6	0.995939888	5.506544722	FDDAPATPPRPLKRK
Bbc1	HSL1	0.991037831	4.714743048	EMPLPQLPKSPSRYS
Bbc1	ACF2	0.999616496	7.86615938	QAIPPPVPNRPGGTT
Bbc1	PEX13	0.833065933	1.79015635	SESAPEVLPRPAALN
Bbc1	VRP1	0.999172096	7.096613123	PTKSPPPPPSPSTMD
Bbc1	UBX2	0.991037831	4.714743048	TLVPPALGPRPLLFT
Bbc1	PIF1	0.908166568	2.387778867	STLNHIIPRRPFICS
Bbc1	MYO5	0.995939888	5.506544722	ANIPPPPPPPPPSSK
Bbc1	ADD37	0.991037831	4.714743048	ATTPPPVLRRPSVTA
Bbc1	SSN8	0.864848328	2.001357636	KFLLHTLYLRPASAI
Bbc1	APP1	0.999700896	8.114717596	TRRRPPPPPIPSTQK
Bbc1	SRV2	0.991037831	4.714743048	APAPPPPPPAPPASV
Bbc1	RTT106	0.820753441	1.718992998	MASFLPVPEKPNLIY
Bbc1	BNI1	0.833065933	1.79015635	SSQPPPPPPPPPPVP
Bbc1	CLA4	0.996349278	5.612830456	PSPSPLNPYRPHHNM
Bbc1	NUP1	0.835917034	1.80738309	TERPPLLPILPIQRL
Bbc1	LAS17	0.957402648	3.155963183	APPPPPRASRPTPNV
Bbc1	HIS3	0.864848328	2.001357636	SRAVVDLSNRPYAVV
Bbc1	SNF2	0.876841404	2.094282357	NSEPPALESSPVTGD
Bbc1	MIP1	0.876841404	2.094282357	CMLRMVRRRPLRVQF
Bbc1	FHL1	0.833065933	1.79015635	IVQTPHVPDRPPSQL
Bbc1	BSP1	0.991037831	4.714743048	NEEKPLLPTRPNKAE
Bem1-2	BOI1	0.820753441	1.718992998	QEVSKSPTRVPEVST
Bem1-2	AIM3	0.876841404	2.094282357	LKDPKSFPPPPLKPG
Bem1-2	MPS1	0.999616496	7.86615938	VFYHRPAPKPPVTKK
Bem1-2	LDB17	0.833065933	1.79015635	KRKAKAPPPPPPPPP
Bem1-2	AMD2	0.833065933	1.79015635	YDSNRPAILPPLYGI

Table S12

SH3 domain	Predicted gene name	Probability score	Rescaled score	Best PWM match
Bem1-2	PRP28	0.999255819	7.203226043	IKMSRSPPRPPSLKI
Bem1-2	BOI2	0.864848328	2.001357636	FVSPRRAPKPPSYPS
Bem1-2	STE20	0.999998414	13.3540484	FIPSRPAPKPPSSAS
Bem1-2	PRK1	0.991037831	4.714743048	KDKSRPPRPPPKPLH
Bem1-2	UBP7	0.999255819	7.203226043	RLRKRPPPPPPVSMP
Bem1-2	AIM21	0.999255819	7.203226043	RPKRRAPPPVPKKPS
Bem1-2	BBC1	0.957402648	3.155963183	PPLPRAPPVPPATFE
Bem1-2	VRP1	0.833065933	1.79015635	KNPTKSPPPPPSPST
Bem1-2	TUS1	0.991037831	4.714743048	VKERRPPPPPPLLYS
Bem1-2	MYO5	0.999255819	7.203226043	RHSKKPAPPPPGMQN
Bem1-2	APP1	0.991037831	4.714743048	VRTRRRPPPPPIPST
Bem1-2	CLA4	0.999851542	8.815210152	FQPQRTAPKPPISAP
Bem1-2	TRM10	0.991037831	4.714743048	EKVKRTPPLPPVPEG
Bem1-2	INP53	0.833065933	1.79015635	KPVLRPPPPPAHKSV
Bem1-2	LAS17	0.833065933	1.79015635	TTKHKAPPPPPPTAE
Boi1	COQ1	0.602620488	0.922863506	LFQRPASSISPLHIL
Boi1	EXO84	0.995939888	5.506544722	YNKVPARSAMRNTVN
Boi1	KCC4	0.999172096	7.096613123	SSPIPLRSSKRITHI
Boi1	AFR1	0.995939888	5.506544722	RNQPPPRSSKRPSLD
Boi1	IRC3	0.833065933	1.79015635	APVYPLKSLLRILEY
Boi1	RGA2	0.833065933	1.79015635	PIKLPERSVKRPLSP
Boi1	DIG2	0.864848328	2.001357636	TRFPQHKNSLRVRYM
Boi1	SEC3	0.999996921	12.69075568	GTYVPERSTARAHDQ
Boi1	MIG3	0.833065933	1.79015635	VSLPPIKSILRQIDN
Boi1	FIR1	0.999998414	13.3540484	SIEIPKRSPLRFTSS
Boi1	BEM2	0.999998414	13.3540484	AKLPPPRSSARLSKS
Boi1	MIG1	0.864848328	2.001357636	ETLPPIRSLPLPFPH
Boi1	YLF2	0.833065933	1.79015635	IPSVPLSNLLRIYQS
Boi1	YAP5	0.991037831	4.714743048	NWKLPPRLPHRAAQR
Boi1	INO1	0.876841404	2.094282357	LIGLPSQNELRFEER
Boi1	IML1	0.820753441	1.718992998	LLDLPSRSRIWIKLK
Boi1	RRN3	0.833065933	1.79015635	QVALPSKNPERINDK
Boi1	VPS13	0.820753441	1.718992998	LQVPRERVELRVTSL
Boi1	ZDS2	0.999998414	13.3540484	GLTIPERSSLRRSKF
Boi1	ZDS1	0.833065933	1.79015635	TMTWPERSSLRRSRF
Boi1	RTT106	0.820753441	1.718992998	LEGVSVLSPLRKKLD
Boi1	NBA1	0.991945222	4.821489883	NPTIPPRSKDRPRSR
Boi1	BEM3	0.999616496	7.86615938	SFVLPPRSAERKAHI
Boi2	GEM1	0.833065933	1.79015635	QDVLPPISIPRDFSS
Boi2	RIF1	0.833065933	1.79015635	NELMPPDSPPRMTEN
Boi2	VPS54	0.833065933	1.79015635	KIVVPEGSPSRNSDS
Boi2	AFR1	0.995939888	5.506544722	RNQPPPRSSKRPSLD
Boi2	YAP6	0.864848328	2.001357636	SYIIPPHSNERKQSA
Boi2	SSF2	0.833065933	1.79015635	QNIFPPLNPARTSLN
Boi2	SWR1	0.833065933	1.79015635	VVDVPVPSLLRGNLR
Boi2	SEC3	0.991037831	4.714743048	GTYVPERSTARAHDQ
Boi2	FIR1	0.999984454	11.07167998	SIEIPKRSPLRFTSS
Boi2	BEM2	0.999616496	7.86615938	AKLPPPRSSARLSKS
Boi2	TIM21	0.833065933	1.79015635	MSSSLPRSLLRLGHR

Table S12

SH3 domain	Predicted gene name	Probability score	Rescaled score	Best PWM match
Boi2	YLF2	0.833065933	1.79015635	IPSVPLSNLLRIYQS
Boi2	SSF1	0.833065933	1.79015635	QNIFPPLNPARTSLN
Boi2	PAN1	0.991037831	4.714743048	RSGLPPSQLARIWTL
Boi2	ERG3	0.833065933	1.79015635	ASLLPRSSILREFLS
Boi2	SMC4	0.833065933	1.79015635	YSQSPPRSPGRSPTR
Boi2	ZDS2	0.999984454	11.07167998	GLTIPERSSLRRSKF
Boi2	NAM7	0.864848328	2.001357636	VILGNPRSLARNTLW
Boi2	NBA1	0.833065933	1.79015635	NPTIPPRSKDRPRSR
Bzz1-1	STP22	0.833065933	1.79015635	LKPPLPPPPPPQPAS
Bzz1-1	PAT1	0.833065933	1.79015635	PSQFAPPPPPPGVNV
Bzz1-1	LDB17	0.999996921	12.69075568	RKAKAPPPPPPPPPS
Bzz1-1	PRP8	0.833065933	1.79015635	SDLALPPPPPPPPGY
Bzz1-1	UBP7	0.999998414	13.3540484	RLRKRPPPPPPVSMP
Bzz1-1	BNR1	0.833065933	1.79015635	KLPQLPPPPPPPPPP
Bzz1-1	AIM21	0.996349278	5.612830456	RPKRRAPPPVPKKPS
Bzz1-1	PTK1	0.833065933	1.79015635	VLASSPPPPPPATHV
Bzz1-1	EAP1	0.833065933	1.79015635	SETSTPPPPPPGLIA
Bzz1-1	ACF2	0.833065933	1.79015635	TTNRGPPPLPPRANV
Bzz1-1	VRP1	0.833065933	1.79015635	PLAPLPPPPPPSVAT
Bzz1-1	MYO5	0.995939888	5.506544722	QANIPPPPPPPPPSS
Bzz1-1	APP1	0.999984849	11.09745922	TRRRPPPPPIPSTQK
Bzz1-1	SRV2	0.833065933	1.79015635	TAAPAPPPPPPAPPA
Bzz1-1	BNI1	0.833065933	1.79015635	GDSPAPPPPPPPPPP
Bzz1-1	LAS17	0.999996921	12.69075568	TKHKAPPPPPPTAET
Bzz1-1	SCD5	0.833065933	1.79015635	QPQHLPPPPPPRAQQ
Bzz1-1	TIM50	0.833065933	1.79015635	FPDLLPPPPPPPYQR
Bzz1-2	AIM3	0.999851542	8.815210152	PKSFPPPPLKPGQKT
Bzz1-2	STP22	0.833065933	1.79015635	LKPPLPPPPPPQPAS
Bzz1-2	LDB17	0.999998414	13.3540484	RKAKAPPPPPPPPPS
Bzz1-2	MPT5	0.820753441	1.718992998	LEPVTPPPLGQMNNK
Bzz1-2	PRP8	0.833065933	1.79015635	SDLALPPPPPPPPGY
Bzz1-2	UBP7	0.999998414	13.3540484	RLRKRPPPPPPVSMP
Bzz1-2	BNR1	0.833065933	1.79015635	KLPQLPPPPPPPPPP
Bzz1-2	AIM21	0.932525543	2.696006168	RPKRRAPPPVPKKPS
Bzz1-2	BBC1	0.864848328	2.001357636	SHTAPSPPPHQNVTA
Bzz1-2	PBS2	0.957402648	3.155963183	IVNKPLPPLPVAGSS
Bzz1-2	NFT1	0.820753441	1.718992998	ENKKLPPAPTVFGLL
Bzz1-2	VRP1	0.991037831	4.714743048	GAPAPPPPPPPPALG
Bzz1-2	TUS1	0.999255819	7.203226043	VKERRPPPPPPLLYS
Bzz1-2	YTA12	0.833065933	1.79015635	SRNIPPPPPPPPPKP
Bzz1-2	MYO5	0.999172096	7.096613123	QANIPPPPPPPPPSS
Bzz1-2	APP1	0.999998414	13.3540484	TRRRPPPPPIPSTQK
Bzz1-2	BNI1	0.833065933	1.79015635	LSSQPPPPPPPPPPV
Bzz1-2	INP53	0.995939888	5.506544722	PVLRPPPPPAHKSVS
Bzz1-2	LAS17	0.999996921	12.69075568	TKHKAPPPPPPTAET
Bzz1-2	MKK1	0.876841404	2.094282357	MKKRPAPPSLPSLSI
Bzz1-2	SCD5	0.833065933	1.79015635	QPQHLPPPPPPRAQQ
Bzz1-2	TIM50	0.833065933	1.79015635	FPDLLPPPPPPPYQR
Cdc25	BUL1	0.936954512	2.763898782	FSHCLLPPSFGIDKY

Table S12

SH3 domain	Predicted gene name	Probability score	Rescaled score	Best PWM match
Cyk3	FUN30	0.833065933	1.79015635	KGKPRLLSPEISLKD
Cyk3	FUS3	0.833065933	1.79015635	KMFPRVNPKGIDLLQ
Cyk3	OLA1	0.833065933	1.79015635	PEEARVIVPSPRFDK
Cyk3	AIM3	0.833065933	1.79015635	NAPERAVPILPPRNN
Cyk3	SPO71	0.833065933	1.79015635	MKAYRANPPLPIDSM
Cyk3	MSS4	0.833065933	1.79015635	MSVLRSQPPSVVPLH
Cyk3	FIR1	0.833065933	1.79015635	PKKSRVLPPLPFPLY
Cyk3	SER3	0.820753441	1.718992998	FSNSRSVAELVIAEI
Cyk3	AIM10	0.833065933	1.79015635	SKSNFPDFPLKKYLK
Cyk3	YBP2	0.833065933	1.79015635	SVPIMPTLPPRPYIT
Cyk3	LSG1	0.833065933	1.79015635	APNEPLLPPLPGQPP
Cyk3	ROG1	0.833065933	1.79015635	SSILLPPLPERAYIM
Cyk3	ACB1	0.820753441	1.718992998	AKAVNELPTKPSTDE
Cyk3	SKN1	0.833065933	1.79015635	QPPDRNLPSHPSSNN
Cyk3	PHO81	0.833065933	1.79015635	VVPLRSLLEVIPGSA
Cyk3	RNH70	0.833065933	1.79015635	EELVKPDVPIVDYLT
Cyk3	UBP7	0.833065933	1.79015635	PIRLRKRPPPPPPVS
Cyk3	TAX4	0.833065933	1.79015635	YSYLRLLPWWPSLAN
Cyk3	ELM1	0.833065933	1.79015635	DTFCRSNESLPNLTV
Cyk3	BCH2	0.833065933	1.79015635	LAYERPLPDLPSTIK
Cyk3	TPO1	0.833065933	1.79015635	MGGDRPYPPSLPSRD
Cyk3	PUT1	0.833065933	1.79015635	YVPWGPPLETKDYLL
Cyk3	SKG3	0.833065933	1.79015635	DSLLFALPTLPHIYY
Cyk3	MSC3	0.833065933	1.79015635	KMTLRSSSDSPTATA
Cyk3	VPS34	0.833065933	1.79015635	ETKVRPLVKVRPIAL
Cyk3	VIP1	0.833065933	1.79015635	KPLLREGKEAPPQFA
Cyk3	TUS1	0.833065933	1.79015635	VKERRPPPPPPLLYS
Cyk3	SIR3	0.833065933	1.79015635	FSYLRWFELKPKLYY
Cyk3	INN1	0.999172096	7.096613123	SMAMRPIPPLPTESE
Cyk3	NBA1	0.999172096	7.096613123	TYLTRPLPSTPNEDS
Cyk3	HMI1	0.833065933	1.79015635	SELVRKLPSGVSPQI
Cyk3	LAS17	0.833065933	1.79015635	NRNNRPVPPPPPMRT
Cyk3	HIS3	0.864848328	2.001357636	AVAIREATSPNGTND
Cyk3	AIM44	0.864848328	2.001357636	SRLVRSKPSTALNAI
Fus1	GIP4	0.999998414	13.3540484	INGRRPRSSSLQSYT
Fus1	SMY2	0.991037831	4.714743048	GRHPLSRTSSLIDSI
Fus1	GID7	0.833065933	1.79015635	IPTPTPATTSLFDHM
Fus1	PBN1	0.833065933	1.79015635	QRWLLERTGSLDKSF
Fus1	EBS1	0.999255819	7.203226043	DLERQMRSSSLDSFS
Fus1	TRP2	0.833065933	1.79015635	PSQRVARPTSLHPFN
Fus1	CDC20	0.876841404	2.094282357	SKNSLKRSSSLNIRN
Fus1	ESP1	0.957402648	3.155963183	KNYREFRVSSLIAPN
Fus1	BNR1	0.999996921	12.69075568	KISLPKRSTSLLKSK
Fus1	RTT101	0.991037831	4.714743048	LRVVLPRTASLQSSN
Fus1	FMP46	0.999255819	7.203226043	TLQRQPRTISLFTND
Fus1	ERG3	0.833065933	1.79015635	FASLLPRSSILREFL
Fus1	SIP5	0.999998414	13.3540484	SRVRTRRTTSLVNNI
Fus1	PSE1	0.833065933	1.79015635	DLEPPARTTALELLT
Fus1	BOP3	0.833065933	1.79015635	KKHRRARSTSSFGVI

Table S12

SH3 domain	Predicted gene name	Probability score	Rescaled score	Best PWM match
Fus1	PIK1	0.864848328	2.001357636	LHNIQPRTSSASSAS
Fus1	MUK1	0.999616496	7.86615938	SLMRPRRSSSLFSNE
Fus1	PRM4	0.833065933	1.79015635	TTTLNPRSSSLALQK
Fus1	MMT2	0.999255819	7.203226043	AAGRAIRTSSLYSTM
Fus1	HAA1	0.864848328	2.001357636	PFTRKPRSSSIDVNH
Fus1	EAF3	0.820753441	1.718992998	GNDDRRRSSSLSPNM
Hof1	AIM3	0.820753441	1.718992998	TVDVSSLPPPPTHRD
Hof1	ALG1	0.957402648	3.155963183	VQNPPSIPILPIAVL
Hof1	AGP2	0.876841404	2.094282357	HVQQDSLPKLPFRSW
Hof1	STP22	0.999984849	11.09745922	QDQAPSLPPKPNTQL
Hof1	CYK3	0.999998414	13.3540484	NNPLPPLPPLPDLDN
Hof1	SDH4	0.999172096	7.096613123	SLTIPFLPVLPQKPG
Hof1	MSN5	0.876841404	2.094282357	PRIVPILPYITRLLY
Hof1	SIZ1	0.999616496	7.86615938	QNSTPVLPTLPQNVP
Hof1	FIR1	0.864848328	2.001357636	PKKSRVLPPLPFPLY
Hof1	ZRG8	0.876841404	2.094282357	TGPPPPLPPPLFPSS
Hof1	SAP1	0.999984454	11.07167998	PTTAPALPSLPPPPL
Hof1	UBP5	0.995939888	5.506544722	NVFSPRIPPLPQQNL
Hof1	YBP2	0.999992195	11.76074609	VPIMPTLPPRPYITI
Hof1	LSG1	0.999998414	13.3540484	APNEPLLPPLPGQPP
Hof1	RMD11	0.999616496	7.86615938	RSKFPSLPSLPIFLS
Hof1	RIM101	0.992996959	4.9614108	STSPQILPPLPVGIS
Hof1	KIC1	0.876841404	2.094282357	NKMRPHLPPLSSGNN
Hof1	UBP7	0.999700896	8.114717596	MPTTPEIPPPLPPKI
Hof1	BNR1	0.999998414	13.3540484	VVKLPQLPPPPPPPP
Hof1	BBC1	0.999816057	8.600886921	ALSAPSIPPVPPTPP
Hof1	ALY2	0.995939888	5.506544722	EQKGPKLPNLPNDAN
Hof1	PBS2	0.876841404	2.094282357	QIVNKPLPPLPVAGS
Hof1	BUD4	0.999998414	13.3540484	PEHVPLLPPLPRWEE
Hof1	HSL1	0.961583486	3.259267849	EMPLPQLPKSPSRYS
Hof1	VRP1	0.957402648	3.155963183	SAPAPPPPPLPAAMS
Hof1	TUS1	0.957402648	3.155963183	SIELPKLPPLNTRNS
Hof1	MYO5	0.864848328	2.001357636	QANIPPPPPPPPPSS
Hof1	APP1	0.999188998	7.117239524	TRRRPPPPPIPSTQK
Hof1	INN1	0.999998414	13.3540484	GLNSPKLPPLPTTSN
Hof1	NBA1	0.999998414	13.3540484	KNELPSLPMLPSEAT
Hof1	TRM10	0.833065933	1.79015635	VKRTPPLPPVPEGMS
Hof1	NUP1	0.833065933	1.79015635	TERPPLLPILPIQRL
Hof1	LAS17	0.999984849	11.09745922	TTPAPALPPASPEVR
Hof1	MKK1	0.996349278	5.612830456	RPAPPSLPSLSINSQ
Hof1	BRO1	0.995939888	5.506544722	NSPAPPLPPLDSKAS
Hof1	BEM3	0.864848328	2.001357636	KSHVPDLPLPTLPDR
Hof1	MKK2	0.961583486	3.259267849	RPVPPPLPPLVLTQK
Hse1	UBP13	0.995939888	5.506544722	TDRPPDVPRKIIVGR
Hse1	FIG1	0.991037831	4.714743048	YVTVPKLPFKLAVNK
Hse1	AIM3	0.935354115	2.738830825	ERAVPILPPRNNVEP
Hse1	CYC8	0.820753441	1.718992998	TNASPAPPPVILQPT
Hse1	ERT1	0.957402648	3.155963183	MNIQPDLPPRKIMIP
Hse1	PAF1	0.999255819	7.203226043	SLPVPQLPPKLLVYP

Table S12

SH3 domain	Predicted gene name	Probability score	Rescaled score	Best PWM match
Hse1	STP22	0.999962608	10.19404889	QDQAPSLPPKPNTQL
Hse1	GDA1	0.833065933	1.79015635	QLSSPCLPPKVNATN
Hse1	YBP2	0.999255819	7.203226043	VPIMPTLPPRPYITI
Hse1	MDS3	0.833065933	1.79015635	MPSNPALPKKLLNVP
Hse1	ADE3	0.833065933	1.79015635	PLQIPPLPLKLLTPV
Hse1	MES1	0.833065933	1.79015635	HSKFPELPSKVHNAV
Hse1	PRK1	0.876841404	2.094282357	KSRPPRPPPKPLHLR
Hse1	UBP7	0.999998414	13.3540484	PEIPPPLPPKIMVHS
Hse1	AIM21	0.876841404	2.094282357	RRAPPPVPKKPSSRI
Hse1	ZAP1	0.999616496	7.86615938	ESKPPQLPPKCSSLR
Hse1	ERM6	0.833065933	1.79015635	NIIKPYLPFKISKWL
Hse1	BPT1	0.833065933	1.79015635	FKNFPTLPSKINSRH
Hse1	ACF2	0.999984849	11.09745922	NRGPPPLPPRANVQP
Hse1	EST2	0.833065933	1.79015635	RCNEPHLPPKWVQRS
Hse1	MVP1	0.991037831	4.714743048	FRMIPELPPKRIGSQ
Hse1	GYL1	0.957402648	3.155963183	SSTPPTLPPRRIEDP
Hse1	APP1	0.992996959	4.9614108	ADVAPVVPPHLEEDS
Hse1	BNI1	0.991037831	4.714743048	PPPPPPVPAKLFGES
Hse1	ACC1	0.991945222	4.821489883	ASENPLLPEKLSQSK
Hse1	ALG8	0.991037831	4.714743048	FVILPQIPPKLTFIL
Hse1	SNX3	0.833065933	1.79015635	KVMVPHLPGKILLSN
Hse1	BSP1	0.991037831	4.714743048	TTSKPSLPEKPQKLR
Lsb1	CCR4	0.833065933	1.79015635	LRDNRPEIPLPHERR
Lsb1	PRX1	0.876841404	2.094282357	AQLKRTAWTLPKQAH
Lsb1	RPL23A	0.835917034	1.80738309	KKVMPAIVVRQAKSW
Lsb1	AIM3	0.999616496	7.86615938	ERAVPILPPRNNVEP
Lsb1	AGP2	0.932525543	2.696006168	QDSLPKLPFRSWGQP
Lsb1	IRA1	0.833065933	1.79015635	LFFDRLLLVLPIESN
Lsb1	YPC1	0.835917034	1.80738309	VHVRRSILALPLGVL
Lsb1	ERT1	0.999616496	7.86615938	MNIQPDLPPRKIMIP
Lsb1	RIF1	0.833065933	1.79015635	ELMPPDSPPRMTENT
Lsb1	PAT1	0.932525543	2.696006168	QFAPPPPPPGVNVNM
Lsb1	ABP1	0.999616496	7.86615938	SSAAPPPPPRRATPE
Lsb1	MPS1	0.876841404	2.094282357	VFYHRPAPKPPVTKK
Lsb1	CMR1	0.996349278	5.612830456	KSASPTLPTRRSRRL
Lsb1	MTC5	0.876841404	2.094282357	DLDDPFTPPRWLHHI
Lsb1	CTH1	0.99811599	6.274353058	EPCRRAPLQLPQLVN
Lsb1	CDC1	0.876841404	2.094282357	NQIFPKKPLRRTVMS
Lsb1	PRP28	0.835917034	1.80738309	LNIIPRDLLRVIIQE
Lsb1	GLC3	0.833065933	1.79015635	GFDYRLAMALPDMWI
Lsb1	ZRG8	0.876841404	2.094282357	TGPPPPLPPPLFPSS
Lsb1	RPS26B	0.932525543	2.696006168	KNRAPPQRPRFNRDN
Lsb1	VTC2	0.932525543	2.696006168	LNSAPTLRLRWTGQL
Lsb1	YBP2	0.99811599	6.274353058	VPIMPTLPPRPYITI
Lsb1	ITC1	0.99811599	6.274353058	VLYKRKPILLPDPKP
Lsb1	RAD54	0.99811599	6.274353058	AGERPRLVPRPINVQ
Lsb1	GTS1	0.99811599	6.274353058	TTSTPPLPRRRATTS
Lsb1	RPS26A	0.932525543	2.696006168	KNRAPPQRPRFNREN
Lsb1	MDS3	0.876841404	2.094282357	LELEPLLTPRSLYMP

Table S12

SH3 domain	Predicted gene name	Probability score	Rescaled score	Best PWM match
Lsb1	CHC1	0.957402648	3.155963183	EKRNRLKILLPFLEQ
Lsb1	GSC2	0.835917034	1.80738309	KSAAPEYTLRTRIWA
Lsb1	ORM1	0.908166568	2.387778867	PVTHRLRISIPGITG
Lsb1	COQ6	0.991037831	4.714743048	ERLSRLLLSLPPESF
Lsb1	SLH1	0.876841404	2.094282357	VSVLPGLQLRRIKDY
Lsb1	STE20	0.99811599	6.274353058	YWMAPEVVSRKEYGP
Lsb1	RIM101	0.932525543	2.696006168	QSKIPMLPSRRTMAG
Lsb1	KIC1	0.835917034	1.80738309	QYKSPSNVPRRLTVS
Lsb1	IMP3	0.996349278	5.612830456	SLLPPTDPFRRKHEQ
Lsb1	SNP1	0.99811599	6.274353058	RNYAPRLPRRETSSS
Lsb1	PRK1	0.999616496	7.86615938	KDKSRPPRPPPKPLH
Lsb1	UBP7	0.833065933	1.79015635	SWKPPDLPIRLRKRP
Lsb1	AIM21	0.99811599	6.274353058	PAGTPNVPTRRPILK
Lsb1	VTC4	0.99811599	6.274353058	SFYNRTAFQLPGDAR
Lsb1	IME2	0.876841404	2.094282357	WYRSPEILLRSGYYS
Lsb1	PBS2	0.876841404	2.094282357	SLSARRAVKLPPGGM
Lsb1	ECM25	0.833065933	1.79015635	ALMDRLVMKLPQAPY
Lsb1	ACF4	0.999616496	7.86615938	TSGPPLLPPRNTMKN
Lsb1	PRR1	0.833065933	1.79015635	LFEDRLPFDPPPNAS
Lsb1	FAS1	0.833065933	1.79015635	KLIGRPPLLVPGMTP
Lsb1	TRK2	0.833065933	1.79015635	RTSSRASLALPFQLR
Lsb1	SPA2	0.833065933	1.79015635	LDAPRPPLPQPMKQE
Lsb1	ACF2	0.997153497	5.861664125	NRGPPPLPPRANVQP
Lsb1	PEX13	0.833065933	1.79015635	SESAPEVLPRPAALN
Lsb1	ORM2	0.908166568	2.387778867	GITHRLRISIPGITG
Lsb1	TUS1	0.957402648	3.155963183	VKERRPPPPPPLLYS
Lsb1	POB3	0.999616496	7.86615938	ANQARKPFLLPATEL
Lsb1	CAC2	0.99811599	6.274353058	GVKNRPAIRIPSLKK
Lsb1	GYL1	0.999616496	7.86615938	RITSPPLPPRADCIE
Lsb1	ROT1	0.833065933	1.79015635	YLAYRPPMMLPTETL
Lsb1	BUL1	0.876841404	2.094282357	FPPKRKPLLRPQRSD
Lsb1	SLM2	0.996349278	5.612830456	NFVPPNLPMRRFKEI
Lsb1	PMS1	0.932525543	2.696006168	DYYNRQELALPKRMC
Lsb1	APP1	0.999616496	7.86615938	RVAPPPLPNRQLPNL
Lsb1	YAF9	0.833065933	1.79015635	ILMSRPGNLLPSNKT
Lsb1	INN1	0.996349278	5.612830456	QPPLPPIPTRDDMSN
Lsb1	MSB3	0.820753441	1.718992998	LDLVPDDDNRQRVEL
Lsb1	CLA4	0.876841404	2.094282357	PSPSPLNPYRPHHNM
Lsb1	NBA1	0.957402648	3.155963183	NDYNPTIPPRSKDRP
Lsb1	CUE5	0.996349278	5.612830456	EEEHPPLPARRKSEE
Lsb1	ASE1	0.932525543	2.696006168	IKTIPDLYIRNAILL
Lsb1	TCB1	0.932525543	2.696006168	RFVSPRIPSRIVKNG
Lsb1	AZF1	0.999616496	7.86615938	SNSMRPPLLIPAATT
Lsb1	ISN1	0.99811599	6.274353058	LGQSRLNLLVPSIGT
Lsb1	LAS17	0.999255819	7.203226043	RLPAPPPPPRRGPAP
Lsb1	MCA1	0.995939888	5.506544722	MAYNRPVYPPPQFQQ
Lsb1	SCD5	0.833065933	1.79015635	QHLPPPPPPRAQQQQ
Lsb1	MIP1	0.835917034	1.80738309	SFPLPPLQGRSLDEH
Lsb1	VTC3	0.932525543	2.696006168	ISGAPTLRLRWIGKL

Table S12

SH3 domain	Predicted gene name	Probability score	Rescaled score	Best PWM match
Lsb1	GYP5	0.999616496	7.86615938	TTSSPPLPPRQNVAT
Lsb1	KAR9	0.835917034	1.80738309	LSSVPPLPYDETDET
Lsb1	MDL2	0.835917034	1.80738309	LAKLPSIRFRSLVTP
Lsb1	TIP41	0.996349278	5.612830456	PTAAPATPPRHICNN
Lsb1	SEC8	0.996349278	5.612830456	TNNAPTLPKRKNPKI
Lsb1	GRS2	0.820753441	1.718992998	NDGFPAILKRISQAL
Lsb1	BSP1	0.999616496	7.86615938	SSSPPPLPTRRDHIK
Lsb3	SLA1	0.995939888	5.506544722	EGPPPAMPARPTATT
Lsb3	AIM3	0.99811599	6.274353058	ERAVPILPPRNNVEP
Lsb3	AGP2	0.932525543	2.696006168	QDSLPKLPFRSWGQP
Lsb3	ERT1	0.99811599	6.274353058	MNIQPDLPPRKIMIP
Lsb3	STP22	0.999998414	13.3540484	PHLKPPLPPPPPPQP
Lsb3	LEU2	0.864848328	2.001357636	LQHEPPLPIWSLDKA
Lsb3	SYP1	0.995939888	5.506544722	ASDTPPLPPHATPKN
Lsb3	ABP1	0.999992195	11.76074609	EEAAPQLPSRSSAAP
Lsb3	YET3	0.936954512	2.763898782	MFSILALPIPSRYRR
Lsb3	LDB17	0.864848328	2.001357636	AKAPPPPPPPPPSRK
Lsb3	CMR1	0.932525543	2.696006168	KSASPTLPTRRSRRL
Lsb3	YRF1-1	0.948492036	2.966018846	KLRSPNTPRRLRKTL
Lsb3	ZRG8	0.833065933	1.79015635	TGPPPPLPPPLFPSS
Lsb3	SAP1	0.833065933	1.79015635	APALPSLPPPPLLNV
Lsb3	ATG18	0.864848328	2.001357636	IGDQPALSPRRLRII
Lsb3	YBP2	0.999998414	13.3540484	VPIMPTLPPRPYITI
Lsb3	ROG1	0.999969822	10.40840039	SILLPPLPERAYIMD
Lsb3	RAD54	0.876841404	2.094282357	AGERPRLVPRPINVQ
Lsb3	GTS1	0.999700896	8.114717596	TTSTPPLPRRRATTS
Lsb3	MDS3	0.996349278	5.612830456	PLKLPPLPLTSDSNE
Lsb3	DIE2	0.602620488	0.922863506	IIVFPILVLRPIFLF
Lsb3	RIM101	0.932525543	2.696006168	PQILPPLPVGISQHL
Lsb3	SNP1	0.932525543	2.696006168	RNYAPRLPRRETSSS
Lsb3	PRK1	0.908166568	2.387778867	RKSMPPLSLTPVNND
Lsb3	UBP7	0.999998414	13.3540484	SWKPPDLPIRLRKRP
Lsb3	BNR1	0.957402648	3.155963183	VVKLPQLPPPPPPPP
Lsb3	PBS2	0.876841404	2.094282357	NKPLPPLPVAGSSKV
Lsb3	CDC6	0.995939888	5.506544722	FDDAPATPPRPLKRK
Lsb3	ECM25	0.864848328	2.001357636	FNAKPPLPIKAVTRP
Lsb3	ACF4	0.999998414	13.3540484	TSGPPLLPPRNTMKN
Lsb3	ACF2	0.999998414	13.3540484	NRGPPPLPPRANVQP
Lsb3	VRP1	0.833065933	1.79015635	TSHAPPLPPTAPPPP
Lsb3	MYO5	0.864848328	2.001357636	ANIPPPPPPPPPSSK
Lsb3	GYL1	0.999998414	13.3540484	RITSPPLPPRADCIE
Lsb3	SLM2	0.932525543	2.696006168	NFVPPNLPMRRFKEI
Lsb3	APP1	0.999998414	13.3540484	RVAPPPLPNRQLPNL
Lsb3	SRV2	0.876841404	2.094282357	PAPPPPPPAPPASVF
Lsb3	INN1	0.876841404	2.094282357	NYNQPPLPPIPTRDD
Lsb3	NBA1	0.876841404	2.094282357	KNELPSLPMLPSEAT
Lsb3	CUE5	0.999992195	11.76074609	EDVPPQLPTRTKSGE
Lsb3	TCB1	0.835917034	1.80738309	TSFYPALPVLTLEEI
Lsb3	LAS17	0.999992195	11.76074609	RGPAPPPPPRASRPT

Table S12

SH3 domain	Predicted gene name	Probability score	Rescaled score	Best PWM match
Lsb3	PUS1	0.932525543	2.696006168	LPKEPRLPKRKVAVM
Lsb3	GYP5	0.999998414	13.3540484	TTSSPPLPPRQNVAT
Lsb3	TIP41	0.932525543	2.696006168	PTAAPATPPRHICNN
Lsb3	SEC8	0.999992195	11.76074609	TNNAPTLPKRKNPKI
Lsb3	GRS2	0.820753441	1.718992998	NEEIPLLSRRLQESG
Lsb3	BSP1	0.999992195	11.76074609	SSSPPPLPTRRDHIK
Lsb4	SLA1	0.995939888	5.506544722	EGPPPAMPARPTATT
Lsb4	AIM3	0.999984454	11.07167998	ERAVPILPPRNNVEP
Lsb4	AGP2	0.99811599	6.274353058	QDSLPKLPFRSWGQP
Lsb4	ERT1	0.999923516	9.478432388	MNIQPDLPPRKIMIP
Lsb4	STP22	0.999984454	11.07167998	QDQAPSLPPKPNTQL
Lsb4	LEU2	0.820753441	1.718992998	HGSAPDLPKNKVNPI
Lsb4	SYP1	0.864848328	2.001357636	VKMSPSLPDEISQIV
Lsb4	ABP1	0.999996921	12.69075568	EEIAPSLPSRNSIPA
Lsb4	LDB17	0.864848328	2.001357636	PPPPPPPPSRKCGTP
Lsb4	CMR1	0.99811599	6.274353058	KSASPTLPTRRSRRL
Lsb4	SHS1	0.999255819	7.203226043	FLNSPDLPERTKLRN
Lsb4	ZRG8	0.957402648	3.155963183	TGPPPPLPPPLFPSS
Lsb4	SAP1	0.957402648	3.155963183	PTTAPALPSLPPPPL
Lsb4	SAP155	0.820753441	1.718992998	GNIIPQLPDNTTVLT
Lsb4	YBP2	0.999998414	13.3540484	VPIMPTLPPRPYITI
Lsb4	LSG1	0.957402648	3.155963183	APNEPLLPPLPGQPP
Lsb4	ROG1	0.999255819	7.203226043	SILLPPLPERAYIMD
Lsb4	GTS1	0.999992195	11.76074609	TTSTPPLPRRRATTS
Lsb4	MDS3	0.876841404	2.094282357	PLKLPPLPLTSDSNE
Lsb4	DIE2	0.602620488	0.922863506	IIVFPILVLRPIFLF
Lsb4	HUA1	0.864848328	2.001357636	LQSQPPRPPRPAANL
Lsb4	STE20	0.876841404	2.094282357	QTHAPTTPNRTSPNR
Lsb4	RIM101	0.99811599	6.274353058	QSKIPMLPSRRTMAG
Lsb4	SNP1	0.876841404	2.094282357	RNYAPRLPRRETSSS
Lsb4	UBP7	0.999996921	12.69075568	SWKPPDLPIRLRKRP
Lsb4	AIM21	0.932525543	2.696006168	SEVTPKVPERPSRRK
Lsb4	BBC1	0.957402648	3.155963183	PNTAPPLPRAPPVPP
Lsb4	TIM17	0.833065933	1.79015635	KPMAPPLPEAPSSQP
Lsb4	CDC6	0.995939888	5.506544722	FDDAPATPPRPLKRK
Lsb4	ECM25	0.864848328	2.001357636	FNAKPPLPIKAVTRP
Lsb4	ACF4	0.999998414	13.3540484	TSGPPLLPPRNTMKN
Lsb4	TPO1	0.833065933	1.79015635	RPYPPSLPSRDLYEV
Lsb4	ACF2	0.999998414	13.3540484	NRGPPPLPPRANVQP
Lsb4	VRP1	0.957402648	3.155963183	SVSAPPLPSASLPTH
Lsb4	MYO5	0.864848328	2.001357636	ANIPPPPPPPPSSK
Lsb4	GYL1	0.999998414	13.3540484	RITSPPLPPRADCIE
Lsb4	SLM2	0.99811599	6.274353058	NFVPPNLPMRRFKEI
Lsb4	APP1	0.999998414	13.3540484	RVAPPPLPNRQLPNL
Lsb4	INN1	0.99811599	6.274353058	NYNQPPLPPIPTRDD
Lsb4	CLA4	0.835917034	1.80738309	PSPSPLNPYRPHHNM
Lsb4	NBA1	0.932525543	2.696006168	KNELPSLPMLPSEAT
Lsb4	CUE5	0.999984454	11.07167998	EDVPPQLPTRTKSGE
Lsb4	TCB1	0.876841404	2.094282357	TSFYPALPVLTLEEI

Table S12

SH3 domain	Predicted gene name	Probability score	Rescaled score	Best PWM match
Lsb4	NUP1	0.957402648	3.155963183	TERPPLLPILPIQRL
Lsb4	LAS17	0.999992195	11.76074609	NRPLPQLPNRNNRPV
Lsb4	MKK1	0.996349278	5.612830456	RPAPPSLPSLSINSQ
Lsb4	PUS1	0.932525543	2.696006168	LPKEPRLPKRKVAVM
Lsb4	GYP5	0.999998414	13.3540484	TTSSPPLPPRQNVAT
Lsb4	TIP41	0.932525543	2.696006168	PTAAPATPPRHICNN
Lsb4	SEC8	0.999992195	11.76074609	TNNAPTLPKRKNPKI
Lsb4	BSP1	0.999984454	11.07167998	SSSPPPLPTRRDHIK
Myo3	AIM3	0.876841404	2.094282357	RSSPKKVPPVVPKKN
Myo3	SMP1	0.908166568	2.387778867	LKPPIGRPPKFPKSP
Myo3	OSH2	0.999992195	11.76074609	AAAPKHAPPPVPNET
Myo3	LDB17	0.999255819	7.203226043	AKAPPPPPPPPPSRK
Myo3	VPS64	0.932525543	2.696006168	TKTPETSPPKRPMGR
Myo3	MTH1	0.864848328	2.001357636	LKRPDMPPPSIIPRK
Myo3	ZRG8	0.99811599	6.274353058	GPPPPLPPPLFPSSS
Myo3	SAP1	0.999992195	11.76074609	PALPSLPPPPLLNVD
Myo3	PUF4	0.908166568	2.387778867	FPPPDFNDPTAPLSS
Myo3	MDS3	0.99811599	6.274353058	TREPNEPPPPCPAMS
Myo3	PTI1	0.99811599	6.274353058	MPMPMSTPPFIPLPL
Myo3	BUB1	0.833065933	1.79015635	QDLPSSQPPVVPKST
Myo3	STE20	0.932525543	2.696006168	KFIPSRPAPKPPSSA
Myo3	RIM101	0.996349278	5.612830456	STSPQILPPLPVGIS
Myo3	PRP8	0.932525543	2.696006168	LALPPPPPPPPGYEI
Myo3	PRK1	0.876841404	2.094282357	KSRPPRPPPKPLHLR
Myo3	UBP7	0.999992195	11.76074609	PTTPEIPPPLPPKIM
Myo3	BNR1	0.999616496	7.86615938	PPPPPPPPPPLPQSL
Myo3	AIM21	0.932525543	2.696006168	MVNPGQLPPSLEKKL
Myo3	BBC1	0.999992195	11.76074609	PAPPALSAPSVPPVP
Myo3	BCK1	0.999998414	13.3540484	IPSPSSSPPPIPKTA
Myo3	PTK2	0.957402648	3.155963183	IGSPPYTPPEVMYFD
Myo3	ACF4	0.996349278	5.612830456	ESTPTSGPPLLPPRN
Myo3	PAN3	0.99811599	6.274353058	NVPPPMQPPPIESSN
Myo3	RCN1	0.908166568	2.387778867	ISPPASPPPEFDFSK
Myo3	VRP1	0.999996921	12.69075568	PNAPLSPAPAVPSIP
Myo3	BDF1	0.996349278	5.612830456	MEVPKEPAPAPPPEP
Myo3	MYO5	0.961583486	3.259267849	ANIPPPPPPPPPSSK
Myo3	CAT8	0.99811599	6.274353058	AMSPLGAPPPPPHKD
Myo3	SSN8	0.820753441	1.718992998	TLWPEFIPPDPTKVT
Myo3	SLM2	0.835917034	1.80738309	FSTPLMSIPLVECTV
Myo3	SRV2	0.991037831	4.714743048	APAPPPPPPAPPASV
Myo3	BNI1	0.999616496	7.86615938	PPPPPPPPPPVPAKL
Myo3	CLA4	0.932525543	2.696006168	APKPPISAPRAPYPS
Myo3	STD1	0.864848328	2.001357636	LKRPDMPPPSLIPHG
Myo3	LAS17	0.999616496	7.86615938	ASAPTTPAPALPPAS
Myo3	BSP1	0.876841404	2.094282357	KNKPKPTPPSPPAKR
Myo5	SMP1	0.835917034	1.80738309	LKPPIGRPPKFPKSP
Myo5	OSH2	0.999998414	13.3540484	AAAPKHAPPPVPNET
Myo5	LDB17	0.999255819	7.203226043	AKAPPPPPPPPPSRK
Myo5	VPS64	0.908166568	2.387778867	TKTPETSPPKRPMGR

Table S12

SH3 domain	Predicted gene name	Probability score	Rescaled score	Best PWM match
Myo5	MTH1	0.864848328	2.001357636	LKRPDMPPPSIIPRK
Myo5	PKH3	0.864848328	2.001357636	YRQPTPSPPLPQMEF
Myo5	ZRG8	0.99811599	6.274353058	GPPPPLPPPLFPSSS
Myo5	SAP1	0.999700896	8.114717596	PALPSLPPPPLLNVD
Myo5	MDS3	0.932525543	2.696006168	TREPNEPPPPCPAMS
Myo5	PTI1	0.996349278	5.612830456	MPMPMSTPPFIPLPL
Myo5	STE20	0.908166568	2.387778867	KFIPSRPAPKPPSSA
Myo5	PRP8	0.99811599	6.274353058	DLALPPPPPPPPGYE
Myo5	UBP7	0.999984849	11.09745922	PTTPEIPPPLPPKIM
Myo5	BNR1	0.999996921	12.69075568	PPPPPPPPPPLPQSL
Myo5	AIM21	0.908166568	2.387778867	MVNPGQLPPSLEKKL
Myo5	BBC1	0.999984849	11.09745922	SAPPVPSAPSVPSAP
Myo5	BCK1	0.999992195	11.76074609	IPSPSSSPPPIPKTA
Myo5	ACF4	0.996349278	5.612830456	ESTPTSGPPLLPPRN
Myo5	PAN3	0.876841404	2.094282357	NVPPPMQPPPIESSN
Myo5	MPE1	0.876841404	2.094282357	PVPPVPLPFGIPPFP
Myo5	RCN1	0.932525543	2.696006168	ISPPASPPPEFDFSK
Myo5	ACF2	0.835917034	1.80738309	NRGPPPLPPRANVQP
Myo5	VRP1	0.999996921	12.69075568	SSMPAPPPPPPPPPG
Myo5	BDF1	0.996349278	5.612830456	PKEPAPAPPPEPDMN
Myo5	YTA12	0.833065933	1.79015635	RNIPPPPPPPPPKPP
Myo5	MYO5	0.999172096	7.096613123	ANIPPPPPPPPPSSK
Myo5	CAT8	0.999255819	7.203226043	AMSPLGAPPPPPHKD
Myo5	APP1	0.835917034	1.80738309	RRPPPPPIPSTQKPS
Myo5	SRV2	0.991037831	4.714743048	APAPPPPPPAPPASV
Myo5	BNI1	0.999255819	7.203226043	PPPPPPPPPPVPAKL
Myo5	STD1	0.864848328	2.001357636	LKRPDMPPPSLIPHG
Myo5	LAS17	0.999923516	9.478432388	ASAPTTPAPALPPAS
Nbp2	AIM3	0.932525543	2.696006168	ITPPRPPPSRSSPKK
Nbp2	MPS1	0.999998414	13.3540484	VFYHRPAPKPPVTKK
Nbp2	CRD1	0.835917034	1.80738309	ALLRRTIPKRPFYHV
Nbp2	SSY1	0.876841404	2.094282357	MSVQRKAPPVFEICS
Nbp2	PRP28	0.99811599	6.274353058	IKMSRSPPRPPSLKI
Nbp2	SPS1	0.835917034	1.80738309	SIRSRTPPSKLYSIQ
Nbp2	HIS1	0.876841404	2.094282357	VTPGRRAPTISKIDD
Nbp2	ILV1	0.864848328	2.001357636	AYLKRVAPHIKIIGV
Nbp2	BOI2	0.999255819	7.203226043	FVSPRRAPKPPSYPS
Nbp2	SHO1	0.957402648	3.155963183	SSKIRPTPRKPSRMA
Nbp2	RPS26B	0.932525543	2.696006168	DRKNRAPPQRPRFNR
Nbp2	RPS26A	0.932525543	2.696006168	DRKNRAPPQRPRFNR
Nbp2	GSC2	0.835917034	1.80738309	QCQQRPDPLPEGDFL
Nbp2	TEL2	0.833065933	1.79015635	TGESRKAPLIPLLKQ
Nbp2	TIF4631	0.833065933	1.79015635	HSFRRDAPPASKDSF
Nbp2	STE20	0.999984454	11.07167998	FIPSRPAPKPPSSAS
Nbp2	RIM101	0.957402648	3.155963183	YPSVRAAPSYSSSGC
Nbp2	PRK1	0.876841404	2.094282357	SRPPRPPPKPLHLRT
Nbp2	UBP7	0.991037831	4.714743048	RLRKRPPPPPPVSMP
Nbp2	AIM21	0.99811599	6.274353058	RPKRRAPPPVPKKPS
Nbp2	BCK1	0.999616496	7.86615938	LAPKREAPKPPANTS

Table S12

SH3 domain	Predicted gene name	Probability score	Rescaled score	Best PWM match
Nbp2	PBS2	0.999998414	13.3540484	LNPNRRAPRRPLSTQ
Nbp2	PTK2	0.876841404	2.094282357	GRSLRKRPTSPSISG
Nbp2	ACP1	0.833065933	1.79015635	RISSRVAPSAYRTIM
Nbp2	TUS1	0.833065933	1.79015635	VKERRPPPPPPLLYS
Nbp2	HSC82	0.876841404	2.094282357	DEETETAPEASTEAP
Nbp2	YPT53	0.833065933	1.79015635	AGQERFAPLAPMYYR
Nbp2	APP1	0.999996921	12.69075568	VRTRRRPPPPPIPST
Nbp2	CLA4	0.999998414	13.3540484	FQPQRTAPKPPISAP
Nbp2	NBA1	0.961583486	3.259267849	TYLTRPLPSTPNEDS
Nbp2	TRM10	0.833065933	1.79015635	EKVKRTPPLPPVPEG
Nbp2	SKM1	0.999998414	13.3540484	FRPHRLAPSAPATKN
Nbp2	MKK1	0.835917034	1.80738309	TMKKRPAPPSLPSLS
Nbp2	HAA1	0.833065933	1.79015635	DVNHRYPPMAPTTVA
Nbp2	BSP1	0.996349278	5.612830456	VVPERVKPAPPVSRS
Pex13	SLA1	0.833065933	1.79015635	GPPPAMPARPTATTE
Pex13	TEL1	0.833065933	1.79015635	TSYIEAIRPQWLSYP
Pex13	AIM3	0.99811599	6.274353058	GGQPPVPVRMQPQPP
Pex13	AGP2	0.999616496	7.86615938	DSLPKLPFRSWGQPY
Pex13	ERT1	0.876841404	2.094282357	NIQPDLPPRKIMIPI
Pex13	STP22	0.999996921	12.69075568	DQAPSLPPKPNTQLQ
Pex13	LEU2	0.820753441	1.718992998	QHEPPLPIWSLDKAN
Pex13	KAR4	0.833065933	1.79015635	YETPVKVRPGWVIVS
Pex13	ABP1	0.996349278	5.612830456	EAAPQLPSRSSAAPP
Pex13	NHP10	0.833065933	1.79015635	ERDPNMPKRPTNAYL
Pex13	RPT2	0.833065933	1.79015635	TLDPALIRPGRIDRK
Pex13	CDC48	0.833065933	1.79015635	QIDPAILRPGRLDQL
Pex13	RPO21	0.833065933	1.79015635	LPVPPPPVRPSISFN
Pex13	CMR1	0.996349278	5.612830456	SASPTLPTRRSRRLR
Pex13	MTC5	0.957402648	3.155963183	LDDPFTPPRWLHHIT
Pex13	PRP28	0.876841404	2.094282357	SRSPPRPPSLKIIDG
Pex13	MTH1	0.995939888	5.506544722	LLIVEKLRPEWGNQL
Pex13	SBE2	0.833065933	1.79015635	LKAVSHFRPSWLPPK
Pex13	RPT3	0.833065933	1.79015635	TLDPALLRPGRLDRK
Pex13	DIG2	0.820753441	1.718992998	TGLPPVPCYPYSSTP
Pex13	SAP1	0.998200276	6.320121922	PALPSLPPPPLLNVD
Pex13	KEG1	0.876841404	2.094282357	FLYAALLIRWLILMP
Pex13	PUF4	0.835917034	1.80738309	QPNPIMPGHPHNISS
Pex13	YBP2	0.999996921	12.69075568	PIMPTLPPRPYITIN
Pex13	LSG1	0.996349278	5.612830456	PLLPPLPGQPPLINI
Pex13	PRP43	0.833065933	1.79015635	IRTVTSVRPEWLIEI
Pex13	PEX14	0.999962608	10.19404889	AMPPTLPHRDWKDYF
Pex13	GTS1	0.876841404	2.094282357	TSTPPLPRRRATTSG
Pex13	MDS3	0.999419549	7.451704849	PSNPALPKKLLNVPY
Pex13	VPS62	0.833065933	1.79015635	GILLYNIRPKWLRSI
Pex13	DIE2	0.957402648	3.155963183	IVFPILVLRPIFLFN
Pex13	COS6	0.957402648	3.155963183	RNDGTIARPLFLVVL
Pex13	STE20	0.908166568	2.387778867	EEQPLPPIPPTKSKT
Pex13	RIM101	0.996349278	5.612830456	SKIPMLPSRRTMAGG
Pex13	ERG7	0.833065933	1.79015635	YSLPMHPGRWWVHTR

Table S12

SH3 domain	Predicted gene name	Probability score	Rescaled score	Best PWM match
Pex13	SBE22	0.833065933	1.79015635	LDVVSHSRPSWLPPK
Pex13	DCD1	0.833065933	1.79015635	KQMAPLMRPSWDSYF
Pex13	PRK1	0.999419549	7.451704849	SRPPRPPPKPLHLRT
Pex13	SSL2	0.833065933	1.79015635	LRPDHASRPLWISPS
Pex13	UBP7	0.999984849	11.09745922	WKPPDLPIRLRKRPP
Pex13	BNR1	0.992996959	4.9614108	PPPPPLPQSLLTEAE
Pex13	AIM21	0.999255819	7.203226043	EVTPKVPERPSRRKT
Pex13	BBC1	0.992996959	4.9614108	SAPPVPPAPPALSAP
Pex13	CDC6	0.833065933	1.79015635	DDAPATPPRPLKRKK
Pex13	ACF4	0.999616496	7.86615938	SGPPLLPPRNTMKNA
Pex13	SLD2	0.820753441	1.718992998	YYGPNSPLKLDEENI
Pex13	KTR2	0.833065933	1.79015635	IPAEDWNRPSWINET
Pex13	PET309	0.820753441	1.718992998	MARGSVPLKSVGSGL
Pex13	ACF2	0.999255819	7.203226043	RGPPPLPPRANVQPP
Pex13	CHS5	0.833065933	1.79015635	HNNIPIVRPEWVRAC
Pex13	SRC1	0.833065933	1.79015635	QTYKKFQRPIWLMFL
Pex13	HSC82	0.835917034	1.80738309	ETEPDLFIRITPKPE
Pex13	GYL1	0.996349278	5.612830456	ITSPPLPPRADCIEE
Pex13	ARK1	0.996349278	5.612830456	KTKPTPPPKPSHLKP
Pex13	SLM2	0.876841404	2.094282357	TSDPTIPAQLNVYDH
Pex13	APP1	0.999700896	8.114717596	VAPPPLPNRQLPNLD
Pex13	SRV2	0.935354115	2.738830825	APPPPPPAPPASVFE
Pex13	INN1	0.999984849	11.09745922	YNQPPLPPIPTRDDM
Pex13	RTT106	0.864848328	2.001357636	ASFLPVPEKPNLIYL
Pex13	CLA4	0.996349278	5.612830456	VAKPKKPARPTMSTA
Pex13	NBA1	0.876841404	2.094282357	DYNPTIPPRSKDRPR
Pex13	CUE5	0.876841404	2.094282357	EEHPPLPARRKSEEE
Pex13	LAS17	0.999700896	8.114717596	PVPPPPPMRTTTEGS
Pex13	WTM2	0.833065933	1.79015635	DVEGKPLRPKWIYQG
Pex13	RPT4	0.833065933	1.79015635	TLDPALLRPGRLDRK
Pex13	SMA1	0.833065933	1.79015635	RQFEEVPFRPWYFAM
Pex13	BRO1	0.864848328	2.001357636	GGPPLLPQKSAAFQS
Pex13	AFT2	0.833065933	1.79015635	CILPSTPTRPLSQSK
Pex13	SRP68	0.876841404	2.094282357	PKMLPIPSKPTLFDL
Pex13	GYP5	0.991037831	4.714743048	TSSPPLPPRQNVATS
Pex13	SEC8	0.957402648	3.155963183	NNAPTLPKRKNPKIF
Pex13	BSP1	0.999255819	7.203226043	EEKPLLPTRPNKAEV
Pin3	SEF1	0.991037831	4.714743048	GGITDRKLYPLPLYN
Pin3	AIM3	0.999962608	10.19404889	RAVPILPPRNNVEPP
Pin3	TAF5	0.833065933	1.79015635	LSSPARDILPLPPKT
Pin3	ERT1	0.957402648	3.155963183	NIQPDLPPRKIMIPI
Pin3	ABP1	0.833065933	1.79015635	AAQPPLPSRNVASGA
Pin3	RTN2	0.833065933	1.79015635	DRLPALEDRIRKLVF
Pin3	SHS1	0.833065933	1.79015635	LNSPDLPERTKLRNI
Pin3	MTC5	0.876841404	2.094282357	LDDPFTPPRWLHHIT
Pin3	DOP1	0.833065933	1.79015635	NYIPERALLPLSHSK
Pin3	PRP42	0.957402648	3.155963183	HLNFQRALLPLAHYD
Pin3	SAC7	0.957402648	3.155963183	LKRPPLSSRPYSYNT
Pin3	HIS1	0.833065933	1.79015635	NHLTDRLLFAIPKKG

Table S12

SH3 domain	Predicted gene name	Probability score	Rescaled score	Best PWM match
Pin3	VTC2	0.99811599	6.274353058	RTVYTRTAFQIPGDD
Pin3	YBP2	0.999255819	7.203226043	PIMPTLPPRPYITIN
Pin3	LSG1	0.835917034	1.80738309	PNEPLLPPLPGQPPL
Pin3	ITC1	0.876841404	2.094282357	EDEPVVARRSNSANV
Pin3	ROG1	0.991037831	4.714743048	ILLPPLPERAYIMDP
Pin3	VAS1	0.820753441	1.718992998	DNLPPVDPKTGEVII
Pin3	COQ6	0.991037831	4.714743048	NFPPVVALRTFGLNL
Pin3	HUA1	0.833065933	1.79015635	QSQPPRPPRPAANLA
Pin3	RIM101	0.957402648	3.155963183	VYSPQLSARLQTILP
Pin3	UBP7	0.995939888	5.506544722	WKPPDLPIRLRKRPP
Pin3	AIM21	0.957402648	3.155963183	EVTPKVPERPSRRKT
Pin3	VTC4	0.999998414	13.3540484	RSFYNRTAFQLPGDA
Pin3	MPM1	0.833065933	1.79015635	IDEYDNPLLPLPFNF
Pin3	PHO90	0.833065933	1.79015635	DLEYYRWPLPRPINL
Pin3	ACF4	0.999255819	7.203226043	SGPPLLPPRNTMKNA
Pin3	YRA2	0.999255819	7.203226043	REEPPLPKRIRISKI
Pin3	CSF1	0.999255819	7.203226043	DSPYHRCPFLLPLFY
Pin3	ACF2	0.999998414	13.3540484	RGPPPLPPRANVQPP
Pin3	MDM1	0.833065933	1.79015635	LEKYLRELLSISEIC
Pin3	GYL1	0.999984454	11.07167998	ITSPPLPPRADCIEE
Pin3	RPS10B	0.833065933	1.79015635	GVEYLREYLNLPEHI
Pin3	PMS1	0.999616496	7.86615938	SDYYNRQELALPKRM
Pin3	APP1	0.999816057	8.600886921	VAPPPLPNRQLPNLD
Pin3	INN1	0.991037831	4.714743048	PPLPPIPTRDDMSNY
Pin3	PUS4	0.991037831	4.714743048	LHEYAREGKPLPRAI
Pin3	CUE5	0.833065933	1.79015635	EEHPPLPARRKSEEE
Pin3	ASE1	0.957402648	3.155963183	KTIPDLYIRNAILLQ
Pin3	TCB1	0.957402648	3.155963183	FVSPRIPSRIVKNGW
Pin3	PNS1	0.833065933	1.79015635	NEKYERPPQPPPAYD
Pin3	LAS17	0.999172096	7.096613123	GPAPPPPPRASRPTP
Pin3	MCA1	0.995939888	5.506544722	NNGYQRPMAPPPNQQ
Pin3	RPS10A	0.833065933	1.79015635	GVEYLREYLNLPEHI
Pin3	SCD5	0.833065933	1.79015635	HLPPPPPPRAQQQQQ
Pin3	MIP1	0.876841404	2.094282357	FPLPPLQGRSLDEHF
Pin3	VTC3	0.996349278	5.612830456	RATYNRTAFQIPGDQ
Pin3	GYP5	0.999996921	12.69075568	TSSPPLPPRQNVATS
Pin3	GRS2	0.864848328	2.001357636	EEIPLLSRRLQESGE
Pin3	BSP1	0.998200276	6.320121922	SSPPPLPTRRDHIKI
Rvs167	AIM3	0.999998414	13.3540484	NAPERAVPILPPRNN
Rvs167	AGP2	0.996349278	5.612830456	NLKIRILPDIVNIAL
Rvs167	DEM1	0.999255819	7.203226043	DIKTRSVPKIPSIES
Rvs167	ERT1	0.999992195	11.76074609	LNFKRLLPRDPSEKS
Rvs167	LEU2	0.864848328	2.001357636	FMALQHEPPLPIWSL
Rvs167	ABP1	0.999616496	7.86615938	DEDEAAQPPLPSRNV
Rvs167	CMR1	0.996349278	5.612830456	RATKSASPTLPTRRS
Rvs167	MSN5	0.957402648	3.155963183	LWRPRIVPILPYITR
Rvs167	FIR1	0.833065933	1.79015635	PKKSRVLPPLPFPLY
Rvs167	YBP2	0.999962608	10.19404889	TLLRRLLYLMPESTS
Rvs167	ROG1	0.999255819	7.203226043	SASSILLPPLPERAY

Table S12

SH3 domain	Predicted gene name	Probability score	Rescaled score	Best PWM match
Rvs167	PEX14	0.957402648	3.155963183	YLYEAMPPTLPHRDW
Rvs167	RAD54	0.876841404	2.094282357	MARRRLPDRPPNGIG
Rvs167	GTS1	0.99811599	6.274353058	LPRRRATTSGPQPAI
Rvs167	PEF1	0.995939888	5.506544722	KPAGRPIPPAPTHYN
Rvs167	RIM101	0.996349278	5.612830456	STSPQILPPLPVGIS
Rvs167	SNP1	0.957402648	3.155963183	LLDRRLQNWNPNVDP
Rvs167	SLM1	0.991037831	4.714743048	RDPNFLLPNLPMRTF
Rvs167	UBP7	0.999996921	12.69075568	RLRKRPPPPPPVSMP
Rvs167	AIM21	0.957402648	3.155963183	RPKRRAPPPVPKKPS
Rvs167	MRS3	0.999255819	7.203226043	NNSTRPIPAIPMDLP
Rvs167	CDC6	0.995939888	5.506544722	SLNSAQVPLTPTTSP
Rvs167	RAD26	0.833065933	1.79015635	PEWRRPHPNIPDAKL
Rvs167	ACF4	0.999998414	13.3540484	TLPNRKPNPPPNRSQ
Rvs167	GRR1	0.833065933	1.79015635	DQLNRLLPNLPEEER
Rvs167	YRA2	0.833065933	1.79015635	LYREREEPPLPKRIR
Rvs167	BCH2	0.991037831	4.714743048	LAYERPLPDLPSTIK
Rvs167	TPO1	0.999255819	7.203226043	MGGDRPYPPSLPSRD
Rvs167	ACF2	0.999998414	13.3540484	GTTNRGPPPLPPRAN
Rvs167	VRP1	0.961583486	3.259267849	DDNVRPSPISPSINP
Rvs167	TUS1	0.833065933	1.79015635	VKERRPPPPPPLLYS
Rvs167	GYL1	0.999998414	13.3540484	ALKRITSPPLPPRAD
Rvs167	DSS1	0.820753441	1.718992998	ATKNRVLLRIPHKLP
Rvs167	SLM2	0.99811599	6.274353058	SSSNFPLNSIPKLDN
Rvs167	YDJ1	0.999255819	7.203226043	ILPPRIVPAIPKKAT
Rvs167	APP1	0.999998414	13.3540484	VRTRRRPPPPPIPST
Rvs167	INN1	0.999255819	7.203226043	SMAMRPIPPLPTESE
Rvs167	UTP23	0.991037831	4.714743048	RRKLRTVPGVPLIHL
Rvs167	CUE5	0.999255819	7.203226043	TQPVRKNPEAPARRR
Rvs167	STD1	0.820753441	1.718992998	SNLKRPDMPPPSLIP
Rvs167	LAS17	0.999998414	13.3540484	NRNNRPVPPPPPMRT
Rvs167	SCD5	0.961583486	3.259267849	QAEKRPSNGLGPPSV
Rvs167	GYP5	0.999998414	13.3540484	SETARKVPPIPTQII
Rvs167	SEC8	0.999992195	11.76074609	KKMLRNVNVLQHAYR
Rvs167	SYT1	0.833065933	1.79015635	FNGDRLVPTLPTVSR
Rvs167	BSP1	0.999998414	13.3540484	VVPERVKPAPPVSRS
Sho1	FUS1	0.999998414	13.3540484	LSRSKPLPLTPNSKY
Sho1	CYK3	0.999172096	7.096613123	ESMNNPLPPLPPLPD
Sho1	PCF11	0.908166568	2.387778867	KQLMKNLPKIPLLND
Sho1	SPS1	0.835917034	1.80738309	YELLKGLPPLSKYDP
Sho1	FIR1	0.833065933	1.79015635	PKKSRVLPPLPFPLY
Sho1	SAP1	0.876841404	2.094282357	APALPSLPPPPLLNV
Sho1	GIP2	0.99811599	6.274353058	KRRSKSLPTTPGIRS
Sho1	LSG1	0.996349278	5.612830456	APNEPLLPPLPGQPP
Sho1	SUT1	0.833065933	1.79015635	RNRDRSLPPLLLPNV
Sho1	STE20	0.999633677	7.911995497	KKEEQPLPPIPPTKS
Sho1	NAS2	0.833065933	1.79015635	KNEDRPLPVLLLREG
Sho1	PIG2	0.932525543	2.696006168	KRRSKSLPITPKSIF
Sho1	PRK1	0.908166568	2.387778867	NGKDKSRPPRPPPKP
Sho1	UBP7	0.998200276	6.320121922	RLRKRPPPPPPVSMP

Table S12

SH3 domain	Predicted gene name	Probability score	Rescaled score	Best PWM match
Sho1	AIM21	0.876841404	2.094282357	RPKRRAPPPVPKKPS
Sho1	PBS2	0.999998414	13.3540484	QIVNKPLPPLPVAGS
Sho1	BCH2	0.999172096	7.096613123	LAYERPLPDLPSTIK
Sho1	NFT1	0.932525543	2.696006168	ENENKKLPPAPTVFG
Sho1	PET309	0.820753441	1.718992998	MNKIDELPLLWKKLR
Sho1	KIN2	0.999172096	7.096613123	QKQNSDLPALPQNAE
Sho1	NHA1	0.833065933	1.79015635	DQDDEELPPLPVEAQ
Sho1	TUS1	0.999998414	13.3540484	HEREKALPPIPFTTT
Sho1	AVO2	0.833065933	1.79015635	YSNNSPLPVLPRRIS
Sho1	BUL1	0.835917034	1.80738309	IESDNSLPHDPPEIQ
Sho1	SFB2	0.833065933	1.79015635	PIRDLSLPPLPITIS
Sho1	INP52	0.833065933	1.79015635	HSTPKPLPPVPALSL
Sho1	DBP2	0.961583486	3.259267849	PNWDEELPKLPTFEK
Sho1	INN1	0.998200276	6.320121922	NYNQPPLPPIPTRDD
Sho1	AAD14	0.999992195	11.76074609	TDLFKPLPEPPTELG
Sho1	ABZ1	0.833065933	1.79015635	QSMDQLLPLLPLFDA
Sho1	NBA1	0.99811599	6.274353058	TYLTRPLPSTPNEDS
Sho1	RGA1	0.995939888	5.506544722	RRLKKNLPSLPTPVI
Sho1	LAS17	0.999998414	13.3540484	PQQNRPLPQLPNRNN
Sho1	MIP1	0.876841404	2.094282357	DPISFPLPPLQGRSL
Sho1	BEM3	0.833065933	1.79015635	HVPDLPLPTLPDRQL
Sho1	BSP1	0.996349278	5.612830456	SSSSSSPPPLPTRRD
Sla1-1-2	PKC1	0.999172096	7.096613123	RANAPLPPQPRKHDK
Sla1-1-2	AIM3	0.999172096	7.096613123	GSITPPRPPPSRSSP
Sla1-1-2	STP22	0.833065933	1.79015635	KPPLPPPPPPQPASN
Sla1-1-2	SYP1	0.995939888	5.506544722	LHIRAPALPPSRKQN
Sla1-1-2	ABP1	0.833065933	1.79015635	RSSAAPPPPPRRATP
Sla1-1-2	LDB17	0.999998414	13.3540484	APPPPPPPPPSRKCG
Sla1-1-2	ZRG8	0.833065933	1.79015635	VLNSPPLPPPARSQS
Sla1-1-2	PRP8	0.833065933	1.79015635	DLALPPPPPPPPGYE
Sla1-1-2	RRD1	0.833065933	1.79015635	IDETPPLPGPRRYGN
Sla1-1-2	UBP7	0.999996921	12.69075568	LRKRPPPPPPVSMPT
Sla1-1-2	BNR1	0.833065933	1.79015635	LPQLPPPPPPPPPPP
Sla1-1-2	ECM25	0.864848328	2.001357636	SELGCLTLPRSRSPS
Sla1-1-2	PTK1	0.833065933	1.79015635	LASSPPPPPPATHVP
Sla1-1-2	EAP1	0.833065933	1.79015635	ETSTPPPPPPGLIAH
Sla1-1-2	ACF2	0.833065933	1.79015635	TTNRGPPPLPPRANV
Sla1-1-2	VRP1	0.833065933	1.79015635	GAPAPPPPPPPPALG
Sla1-1-2	YTA12	0.833065933	1.79015635	NIPPPPPPPPPKPPL
Sla1-1-2	MYO5	0.999172096	7.096613123	ANIPPPPPPPPPSSK
Sla1-1-2	ARK1	0.833065933	1.79015635	KKTKPTPPPKPSHLK
Sla1-1-2	MSG5	0.833065933	1.79015635	VSPRPTPPSLSMRRS
Sla1-1-2	APP1	0.999969822	10.40840039	TRRRPPPPPIPSTQK
Sla1-1-2	INP52	0.961583486	3.259267849	LNVLPPPPPTSRHNK
Sla1-1-2	SRV2	0.833065933	1.79015635	AAPAPPPPPPAPPAS
Sla1-1-2	BNI1	0.833065933	1.79015635	DSPAPPPPPPPPPPP
Sla1-1-2	LAS17	0.999172096	7.096613123	VRLPAPPPPPRRGPA
Sla1-1-2	SCD5	0.991945222	4.821489883	PQHLPPPPPPRAQQQ
Sla1-1-2	LGE1	0.833065933	1.79015635	YHHRETPPPPPSNGY

Table S12

SH3 domain	Predicted gene name	Probability score	Rescaled score	Best PWM match
Sla1-1-2	TIM50	0.833065933	1.79015635	PDLLPPPPPPPYQRP
Sla1-1-2	BSP1	0.999172096	7.096613123	SSSSPPPLPTRRDHI
Sla1-3	AIM3	0.833065933	1.79015635	VGGQPPVPVRMQPQP
Sla1-3	MEC1	0.864848328	2.001357636	APSAMVVPVRKNLDI
Sla1-3	ABP1	0.833065933	1.79015635	SSAAPPPPPRRATPE
Sla1-3	OCA4	0.833065933	1.79015635	LCREPEVPQRLLTLI
Sla1-3	MPS1	0.9944892	5.201045387	NSPNQPINARETVEL
Sla1-3	UBX3	0.833065933	1.79015635	FKLITPVPRRELDLS
Sla1-3	CYK3	0.833065933	1.79015635	KVVIPPVPSRYSDER
Sla1-3	ACK1	0.820753441	1.718992998	INMFPPARARESSHK
Sla1-3	HEM14	0.833065933	1.79015635	EHGVPVVPSREVTIN
Sla1-3	GCD11	0.833065933	1.79015635	IVKTIPVPPRDFMIS
Sla1-3	CSE1	0.864848328	2.001357636	QIILPNVTLREEDVE
Sla1-3	KRE11	0.991037831	4.714743048	DYKIPVVPPRETYFA
Sla1-3	HUA1	0.999172096	7.096613123	LQSQPPRPPPRPAANL
Sla1-3	AIM21	0.932525543	2.696006168	SEVTPKVPERPSRRK
Sla1-3	MGA2	0.864848328	2.001357636	EVEKPGVACREEQSE
Sla1-3	RSM7	0.833065933	1.79015635	ALKSVPIPPRKNATL
Sla1-3	ACF2	0.833065933	1.79015635	QAIPPPVPNRPGGTT
Sla1-3	GYL1	0.833065933	1.79015635	RITSPPLPPRADCIE
Sla1-3	APP1	0.876841404	2.094282357	RVAPPPLPNRQLPNL
Sla1-3	LAS17	0.833065933	1.79015635	RLPAPPPPPRRGPAP
Sla1-3	LDB19	0.864848328	2.001357636	SQRLPGEPGREQAPN
Sla1-3	SCD5	0.833065933	1.79015635	QHLPPPPPPRAQQQQ
Sla1-3	GYP5	0.833065933	1.79015635	TTSSPPLPPRQNVAT
Sla1-3	CSR2	0.864848328	2.001357636	IIHPGPEPPRYDEIS
