# Supplementary material for: Bayesian Modeling of the Yeast SH3 Domain Interactome Predicts Spatiotemporal Dynamics of Endocytosis Proteins
Source: PLoS Biol. 2009 Oct 20;7(10):e1000218. doi: 10.1371/journal.pbio.1000218 (PMC2756588; doi:10.1371/journal.pbio.1000218)
Supplement: Table S13 — Protein dynamics and interaction scores for endocytosis proteins in yeast. The modular localization is represented for all proteins with characterized dynamics during endocytosis. The proteins are separated into their known endocytic modules. For each protein, the total predicted SH3-mediated interaction score (taken by summing all associated Bayesian probability scores) for every endocytic module (abbreviations for the respective modules are as follows: C, coat; W/M, WASP/Myo; S, scission; A, actin) is calculated. The time frame represents the point at which the protein appears (START) and is no longer observed (END) during endocytosis, with the lifetime taken as the difference between the two time points. Each protein was predicted to be part of the module for which it obtained the highest interaction score (highlighted in yellow). Cross-module proteins were considered to be proteins with interaction scores for a particular module that did not exceed the median interaction score across all modules by more than 2-fold. We could not predict the localization of Rvs167p because its corresponding scission module is only comprised of two proteins, itself and Rvs161p, which is not predicted to bind to any SH3 domain proteins required during endocytosis. To reduce the effect of weak interactions, predictions were only made for proteins that had module interactions scores of five and higher. Based on this criterion, the modular localization of some proteins could not be predicted (N/A). (0.03 MB PDF) [file pbio.1000218.s022.pdf]

Table S13

		Summed Interaction Score				Time frame			
Known localization	Protein	C	W/M	S	A	Start	END	Lifetime	Predicted localization
Coat	CLC1	-	-	-	-	-90	-5	85	N/A
Coat	CHC1	-	-	-	-	-90	-5	85	N/A
Coat	EDE1	0.51	-	-	-	-90	-15	75	N/A
Coat	ENT1	0.51	-	-	-	-35	-5	30	N/A
Coat	ENT2	0.51	-	-	-	-35	-5	30	N/A
Coat	SLA2	0.7	0.7	-	-	-35	-5	30	N/A
Coat	END3	-	0.51	-	-	-35	-5	30	N/A
Coat	PAN1	0.7	3.5	-	-	-35	-5	30	N/A
Coat	SLA1	8.12	29.15	0.7	8.24	-35	-5	30	WASP/Myo
Coat	GTS1	11.76	1.12	6.27	0.56	-25	-5	20	Coat
Coat	LSB5	-	0.51	-	-	-25	-5	20	N/A
WASP/Myo	LAS17	20.65	45.89	13.35	1.81	-35	-5	30	WASP/Myo
WASP/Myo	BZZ1	2.61	47.88	-	2.8	-25	-5	20	WASP/Myo
WASP/Myo	VRP1	5.65	38.98	3.26	3.16	-25	-5	20	WASP/Myo
WASP/Myo	BBC1	14.39	42.42	-	4.71	-15	-5	10	WASP/Myo
WASP/Myo	MYO3	1.77	36.98	-	3.21	-15	-5	10	WASP/Myo
WASP/Myo	MYO5	9.8	61.74	0.51	1.12	-15	-5	10	WASP/Myo
Actin	ABP1	16.83	10.94	7.87	31.62	-15	0	15	Actin
Actin	CAP1	-	1.12	-	-	-15	0	15	N/A
Actin	CAP2	-	-	-	-	-15	0	15	N/A
Actin	SAC6	-	-	-	-	-15	0	15	N/A
Actin	ACT1	-	-	-	-	-15	0	15	N/A
Actin	ARP3	-	-	-	-	-15	0	15	N/A
Actin	ARP2	-	-	0.7	-	-15	0	15	N/A
Actin	ARK1	1.79	0.7	-	11.07	-14	0	14	Actin
Actin	PRK1	1.26	3.21	-	13.35	-14	0	14	Actin
Actin	AIP1	-	-	-	-	-13	0	13	N/A
Actin	COF1	-	-	-	-	-13	0	13	N/A
Actin	SJL2	3.26	0.7	-	7.2	-10	0	10	Actin
Scission	RVS167	6.97	17.12	-	8.57	-12	-2	10	N/A
Scission	RVS161	-	-	-	-	-12	-2	10	N/A
N/A	SCD5	6.61	5.68	3.26	-	N/A	N/A	N/A	Coat
N/A	AIM21	6.1	24.47	3.16	7.87	N/A	N/A	N/A	WASP/Myo
N/A	SCP1	-	-	-	7.87	N/A	N/A	N/A	Actin
N/A	BSP1	18.17	7.5	13.35	7.2	N/A	N/A	N/A	Cross-module
N/A	LSB3	14.13	15.55	-	14.71	N/A	N/A	N/A	Cross-module
N/A	LSB4	18.29	20.08	-	13.25	N/A	N/A	N/A	Cross-module
N/A	TWF1	-	-	-	-	N/A	N/A	N/A	N/A
