# Supplementary material for: Bayesian Modeling of the Yeast SH3 Domain Interactome Predicts Spatiotemporal Dynamics of Endocytosis Proteins
Source: PLoS Biol. 2009 Oct 20;7(10):e1000218. doi: 10.1371/journal.pbio.1000218 (PMC2756588; doi:10.1371/journal.pbio.1000218)
Supplement: Text S1 — Supporting Materials and Methods . (0.05 MB DOC) [file pbio.1000218.s023.doc]

**Supporting Information for**

**Bayesian modeling of the yeast SH3 domain interactome predicts spatiotemporal dynamics of endocytosis proteins**

Raffi Tonikian^1,2*^, Xiaofeng Xin^1,2*^, Christopher P. Toret^3,+,*^, David Gfeller^1,2^, Christiane Landgraf^4^, Simona Panni^5,6^, Serena Paoluzi^5^, Luisa Castagnoli^5^, Bridget Currell^7^, Somasekar Seshagiri^7^, Haiyuan Yu^8^, Barbara Winsor^9^, Marc Vidal^8^, Mark B. Gerstein^10,11,12^, Gary D. Bader^1,2^, Rudolf Volkmer^4,#^, Gianni Cesareni^5,13,#^, David G. Drubin^3#^, Philip M. Kim^10, ‡,#^, ^14,‡,#^, Charles Boone^1,2#^.

1. Terrence Donnelly Center for Cellular and Biomolecular Research, Banting and Best Department of Medical Research, University of Toronto, Toronto, Ontario, Canada
2. Department of Molecular Genetics, University of Toronto, Toronto, Ontario, Canada
3. Department of Molecular and Cell Biology, University of California, Berkeley, Berkeley, California, United States of America
4. Institute of Medical Immunology, Charité-Universitätsmedizin Berlin, Berlin, Germany
5. Department of Biology University of Rome Tor Vergata, Rome, Italy
6. Department of Cell Biology, University of Calabria, Via P. Bucci 6C, 87036 Rende (CS)
7. Department of Molecular Biology, Genentech, South San Francisco, California, United States of America
8. Center for Cancer Systems Biology (CCSB) Department of Cancer Biology, Dana-Farber Cancer Institute and Department of Genetics, Harvard Medical School, 44 Binney Street, Boston, Massachusetts, USA.
9. CNRS et Université de Strasbourg UMR7156, Génétique moléculaire, Génomique et Microbiologie, 21 rue Descartes, 67084 Strasbourg, France
10. Department of Molecular Biophysics and Biochemistry, Yale University, New Haven, Connecticut, United States of America
11. Program in Computational Biology and Bioinformatics, Yale University, New Haven, Connecticut, United States of America
12. Department of Computer Science, Yale University, New Haven, Connecticut, United States of America
13. Research Institute "Fondazione Santa Lucia", Rome, Italy
14. Department of Protein Engineering, Genentech, South San Francisco, California, United States of America

* These authors contributed equally to this work

+ Current address: Department of Biological Sciences, Stanford University, Stanford, California, United States of America

‡ Current address: Terrence Donnelly Center for Cellular and Biomolecular Research, Banting and Best Department of Medical Research, University of Toronto, Toronto, Ontario, Canada

# To whom correspondence should be addressed. E-mail: [rve@charite.de](mailto:rve@charite.de) (RV); cesareni@uniroma2.it (GC); [drubin@berkeley.edu](mailto:drubin@berkeley.edu) (DGD); [pmkim@alum.mit.edu](mailto:pmkim@alum.mit.edu) (PMK); [sachdev.sidhu@utoronto.ca](mailto:sachdev.sidhu@utoronto.ca) (SSS); [charlie.boone@utoronto.ca](mailto:charlie.boone@utoronto.ca) (CB)

**SH3 domain boundary determination based on fungal species alignment**

The domain boundaries for 3 SH3 domains, namely Bem1-2, Bud14, and Sla1-1 were re-evaluated based on multiple sequence alignments of their respective homologues across several fungal species. The protein sequences used in the multiple sequence alignment were retrieved from the “Fungal Alignment” tool on the *Saccharomyces* Genome Database website using the default parameters and the sequence alignments were visualized using the Jalview software [1].

Intuitively, one would expect to see greater sequence homology in regions encoding orthologous SH3 domains and higher sequence divergence in adjacent, non-conserved regions. Aligning Bem1p orthologues from several fungal species revealed a highly conserved 50 amino acid extension C-terminal to the second SH3 domain, suggesting that this extended region may play a critical role in SH3 domain folding and/or stabilization. Indeed, cloning and expression of the extended construct resulted in the purification of soluble, stable GST fusion protein.

Analogously, it was found that there is virtually no demarcation between the two N-terminal SH3 domains in Sla1p, a feature that is conserved throughout the fungal species examined. A construct encoding the 2 SH3 domains in tandem resulted in the purification of a soluble, stable GST fusion protein, which we refer to as Sla1-1/2. To test the specificity of each domain separately, we specifically mutated a conserved Trp residue to Ser in the binding sites of each SH3 domain (W41 and W108, respectively). The tandem constructs with a mutant version of each SH3 domain were subsequently retested in the phage display analysis, which resulted in the selection of peptides that resembled the wild-type tandem SH3 domain construct. Furthermore, the wild-type and mutant construct behaved similarly in the SPOT assay as they bound to the same peptide ligands. We therefore concluded that the 2 N-terminal SH3 domains from Sla1p have identical binding specificities.

**Design of a proline rich peptide array representing the yeast proteome**

In order to compile a list of yeast peptides containing most of the possible SH3 domain targets, a collection of regular expressions were derived that matched most of the SH3 domain specificities described in this study and our previous analysis [2]. The stringency of the regular expression was set somewhat arbitrarily as a compromise between the ambition to avoid missing putative ligand peptides weakly matching a strict consensus and the limits in the total number of peptides to be probed, imposed by the experimental techniques. Below is the list of regular expressions used to identify putative ligands in the yeast proteome, where “.” represents any amino acid and brackets represent multiple residues allowed at a unique position, respectively:

...[RK].[AVLIMFHRTP]P..P.....

......P.[ILMVPYAFTR]P.[RKW]...

...[KR][KR][AHPKRLG]P[PLV].......

..[KRP]......P.[KR]P..

..[KRP]....P...[KR]P..

.[FPLWA].[WYLMFHP].[AVLIMFHPR]P..P.....

[FPLWA]..[WYLMFHP].[AVLIMFHPP]P..P.....

..R..[FLIYM].[FLIYM]P.....

….P.R.A[VP]…..

....RP[AS]....Y...

.....[GP]P.[IVL].P[FWY]...

.....P.R.P.R...

......PR.P.R...

..R[YFLEP]..[AVLIMFHWRTP]P[GSDLIAV]P.....

....RP[AVLIMFHRKTP][AVLIMFHRTP][AVLIMFHRTP]..[LP]...

We next designed a python script that searches all the 15mers in the yeast proteome for peptides matching any of the regular expressions. Peptides matching more than one regular expression are represented only once in the list. Finally all the 15 amino acid long peptides containing 4 or more prolines between position 3 and 12 were also included.

This strategy retrieved a total of 2,953 predicted peptides (Supplementary Table S6) that were synthesized on cellulose membrane by the SPOT technique (see Materials and Methods). This array was probed with 26 SH3 domains and the 295 peptides binding to at least one of the domains were re-synthesized on new smaller membranes together with normalization controls (peptide LASDLIVPRR that reacts with the anti-GST antibody). The different experiments were normalized by setting to 100 the value for the average of the control peptides. The values for the 295 peptides tested against 26 SH3 domains are listed in Supplementary Table S7.

**Redundancy training for Bayesian model**

A potential issue for interaction predictors is that predictions tend to be better for interactions that are more similar to the ones contained in the training set. This is particularly true for predictors based on specificity. Since our predictor is merely an integration of 3 different experimental results it is unlikely to be affected by such issues. Nonetheless, we performed a redundancy reduction to rule out any such possibility. To this end, we removed all domains from our set that were similar in binding profile to another domain. We then re-trained our Bayesian model and assessed its accuracy by tenfold cross-validation as before. As can be seen in Supplementary Figure S4, the accuracy remains very high.

**Supplementary References**

1. Cherry JM, Ball C, Weng S, Juvik G, Schmidt R et al. (1997) Genetic and physical maps of Saccharomyces cerevisiae. Nature 387(6632 Suppl): 67-73.

2. Tong AH, Drees B, Nardelli G, Bader GD, Brannetti B et al. (2002) A combined experimental and computational strategy to define protein interaction networks for peptide recognition modules. Science (New York, NY 295(5553): 321-324.

3. Kaksonen M, Toret CP, Drubin DG (2005) A modular design for the clathrin- and actin-mediated endocytosis machinery. Cell 123(2): 305-320.

4. Zarrinpar A, Park SH, Lim WA (2003) Optimization of specificity in a cellular protein interaction network by negative selection. Nature 426(6967): 676-680.
